# Merging Enzymatic Catalysis with Iron Catalysis for Highly Stereoselective Heparan Sulfate Oligosaccharide Assembly

**DOI:** 10.1021/jacs.5c19067

**Published:** 2025-12-22

**Authors:** Dakang Zhang, Zixiang Jiang, Le Yin, Xiao-Wen Zhang, Haoyu Yang, Pinzhi Wang, Changmin Xie, Eduardo Stancanelli, Yongmei Xu, Haoran Wang, Junjiang Sun, Jian Liu, Hao Xu

**Affiliations:** † Department of Chemistry, 427969 Brandeis University, 415 South Street, Waltham, Massachusetts 02453, United States; ‡ Division of Chemical Biology and Medicinal Chemistry, Eshelman School of Pharmacy, 15521 University of North Carolina, Chapel Hill, North Carolina 27599, United States; § 680030 Glycan Therapeutics, 617 Hutton St, Raleigh, North Carolina 27606, United States

## Abstract

Heparan sulfate (HS) regulates numerous biological processes, but it occurs naturally as a heterogeneous mixture of sulfated complex glycans. Therefore, pursuing a broadly effective approach for homogeneous HS synthesis to advance biological studies has been an outstanding challenge in glycoscience. By merging the catalytic power of sulfotransferases (SULTs) with two distinct iron-catalyzed glycosylation reactions, we report herein a highly stereoselective and generally applicable HS assembly strategy. Every glycosidic linkage in HS was assembled by one of these iron-catalyzed, entirely stereoselective glycosylation reactions. An array of sulfate groups that are essential to HS’s function were installed at their desired locations via the SULT-controlled sulfation of HS precursors. This general approach is showcased in the synthesis of an anticoagulant HS hexasaccharide and other full-length precursors of HS octasaccharides.

## Graphical abstract

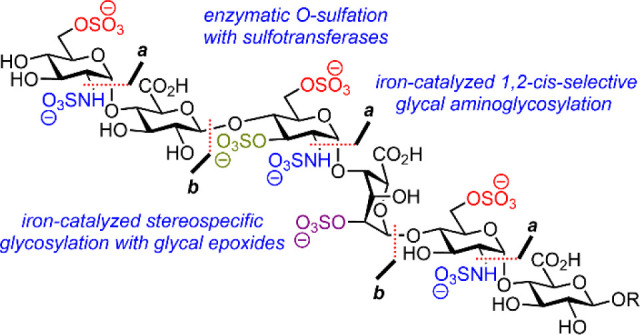

## Introduction

Heparan sulfate (HS) is a class of sulfated complex glycosaminoglycans (GAGs) that mediate essential biological processes at the cell surface and in the extracellular matrix.^1–4^ It regulates cell growth and proliferation, immune response, inflammation, and other physiological phenomena through selective HS–protein interactions.^5,6^ The naturally isolated HS exists as a heterogeneous mixture of complex glycans with a variety of lengths and sulfation patterns (Figure 1A);^7,8^ therefore, the limited availability of homogeneous HS represents a major roadblock that hampers the study of its important biological functions, which inspired the development of enabling enzymatic and chemical syntheses of HS.

**Figure 1:**
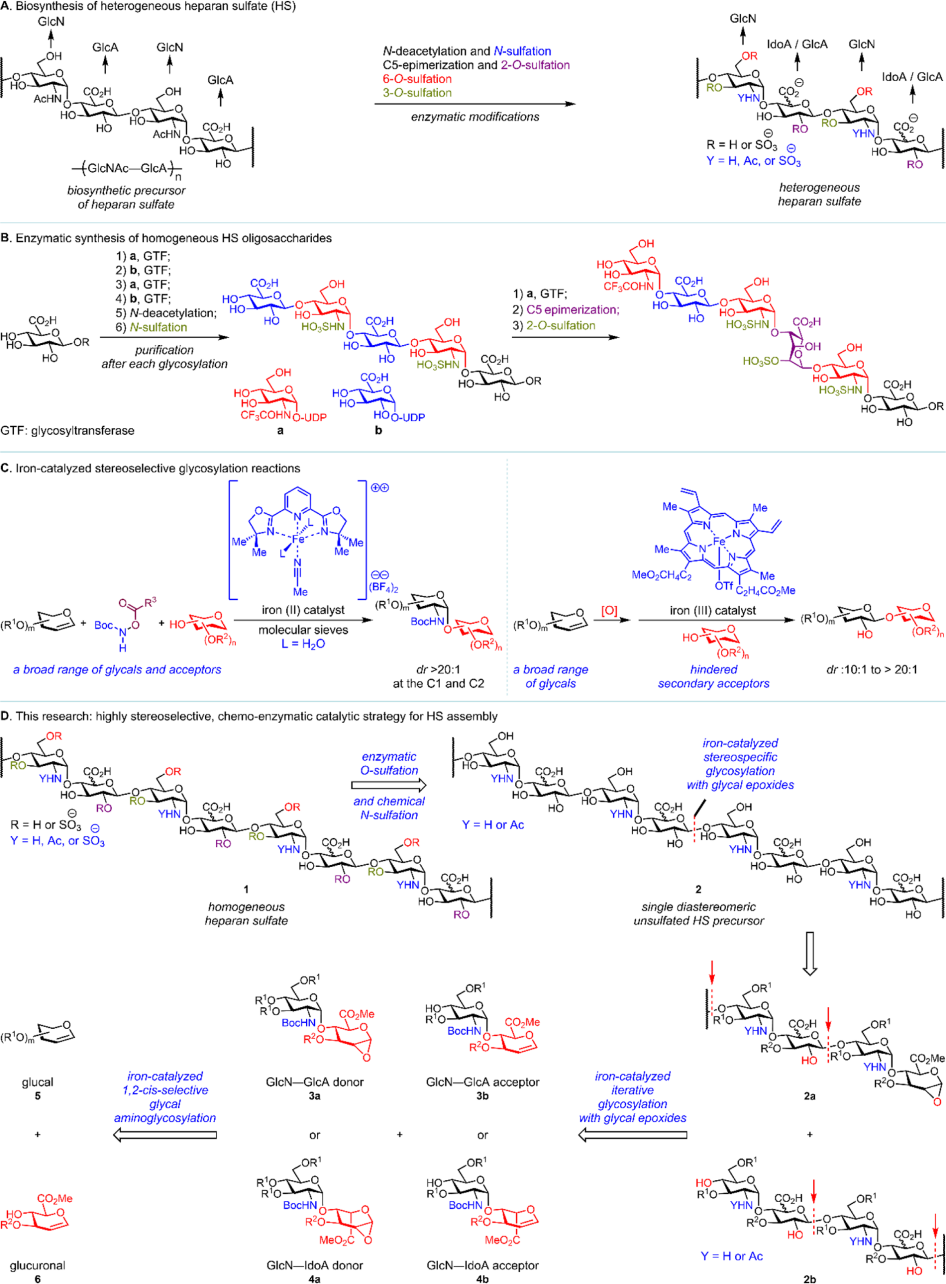
(A) Biosynthesis of heterogeneous heparan sulfate (HS). (B) Enzymatic synthesis of homogeneous HS oligosaccharides. (C) The iron-catalyzed 1,2-*cis-*selective glycal aminoglycosylation and the iron-catalyzed stereospecific glycosylation with glycal epoxides. (D) This research: highly stereoselective, chemo-enzymatic catalytic strategy for HS assembly.

The HS backbone is composed of two repeating disaccharide units: glucosamine (GlcN)-α-1,4-glucuronic acid (GlcA) and GlcN-α-1,4-iduronic acid (IdoA), both of which are difficult to synthesize in high stereoselectivity (Figure 1A).^9^ Site-selective sulfation at the *N*-, 6-*O*-, and 3-*O* positions of GlcNs and at the 2-*O*-position of IdoAs further adds to its structural complexity (Figure 1A).^7^ Enzymatic HS synthesis leverages the high selectivity of glycosyltransferases (GTFs) and sulfotransferases (SULTs);^10–13^ however, GTFs can extend the HS backbone with sugar nucleotides only one at a time and they are incompatible with rare and complex sugar substrates (Figure 1B).^14^ Additionally, enzymatic conversion of GlcAs to rare IdoAs is tedious: only the 2-*O*-sulfated IdoA (IdoA2S) can be obtained in high yield and this requires the cooperative catalytic activities of both *C5*-epimerases and 2-*O*-sulfotransferases.^15^ This could lead to the generation of 2-*O*-sulfated GlcA (GlcA2S)-containing byproducts,^15^ which often requires tedious purification that adds to the difficulty for process development of large-scale HS synthesis.

Valuable chemical glycosylation methods have also been developed, which facilitate an array of elegant syntheses of HS oligosaccharides (Figure 2).^16–40^ Most notably among these methods include: (a) 1,2-*cis*-selective glycosylation of 1,6-anhydro-l-idopyranoses discovered by Hung;^20–22^ (b) 1,2-*cis*-selective glycosylation of glucuronic and iduronic ester acceptors locked in ^1^C_4_ conformations reported by Seeberger;^23,24^ (c) modular and reiterative glycosylation approach developed by Boons;^25–27^ (d) preactivation-based, one-pot glycosylation approach reported by Huang;^28–31^ (e) anomeric reactivity-based, one-pot glycosylation discovered by Wong;^32,33^ (f) dehydrative glycosylation approach developed by van der Marel;^34^ and (g) GAG degradation for building block synthesis reported by Hsieh-Wilson.^35,36^ These syntheses demonstrate the power of chemical glycosylation; however, the reactivity and stereoselectivity of chemical glycosylation often vary with substrates, so it is difficult to develop a highly stereoselective glycosylation method that is also broadly effective. Thus, pursuing a broadly effective approach for HS synthesis has been an outstanding challenge in glycoscience.

**Figure 2:**
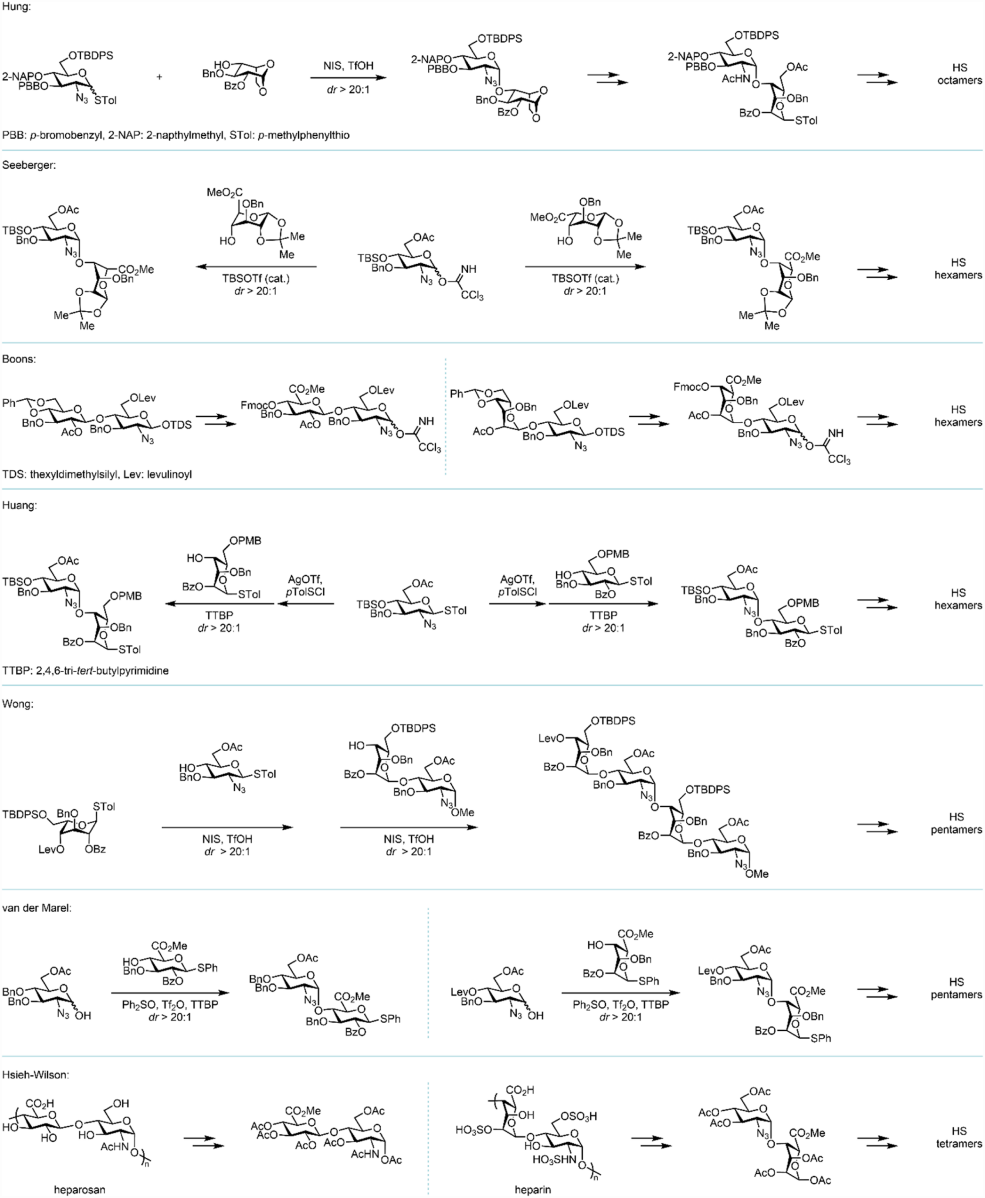
Selected glycosylation methods and strategies for chemical syntheses of HS oligosaccharides.

To emulate these enabling glycosylation approaches, we have recently developed two iron-catalyzed stereoselective glycosylation methods that are effective for a broad range of substrates: (a) an iron-catalyzed, exclusively 1,2-*cis*-seletive glycal aminoglycosylation^41–44^ and (b) an iron-catalyzed highly stereospecific glycosylation with glycal epoxides (Figure 1C).^45^

We envisioned a stereoselective HS assembly strategy by merging the catalytic power of multiple sulfotransferases (SULTs) with these two distinct iron-catalyzed glycosylation reactions. We hypothesized that every glycosidic linkage in HS could be assembled via one of these iron-catalyzed, entirely stereoselective glycosylation reactions. We predicted that a variety of *O*-sulfate groups that are critical to HS’s functions could be directly installed at their desired locations via the SULT-controlled sulfation of the HS precursors (Figure 1D). Herein, we report the development of a generally applicable and highly stereoselective, chemo-enzymatic catalytic strategy for HS assembly. This approach is showcased in the synthesis of an anticoagulant HS hexasaccharide^12^ and other full-length precursors of HS octasaccharides that were previously difficult to assemble.

## Results and Discussion

We envisioned that a homogeneous HS **1** could be derived from its precursor **2** by chemical *N*-sulfation followed by SULT-controlled, site-selective *O*-sulfation (Figure 1D). We hypothesized that **2** could be synthesized via the iron-catalyzed stereospecific glycosylation of **2b** with glycal epoxide **2a**,^45^ both of which could be assembled via the iron-catalyzed iterative glycosylation of the GlcN–GlcA module (**3a**/**b**) and the GlcN–IdoA module (**4a**/**b**). Notably, this iterative process should be entirely stereoselective in forging all the GlcA-β-1,4-GlcN and IdoA-α-1,4-GlcN linkages. Since limited methods are effective in activating GlcA-based donors for complex-glycan synthesis,^9,46,47^ the iron-catalyzed stereospecific glycosylation with glycal epoxides should be fit for this difficult transformation: this method is particularly effective for previously problematic sterically hindered glycosyl acceptors and electron-deficient glucuronic ester epoxide^45,46,48,49^ donors.

Furthermore, we hypothesized that both disaccharide modules (GlcN-α-1,4-GlcA (**3b**) and GlcN-α-1,4-IdoA (**4b**)) could be prepared via the iron-catalyzed, exclusively 1,2-*cis*-selective aminoglycosylation of glucuronal **6** with glucal **5** (Figure 1D).^41^ The vast majority of the existing 1,2-*cis*-selective aminoglycosylation methods leverage non-neighboring-participating groups to achieve the high *cis*-selectivity.^50^ These valuable methods are most effective for substrates with specific substituents or conformations, but structural variations often lead to decreased *cis*-selectivity.^23,25,29,51–59^ This difficult glycosylation would require that glucuronal **6** does not function as a donor but rather behaves as an acceptor, such that the iron catalyst could simultaneously activate the C4-OH of **6** and an amination reagent when it transfers both moieties to glucal **5** in an exclusively *cis*-selective manner.^41^ However, in our previous study, the hindered, electron-deficient C4-OH glucuronals or iduronals had not been successfully applied as glycosyl acceptors. We reasoned that use of an electron-rich glucal **5** as the donor and an electron-deficient glucuronal **6** as the acceptor would allow for selective nitrogen atom transfer to the electron-rich glucal **5** while leaving the electron-deficient olefin moiety in glucuronal **6** intact, so that it could be activated in the next glycosylation.

We selected glucal **5a** and an array of glucuronals (**6a**–**6c**) for the iron-catalyzed glycal *cis*-aminoglycosylation (Figure 3). These glucuronals can be synthesized from readily available **7** via decagram-scale, chemo-enzymatic reactions (Figure 3B). Amano Lipase selectively deacetylates the more reactive allylic C3-OAc group of **7**. After allylation (or benzylation), the C4-OH group was liberated with K_2_CO_3_ in MeOH to give **6a** and **6b**. Alternatively, dideacetylation of **7** followed by selective enzymatic acetylation of allylic C3-OH affords **6c**.

**Figure 3:**
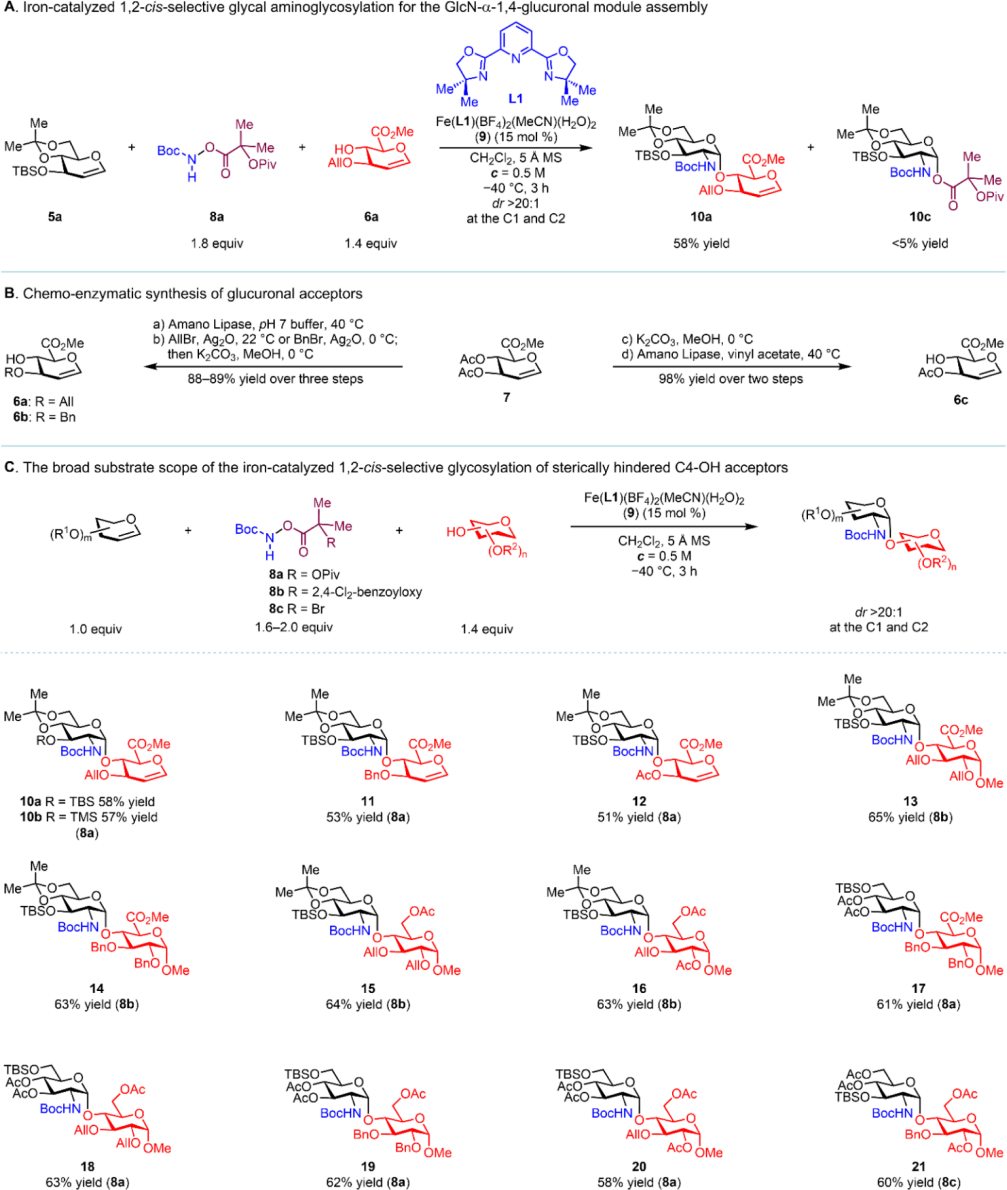
(A) Assembly of the GlcN-α-1,4-glucuronal module via the iron-catalyzed exclusively 1,2-*cis*-selective glycal aminoglycosylation. (B) Chemo-enzymatic synthesis of glucuronal acceptors. a) Amano Lipase (20 wt %), phosphate buffer (pH = 7)/acetone 3:1, 40 °C. b) AllBr or BnBr (4.0 equiv), Ag_2_O (2.5 equiv), toluene, 5 Å molecular sieves, 0 or 22 °C; then K_2_CO_3_ (0.2 equiv), MeOH, 0 °C. c) K_2_CO_3_ (0.2 equiv), MeOH, 0 °C. d) Amano Lipase (30 wt %), vinyl acetate (2.0 equiv), THF, 40 °C. See Supporting Information for details. (C) The broad substrate scope of the iron-catalyzed 1,2-*cis*-selective glycal aminoglycosylation of sterically hindered C4-OH acceptors. The glycosylation was carried out in CH_2_Cl_2_ at −40 °C for 3 h. All yields are isolated yields. See Supporting Information for details.

We discovered that the iron catalyst, Fe­(**L1**)­(BF_4_)_2_(MeCN)­(H_2_O)_2_ (**9**),^60^ is uniquely effective in promoting this exclusively 1,2-*cis*-selective aminoglycosylation of **6a** with glucal **5a** in the presence of amination reagent **8a**, affording the desired 1,2-*cis*-aminoglycoside **10a** on multigram scale (Figure 3A, 58% yield, *dr* > 20:1). It is worth noting that the hindered *O*-acyl activating group in **8a** is optimal for suppressing the competing glycal 1,2-*cis*-aminoacyloxylation^61,62^ (**10c**, <5% yield). The major byproduct is 1,2-*cis*-aminoglycosyl fluoride **s4** (10–15% yield), which is presumably due to the competing fluoride ion transfer from the tetrafluoroborate counteranion (Figure S3c).^41^ Glycosylation of glucuronals **6b** and **6c** with glucal **5a** similarly affords the desired 1,2-*cis*-aminoglycosides **11** (53% yield) and **12** (51% yield) as single diastereomers on multigram scale (Figure 3C).

To evaluate the generality of this method, we explored a variety of glucuronic ester and glucose-derived, hindered C4-OH acceptors: all of these electronically distinct acceptors can be readily glycosylated and converted to 1,2-*cis*-aminoglycosides in decent yields (**13**–**16**, *dr* > 20:1 in Figure 3C). Furthermore, electron-deficient glycals (**S11** and **S14**) with two electron-withdrawing acetyl groups and a single electron-donating silyl group are both excellent donors in this entirely 1,2-*cis*-selective glycosylation (corresponding products **17**–**21** in Figure 3C). Notably, amination reagent **8c** is uniquely effective for glycosylation with 4,6-di-*O*-acetyl-3-*O*-TBS-d-glucal (**S14**) (product **21** in Figure 3C).

However, direct glycosylation of iduronal **22** with glucal **5a** to afford the desired GlcN-α-1,4-iduronal linkage proved challenging, providing **23** in only 25% yield (*dr* > 20:1) along with **10c** (20% yield, *dr* > 20:1) and **25** (36% yield based on **22**), in which **22** acts as both the glycosyl donor and acceptor (Scheme 1A). Iduronal **22** predominantly adopts the ^5^H_4_ conformation (estimated >96%);^63^ therefore, coordination of the iron catalyst **9** with the pseudoaxial C4-OH of ^5^H_4_ conformer of **22** and the subsequent catalyst oxidation would generate a reactive iron-nitrenoid or iron iminyl radical species **24**.^64^ **24** could readily promote the proximity-induced, nitrogen atom transfer and cyclization, affording the undesired 1,4-anhydrosugar **25** (Scheme 1A).

**Scheme 1:**
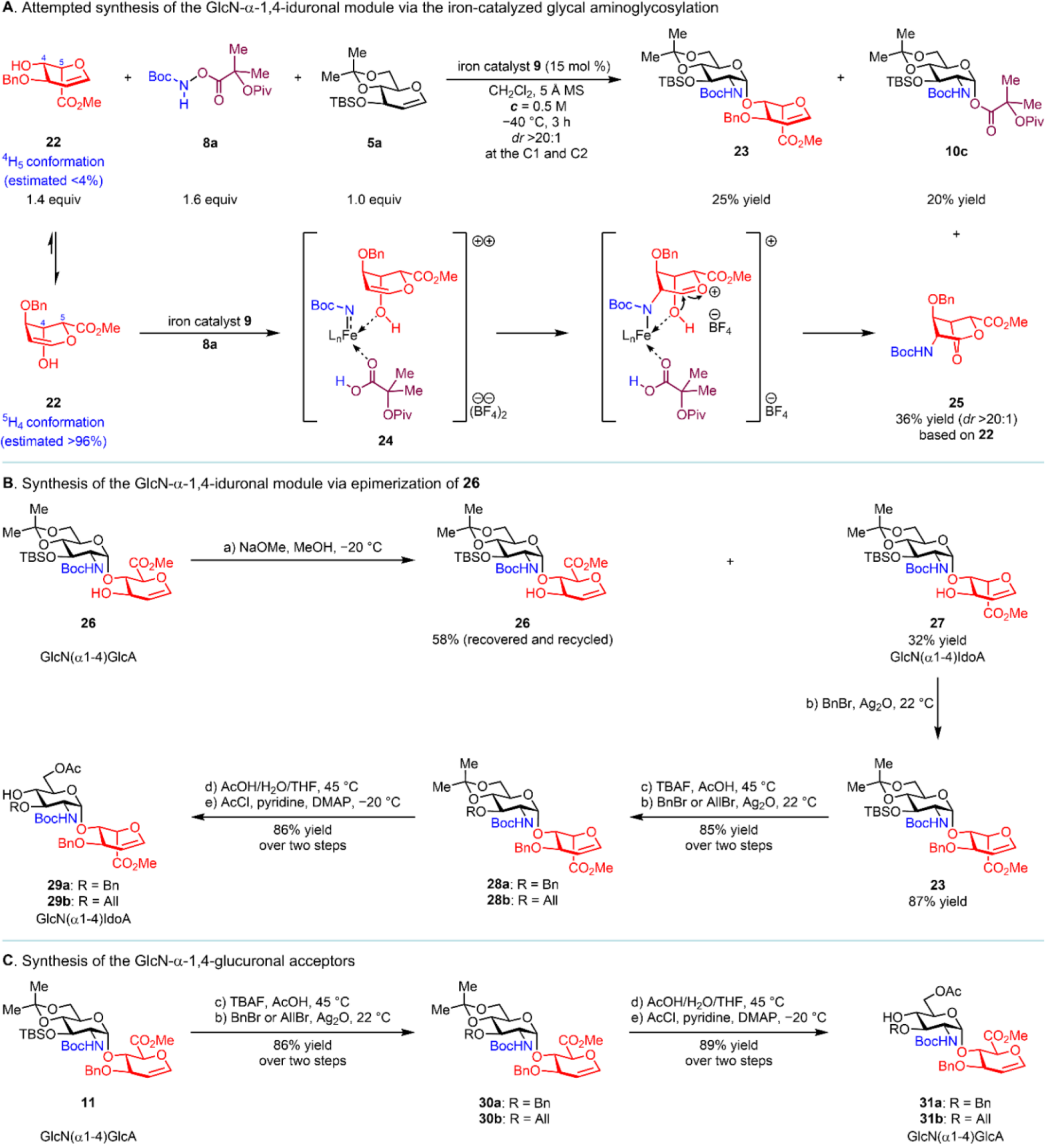
Assembly of the GlcN-α-1,4-iduronal Module and Synthesis of the GlcN-α-1,4-glucuronal/GlcN-α-1,4-iduronal Acceptors

a NaOMe (3.0 equiv), MeOH, 3 Å molecular sieves, −20 °C. ^*b*^BnBr (4.0 equiv) or AllBr (4.0 equiv), Ag_2_O (3.0 equiv), toluene, 5 Å molecular sieves, 22 °C. ^*c*^TBAF (2.0 equiv), AcOH (1.0 equiv), THF, 45 °C. ^*d*^AcOH/H_2_O/THF 2:2:1, 45 °C. ^*e*^AcCl (1.0 equiv), pyridine (1.05 equiv), DMAP (5 mol %), CH_2_Cl_2_, −20 °C. See Supporting Information for details.

We therefore explored the epimerization of GlcN-α-1,4-glucuronal module **11** (Figure S8) to generate the required GlcN-α-1,4-iduronal module **23**, even though HS fragments are known to undergo β-elimination on treatment with base.^65,66^ NaOMe-induced epimerization of **11** in anhydrous MeOH^49,67^ afforded a chromatographically inseparable mixture of **23** and **11** (Figure S8). Fortunately, multigram-scale epimerization of **26** (prepared in one-step from **10a** (96% yield, polymethylhydrosiloxane, ZnCl_2_, Pd­(PPh_3_)_4_)^68^ or **12** (96% yield, K_2_CO_3_ in MeOH)) provided the desired GlcN-α-1,4-iduronal module **27** that is readily separable from **26** by recrystallization: **27** (32% yield) along with recovered **26** (58% yield), which was re-epimerized to generate additional **27** (Scheme 1B). Benzylation of **27** afforded disaccharide **23** (87% yield), which was converted to GlcN-α-1,4-iduronal glycosyl acceptor **29a**/**29b** (Scheme 1B, 74% yield over 4 steps). GlcN-α-1,4-glucuronal glycosyl acceptor **31a**/**31b** were obtained analogously from disaccharide **11** (Scheme 1C).

With glycosyl donors **11** and **23** and acceptors **29** and **31** in hand, we were positioned to explore the proposed iterative glycosylation strategy for HS assembly (Scheme 2). The standard stereospecific glycosylation methods with glycal epoxides are only effective for primary acceptors and more reactive secondary glycal sugar acceptors, *but ineffective for the vast majority of secondary sugar acceptors*.^69–80^ With sterically hindered acceptors, these methods often induce glycal epoxide decomposition and S_N_1-type glycosylation that affords an inseparable diastereomeric mixture of glycosylation products in low yields. We have recently developed an iron (III) porphyrin-catalyzed highly stereospecific glycosylation with glycal epoxides that is particularly effective for previously problematic, hindered secondary acceptors and electron-deficient glucuronic ester epoxides (Figure 1C).^45^ Therefore, we explored the applicability of this method in the context of HS synthesis (Scheme 2).

**Scheme 2:**
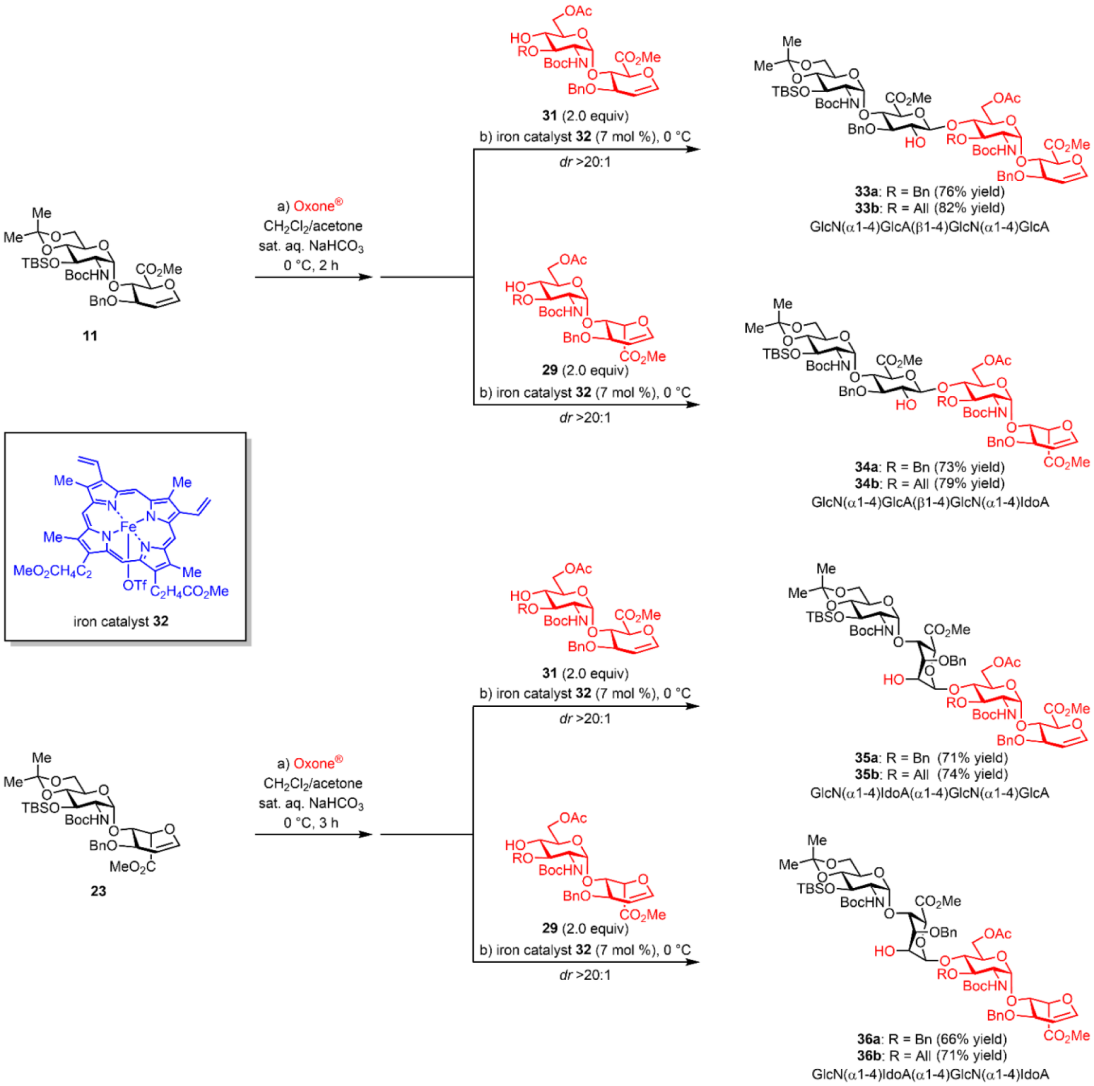
Iron-Catalyzed Stereospecific Glycosylation with Glycal Epoxides for Rapid Assembly of HS Tetrasaccharides

a Epoxidation was carried out in a biphasic reaction medium with Oxone and acetone. The glycal epoxide was dried azeotropically with toluene, assayed by ^1^H NMR, and then directly used. Oxone (3.5 equiv), CH_2_Cl_2_/acetone 5:1, saturated NaHCO_3_ solution, 0 °C. ^*b*^The glycosylation was carried out with iron­(III) porphyrin triflate catalyst **32** (7 mol %) and **29**/**31** (2.0 equiv) in the presence of 5 Å molecular sieves in CH_2_Cl_2_ at 0 °C. Iron­(III) porphyrin catalyst **32** was formed in situ from the corresponding iron porphyrin chloride (10 mol %) and AgOTf (7 mol %). All yields are isolated yields. See Supporting Information for details.

Epoxidation of GlcN-α-1,4-glucuronal **11** in a biphasic reaction medium with Oxone^81^ quantitatively afforded the corresponding glucuronic ester α-epoxide (Figure S12, *dr* > 20:1, ^3^ *J*_H1–H2_ = 2.4 Hz),^82^ which was azeotropically dried and used directly. Evaluation of an array of previously reported Brønsted acid and Lewis acid catalysts (including a stoichiometric amount of ZnCl_2_) for glycosylation of a hindered C4-OH model substrate **S10** with this glucuronic ester epoxide revealed that they mostly induce glycal epoxide decomposition and are therefore unsuitable for the desired glycosylation (Table S2).^69–80^ Fortunately, the readily available, hemin-derived iron catalyst **32** ^45^ induced the stereospecific glycosylation of GlcN-α-1,4-glucuronal **31** with this glucuronic ester epoxide, affording the desired GlcA-β-1,4-GlcN linkage within tetrasaccharide **33** in excellent yield (Scheme 2, **33a**/**b**, 76–82% yield, *dr* > 20:1, **33b**: ^3^ *J*_H1–H2_ = 7.9 Hz, ^1^ *J* ^13^_C1–H1_ = 160.7 Hz). Analogously, tetrasaccharide **34** was obtained in this iron-catalyzed glycosylation of GlcN-α-1,4-iduronal **29** (**34a**/**b**, 73–79% yield, *dr* > 20:1, **34b**: ^3^ *J*_H1–H2_ = 7.6 Hz, ^1^ *J* ^13^_C1–H1_ = 159.7 Hz). Additionally, epoxidation of GlcN-α-1,4-iduronal **23** quantatively afforded the corresponding α-epoxide (Figure S15, *dr* > 20:1, ^3^ *J*_H1–H2_ < 1.0 Hz). Once again, iron catalyst **32** is effective in glycosylating both GlcN-α-1,4-glucuronal module **31** and GlcN-α-1,4-iduronal module **29** with this iduronic ester epoxide, delivering tetrasaccharides **35** and **36** with the desired IdoA-α-1,4-GlcN linkage as single diastereomers (Scheme 2, 71–74% yield for **35a**/**b**, *dr* > 20:1, **35b**: ^3^ *J*_H1–H2_ = 1.8 Hz, ^1^ *J* ^13^_C1–H1_ = 170.8 Hz; 66–71% yield for **36a**/**b**, *dr* > 20:1, **36b**: ^3^ *J*_H1–H2_ = 2.2 Hz, ^1^ *J* ^13^_C1–H1_ = 170.1 Hz).

The successful modular synthesis of these four HS tetrasaccharides (**33**–**36**) provides the basis for development of a general HS assembly strategy. Therefore, we have targeted an anticoagulant HS hexasaccharide (Scheme 3) and several full-length precursors of HS octasaccharides (Scheme 4).

**Scheme 3:**
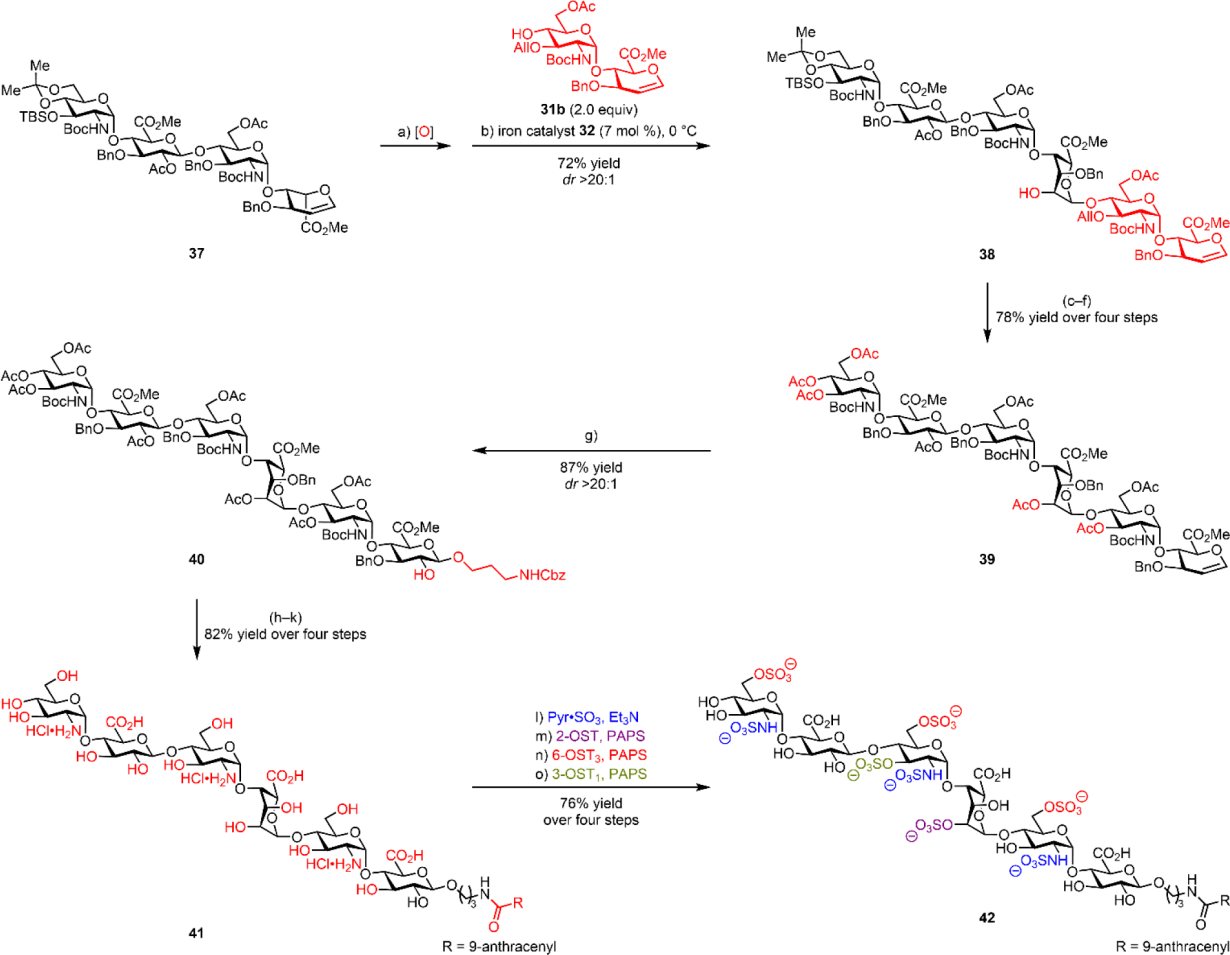
Merging Enzymatic Sulfation with the Iron-Catalyzed Reiterative Glycosylation for HS Hexasaccharide Synthesis

a Oxone (3.5 equiv), CH_2_Cl_2_/acetone 5:1, saturated NaHCO_3_ solution, 0 °C. ^*b*^The reaction was carried out with iron catalyst **32** in the presence of 5 Å molecular sieves in CH_2_Cl_2_ at 0 °C. ^*c*^TBAF (3 equiv), AcOH (1.5 equiv), THF, 35 °C. ^*d*^AcOH/H_2_O/THF 2:2:1, 45 °C; then Ac_2_O (6 equiv), pyridine (7 equiv), DMAP (10 mol %), CH_2_Cl_2_, 22 °C. ^*e*^PMHS (20 equiv), ZnCl_2_ (1 M solution in Et_2_O, 1 equiv), and Pd­(PPh_3_)_4_ (10 mol %), THF, 22 °C. ^*f*^Ac_2_O (2 equiv), pyridine (3 equiv), DMAP (10 mol %), CH_2_Cl_2_, 22 °C. ^*g*^DMDO (1.1 equiv), CH_2_Cl_2_/acetone, 0 °C; then iron catalyst **32** (7 mol %), CbzHN­(CH_2_)_3_OH (3.0 equiv), CH_2_Cl_2_, 5 Å molecular sieves, – 20 °C. ^*h*^Pd/C (20 wt %), H_2_ (1 atm), PTSA·H_2_O (1.1 equiv), THF, 22 °C. ^*i*^anthracene-9-carbonyl chloride (1.1 equiv), DIPEA (2.1 equiv), CH_2_Cl_2_, 0 °C. ^*j*^H_2_O_2_ (30% aqueous solution, 75 equiv), LiOH (3 M aqueous solution, 25 equiv), MeOH/H_2_O, 0 °C. ^*k*^HCl (4 M solution in 1,4-dioxane, 50 equiv), 22 °C. ^*l*^Pyr·SO_3_ (15 equiv), 10% Et_3_N in MeOH. ^*m*^2-OST, PAPS, MOPS buffer (pH = 7.2). ^*n*^6-OST_3_, PAPS, MOPS buffer (pH = 7.2). ^*o*^3-OST_1_, PAPS, MOPS buffer (pH = 7.2). All yields are isolated yields. See Supporting Information for details. PMHS: polymethylhydrosiloxane. 2-OST: 2-*O*-sulfotransferase. 6-OST_3_: 6-*O*-sulfotransferase isoform 3. 3-OST-1:3-*O*-sulfotransferase isoform 1. PAPS: 3′-phosphoadenosine-5′-phosphosulfate. MOPS: 3-(*N*-morpholino)­propanesulfonic acid

**Scheme 4:**
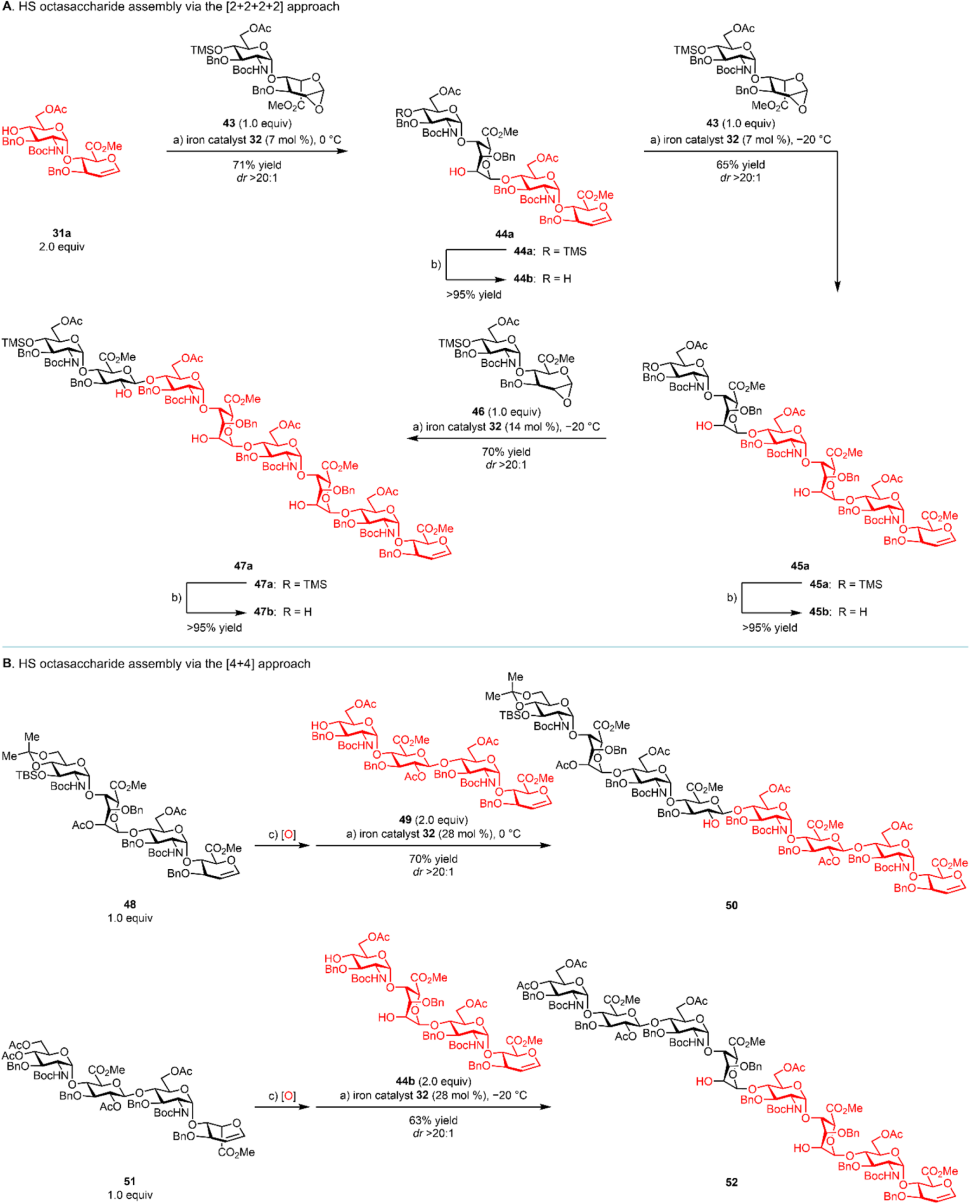
HS Octasaccharide Assembly via the Iron-Catalyzed Reiterative Glycosylation

a The reaction was carried out with iron catalyst **32** in the presence of 5 Å molecular sieves in CH_2_Cl_2_ at −20 or 0 °C. ^*b*^TBAF (1.0 equiv), AcOH (2.0 equiv), THF, 0 °C. ^*c*^DMDO (1.1 equiv), CH_2_Cl_2_/acetone, 0 °C. See Supporting Information for details.

Acetylation of **33a** (Ac_2_O with pyridine) afforded tetrasaccharide **37** (>95% yield), which was epoxidized to cleanly afford the corresponding iduronic ester α-epoxide (Figure S19, *dr* > 20:1, ^3^ *J*_H1–H2_ < 1.0 Hz). The glycal epoxide readily glycosylated a GlcN-α-1,4-glucuronal acceptor **31b** in the presence of iron catalyst **32** to stereospecifically generate hexasaccharide **38** in high yield (Scheme 3, 72%, *dr* > 20:1). This hexasaccharide could easily be activated as a glycosyl donor for further iterative glycosylation to produce longer oligosaccharides. For our current purposes, hexasaccharide **38** was converted to an unprotected, but unsulfated, HS precursor **41** in several steps (Scheme 3). Desilylation of **38** followed by acetonide opening and peracetylation allowed for the palladium-catalyzed selective 3-*O*-deallylation in high yield (95%). The following acetylation afforded hexasaccharide **39** (78% yield over 4 steps). **39** with a glucuronal moiety underwent smooth epoxidation to afford a glucuronic ester α-epoxide (*dr* > 20:1), with which the iron-catalyzed stereospecific glycosylation of an *N*-Cbz-protected amino alcohol linker provided **40** (87% yield, *dr* > 20:1). Hydrogenolysis of **40** followed by selective *N*-acylation with 9-anthracenecarbonyl chloride afforded a hexasaccharide (82% yield over 2 steps) with a long wavelength absorption (λ_max_ = 365 nm) that greatly facilitated purification of the multistep enzymatic sulfation products and does not interfere with its biological activity. Hydrolysis of this hexasaccharide under mild conditions (LiOH with H_2_O_2_)^83^ and the subsequent *N*-Boc group removal provided the unprotected, but unsulfated HS hexasaccharide **41** in high yield (>95% over 2 steps).

*N*-Sulfation of hexasaccharide **41** was achieved chemically in high yield using SO_3_·pyr (94%). Site-selective *O*-sulfation of **41** was achieved with an array of recombinant HS sulfotransferases, including 2-*O*-sulfotransferase (2-OST), 6-*O*-sulfotransferase-3 (6-OST-3) and 3-*O*-sulfotransferase-1 (3-OST-1). Each enzyme transfers a sulfate group from 3′-phosphoadenosine-5′-phosphosulfate (PAPS) to the desired location of HS. First, a 2-*O*-sulfotransferase (2-OST) selectively sulfates the C2-OH of the IdoA moiety in **41**. Next, 6-OST-3 site-selectively installed three sulfate groups at the C6-OH positions of GlcNs within **41**. Finally, 3-OST-1 precisely transferred a sulfate group to the C3-OH group of GlcN within the newly generated GlcNS6S–IdoA2S module, affording HS hexasaccharide **42** (81% yield for the 3 enzymatic steps). It is worth noting that 3-OST-1 is known to site-selectively sulfate only the GlcNS6S moiety within the trisaccharide module of GlcA-GlcNS6S-IdoA2S.^84^

With the sulfated hexasaccharide **42** in hand, we evaluated its anticoagulent activity. We measured the inhibitory effective of **42** against the activity of factor Xa, a crucial enzyme involved in blood clotting cascade, to evaluate the potential anticoagulant activity and compared the activity with fondaparinux, an FDA-approved anticoagulant drug (Figure S32). The IC_50_ value for **42** was determined to be 14 ng/mL, which is comparable to that of fondaparinux (7.3 ng/mL). Furthermore, we measured the clearance rate of **42** in mice after subcutaneous administration (Figure S33). The plasma concentration of **42** reached the maximum between 15 min and one hour and it was completely cleared after four hours.

We have demonstrated that the HS backbone can be extended from the reducing end by adding a disaccharide unit each time as a glycosyl acceptor. It is also possible to extend the HS backbone from the nonreducing end by adding a disaccharide module each time as a glycosyl donor (Scheme 4). Iron catalyst **32** promoted the stereospecific glycosylation of GlcN-α-1,4-glucuronal **31a** with an iduronic ester α-epoxide **43** affording tetrasaccharide **44a** (71% yield, *dr* > 20:1), which was selectiveily desilylated to give **44b** (>95% yield). A second iron-catalyzed stereospecific glycosylation of tetrasaccharide **44b** with iduronic ester epoxide **43** generated hexasaccharide **45a** (65% yield, *dr* > 20:1), which was again selectiveily desilylated to afford **45b** (>95% yield). Most notably, a third iron-catalyzed glycosylation of hexasaccharide **45b** with glucuronic ester α-epoxide **46** effectively provided octasaccharide **47a** as a single diastereomer (70% yield, *dr* > 20:1). Interestingly, the unprotected C2-OH groups on the iduronic ester moieties in **44b** and **45b** did not interfere with these challenging glycosylations.

Finally, the iron-catalyzed stereospecific glycosylation can join two tetrasaccharide fragments to achieve a more convergent HS assembly (Scheme 4). The iron-catalyzed stereospecific glycosylation of tetrasaccharide **49** with the glucuronic ester α-epoxide derived from tetrasaccharide **48** generated single disastereomeric octasaccharide **50** in high yield (Scheme 4, 70%, *dr* > 20:1). Epoxidation of tetrasaccharide module **51** quantitatively afforded the corresponding iduronic ester α-epoxide (Figure S30, *dr* > 20:1, ^3^ *J*_H1–H2_ < 1.0 Hz), which underwent an entirely stereospecific glycosylation with tetrasaccharide **44b** in the presence of iron catalyst **32**, affording octasaccharide **52** in good yield (Scheme 4, 63%, *dr* > 20:1).

## Conclusions

In conclusion, we have developed a highly stereoselective and generally applicable homogeneous HS oligosaccharide assembly strategy by merging the catalytic power of sulfotransferases (SULTs) with two distinct iron-catalyzed glycosylation reactions. Every glycosidic linkage in HS was assembled by one of two iron-catalyzed, entirely stereoselective glycosylation reactions. An array of sulfate groups that are essential to HS’s functions were installed at their desired locations via the SULT-controlled sulfation of HS precursors. This chemo-enzymatic catalytic approach is showcased in the synthesis of an anticoagulant HS hexasaccharide and other full-length precursors of HS octasaccharides. Our current effort focuses on the applications of this chemo-enzymatic catalytic approach in rapid assembly of other glycosaminoglycans.

## Supplementary Material



## Notes

Experimental procedure, characterization data for all new compounds and selected NMR spectra (PDF)
