# Supplementary material for: Merging Enzymatic Catalysis with Iron Catalysis for Highly Stereoselective Heparan Sulfate Oligosaccharide Assembly
Source: J Am Chem Soc. 2025 Dec 22;148(1):1766–78. doi: 10.1021/jacs.5c19067 (PMC12814361; doi:10.1021/jacs.5c19067)
Supplement: Supplementary file 1 [file ja5c19067_si_001.pdf]

Supporting Information

Merging Enzymatic Catalysis with Iron Catalysis for Highly Stereoselective Heparan Sulfate Oligosaccharide Assembly

Dakang Zhang,[‡a] Zixiang Jiang,[‡a] Le Yin,[‡a] Xiao-Wen Zhang,[a] Haoyu Yang,[a] Pinzhi Wang,[a] Changmin Xie,[a] Eduardo Stancanelli,[c] Yongmei Xu,[b] Haoran Wang,[b] Junjiang Sun,[b] Jian Liu,*[b] and Hao Xu*[a]

haohxu@brandeis.edu

liuj@email.unc.edu

[a]*Department of Chemistry, Brandeis University, 415 South Street, Waltham, Massachusetts 02453, United States*

[b]*Division of Chemical Biology and Medicinal Chemistry, Eshelman School of Pharmacy, University of North Carolina, Chapel Hill, North Carolina 27599, United States*

[c]*Glycan Therapeutics, 617 Hutton St, Raleigh, North Carolina 27606, United States*

A. General Information

B. Chemo-Enzymatic Synthesis of Glucuronal Acceptors for the Iron-Catalyzed 1,2-*cis*-Selective Glycal Aminoglycosylation

C. Assembly of the GlcN-α-1,4-Glucuronal Module via the Iron-Catalyzed 1,2-*cis*-Selective Glycal Aminoglycosylation

D. Assembly of the GlcN-α-1,4-Iduronal Module

E. The Synthesis of GlcN-α-1,4-Glucuronal Acceptors

F. Catalyst Discovery for the Iron-Catalyzed Stereospecific and Reiterative Glycosylation with Glycal Epoxides

G. Assembly of Four Heparan Sulfate Tetrasaccharide Modules via the Iron-Catalyzed Stereospecific Glycosylation with Glycal Epoxides

H. Assembly of Heparan Sulfate GlcN(α1-4)GlcA(β1-4)GlcN(α1-4)IdoA(α1-4)GlcN(α1-4)GlcA Module and Chemo-Enzymatic Sulfation for Heparan Sulfate Hexasaccharide Synthesis

I. Assembly of Full-Length Precursors of Heparan Sulfate Octasaccharides

J. Biological Assays of the Heparan Sulfate Hexasaccharide

K. References

L. NMR Spectra

A. General Information

General Procedures. All reactions were performed in oven-dried or flame-dried round-bottom flasks and vials. Stainless steel syringes and cannula were used to transfer air- and moisture-sensitive liquids. Flash chromatography was performed using silica gel 60 (230−400 mesh) from Sigma–Aldrich.

Materials. Commercial reagents were purchased from Sigma–Aldrich, TCI, Oakwood Chemicals, Combi-Blocks, Chem-Impex, Thermo Fischer Scientific and used as received. All solvents were used after being freshly distilled unless otherwise noted.

Instrumentation. Proton nuclear magnetic resonance (^{1}H NMR) spectra and carbon nuclear magnetic resonance (^{13}C NMR) spectra were recorded on Bruker Advance NEO 400 (400 MHz), Varian 400-MR (400 MHz), Bruker Advance NEO 800 (800 MHz), and Bruker Advance III (850 MHz). Chemical shifts for protons are reported in parts per million downfield from tetramethylsilane and are referenced to the NMR solvent residual peak ($CHCl_3$ δ 7.26, CD_3OD δ 3.31, acetone-d_6 δ 2.05, C_6D_6 δ 7.16, D_2O δ 4.79). Chemical shifts for carbons are reported in parts per million downfield from tetramethylsilane and are referenced to the carbon resonances of the NMR solvent ($CDCl_3$ δ 77.0, CD_3OD δ 49.0, acetone-d_6 δ 29.8, C_6D_6 δ 128.0). Data are represented as follows: chemical shift, multiplicity (br = broad, s = singlet, d = doublet, t = triplet, q = quartet, quint = quintet, m = multiplet), coupling constants in Hertz (Hz), and integration. The mass spectroscopic data were obtained using a Bruker timsTOF Pro instrument by electrospray ionization (ESI) and a Vanquish Flex UHPLC system (Thermo Fisher Scientific) coupled with Orbitrap Exploris 240 mass spectrometer by electrospray ionization (ESI). Infrared (IR) spectra were obtained using a Nicolet IR200 spectrometer with a diamond ATR. Data are represented as follows: frequency of absorption (cm^{-1}) and absorption strength (s = strong, m = medium, w = weak). Optical rotations were measured on a Jasco P-2000 Polarimeter. The cuvette dimension is 10 cm and holds 1.5 mL.

Abbreviations Used: THF–tetrahydrofuran, EtOAc–ethyl acetate, EtOH–ethanol, Et_2O–diethyl ether, CH_2Cl_2–dichloromethane, MeCN–acetonitrile, DMF–dimethylformamide, TMSOTf–

trimethylsilyl trifluoromethanesulfonate, TFA–trifluoroacetic acid, TLC–thin layer chromatography, Boc_2O–di-*tert*-butyl dicarbonate, DMAP–4-dimethylaminopyridine, DCC–*N*,*N'*-dicyclohexylcarbodiimide, DIPEA−*N*,*N*-diisopropylethylamine, TMSCl−trimethylsilyl chloride, TBSCl−*tert*-butyldimethylsilyl chloride, TMSOTf−trimethylsilyl trifluoromethanesulfonate, Ac_2O−acetic anhydride, AcCl−acetyl chloride, TBAF−tetra-*n*-butylammonium fluoride, TEMPO−2,2,6,6-tetramethyl-1-piperidinyloxy, Pd/C−palladium on carbon, AcOH−acetic acid, PMHS−polymethylhydrosiloxane, ddH_2O−double-distilled water, Pyr•SO_3−sulfur trioxide pyridine complex, PAPS−3'-phosphoadenosine-5'-phosphosulfate, 2-OST−2-*O*-sulfotransferase, MOPS−(3-(*N*-morpholino)propanesulfonic acid), 6-OST_3−6-*O*-sulfotransferase isoform 3, 3-OST-1−3-*O*-sulfotransferase isoform 1.

B. Chemo-Enzymatic Synthesis of Glucuronal Acceptors for the Iron-Catalyzed 1,2-*cis*-Selective Glycal Aminoglycosylation

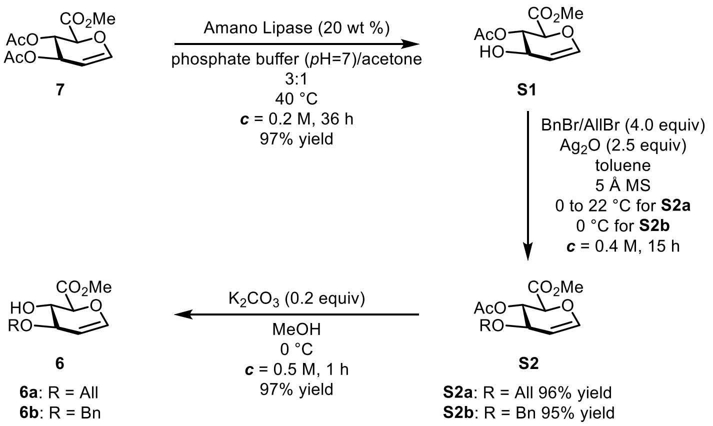

Figure S1. Chemo-Enzymatic Synthesis of Glucuronals **6a** and **6b**.

To a 1 L round bottom flask were added *Amano Lipase from Pseudomonas fluorescens* (3.0 g, 20 wt %), compound **7**[1] (15.0 g, 58 mmol, 1.0 equiv), aqueous NaH_2PO_4/Na_2HPO_4 buffer (217.5 mL, *p*H = 7.0), and acetone (72.5 mL). The mixture was stirred at 40 °C for 36 h until the starting material **7** was fully consumed (monitored by TLC). Acetone was subsequently removed *in vacuo*, followed by addition of EtOAc (300 mL) to the mixture. The organic phase was separated from the aqueous one which was further extracted with EtOAc (300 mL ×3). The combined organic phase was washed with brine (500 mL) and dried over Na_2SO_4. After concentration *in vacuo*, the residue was purified through a silica gel flash column (hexanes/EtOAc: from 100:1 to 3:2) to afford the desired product **S1** as a white solid (12.2 g, 97% yield, m.p. 78–79 °C).

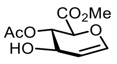

S1

Methyl 4-*O*-acetyl-D-glucuronal (**S1**): $[\alpha]_D^{22}$ +28.0 (acetone, *c* = 1.0); IR ν_{max} (neat)/cm^{-1}: 3518 (m), 2359 (m), 1731 (s), 1645 (m), 1251 (s), 1236 (s), 1212 (s); ^{1}H NMR (400 MHz, $CDCl_3$) δ 6.61 (d, *J* = 6.3 Hz, 1H), 5.32 (td, *J* = 3.0, 1.5 Hz, 1H), 5.02 (ddd, *J* = 6.3, 5.1, 1.5 Hz, 1H), 4.77

(dd, *J* = 3.0, 1.3 Hz, 1H), 3.99 (q, *J* = 4.3 Hz, 1H), 3.76 (s, 3H), 2.10 (s, 3H), 2.08 (brs, 1H); ^{13}C NMR (100 MHz, $CDCl_3$) δ 169.9, 168.3, 144.9, 100.4, 72.2, 69.9, 60.9, 52.4, 20.9; HRMS: m/z (ESI) calcd for $C_9H_{13}O_6^+$, $[M + H]^+$, 217.0707, found 217.0709.

To a 250 mL flame-dried round-bottom flask equipped with a stir bar, compound **S1** (5.0 g, 23.1 mmol, 1.0 equiv), Ag_2O (13.4 g, 57.8 mmol, 2.5 equiv), freshly activated 5 Å molecular sieves (powder, *ca*. 1.0 g), and anhydrous toluene (57.8 mL) were added. The mixture was cooled to 0 °C before benzyl bromide (11.0 mL, 92.4 mmol, 4.0 equiv) or allyl bromide (8.0 mL, 92.4 mmol, 4.0 equiv) was added dropwise. The reaction mixture was then stirred at 22 °C (for **S2a**) or at 0 °C (for **S2b**) for 15 h until the starting material **S1** was fully consumed (monitored by TLC). The reaction mixture was then filtered through a short pad of Celite® (rinsed with acetone) and concentrated *in vacuo*. The residue was purified through a silica gel flash column (hexanes/EtOAc: from 100:1 to 5:1) to afford the desired product **S2a** (5.68 g, 96% yield) as colorless oil or the desired product **S2b** (6.73 g, 95% yield) as a white solid.

To a 250 mL flame-dried round bottom flask equipped with a stir bar were added compound **S2a** (6.73 g, 22.0 mmol, 1.0 equiv) or **S2b** (5.68 g, 22.2 mmol, 1.0 equiv) and anhydrous MeOH (44 mL). The solution was cooled to 0 °C, and anhydrous K_2CO_3 powder (610 mg, 4.4 mmol, 0.2 equiv) was then added. The reaction mixture was stirred for 1 h at 0 °C, with progress monitored by TLC until completion. Amberlite® IRC 120 H (*ca*. 5 g) was then added to the mixture at 0 °C to quench the reaction. The reaction mixture was filtered through a piece of cotton (rinsed with MeOH), and the filtrate was concentrated *in vacuo*. The residue was purified through a silica gel flash column (hexanes/EtOAc: from 100:1 to 2:1) to afford the desired product **6a** (5.64 g, 97% yield) or **6b** (4.61 g, 97% yield) both as colorless oil.

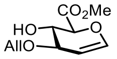

6a

Methyl 3-*O*-allyl-D-glucuronal (**6a**): $[\alpha]_D^{23}$ −17.2 (acetone, *c* = 1.0); IR ν_{max} (neat)/cm^{-1}: 3450 (w), 1741 (s), 1649 (s), 1244 (s), 1207 (s), 1102 (s), 1060 (s); ^1H NMR (400 MHz, $CDCl_3$) δ 6.59

(d, J = 6.2 Hz, 1H), 5.86 (ddt, J = 17.3, 10.4, 5.6 Hz, 1H), 5.26 (dd, J = 17.3, 1.7 Hz, 1H), 5.17 (dd, J = 10.4, 1.7 Hz, 1H), 4.96 (ddd, J = 6.2, 4.7, 1.3 Hz, 1H), 4.62 (dd, J = 4.3, 1.1 Hz, 1H), 4.33 (dtd, J = 8.1, 4.1, 1.3 Hz, 1H), 4.04 – 4.00 (m, 2H), 3.79 (ddd, J = 4.5, 3.7, 1.0 Hz, 1H), 3.74 (s, 3H), 2.25 (d, J = 9.1 Hz, 1H); ^{13}C NMR (100 MHz, $CDCl_3$) δ 168.5, 144.8, 134.4, 117.2, 98.9, 75.1, 70.2, 69.1, 66.8, 52.4; HRMS: m/z (ESI) calcd for $C_{10}H_{15}O_5^+$, $[M + H]^+$, 215.0914, found 215.0917.

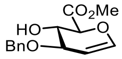

6b

Methyl 3-*O*-benzyl-D-glucuronal (**6b**): $[\alpha]_D^{22}$ −49.0 (acetone, c = 1.0); IR ν_{max} (neat)/cm^{-1}: 3446 (w), 1739 (m), 1648 (m), 1244 (m), 1205 (m), 1101 (s), 1065 (s); ^{1}H NMR (400 MHz, $CDCl_3$) δ 7.38 – 7.27 (m, 5H), 6.61 (d, J = 6.2 Hz, 1H), 4.99 (ddd, J = 6.2, 4.7, 1.3 Hz, 1H), 4.63 (dd, J = 4.2, 0.9 Hz, 1H), 4.55 (ABq, $\Delta\nu_{AB}$ = 17.2 Hz, J = 11.5 Hz, 2H), 4.43 – 4.32 (m, 1H), 3.85 (ddd, J = 4.7, 3.5, 0.9 Hz, 1H), 3.61 (s, 3H), 2.23 (d, J = 8.2 Hz, 1H); ^{13}C NMR (100 MHz, $CDCl_3$) δ 168.4, 144.9, 137.9, 128.3 (2C), 127.9 (2C), 127.7, 98.9, 75.2, 70.2, 70.1, 67.0, 52.3; HRMS: m/z (ESI) calcd for $C_{14}H_{17}O_5^+$, $[M + H]^+$, 265.1071, found 265.1077.

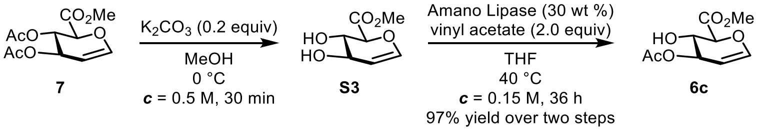

Figure S2. Chemo-Enzymatic Synthesis of Glucuronal **6c**.

To a 500 mL flame-dried round bottom flask equipped with a stir bar were added compound **7** (15.0 g, 58.08 mmol, 1.0 equiv) and anhydrous MeOH (116.1 mL). The solution was cooled to 0 °C, and anhydrous K_2CO_3 (powder, 1.605 g, 11.61 mmol, 0.2 equiv) was then added. The reaction mixture was stirred for 30 min at 0 °C, with progress monitored by TLC until

completion. Amberlite® IRC 120 H (13 g) was then added to the mixture at 0 °C to quench the reaction. The mixture was then filtered (rinsed with MeOH), and the filtrate was concentrated *in vacuo* to afford the crude product **S3** as a white solid which was used directly in the next step without further purification.

To a 1 L round bottom flask equipped with a stir bar were added *Amano Lipase from Pseudomonas fluorescens* (4.5 g, 30 wt %), crude product **S3** from the last step (58.08 mmol, 1.0 equiv), vinyl acetate (10.71 mL, 116.16 mmol, 2.0 equiv), and THF (399 mL). The mixture was stirred at 40 °C for 36 h, with progress monitored by TLC until completion. The mixture was concentrated *in vacuo*, and the residue was purified through a silica gel flash column (hexanes/EtOAc: from 100:1 to 3:2) to afford the desired product **6c** as a white solid (12.18 g, 97% yield over two steps, m.p. 77–79 °C).

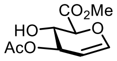

6c

Methyl 3-*O*-acetyl-D-glucuronal (**6c**): $[\alpha]_D^{22}$ −53.2 (acetone, *c* = 0.25); IR ν_{max} (neat)/cm^{-1}: 3460 (w), 1734 (s), 1650 (m), 1372 (m), 1231 (s), 1103 (m), 1020 (m); ^{1}H NMR (400 MHz, $CDCl_3$) δ 6.62 (dd, *J* = 6.2, 0.9 Hz, 1H), 5.08 (dddd, *J* = 4.7, 4.5, 1.0, 0.9 Hz, 1H), 4.94 (ddd, *J* = 6.2, 4.5, 1.2 Hz, 1H), 4.63 (dd, *J* = 4.9, 1.0 Hz, 1H), 4.31 (dddd, *J* = 6.4, 4.9, 4.7, 1.2 Hz, 1H), 3.81 (s, 3H), 2.71 (d, *J* = 6.4 Hz, 1H), 2.03 (s, 3H); ^{13}C NMR (100 MHz, $CDCl_3$) δ 170.4, 168.3, 146.0, 97.7, 75.2, 66.84, 66.76, 52.4, 21.0; HRMS: m/z (ESI) calcd for $C_9H_{13}O_6^+$, $[M + H]^+$, 217.0707, found 217.0711.

C. Assembly of the GlcN-α-1,4-Glucuronal Module via the Iron-Catalyzed 1,2-*cis*-Selective Glycal Aminoglycosylation

Table S1. Amination Reagent Discovery for the Iron-Catalyzed 1,2-*cis*-Selective Glycal Aminoglycosylation of Glucuronal Acceptor **6a**

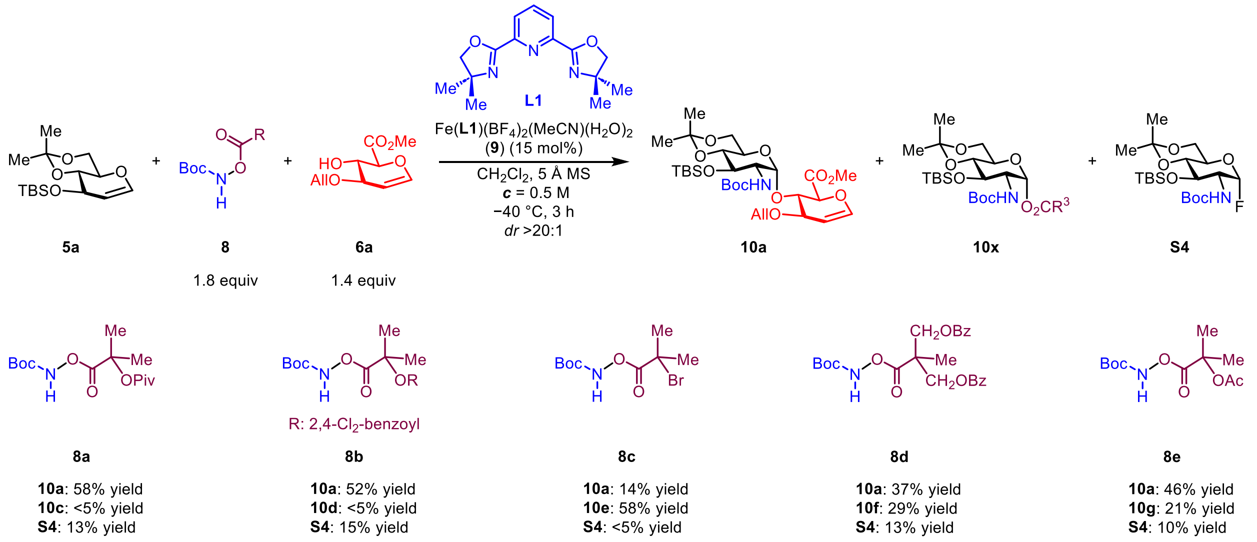

All yields are isolated yields. Reactions were performed on a 0.5 mmol scale with iron catalyst **9** (15 mol %), glycal **5a** (1.0 equiv), glycosyl acceptor **6a** (1.4 equiv), and amination reagents **8** (1.8 equiv) in CH_2Cl_2 with 5 Å molecular sieves at −40 °C for 3 h.

General Procedure for the Amination Reagent Discovery

To a flame-dried sealable 2-dram vial (vial **A**) equipped with a stir bar were added iron catalyst Fe(**L1**)$(BF_4)_2$(MeCN)$(H_2O)_2$ (**9**) (43.5 mg, 0.075 mmol, 15 mol %) and freshly activated 5 Å powdered molecular sieves (*ca.* 200 mg). After the vial was evacuated and backfilled with N_2 three times, the vial was cooled to −78 °C. To a second flame-dried sealable 2-dram vial (vial **B**) was added glycal **5a** (150 mg, 0.5 mmol, 1.0 equiv) and glycosyl acceptor **6a** (150 mg, 0.7 mmol, 1.4 equiv). Vial **B** was evacuated and backfilled with N_2 three times, and then anhydrous CH_2Cl_2 (0.5 mL) was added, then vial **B** solution was quickly transferred into vial **A** via a

syringe dropwise within 1 min. To a third flame-dried sealable 2-dram vial (vial **C**) was added amination reagent **8** (0.8 mmol, 1.6 equiv). Vial **C** was evacuated and backfilled with N_2 three times, and then anhydrous CH_2Cl_2 (0.5 mL) was added. The solution in vial **C** was added to vial **A** via a syringe dropwise within 1 min. The reaction mixture was kept at −78 °C for 5 min and transferred to −40 °C for an additional 3 h, then quenched by imidazole (20.4 mg in 1 mL CH_2Cl_2) and diluted with Et_2O (4 mL) subsequently at the same temperature. The mixture was stirred for 2 min at −40 °C and warmed up to room temperature. The solution was then filtered through a piece of cotton and washed with saturated $NaHCO_3$ solution (2 mL). The organic layer was separated from the aqueous one. The aqueous phase was further extracted with EtOAc (2 mL × 3). The combined organic layers were dried over anhydrous Na_2SO_4 and concentrated *in vacuo*. The residue was purified through a silica gel flash column to afford the desired glycal *cis*-aminoglycosylation product **10a** as white foam, the *cis*-aminoacyloxylation product **10x** as white foam, and the *cis*-aminofluorination product **S4** as a white solid.

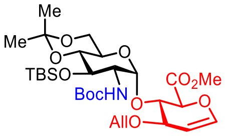

10a

Methyl 3-*O*-allyl-4-*O*-(2-*tert*-butoxycarbonylamino-3-*O*-*tert*-butyldimethylsilyl-4,6-*O*-isopropylidene-2-deoxy-α-D-glucopyranosyl)-D-glucuronal (10a): $[\alpha]_D^{25}$ +27.8 (acetone, *c* = 1.0); IR ν_{max} (neat)/cm^{-1}: 2929 (w), 1720 (m), 1503 (m), 1367 (m), 1248(s), 1171 (s), 1128 (s), 1075 (s), 1033 (s), 995 (s), 875 (s), 778 (s); ^{1}H NMR (400 MHz, $CDCl_3$) δ 6.64 (d, *J* = 6.3 Hz, 1H), 5.84 (ddt, *J* = 17.2, 10.3, 5.7 Hz, 1H), 5.24 (dd, *J* = 17.2, 1.5 Hz, 1H), 5.17 (dd, *J* = 10.3, 1.5 Hz, 1H), 5.01 (d, *J* = 4.0 Hz, 1H), 4.98 – 4.92 (m, 1H), 4.79 (dd, *J* = 3.2, 1.3 Hz, 1H), 4.55 (d, *J* = 9.9 Hz, 1H), 4.31 (td, *J* = 3.1, 1.5 Hz, 1H), 3.98 (dd, *J* = 12.4, 5.7 Hz, 1H), 3.92 (dd, *J* = 12.4, 5.7 Hz, 1H), 3.87 – 3.77 (m, 2H), 3.76 – 3.66 (m, 5H), 3.61 (td, *J* = 9.6, 4.9 Hz, 1H), 3.57 – 3.44 (m, 2H), 1.47 (s, 3H), 1.43 (s, 9H), 1.39 (s, 3H), 0.86 (s, 9H), 0.05 (s, 3H), 0.04 (s, 3H); ^{13}C NMR (100 MHz, $CDCl_3$) δ 168.0, 155.1, 145.3, 134.2, 117.5, 99.3, 99.0, 98.0, 79.5, 74.6, 73.9, 72.1, 71.0, 68.5, 67.0, 64.4, 62.2, 55.4, 52.2, 29.0, 28.4 (3C), 25.7 (3C), 18.9, 18.2, -4.2, -5.1; HRMS: m/z (ESI) calcd for $C_{30}H_{52}NO_{11}Si^+$, $[M + H]^+$, 630.3304, found 630.3326. $^1J_{^{13}C1-H1}$ = 170.6 Hz.

The C1 stereochemistry was determined by measuring ${}^{1}J^{13}{}_{C1–H1}$ through un-decoupled HSQC experiments. A ${}^{1}J^{13}{}_{\mathrm{C1–H1}}$ value of 170.6 Hz suggested that the newly formed glycosidic bond is in axial position.

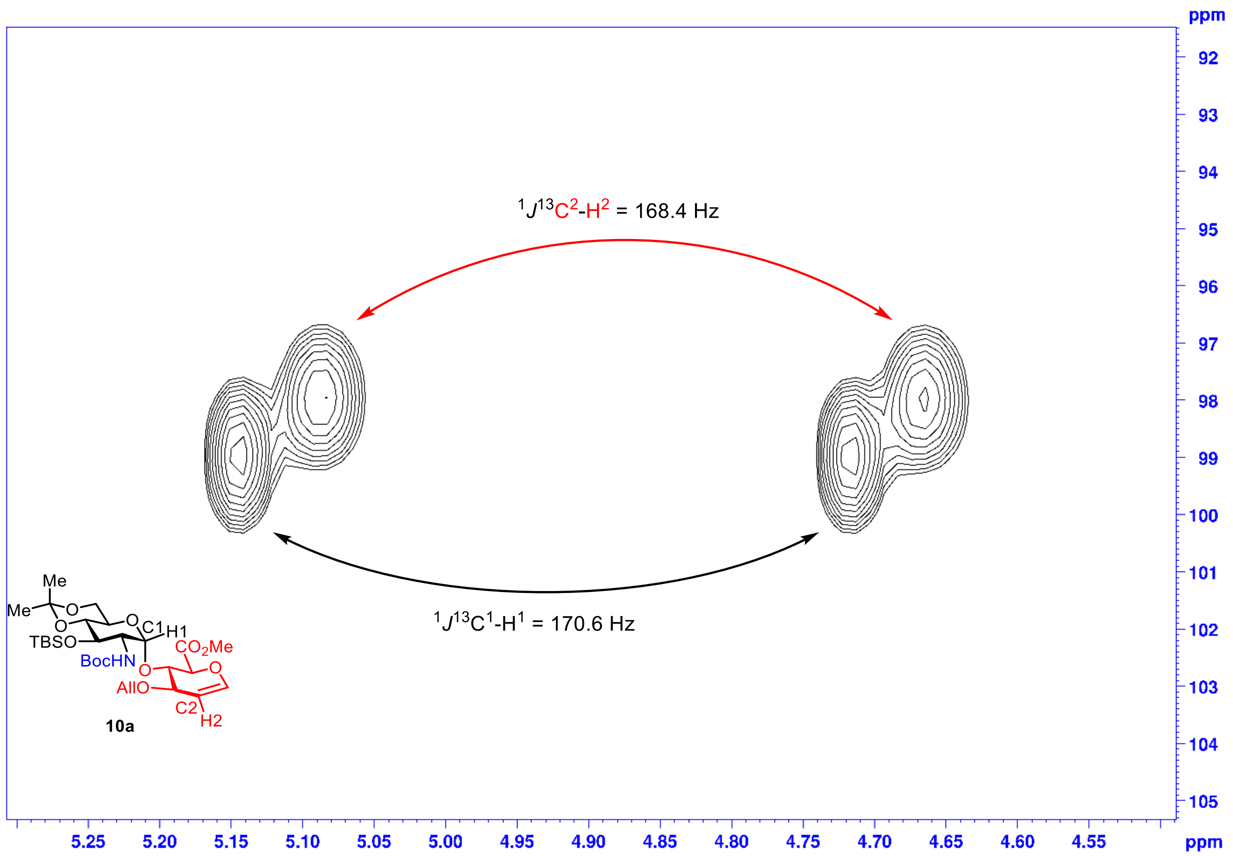

Figure S3a. Un-decoupled HSQC Analysis to Determine Stereochemistry of **10a** at the C1 Position.

The C2 stereochemistry was determined by the *NOESY* experiment of **10a** in C_6D_6: strong *NOE* was observed between H1 and H2, H2 and H4, and H3 and N-H; however, there was no *NOE* observed between H1 and H3. These data corroborated that the C–H2 bond is in axial position.

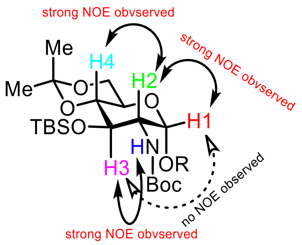

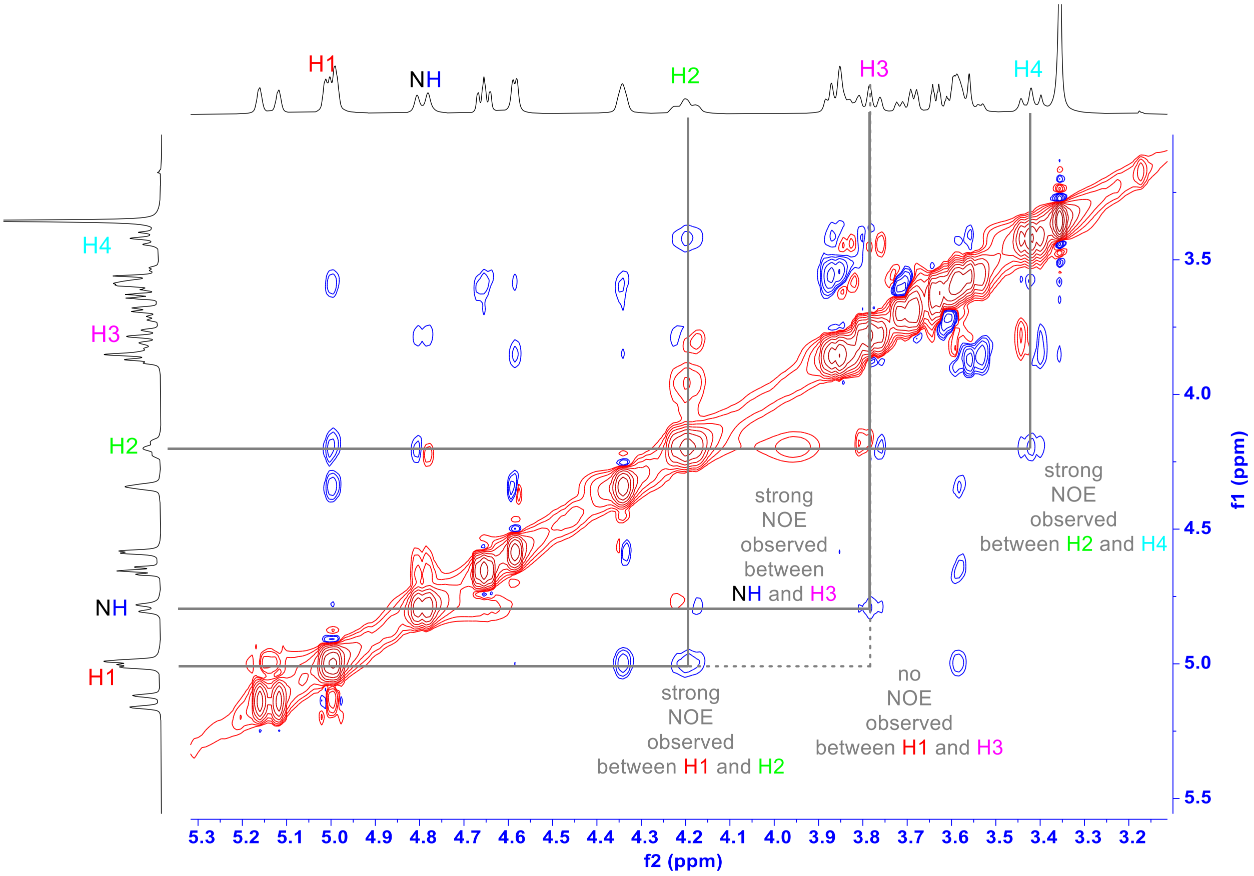

Figure S3b. The *NOESY* Experiment to Determine Stereochemistry of **10a** at the C2 Position.

The spectroscopic data of **10e**, **10f**, **10g**, and **S4** match those reported in literature.[2]

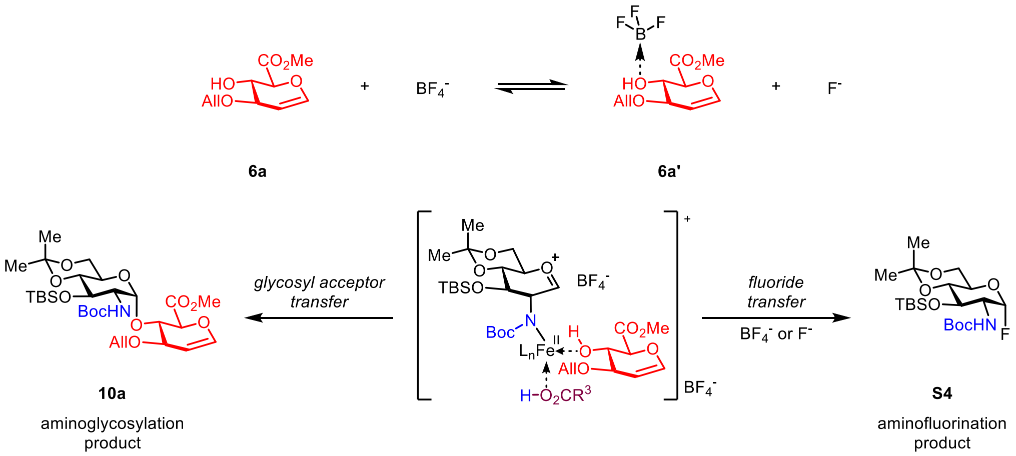

Figure S3c. A Proposed Mechanistic Pathway for 1,2-*cis*-Aminoglycosyl fluoride Formation.

The isolation of 1,2-*cis*-2-aminoglycosyl fluoride **S4** suggested the intermediacy of a glycosyl oxocarbenium ion in the glycosylation. The glycosyl oxocarbenium ion can be directly captured by the tetrafluoroborate ion and it can also be trapped by fluoride ion which was released through interaction between tetrafluoroborate ion and glycosyl acceptor **6a**.[2]

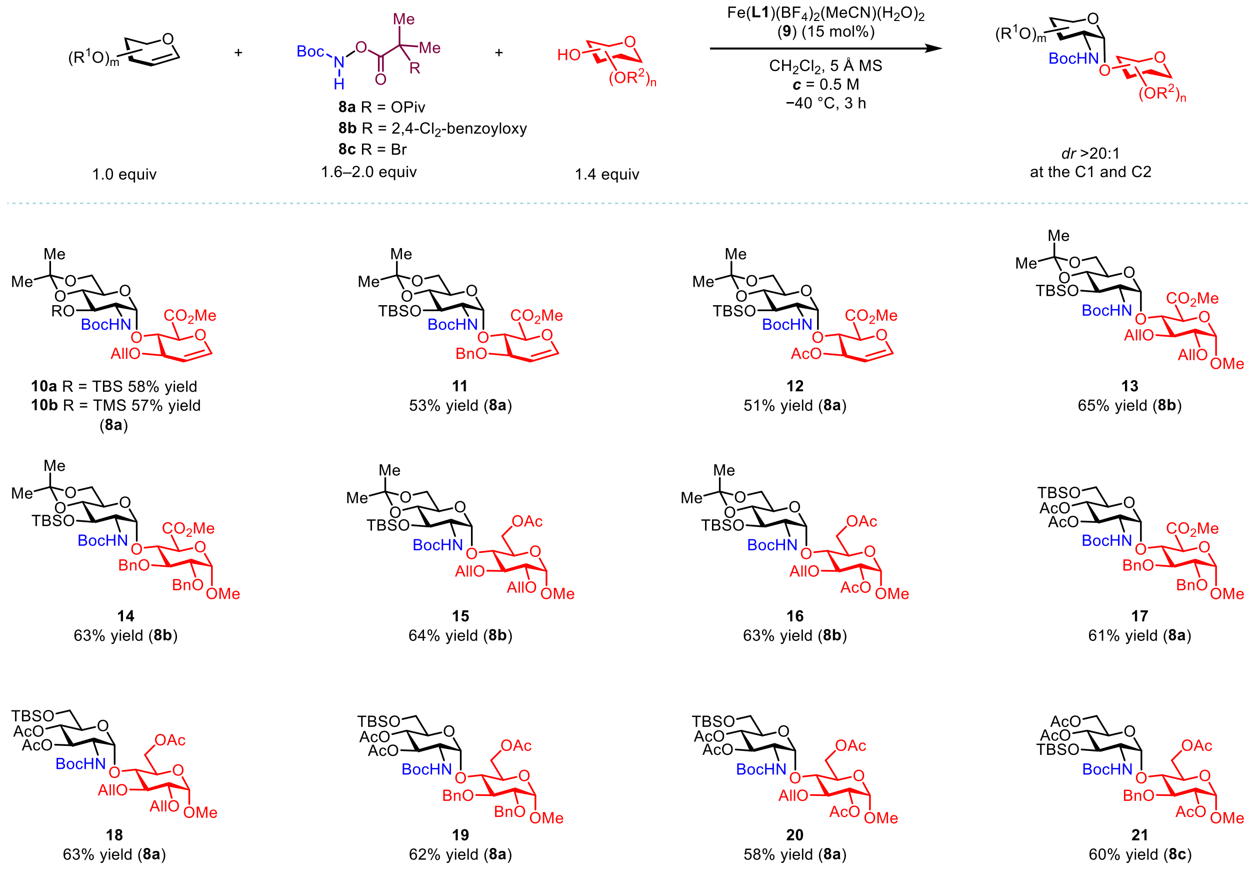

Figure S4. Substrate Scope for the Iron-Catalyzed 1,2-*cis*-Selective Glycal Aminoglycosylation of Sterically Hindered C4-OH Glycosyl Acceptors

General Procedure A: Small-Scale Iron-Catalyzed Glycal *cis*-Aminoglycosylation

To a flame-dried sealable 2-dram vial (vial **A**) equipped with a stir bar were added iron catalyst Fe(**L1**)$(BF_4)_2$(MeCN)$(H_2O)_2$ (**9**) (43.5 mg, 0.075 mmol, 15 mol %) and freshly activated 5 Å powdered molecular sieves (*ca.* 200 mg). After the vial was evacuated and backfilled with N_2 three times, the vial was cooled to −78 °C. To a second flame-dried sealable 2-dram vial (vial **B**) was added glycal (0.5 mmol, 1.0 equiv) and glycosyl acceptor (0.7 mmol, 1.4 equiv). Vial **B** was evacuated and backfilled with N_2 three times, and then anhydrous CH_2Cl_2 (0.5 mL) was added, then vial **B** solution was quickly transferred into vial **A** via a syringe dropwise within 1 min. To a third flame-dried sealable 2-dram vial (vial **C**) was added an amination reagent (0.8 mmol, 1.6 equiv). Vial **C** was evacuated and backfilled with N_2 three times, and then anhydrous CH_2Cl_2

(0.5 mL) was added. The solution in vial **C** was added to vial **A** via a syringe dropwise within 1 min. The reaction mixture was kept at −78 °C for 5 min and transferred to −40 °C for an additional 3 h, then quenched by imidazole (20.4 mg in 1 mL CH_2Cl_2) and diluted with Et_2O (4 mL) subsequently at the same temperature. The mixture was stirred for 2 min at −40 °C and warmed up to room temperature. The solution was then filtered through a piece of cotton and washed with saturated $NaHCO_3$ solution (2 mL). The organic layer was separated from the aqueous one. The aqueous phase was further extracted with EtOAc (2 mL × 3). The combined organic layers were dried over anhydrous Na_2SO_4 and concentrated *in vacuo*. The residue was purified through a silica gel flash column to afford the desired glycal *cis*-aminoglycosylation product.

General Procedure B: Large-Scale Iron-Catalyzed Glycal *cis*-Aminoglycosylation

To a flame-dried 250 mL round bottom flask (flask **A**) equipped with a stir bar were added glycal (20 mmol, 1.0 equiv), glycosyl acceptor (28 mmol, 1.4 equiv), iron catalyst Fe(**L1**)$(BF_4)_2$(MeCN)$(H_2O)_2$ (**9**) (1.74 g, 3 mmol, 15 mol %), and freshly activated 5 Å powdered molecular sieves (*ca.* 8 g). After the flask was evacuated and backfilled with N_2 twice and cooled to −78 °C, anhydrous CH_2Cl_2 (20 mL) was added. To a flame-dried 50 mL round bottom flask (flask **B**) was added an amination reagent (32 mmol, 1.6 equiv). Flask **B** was evacuated and backfilled with N_2 twice and anhydrous CH_2Cl_2 (20 mL) was added. The solution in flask **B** was then transferred to flask **A** via a syringe in 10 min. The reaction was kept at −78 °C for an additional 3 min before switched to −40 °C. The reaction was kept at −40 °C for 4 h and subsequently quenched by imidazole (820 mg in 10 mL CH_2Cl_2) at the same temperature. Et_2O (80 mL) was then added to the mixture to precipitate the iron catalyst. The mixture was stirred for two minutes and then warmed up to room temperature. The solution was then filtered through a short pad of Celite® and washed with saturated aqueous $NaHCO_3$ solution (30 mL). The organic phase was separated from the aqueous one, which was further extracted with EtOAc (30 mL × 3). The combined organic phase was dried over anhydrous Na_2SO_4 and concentrated *in vacuo*. The residue was purified through a silica gel flash column to afford the desired glycal *cis*-aminoglycosylation product.

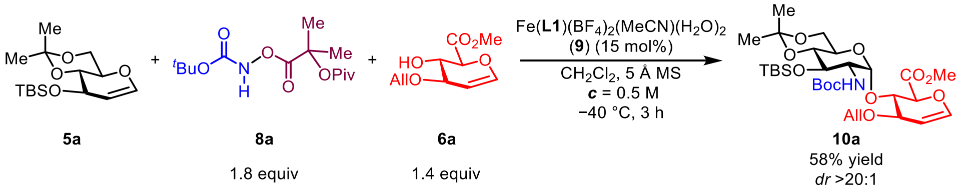

5a was synthesized according to a literature procedure.[2]

The glycal *cis*-aminoglycosylation was carried out on a 20 mmol scale by following the **General Procedure B** with the modifications: **8a** (1.8 equiv) was used. The desired product **10a** was purified through a silica gel flash column (hexanes/EtOAc: from 100:1 to 6:1) as white foam (7.31 g, 58% yield).

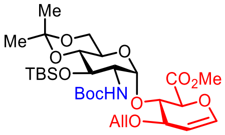

10a

Methyl 3-*O*-allyl-4-*O*-(2-*tert*-butoxycarbonylamino-3-*O*-*tert*-butyldimethylsilyl-4,6-*O*-isopropylidene-2-deoxy-α-D-glucopyranosyl)-D-glucuronal (**10a**): $[\alpha]_D^{25}$ +27.8 (acetone, *c* = 1.0); IR ν_{max} (neat)/cm^{-1}: 2929 (w), 1720 (m), 1503 (m), 1367 (m), 1248 (s), 1171 (s), 1128 (s), 1075 (s), 1033 (s), 995 (s), 875 (s), 778 (s); ^{1}H NMR (400 MHz, $CDCl_3$) δ 6.64 (d, *J* = 6.3 Hz, 1H), 5.84 (ddt, *J* = 17.2, 10.3, 5.7 Hz, 1H), 5.24 (dd, *J* = 17.2, 1.5 Hz, 1H), 5.17 (dd, *J* = 10.3, 1.5 Hz, 1H), 5.01 (d, *J* = 4.0 Hz, 1H), 4.98 – 4.92 (m, 1H), 4.79 (dd, *J* = 3.2, 1.3 Hz, 1H), 4.55 (d, *J* = 9.9 Hz, 1H), 4.31 (td, *J* = 3.1, 1.5 Hz, 1H), 3.98 (dd, *J* = 12.4, 5.7 Hz, 1H), 3.92 (dd, *J* = 12.4, 5.7 Hz, 1H), 3.87 – 3.77 (m, 2H), 3.76 – 3.66 (m, 5H), 3.61 (td, *J* = 9.6, 4.9 Hz, 1H), 3.57 – 3.44 (m, 2H), 1.47 (s, 3H), 1.43 (s, 9H), 1.39 (s, 3H), 0.86 (s, 9H), 0.05 (s, 3H), 0.04 (s, 3H); ^{13}C NMR (100 MHz, $CDCl_3$) δ 168.0, 155.1, 145.3, 134.2, 117.5, 99.3, 99.0, 98.0, 79.5, 74.6, 73.9, 72.1, 71.0, 68.5, 67.0, 64.4, 62.2, 55.4, 52.2, 29.0, 28.4 (3C), 25.7 (3C), 18.9, 18.2, -4.2, -5.1;

HRMS: m/z (ESI) calcd for $C_{30}H_{52}NO_{11}Si^+$, $[M + H]^+$, 630.3304, found 630.3326. $^1J_{^{13}C1-H1}$ = 170.6 Hz.

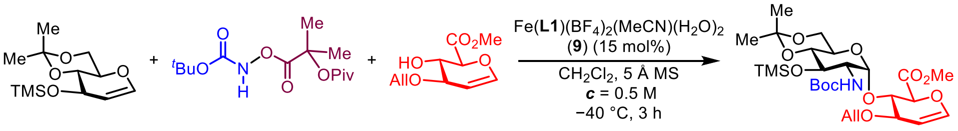

S5 **8a** **6a** **10b**

1.8 equiv 1.4 equiv 57% yield *dr* >20:1

S5 was synthesized according to a literature procedure.[2]

The glycal *cis*-aminoglycosylation was carried out on a 0.5 mmol scale by following the **General Procedure A** with the modifications: **8a** (1.8 equiv) was used. The desired product **10b** was purified through a silica gel flash column (hexanes/EtOAc: from 100:1 to 6:1) as white foam (168 mg, 57% yield).

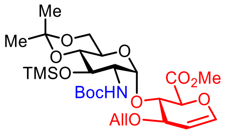

10b

Methyl 3-*O*-allyl-4-*O*-(2-*tert*-butoxycarbonylamino-4,6-*O*-isopropylidene-3-*O*-trimethylsilyl-2-deoxy-α-D-glucopyranosyl)-D-glucuronal (10b): $[\alpha]_D^{22}$ +41.8 (acetone, *c* = 1.0); IR ν_{max} (neat)/cm^{-1}: 2970 (w), 2360 (w), 2341 (w), 1722 (s), 1650 (w), 1503 (m), 1366 (s); ^{1}H NMR (400 MHz, $CDCl_3$) δ 7.25 (d, *J* = 6.3 Hz, 1H), 6.58 – 6.36 (m, 1H), 5.86 (dd, *J* = 17.2, 1.7 Hz, 1H), 5.79 (dd, *J* = 10.4, 1.7 Hz, 1H), 5.66 (d, *J* = 3.9 Hz, 1H), 5.61 – 5.53 (m, 1H), 5.40 (dd, *J* = 3.3, 1.3 Hz, 1H), 5.15 (d, *J* = 10.0 Hz, 1H), 4.93 (d, *J* = 1.6 Hz, 1H), 4.61 (ddt, *J* = 12.5, 5.6, 1.4 Hz, 1H), 4.57 – 4.50 (m, 1H), 4.50 – 4.36 (m, 3H), 4.35 – 4.29 (m, 4H), 4.26 – 4.19 (m, 1H), 4.19 – 4.04 (m, 2H), 2.08 (s, 3H), 2.06 (s, 9H), 2.00 (s, 3H), 0.71 (s, 9H); ^{13}C NMR (100 MHz, $CDCl_3$) δ 168.0, 155.3, 145.2, 134.2, 117.5, 99.3, 99.0, 98.0, 79.5, 74.3, 73.9, 72.1, 71.4, 68.4, 67.1, 64.5, 62.1, 55.3, 52.2, 28.9, 28.3 (3C), 18.9, 0.3 (3C); HRMS: m/z (ESI) calcd for $C_{27}H_{46}NO_{11}Si^+$, $[M + H]^+$, 588.2835, found 588.2838. $^1J_{^{13}C1-H1}$ = 170.5 Hz.

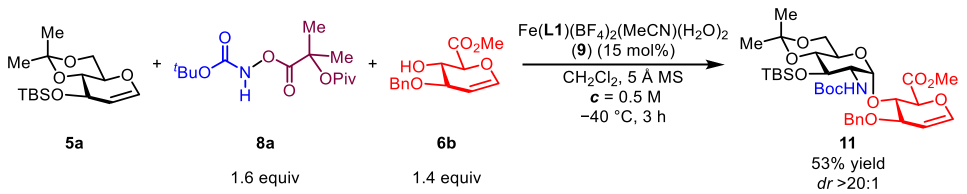

The glycal *cis*-aminoglycosylation was carried out on a 20 mmol scale by following the **General Procedure B**. The desired product **11** was purified through a silica gel flash column (hexanes/EtOAc: from 100:1 to 6:1) as white foam (7.21 g, 53% yield).

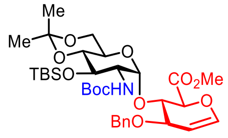

11

Methyl 3-*O*-benzyl-4-*O*-(2-*tert*-butoxycarbonylamino-3-*O*-*tert*-butyldimethylsilyl-4,6-*O*-isopropylidene-2-deoxy-α-D-glucopyranosyl)-D-glucuronal (**11**): $[\alpha]_D^{23}$ +27.9 (acetone, *c* = 1.0); IR ν_{max} (neat)/cm^{-1}: 2952 (w), 2928 (w), 2357 (w), 2359 (w), 1763 (w), 1721 (m), 1648 (w), 1501 (m), 1367 (w), 1129 (s), 1072 (s), 996 (m), 875 (m), 863 (m); ^{1}H NMR (400 MHz, $CDCl_3$) δ 7.37 – 7.25 (m, 5H), 6.67 (d, *J* = 6.3 Hz, 1H), 5.03 – 4.94 (m, 2H), 4.82 (d, *J* = 1.8 Hz, 1H), 4.57 – 4.48 (m, 2H), 4.42 (d, *J* = 11.3 Hz, 1H), 4.36 (q, *J* = 2.4 Hz, 1H), 3.90 – 3.76 (m, 3H), 3.71 (t, *J* = 10.3 Hz, 1H), 3.62 (td, *J* = 9.4, 4.9 Hz, 1H), 3.56 (s, 3H), 3.58 – 3.46 (m, 2H), 1.47 (s, 3H), 1.42 (s, 9H), 1.39 (s, 3H), 0.86 (s, 9H), 0.05 (s, 3H), 0.04 (s, 3H); ^{13}C NMR (100 MHz, $CDCl_3$) δ 168.2, 155.3, 145.6, 137.7, 128.5 (2C), 128.1 (2C), 128.0, 99.5, 99.2, 98.1, 79.7, 74.8, 74.0, 72.4, 71.2, 69.7, 67.1, 64.6, 62.4, 55.6, 52.3, 29.2, 28.6 (3C), 25.9 (3C), 19.1, 18.4, -4.0, -4.9; HRMS: m/z (ESI) calcd for $C_{34}H_{54}NO_{11}Si^+$, $[M + H]^+$, 680.3461, found 680.3458. $^1J_{^{13}C1-H1}$ = 168.3 Hz.

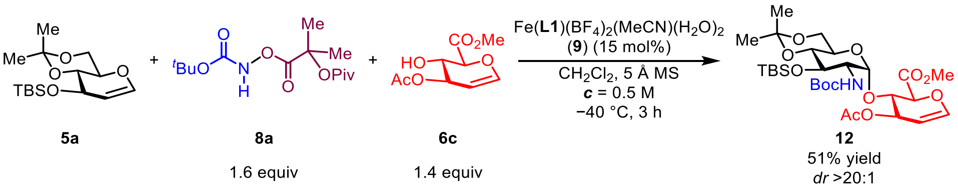

The glycal *cis*-aminoglycosylation was carried out on a 20 mmol scale by following the **General Procedure B**. The desired product **12** was purified through a silica gel flash column (hexanes/EtOAc: from 100:1 to 4:1) as white foam (6.44 g, 51% yield).

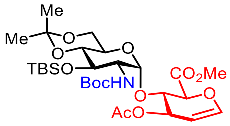

12

Methyl 3-*O*-acetyl-4-*O*-(2-*tert*-butoxycarbonylamino-3-*O*-*tert*-butyldimethylsilyl-4,6-*O*-isopropylidene-2-deoxy-α-D-glucopyranosyl)-D-glucuronal (**12**): $[\alpha]_D^{23}$ +36.9 (acetone, *c* = 1.0); IR ν_{max} (neat)/cm^{-1}: 2954 (w), 2929 (w), 2857 (w), 1722 (s), 1650 (w), 1503 (m), 1368 (s), 1282 (s), 1172 (s), 1076 (s), 1035 (s), 875 (m), 837 (m), 779 (m); ^{1}H NMR (400 MHz, $CDCl_3$) δ 6.72 – 6.65 (d, *J* = 5.5 Hz, 1H), 5.07 (d, *J* = 3.9 Hz, 1H), 5.00 – 4.93 (m, 2H), 4.83 (d, *J* = 3.1 Hz, 1H), 4.51 (d, *J* = 9.9 Hz, 1H), 4.32 (d, *J* = 2.4 Hz, 1H), 3.87 – 3.80 (m, 2H), 3.77 (s, 3H), 3.70 (t, *J* = 10.3 Hz, 1H), 3.64 – 3.46 (m, 3H), 1.97 (s, 3H), 1.46 (s, 3H), 1.43 (s, 9H), 1.38 (s, 3H), 0.85 (s, 9H), 0.05 (s, 3H), 0.04 (s, 3H); ^{13}C NMR (100 MHz, $CDCl_3$) δ 169.7, 167.9, 155.3, 146.7, 99.6, 99.2, 97.1, 79.9, 74.8, 74.1, 72.0, 71.2, 64.8, 63.3, 62.4, 55.5, 52.5, 29.2, 28.5 (3C), 25.9 (3C), 21.1, 19.1, 18.4, -4.0, -4.9; HRMS: m/z (ESI) calcd for $C_{29}H_{50}NO_{12}Si^+$, $[M + H]^+$, 632.3097, found 632.3089. $^1J_{^{13}C1–H1}$ = 172.0 Hz.

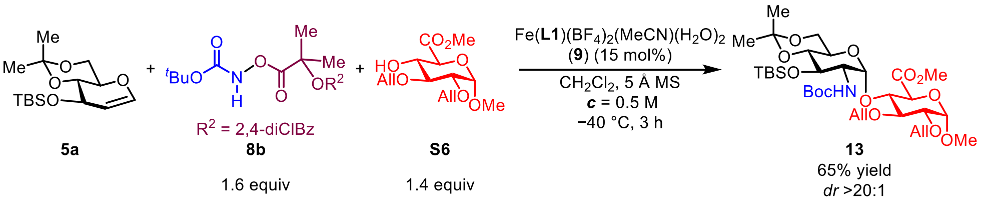

S6 was synthesized according to a modified literature procedure.[3]

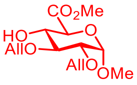

S6

Methyl (methyl 2,3-di-*O*-allyl-α-D-glucopyranosid)uronate (**S6**): $[\alpha]_D^{23}$ +100.5 (acetone, *c* = 1.0); IR ν_{max} (neat)/cm^{-1}: 3487 (w), 1749 (m), 1197 (m), 1149 (m), 1048 (s), 993 (m), 923 (m); ^{1}H NMR (400 MHz, $CDCl_3$) δ 6.08 – 5.74 (m, 2H), 5.40 – 5.24 (m, 2H), 5.22 – 5.12 (m, 2H), 4.86 (d, *J* = 3.5 Hz, 1H), 4.35 (ddt, *J* = 12.6, 5.6, 1.5 Hz, 1H), 4.27 (ddt, *J* = 12.6, 5.9, 1.4 Hz, 1H), 4.21 (ddt, *J* = 12.8, 5.5, 1.4 Hz, 1H), 4.18 – 4.08 (m, 2H), 3.82 (s, 3H), 3.77 (td, *J* = 8.9, 2.9 Hz, 1H), 3.68 (t, *J* = 8.8 Hz, 1H), 3.47 (s, 3H), 3.44 (dd, *J* = 9.2, 3.4 Hz, 1H), 2.97 (d, *J* = 3.0 Hz, 1H); ^{13}C NMR (100 MHz, $CDCl_3$) δ 170.7, 135.0, 134.5, 117.9, 117.1, 98.7, 79.8, 78.3, 74.1, 72.7, 71.7, 70.6, 55.8, 52.7; HRMS: m/z (ESI) calcd for $C_{14}H_{23}O_7^+$, $[M + H]^+$, 303.1438, found 303.1422.

The glycal *cis*-aminoglycosylation was carried out on a 0.5 mmol scale by following the **General Procedure A**. The desired product **13** was purified through a silica gel flash column (hexanes/EtOAc: from 100:1 to 5:1) as white foam (233 mg, 65% yield).

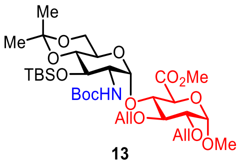

13

Methyl (2-*tert*-butoxycarbonylamino-3-*O*-*tert*-butyldimethylsilyl-4,6-*O*-isopropylidene-2-deoxy-α-D-glucopyranosyl)-(1→4)-(methyl 2,3-di-*O*-allyl-α-D-glucopyranosid)uronate (13):

$[\alpha]_D^{23}$ +63.5 (acetone, c = 0.5); IR ν_{max} (neat)/cm^{-1}: 2936 (m), 2360 (s), 2341 (s), 1754 (m), 1723 (s), 1504 (m), 1368 (s); ^{1}H NMR (400 MHz, $CDCl_3$) δ 5.93 – 5.87 (m, 2H), 5.29 (dd, J = 7.8, 1.6 Hz, 1H), 5.26 – 5.22 (m, 2H), 5.22 – 5.18 (m, 1H), 5.14 (dd, J = 10.3, 1.8 Hz, 1H), 4.83 (d, J = 10.3 Hz, 1H), 4.79 (d, J = 3.5 Hz, 1H), 4.36 (dd, J = 11.8, 6.1 Hz, 1H), 4.23 – 4.03 (m, 4H), 3.93 (t, J = 9.4 Hz, 1H), 3.84 – 3.73 (m, 6H), 3.64 (t, J = 10.5 Hz, 1H), 3.56 – 3.40 (m, 6H), 3.27 (td, J = 10.0, 5.2 Hz, 1H), 1.44 (s, 3H), 1.41 (s, 9H), 1.36 (s, 3H), 0.86 (s, 9H), 0.05 (s, 3H), 0.04 (s, 3H); ^{13}C NMR (100 MHz, $CDCl_3$) δ 169.7, 155.0, 134.4, 118.2, 117.7, 99.4, 99.2, 98.6, 80.2, 79.4, 79.1, 75.0, 74.5, 74.3, 72.6, 71.3, 70.4, 64.3, 61.9, 55.8, 55.3, 52.7, 29.1, 28.5 (3C), 28.3 (3C), 25.8, 18.9, 18.3, -4.1, -5.1; HRMS: m/z (ESI) calcd for $C_{34}H_{60}NO_{13}Si^+$, [M + H]$^+$, 718.3828, found 718.3822. $^1J^{13}{}_{C1-H1}$ = 177.0 Hz, 172.9 Hz.

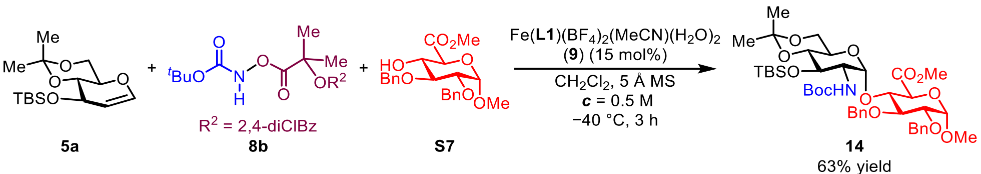

S7 was synthesized according to a literature procedure.[3]

The glycal *cis*-aminoglycosylation was carried out on a 0.5 mmol scale by following the **General Procedure A**. The desired product **14** was purified through a silica gel flash column (hexanes/EtOAc: from 100:1 to 5:1) as white foam (258 mg, 63% yield).

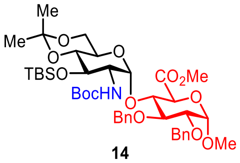

14

Methyl (2-*tert*-butoxycarbonylamino-3-*O*-*tert*-butyldimethylsilyl-4,6-*O*-isopropylidene-2-deoxy-α-D-glucopyranosyl)-(1→4)-(methyl 2,3-di-*O*-benzyl-α-D-glucopyranosid)uronate

(**14**): $[\alpha]_D^{23}$ + 42.3 (acetone, *c* = 0.5); IR ν_{max} (neat)/cm^{-1}: 2952 (w), 2360 (s), 2342 (s), 1754 (m), 1721 (s), 1499 (m), 1369 (m); ^{1}H NMR (400 MHz, $CDCl_3$) δ 7.40 – 7.27 (m, 10H), 5.45 (d, *J* = 3.9 Hz, 1H), 4.82 (d, *J* = 10.3 Hz, 1H), 4.78 (ABq, $\Delta\nu_{AB}$ = 95.1 Hz, *J* = 9.9 Hz, 2H), 4.66 (ABq, $\Delta\nu_{AB}$ = 74.2 Hz, *J* = 12.0 Hz, 2H), 4.54 (d, *J* = 3.6 Hz, 1H), 4.18 (d, *J* = 9.3 Hz, 1H), 4.00 (t, *J* = 9.0 Hz, 1H), 3.95 (t, *J* = 8.8 Hz, 1H), 3.87 (td, *J* = 10.0, 4.1 Hz, 1H), 3.82 (s, 3H), 3.77 (dd, *J* = 10.6, 5.3 Hz, 1H), 3.63 (t, *J* = 10.6 Hz, 1H), 3.56 (dd, *J* = 9.0, 3.5 Hz, 1H), 3.51 (t, *J* = 9.0 Hz, 1H), 3.46 (t, *J* = 9.0 Hz, 1H), 3.41 (s, 3H), 3.18 (td, *J* = 10.0, 5.2 Hz, 1H), 1.43 (s, 3H), 1.35 (s, 3H), 1.31 (s, 9H), 0.86 (s, 9H), 0.06 (s, 3H), 0.05 (s, 3H); ^{13}C NMR (100 MHz, $CDCl_3$) δ 169.8, 155.0, 137.7, 137.6, 128.6 (2C), 128.5 (2C), 128.4 (2C), 128.3 (2C), 128.2, 127.9, 99.2, 98.7, 98.5, 81.2, 79.6, 79.6, 76.7, 75.6, 74.4, 73.6, 71.4, 70.1, 64.0, 61.8, 55.9, 55.2, 52.8, 29.1, 28.4 (3C), 25.8 (3C), 18.9, 18.3, -4.1, -5.1; HRMS: m/z (ESI) calcd for $C_{42}H_{64}NO_{13}Si^+$, $[M + H]^+$, 818.4141, found 818.4157. $^1J_{^{13}C1-H1}$ = 173.6 Hz, 169.8 Hz.

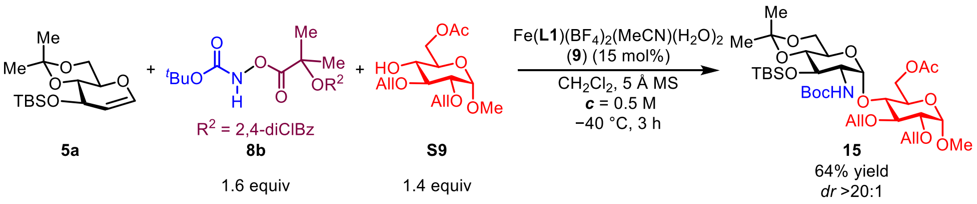

S9 was synthesized according to the following procedure.

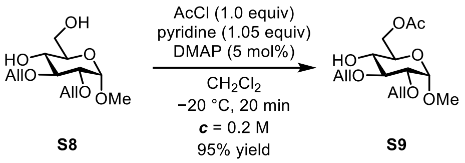

To a 250 mL flame-dried round bottom flask equipped with a stir bar, compound **S8**[4] (5.0 g, 18.23 mmol, 1.0 equiv), DMAP (111 mg, 0.91 mmol, 5 mol %), and anhydrous CH_2Cl_2 (73 mL) were added. The mixture was cooled to −20 °C before anhydrous pyridine (1.54 mL, 19.14 mmol, 1.05 equiv) and AcCl (1M in CH_2Cl_2, 18.23 mL, 18.23 mmol, 1.0 equiv) were added dropwise sequentially. The reaction mixture was stirred at −20 °C for 20 min until the starting

material **S8** was fully consumed (monitored by TLC). The reaction mixture was then quenched with H_2O (80 mL) and the organic phase was separated from the aqueous one. The aqueous phase was further extracted with CH_2Cl_2 (100 mL × 2). The combined organic phase was washed with brine (80 mL) and dried over Na_2SO_4. After concentration *in vacuo*, the residue was purified through a silica gel flash column (hexanes/EtOAc: from 100:1 to 3:2) to afford the desired product **S9** (5.48 g, 95% yield) as colorless oil.

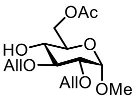

S9

Methyl 6-*O*-acetyl-2,3-di-*O*-allyl-α-D-glucopyranoside (**S9**): $[\alpha]_D^{23}$ +100.0 (acetone, *c* = 1.0); IR ν_{max} (neat)/cm^{-1}: 3466 (w), 2913 (w), 1739 (s), 1369 (m), 1240 (s), 1194 (w), 1046 (s); ^{1}H NMR (400 MHz, $CDCl_3$) δ 6.11 – 5.71 (m, 2H), 5.30 – 5.17 (m, 2H), 5.18 – 5.04 (m, 2H), 4.72 (d, *J* = 3.6 Hz, 1H), 4.47 – 4.28 (m, 2H), 4.25 – 4.13 (m, 2H), 4.13 – 4.01 (m, 2H), 3.69 (ddd, *J* = 10.0, 4.8, 2.3 Hz, 1H), 3.57 (t, *J* = 9.2 Hz, 1H), 3.46 – 3.27 (m, 5H), 2.58 (brs, 1H), 2.04 (s, 3H); ^{13}C NMR (100 MHz, $CDCl_3$) δ 171.4, 135.0, 134.6, 117.8, 117.2, 98.3, 80.7, 79.3, 74.2, 72.3, 69.9, 69.3, 63.3, 55.2, 20.8; HRMS: m/z (ESI) calcd for $C_{15}H_{25}O_7^+$, $[M + H]^+$, 317.1595, found 317.1582.

The glycal *cis*-aminoglycosylation was carried out on a 0.5 mmol scale by following the **General Procedure A**. The desired product **15** was purified through a silica gel flash column (hexanes/Et_2O: from 100:1 to 3:2) as white foam (234 mg, 64% yield).

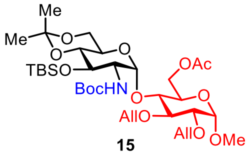

15

Methyl 2-*tert*-butoxycarbonylamino-3-*O*-*tert*-butyldimethylsilyl-4,6-*O*-isopropylidene-2-deoxy-α-D-glucopyranosyl-(1→4)-6-*O*-acetyl-2,3-di-*O*-allyl-α-D-glucopyranoside (**15**): $[\alpha]_D^{23}$ +73.4 (acetone, *c* = 1.0); IR ν_{max} (neat)/cm^{-1}: 2929 (m), 2856 (m), 2332 (w), 1745 (s), 1721 (s), 1505 (m), 1368 (s); ^{1}H NMR (400 MHz, $CDCl_3$) δ 6.09 – 5.80 (m, 2H), 5.32 – 5.28 (m, 2H), 5.26 (dd, *J* = 3.7, 1.8 Hz, 1H), 5.20 (dd, *J* = 10.2, 1.5 Hz, 1H), 5.15 (dd, *J* = 10.4, 1.7 Hz, 1H), 5.06 (d,

J = 10.1 Hz, 1H), 4.76 (d, *J* = 3.6 Hz, 1H), 4.51 – 4.36 (m, 2H), 4.23 – 4.07 (m, 4H), 3.89 – 3.73 (m, 4H), 3.73 – 3.64 (m, 2H), 3.59 – 3.47 (m, 3H), 3.45 – 3.40 (m, 4H), 2.10 (s, 3H), 1.45 (s, 3H), 1.42 (s, 9H), 1.38 (s, 3H), 0.86 (s, 9H), 0.05 (s, 3H), 0.04 (s, 3H); ^{13}C NMR (100 MHz, $CDCl_3$) δ 170.5, 155.2, 134.5, 134.4, 118.1, 117.6, 99.9, 99.4, 97.8, 80.7, 79.9, 79.4, 74.7, 74.5, 73.9, 72.4, 71.4, 68.1, 64.9, 63.0, 62.3, 55.6, 55.4, 29.0, 28.5 (3C), 25.8 (3C), 20.8, 19.0, 18.3, -4.1, -5.0; HRMS: m/z (ESI) calcd for $C_{35}H_{62}NO_{13}Si^+$, [M + H]$^+$, 732.3985, found 732.3970. $^1J_{^{13}C1-H1}$ = 172.1 Hz, 169.0 Hz.

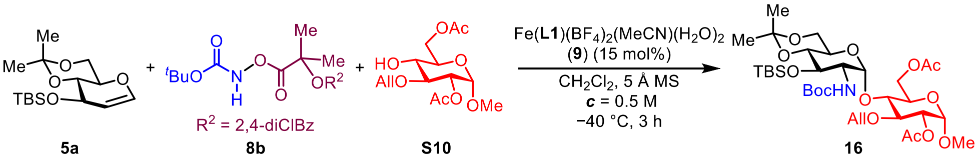

5a	8b	S10		16
	R^2 = 2,4-diClBz			
	1.6 equiv	1.4 equiv		63% yield
				dr >20:1

S10 was synthesized according to a literature procedure.[5]

The glycal *cis*-aminoglycosylation was carried out on a 0.5 mmol scale by following the **General Procedure A**. The desired product **16** was purified through a silica gel flash column (hexanes/EtOAc: from 100:1 to 4:1) as white foam (231 mg, 63% yield).

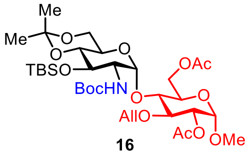

16

Methyl 2-*tert*-butoxycarbonylamino-3-*O*-*tert*-butyldimethylsilyl-4,6-*O*-isopropylidene-2-deoxy-α-D-glucopyranosyl-(1→4)-2,6-di-*O*-acetyl-3-*O*-allyl-α-D-glucopyranoside (16): $[\alpha]_D^{23}$ +82.8 (acetone, *c* = 1.0); IR ν_{max} (neat)/cm^{-1}: 2930 (m), 2361 (w), 2332 (w), 1746 (s), 1721 (s), 1506 (w), 1369 (m); ^{1}H NMR (400 MHz, $CDCl_3$) δ 5.87 (ddt, *J* = 17.3, 10.7, 5.6 Hz, 1H), 5.29 – 5.19 (m, 2H), 5.14 (dd, *J* = 10.5, 1.4 Hz, 1H), 5.06 (d, *J* = 10.1 Hz, 1H), 4.86 (d, *J* = 3.7 Hz, 1H), 4.78 (dd, *J* = 9.9, 3.6 Hz, 1H), 4.49 (dd, *J* = 12.0, 1.6 Hz, 1H), 4.24 – 4.11 (m, 3H), 3.94 – 3.80

(m, 2H), 3.81 – 3.75 (m, 3H), 3.68 (t, J = 10.2 Hz, 1H), 3.60 – 3.44 (m, 3H), 3.38 (s, 3H), 2.12 (s, 3H), 2.10 (s, 3H), 1.45 (s, 3H), 1.42 (s, 9H), 1.38 (s, 3H), 0.86 (s, 9H), 0.05 (s, 3H), 0.04 (s, 3H); ^{13}C NMR (100 MHz, $CDCl_3$) δ 170.5, 170.0, 155.2, 134.1, 117.4, 100.2, 99.4, 96.7, 79.5, 79.2, 75.0, 74.7, 73.8, 73.6, 71.3, 68.2, 65.0, 62.8, 62.2, 55.7, 55.3, 29.0, 28.5 (3C), 25.8 (3C), 21.1, 20.8, 18.9, 18.3, -4.1, -5.1; HRMS: m/z (ESI) calcd for $C_{34}H_{60}NO_{14}Si^+$, $[M + H]^+$, 734.3778, found 734.3782. $^1J_{^{13}C1–H1}$ = 173.0 Hz, 174.5 Hz.

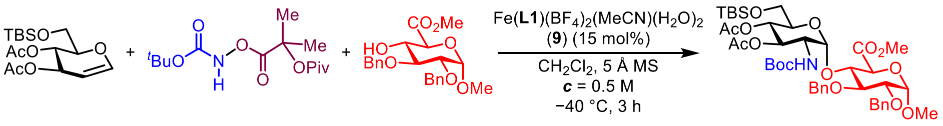

S11 | **8a** 2.0 equiv | **S7** 1.4 equiv | **17** 61% yield *dr* >20:1

S11 was synthesized according to a literature procedure.[6]

The glycal *cis*-aminoglycosylation was carried out on a 0.5 mmol scale by following the **General Procedure A** with the modifications: **8a** (2.0 equiv) was used. The desired product **17** was purified through a silica gel flash column (hexanes/acetone: from 100:1 to 6:1) as white foam (263 mg, 61% yield).

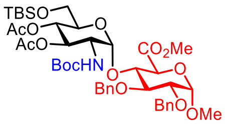

17

Methyl (3,4-di-*O*-acetyl-2-*tert*-butoxycarbonylamino-6-*O*-*tert*-butyldimethylsilyl-2-deoxy-α-D-glucopyranosyl)-(1→4)-(methyl 2,3-di-*O*-benzyl-α-D-glucopyranosid)uronate (17): $[\alpha]_D^{23}$ +74.1 (acetone, c = 0.5); IR ν_{max} (neat)/cm^{-1}: 2930 (m), 2857 (w), 1752 (s), 1719 (m), 1499 (m), 1242 (s), 1042 (s); ^{1}H NMR (400 MHz, $CDCl_3$) δ 7.40 – 7.26 (m, 10H), 5.44 (d, J = 3.7 Hz, 1H), 5.15 (t, J = 9.5 Hz, 1H), 5.10 (t, J = 9.8 Hz, 1H), 4.93 (d, J = 10.1 Hz, 1H), 4.82 (ABq, $\Delta\nu_{AB}$ =

63.5 Hz, J = 10.5 Hz, 2H), 4.62 (ABq, $\Delta\nu_{AB}$ = 56.7 Hz, J = 12.0 Hz, 2H), 4.56 (d, J = 3.4 Hz, 1H), 4.18 (d, J = 9.5 Hz, 1H), 4.01 (t, J = 9.1 Hz, 1H), 3.99 – 3.90 (m, 2H), 3.78 (s, 3H), 3.67 (dd, J = 11.3, 2.7 Hz, 1H), 3.64 – 3.55 (m, 2H), 3.54 – 3.47 (m, 1H), 3.41 (s, 3H), 2.00 (s, 3H), 1.98 (s, 3H), 1.31 (s, 9H), 0.87 (s, 9H), 0.01 (s, 6H); ^{13}C NMR (100 MHz, $CDCl_3$) δ 171.1, 169.7, 168.8, 155.0, 137.8, 137.6, 128.6 (2C), 128.5 (2C), 128.25 (2C), 128.16, 128.0 (2C), 127.8, 98.4, 97.8, 80.7, 79.8, 79.6, 75.3, 74.5, 73.5, 72.0, 70.5, 70.1, 68.5, 61.6, 55.8, 52.9, 52.6, 28.2 (3C), 25.8 (3C), 20.8, 20.7, 18.3, -5.5, -5.6; HRMS: m/z (ESI) calcd for $C_{43}H_{64}NO_{15}Si^+$, [M + H]$^+$, 862.4040, found 862.4066. $^1J_{^{13}C1-H1}$ = 177.1 Hz, 168.7 Hz.

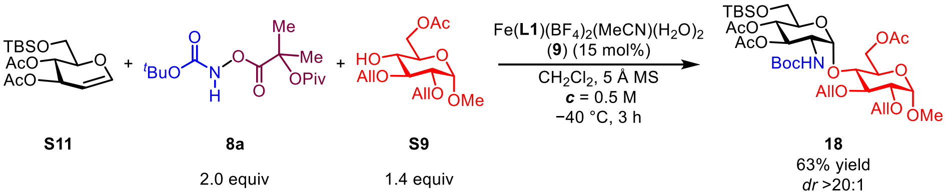

The glycal *cis*-aminoglycosylation was carried out on a 0.5 mmol scale by following the **General Procedure A** with the modifications: **8a** (2.0 equiv) was used. The desired product **18** was purified through a silica gel flash column (hexanes/acetone: from 100:1 to 5:1) as white foam (244 mg, 63% yield).

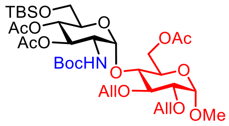

18

Methyl 3,4-di-*O*-acetyl-2-*tert*-butoxycarbonylamino-6-*O*-*tert*-butyldimethylsilyl-2-deoxy-α-D-glucopyranosyl-(1→4)-6-*O*-acetyl-2,3-di-*O*-allyl-α-D-glucopyranoside (18): $[\alpha]_D^{23}$ +90.3 (acetone, c = 1.0); IR ν_{max} (neat)/cm^{-1}: 2930 (w), 2359 (w), 2342 (w), 1747 (s), 1718 (m), 1506 (w), 1236 (s); ^{1}H NMR (400 MHz, $CDCl_3$) δ 6.07 – 5.80 (m, 2H), 5.34 – 5.20 (m, 5H), 5.22 – 5.05 (m, 4H), 4.76 (d, J = 3.5 Hz, 1H), 4.48 – 4.37 (m, 2H), 4.33 – 4.19 (m, 2H), 4.18 – 4.10 (m,

2H), 3.96 (td, J = 10.0, 3.5 Hz, 1H), 3.85 – 3.74 (m, 1H), 3.77 – 3.71 (m, 1H), 3.74 – 3.64 (m, 1H), 3.66 – 3.59 (m, 2H), 3.47 – 3.42 (m, 1H), 3.42 (s, 3H), 2.10 (s, 3H), 2.001 (s, 3H), 1.997 (s, 3H), 1.40 (s, 9H), 0.88 (s, 9H), 0.02 (s, 6H); ^{13}C NMR (100 MHz, $CDCl_3$) δ 170.9, 170.5, 169.1, 155.2, 134.4 (two peaks overlapped, 2C), 118.1, 117.7, 99.2, 97.7, 80.2, 80.0, 79.6, 76.1, 74.2, 72.3, 72.1, 71.5, 68.4, 68.2, 63.3, 61.9, 55.3, 53.3, 28.3 (3C), 25.8 (3C), 20.9, 20.7 (two peaks overlapped, 2C), 18.3, -5.5 (two peaks overlapped, 2C); HRMS: m/z (ESI) calcd for $C_{36}H_{62}NO_{15}Si^+$, $[M + H]^+$, 776.3883, found 776.3873. $^1J^{13}{}_{C1-H1}$ = 174.5 Hz, 169.6 Hz.

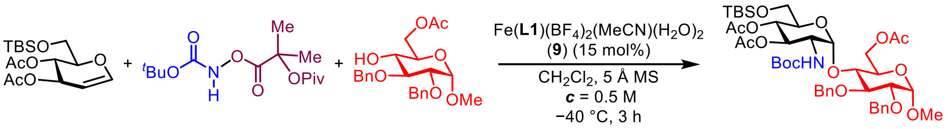

S13 was synthesized according to the following procedure.

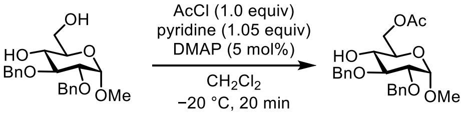

To a 250 mL flame-dried round bottom flask equipped with a stir bar, compound **S12**[7] (5.0 g, 13.35 mmol, 1.0 equiv), DMAP (82 mg, 0.67 mmol, 5 mol %), and anhydrous CH_2Cl_2 (53.5 mL) were added. The mixture was cooled to −20 °C before anhydrous pyridine (1.13 mL, 14.02 mmol, 1.05 equiv) and AcCl (1M in CH_2Cl_2, 13.35 mL, 13.35 mmol, 1.0 equiv) were added dropwise sequentially. The reaction mixture was stirred at −20 °C for 20 min until the starting material **S12** was fully consumed (monitored by TLC). The reaction mixture was then quenched with H_2O (60 mL) and the organic phase was separated from the aqueous one. The aqueous phase was further extracted with CH_2Cl_2 (80 mL × 2). The combined organic phase was washed with brine (60 mL) and dried over Na_2SO_4. After concentration *in vacuo*, the residue was

purified through a silica gel flash column (hexanes/EtOAc: from 100:1 to 3:2) to afford the desired product **S13** (5.34 g, 96% yield) as colorless oil.

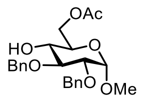

S13

Methyl 6-*O*-acetyl-2,3-di-*O*-benzyl-α-D-glucopyranoside (**S13**): $[\alpha]_D^{23}$ +77.7 (acetone, *c* = 1.0); IR ν_{max} (neat)/cm^{-1}: 3467 (w), 2911 (w), 1739 (s), 1367 (w), 1241 (s), 1052 (s), 1028 (s); ^{1}H NMR (400 MHz, $CDCl_3$) δ 7.45 – 7.26 (m, 10H), 4.87 (ABq, $\Delta\nu_{AB}$ = 100.2 Hz, *J* = 11.3 Hz, 2H), 4.72 (ABq, $\Delta\nu_{AB}$ = 45.8 Hz, *J* = 12.1 Hz, 2H), 4.62 (d, *J* = 3.6 Hz, 1H), 4.41 (dd, *J* = 12.1, 4.7 Hz, 1H), 4.21 (dd, *J* = 12.1, 2.2 Hz, 1H), 3.79 (t, *J* = 9.2 Hz, 1H), 3.74 (ddd, *J* = 10.0, 4.8, 2.2 Hz, 1H), 3.51 (dd, *J* = 9.6, 3.6 Hz, 1H), 3.42 (dd, *J* = 10.0, 8.9 Hz, 1H), 3.38 (s, 3H), 2.46 (brs, 1H), 2.08 (s, 3H); ^{13}C NMR (100 MHz, $CDCl_3$) δ 171.3, 138.6, 137.9, 128.6 (2C), 128.5 (2C), 128.1 (2C), 128.04 (2C), 128.00, 127.9, 98.2, 81.1, 79.5, 75.6, 73.2, 69.9, 69.2, 63.2, 55.3, 20.8; HRMS: m/z (ESI) calcd for $C_{23}H_{29}O_7^+$, $[M + H]^+$, 417.1908, found 417.1903.

The glycal *cis*-aminoglycosylation was carried out on a 0.5 mmol scale by following the **General Procedure A** with the modifications: **8a** (2.0 equiv) was used. The desired product **19** was purified through a silica gel flash column (hexanes/acetone: from 100:1 to 6:1) as white foam (272 mg, 62% yield).

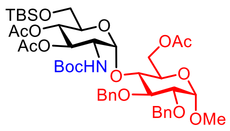

19

Methyl 3,4-di-*O*-acetyl-2-*tert*-butoxycarbonylamino-6-*O*-*tert*-butyldimethylsilyl-2-deoxy-α-D-glucopyranosyl-(1→4)-6-*O*-acetyl-2,3-di-*O*-benzyl-α-D-glucopyranoside (**19**): $[\alpha]_D^{23}$ +74.1 (acetone, *c* = 0.5); IR ν_{max} (neat)/cm^{-1}: 2930 (w), 2858 (w), 1747 (s), 1718 (m), 1366 (m), 1238 (s), 1038 (s); ^{1}H NMR (400 MHz, $CDCl_3$) δ 7.38 – 7.23 (m, 10H), 5.52 (d, *J* = 3.9 Hz, 1H), 5.20 – 5.08 (m, 2H), 5.05 (d, *J* = 10.2 Hz, 1H), 4.87 (ABq, $\Delta\nu_{AB}$ = 64.0 Hz, *J* = 10.6 Hz, 2H), 4.60 (ABq, $\Delta\nu_{AB}$ = 51.9 Hz, *J* = 12.0 Hz, 2H), 4.55 (d, *J* = 3.5 Hz, 1H), 4.37 (dd, *J* = 12.1, 1.8 Hz, 1H), 4.28 – 4.17 (m, 1H), 4.07 – 3.90 (m, 2H), 3.86 – 3.72 (m, 3H), 3.69 – 3.58 (m, 2H), 3.53

(dd, J = 9.5, 3.5 Hz, 1H), 3.38 (s, 3H), 2.08 (s, 3H), 2.003 (s, 3H), 2.000 (s, 3H), 1.29 (s, 9H), 0.87 (s, 9H), 0.012 (s, 3H), 0.009 (s, 3H); ^{13}C NMR (100 MHz, $CDCl_3$) δ 171.0, 170.5, 169.0, 155.0, 138.0, 137.7, 128.5 (2C), 128.4 (2C), 128.2 (2C), 128.1, 127.9 (2C), 127.7, 98.4, 97.5, 81.2, 80.6, 79.8, 75.0, 74.6, 73.3, 72.2, 71.3, 68.3, 67.9, 63.4, 61.8, 55.3, 52.9, 28.1 (3C), 25.9 (3C), 20.8, 20.7 (two peaks overlapped, 2C), 18.3, -5.5 (2C); HRMS: m/z (ESI) calcd for $C_{44}H_{66}NO_{15}Si^+$, [M + H]$^+$, 876.4196, found 876.4210. $^1J_{^{13}C1-H1}$ = 176.7 Hz, 169.1 Hz.

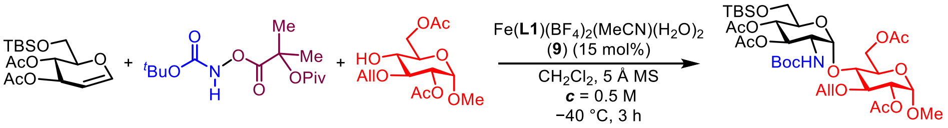

The glycal *cis*-aminoglycosylation was carried out on a 0.5 mmol scale by following the **General Procedure A** with the modifications: **8a** (2.0 equiv) was used. The desired product **20** was purified through a silica gel flash column (hexanes/acetone: from 100:1 to 6:1) as white foam (226 mg, 58% yield).

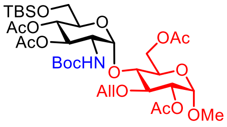

20

Methyl 3,4-di-*O*-acetyl-2-*tert*-butoxycarbonylamino-6-*O*-*tert*-butyldimethylsilyl-2-deoxy-α-D-glucopyranosyl-(1→4)-2,6-di-*O*-acetyl-3-*O*-allyl-α-D-glucopyranoside (**20**): $[\alpha]_D^{23}$ +102.9 (acetone, c = 1.0); IR ν_{max} (neat)/cm^{-1}: 2931 (w), 2857 (w), 1745 (s), 1719 (m), 1367 (m), 1233 (s), 1038 (s); ^{1}H NMR (400 MHz, $CDCl_3$) δ 6.02 – 5.78 (m, 1H), 5.30 (d, J = 10.1 Hz, 1H), 5.29 – 5.18 (m, 2H), 5.19 – 5.03 (m, 3H), 4.85 (dd, J = 3.7, 1.1 Hz, 1H), 4.78 (dd, J = 9.9, 3.6 Hz, 1H), 4.48 (d, J = 12.2 Hz, 1H), 4.32 – 4.17 (m, 3H), 4.01 – 3.86 (m, 2H), 3.87 – 3.72 (m, 3H), 3.66 – 3.61 (m, 2H), 3.37 (s, 3H), 2.12 (s, 3H), 2.10 (s, 3H), 2.00 (s, 3H), 2.00 (s, 3H), 1.40 (s, 9H),

0.87 (s, 9H), 0.02 (s, 6H); ^{13}C NMR (100 MHz, $CDCl_3$) δ 171.0, 170.4, 170.0, 169.0, 155.2, 134.1, 117.5, 99.4, 96.7, 79.7, 78.8 (two peaks overlapped, 2C), 76.3, 73.91, 73.88, 72.0, 71.7, 68.32, 68.27, 62.9, 61.9, 55.2, 53.4, 28.2 (3C), 25.8 (3C), 21.1, 20.8, 20.7, 20.7, 18.3, -5.5; HRMS: m/z (ESI) calcd for $C_{35}H_{60}NO_{16}Si^+$, $[M + H]^+$, 778.3676, found 778.3651. $^1J_{^{13}C1-H1}$ = 174.8 Hz, 171.4 Hz.

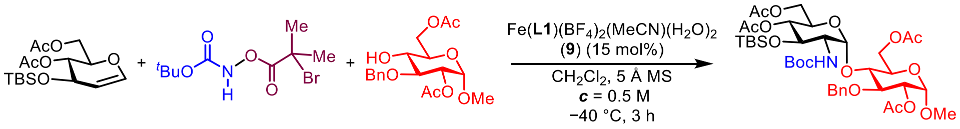

S14	8c	S15	21
	1.8 equiv	1.4 equiv	60% yield, *dr* >20:1

S14 was synthesized according to a literature procedure.[8]

S15 was synthesized according to a modified literature procedure.[5]

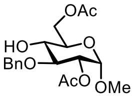

S15

Methyl 2,6-di-*O*-acetyl-3-*O*-benzyl-α-D-glucopyranoside (**S15**): $[\alpha]_D^{23}$ +119.6 (acetone, *c* = 1.0); IR ν_{max} (neat)/cm^{-1}: 3483 (w), 2939 (w), 1741 (s), 1370 (m), 1236 (s), 1127 (m), 1040 (s); ^{1}H NMR (400 MHz, $CDCl_3$) δ 7.44 – 7.26 (m, 5H), 4.90 (d, *J* = 3.6 Hz, 1H), 4.83 (dd, *J* = 10.0, 3.7 Hz, 1H), 4.77 (ABq, $\Delta\nu_{AB}$ = 25.5 Hz, *J* = 11.6 Hz, 2H), 4.46 (dd, *J* = 12.2, 4.4 Hz, 1H), 4.25 (dd, *J* = 12.2, 2.3 Hz, 1H), 3.84 (dd, *J* = 9.9, 8.8 Hz, 1H), 3.77 (ddd, *J* = 10.0, 4.4, 2.2 Hz, 1H), 3.52 (dd, *J* = 10.0, 8.8 Hz, 1H), 3.39 (s, 3H), 2.63 (brs, 1H), 2.11 (s, 3H), 2.08 (s, 3H); ^{13}C NMR (100 MHz, $CDCl_3$) δ 171.5, 170.3, 138.3, 128.6 (2C), 127.9, 127.7 (2C), 97.2, 79.4, 75.3, 73.2, 70.0, 69.4, 62.9, 55.2, 20.9, 20.8; HRMS: m/z (ESI) calcd for $C_{18}H_{25}O_8^+$, $[M + H]^+$, 369.1544, found 369.1552.

The glycal *cis*-aminoglycosylation was carried out on a 0.5 mmol scale by following the **General Procedure A** with the modifications: **8c** (1.8 equiv) was used. The desired product **21** was purified through a silica gel flash column (hexanes/EtOAc: from 100:1 to 2:1) as white foam (248 mg, 60% yield).

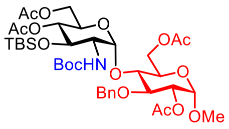

21

Methyl 4,6-di-*O*-acetyl-2-*tert*-butoxycarbonylamino-3-*O*-*tert*-butyldimethylsilyl-2-deoxy-α-D-glucopyranosyl-(1→4)-2,6-di-*O*-acetyl-3-*O*-benzyl-α-D-glucopyranoside (**21**): $[\alpha]_D^{23}$ +68.9 (acetone, *c* = 2.0); IR ν_{max} (neat)/cm^{-1}: 2931 (w), 2857 (w), 1744 (s), 1506 (w), 1368 (m), 1234 (s), 1036 (s); ^{1}H NMR (400 MHz, $CDCl_3$) δ 7.42 – 7.14 (m, 5H), 5.47 (d, *J* = 3.7 Hz, 1H), 5.02 – 4.91 (m, 2H), 4.87 (d, *J* = 3.7 Hz, 1H), 4.85 – 4.77 (m, 2H), 4.68 (d, *J* = 11.6 Hz, 1H), 4.50 (d, *J* = 11.9 Hz, 1H), 4.23 – 4.12 (m, 2H), 4.04 (t, *J* = 8.7 Hz, 1H), 3.99 – 3.91 (m, 2H), 3.90 – 3.78 (m, 3H), 3.74 (t, *J* = 9.4 Hz, 1H), 3.40 (s, 3H), 2.11 (s, 3H), 2.08 (s, 3H), 2.06 (s, 3H), 1.82 (s, 3H), 1.33 (s, 9H), 0.83 (s, 9H), 0.06 (s, 3H), 0.02 (s, 3H); ^{13}C NMR (100 MHz, $CDCl_3$) δ 170.8, 170.5, 170.1, 169.3, 154.9, 138.0, 128.5 (2C), 127.6, 126.9 (2C), 99.0, 96.6, 80.7, 79.9, 74.7, 74.4, 73.7, 71.2, 70.7, 69.4, 68.0, 62.9, 62.3, 55.4, 55.0, 28.4 (3C), 25.5 (3C), 21.2, 20.8, 20.7, 20.5, 17.7, -4.3, -4.5; HRMS: m/z (ESI) calcd for $C_{39}H_{62}NO_{16}Si^+$, $[M + H]^+$, 828.3822, found 828.3814. $^1J_{^{13}C1\text{–}H1}$ = 174.0 Hz, 171.4 Hz.

D. Assembly of the GlcN-α-1,4-Iduronal Module

a. Attempted Direct Generation of the GlcN-α-1,4-Iduronal Module via the Iron-Catalyzed Glycal *cis*-Aminoglycosylation

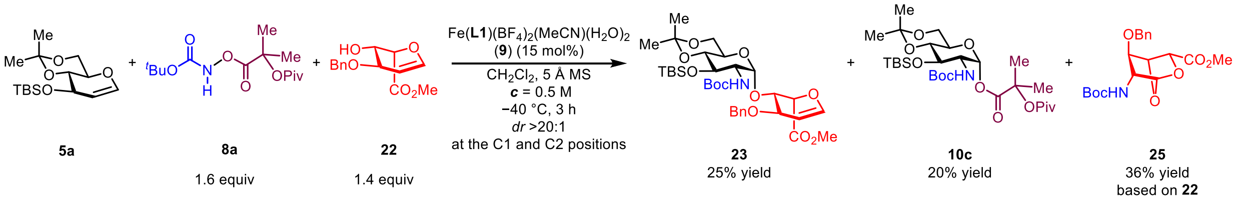

Figure S5. Iron-Catalyzed 1,2-*cis*-Aminoglycosylation of Iduronal **22** with **5a**.

22 was synthesized according to the following procedure.

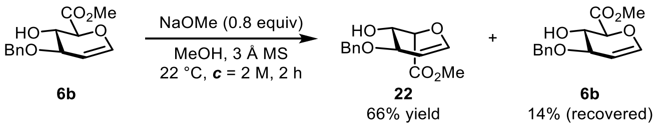

To a 100 mL flame-dried round-bottom flask equipped with a stir bar, glucuronal **6b** (4.0 g, 15.1 mmol, 1.0 equiv) and freshly activated 3 Å molecular sieves powder (*ca*. 200 mg) were added. After the flask was evacuated and backfilled with N_2 three times, anhydrous MeOH (3.6 mL) was added, and the solution was cooled to 0 °C. Freshly prepared NaOMe solution (3.0 M solution in anhydrous MeOH, 4.0 mL, 12.1 mmol, 0.8 equiv) was added dropwise into the reaction mixture over 5 min at 0 °C. The reaction mixture was stirred at 22 °C for an additional 2 h and subsequently diluted with anhydrous MeOH (30 mL). Amberlite® IRC 120 H (8.5 g) was then added to the reaction mixture at 0 °C to quench the reaction and the resulting mixture was stirred for 5 min until *p*H reached 7. The mixture was subsequently filtered, and the filtrate was concentrated *in vacuo*. The residue was purified through a silica gel flash column (hexanes/EtOAc: from 100:1 to 2.5:1) to afford iduronal **22** (2.64 g, 66% yield) and recovered **6b** (560 mg, 14% yield) both as colorless oil.

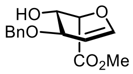

22

Methyl 3-*O*-benzyl-L-guluronal (**22**): $[\alpha]_D^{23}$ −176.8 (acetone, *c* = 1.0); IR ν_{max} (neat)/cm^{-1}: 3467 (w), 1743 (s), 1644 (s), 1240 (s), 1098 (s), 1064 (s), 1028 (m); ^{1}H NMR (400 MHz, $CDCl_3$) δ 7.43 – 7.27 (m, 5H), 6.65 (d, *J* = 6.1 Hz, 1H), 5.03 (ddd, *J* = 6.1, 5.2, 1.6 Hz, 1H), 4.68 (ABq, $\Delta\nu_{AB}$ = 12.7 Hz, *J* = 11.8 Hz, 2H), 4.57 (d, *J* = 1.5 Hz, 1H), 4.28 (dddd, *J* = 7.6, 2.3, 1.6, 1.5 Hz, 1H), 3.85 (s, 3H), 3.74 (dd, *J* = 5.2, 2.3 Hz, 1H), 2.02 (d, *J* = 7.6 Hz, 1H); ^{13}C NMR (100 MHz, $CDCl_3$) δ 169.1, 145.8, 137.9, 128.5 (2C), 127.9, 127.7 (2C), 99.2, 73.1, 70.2, 69.6, 67.3, 52.6; HRMS: m/z (ESI) calcd for $C_{14}H_{17}O_5^+$, $[M + H]^+$, 265.1071, found 265.1069.

To a flame-dried sealable 2-dram vial (vial **A**) equipped with a stir bar were added iron catalyst Fe(**L1**)$(BF_4)_2$(MeCN)$(H_2O)_2$ (**9**) (43.5 mg, 0.075 mmol, 15 mol %) and freshly activated 5 Å powdered molecular sieves (*ca.* 200 mg). After the vial was evacuated and backfilled with N_2 three times, the vial was cooled to −78 °C. To a second flame-dried sealable 2-dram vial (vial **B**) was added glycal **5a** (150 mg, 0.5 mmol, 1.0 equiv) and glycosyl acceptor **22** (185 mg, 0.7 mmol, 1.4 equiv). Vial **B** was evacuated and backfilled with N_2 three times, and then anhydrous CH_2Cl_2 (0.5 mL) was added, then vial **B** solution was quickly transferred into vial **A** via a syringe dropwise within 1 min. To a third flame-dried sealable 2-dram vial (vial **C**) was added acyloxyl carbamate **8a** (243 mg, 0.8 mmol, 1.6 equiv). Vial **C** was evacuated and backfilled with N_2 three times, and then anhydrous CH_2Cl_2 (0.5 mL) was added. The solution in vial **C** was added to vial **A** via a syringe dropwise within 1 min. The reaction mixture was kept at −78 °C for 5 min and transferred to −40 °C for an additional 3 h, then quenched by imidazole (20.4 mg in 1 mL CH_2Cl_2) and diluted with Et_2O (4 mL) subsequently at the same temperature. The mixture was stirred for 2 min at −40 °C and warmed up to room temperature. The solution was then filtered through a piece of cotton and washed with saturated $NaHCO_3$ solution (2 mL). The organic layer was separated from the aqueous one. The aqueous phase was further extracted with EtOAc (2 mL × 3). The combined organic layers were dried over anhydrous Na_2SO_4 and concentrated *in vacuo*. The residue was purified through a silica gel flash column (hexanes/EtOAc: from 100:1 to 6:1) to afford the desired product **23** as white foam (85 mg, 25%

yield), byproduct **10c** (60 mg, 20% yield) as white foam, and byproduct **25** as a white solid (96 mg, 36% yield based on glycosyl acceptor **22**, m.p. 163.4–163.7 °C).

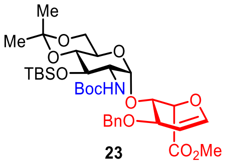

23

Methyl 3-*O*-benzyl-4-*O*-(2-*tert*-butoxycarbonylamino-3-*O*-*tert*-butyldimethylsilyl-4,6-*O*-isopropylidene-2-deoxy-α-D-glucopyranosyl)-L-guluronal (**23**): $[\alpha]_D^{23}$ −26.3 (acetone, *c* = 1.0); IR ν_{max} (neat)/cm^{-1}: 2953 (m), 2929 (m), 1767 (m), 1722 (s), 1506 (s), 1368 (s), 1249 (s); ^{1}H NMR (400 MHz, $CDCl_3$) δ 7.38 – 7.25 (m, 5H), 6.68 (d, *J* = 6.2 Hz, 1H), 4.99 (t, *J* = 5.0 Hz, 1H), 4.80 (d, *J* = 3.8 Hz, 1H), 4.64 – 4.60 (m, 2H), 4.57 (d, *J* = 11.7 Hz, 1H), 4.48 (d, *J* = 9.9 Hz, 1H), 4.28 (d, *J* = 1.7 Hz, 1H), 3.86 (s, 3H), 3.80 (dd, *J* = 10.5, 5.1 Hz, 1H), 3.76 – 3.68 (m, 2H), 3.66 (d, *J* = 10.4 Hz, 1H), 3.49 – 3.40 (m, 2H), 3.40 – 3.31 (m, 1H), 1.44 (s, 3H), 1.38 (s, 9H), 1.35 (s, 3H), 0.83 (s, 9H), 0.024 (s, 3H), 0.020 (s, 3H); ^{13}C NMR (100 MHz, $CDCl_3$) δ 168.6, 155.1, 146.0, 137.6, 128.5 (2C), 127.9, 127.7 (2C), 99.2, 98.4, 96.8, 79.3, 74.4, 72.5, 70.98, 70.95, 70.2, 65.8, 64.2, 62.0, 55.2, 52.5, 29.0, 28.4 (3C), 25.7 (3C), 18.9, 18.2, -4.2, -5.1; HRMS: m/z (ESI) calcd for $C_{34}H_{54}NO_{11}Si^+$, $[M + H]^+$, 680.3461, found 680.3455. $^1J^{13}{}_{C1–H1}$ = 170.3 Hz.

The C1 stereochemistry was determined by measuring $^1J^{13}{}_{C1–H1}$ through un-decoupled HSQC experiments. A $^1J^{13}{}_{C1–H1}$ value of 170.3 Hz suggested that the newly formed glycosidic bond is in axial position.

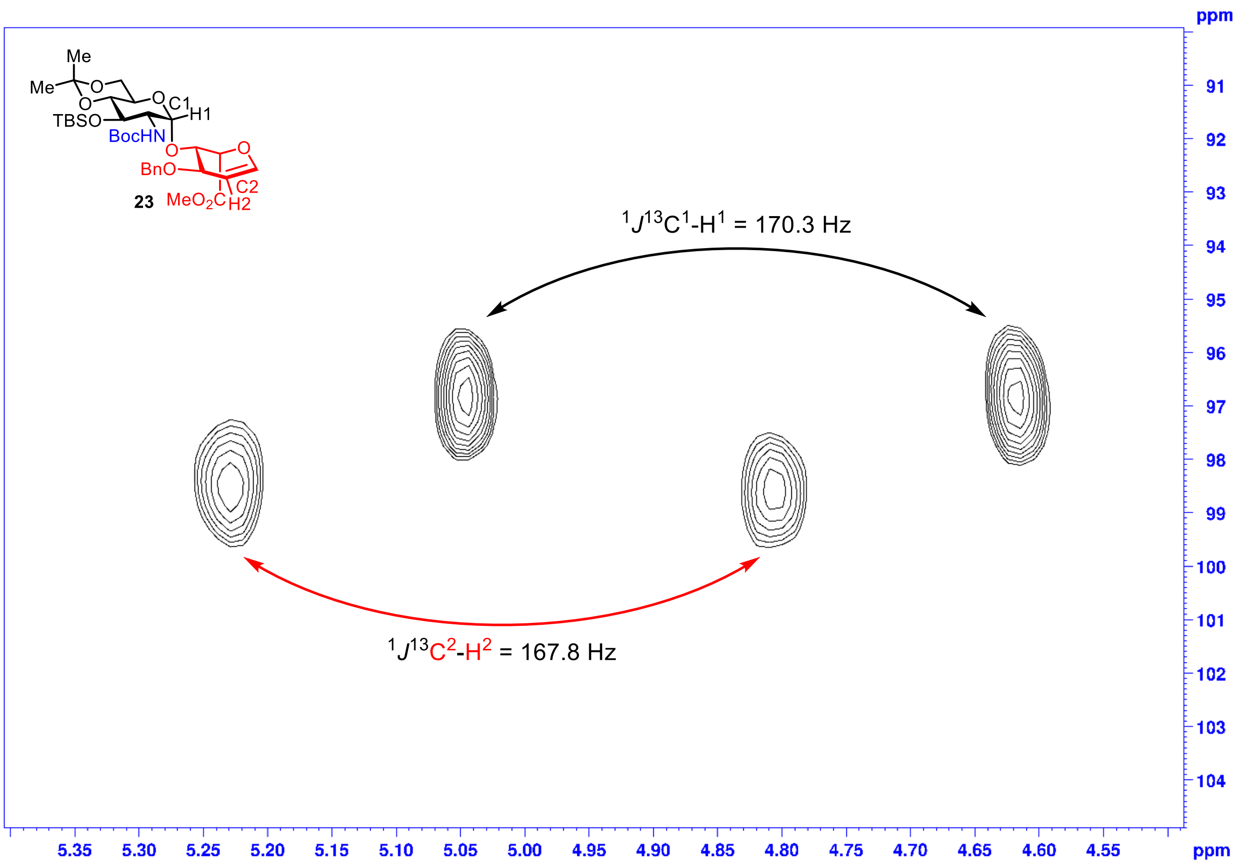

Figure S6a. Un-decoupled HSQC Analysis to Determine Stereochemistry of **23** at the C1 Position.

The C2 stereochemistry was determined by the *NOESY* experiment of **23** in C_6D_6: strong *NOE* was observed between H1 and H2, H2 and H4, and H3 and N-H; however, there was no *NOE* observed between H1 and H3. These data corroborated that the C–H2 bond is in axial position.

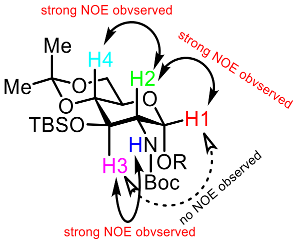

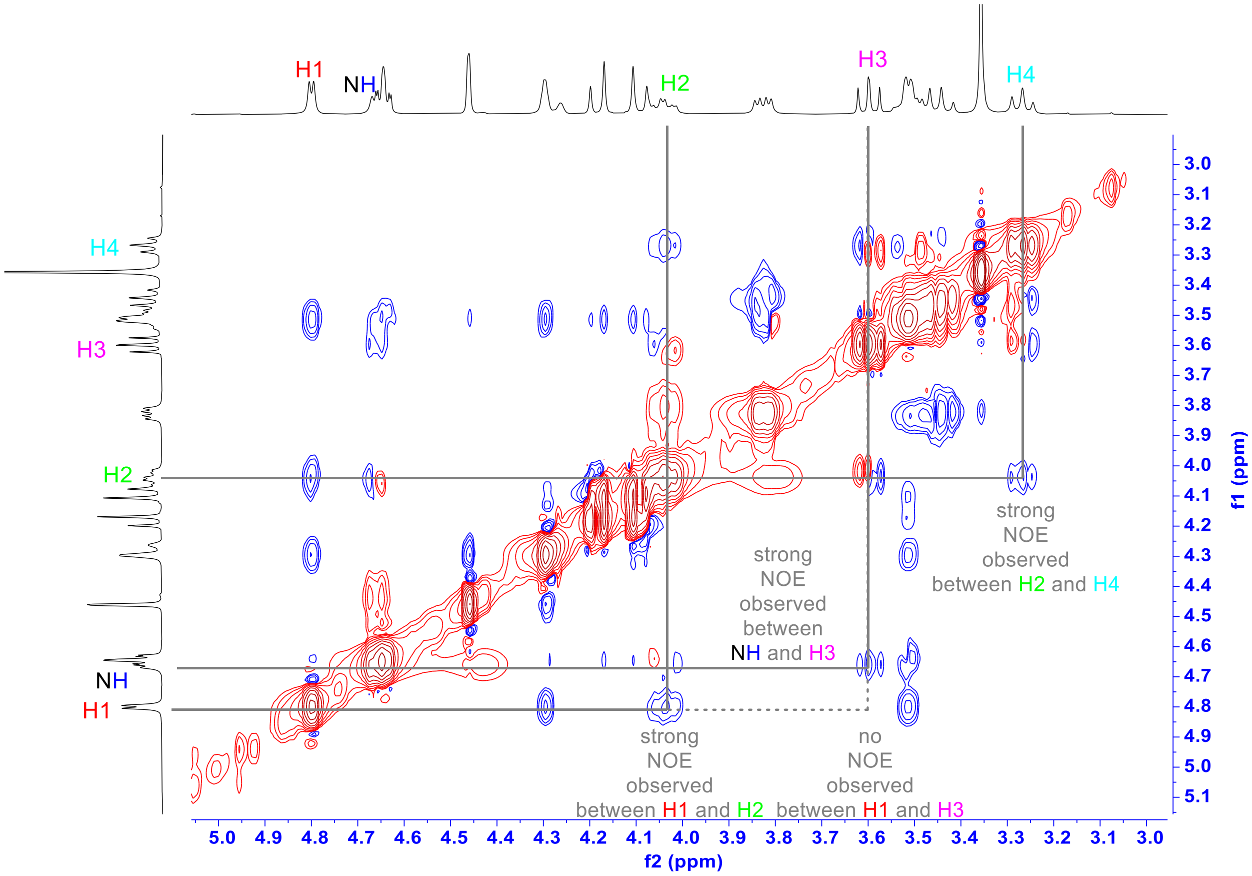

Figure S6b. The *NOESY* Experiment to Determine Stereochemistry of **23** at the C2 Position.

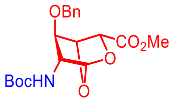

25

Methyl (1,4-anhydro-3-*O*-benzyl-2-*tert*-butoxycarbonylamino-2-deoxy-β-L-idopyranosid)uronate (**25**): $[\alpha]_D^{23}$ +0.1 (acetone, *c* = 0.5); IR ν_{max} (neat)/cm^{-1}: 3381 (w), 2967 (w), 1757 (s), 1701 (s), 1530 (s), 1210 (s), 1164 (s); ^1H NMR (400 MHz, $CDCl_3$) δ 7.40 – 7.28 (m, 5H), 5.52 (d, *J* = 1.1 Hz, 1H), 4.93 (d, *J* = 4.9 Hz, 1H), 4.83 (s, 1H), 4.79 (d, *J* = 12.1 Hz, 1H), 4.61 – 4.49 (m, 2H), 3.90 (d, *J* = 9.6 Hz, 1H), 3.76 (s, 3H), 3.69 (dt, *J* = 4.9, 1.3 Hz, 1H),

1.47 (s, 9H); ^{13}C NMR (100 MHz, $CDCl_3$) δ 170.6, 154.4, 136.9, 128.5 (2C), 128.1, 128.0 (2C), 104.9, 83.3, 80.2, 79.5, 72.5, 70.9, 58.7, 52.5, 28.3 (3C); HRMS: m/z (ESI) calcd for $C_{19}H_{26}NO_7^+$, $[M + H]^+$, 380.1704, found 380.1713.

The structure of **25** was determined based on the X-ray crystallographic analysis of an analogous cyclization product **S16** from glucuronal **6c**.

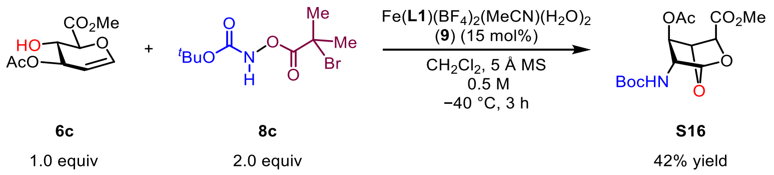

To a flame-dried sealable 2-dram vial (vial **A**) equipped with a stir bar were added glycal **6c** (108 mg, 0.5 mmol, 1.0 equiv), iron catalyst Fe(**L1**)$(BF_4)_2(MeCN)(H_2O)_2$ (**9**) (43.5 mg, 0.075 mmol, 15 mol %) and freshly activated 5 Å molecular sieves, powder (*ca.* 150 mg After the vial was evacuated and backfilled with N_2 three times, the vial was cooled to −78 °C. To a second flame-dried sealable 2-dram vial (vial **B**) was added acyloxyl carbamate **8c** (282 mg, 1 mmol, 2.0 equiv). Vial **B** was evacuated and backfilled with N_2 three times, and then anhydrous CH_2Cl_2 (0.5 mL) was added. The solution in vial **B** was added to vial **A** via a syringe dropwise within 1 min. The reaction mixture was kept at −78 °C for 5 min and transferred to −40 °C for an additional 3 h, then quenched by imidazole (20.4 mg in 1 mL CH_2Cl_2) and diluted with Et_2O (4 mL) subsequently at the same temperature. The mixture was stirred for 2 min at −40 °C and warmed up to room temperature. The solution was then filtered through a piece of cotton and washed with saturated $NaHCO_3$ solution (2 mL). The organic layer was separated from the aqueous one. The aqueous phase was further extracted with EtOAc (2 mL × 3). The combined organic layers were dried over anhydrous Na_2SO_4 and concentrated *in vacuo*. The residue was purified through a silica gel flash column (hexanes/EtOAc: from 100:1 to 3:2) to afford product **S16** as a white solid (70 mg, 42% yield, m.p. 154–155 °C).

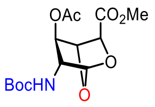

S16

Methyl (3-*O*-acetyl-1,4-anhydro-2-*tert*-butoxycarbonylamino-2-deoxy-α-D-glucopyranosid)uronate (**S16**): $[\alpha]_D^{22}$ +2.0 (acetone, *c* = 0.3); IR ν_{max} (neat)/cm^{-1}: 3371 (w), 2971 (w), 2360 (s), 1744 (s), 1716 (s), 1367 (s), 1229 (s); ^{1}H NMR (400 MHz, $CDCl_3$) δ 5.56 (s, 1H), 5.07 (t, *J* = 3.6 Hz, 1H), 4.86 – 4.69 (m, 2H), 4.42 (d, *J* = 3.6 Hz, 1H), 4.01 (s, 1H), 3.79 (s, 3H), 2.04 (s, 3H), 1.43 (s, 9H); ^{13}C NMR (100 MHz, $CDCl_3$) δ 169.9, 168.5, 154.7, 105.6, 80.3, 78.2, 76.3, 74.8, 59.4, 52.1, 28.2 (3C), 20.5; HRMS: m/z (ESI) calcd for $C_{14}H_{22}NO_8^+$, [M + H]$^+$, 332.1340, found 332.1336.

The structure of **S16** was elucidated by X-ray crystallographic analysis. Its structure has been deposited in CCDC with Deposition Number 2497373.

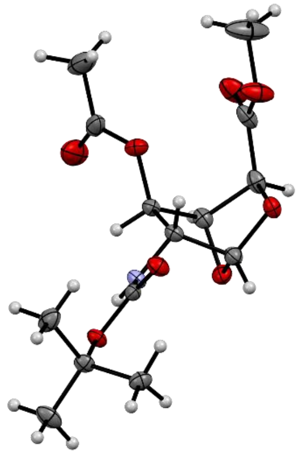

Figure S7. X-ray Crystallographic Analysis of **S16**.

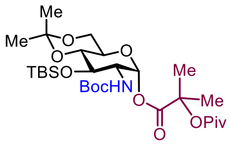

10c

2-*tert*-Butoxycarbonylamino-3-*O*-*tert*-butyldimethylsilyl-4,6-*O*-isopropylidene-2-deoxy-α-D-glucopyranosyl 2-pivaloyloxy-2-methylpropanoate (**10c**): $[\alpha]_D^{23}$ +43.8 (acetone, *c* = 1.0); IR ν_{max} (neat)/cm^{-1}: 3400 (w), 1761 (m), 1721 (s), 1367 (m), 1139 (s), 1078 (s), 994 (s); ^{1}H NMR (400 MHz, $CDCl_3$) δ 6.15 (d, *J* = 3.6 Hz, 1H), 4.70 (d, *J* = 9.9 Hz, 1H), 3.99 (ddd, *J* = 9.9, 9.6, 3.6 Hz, 1H), 3.84 (dd, *J* = 10.3, 3.8 Hz, 1H), 3.75 – 3.64 (m, 1H), 3.64 – 3.48 (m, 3H), 1.56 (s, 6H), 1.46 (s, 3H), 1.41 (s, 9H), 1.38 (s, 3H), 1.23 (s, 9H), 0.85 (s, 9H), 0.04 (s, 6H); ^{13}C NMR (100 MHz, $CDCl_3$) δ 177.8, 170.9, 155.3, 99.5, 93.4, 79.5, 77.8, 74.4, 71.3, 66.2, 62.1, 54.7, 38.6, 28.9, 28.4 (3C), 26.9 (3C), 25.7 (3C), 24.6, 24.1, 18.9, 18.2, -4.2, -5.2; HRMS: m/z (ESI) calcd for $C_{29}H_{54}NO_{10}Si^+$, $[M + H]^+$, 604.3511, found 604.3522. $^1J_{^{13}C1-H1}$ = 178.2 Hz.

b. Attempted Direct Epimerization of GlcN-α-1,4-Glucuronal Module 11 to Generate GlcN-α-1,4-Iduronal Module 23.

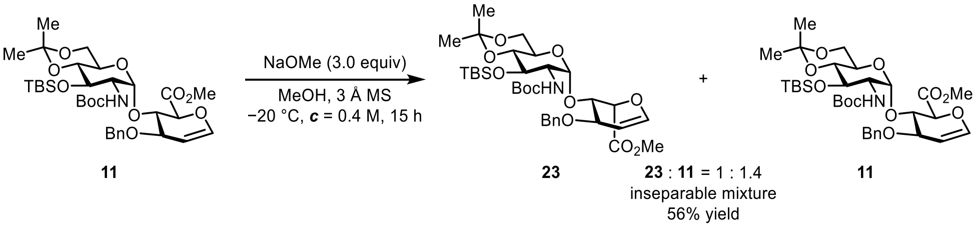

Figure S8. Attempted Direct Epimerization of GlcN-α-1,4-Glucuronal Module **11** to Generate GlcN-α-1,4-Iduronal Module **23**.

To a flame-dried sealable 2-dram vial equipped with a stir bar, compound **11** (500 mg, 0.735 mmol, 1.0 equiv) and freshly activated 3 Å molecular sieves powder (*ca.* 200 mg) were added. After the vial was evacuated and backfilled with N_2 three times, anhydrous MeOH (1.1 mL) was

added, and the solution was cooled to –20 °C. Freshly prepared NaOMe solution (3.0 M solution in anhydrous MeOH, 0.735 mL, 2.21 mmol, 3.0 equiv) was added dropwise into the reaction mixture over 10 min at –20 °C. After 15 h, the reaction mixture was transferred via syringe to a 50 mL flame-dried round-bottom flask containing a stirred suspension of Amberlite® IRC 120 H (1.6 g) in anhydrous MeOH (10 mL) at –20 °C. The resulting mixture was stirred for an additional 5 mins until *p*H reached 7. The mixture was subsequently filtered, and the filtrate was concentrated *in vacuo*. The residue was purified through a silica gel flash column (hexanes/EtOAc: from 100:1 to 6:1) to afford an inseparable mixture of **23** and **11** as white foam (**23** : **11** = 1 : 1.4, 280 mg, 56% yield).

c. Epimerization of GlcN-α-1,4-Glucuronal Modules 26 to Generate GlcN-α-1,4-Iduronal Module 27.

Synthesis of 26 from 10a and 12 for Epimerization of 26

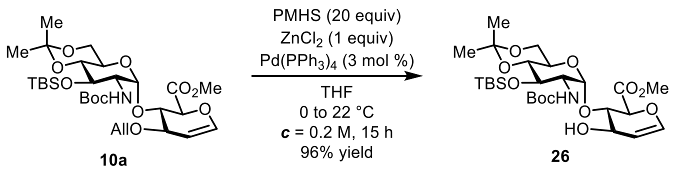

To a 100 mL flame-dried round-bottom flask equipped with a stir bar were added compound **10a** (3.0 g, 4.77 mmol, 1.0 equiv), $Pd(PPh_3)_4$ (165 mg, 0.144 mmol, 3 mol %), and polymethylhydrosiloxane (5.73 g, 95.4 mmol, 20 equiv).[9] After the flask was evacuated and backfilled with N_2 three times, anhydrous THF (23.85 mL) was added. The solution was then cooled to 0 °C before $ZnCl_2$ (1 M solution in Et_2O, 4.77 mL, 4.77 mmol, 1.0 equiv) was added dropwise. The reaction mixture was gradually warmed to room temperature and stirred for an additional 15 h until the starting material **10a** was fully consumed (monitored by TLC). The reaction mixture was then diluted with EtOAc (40 mL) and quenched with saturated aqueous $NaHCO_3$ solution (20 mL). The organic phase was separated from the aqueous one, which was further extracted with EtOAc (40 mL × 2). The combined organic phase was washed with brine (40 mL) and dried over Na_2SO_4. After concentration *in vacuo*, the residue was purified through

a silica gel flash column hexanes/EtOAc: from 100:1 to 2:1) to afford the desired product **26** as white foam (2.7 g, 96% yield).

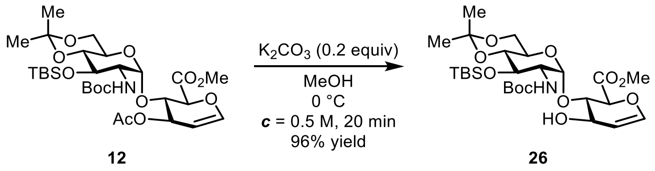

To a 50 mL flame-dried round bottom flask equipped with a stir bar were added compound **12** (4.0 g, 6.34 mmol, 1.0 equiv) and anhydrous MeOH (12.6 mL). The solution was cooled to 0 °C, and anhydrous K_2CO_3 (powder, 176 mg, 1.26 mmol, 0.2 equiv) was then added. The reaction mixture was stirred for 20 min at 0 °C, with progress monitored by TLC until completion. Amberlite® IRC 120 H (1.42 g) was then added to the mixture at 0 °C to quench the reaction. The reaction mixture was filtered through a piece of cotton (rinsed with 6 mL of MeOH), and the filtrate was concentrated *in vacuo*. The residue was purified through a silica gel flash column (hexanes/EtOAc: from 100:1 to 2:1) to afford the desired product **26** as white foam (3.58 g, 96% yield).

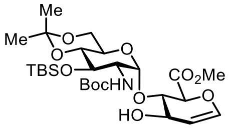

26

Methyl 4-*O*-(2-*tert*-butoxycarbonylamino-3-*O*-*tert*-butyldimethylsilyl-4,6-*O*-isopropylidene-2-deoxy-α-D-glucopyranosyl)-D-glucuronal (**26**): $[\alpha]_D^{23}$ +72.0 (acetone, *c* = 1.0); IR ν_{max} (neat)/cm^{-1}: 3455 (w), 2953 (m), 1723 (s), 1506 (s), 1369 (s), 1249 (s), 1172 (s), 1131 (s), 1080 (s), 1040 (s); ^1H NMR (400 MHz, $CDCl_3$) δ 6.62 (d, *J* = 6.2 Hz, 1H), 5.08 (d, *J* = 3.5 Hz, 1H), 5.03 – 4.93 (m, 1H), 4.84 – 4.76 (m, 1H), 4.56 (d, *J* = 9.5 Hz, 1H), 4.19 (s, 1H), 4.02 (s, 1H), 3.81 (td, *J* = 10.6, 4.1 Hz, 2H), 3.75 (s, 3H), 3.69 (t, *J* = 10.3 Hz, 1H), 3.58 (td, *J* = 9.5, 4.9 Hz, 1H), 3.52 – 3.48 (m, 2H), 1.92 (s, 1H), 1.46 (s, 3H), 1.42 (s, 9H), 1.38 (s, 3H), 0.85 (s, 9H), 0.04 (s, 3H), 0.04 (s, 3H); ^{13}C NMR (100 MHz, $CDCl_3$) δ 168.8, 155.2, 145.1, 100.5, 99.3, 99.2, 79.7,

74.8, 74.5, 73.8, 71.0, 64.3, 62.1, 61.8, 55.5, 52.3, 29.0, 28.4 (3C), 25.7 (3C), 18.9, 18.2, -4.1, -5.1; HRMS: m/z (ESI) calcd for $C_{27}H_{48}NO_{11}Si^+$, $[M + H]^+$, 590.2991, found 590.3002.

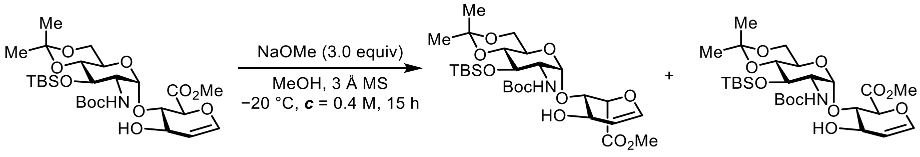

26

27
32% yield

26
58% (recovered)

To a 100 mL flame-dried round-bottom flask equipped with a stir bar, compound **26** (4.5 g, 7.63 mmol, 1.0 equiv) and freshly activated 3 Å molecular sieves powder (*ca*. 500 mg) were added. After the flask was evacuated and backfilled with N_2 three times, anhydrous MeOH (11.45 mL) was added, and the solution was cooled to –20 °C. Freshly prepared NaOMe solution (3.0 M solution in anhydrous MeOH, 7.63 mL, 22.89 mmol, 3.0 equiv) was added dropwise into the reaction mixture over 10 min at –20 °C. After 15 h, the reaction mixture was transferred via syringe to a 250 mL flame-dried round-bottom flask containing a stirred suspension of Amberlite® IRC 120 H (16.1 g) in anhydrous MeOH (50 mL) at –20 °C. The resulting mixture was stirred for an additional 5 mins until *p*H reached 7. The mixture was subsequently filtered, and the filtrate was concentrated *in vacuo*. The residue was purified through a silica gel flash column (hexanes/EtOAc: from 100:1 to 2:1) to afford the mixture of compound **27** and **26** as white foam (**27** : **26** = 1 : 1.8, 4.05 g, 90% yield).

The obtained foam (**26** and **27**) was transferred into a 250 mL round bottom flask equipped with a stir bar. Hexanes (80 mL) were added to the flask and the mixture was stirred at 60 °C until all the solids had dissolved. The flask was then gradually cooled to room temperature and further cooled to 0 °C for recrystallization. The mixture was filtered through a Büchner funnel and rinsed with cold hexanes (20 mL): **27** was collected as a white solid (940 mg, *dr* >20:1, 65% recovery of **27** from the mixture, m.p. 146.2–147.5 °C). The mother liquor was concentrated *in vacuo* and the resulting foam (3.11 g) was subjected to another recrystallization using 30 mL of

hexanes at 60 °C. **27** was obtained again as a white solid (250 mg, *dr* >20:1, 49% recovery of **27** from the mixture).

The recovered mixture (2.86 g, 4.85 mmol, 1.0 equiv) was subjected to another round of epimerization at –20 °C. The reaction progress was then monitored (until **27** : **26** = 1 : 1.8) by ^{1}H NMR analysis of aliquots that were quenched using Amberlite® IRC in anhydrous MeOH.

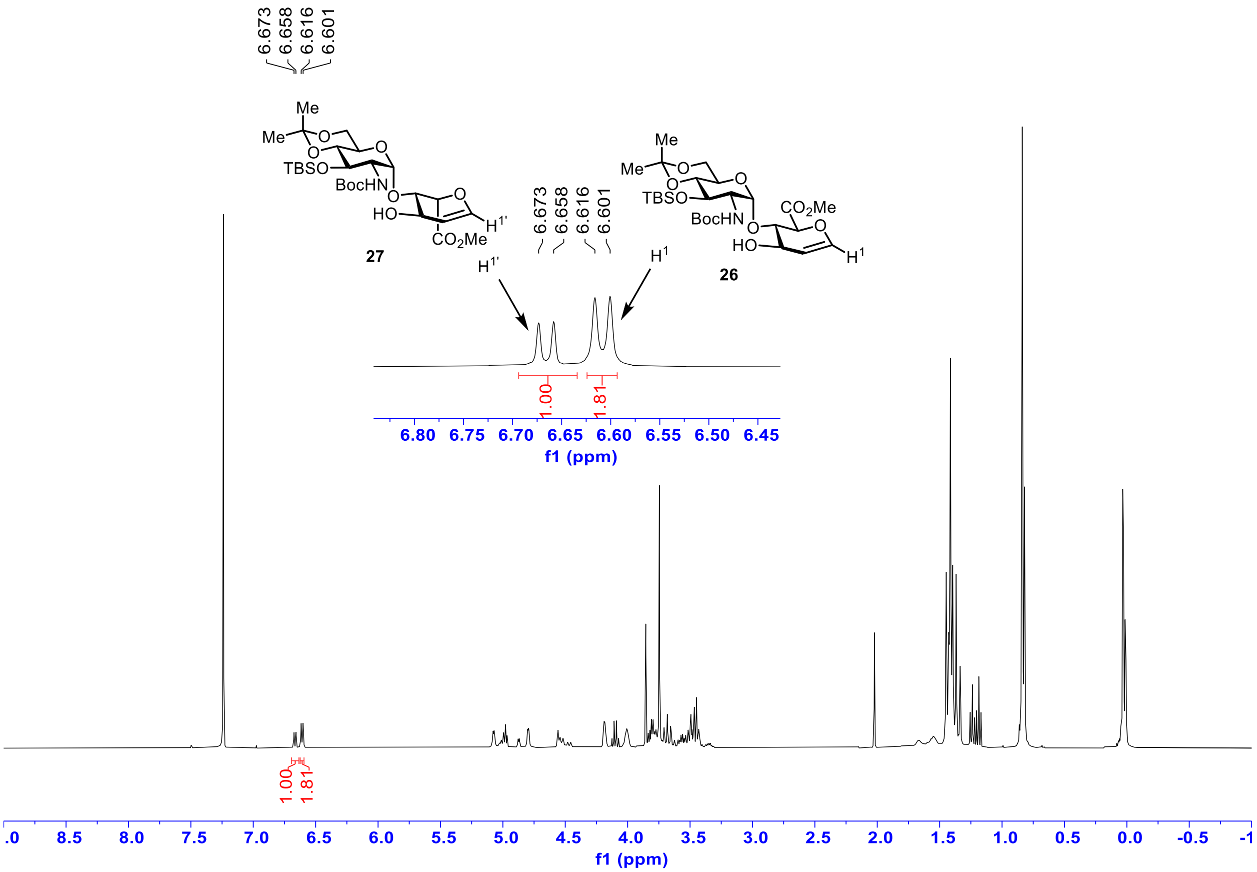

Figure S9. ^{1}H NMR Analysis of the Crude Epimerization Mixture.

Around 10 h, the ratio of epimerized product **27** *vs* unepimerized starting material **26** reached 1 : 1.8, as determined by ^{1}H NMR analysis (**Figure S9**). Further reaction will induce significant decomposition of **27** to afford the β-elimination product. Subjecting product **27** under the epimerization condition does not convert it back to the unepimerized starting material **26**.

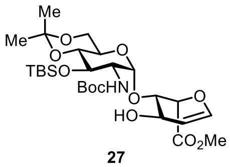

27

Methyl 4-*O*-(2-*tert*-butoxycarbonylamino-3-*O*-*tert*-butyldimethylsilyl-4,6-*O*-isopropylidene-2-deoxy-α-D-glucopyranosyl)-L-guluronal (**27**): $[\alpha]_D^{23}$ +10.3 (acetone, *c* = 1.0); IR ν_{max} (neat)/cm^{-1}: 3431 (w), 2930 (w), 1700 (m), 1512 (m), 1367 (m), 1248 (s), 1170 (s), 1083 (s), 1035 (s), 1001 (s); ^{1}H NMR (400 MHz, $CDCl_3$) δ 6.67 (d, *J* = 6.1 Hz, 1H), 5.02 (td, *J* = 5.8, 1.8 Hz, 1H), 4.88 (d, *J* = 4.0 Hz, 1H), 4.58 (d, *J* = 1.7 Hz, 1H), 4.52 (d, *J* = 9.9 Hz, 1H), 4.20 (d, *J* = 2.1 Hz, 1H), 4.00 (td, *J* = 5.0, 2.3 Hz, 1H), 3.86 (s, 3H), 3.80 (dd, *J* = 10.6, 5.2 Hz, 1H), 3.72 (td, *J* = 9.1, 4.0 Hz, 1H), 3.66 (t, *J* = 10.4 Hz, 1H), 3.51 – 3.40 (m, 2H), 3.36 – 3.34 (m, 1H), 2.33 (d, *J* = 5.4 Hz, 1H), 1.44 (s, 3H), 1.40 (s, 9H), 1.34 (s, 3H), 0.83 (s, 9H), 0.02 (s, 3H), 0.02 (s, 3H); ^{13}C NMR (100 MHz, $CDCl_3$) δ 168.8, 155.3, 146.0, 100.3, 99.3, 96.9, 79.5, 74.5, 73.1, 71.9, 71.0, 64.2, 62.0, 59.5, 55.3, 52.7, 29.1, 28.4 (3C), 25.7 (3C), 18.9, 18.2, -4.1, -5.0; HRMS: m/z (ESI) calcd for $C_{27}H_{48}NO_{11}Si^+$, $[M + H]^+$, 590.2991, found 590.2986.

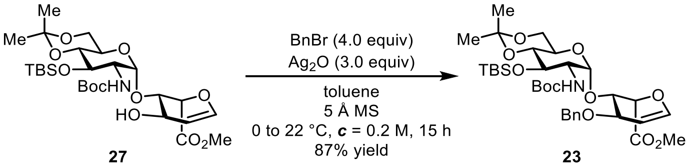

To a 100 mL flame-dried round-bottom flask equipped with a stir bar, compound **27** (1.0 g, 1.70 mmol, 1.0 equiv), Ag_2O (1.18 g, 5.09 mmol, 3.0 equiv), and freshly activated 5 Å molecular sieves (powder, *ca.* 300 mg) were added. After the flask was evacuated and backfilled with N_2 three times, anhydrous toluene (8.5 mL) was added. The mixture was cooled to 0 °C before freshly distilled benzyl bromide (0.81 mL, 6.78 mmol, 4.0 equiv) was added dropwise. The reaction mixture was then stirred at 22 °C for 18 h until the starting material **27** was fully consumed (monitored by TLC). The mixture was filtered through a short pad of Celite®, further rinsed with acetone (20 mL). The filtrate was concentrated *in vacuo*, and the residue was

purified through a silica gel flash column (hexanes/EtOAc: from 100:1 to 5:1) to afford product **23** as white foam (1.0 g, 87 % yield).

d. The Synthesis of GlcN-α-1,4-Iduronal Module Acceptors

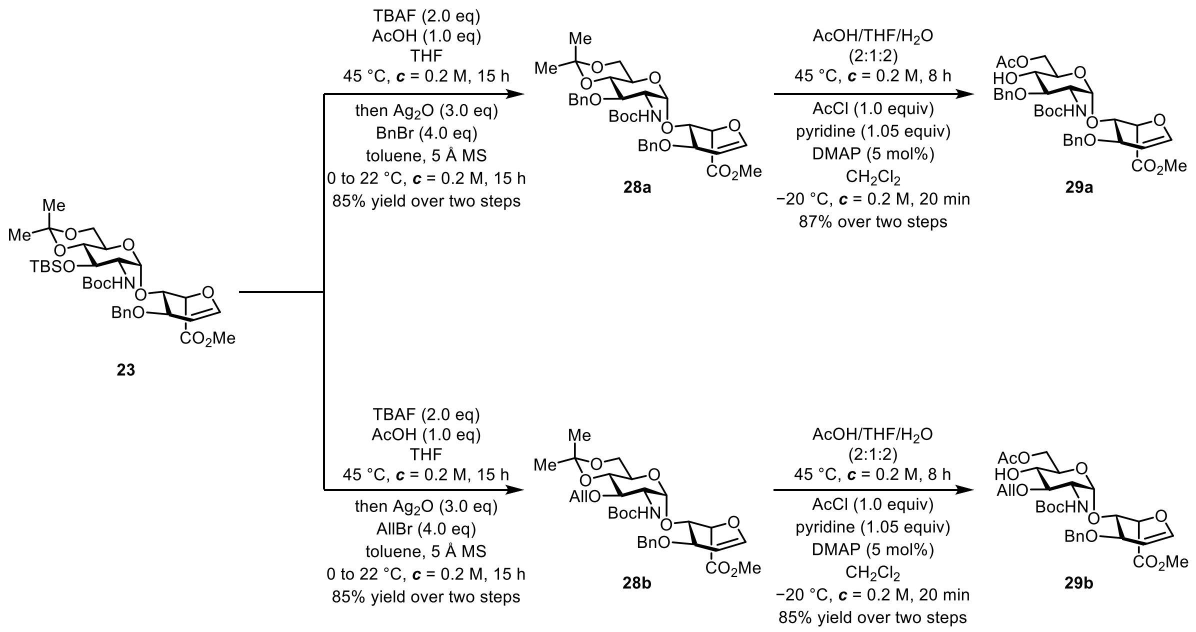

Figure S10. Conversion of GlcN-α-1,4-Iduronal Donor **23** to Acceptor **29**.

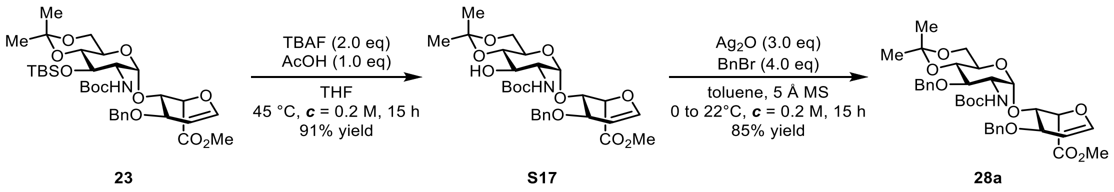

To a 100 mL flame-dried round-bottom flask equipped with a stir bar, compound **23** (1.72 g, 2.53 mmol, 1.0 equiv) and anhydrous THF (7.2 mL) were added. To the mixture was then added

a premixed solution of TBAF (1 M solution in THF, 5 mL, 5 mmol, 2.0 equiv) and AcOH (0.14 mL, 2.53 mmol, 1.0 equiv). The reaction mixture was stirred for 15 h at 45 °C until the starting material **23** was fully consumed (monitored by TLC). The reaction mixture was then cooled down to room temperature and diluted with EtOAc (10 mL) and quenched with saturated aqueous $NaHCO_3$ solution (10 mL). The organic phase was separated from the aqueous one, and the aqueous phase was further extracted with EtOAc (15 mL × 3). The combined organic phase was washed with brine (20 mL), dried over Na_2SO_4, and concentrated *in vacuo*. The residue was purified through a silica gel flash column (hexanes/EtOAc: from 100:1 to 3:2) to afford the desired product **S17** (1.30 g, 91% yield) as white foam.

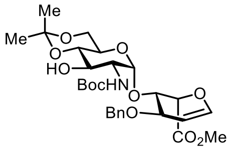

S17

Methyl 3-*O*-benzyl-4-*O*-(2-*tert*-butoxycarbonylamino-4,6-*O*-isopropylidene-2-deoxy-α-D-glucopyranosyl)-L-guluronal (**S17**): $[\alpha]_D^{23}$ −31.7 (acetone, *c* = 0.5); IR ν_{max} (neat)/cm^{-1}: 3442 (w), 2978 (w), 1711 (s), 1509 (m), 1367 (m), 1248 (m), 1209 (m), 1169 (s), 1147 (s), 1068 (s), 1028 (s), 1015 (s); ^{1}H NMR (400 MHz, $CDCl_3$) δ 7.37 – 7.23 (m, 5H), 6.64 (d, *J* = 6.2 Hz, 1H), 4.97 (t, *J* = 4.7 Hz, 1H), 4.87 (s, 1H), 4.73 (d, *J* = 7.8 Hz, 1H), 4.65 – 4.58 (m, 2H), 4.55 (d, *J* = 11.8 Hz, 1H), 4.32 – 4.26 (m, 1H), 3.83 (s, 3H), 3.83 – 3.78 (m, 1H), 3.73 – 3.65 (m, 3H), 3.58 (t, *J* = 9.4 Hz, 1H), 3.50 (t, *J* = 9.2 Hz, 1H), 3.35 (td, *J* = 10.0, 5.3 Hz, 1H), 2.69 (brs, 1H), 1.47 (s, 3H), 1.38 (s, 9H), 1.37 (s, 3H); ^{13}C NMR (100 MHz, $CDCl_3$) δ 168.6, 156.0, 146.0, 137.5, 128.5 (2C), 127.9, 127.6 (2C), 99.7, 98.5, 95.9, 80.0, 74.1, 72.4, 70.9, 70.1, 69.9, 65.9, 63.9, 61.8, 55.0, 52.5, 29.0, 28.2 (3C), 19.0; HRMS: m/z (ESI) calcd for $C_{28}H_{40}NO_{11}^+$, $[M + H]^+$, 566.2596, found 566.2583.

To a 50 mL flame-dried round-bottom flask equipped with a stir bar, the crude desilylated product **S17** from the previous step (1.30 g, 2.30 mmol, 1.0 equiv), Ag_2O (1.60 g, 6.91 mmol, 3.0 equiv), freshly activated 5 Å molecular sieves (powder, *ca.* 300 mg), and anhydrous toluene (11.5 mL) were added. The mixture was cooled to 0 °C before freshly distilled benzyl bromide

(1.09 mL, 9.20 mmol, 4.0 equiv) was added dropwise. The reaction mixture was then stirred at room temperature for 15 h until the starting material **S17** was fully consumed (monitored by TLC). The reaction mixture was then filtered through a short pad of Celite®, further rinsed with acetone (10 mL), and concentrated *in vacuo*. The residue was purified through a silica gel flash column (hexanes/EtOAc: from 100:1 to 5:1) to afford the desired product **28a** (1.28 g, 85% yield) as white foam.

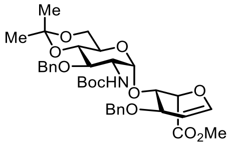

28a

Methyl 3-*O*-benzyl-4-*O*-(3-*O*-benzyl-2-*tert*-butoxycarbonylamino-4,6-*O*-isopropylidene-2-deoxy-α-D-glucopyranosyl)-L-guluronal (**28a**): $[\alpha]_D^{23}$ −13.7 (acetone, *c* = 1.0); IR ν_{max} (neat)/cm^{-1}: 3431 (w), 2976 (w), 1716 (s), 1501 (m), 1367 (s), 1249 (s), 1211 (s), 1170 (s), 1126 (s), 1067 (s), 1036 (s); ^{1}H NMR (400 MHz, $CDCl_3$) δ 7.39 – 7.22 (m, 10H), 6.67 (d, *J* = 6.2 Hz, 1H), 4.99 (t, *J* = 5.1 Hz, 1H), 4.89 (d, *J* = 3.5 Hz, 1H), 4.83 (d, *J* = 11.9 Hz, 1H), 4.66 – 4.51 (m, 5H), 4.31 (s, 1H), 3.87 (s, 3H), 3.85 (d, *J* = 4.9 Hz, 1H), 3.82 (d, *J* = 5.0 Hz, 1H), 3.76 – 3.67 (m, 3H), 3.46 – 3.38 (m, 2H), 1.46 (s, 3H), 1.40 (s, 9H), 1.39 (s, 3H); ^{13}C NMR (100 MHz, $CDCl_3$) δ 168.6, 155.3, 145.9, 138.7, 137.5, 128.4 (2C), 128.0 (2C), 127.8, 127.6 (2C), 127.3 (2C), 127.2, 99.2, 98.5, 96.6, 79.4, 77.1, 74.8, 74.1, 72.4, 71.1, 70.1, 66.0, 64.2, 62.0, 53.6, 52.5, 29.1, 28.2 (3C), 19.0; HRMS: m/z (ESI) calcd for $C_{35}H_{46}NO_{11}^+$, $[M + H]^+$, 656.3065, found 656.3057.

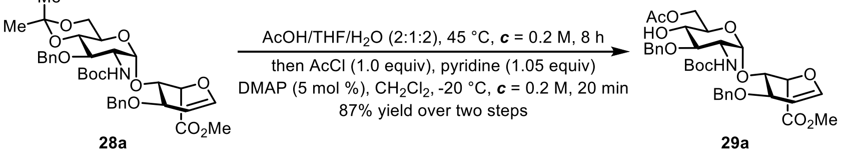

28a **29a**

To a 50 mL flame-dried round-bottom flask equipped with a stir bar, compound **28a** (1.28 g, 1.95 mmol, 1.0 equiv) and a mixture of AcOH/THF/H_2O (2:1:2 vol/vol/vol, 9.7 mL) were added. The mixture was stirred at 45 °C for 8 h until the starting material **28a** was fully consumed (monitored by TLC). The reaction mixture was concentrated *in vacuo* and azeotropically dried with toluene (7 mL × 4) to remove AcOH and H_2O. The crude hydrolysis product was obtained as white foam and directly used in the next step without further purification.

To a 50 mL flame-dried round-bottom flask equipped with a stir bar, the crude hydrolysis product (1.95 mmol, 1.0 equiv) from the last step, DMAP (12 mg, 0.1 mmol, 5 mol %), and anhydrous CH_2Cl_2 (7.8 mL) were added. The mixture was cooled to –20 °C before anhydrous pyridine (0.17 mL, 2.05 mmol, 1.05 equiv) and acetyl chloride (1 M solution in CH_2Cl_2, 1.95 mL, 1.95 mmol, 1.0 equiv) were added dropwise sequentially. The reaction mixture was stirred at –20 °C for 20 min until the starting material was fully consumed (monitored by TLC). The reaction mixture was then quenched with saturated aqueous NH_4Cl solution (10 mL) and the organic phase was separated from the aqueous one. The aqueous phase was further extracted with CH_2Cl_2 (10 mL × 2). The combined organic phase was washed with brine (15 mL) and dried over Na_2SO_4. After concentration *in vacuo*, the residue was purified through a silica gel flash column (hexanes/EtOAc: from 100:1 to 2:1) to afford the desired product **29a** (1.12 g, 87% yield over two steps) as white foam.

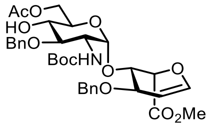

29a

Methyl 3-*O*-benzyl-4-*O*-(6-*O*-acetyl-3-*O*-benzyl-2-*tert*-butoxycarbonylamino-2-deoxy-α-D-glucopyranosyl)-L-guluronal (**29a**): $[\alpha]_D^{23}$ −3.8 (acetone, *c* = 2.0); IR ν_{max} (neat)/cm^{-1}: 3433 (w), 2975 (w), 1740 (s), 1718 (s), 1507 (m), 1367 (m), 1246 (s), 1168 (m), 1062 (s), 1035 (s); ^{1}H NMR (400 MHz, $CDCl_3$) δ 7.36 – 7.24 (m, 10H), 6.65 (d, *J* = 6.2 Hz, 1H), 4.98 (t, *J* = 6.1 Hz, 2H), 4.87 (d, *J* = 2.8 Hz, 1H), 4.71 (s, 2H), 4.65 – 4.60 (m, 2H), 4.60 – 4.54 (m, 1H), 4.41 (dd, *J* = 12.0, 2.9 Hz, 1H), 4.33 (d, *J* = 1.4 Hz, 1H), 4.25 (d, *J* = 11.9 Hz, 1H), 3.87 – 3.82 (m, 1H),

3.80 (s, 3H), 3.74 (dd, J = 5.5, 2.3 Hz, 1H), 3.59 – 3.52 (m, 1H), 3.47 (t, J = 8.8 Hz, 1H), 3.42 – 3.36 (m, 1H), 2.91 (d, J = 2.2 Hz, 1H), 2.08 (s, 3H), 1.38 (s, 9H); ^{13}C NMR (100 MHz, $CDCl_3$) δ 171.4, 168.6, 155.1, 145.9, 138.2, 137.5, 128.4 (2C), 128.3 (2C), 127.8, 127.7 (2C), 127.60, 127.56 (2C), 98.4, 96.0, 79.7, 79.5, 74.5, 72.3, 70.8 (two peaks overlapped, 2C), 70.0, 69.7, 65.5, 62.7, 53.3, 52.5, 28.2 (3C), 20.7; HRMS: m/z (ESI) calcd for $C_{34}H_{44}NO_{12}^+$, $[M + H]^+$, 658.2858, found 658.2871.

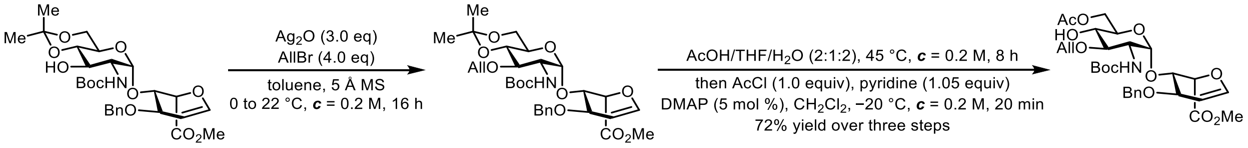

The synthesis of **29b** was carried out on a 2.53 mmol scale using analogous procedures to obtain **29a**. The desired product **29b** was purified through a silica gel flash column (hexanes/EtOAc: from 100:1 to 2:1) as white foam (1.11 g, 72% yield over three steps).

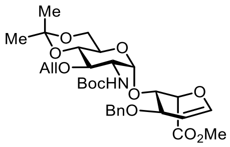

28b

Methyl 3-*O*-benzyl-4-*O*-(3-*O*-allyl-2-*tert*-butoxycarbonylamino-4,6-*O*-isopropylidene-2-deoxy-α-D-glucopyranosyl)-L-guluronal (**28b**): $[\alpha]_D^{23}$ −20.0 (acetone, c = 1.0); IR ν_{max} (neat)/cm^{-1}: 3446 (w), 2990 (w), 1717 (s), 1506 (m), 1367 (m), 1247 (s), 1204 (s), 1128 (s), 1070 (s), 1036 (s); ^{1}H NMR (400 MHz, $CDCl_3$) δ 7.44 – 7.28 (m, 5H), 6.67 (d, J = 6.2 Hz, 1H), 5.82 (ddt, J = 17.3, 10.7, 5.3 Hz, 1H), 5.22 (dd, J = 17.3, 1.8 Hz, 1H), 5.10 (dd, J = 10.3, 1.8 Hz, 1H), 5.00 (t, J = 5.2 Hz, 1H), 4.88 (d, J = 3.9 Hz, 1H), 4.64 – 4.59 (m, 3H), 4.56 – 4.52 (m, 1H), 4.34 – 4.24 (m, 2H), 4.01 (dd, J = 13.0, 5.7 Hz, 1H), 3.87 (s, 3H), 3.80 (td, J = 10.4, 4.6 Hz, 2H), 3.75 – 3.68 (m, 2H), 3.68 – 3.61 (m, 1H), 3.38 (dt, J = 10.1, 5.1 Hz, 1H), 3.31 (t, J = 9.6 Hz, 1H),

1.47 (s, 3H), 1.40 (s, 9H), 1.36 (s, 3H); ^{13}C NMR (100 MHz, $CDCl_3$) δ 168.7, 155.5, 146.0, 137.6, 135.1, 128.5 (2C), 128.0, 127.7 (2C), 116.2, 99.3, 98.7, 96.5, 79.5, 77.1, 74.8, 73.1, 72.5, 71.0, 70.2, 66.0, 64.3, 62.1, 53.7, 52.6, 29.2, 28.3 (3C), 19.1; HRMS: m/z (ESI) calcd for $C_{31}H_{44}NO_{11}^+$, $[M + H]^+$, 606.2909, found 606.2917.

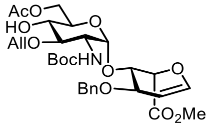

29b

Methyl 3-*O*-benzyl-4-*O*-(6-*O*-acetyl-3-*O*-allyl-2-*tert*-butoxycarbonylamino-2-deoxy-α-D-glucopyranosyl)-L-guluronal (**29b**): $[\alpha]_D^{23}$ −12.8 (acetone, *c* = 1.0); IR ν_{max} (neat)/cm^{-1}: 3442 (w), 2976 (w), 1739 (s), 1719 (s), 1506 (m), 1246 (s), 1171 (m), 1103 (m), 1061 (s), 1035 (s), 1003 (m); ^{1}H NMR (400 MHz, $CDCl_3$) δ 7.54 – 7.25 (m, 5H), 6.67 (d, *J* = 6.2 Hz, 1H), 5.88 (ddt, *J* = 17.3, 10.9, 5.6 Hz, 1H), 5.26 (dd, *J* = 17.3, 1.7 Hz, 1H), 5.15 (dd, *J* = 10.9, 1.7 Hz, 1H), 5.01 (t, *J* = 5.8 Hz, 1H), 4.88 (d, *J* = 3.7 Hz, 1H), 4.72 – 4.54 (m, 4H), 4.45 (dd, *J* = 12.3, 5.6 Hz, 1H), 4.34 (d, *J* = 2.0 Hz, 1H), 4.27 – 4.13 (m, 3H), 3.84 (s, 3H), 3.76 (dd, *J* = 5.3, 2.1 Hz, 2H), 3.58 – 3.53 (m, 1H), 3.45 (t, *J* = 9.5 Hz, 1H), 3.28 (t, *J* = 9.6 Hz, 1H), 2.91 (brs, 1H), 2.11 (s, 3H), 1.41 (s, 9H); ^{13}C NMR (100 MHz, $CDCl_3$) δ 171.6, 168.7, 155.2, 146.1, 137.6, 134.8, 128.6 (2C), 128.0, 127.7 (2C), 117.1, 98.6, 96.2, 79.9, 79.7, 73.6, 72.4, 70.9, 70.8, 70.2, 69.8, 65.7, 62.7, 53.5, 52.6, 28.3 (3C), 20.8; HRMS: m/z (ESI) calcd for $C_{30}H_{42}NO_{12}^+$, $[M + H]^+$, 608.2702, found 608.2722.

E. The Synthesis of GlcN-α-1,4-Glucuronal Acceptors

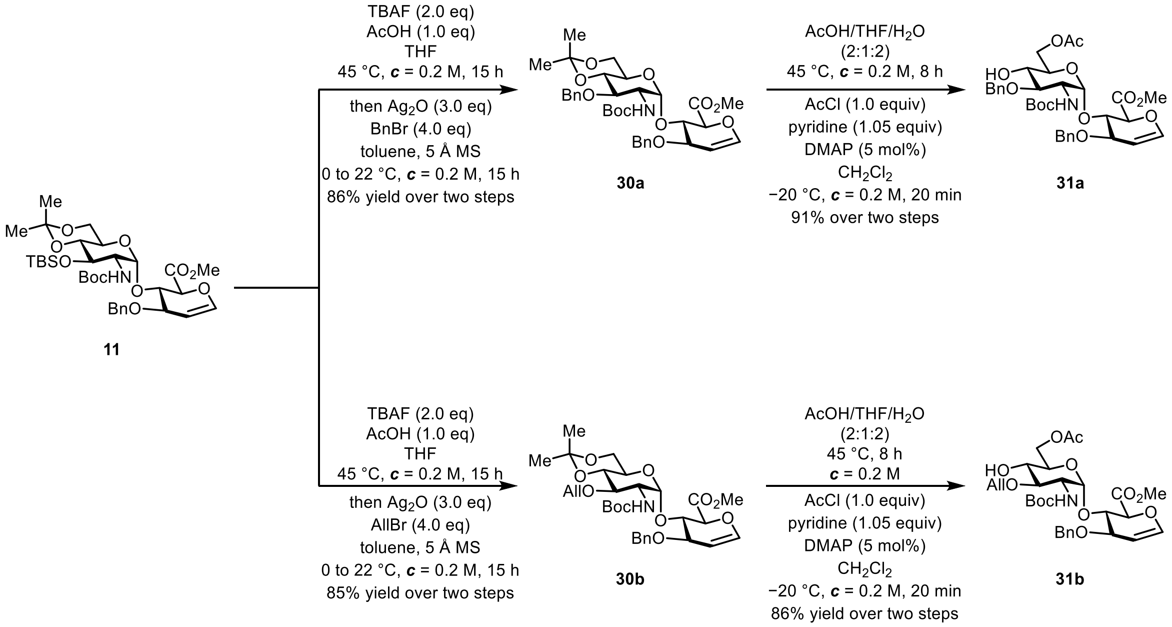

Figure S11. Conversion of the GlcN-α-1,4-Glucuronal Donor **11** to the Acceptor **31**.

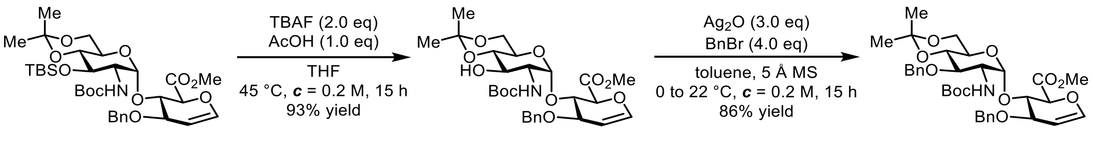

To a 100 mL flame-dried round-bottom flask equipped with a stir bar, compound **11** (1.72 g, 2.53 mmol, 1.0 equiv) and anhydrous THF (7.2 mL) were added. To the mixture was then added a premixed solution of TBAF (5 mL, 1.0 M in THF, 2.0 equiv) and AcOH (0.14 mL, 1.0 equiv). The reaction mixture was stirred for 15 h at 45 °C until the starting material **11** was fully consumed (monitored by TLC). The reaction mixture was then cooled down to room temperature and diluted with EtOAc (10 mL) and quenched with saturated aqueous $NaHCO_3$ solution (10 mL). The organic phase was separated from the aqueous one, and the aqueous phase was further extracted with EtOAc (15 mL × 3). The combined organic phase was washed

with brine (20 mL), dried over Na_2SO_4, and concentrated *in vacuo*. The residue was purified through a silica gel flash column (hexanes/EtOAc: from 100:1 to 3:2) to afford the desired product **S18** (1.33 g, 93% yield) as white foam.

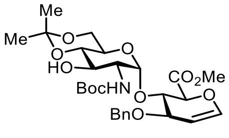

S18

Methyl 3-*O*-benzyl-4-*O*-(2-*tert*-butoxycarbonylamino-4,6-*O*-isopropylidene-2-deoxy-α-D-glucopyranosyl)-D-glucuronal (**S18**): $[\alpha]_D^{23}$ +46.9 (acetone, *c* = 0.5); IR ν_{max} (neat)/cm^{-1}: 3442 (w), 2976 (w), 1712(s), 1651 (m), 1508 (m), 1368 (m), 1170 (s), 1129 (s), 1049 (s), 1034 (s), 1016 (m); ^{1}H NMR (400 MHz, $CDCl_3$) 7.32 – 7.22 (m, 5H), 6.61 (d, *J* = 6.3 Hz, 1H), 5.04 (d, *J* = 3.5 Hz, 1H), 4.95 (t, *J* = 5.6 Hz, 1H), 4.80 (d, *J* = 9.3 Hz, 1H), 4.77 (dd, *J* = 2.9, 1.1 Hz, 1H), 4.44 (ABq, $\Delta\nu_{AB}$ = 39.6 Hz, *J* = 11.3 Hz, 2H), 4.34 (dd, *J* = 4.3, 2.7 Hz, 1H), 3.83 (dd, *J* = 10.2, 4.8 Hz, 1H), 3.79 – 3.72 (m, 2H), 3.69 (d, *J* = 10.2 Hz, 1H), 3.67 – 3.60 (m, 2H), 3.55 (d, *J* = 9.0 Hz, 1H), 3.52 (s, 3H), 2.80 (brs, 1H), 1.48 (s, 3H), 1.40 (s, 9H), 1.39 (s, 3H); ^{13}C NMR (100 MHz, $CDCl_3$) δ 167.9, 156.0, 145.4, 137.4, 128.3 (2C), 127.9 (2C), 127.7, 99.8, 98.1, 97.8, 80.1, 74.3, 73.8, 72.1, 70.0, 69.4, 66.8, 64.2, 62.0, 55.2, 52.1, 29.0, 28.2 (3C), 19.0; HRMS: m/z (ESI) calcd for $C_{28}H_{40}NO_{11}^+$, $[M + H]^+$, 566.2596, found 566.2581.

To a 50 mL flame-dried round-bottom flask equipped with a stir bar, the desilylated product **S18** from the previous step (1.33 g, 2.35 mmol, 1.0 equiv), Ag_2O (1.63 g, 7.05 mmol, 3.0 equiv), freshly activated 5 Å molecular sieves (powder, *ca.* 300 mg), and anhydrous toluene (11.8 mL) were added. The mixture was cooled to 0 °C before freshly distilled benzyl bromide (1.12 mL, 9.4 mmol, 4.0 equiv) was added dropwise. The reaction mixture was then stirred at room temperature for 15 h until the starting material **S18** was fully consumed (monitored by TLC). The reaction mixture was then filtered through a short pad of Celite®, further rinsed with acetone (10 mL), and concentrated *in vacuo*. The residue was purified through a silica gel flash column

(hexanes/EtOAc: from 100:1 to 4:1) to afford the desired product **30a** (1.33 g, 86% yield) as white foam.

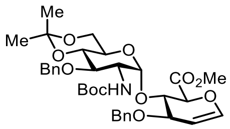

30a

Methyl 3-*O*-benzyl-4-*O*-(3-*O*-benzyl-2-*tert*-butoxycarbonylamino-4,6-*O*-isopropylidene-2-deoxy-α-D-glucopyranosyl)-D-glucuronal (**30a**): $[\alpha]_D^{23}$ +45.3 (acetone, *c* = 0.5); IR ν_{max} (neat)/cm^{-1}: 2976 (w), 1763 (m), 1716 (s), 1499 (m), 1367 (m), 1266 (s), 1128 (s), 1108 (s), 1037 (s); ^{1}H NMR (400 MHz, $CDCl_3$) δ 7.34 – 7.25 (m, 10H), 6.65 (d, *J* = 6.3 Hz, 1H), 5.05 (s, 1H), 4.98 (s, 1H), 4.85 (d, *J* = 11.9 Hz, 1H), 4.81 (s, 1H), 4.64 – 4.48 (m, 3H), 4.42 (d, *J* = 11.2 Hz, 1H), 4.37 (s, 1H), 3.94 – 3.84 (m, 2H), 3.78 – 3.68 (m, 4H), 3.55 (s, 3H), 3.49 (t, *J* = 9.4 Hz, 1H), 1.49 (s, 3H), 1.43 (s, 12H); ^{13}C NMR (100 MHz, $CDCl_3$) δ 168.0, 155.4, 145.4, 138.8, 137.6, 128.3 (2C), 128.1 (2C), 127.9 (2C), 127.8 (2C), 127.4, 127.3, 99.4, 98.8, 98.0, 79.7, 77.1, 75.1, 74.0, 73.8, 72.3, 69.5, 66.9, 64.5, 62.3, 53.9, 52.1, 29.2, 28.4 (3C), 19.1; HRMS: m/z (ESI) calcd for $C_{35}H_{46}NO_{11}^+$, $[M + H]^+$, 656.3065, found 656.3082.

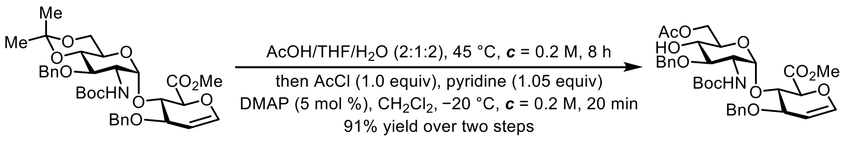

30a **31a**

To a 50 mL flame-dried round-bottom flask equipped with a stir bar, compound **30a** (490 mg, 0.75 mmol, 1.0 equiv) and a mixture of AcOH/THF/H_2O (2:1:2 vol/vol/vol, 3.8 mL) were added. The mixture was stirred at 45 °C for 8 h until the starting material **30a** was fully consumed (monitored by TLC). The reaction mixture was concentrated *in vacuo* and azeotropically dried

with toluene (5 mL × 4) to remove AcOH and H_2O. The crude hydrolysis product was obtained as white foam and directly used in the next step without further purification.

To a 50 mL flame-dried round-bottom flask equipped with a stir bar, the crude hydrolysis product from the last step, DMAP (4.6 mg, 0.0375 mmol, 5 mol %), and anhydrous CH_2Cl_2 (3 mL) were added. The mixture was cooled to –20 °C before anhydrous pyridine (63 μL, 0.79 mmol, 1.05 equiv) and acetyl chloride (1M in CH_2Cl_2, 0.75 mL, 0.75 mmol, 1.0 equiv) were added dropwise sequentially. The reaction mixture was stirred at –20 °C for 20 min until the starting material was fully consumed (monitored by TLC). The reaction mixture was then quenched with H_2O (2 mL) and the organic phase was separated from the aqueous one. The aqueous phase was further extracted with CH_2Cl_2 (3 mL × 2). The combined organic phase was washed with brine (5 mL) and dried over Na_2SO_4. After concentration *in vacuo*, the residue was purified through a silica gel flash column (hexanes/EtOAc: from 100:1 to 2:1) to afford the desired product **31a** (449 mg, 91% yield over two steps) as white foam.

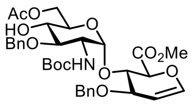

31a

Methyl 3-*O*-benzyl-4-*O*-(6-*O*-acetyl-3-*O*-benzyl-2-*tert*-butoxycarbonylamino-2-deoxy-α-D-glucopyranosyl)-D-glucuronal (**31a**): $[\alpha]_D^{23}$ +49.2 (acetone, *c* = 2.0); IR ν_{max} (neat)/cm^{-1}: 3447 (w), 2976 (w), 1736 (s), 1718 (s), 1500 (m), 1367 (m), 1247 (s), 1132 (m), 1064 (s), 1036 (s); ^{1}H NMR (400 MHz, $CDCl_3$) δ 7.48 – 7.19 (m, 10H), 6.63 (d, *J* = 6.3 Hz, 1H), 5.03 (d, *J* = 3.8 Hz, 1H), 5.02 – 4.95 (m, 1H), 4.92 (dd, *J* = 2.8, 1.4 Hz, 1H), 4.74 (s, 2H), 4.58 (d, *J* = 9.7 Hz, 1H), 4.47 (ABq, $\Delta\nu_{AB}$ = 40.9 Hz, *J* = 11.3 Hz, 2H), 4.39 – 3.35 (m, 2H), 4.29 (d, *J* = 12.1 Hz, 1H), 3.96 – 3.82 (m, 2H), 3.80 – 3.70 (m, 1H), 3.52 (s, 3H), 3.47 (q, *J* = 6.7 Hz, 2H), 2.89 (s, 1H), 2.07 (s, 3H), 1.42 (s, 9H); ^{13}C NMR (100 MHz, $CDCl_3$) δ 171.4, 167.9, 155.1, 145.4, 138.2, 137.4, 128.4 (2C), 128.3 (2C), 127.9 (2C), 127.73 (2C), 127.67, 127.6, 98.9, 97.9, 80.1, 79.8, 74.5, 73.4, 72.8, 70.8, 70.1, 69.4, 66.6, 63.1, 53.6, 52.0, 28.3 (3C), 20.7; HRMS: m/z (ESI) calcd for $C_{34}H_{44}NO_{12}^+$, $[M + H]^+$, 658.2858, found 658.2849.

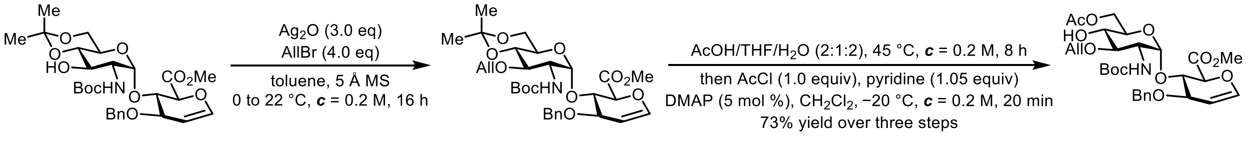

S18 **30b** **31b**

The synthesis of **31b** was carried out on a 2.5 mmol scale using the analogous procedures to obtain **31a**. The desired product **31b** was purified through a silica gel flash column (hexanes/EtOAc: from 100:1 to 2:1) as white foam (1.11 g, 73% yield over three steps).

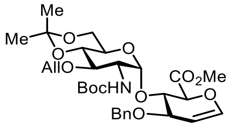

30b

Methyl 3-*O*-benzyl-4-*O*-(3-*O*-allyl-2-*tert*-butoxycarbonylamino-4,6-*O*-isopropylidene-2-deoxy-α-D-glucopyranosyl)-D-glucuronal (**30b**): $[\alpha]_D^{23}$ +38.9 (acetone, *c* = 1.0); IR ν_{max} (neat)/cm^{-1}: 3447 (w), 2978 (w), 1718 (s), 1500 (m), 1367 (m), 1202 (s), 1171 (s), 1129 (s), 1108 (s), 1073 (s), 1037 (s); ^{1}H NMR (400 MHz, $CDCl_3$) δ 7.39 – 7.27 (m, 5H), 6.66 (d, *J* = 6.3 Hz, 1H), 5.85 (ddt, *J* = 17.2, 10.6, 5.4 Hz, 1H), 5.25 (dd, *J* = 17.3, 1.8 Hz, 1H), 5.11 (dd, *J* = 10.5, 1.8 Hz, 1H), 5.05 (d, *J* = 3.9 Hz, 1H), 5.00 (t, *J* = 6.2 Hz, 1H), 4.81 (dd, *J* = 2.9, 1.3 Hz, 1H), 4.59 (d, *J* = 9.5 Hz, 1H), 4.47 (ABq, $\Delta\nu_{AB}$ = 40.1 Hz, *J* = 11.3 Hz, 2H), 4.39 – 4.36 (m, 1H), 4.32 (dd, *J* = 13.2, 5.0 Hz, 1H), 4.04 (dd, *J* = 13.2, 5.7 Hz, 1H), 3.89 – 3.80 (m, 2H), 3.80 – 3.71 (m, 2H), 3.73 – 3.62 (m, 2H), 3.55 (s, 3H), 3.39 (t, *J* = 9.2 Hz, 1H), 1.49 (s, 3H), 1.43 (s, 9H), 1.40 (s, 3H); ^{13}C NMR (100 MHz, $CDCl_3$) δ 168.0, 155.5, 145.4, 137.6, 135.1, 128.4 (2C), 128.0 (2C), 127.8, 116.2, 99.4, 98.8, 98.0, 79.7, 77.1, 75.0, 73.8, 73.0, 72.2, 69.5, 66.8, 64.6, 62.3, 53.9, 52.2, 29.1, 28.4 (3C), 19.1; HRMS: m/z (ESI) calcd for $C_{31}H_{44}NO_{11}^+$, $[M + H]^+$, 606.2909, found 606.2907.

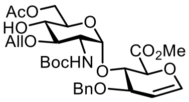

31b

Methyl 3-*O*-benzyl-4-*O*-(6-*O*-acetyl-3-*O*-allyl-2-*tert*-butoxycarbonylamino-2-deoxy-α-D-glucopyranosyl)-D-glucuronal (**31b**): $[\alpha]_D^{23}$ +45.5 (acetone, *c* = 2.0); IR ν_{max} (neat)/cm^{-1}: 3446 (w), 2977(w), 1717(s), 1505 (m), 1367 (m), 1246 (s), 1166 (m), 1108 (m), 1062 (s), 1035 (s), 1003 (m); ^{1}H NMR (400 MHz, $CDCl_3$) δ 7.37 – 7.26 (m, 5H), 6.64 (d, *J* = 6.3 Hz, 1H), 5.89 (ddt, *J* = 17.2, 10.4, 5.6 Hz, 1H), 5.27 (dd, *J* = 17.2, 1.6 Hz, 1H), 5.16 (dd, *J* = 10.4, 1.6 Hz, 1H), 5.04 (d, *J* = 3.8 Hz, 1H), 5.00 (t, *J* = 5.8 Hz, 1H), 4.93 (d, *J* = 1.3 Hz, 1H), 4.58 (d, *J* = 9.7 Hz, 1H), 4.52 (d, *J* = 11.3 Hz, 1H), 4.47 – 4.36 (m, 3H), 4.30 (d, *J* = 12.1 Hz, 1H), 4.27 – 4.14 (m, 2H), 3.91 – 3.80 (m, 2H), 3.80 – 3.71 (m, 1H), 3.53 (s, 3H), 3.46 (dt, *J* = 10.1, 4.5 Hz, 1H), 3.36 (t, *J* = 9.6 Hz, 1H), 2.82 (d, *J* = 3.4 Hz, 1H), 2.09 (s, 3H), 1.44 (s, 9H); ^{13}C NMR (100 MHz, $CDCl_3$) δ 171.5, 167.9, 155.2, 145.5, 137.5, 134.8, 128.4 (2C), 128.0 (2C), 127.8, 117.1, 99.0, 98.0, 80.3, 79.9, 73.5, 73.0, 70.9, 70.1, 69.5 (two peaks overlapped, 2C), 66.7, 63.1, 53.6, 52.1, 28.4 (3C), 20.7; HRMS: m/z (ESI) calcd for $C_{30}H_{42}NO_{12}^+$, $[M + H]^+$, 608.2702, found 608.2711.

F. Catalyst Discovery for the Iron-Catalyzed Stereospecific and Reiterative Glycosylation with Glycal Epoxides

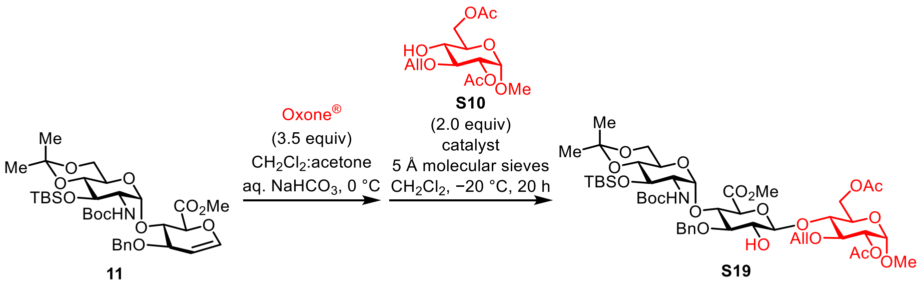

a. General Procedure for Glycal Epoxidation

To a 25 mL flask equipped with a stir bar at 0 °C, were added glycal **11** (81.5 mg, 0.12 mmol, 1.0 equiv) in CH_2Cl_2/acetone mixture (v/v: 5:1, 2.0 mL) and saturated aqueous $NaHCO_3$ solution (3.4 mL), followed by addition of Oxone® ($KHSO_5 \cdot 0.5KHSO_4 \cdot 0.5K_2SO_4$) (258.6 mg, 0.42 mmol, 3.5 equiv) in H_2O (2.2 mL) dropwise. After stirring vigorously at 0 °C for 2 h, the reaction mixture was extracted with CH_2Cl_2 (3 mL × 3). The combined organic phase was dried over anhydrous Na_2SO_4 and concentrated *in vacuo*. The residue was further azeotropically dried with anhydrous toluene (1.0 mL × 3). The obtained glycal epoxide was assayed by ^1H NMR to get the diastereomeric ratio (*dr* > 20:1) and directly used in the next step.

Stereochemistry Determination of Glycal Epoxide S20

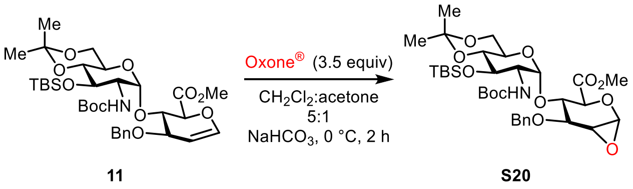

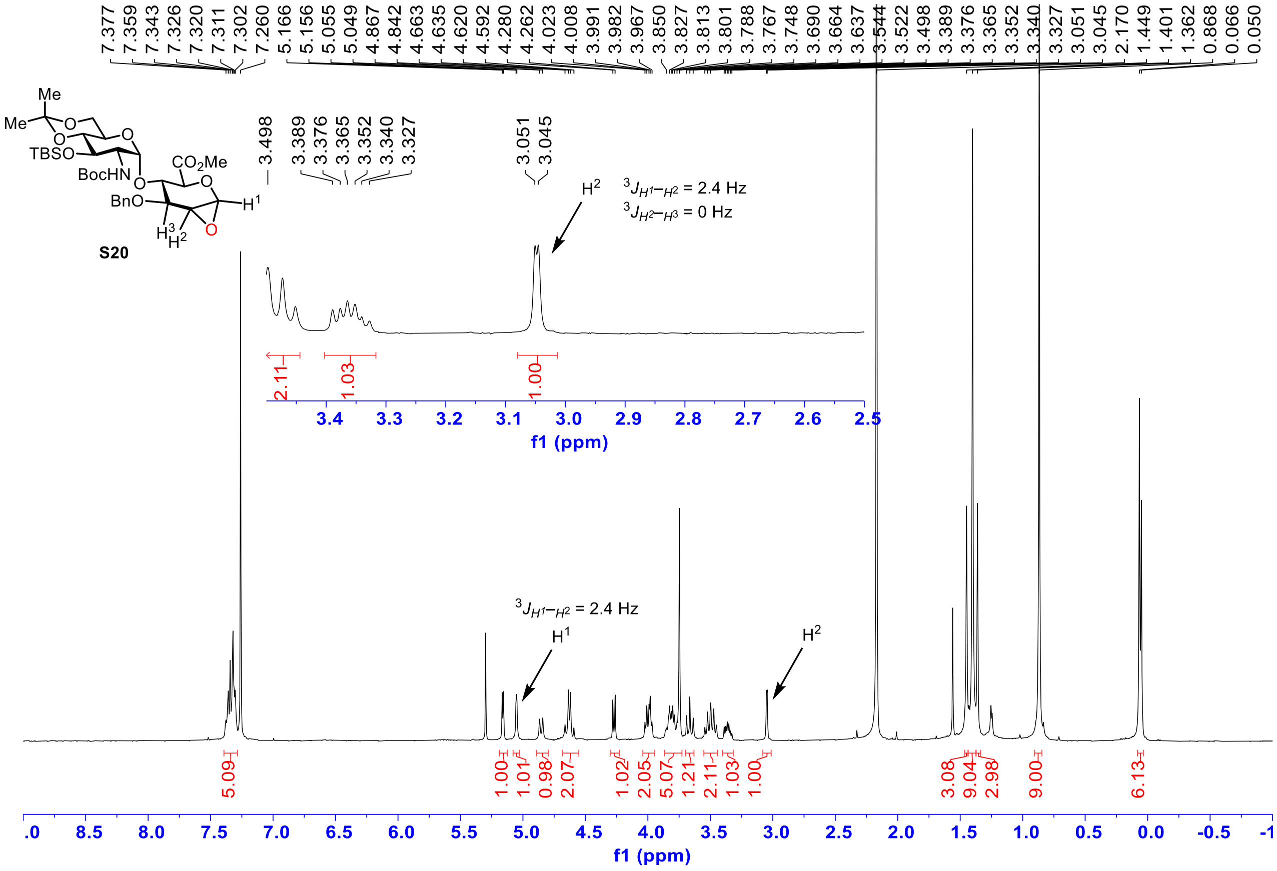

Figure S12. Stereochemistry Determination of Glycal Epoxide **S20**.

The stereochemistry of the glycal epoxide **S20** was determined by ^{1}H NMR analysis. It is known that the C(2)–H chemical shift of a glucal α-epoxide is around 3.0 ppm, whereas that of a glucal β-epoxide is around 3.3 ppm.[10]

Additionally, the $^{3}J_{H1-H2}$ value for a typical glucal α-epoxide is around 2.4 Hz, reflecting the dihedral angle ($\Phi_{1,2}$) that approaches 0°, whereas the $^{3}J_{H2-H3}$ value is < 0.5 Hz because the dihedral angle ($\Phi_{2,3}$) approaches 90°, characteristic of a *trans*-relationship between H^2 and H^3 that is pseudo-axial.[10] As a result, the H^2 signal of a glucal α-epoxide appears as a doublet.

In contrast, while a glucal β-epoxide retains a similar $\Phi_{1,2}$ and thus a comparable ${}^3J_{H1-H2}$, its $\Phi_{2,3}$ is around 45°, leading to a ${}^3J_{H2-H3}$ around 1.7 Hz.[10] This causes the H^2 signal to appear as a doublet of doublets (though often observed as a triplet due to the broadening line width).

Based on these data, we have assigned glycal epoxide **S20** as an α-epoxide (*dr* > 20:1) with ${}^3J_{H1-H2}$ = 2.4 Hz and ${}^3J_{H2-H3}$ = 0 Hz. This assignment was further corroborated by the stereochemistry analysis of the glycosylation product 1,2-*trans*-β-glycoside **S19**.

The iron porphyrin catalyst used for the iron-catalyzed stereospecific glycosylation with glycal epoxides is prepared according to the following procedures:

b. Synthesis of the Hemin Dimethyl Ester S23

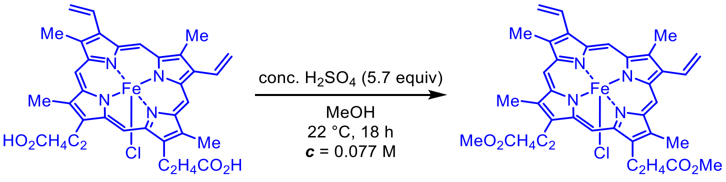

S22 **S23**

To a 100 mL oven-dried round-bottom flask equipped with a magnetic stirring bar were added hemin (**S22**) (1.5 g, 2.3 mmol, 1 equiv), MeOH (30 mL), and concentrated H_2SO_4 (0.7 mL, 13 mmol, 5.7 equiv). The reaction was stirred for 18 hours at room temperature. H_2O (30 mL) was added to the reaction mixture, followed by extraction with CH_2Cl_2 (30 mL × 4). The combined organic layer was washed with water (30 mL × 2) and brine (30 mL × 1), dried over Na_2SO_4, and concentrated *in vacuo*. The residue was further dried azeotropically with anhydrous toluene (10 mL x 2) and subsequently dried *in vacuo* to afford hemin dimethyl ester (**S23**) as dark purple powder (1.49 g, 95% yield).

Table S2. Catalyst Discovery for the Iron-Catalyzed Stereospecific Glycosylation of a Model Substrate **S10** with Glycal Epoxide **S20**

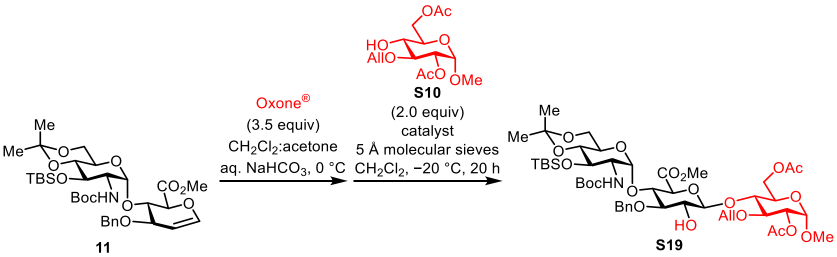

entry[a]	catalyst/promotor	conversion (%)[a]	yield of **S19** (%)[b]	*dr*[b]
1	methanesulfonic acid (10 mol %)	>95	<5	NA
2	camphorsulfonic acid (10 mol %)	>95	<5	NA
3	**S21a** (10 mol %)	<10	<5	NA
4	$(PhO)_2POOH$ (10 mol %)	<10	<5	NA
5	**S21b** (10 mol %)	<10	<5	NA
6	**S21c** (10 mol %)	<10	<5	NA
7	**S21d** (10 mol %)	<10	<5	NA
8	$ZnCl_2$ (1.0 equiv)[c]	72	34	4.3:1
9	$Zn(OTf)_2$ (20 mol %)	40	<5	NA
10	TMSOTf (10 mol %)	>95	12	4.8:1
11	$Fe(OTf)_2$–**L1** (10 mol %)[d]	69	47	5.6:1
12	$Fe(OTf)_2$–**L2** (10 mol %)[d]	56	37	2.2:1
13	**S21e** (7 mol %)[e]	81	15	5.0:1
14	**S21f** (7 mol %)[e]	>95	29	4.8:1
15	**S21g** (7 mol %)[e]	>95	56	>20:1
16	**S21h** (7 mol %)[e]	>95	77	>20:1
17	**S21i** (7 mol %)[e] or **S21j** (7 mol%)[e]	<5	NA	NA
18	**32** (7 mol %)[e]	>95	71	>20:1

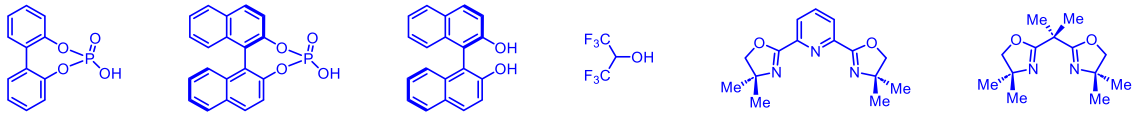

S21a **S21b** **S21c** **S21d** **L1** **L2**

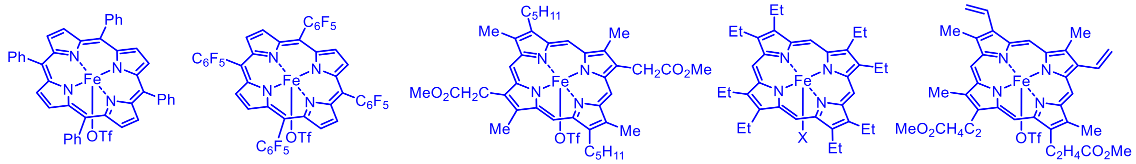

S21e **S21f** **S21g** **S21h**: X = OTf **32**
S21i: X = $MeSO_3$
S21j: X = 4-dodecylPhSO_3

[a]Epoxidation was carried out in a biphasic reaction medium with Oxone® and acetone. The glycal epoxide was dried azeotropically with toluene, assayed by ^{1}H NMR, and then directly used. The glycosylation was carried out at −20 °C in CH_2Cl_2. The reaction was quenched by methanol and imidazole for conversion measurement. [b]Isolated yield; *dr* was determined by ^{1}H NMR analysis. [c]0 °C in THF. [d]CH_2Cl_2/MeCN (10:1) as the solvent. [e]Iron(III) porphyrin triflate/sulfonate catalysts were formed *in situ* from the corresponding iron porphyrin chloride and AgOTf or silver sulfonates.

Procedure for Preparation of Iron(III) Porphyrin Triflate Catalyst 32

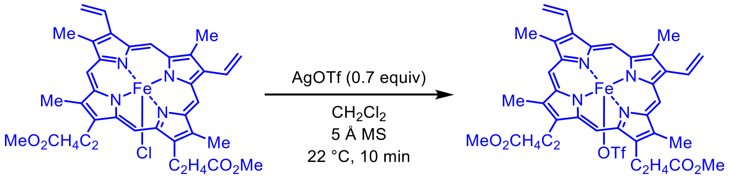

S23 **32**

To a flame-dried sealable 2-dram vial equipped with a stir bar were added hemin dimethyl ester (**S23**) (34.0 mg, 0.05 mmol, 10 mol %), AgOTf (9.0 mg, 0.035 mmol, 7 mol %), and freshly activated 5 Å molecular sieves, powder (*ca.* 30 mg). After the vial was evacuated and backfilled with N_2 three times, anhydrous CH_2Cl_2 (0.6 mL) was added and the solution was stirred at room temperature for 10 min. The resulting iron(III) porphyrin triflate catalyst **32** solution was directly used.

G. Assembly of Four Heparan Sulfate Tetrasaccharide Modules via the Iron-Catalyzed Stereospecific Glycosylation with Glycal Epoxides

a. Heparan Sulfate GlcN(α1-4)GlcA(β1-4)GlcN(α1-4)GlcA Module Synthesis

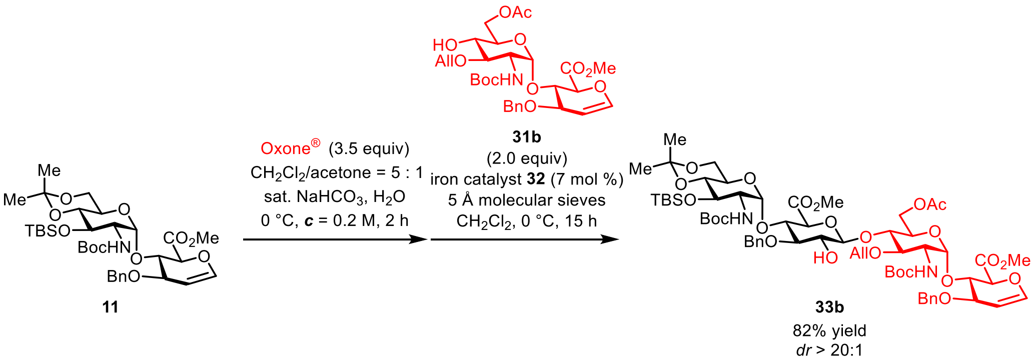

To a 100 mL flask equipped with a stir bar at 0 °C, were added glycal **11** (340 mg, 0.5 mmol, 1.0 equiv) in CH_2Cl_2/acetone mixture (v/v: 5:1, 8.3 mL) and saturated aqueous $NaHCO_3$ solution (14.2 mL), followed by addition of Oxone® ($KHSO_5 \cdot 0.5KHSO_4 \cdot 0.5K_2SO_4$) (1.08 g, 1.75 mmol, 3.5 equiv) in H_2O (9.2 mL) dropwise. After stirring vigorously at 0 °C for 2 h, the reaction mixture was extracted with CH_2Cl_2 (10 mL × 3). The combined organic phase was dried over anhydrous Na_2SO_4 and concentrated *in vacuo*. The residue was further azeotropically dried with anhydrous toluene (5 mL × 3). The obtained glycal epoxide was assayed by ^{1}H NMR to get the diastereomeric ratio (*dr* > 20:1) and directly used in the next step.

To a flame-dried sealable 2-dram vial equipped with a stir bar were added glycosyl acceptor **31b** (658 mg, 1.0 mmol, 2.0 equiv) and freshly activated 5 Å molecular sieves, powder (*ca.* 500 mg). After the vial was evacuated and backfilled with N_2, anhydrous CH_2Cl_2 (0.7 mL) was added. The iron(III) porphyrin catalyst **32** (0.035 mmol, 7 mol %) solution was added to the vial and the resulting mixture was stirred at −78 °C for 5 min before a solution of the aforementioned glycal epoxide in anhydrous CH_2Cl_2 (1.0 mL) was then added to the mixture at −78 °C dropwise. The reaction mixture was kept at 0 °C for 15 h and then quenched with imidazole (7 mg) in CH_2Cl_2 (0.1 mL) at the same temperature. The mixture was filtered through a pad of Celite® silica gel and eluted with CH_2Cl_2 (10 mL). The organic layer was then concentrated *in vacuo* and the *dr* (>20:1) was determined based on the ^{1}H NMR analysis of the crude reaction mixture. The residue was purified through a silica gel flash column (hexanes/EtOAc: from 100:1 to 4:1) to

afford the corresponding glycosylation product **33b** as white foam (534 mg, 82% yield, *dr* >20:1).

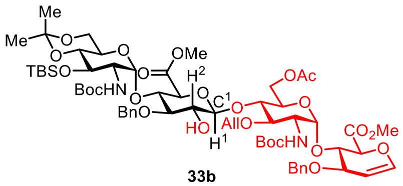

33b

Methyl 3-*O*-benzyl-4-*O*-[(2-*tert*-butoxycarbonylamino-3-*O*-*tert*-butyldimethylsilyl-4,6-*O*-isopropylidene-2-deoxy-α-D-glucopyranosyl)-(1→4)-(methyl 3-*O*-benzyl-β-D-glucopyranosyluronate)-(1→4)-(6-*O*-acetyl-3-*O*-allyl-2-*tert*-butoxycarbonylamino-2-deoxy-α-D-glucopyranosyl)]-D-glucuronal (**33b**): $[\alpha]_D^{23}$ +30.3 (acetone, *c* = 1.0); IR ν_{max} (neat)/cm^{-1}: 3456 (w), 2952 (s), 2931 (m), 2362 (m), 1752 (s), 1722 (m), 1652 (w); ^{1}H NMR (800 MHz, $CDCl_3$) δ 7.38 – 7.26 (m, 10H), 6.60 (d, *J* = 6.3 Hz, 1H), 5.81 (ddt, *J* = 17.2, 10.9, 5.6 Hz, 1H), 5.35 (d, *J* = 3.9 Hz, 1H), 5.21 (dd, *J* = 17.2, 1.7 Hz, 1H), 5.09 (dd, *J* = 10.9, 1.7 Hz, 1H), 5.02 – 4.96 (m, 2H), 4.92 (s, 1H), 4.92 – 4.86 (m, 2H), 4.70 (d, *J* = 10.4 Hz, 1H), 4.62 (d, *J* = 12.0 Hz, 1H), 4.54 – 4.48 (m, 2H), 4.42 (d, *J* = 7.9 Hz, 1H), 4.41 (d, *J* = 12.0 Hz, 1H), 4.35 – 4.32 (s, 1H), 4.31 (dd, *J* = 12.6, 5.6 Hz, 1H), 4.12 (dd, *J* = 12.3, 5.9 Hz, 1H), 4.03 – 3.96 (m, 2H), 3.89 (dd, *J* = 10.4, 6.0 Hz, 1H), 3.87 – 3.83 (m, 2H), 3.82 – 3.79 (m, 1H), 3.77 (s, 3H), 3.76 – 3.73 (m, 2H), 3.61 (t, *J* = 10.6 Hz, 1H), 3.59 – 3.53 (m, 3H), 3.51 (s, 3H), 3.49 (t, *J* = 9.6 Hz, 1H), 3.44 (t, *J* = 9.3 Hz, 1H), 3.40 (t, *J* = 9.6 Hz, 1H), 3.23 (brs, 1H), 3.21 – 3.16 (m, 1H), 2.07 (s, 3H), 1.42 (s, 9H), 1.42 (s, 3H), 1.33 (s, 3H), 1.32 (s, 9H), 0.86 (s, 9H), 0.06 (s, 3H), 0.04 (s, 3H); ^{13}C NMR (100 MHz, $CDCl_3$) δ 171.3, 168.3, 167.9, 155.1, 155.0, 145.4, 137.6, 137.5, 134.9, 128.5 (2C), 128.3 (two peaks overlapped, 4C), 128.0 (two peaks overlapped, 3C), 127.8, 116.6, 103.8, 99.2, 99.0, 98.8, 98.0, 84.0, 79.9, 79.6, 78.8, 78.6, 77.2, 75.0, 74.7, 74.4, 73.94, 73.86, 73.5, 73.3, 71.3, 70.3, 69.4, 66.9, 64.1, 63.1, 61.8, 55.2, 53.9, 52.6, 52.0, 29.1, 28.4 (3C), 28.3 (3C), 25.7 (3C), 20.9, 18.9, 18.2, -4.1, -5.1; HRMS: m/z (ESI) calcd for $C_{64}H_{95}N_2O_{24}Si^+$, $[M + H]^+$, 1303.6039, found 1303.6081. $^1J_{^{13}C1–H1}$ = 174.5 Hz, 171.7 Hz, 160.7 Hz. $^3J_{H1–H2}$ = 7.9 Hz.

Stereochemistry Determination of Glycosylation Product 33b

The stereochemistry of newly formed anomeric center (C1) of **33b** was determined by measuring $^{1}J^{13}_{C1–H1}$ (160.7 Hz) through un-decoupled HSQC experiments.[11-12] The C2 stereochemistry was determined by measuring $^{3}J_{H1–H2}$ (7.9 Hz).

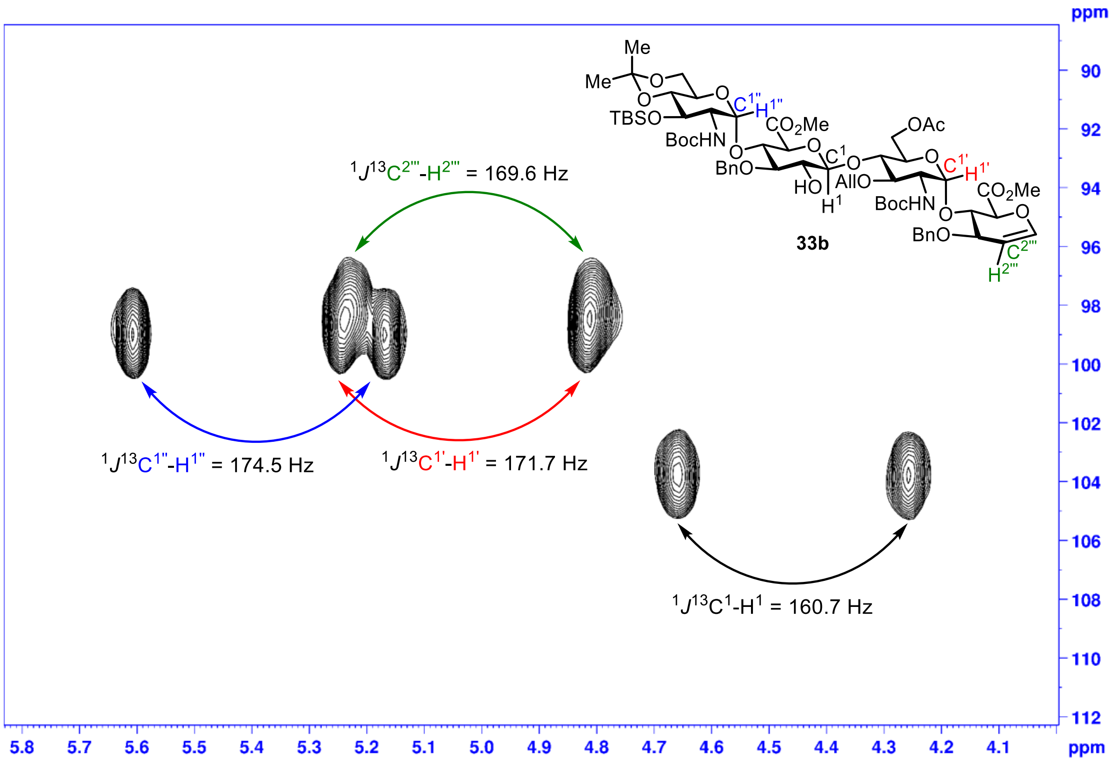

Figure S13. Un-decoupled HSQC Analysis to Determine Stereochemistry of **33b**.

A $^{1}J^{13}_{C1–H1}$ value of 160.7 Hz suggested that the newly formed glycosidic bond is in equatorial position. The $^{3}J_{H1–H2}$ value of 7.9 Hz suggested that the H2 is in axial position and corroborated the exclusive formation of the α-epoxide in glycal epoxidation.

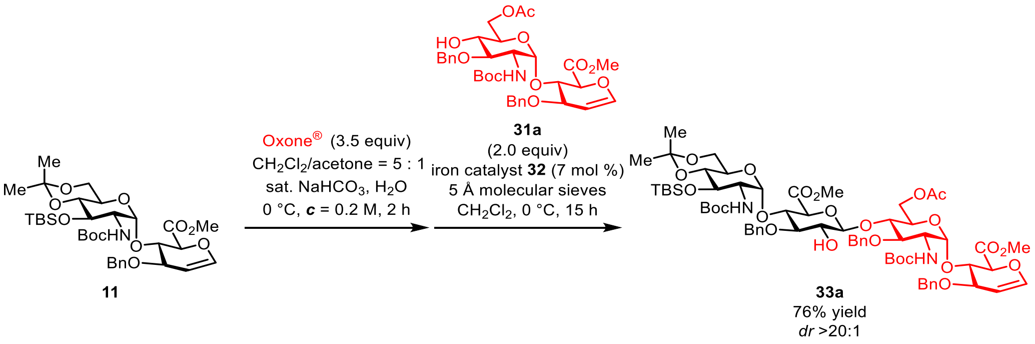

The epoxidation of **11** and the subsequent iron-catalyzed stereospecific glycosylation with glycosyl acceptor **31a** were carried out on a 0.5 mmol scale using procedures analogous to those described above. The desired glycosylation product **33a** was obtained through a silica gel flash column (hexanes/EtOAc: from 100:1 to 4:1) as white foam (514 mg, 76% yield). The stereochemistry of **33a** was determined by measuring $^3J_{H1-H2}$ (9.3 Hz) and $^1J^{13}{}_{C1-H1}$ (161.2 Hz).

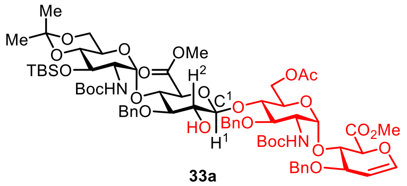

33a

Methyl 3-*O*-benzyl-4-*O*-[(2-*tert*-butoxycarbonylamino-3-*O*-*tert*-butyldimethylsilyl-4,6-*O*-isopropylidene-2-deoxy-α-D-glucopyranosyl)-(1→4)-(methyl 3-*O*-benzyl-β-D-glucopyranosyluronate)-(1→4)-(6-*O*-acetyl-3-*O*-benzyl-2-*tert*-butoxycarbonylamino-2-deoxy-α-D-glucopyranosyl)]-D-glucuronal (**33a**): $[\alpha]_D^{23}$ +42.4 (acetone, *c* = 2.0); IR ν_{max} (neat)/cm^{-1}: 3457 (w), 2953 (m), 2929 (w), 1722 (m), 1652 (w), 1501 (m), 1367 (m); ^{1}H NMR (800 MHz, C_6D_6) δ 7.55 (d, *J* = 7.5 Hz, 2H), 7.49 (d, *J* = 7.5 Hz, 2H), 7.29 (t, *J* = 7.5 Hz, 2H), 7.27 – 7.22 (m, 4H), 7.18 (t, *J* = 7.5 Hz, 2H), 7.14 – 7.08 (m, 3H), 6.50 (d, *J* = 6.3 Hz, 1H), 5.56 (d, *J* = 3.9 Hz, 1H), 5.15 (d, *J* = 10.0 Hz, 1H), 5.11 – 5.07 (m, 2H), 5.05 (d, *J* = 11.4 Hz, 1H), 5.00 (d, *J* = 3.8 Hz, 1H), 4.91 (dd, *J* = 11.8, 1.7 Hz, 1H), 4.88 (d, *J* = 10.4 Hz, 1H), 4.79 – 4.74 (m, 1H), 4.59 (d, *J* = 9.3 Hz, 1H), 4.41 – 4.36 (m, 2H), 4.32 – 4.22 (m, 3H), 4.21 – 4.16 (m, 2H), 4.15 – 4.09 (m, 2H), 4.02 (t, *J* = 9.7 Hz, 1H), 3.96 – 3.89 (m, 1H), 3.76 (t, *J* = 9.2 Hz, 1H), 3.71 (d, *J* = 9.7 Hz, 1H), 3.64 – 3.58 (m, 2H), 3.55 – 3.45 (m, 4H), 3.44 (s, 3H), 3.39 (t, *J* = 9.0 Hz, 1H), 3.34 – 3.29 (m, 1H), 3.29 – 3.25 (m, 1H), 3.21 (s, 3H), 1.82 (s, 3H), 1.42 (s, 9H), 1.40 (s, 3H), 1.39 (s, 9H), 1.20 (s, 3H), 1.10 (s, 9H), 0.25 (s, 3H), 0.22 (s, 3H); ^{13}C NMR (100 MHz,

$CDCl_3$) δ 171.3, 168.0, 167.8, 155.1, 155.0, 145.3, 138.3, 137.5, 137.4, 128.5 (2C), 128.3 (two peaks overlapped, 4C), 128.1 (2C), 127.9 (two peaks overlapped, 4C), 127.8, 127.6, 127.3, 103.6, 99.1, 99.0, 98.7, 98.0, 83.8, 79.8, 79.5, 78.4, 78.2, 74.9, 74.6 (two peaks overlapped, 2C), 74.3, 73.9, 73.4, 73.2, 71.3 (two peaks overlapped, 2C), 70.3, 69.4, 66.8, 64.1, 63.0, 61.7, 55.2, 53.8, 52.5, 52.0, 29.0, 28.3 (3C), 28.2 (3C), 25.7 (3C), 20.8, 18.8, 18.2, -4.2, -5.2; HRMS: m/z (ESI) calcd for $C_{68}H_{97}N_2O_{24}Si^+$, $[M + H]^+$, 1353.6195, found 1353.6157. $^1J_{^{13}C1-H1}$ = 175.6 Hz, 177.2 Hz, 161.2 Hz. $^3J_{H1-H2}$ = 9.3 Hz.

b. Heparan Sulfate GlcN(α1-4)GlcA(β1-4)GlcN(α1-4)IdoA Module Synthesis

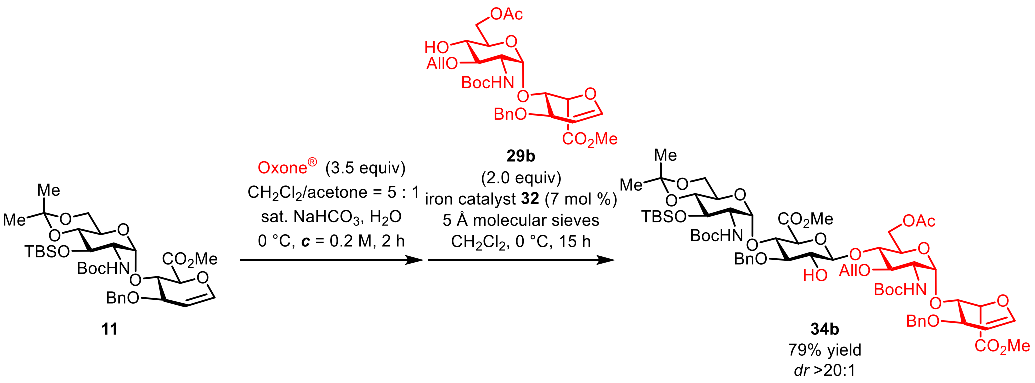

To a 100 mL flask equipped with a stir bar at 0 °C, were added glycal **11** (340 mg, 0.5 mmol, 1.0 equiv) in CH_2Cl_2/acetone mixture (v/v: 5:1, 8.3 mL) and saturated aqueous $NaHCO_3$ solution (14.2 mL), followed by addition of Oxone® ($KHSO_5 \cdot 0.5KHSO_4 \cdot 0.5K_2SO_4$) (1.08 g, 1.75 mmol, 3.5 equiv) in H_2O (9.2 mL) dropwise. After stirring vigorously at 0 °C for 2 h, the reaction mixture was extracted with CH_2Cl_2 (10 mL × 3). The combined organic phase was dried over anhydrous Na_2SO_4 and concentrated *in vacuo*. The residue was further azeotropically dried with anhydrous toluene (5 mL × 3). The obtained glycal epoxide was assayed by ^{1}H NMR to get the diastereomeric ratio (*dr* > 20:1) and directly used in the next step.

To a flame-dried sealable 2-dram vial equipped with a stir bar were added glycosyl acceptor **29b** (658 mg, 1.0 mmol, 2.0 equiv) and freshly activated 5 Å molecular sieves, powder (*ca.* 500 mg). After the vial was evacuated and backfilled with N_2, anhydrous CH_2Cl_2 (0.7 mL) was added. The iron porphyrin triflate catalyst **32** (0.035 mmol, 7 mol %) solution was added to the vial and the resulting mixture was stirred at −78 °C for 5 min before a solution of the aforementioned

glycal epoxide in anhydrous CH_2Cl_2 (1.0 mL) was then added to the mixture at −78 °C dropwise. The reaction mixture was kept at 0 °C for 15 h and then quenched with a solution of imidazole (7 mg) in CH_2Cl_2 (0.1 mL) at the same temperature. The mixture was filtered through a pad of Celite® silica gel and eluted with CH_2Cl_2 (10 mL). The organic layer was then concentrated *in vacuo*, and the residue was purified through a silica gel flash column (hexanes/EtOAc: from 100:1 to 2:1) to afford the corresponding glycosylation product **34b** as white foam (515 mg, 79% yield).

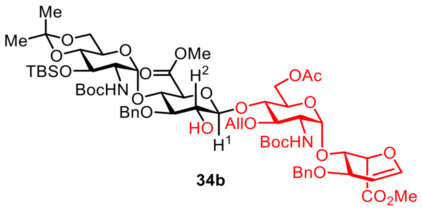

34b

Methyl 3-*O*-benzyl-4-*O*-[(2-*tert*-butoxycarbonylamino-3-*O*-*tert*-butyldimethylsilyl-4,6-*O*-isopropylidene-2-deoxy-α-D-glucopyranosyl)-(1→4)-(methyl 3-*O*-benzyl-β-D-glucopyranosyluronate)-(1→4)-(6-*O*-acetyl-3-*O*-allyl-2-*tert*-butoxycarbonylamino-2-deoxy-α-D-glucopyranosyl)]-L-guluronal (**34b**): $[\alpha]_D^{23}$ +3.5 (acetone, *c* = 1.0); IR ν_{max} (neat)/cm^{-1}: 3452 (w), 2976 (s), 2929 (m), 2174 (m), 1751 (s), 1719 (m), 1647 (w); ^{1}H NMR (800 MHz, $CDCl_3$) δ 7.37 – 7.26 (m, 10H), 6.65 (d, *J* = 6.1 Hz, 1H), 5.81 (ddt, *J* = 17.2, 10.5, 5.2 Hz, 1H), 5.36 (d, *J* = 3.9 Hz, 1H), 5.21 (dd, *J* = 17.2, 1.7 Hz, 1H), 5.10 (dd, *J* = 10.5, 1.7 Hz, 1H), 4.99 (t, *J* = 5.8 Hz, 1H), 4.90 – 4.84 (m, 2H), 4.83 (d, *J* = 3.8 Hz, 1H), 4.71 (d, *J* = 10.3 Hz, 1H), 4.63 (d, *J* = 11.8 Hz, 1H), 4.61 – 4.57 (m, 2H), 4.54 (d, *J* = 12.1 Hz, 1H), 4.51 (d, *J* = 9.8 Hz, 1H), 4.43 (d, *J* = 7.6 Hz, 1H), 4.30 (s, 1H), 4.26 (dd, *J* = 12.6, 5.2 Hz, 1H), 4.22 (dd, *J* = 12.3, 3.7 Hz, 1H), 4.02 – 3.96 (m, 2H), 3.88 (d, *J* = 9.9 Hz, 1H), 3.87 – 3.83 (m, 1H), 3.80 (s, 3H), 3.78 (s, 3H), 3.77 – 3.74 (m, 2H), 3.73 (d, *J* = 5.3 Hz, 1H), 3.65 – 3.59 (m, 3H), 3.58 (t, *J* = 9.1 Hz, 1H), 3.54 (t, *J* = 8.4 Hz, 1H), 3.50 (t, *J* = 9.3 Hz, 1H), 3.45 (t, *J* = 9.2 Hz, 1H), 3.37 (t, *J* = 9.2 Hz, 1H), 3.25 (brs, 1H), 3.22 – 3.16 (m, 1H), 2.12 (s, 3H), 1.43 (s, 3H), 1.39 (s, 9H), 1.34 (s, 3H), 1.33 (s, 9H), 0.86 (s, 9H), 0.06 (s, 3H), 0.04 (s, 3H); ^{13}C NMR (100 MHz, $CDCl_3$) δ 171.3, 168.7, 168.3, 155.2, 155.0, 146.0, 137.6, 137.5, 134.8, 128.5 (two peaks overlapped, 4C), 128.4 (2C), 128.0 (2C), 127.7 (two peaks overlapped, 2C), 116.7, 103.4, 99.2, 99.0, 98.5, 95.8, 84.0, 79.7, 79.6, 78.2, 77.6, 75.0, 74.6, 74.6, 74.4, 73.9, 73.5, 72.4, 71.3, 71.1, 70.14, 70.06, 65.7, 64.1, 62.7, 61.8,

55.2, 53.5, 52.7, 52.6, 29.0, 28.4 (3C), 28.3 (3C), 25.7 (3C), 21.0, 18.8, 18.2, -4.1, -5.1; HRMS: m/z (ESI) calcd for $C_{64}H_{95}N_2O_{24}Si^+$, $[M + H]^+$, 1303.6039, found 1303.6078. $^1J_{^{13}C1-H1}$ = 176.9 Hz, 171.3 Hz, 159.7 Hz. $^3J_{H1-H2}$ = 7.6 Hz.

The stereochemistry of newly formed anomeric center (C1) of **34b** was determined by measuring $^1J_{^{13}C1-H1}$ (159.7 Hz) through un-decoupled HSQC experiments.[11-12] The C2 stereochemistry was determined by measuring $^3J_{H1-H2}$ (7.6 Hz).

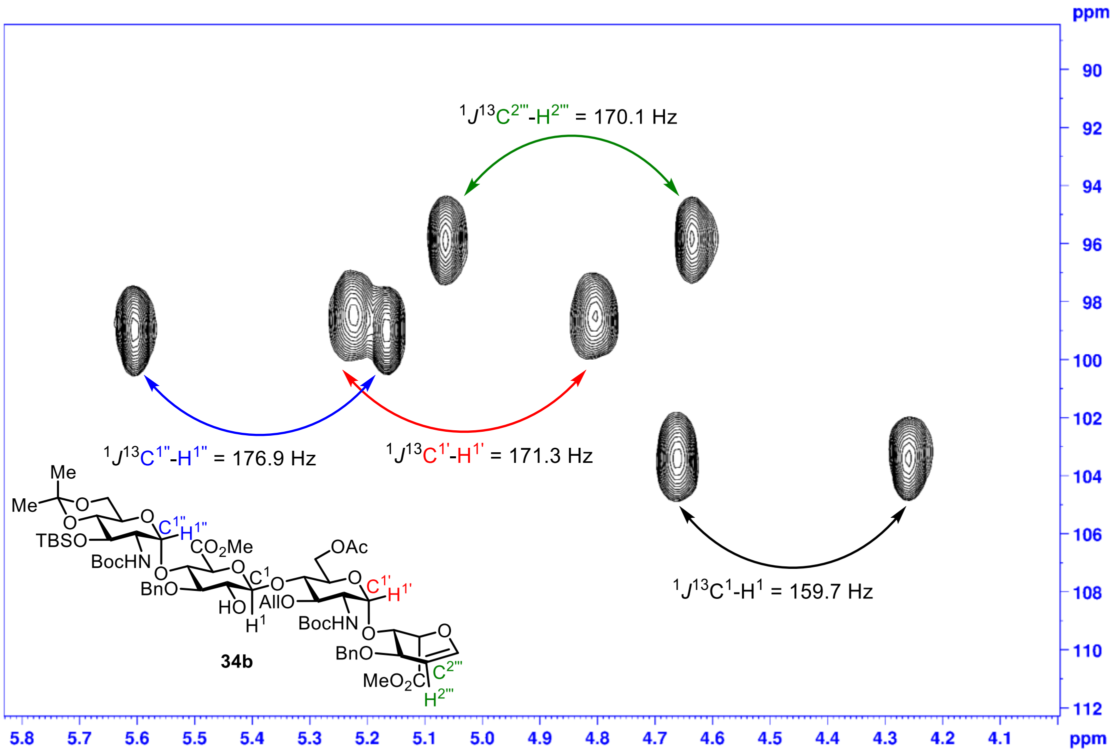

Figure S14. Un-decoupled HSQC Analysis to Determine Stereochemistry of **34b**.

A $^1J_{^{13}C1-H1}$ value of 159.7 Hz suggested that the newly formed glycosidic bond is in equatorial position. The $^3J_{H1-H2}$ value of 7.6 Hz suggested that the H2 is in axial position and corroborated the exclusive formation of the α-epoxide in glycal epoxidation.

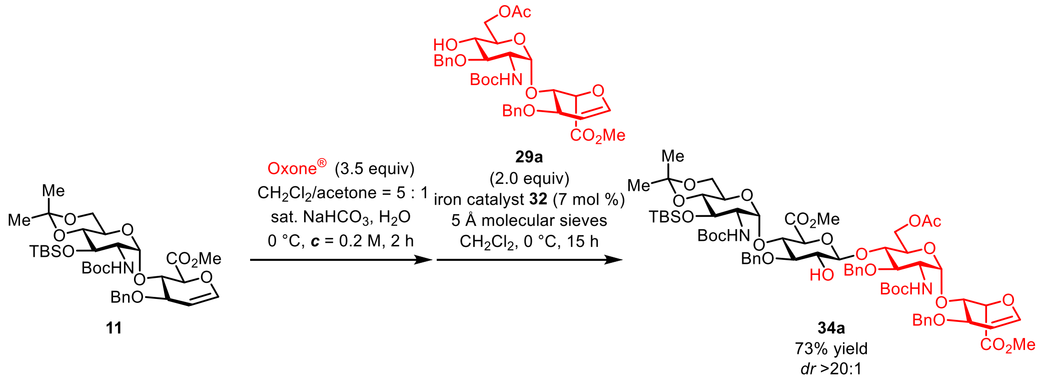

The epoxidation of **11** and the subsequent iron-catalyzed stereospecific glycosylation with glycosyl acceptor **29a** were carried out on a 0.5 mmol scale using procedures analogous to those described above. The desired glycosylation product **34a** was obtained through a silica gel flash column (hexanes/EtOAc: from 100:1 to 4:1) as white foam (494 mg, 73% yield). The stereochemistry of **34a** was determined by measuring $^{3}J_{H1–H2}$ (8.3 Hz) and $^{1}J_{^{13}C1–H1}$ (161.1 Hz).

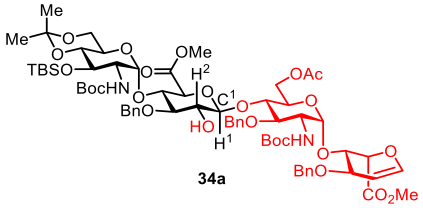

34a

Methyl 3-*O*-benzyl-4-*O*-[(2-*tert*-butoxycarbonylamino-3-*O*-*tert*-butyldimethylsilyl-4,6-*O*-isopropylidene-2-deoxy-α-D-glucopyranosyl)-(1→4)-(methyl 3-*O*-benzyl-β-D-glucopyranosyluronate)-(1→4)-(6-*O*-acetyl-3-*O*-benzyl-2-*tert*-butoxycarbonylamino-2-deoxy-α-D-glucopyranosyl)]-L-guluronal (**34a**): $[\alpha]_D^{23}$ +8.6 (acetone, *c* = 0.5); IR ν_{max} (neat)/cm^{-1}: 3451 (w), 2970 (w), 2361 (m), 2177 (w), 1740 (s), 1724 (s), 1367 (s); ^{1}H NMR (800 MHz, C_6D_6) δ 7.53 (d, *J* = 7.5 Hz, 2H), 7.44 (d, *J* = 7.5 Hz, 2H), 7.28 (t, *J* = 7.5 Hz, 2H), 7.23 (t, *J* = 7.5 Hz, 2H), 7.21 (d, *J* = 7.5 Hz, 2H), 7.19 – 7.15 (m, 2H), 7.14 – 7.08 (m, 3H), 6.40 (d, *J* = 6.1 Hz, 1H), 5.58 (d, *J* = 3.8 Hz, 1H), 5.11 (d, *J* = 10.1 Hz, 1H), 5.08 (d, *J* = 10.3 Hz, 1H), 5.00 – 4.90 (m, 3H), 4.86 (d, *J* = 10.3 Hz, 1H), 4.80 – 4.76 (m, 1H), 4.62 (d, *J* = 9.2 Hz, 1H), 4.59 (s, 1H), 4.45 – 4.39 (m, 2H), 4.35 (d, *J* = 11.5 Hz, 2H), 4.31 (d, *J* = 11.6 Hz, 1H), 4.27 – 4.20 (m, 1H), 4.21 – 4.16 (m, 3H), 4.14 (t, *J* = 9.3 Hz, 1H), 3.97 – 3.89 (m, 2H), 3.77 (d, *J* = 10.0 Hz, 1H), 3.73 (t, *J* = 9.3 Hz, 1H), 3.67 – 3.60 (m, 3H), 3.57 – 3.52 (m, 1H), 3.51 (s, 3H), 3.47 – 3.38 (m,

6H), 3.33 – 3.28 (m, 1H), 1.82 (s, 3H), 1.41 (s, 9H), 1.38 (s, 3H), 1.34 (s, 9H), 1.19 (s, 3H), 1.09 (s, 9H), 0.24 (s, 3H), 0.21 (s, 3H); ^{13}C NMR (100 MHz, C_6D_6) δ 171.2, 168.8, 168.4, 155.4 (two peaks overlapped, 2C), 146.2, 139.3, 138.6, 138.5, 129.0 (2C), 128.8 (2C), 128.7 (2C), 128.5 (2C), 128.2 (2C), 128.0 (2C), 127.93, 127.89, 127.5, 104.1, 99.8, 99.4, 99.0, 96.6, 83.8, 79.34, 79.31, 78.5, 78.4, 75.24, 75.18, 75.1, 74.9, 74.8, 74.5, 72.9, 72.2, 72.1, 71.1, 70.2, 66.7, 64.7, 63.4, 62.1, 55.9, 54.5, 52.33, 52.28, 29.4, 28.6 (3C), 28.4 (3C), 26.2 (3C), 20.8, 19.1, 18.7, -3.7, -4.8; HRMS: m/z (ESI) calcd for $C_{68}H_{97}N_2O_{24}Si^+$, $[M + H]^+$, 1353.6195, found 1353.6223. $^1J_{^{13}C1\text{–}H1}$ = 172.5 Hz, 170.5 Hz, 161.1 Hz. $^3J_{H1\text{–}H2}$ = 8.3 Hz.

c. Heparan Sulfate GlcN(α1-4)IdoA(α1-4)GlcN(α1-4)GlcA Module Synthesis

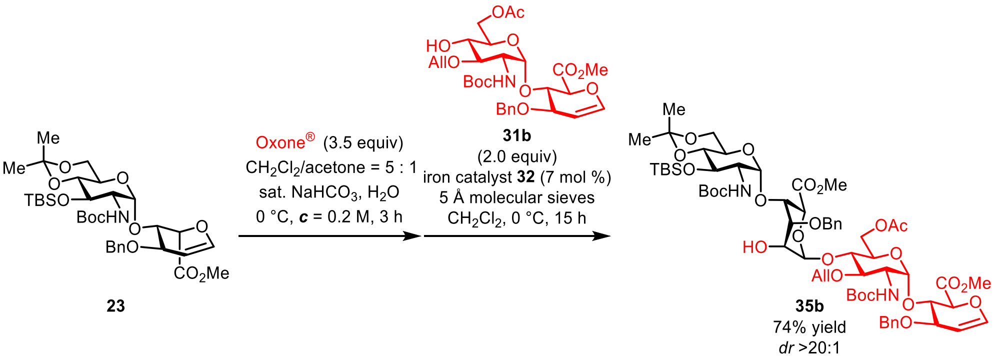

To a 100 mL flask equipped with a stir bar at 0 °C, were added glycal **23** (340 mg, 0.5 mmol, 1.0 equiv) in CH_2Cl_2/acetone mixture (v/v: 5:1, 8.3 mL) and saturated aqueous $NaHCO_3$ solution (14.2 mL), followed by addition of Oxone® ($KHSO_5 \cdot 0.5KHSO_4 \cdot 0.5K_2SO_4$) (1.08 g, 1.75 mmol, 3.5 equiv) in H_2O (9.2 mL) dropwise. After stirring vigorously at 0 °C for 3 h, the reaction mixture was extracted with CH_2Cl_2 (10 mL × 3). The combined organic phase was dried over anhydrous Na_2SO_4 and concentrated *in vacuo*. The residue was further azeotropically dried with anhydrous toluene (5 mL × 3). The obtained glycal epoxide was assayed by ^{1}H NMR to get the diastereomeric ratio (*dr* > 20:1) and directly used in the next step.

Stereochemistry Determination of Glycal Epoxide S24

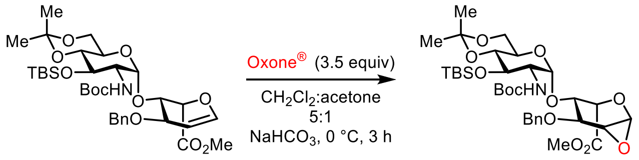

23 **S24**

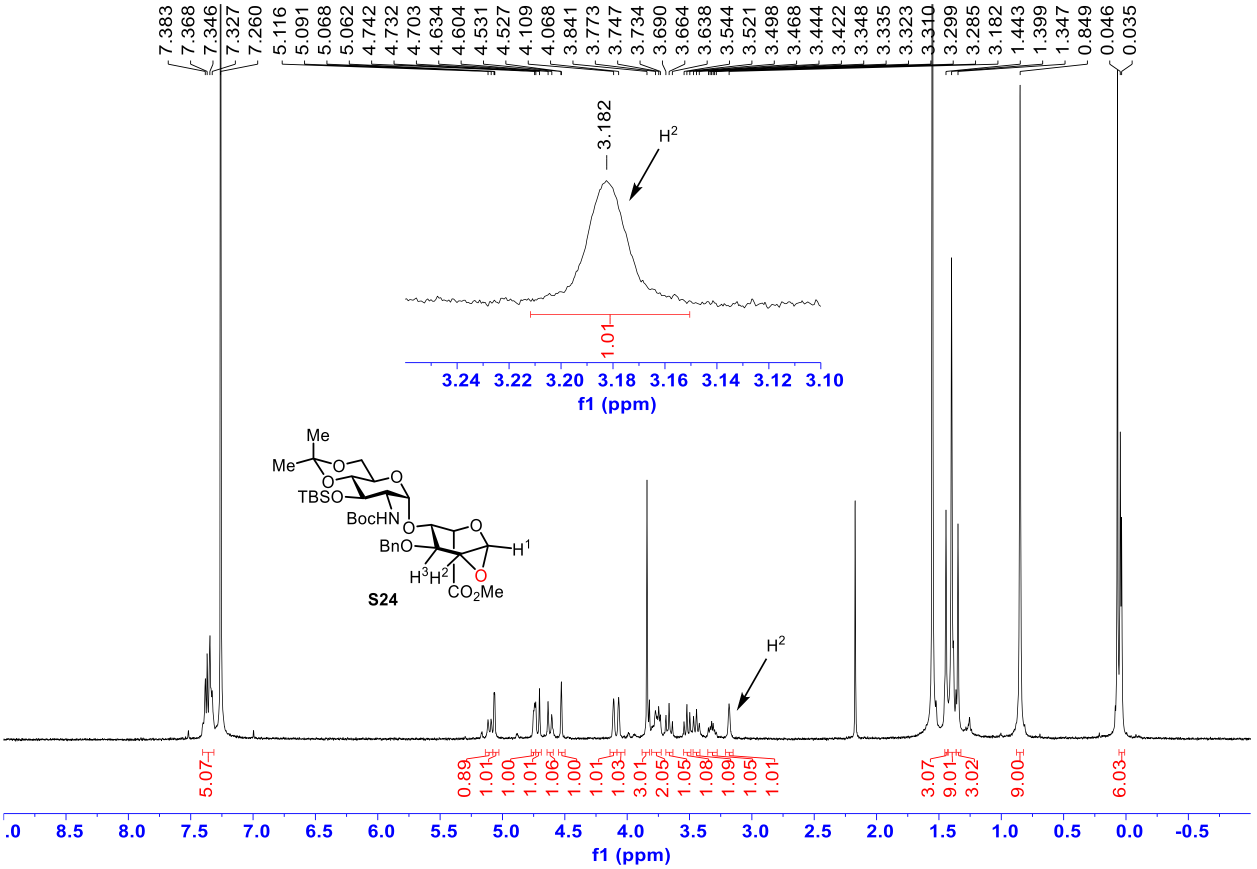

Figure S15. Stereochemistry Determination of Iduronate Ester α-Epoxide **S24**.

Analogous to a glucuronate ester α-epoxide, a typical iduronate ester α-epoxide is expected to have a dihedral angle ($\Phi_{1,2}$) of approximately 0° and a dihedral angle ($\Phi_{2,3}$) of about 90°. This conformation results in a small $^3J_{H1-H2}$ value and a negligible $^3J_{H2-H3}$ (~0 Hz). Consequently, the H2 resonance of an iduronate ester α-epoxide appears as a doublet, or as a broad singlet when $^3J_{H1-H2}$ value is too small to be resolved.

For glycal epoxide **S24**, the H2 resonance was observed as a broad singlet in the ^{1}H NMR spectrum, consistent with an α-epoxide assignment (*dr* > 20:1). This assignment was further corroborated by the stereochemistry analysis of the glycosylation product 1,2-*trans*-α-glycoside **35b**.

To a flame-dried sealable 2-dram vial equipped with a stir bar were added glycosyl acceptor **31b** (658 mg, 1.0 mmol, 2.0 equiv) and freshly activated 5 Å molecular sieves, powder (*ca.* 500 mg). After the vial was evacuated and backfilled with N_2, anhydrous CH_2Cl_2 (0.7 mL) was added. The iron porphyrin triflate catalyst **32** (0.035 mmol, 7 mol %) solution was added to the vial and the resulting mixture was stirred at −78 °C for 5 min before a solution of the aforementioned glycal epoxide in anhydrous CH_2Cl_2 (1.0 mL) was then added to the mixture at −78 °C dropwise. The reaction mixture was kept at 0 °C for 15 h and then quenched with a solution of imidazole (7 mg) in CH_2Cl_2 (0.1 mL) at the same temperature. The mixture was filtered through a pad of Celite® silica gel and eluted with CH_2Cl_2 (10 mL). The organic layer was then concentrated *in vacuo*, and the residue was purified through a silica gel flash column (hexanes/EtOAc: from 100:1 to 2:1) to afford the corresponding glycosylation product **35b** as white foam (482 mg, 74% yield).

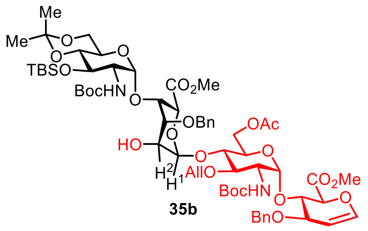

35b

Methyl 3-*O*-benzyl-4-*O*-[(2-*tert*-butoxycarbonylamino-3-*O*-*tert*-butyldimethylsilyl-4,6-*O*-isopropylidene-2-deoxy-α-D-glucopyranosyl)-(1→4)-(methyl 3-*O*-benzyl-α-L-idopyranosyluronate)-(1→4)-(6-*O*-acetyl-3-*O*-allyl-2-*tert*-butoxycarbonylamino-2-deoxy-α-D-glucopyranosyl)]-D-glucuronal (**35b**): $[\alpha]_D^{23}$ +22.2 (acetone, *c* = 1.0); IR ν_{max} (neat)/cm^{-1}: 3452 (w), 2976 (s), 2933 (m), 2191 (m), 1763 (s), 1718 (m), 1653 (w); ^{1}H NMR (400 MHz, $CDCl_3$) δ 7.75 – 7.20 (m, 10H), 6.62 (d, *J* = 6.3 Hz, 1H), 5.75 (ddt, *J* = 17.2, 10.5, 5.4 Hz, 1H), 5.12 (dd, *J* = 17.2, 1.7 Hz, 1H), 5.08 (d, *J* = 1.8 Hz, 1H), 5.05 (d, *J* = 3.7 Hz, 1H), 5.02 (dd, *J* = 10.5, 1.7 Hz, 1H), 5.01 – 4.97 (m, 2H), 4.96 (d, *J* = 2.8 Hz, 1H), 4.91 (d, *J* = 2.5 Hz, 1H), 4.78 (d,

J = 8.7 Hz, 1H), 4.63 (ABq, $\Delta\nu_{AB}$ = 42.8 Hz, J = 11.3 Hz, 2H), 4.54 – 4.47 (m, 2H), 4.43 (d, J = 11.3 Hz, 1H), 4.36 (s, 1H), 4.13 – 4.09 (m, 2H), 4.08 – 4.06 (m, 1H), 3.96 (dd, J = 12.7, 5.6 Hz, 1H), 3.93 – 3.87 (m, 2H), 3.86 – 3.84 (m, 1H), 3.82 – 3.78 (m, 4H), 3.77 – 3.70 (m, 5H), 3.66 (t, J = 10.5 Hz, 1H), 3.52 (s, 3H), 3.50 – 3.43 (m, 2H), 3.43 – 3.34 (m, 2H), 2.85 (brs, 1H), 2.10 (s, 3H), 1.45 (s, 3H), 1.42 (s, 9H), 1.38 (s, 9H), 1.35 (s, 3H), 0.84 (s, 9H), 0.04 (s, 3H), 0.03 (s, 3H); ^{13}C NMR (100 MHz, $CDCl_3$) δ 170.8, 169.6, 167.8, 155.4, 155.0, 145.4, 137.5 (two peaks overlapped, 2C), 134.5, 128.5 (2C), 128.4 (2C), 128.0 (two peaks overlapped, 3C), 127.9 (2C), 127.8, 116.3, 101.4, 99.3, 98.7, 98.0, 97.1, 79.9, 79.8, 78.0, 75.7, 74.4, 73.4, 73.3, 73.0, 72.7, 71.9, 71.7, 70.8, 70.5, 69.5, 68.8, 67.7, 66.7, 64.2, 62.7, 62.0, 55.7, 53.4, 52.2, 52.1, 29.1, 28.4 (3C), 28.3 (3C), 25.7 (3C), 20.9, 18.9, 18.2, -4.1, -5.1; HRMS: m/z (ESI) calcd for $C_{64}H_{95}N_2O_{24}Si^+$, $[M + H]^+$, 1303.6039, found 1303.6068. $^1J_{^{13}C1-H1}$ = 171.8 Hz, 170.8 Hz, 170.5 Hz. $^3J_{H1-H2}$ = 1.8 Hz.

The stereochemistry of newly formed anomeric center (C1) of **35b** was determined by measuring $^1J_{^{13}C1-H1}$ (170.8 Hz) through un-decoupled HSQC experiments.[11-12] The C2 stereochemistry was determined by the stereochemistry of glycal epoxidation and corroborated by measuring $^3J_{H1-H2}$ (1.8 Hz).

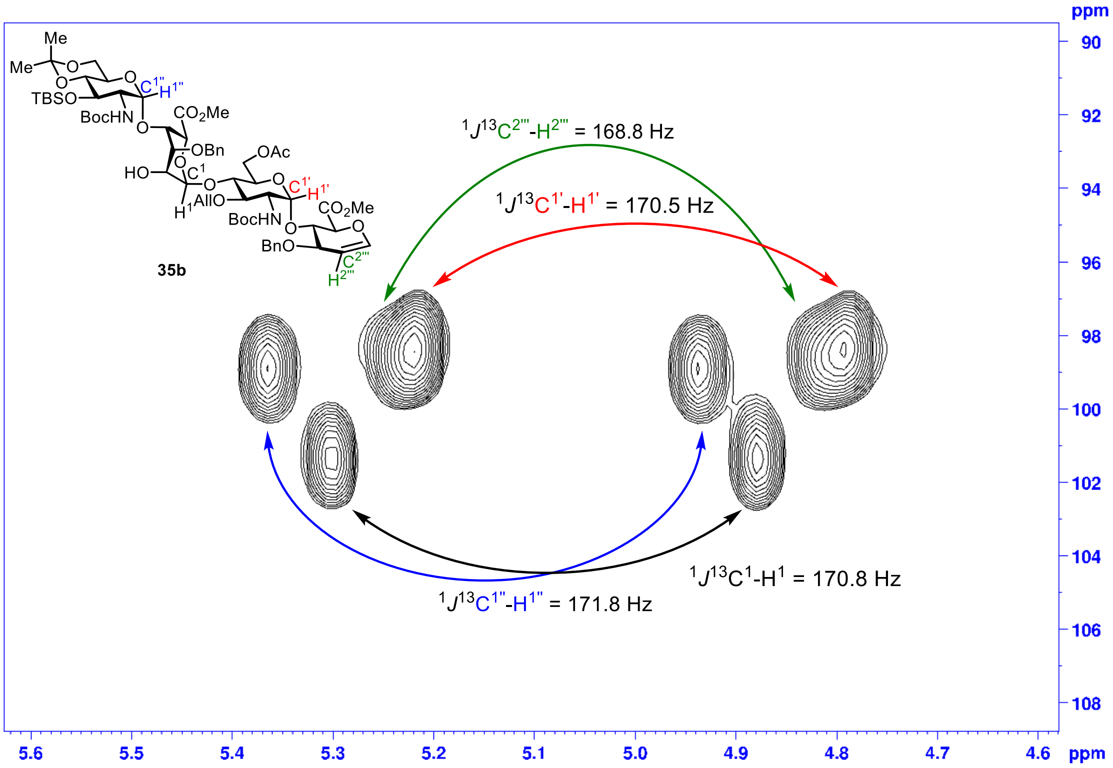

Figure S16. Un-decoupled HSQC Analysis to Determine Stereochemistry of **35b**.

NMR spectroscopic and X-ray crystallographic studies have demonstrated that L-iduronate esters preferentially adopt the $^{1}C_{4}$ conformation.[13-15] The $^{1}J_{^{13}C1-H1}$ value of 170.8 Hz suggested that the newly formed glycosidic bond in **35b** is in the axial position, which confirmed the formation of a 1,2-*trans*-α-glycosidic linkage.

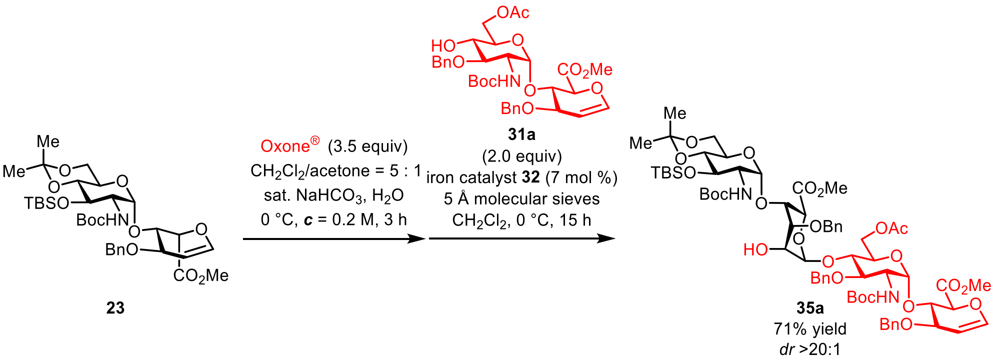

The epoxidation of **23** and the subsequent iron-catalyzed stereospecific glycosylation with glycosyl acceptor **31a** were carried out on a 0.5 mmol scale using procedures analogous to those described above. The desired glycosylation product **35a** was obtained through a silica gel flash column (hexanes/acetone: from 100:1 to 6:1) as white foam (481 mg, 71% yield). The stereochemistry of **35a** was determined by measuring $^3J_{H1-H2}$ (2.2 Hz) and $^1J_{^{13}C1-H1}$ (169.6 Hz).

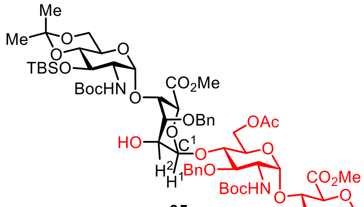

35a

Methyl 3-*O*-benzyl-4-*O*-[(2-*tert*-butoxycarbonylamino-3-*O*-*tert*-butyldimethylsilyl-4,6-*O*-isopropylidene-2-deoxy-α-D-glucopyranosyl)-(1→4)-(methyl 3-*O*-benzyl-α-L-idopyranosyluronate)-(1→4)-(6-*O*-acetyl-3-*O*-benzyl-2-*tert*-butoxycarbonylamino-2-deoxy-α-D-glucopyranosyl)]-D-glucuronal (**35a**): $[\alpha]_D^{23}$ +36.8 (acetone, *c* = 1.0); IR ν_{max} (neat)/cm^{-1}: 3456 (w), 2970 (w), 2952 (w), 2361 (m), 1739 (s), 1652 (w), 1558 (m); ^{1}H NMR (400 MHz, $CDCl_3$) δ 7.35 – 7.11 (m, 15H), 6.61 (d, *J* = 6.3 Hz, 1H), 5.08 (d, *J* = 2.2 Hz, 1H), 5.03 – 4.93 (m, 3H), 4.93 – 4.87 (m, 1H), 4.86 – 4.74 (m, 2H), 4.67 (d, *J* = 11.2 Hz, 1H), 4.64 – 4.55 (m, 2H), 4.51 – 4.45 (m, 3H), 4.43 – 4.37 (m, 2H), 4.34 (d, *J* = 2.1 Hz, 1H), 4.12 (dd, *J* = 12.3, 5.1 Hz, 1H), 4.00 – 3.87 (m, 3H), 3.85 – 3.80 (m, 2H), 3.79 – 3.75 (m, 1H), 3.75 – 3.66 (m, 4H), 3.65 – 3.57 (m, 2H), 3.53 – 3.46 (m, 4H), 3.46 – 3.35 (m, 4H), 3.34 – 3.21 (m, 1H), 2.82 (d, *J* = 9.5 Hz, 1H), 2.08 (s, 3H), 1.40 (s, 3H), 1.34 (s, 9H), 1.30 (s, 3H), 1.28 (s, 9H), 0.81 (s, 9H), -0.01 (s, 3H), -0.01 (s, 3H); ^{13}C NMR (100 MHz, $CDCl_3$) δ 170.8, 169.2, 167.8, 155.3, 154.9, 145.4, 138.0, 137.4 (two peaks overlapped, 2C), 128.4 (2C), 128.3 (2C), 128.0 (2C), 127.94 (2C), 127.91 (2C),

127.8 (2C), 127.12 (two peaks overlapped, 2C), 127.07, 101.1, 99.2, 98.7, 98.0, 97.1, 79.72, 79.68, 78.0, 75.7, 74.3, 73.4 (two peaks overlapped, 2C), 73.1, 73.0, 72.8, 71.6, 70.7, 70.6, 69.4, 68.8, 67.9, 66.7, 64.1, 62.7, 61.9, 55.6, 53.5, 52.0, 51.8, 29.0, 28.2 (3C), 28.1 (3C), 25.7 (3C), 20.8, 18.8, 18.1, -4.2, -5.2; HRMS: m/z (ESI) calcd for $C_{68}H_{97}N_2O_{24}Si^+$, $[M + H]^+$, 1353.6195, found 1353.6132. $^1J_{^{13}C1-H1}$ = 169.6 Hz, 172.8 Hz, 169.6 Hz. $^3J_{H1-H2}$ = 2.2 Hz.

d. Heparan Sulfate GlcN(α1-4)IdoA(α1-4)GlcN(α1-4)IdoA Module Synthesis

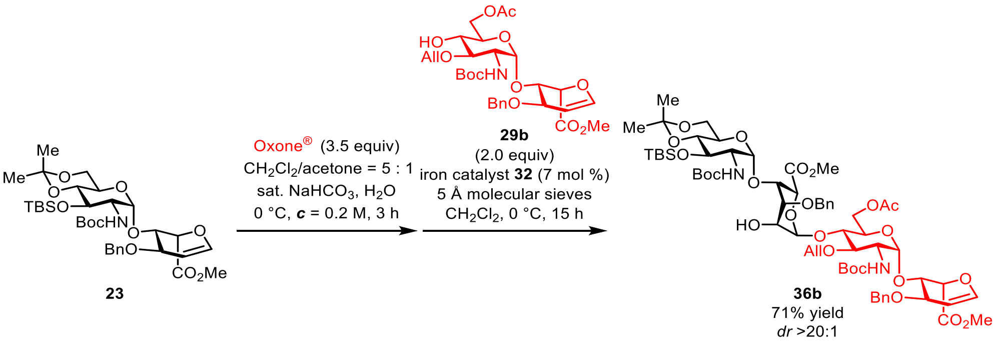

To a 100 mL flask equipped with a stir bar at 0 °C, were added glycal **23** (340 mg, 0.5 mmol, 1.0 equiv) in CH_2Cl_2/acetone mixture (v/v: 5:1, 8.3 mL) and saturated aqueous $NaHCO_3$ solution (14.2 mL), followed by addition of Oxone® ($KHSO_5 \cdot 0.5KHSO_4 \cdot 0.5K_2SO_4$) (1.08 g, 1.75 mmol, 3.5 equiv) in H_2O (9.2 mL) dropwise. After stirring vigorously at 0 °C for 3 h, the reaction mixture was extracted with CH_2Cl_2 (10 mL × 3). The combined organic phase was dried over anhydrous Na_2SO_4 and concentrated *in vacuo*. The residue was further azeotropically dried with anhydrous toluene (5 mL × 3). The obtained glycal epoxide was assayed by ^{1}H NMR to get the diastereomeric ratio (*dr* > 20:1) and directly used in the next step.

To a flame-dried sealable 2-dram vial equipped with a stir bar were added glycosyl acceptor **29b** (658 mg, 1.0 mmol, 2.0 equiv) and freshly activated 5 Å molecular sieves, powder (*ca.* 500 mg). After the vial was evacuated and backfilled with N_2, anhydrous CH_2Cl_2 (0.7 mL) was added. The iron porphyrin triflate catalyst **32** (0.035 mmol, 7 mol %) solution was added to the vial and the resulting mixture was stirred at −78 °C for 5 min before a solution of the aforementioned glycal epoxide in anhydrous CH_2Cl_2 (1.0 mL) was then added to the mixture at −78 °C dropwise. The reaction mixture was kept at 0 °C for 15 h and then quenched with a solution of imidazole (7

mg) in CH_2Cl_2 (0.1 mL) at the same temperature. The mixture was filtered through a pad of Celite® silica gel and eluted with CH_2Cl_2 (10 mL). The organic layer was then concentrated *in vacuo*, and the residue was purified through a silica gel flash column (hexanes/EtOAc: from 100:1 to 3:1) to afford the corresponding glycosylation product **36b** as white foam (463 mg, 71% yield).

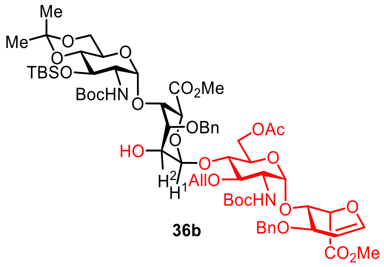

36b

Methyl 3-*O*-benzyl-4-*O*-[(2-*tert*-butoxycarbonylamino-3-*O*-*tert*-butyldimethylsilyl-4,6-*O*-isopropylidene-2-deoxy-α-D-glucopyranosyl)-(1→4)-(methyl 3-*O*-benzyl-α-L-idopyranosyluronate)-(1→4)-(6-*O*-acetyl-3-*O*-allyl-2-*tert*-butoxycarbonylamino-2-deoxy-α-D-glucopyranosyl)]-L-guluronal (**36b**): $[\alpha]_D^{23}$ −3.9 (acetone, *c* = 1.0); IR ν_{max} (neat)/cm^{-1}: 3568 (w), 3444 (w), 2976 (m), 2934 (m), 2161 (w), 1768 (m), 1718 (m); ^{1}H NMR (400 MHz, $CDCl_3$) δ 7.59 – 7.27 (m, 10H), 6.65 (d, *J* = 6.1 Hz, 1H), 5.74 (ddt, *J* = 17.3, 10.6, 5.4 Hz, 1H), 5.11 (dd, *J* = 17.3, 1.7 Hz, 1H), 5.06 (d, *J* = 2.2 Hz, 1H), 5.03 (d, *J* = 3.1 Hz, 1H), 5.01 (dd, *J* = 10.6, 1.7 Hz, 1H), 5.00 – 4.93 (m, 2H), 4.82 (d, *J* = 3.5 Hz, 1H), 4.80 (d, *J* = 9.2 Hz, 1H), 4.66 (d, *J* = 11.0 Hz, 1H), 4.63 – 4.53 (m, 4H), 4.53 – 4.42 (m, 2H), 4.32 – 4.27 (m, 1H), 4.18 – 4.01 (m, 3H), 3.95 (dd, *J* = 12.7, 5.4 Hz, 1H), 3.87 – 3.80 (m, 2H), 3.79 – 3.75 (m, 5H), 3.75 – 3.69 (m, 5H), 3.68 – 3.59 (m, 3H), 3.54 – 3.43 (m, 2H), 3.40 – 3.28 (m, 2H), 2.82 (d, *J* = 9.7 Hz, 1H), 2.14 (s, 3H), 1.45 (s, 3H), 1.39 (s, 9H), 1.38 (s, 9H), 1.35 (s, 3H), 0.84 (s, 9H), 0.04 (s, 3H), 0.03 (s, 3H); ^{13}C NMR (100 MHz, $CDCl_3$) δ 170.8, 169.6, 168.6, 155.4, 155.0, 146.1, 137.5, 137.4, 134.5, 128.54 (2C), 128.53 (2C), 128.00, 128.98, 127.8 (2C), 127.7 (2C), 116.3, 101.3, 99.3, 98.5, 97.0, 96.1, 80.0, 79.7, 77.9, 75.3, 74.4, 73.1, 72.7, 72.4, 72.3, 71.5, 71.2, 70.8, 70.3, 70.2, 68.6, 67.7, 65.8, 64.2, 62.3, 62.0, 55.7, 53.4, 52.5, 52.1, 29.0, 28.31 (3C), 28.28 (3C), 25.7 (3C), 20.9, 18.9, 18.2, -4.1, -5.2; HRMS: m/z (ESI) calcd for $C_{64}H_{95}N_2O_{24}Si^+$, $[M + H]^+$, 1303.6039, found 1303.5992. $^1J_{^{13}C1-H1}$ = 170.1 Hz, 175.5 Hz, 173.3 Hz. $^3J_{H1-H2}$ = 2.2 Hz.

The stereochemistry of newly formed anomeric center (C1) of **36b** was determined by measuring $^{1}J_{^{13}C1–H1}$ (170.1 Hz) through un-decoupled HSQC experiments.[11-12] The C2 stereochemistry determined by the stereochemistry of glycal epoxidation and corroborated by measuring $^{3}J_{H1–H2}$ (2.2 Hz).

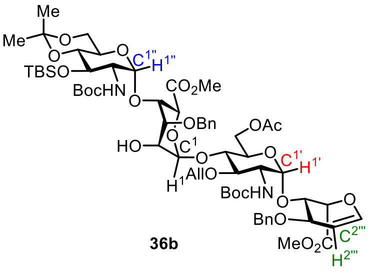

36b

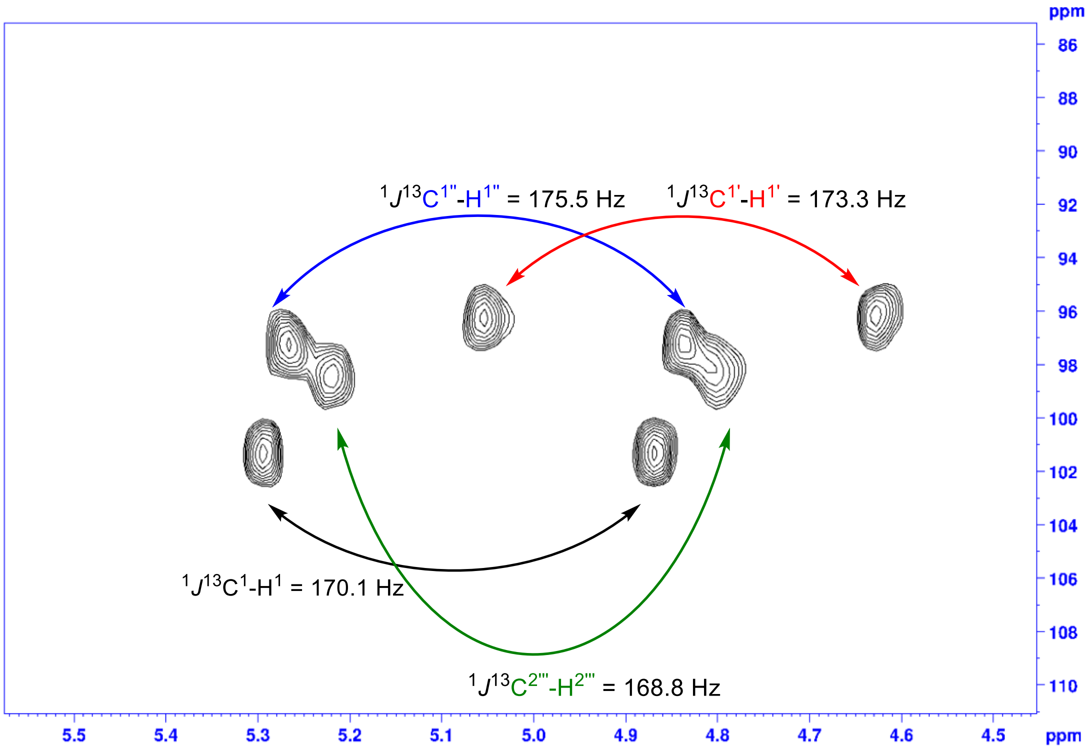

Figure S17. Un-decoupled HSQC Analysis to Determine Stereochemistry of **36b**.

A $^{1}J_{^{13}C1–H1}$ value of 170.1 Hz suggested that the newly formed glycosidic bond is in axial position, which confirmed the formation of a 1,2-*trans*-α-glycosidic linkage.

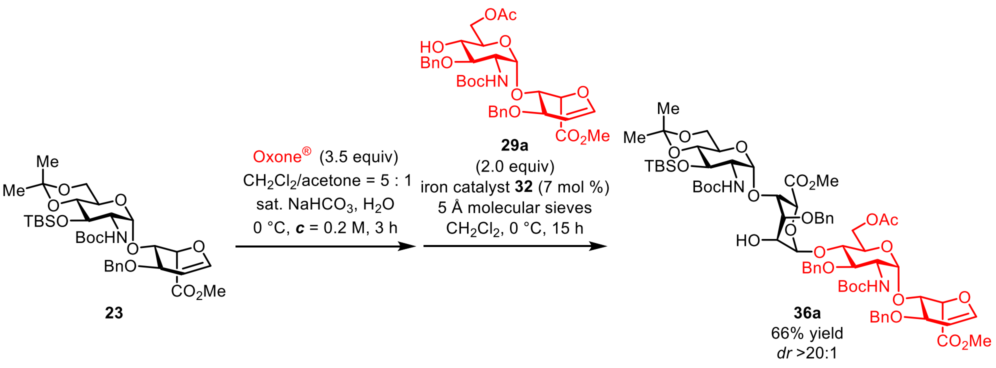

The epoxidation of **23** and the subsequent iron-catalyzed stereospecific glycosylation with glycosyl acceptor **29a** were carried out on a 0.5 mmol scale using procedures analogous to those described above. The desired glycosylation product **36a** was obtained through a silica gel flash column (hexanes/EtOAc: from 100:1 to 4:1) as white foam (447 mg, 66% yield). The stereochemistry of **36a** was determined by measuring $^{3}J_{H1-H2}$ (2.4 Hz) and $^{1}J^{13}{}_{C1-H1}$ (170.9 Hz).

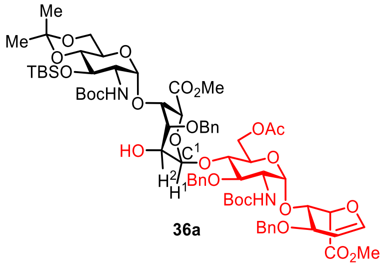

Methyl 3-*O*-benzyl-4-*O*-[(2-*tert*-butoxycarbonylamino-3-*O*-*tert*-butyldimethylsilyl-4,6-*O*-isopropylidene-2-deoxy-α-D-glucopyranosyl)-(1→4)-(methyl 3-*O*-benzyl-α-L-idopyranosyluronate)-(1→4)-(6-*O*-acetyl-3-*O*-benzyl-2-*tert*-butoxycarbonylamino-2-deoxy-α-D-glucopyranosyl)]-L-guluronal (**36a**): $[\alpha]_D^{23}$ +3.6 (acetone, *c* = 1.0); IR ν_{max} (neat)/cm^{-1}: 3446 (w), 2970 (m), 2361 (m), 1739 (s), 1721 (s), 1652 (w), 1558 (s); ^{1}H NMR (800 MHz, C_6D_6) δ 7.37 (d, *J* = 7.5 Hz, 2H), 7.31 (d, *J* = 7.5 Hz, 2H), 7.27 (t, *J* = 7.5 Hz, 2H), 7.20 (d, *J* = 7.5 Hz, 2H), 7.19 – 7.14 (m, 4H), 7.13 – 7.08 (m, 2H), 7.05 (d, *J* = 7.5 Hz, 1H), 6.41 (d, *J* = 6.1 Hz, 1H), 5.38 (d, *J* = 2.4 Hz, 1H), 5.24 – 5.18 (m, 2H), 5.01 (s, 1H), 4.86 (d, *J* = 3.7 Hz, 1H), 4.84 – 4.76 (m, 1H), 4.69 – 4.64 (m, 2H), 4.63 – 4.58 (m, 2H), 4.49 (d, *J* = 11.1 Hz, 1H), 4.43 – 4.35 (m, 2H), 4.32 (d, *J* = 11.0 Hz, 1H), 4.29 (d, *J* = 11.7 Hz, 1H), 4.27 – 4.22 (m, 2H), 4.23 – 4.15 (m, 3H),

3.98 – 3.92 (m, 2H), 3.90 – 3.87 (m, 1H), 3.85 (s, 1H), 3.80 – 3.76 (m, 1H), 3.73 – 3.67 (t, J = 8.5 Hz, 1H), 3.64 – 3.60 (m, 1H), 3.58 (d, J = 5.1 Hz, 1H), 3.56 – 3.54 (m, 1H), 3.54 – 3.49 (m, 1H), 3.43 (s, 4H), 3.40 – 3.31 (m, 4H), 1.76 (s, 3H), 1.51 (s, 9H), 1.35 (s, 3H), 1.31 (s, 9H), 1.19 (s, 3H), 1.02 (d, J = 1.9 Hz, 9H), 0.14 (s, 3H), 0.10 (s, 3H); ^{13}C NMR (100 MHz, $CDCl_3$) δ 170.9, 169.3, 168.6, 155.4, 155.0, 146.1, 138.1, 137.5, 137.4, 128.6 (2C), 128.5 (2C), 128.1 (2C), 127.98 (2C), 127.97 (2C), 127.7 (2C), 127.2 (two peaks overlapped, 2C), 127.1, 101.1, 99.3, 98.5, 97.0, 96.3, 79.7, 79.6, 77.9, 75.4, 74.4, 73.4, 73.3, 72.9, 72.4, 71.44, 71.38, 70.8, 70.4, 70.2, 68.6, 67.9, 66.0, 64.1, 62.3, 62.0, 55.6, 53.4, 52.5, 51.9, 29.0, 28.3 (3C), 28.2 (3C), 25.7 (3C), 20.9, 18.9, 18.2, -4.1, -5.2; HRMS: m/z (ESI) calcd for $C_{68}H_{97}N_2O_{24}Si^+$, $[M + H]^+$, 1353.6195, found 1353.6229. $^1J_{^{13}C1-H1}$ = 175.2 Hz, 174.4 Hz, 170.9 Hz. $^3J_{H1-H2}$ = 2.4 Hz.

H. Assembly of Heparan Sulfate GlcN(α1-4)GlcA(β1-4)GlcN(α1-4)IdoA(α1-4)GlcN(α1-4)GlcA Module and Chemo-Enzymatic Sulfation for Heparan Sulfate Hexasaccharide Synthesis

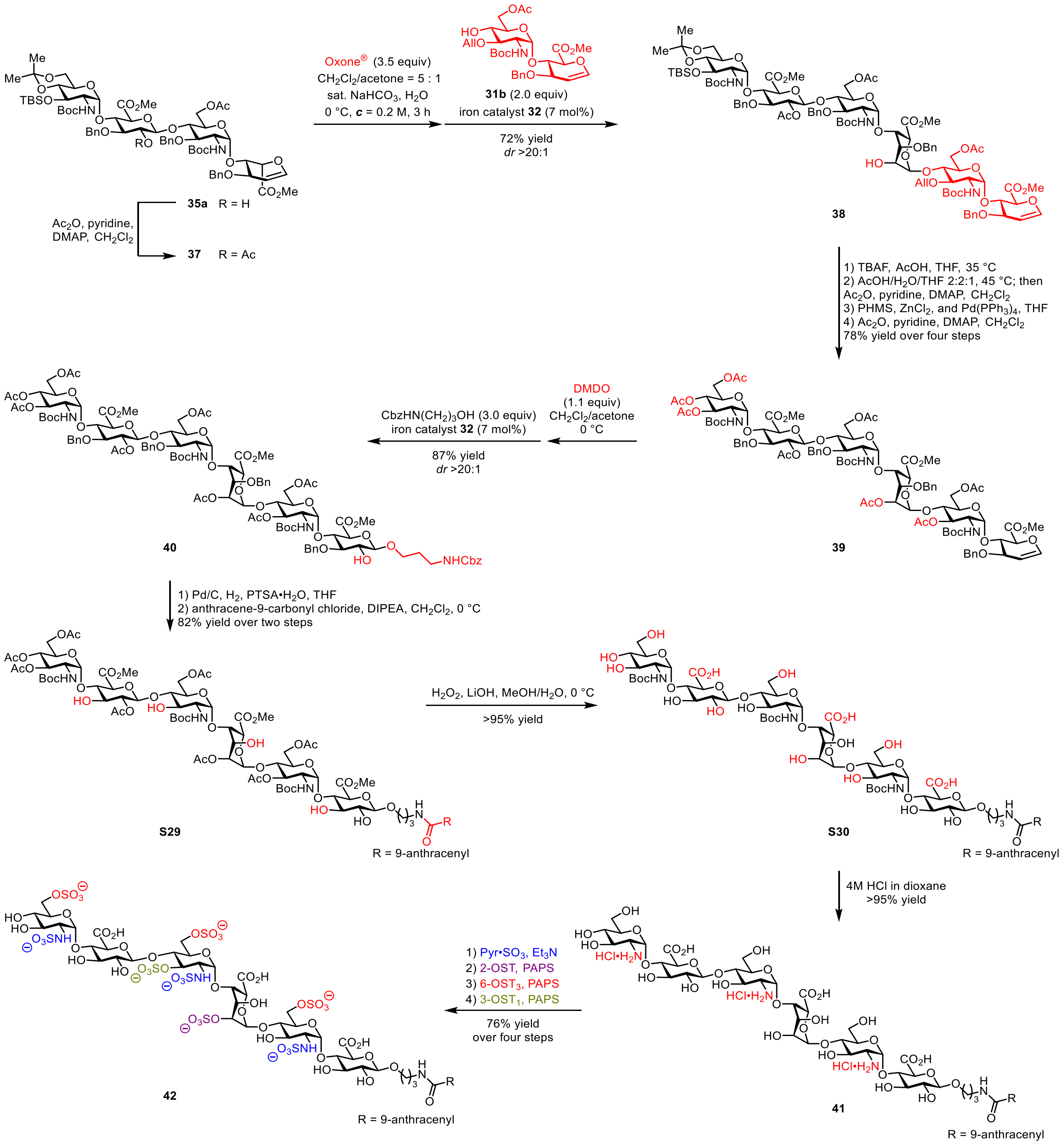

Figure S18. Heparan Sulfate Hexasaccharide **42** Synthesis.

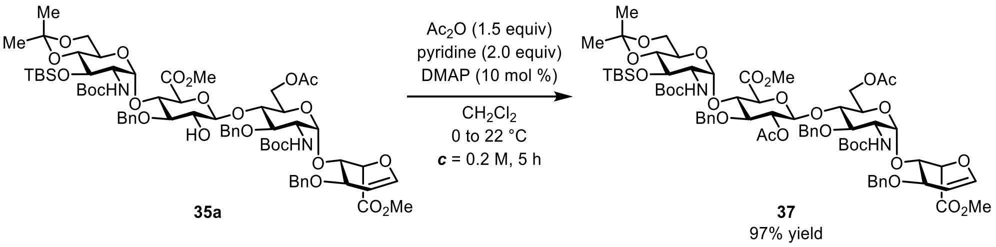

To a flame-dried sealable 2-dram vial equipped with a stir bar were added tetrasaccharide **35a** (271 mg, 0.2 mmol, 1.0 equiv) and DMAP (2.4 mg, 0.02 mmol, 10 mol %). After the vial was evacuated and backfilled with N_2, anhydrous CH_2Cl_2 (1.0 mL) was added. The solution was then cooled to 0 °C before pyridine (32 μL, 0.4 mmol, 2.0 equiv) and Ac_2O (28 μL, 0.3 mmol, 1.5 equiv) were added dropwise sequentially. The reaction mixture was gradually warmed to room temperature and stirred for 5 h, with progress monitored by TLC until completion. The reaction mixture was then diluted with CH_2Cl_2 (2 mL) and quenched with saturated aqueous NH_4Cl solution (1.5 mL). The organic phase was separated from the aqueous one, which was further extracted with CH_2Cl_2 (3 mL × 3). The combined organic phase was dried over Na_2SO_4. After concentration *in vacuo*, the residue was purified through a silica gel flash column (hexanes/EtOAc: from 100:1 to 2:1) to afford the desired product **37** (271 mg, 97% yield) as white foam.

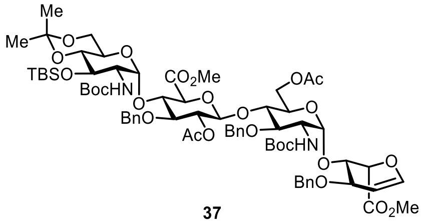

37

Methyl 3-*O*-benzyl-4-*O*-[(2-*tert*-butoxycarbonylamino-3-*O*-*tert*-butyldimethylsilyl-4,6-*O*-isopropylidene-2-deoxy-α-D-glucopyranosyl)-(1→4)-(methyl 2-*O*-acetyl-3-*O*-benzyl-β-D-glucopyranosyluronate)-(1→4)-(6-*O*-acetyl-3-*O*-benzyl-2-*tert*-butoxycarbonylamino-2-deoxy-α-D-glucopyranosyl)]-L-guluronal (**37**): $[\alpha]_D^{23}$ +13.4 (acetone, *c* = 1.0); IR ν_{max} (neat)/cm^{-1}: 3649 (w), 3629 (w), 2977 (s), 2191 (m), 2037 (s), 1751 (m), 1717 (s); ^{1}H NMR (400 MHz, $CDCl_3$) δ 7.55 – 7.03 (m, 15H), 6.62 (d, *J* = 6.2 Hz, 1H), 5.25 (d, *J* = 3.8 Hz, 1H), 5.14 – 5.00 (m, 1H), 4.99 – 4.88 (m, 2H), 4.85 (d, *J* = 3.6 Hz, 1H), 4.77 (d, *J* = 10.1 Hz, 1H), 4.70 (d, *J* = 10.8 Hz, 1H), 4.61 (d, *J* = 11.8 Hz, 1H), 4.59 – 4.55 (m, 2H), 4.55 – 4.51 (m, 2H), 4.48 (d, *J* =

11.5 Hz, 1H), 4.42 (d, J = 12.0 Hz, 1H), 4.36 (d, J = 9.2 Hz, 1H), 4.31 – 4.25 (m, 1H), 4.24 – 4.17 (m, 1H), 4.15 (t, J = 9.2 Hz, 1H), 3.88 – 3.81 (m, 3H), 3.79 (s, 3H), 3.77 – 3.73 (m, 2H), 3.72 – 3.67 (m, 2H), 3.67 – 3.61 (m, 2H), 3.59 (s, 3H), 3.53 – 3.37 (m, 3H), 3.27 – 3.16 (m, 1H), 2.15 (s, 3H), 1.94 (s, 3H), 1.43 (s, 3H), 1.35 (s, 3H), 1.34 (s, 9H), 1.33 (s, 9H), 0.86 (s, 9H), 0.06 (s, 3H), 0.05 (s, 3H); ^{13}C NMR (100 MHz, $CDCl_3$) δ 170.6, 169.0, 168.9, 167.7, 155.2, 154.9, 145.8, 138.5, 137.5, 136.9, 128.49 (2C), 128.47 (2C), 128.1 (2C), 127.91, 127.87, 127.8 (2C), 127.7 (2C), 127.6 (2C), 127.2, 101.0, 99.2, 99.0, 98.5, 95.6, 82.7, 79.7, 79.5, 77.9, 77.6, 75.0, 74.9, 74.7, 74.3, 74.2, 72.8, 72.4, 71.2, 71.0, 70.1, 69.4, 65.6, 64.3, 61.7, 61.6, 55.2, 53.5, 52.7, 52.5, 29.0, 28.3 (3C), 28.2 (3C), 25.7 (3C), 20.9, 20.5, 18.8, 18.2, -4.2, -5.1; HRMS: m/z (ESI) calcd for $C_{70}H_{99}N_2O_{25}Si^+$, $[M + H]^+$, 1395.6301, found 1395.6319.

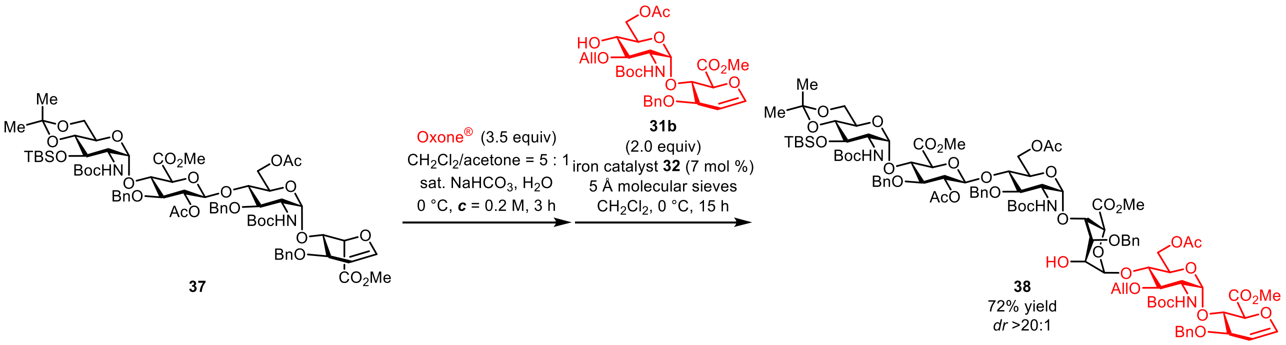

To a 50 mL flask equipped with a stir bar at 0 °C, were added glycal **37** (350 mg, 0.25 mmol, 1.0 equiv) in CH_2Cl_2/acetone mixture (v/v: 5:1, 4.2 mL) and saturated aqueous $NaHCO_3$ solution (7.1 mL), followed by addition of Oxone® ($KHSO_5 \cdot 0.5KHSO_4 \cdot 0.5K_2SO_4$) (540 mg, 0.88 mmol, 3.5 equiv) in H_2O (4.6 mL) dropwise. After stirring vigorously at 0 °C for 3 h, the reaction mixture was extracted with CH_2Cl_2 (10 mL × 3). The combined organic phase was dried over anhydrous Na_2SO_4 and concentrated *in vacuo*. The residue was further azeotropically dried with anhydrous toluene (3.0 mL × 3 mL). The obtained glycal epoxide was assayed by ^{1}H NMR to get the diastereomeric ratio (*dr* > 20:1) and directly used in the next step.

Determination of the Stereochemistry of Glycal Epoxide S25

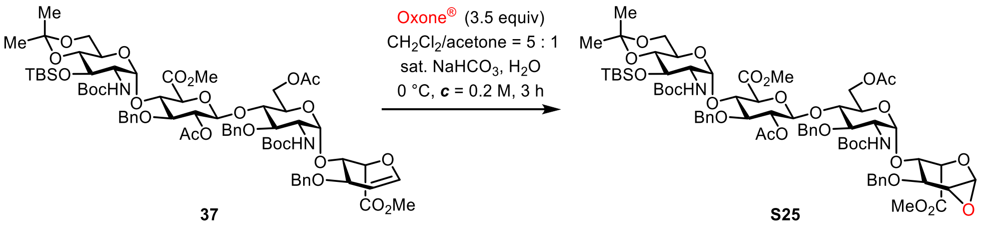

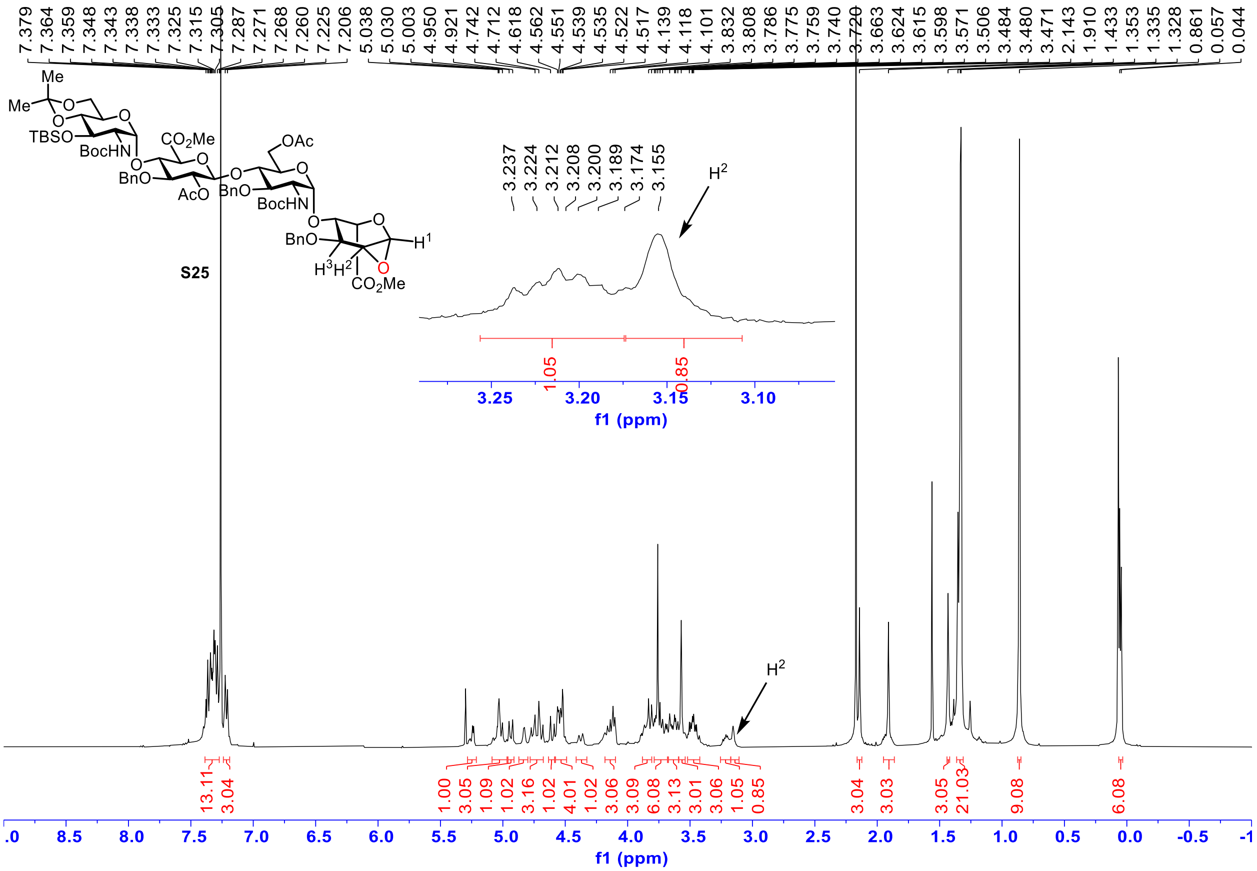

Figure S19. Stereochemistry Determination of Glycal Epoxide **S25**.

Analogous to glucuronate ester α-epoxides, a typical iduronate ester β-epoxide is expected to have a dihedral angle ($\Phi_{1,2}$) of approximately 0° and a dihedral angle ($\Phi_{2,3}$) of about 90°. This conformation results in a small $^3J_{H1-H2}$ value and a negligible $^3J_{H2-H3}$ (~0 Hz). Consequently, the H2 resonance of an iduronate ester α-epoxide appears as a doublet, or as a broad singlet when $^3J_{H1-H2}$ value is too small to be resolved.

For glycal epoxide **S25**, the H2 resonance was observed as a broad singlet in the ^{1}H NMR spectrum, consistent with an α-epoxide assignment (*dr* > 20:1). This assignment was further corroborated by the stereochemistry analysis of the glycosylation product 1,2-*trans*-α-glycoside **38**.

To a flame-dried sealable 2-dram vial (vial **A**) equipped with a stir bar were added glycosyl acceptor **31b** (304 mg, 0.5 mmol, 2.0 equiv) and freshly activated 5 Å molecular sieves, powder (*ca.* 200 mg). After the vial was evacuated and backfilled with N_2, anhydrous CH_2Cl_2 (0.3 mL) was added. To a second flame-dried sealable 2-dram vial (vial **B**) equipped with a stir bar were added the iron porphyrin triflate catalyst **32** (0.018 mmol, 7 mol %), and freshly activated 5 Å molecular sieves, powder (*ca.* 30 mg). Vial **B** was evacuated and backfilled with N_2 three times and anhydrous CH_2Cl_2 (0.3 mL) was added. The mixture in vial **B** was stirred at room temperature for 5 min before it was transferred to vial **A**. The mixture was stirred at −78 °C for 5 min and a solution of the aforementioned glycal epoxide in anhydrous CH_2Cl_2 (0.4 mL) was then added to the mixture at −78 °C dropwise. The reaction mixture was kept at 0 °C for 15 h and then quenched with a solution of imidazole (4 mg) in CH_2Cl_2 (0.1 mL) at the same temperature. The mixture was filtered through a pad of Celite® silica gel and eluted with CH_2Cl_2 (5 mL). The organic layer was then concentrated *in vacuo*, and the residue was purified through a silica gel flash column (CH_2Cl_2/MeOH: from 100:1 to 30:1) to afford the corresponding glycosylation product **38** as white foam (363 mg, 72% yield).

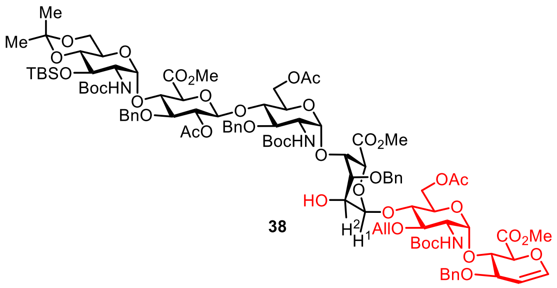

38

Methyl 3-*O*-benzyl-4-*O*-[(2-*tert*-butoxycarbonylamino-3-*O*-*tert*-butyldimethylsilyl-4,6-*O*-isopropylidene-2-deoxy-α-D-glucopyranosyl)-(1→4)-(methyl 2-*O*-acetyl-3-*O*-benzyl-β-D-glucopyranosyluronate)-(1→4)-(6-*O*-acetyl-3-*O*-benzyl-2-*tert*-butoxycarbonylamino-2-deoxy-α-D-glucopyranosyl)-(1→4)-(methyl 3-*O*-benzyl-α-L-idopyranosyluronate)-(1→4)-(6-

***O*-acetyl-3-*O*-allyl-2-*tert*-butoxycarbonylamino-2-deoxy-α-D-glucopyranosyl)]-D-glucuronal** (**38**): $[\alpha]_D^{23}$ +23.8 (acetone, *c* = 0.5); IR ν_{max} (neat)/cm^{-1}: 3453 (w), 2928 (m), 2358 (w), 1723 (s), 1650 (w), 1367 (s), 1246 (s); ^{1}H NMR (400 MHz, acetone-d_6) δ 7.46 – 7.09 (m, 20H), 6.58 (d, *J* = 6.3 Hz, 1H), 6.27 (d, *J* = 9.4 Hz, 1H), 5.89 (d, *J* = 10.1 Hz, 1H), 5.88 – 5.78 (m, 1H), 5.78 (d, *J* = 10.0 Hz, 3H), 5.40 (d, *J* = 3.9 Hz, 1H), 5.23 (d, *J* = 4.8 Hz, 1H), 5.14 (d, *J* = 3.8 Hz, 1H), 5.14 (d, *J* = 17.0 Hz, 1H), 5.08 (d, *J* = 3.6 Hz, 1H), 5.06 – 5.03 (m, 1H), 5.02 – 5.01 (m, 1H), 5.01 – 4.99 (m, 1H), 4.97 (d, *J* = 2.0 Hz, 1H), 4.88 – 4.81 (m, 2H), 4.79 (d, *J* = 8.0 Hz, 1H), 4.79 – 4.71 (m, 2H), 4.68 (d, *J* = 10.4 Hz, 1H), 4.59 (d, *J* = 11.4 Hz, 1H), 4.56 – 4.51 (m, 2H), 4.51 – 4.44 (m, 2H), 4.43 – 4.40 (m, 1H), 4.34 – 4.24 (m, 3H), 4.16 – 4.08 (m, 4H), 4.07 – 4.03 (m, 1H), 4.03 – 3.87 (m, 4H), 3.86 – 4.76 (m, 4H), 3.78 – 3.70 (m, 5H), 3.70 – 3.65 (m, 5H), 3.64 – 3.54 (m, 3H), 3.53 – 3.49 (m, 4H), 3.29 – 3.20 (m, 1H), 2.09 (s, 3H), 2.08 (s, 3H), 2.06 (s, 3H), 1.50 (s, 3H), 1.40 (s, 9H), 1.34 (s, 3H), 1.34 (s, 9H), 1.26 (s, 9H), 0.88 (s, 9H), 0.10 (s, 3H), 0.08 (s, 3H); ^{13}C NMR (100 MHz, acetone-d_6) δ 171.2, 170.9 (two peaks overlapped, 2C), 169.9, 169.4, 168.5, 156.4, 156.3, 156.2, 145.8, 140.4, 139.6, 139.4, 138.6, 136.7, 129.3 (2C), 129.2 (2C), 129.1 (2C), 129.0 (2C), 128.9 (2C), 128.8 (2C), 128.71, 128.67 (2C), 128.6 (2C), 128.3, 128.3, 127.8, 115.8, 101.8 (two peaks overlapped, 2C), 99.9, 99.7, 99.5, 99.4, 98.4, 83.3, 79.33, 79.29 (two peaks overlapped, 2C), 79.2, 79.1, 78.9 (two peaks overlapped, 2C), 78.2, 77.6, 75.6, 75.5, 75.2, 74.9, 74.4, 74.3, 74.2, 74.1, 73.74, 73.66, 72.1, 71.6, 71.4, 71.0, 70.5, 70.1, 68.6, 65.1, 63.5, 63.1, 62.4, 56.4, 55.3, 55.0, 52.9, 52.5, 52.2, 29.6, 28.8 (3C), 28.7 (3C), 28.6 (3C), 26.3 (3C), 20.92, 20.89, 20.86, 19.3, 18.9, -3.7, -4.8; HRMS: m/z (ESI) calcd for $C_{100}H_{140}N_3O_{38}Si^+$, [M + H]$^+$, 2018.8879, found 2018.8826. $^1J_{^{13}C1–H1}$ = 173.2 Hz, 170.5 Hz, 170.2 Hz, 175.0 Hz, 162.0 Hz. $^3J_{H1–H2}$ = 4.8 Hz.

The stereochemistry of newly formed anomeric center (C1) of **38** was determined by measuring $^1J_{^{13}C1–H1}$ (170.2 Hz) through un-decoupled HSQC experiments.[11-12] The C2 stereochemistry determined by the stereochemistry of glycal epoxidation and corroborated by measuring $^3J_{H1–H2}$ (4.8 Hz).

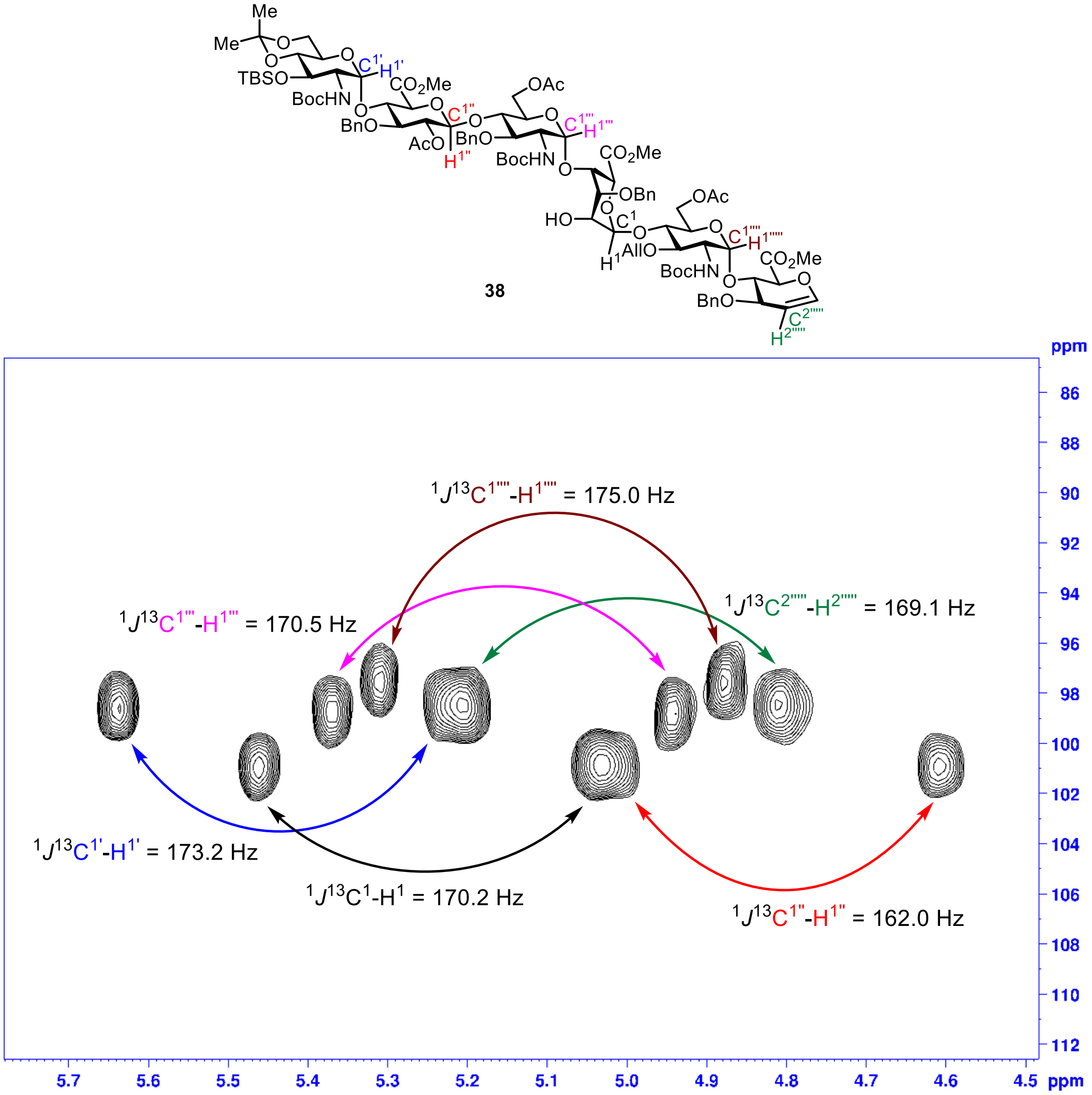

Figure S20. Un-decoupled HSQC Analysis to Determine Stereochemistry of **38**.

A $^1J_{^{13}C1-H1}$ value of 170.2 Hz suggested that the newly formed glycosidic bond is in axial position, which confirmed the formation of a 1,2-*trans*-α-glycosidic linkage.

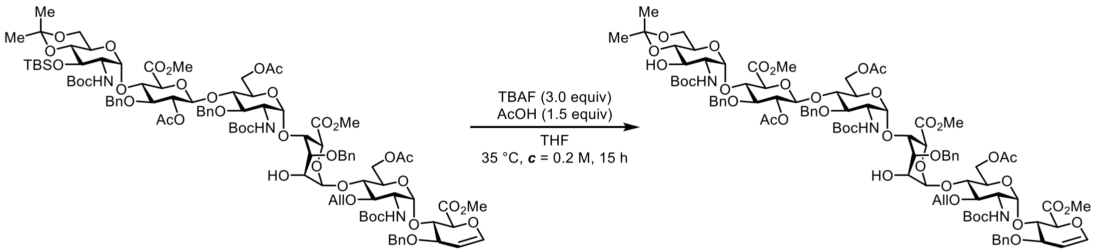

38 **S26** 88% yield

To a sealable 2-dram vial equipped with a stir bar were added compound **38** (300 mg, 0.149 mmol, 1.0 equiv) and THF (0.3 mL). To the mixture was then added a premixed solution of TBAF (1 M solution in THF, 0.446 mL, 0.446 mmol, 3.0 equiv) and AcOH (13 μL, 0.223 mmol, 1.5 equiv). The reaction mixture was stirred for 15 h at 35 °C until the starting material **38** was fully consumed (monitored by TLC). The reaction was then cooled down to room temperature and diluted with EtOAc (3 mL) before H_2O (2 mL) was added. The organic phase was separated from the aqueous one, which was further extracted with EtOAc (3 mL × 3). The combined organic phase was washed with brine (3 mL) and dried over Na_2SO_4. After concentration *in vacuo*, the residue was purified through a silica gel flash column (hexanes/EtOAc: from 100:1 to 2:3) to afford the desired product **S26** (249 mg, 88% yield) as white foam.

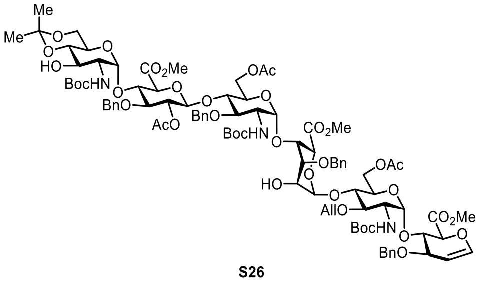

S26

Methyl 3-*O*-benzyl-4-*O*-[(2-*tert*-butoxycarbonylamino-4,6-*O*-isopropylidene-2-deoxy-α-D-glucopyranosyl)-(1→4)-(methyl 2-*O*-acetyl-3-*O*-benzyl-β-D-glucopyranosyluronate)-(1→4)-(6-*O*-acetyl-3-*O*-benzyl-2-*tert*-butoxycarbonylamino-2-deoxy-α-D-glucopyranosyl)-(1→4)-(methyl 3-*O*-benzyl-α-L-idopyranosyluronate)-(1→4)-(6-*O*-acetyl-3-*O*-allyl-2-*tert*-butoxycarbonylamino-2-deoxy-α-D-glucopyranosyl)]-D-glucuronal (**S26**): $[\alpha]_D^{21}$ +30.7 (acetone, c = 1.0); IR ν_{max} (neat)/cm^{-1}: 3436 (w), 2929 (w), 2048 (w), 1745 (m), 1650 (w), 1368

(m), 1135 (s); ^{1}H NMR (400 MHz, acetone-d_6) δ 7.49 – 7.12 (m, 20H), 6.58 (d, *J* = 6.3 Hz, 1H), 6.27 (d, *J* = 9.4 Hz, 1H), 5.88 (d, *J* = 9.0 Hz, 1H), 5.86 – 5.69 (m, 2H), 5.38 (d, *J* = 3.8 Hz, 1H), 5.24 (d, *J* = 4.9 Hz, 1H), 5.18 – 5.10 (m, 2H), 5.08 (d, *J* = 3.7 Hz, 1H), 5.05 (s, 1H), 5.04 – 4.94 (m, 4H), 4.89 – 4.79 (m, 4H), 4.75 (d, *J* = 10.8 Hz, 1H), 4.69 (d, *J* = 10.7 Hz, 1H), 4.65 (d, *J* = 6.2 Hz, 1H), 4.62 – 4.56 (m, 1H), 4.55 – 4.48 (m, 3H), 4.47 – 4.39 (m, 2H), 4.35 – 4.20 (m, 3H), 4.18 – 4.03 (m, 5H), 4.03 – 3.88 (m, 4H), 3.86 – 3.81 (m, 2H), 3.81 – 3.71 (m, 8H), 3.70 – 3.65 (m, 2H), 3.62 (s, 3H), 3.61 – 3.54 (m, 4H), 3.53 (s, 3H), 3.29 – 3.27 (m, 1H), 2.085 (s, 3H), 2.082 (s, 3H), 2.05 (s, 3H), 1.47 (s, 3H), 1.40 (s, 9H), 1.35 (s, 9H), 1.31 (s, 3H), 1.26 (s, 9H); ^{13}C NMR (100 MHz, acetone-d_6) δ 171.1, 170.9, 170.9, 169.9, 169.2, 168.5, 156.7, 156.4, 156.2, 145.8, 140.4, 139.6, 139.4, 138.9, 136.7, 129.14 (2C), 129.09 (2C), 129.05 (2C), 129.0 (2C), 128.9 (2C), 128.8 (2C), 128.7 (2C), 128.61 (2C), 128.57, 128.32, 128.26, 127.7, 115.8, 101.8, 101.7, 100.0, 99.7, 99.5, 99.2, 98.4, 83.1, 79.3, 79.2, 79.1, 78.9 (two peaks overlapped, 2C), 78.2, 77.5, 75.7, 75.5, 75.4 (two peaks overlapped, 2C), 75.3, 74.4, 74.2, 74.14, 74.11, 73.7, 73.6, 71.6, 71.5, 71.4, 71.0, 70.5, 70.2, 70.1, 68.6, 65.2, 63.5, 63.1, 62.4, 56.6, 55.3, 55.0, 52.9, 52.5, 52.2, 29.7, 28.7 (3C), 28.64 (3C), 28.61 (3C), 20.91, 20.87, 20.85, 19.4; HRMS: m/z (ESI) calcd for $C_{94}H_{126}N_3O_{38}^+$, $[M + H]^+$, 1904.8014, found 1904.8045. $^1J_{^{13}C1-H1}$ = 175.2 Hz, 168.3 Hz, 170.8 Hz, 172.8 Hz, 160.0 Hz.

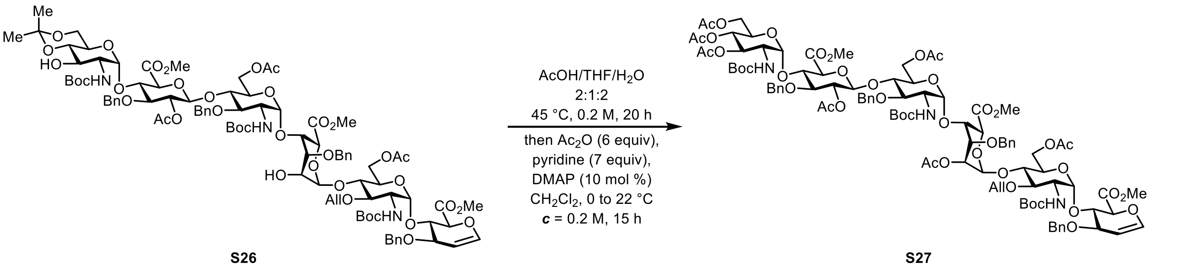

S26

S27
96% yield over two steps

To a 2-dram vial equipped with a stir bar were added **S26** (249 mg, 0.13 mmol, 1.0 equiv) and THF (0.13 mL). AcOH (0.26 mL) and H_2O (0.26 mL) were then added sequentially to the mixture. The reaction mixture was stirred at 45 °C for 20 h until the starting material was fully consumed (monitored by TLC). The reaction was then cooled down to room temperature. After

concentration *in vacuo*, the residue was azeotropically dried with anhydrous toluene (3 mL × 5) and directly used in the next step without further purification.

To a flame-dried sealable 2-dram vial equipped with a stir bar were added crude product from the previous step (0.13 mmol, 1.0 equiv) and DMAP (1.6 mg, 0.013 mmol, 10 mol %). After the vial was evacuated and backfilled with N_2, anhydrous CH_2Cl_2 (0.65 mL) was added. The solution was then cooled to 0 °C before pyridine (73 μL, 0.91 mmol, 7.0 equiv) and Ac_2O (74 μL, 0.78 mmol, 6.0 equiv) were added dropwise sequentially. The reaction mixture was gradually warmed to room temperature and stirred for 15 h, with progress monitored by TLC until completion. The reaction mixture was then diluted with CH_2Cl_2 (2 mL) and quenched with saturated aqueous NH_4Cl solution (1.5 mL). The organic phase was separated from the aqueous one, which was further extracted with CH_2Cl_2 (3 mL × 3). The combined organic phase was dried over Na_2SO_4. After concentration *in vacuo*, the residue was purified through a silica gel flash column (hexanes/EtOAc: from 100:1 to 2:3) to afford the desired product **S27** (254 mg, 96% yield over two steps) as white foam.

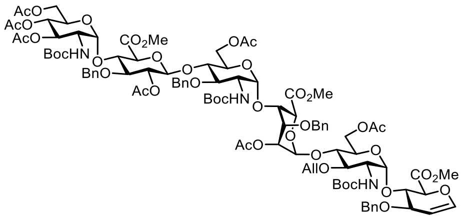

S27

Methyl 3-*O*-benzyl-4-*O*-[(3,4,6-tri-*O*-acetyl-2-*tert*-butoxycarbonylamino-2-deoxy-α-D-glucopyranosyl)-(1→4)-(methyl 2-*O*-acetyl-3-*O*-benzyl-β-D-glucopyranosyluronate)-(1→4)-(6-*O*-acetyl-3-*O*-benzyl-2-*tert*-butoxycarbonylamino-2-deoxy-α-D-glucopyranosyl)-(1→4)-(methyl 2-*O*-acetyl-3-*O*-benzyl-α-L-idopyranosyluronate)-(1→4)-(6-*O*-acetyl-3-*O*-allyl-2-*tert*-butoxycarbonylamino-2-deoxy-α-D-glucopyranosyl)]-D-glucuronal (**S27**): $[\alpha]_D^{22}$ +31.8 (acetone, c = 1.0); IR ν_{max} (neat)/cm^{-1}: 3446 (w), 2928 (w), 2025 (m), 1747 (s), 1651 (w), 1367 (m), 1230 (s); ^{1}H NMR (400 MHz, acetone-d_6) δ 7.55 – 7.15 (m, 20H), 6.58 (d, J = 6.3 Hz, 1H), 5.93 – 5.74 (m, 4H), 5.51 (d, J = 3.7 Hz, 1H), 5.20 (d, J = 3.7 Hz, 1H), 5.17 – 5.06 (m, 5H), 5.04 – 4.94 (m, 6H), 4.93 (t, J = 4.3 Hz, 1H), 4.85 (d, J = 7.9 Hz, 1H), 4.81 (d, J = 10.7 Hz, 1H), 4.78

– 4.69 (m, 3H), 4.60 (d, J = 11.1 Hz, 1H), 4.58 – 4.45 (m, 4H), 4.43 – 4.37 (m, 1H), 4.32 – 4.18 (m, 6H), 4.18 – 4.10 (m, 3H), 4.10 – 4.03 (m, 2H), 4.03 – 3.91 (m, 4H), 3.89 (d, J = 8.8 Hz, 1H), 3.87 – 3.79 (m, 2H), 3.79 – 3.70 (m, 5H), 3.66 – 3.56 (m, 4H), 3.56 – 3.49 (m, 4H), 2.10 (s, 3H), 2.09 (s, 3H), 2.08 (s, 3H), 2.04 (s, 3H), 2.02 (s, 3H), 1.99 (s, 3H), 1.95 (s, 3H), 1.39 (s, 9H), 1.34 (s, 9H), 1.29 (s, 9H); ^{13}C NMR (100 MHz, acetone-d_6) δ 170.9, 170.8 (three peaks overlapped, 3C), 170.6, 170.2, 170.0, 169.9, 169.3, 168.6, 156.22, 156.19, 156.0, 145.8, 140.5, 139.4, 139.0, 138.7, 136.5, 129.23 (2C), 129.17 (2C), 129.1 (2C), 128.9 (2C), 128.73 (two peaks overlapped, 4C), 128.70 (2C), 128.6 (two peaks overlapped, 3C), 128.5, 128.3, 127.8, 116.0, 101.8, 99.6, 99.5, 99.0, 98.4, 98.2, 82.7, 79.7 (three peaks overlapped, 3C), 79.4, 79.2, 79.1, 77.1, 75.7, 75.5, 75.3, 75.2, 75.0, 74.4, 74.2, 74.1, 73.8, 73.7, 72.9, 72.0, 70.8, 70.6, 70.4, 70.1, 69.7, 69.5, 69.0, 68.9, 63.1, 63.0, 62.4, 55.4, 54.9, 54.0, 53.0, 52.5, 52.2, 28.7 (3C), 28.6 (3C), 28.5 (3C), 21.2, 21.0, 20.9, 20.9, 20.7 (two peaks overlapped, 2C), 20.6; HRMS: m/z (ESI) calcd for $C_{99}H_{130}N_3O_{42}^+$, $[M + H]^+$, 2032.8123, found 2032.8196. $^1J_{^{13}C1-H1}$ = 174.3 Hz, 171.8 Hz, 170.9 Hz, 170.9 Hz, 161.8 Hz.

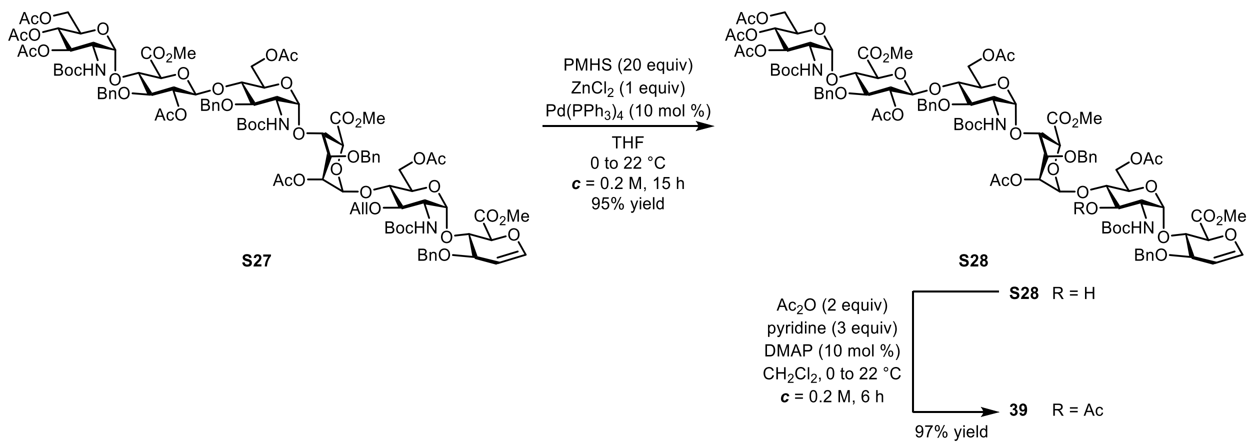

To a flame-dried sealable 2-dram vial equipped with a stir bar were added compound **S27** (254 mg, 0.125 mmol, 1.0 equiv), $Pd(PPh_3)_4$ (14.4 mg, 0.0125 mmol, 10 mol %), and polymethylhydrosiloxane (150 mg, 2.5 mmol, 20 equiv).[9] After the vial was evacuated and backfilled with N_2 three times, anhydrous THF (0.62 mL) was added. The solution was then cooled to 0 °C before $ZnCl_2$ (1 M solution in Et_2O, 0.125 mL, 0.125 mmol, 1.0 equiv) was added

dropwise. The reaction mixture was gradually warmed to room temperature and stirred for 15 h until the starting material **S27** was fully consumed (monitored by TLC). The reaction mixture was then diluted with EtOAc (3 mL) and quenched with saturated aqueous $NaHCO_3$ solution (2 mL). The organic phase was separated from the aqueous one, which was further extracted with EtOAc (3 mL × 3). The combined organic phase was dried over Na_2SO_4. After concentration *in vacuo*, the residue was purified through a silica gel flash column (hexanes/acetone: from 100:1 to 3:2) to afford the desired product **S28** (237 mg, 95% yield) as white foam.

To a flame-dried sealable 2-dram vial equipped with a stir bar were added **S28** (237 mg, 0.119 mmol, 1.0 equiv) from the previous step and DMAP (1.5 mg, 0.012 mmol, 10 mol %). After the vial was evacuated and backfilled with N_2, anhydrous CH_2Cl_2 (0.6 mL) was added. The solution was then cooled to 0 °C before pyridine (29 μL, 0.357 mmol, 3.0 equiv) and Ac_2O (23 μL, 0.238 mmol, 2.0 equiv) were added dropwise sequentially. The reaction mixture was gradually warmed to room temperature and stirred for 6 h, with progress monitored by TLC until completion. The reaction mixture was then diluted with CH_2Cl_2 (2 mL) and quenched with saturated aqueous NH_4Cl solution (1 mL). The organic phase was separated from the aqueous one, which was further extracted with CH_2Cl_2 (2 mL × 3). The combined organic phase was dried over Na_2SO_4. After concentration *in vacuo*, the residue was purified through a silica gel flash column (hexanes/EtOAc: from 100:1 to 1:1) to afford the desired product **39** (235 mg, 97% yield) as white foam.

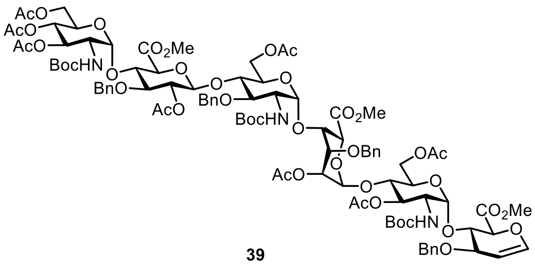

39

Methyl 3-*O*-benzyl-4-*O*-[(3,4,6-tri-*O*-acetyl-2-*tert*-butoxycarbonylamino-2-deoxy-α-D-glucopyranosyl)-(1→4)-(methyl 2-*O*-acetyl-3-*O*-benzyl-β-D-glucopyranosyluronate)-(1→4)-(6-*O*-acetyl-3-*O*-benzyl-2-*tert*-butoxycarbonylamino-2-deoxy-α-D-glucopyranosyl)-(1→4)-

(methyl 2-*O*-acetyl-3-*O*-benzyl-α-L-idopyranosyluronate)-(1→4)-(3,6-di-*O*-acetyl-2-*tert*-butoxycarbonylamino-2-deoxy-α-D-glucopyranosyl)]-D-glucuronal (**39**): $[\alpha]_D^{22}$ +21.7 (acetone, *c* = 1.0); IR ν_{max} (neat)/cm^{-1}: 3629 (w), 3443 (w), 2927 (w), 2359 (m), 1746 (s), 1717 (m), 1652 (w); ^{1}H NMR (400 MHz, acetone-d_6) δ 7.42 – 7.18 (m, 20H), 6.56 (d, *J* = 6.3 Hz, 1H), 5.95 – 5.75 (m, 2H), 5.51 (d, *J* = 3.7 Hz, 1H), 5.42 (d, *J* = 9.9 Hz, 1H), 5.24 (d, *J* = 3.8 Hz, 1H), 5.17 (d, *J* = 3.9 Hz, 1H), 5.13 (d, *J* = 9.6 Hz, 1H), 5.10 – 5.04 (m, 3H), 5.04 – 4.98 (m, 3H), 4.96 (dd, *J* = 3.4, 1.2 Hz, 1H), 4.89 – 4.83 (m, 2H), 4.83 – 4.78 (m, 2H), 4.77 – 4.68 (m, 3H), 4.63 – 4.55 (m, 2H), 4.55 – 4.48 (m, 2H), 4.48 – 4.40 (m, 2H), 4.31 – 4.22 (m, 3H), 4.22 – 4.15 (m, 2H), 4.15 – 4.09 (m, 2H), 4.08 – 4.01 (m, 2H), 4.01 – 3.96 (m, 2H), 3.96 – 3.91 (m, 1H), 3.92 – 3.87 (m, 2H), 3.87 – 3.82 (m, 2H), 3.82 – 3.76 (m, 4H), 3.76 – 3.70 (m, 1H), 3.66 – 3.55 (m, 4H), 3.52 (s, 3H), 2.11 (s, 3H), 2.08 (s, 6H), 2.04 (s, 3H), 2.02 (s, 3H), 1.99 (s, 3H), 1.97 (s, 3H), 1.95 (s, 3H), 1.39 (s, 9H), 1.34 (s, 9H), 1.28 (s, 9H); ^{13}C NMR (100 MHz, acetone-d_6) δ 171.0, 170.93, 170.85, 170.8 (two peaks overlapped, 2C), 170.6, 170.1, 170.0, 169.9, 169.3, 168.5, 156.1, 156.0 (two peaks overlapped, 2C), 145.8, 140.4, 139.3, 139.0, 138.7, 129.23 (2C), 129.18 (2C), 129.1 (2C), 128.9 (2C), 128.71 (two peaks overlapped, 4C), 128.65 (two peaks overlapped, 3C), 128.4 (two peaks overlapped, 3C), 128.3, 127.8, 101.8, 100.3, 99.6, 99.1, 98.4, 98.0, 82.7, 79.7, 79.6, 79.5, 79.32, 79.30, 78.0, 75.7, 75.5, 75.3, 75.1, 75.0, 74.4, 74.3, 74.2, 73.7, 72.7, 72.6, 72.0, 70.9, 70.6, 70.4, 70.2, 70.0, 69.5, 69.1, 69.0, 63.1, 63.0, 62.4, 54.9, 54.4, 54.0, 52.9, 52.7, 52.2, 28.6 (3C), 28.51 (3C), 28.49 (3C), 21.24, 21.15, 21.0, 20.84, 20.80, 20.7 (two peaks overlapped, 2C), 20.6; HRMS: m/z (ESI) calcd for $C_{98}H_{128}N_3O_{43}^{+}$, [M + H]$^{+}$, 2034.7916, found 2034.7866. $^{1}J_{^{13}\mathrm{C1-H1}}$ = 174.8 Hz, 170.7 Hz, 170.7 Hz, 174.6 Hz, 161.3 Hz.

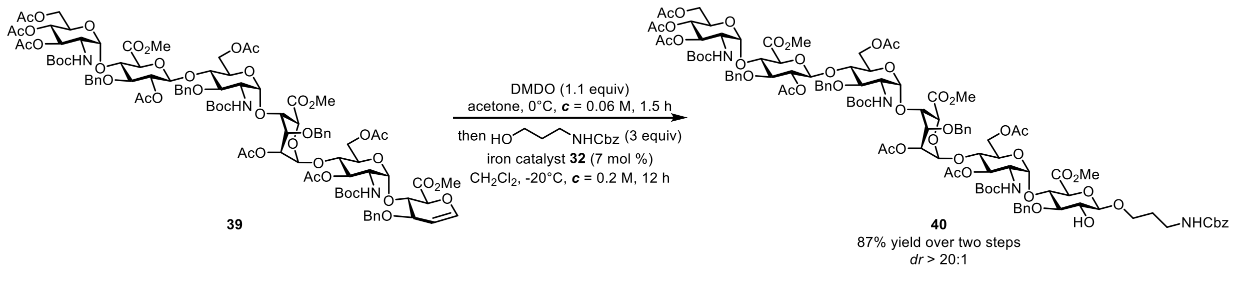

To a flame-dried sealable 2-dram vial equipped with a stir bar were added compound **39** (235 mg, 0.115 mmol, 1.0 equiv) and anhydrous CH_2Cl_2 (0.58 mL). The solution was cooled to 0 °C before DMDO (0.06 M solution in acetone, 2.11 mL, 0.127 mmol, 1.1 equiv)[16] was added. The reaction was kept at 0 °C and stirred for 1.5 h. The reaction mixture was then concentrated *in vacuo* with a 10 °C water bath. The residue was then re-dissolved in anhydrous CH_2Cl_2 (1 mL) and dried over anhydrous Na_2SO_4. The solution was transferred to a separate flame-dried sealable 2-dram vial and concentrated once more *in vacuo* at 10 °C. The residue was further dried azeotropically with anhydrous toluene (2 mL × 3), yielding the corresponding glycal epoxide as white foam, which was used directly in the next step without further purification.

To the same flame-dried sealable 2-dram vial (vial **A**) containing the glycal epoxide (0.115 mmol, 1.0 equiv) were added a stir bar, benzyl (3-hydroxypropyl)carbamate (72 mg, 0.345 mmol, 3.0 equiv), and freshly activated 5 Å molecular sieves, powder (*ca.* 100 mg). After vial **A** was evacuated and backfilled with N_2 three times, anhydrous CH_2Cl_2 (0.38 mL) was added and the vial was cooled to –20 °C. To a separate flame-dried sealable 2-dram vial (vial **B**) were added the iron porphyrin triflate catalyst **32** (0.0081 mmol, 0.07 equiv) and freshly activated 5 Å molecular sieves, powder (*ca.* 50 mg). After vial **B** was evacuated and backfilled with N_2 three times, anhydrous CH_2Cl_2 (0.2 mL) was added. Vial **B** was stirred for 5 min at room temperature before the mixture was transferred to vial **A** dropwise via a syringe. The reaction was then stirred at –20 °C for 12 h until the epoxide was fully consumed (monitored by TLC). The reaction mixture was quenched with imidazole (1.0 M solution in CH_2Cl_2, 58 μL, 0.058 mmol, 0.5 equiv) and filtered through a short pad of silica (eluted with EtOAc). The filtrate was concentrated *in vacuo*, and the residue was purified through a silica gel flash column (hexanes/acetone: from 100:1 to 1:1) to afford the desired product **40** as white foam (226 mg, 87 % yield over two steps).

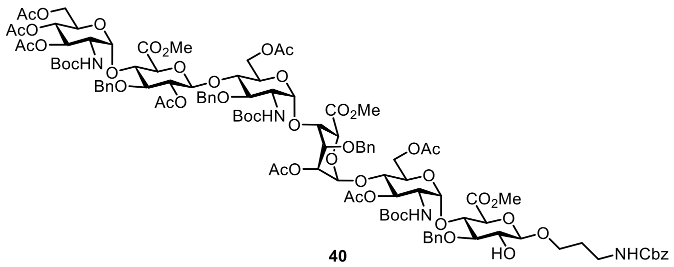

40

Methyl (3,4,6-tri-*O*-acetyl-2-*tert*-butoxycarbonylamino-2-deoxy-α-D-glucopyranosyl)-(1→4)-(methyl 2-*O*-acetyl-3-*O*-benzyl-β-D-glucopyranosyluronate)-(1→4)-(6-*O*-acetyl-3-*O*-benzyl-2-*tert*-butoxycarbonylamino-2-deoxy-α-D-glucopyranosyl)-(1→4)-(methyl 2-*O*-acetyl-3-*O*-benzyl-α-L-idopyranosyluronate)-(1→4)-(3,6-di-*O*-acetyl-2-*tert*-butoxycarbonylamino-2-deoxy-α-D-glucopyranosyl)-(1→4)-(*N*-benzyloxycarbonyl-3-aminopropyl 3-*O*-benzyl-β-D-glucopyranosid)uronate (**40**): $[\alpha]_D^{21}$ +21.4 (acetone, *c* = 1.0); IR ν_{max} (neat)/cm^{-1}: 3629 (w), 3443 (w), 2931 (w), 2359 (m), 1746 (s), 1718 (s), 1653 (w); ^{1}H NMR (400 MHz, acetone-d_6) δ 7.46 – 7.18 (m, 25H), 6.35 (t, *J* = 6.2 Hz, 1H), 5.90 – 5.81 (m, 2H), 5.74 (d, *J* = 10.0 Hz, 1H), 5.55 – 5.44 (m, 2H), 5.15 (d, *J* = 4.1 Hz, 1H), 5.14 – 5.07 (m, 4H), 5.07 – 5.02 (m, 3H), 5.03 – 4.95 (m, 2H), 4.85 (d, *J* = 7.9 Hz, 1H), 4.83 – 4.78 (m, 4H), 4.78 – 4.73 (m, 3H), 4.70 (d, *J* = 11.6 Hz, 1H), 4.60 (d, *J* = 11.1 Hz, 1H), 4.52 (d, *J* = 11.7 Hz, 1H), 4.41 (d, *J* = 8.0 Hz, 1H), 4.32 (dd, *J* = 12.2, 2.0 Hz, 1H), 4.29 – 4.20 (m, 4H), 4.18 (d, *J* = 8.6 Hz, 1H), 4.16 – 4.06 (m, 4H), 3.99 – 3.94 (m, 2H), 3.94 – 3.89 (m, 3H), 3.89 – 3.87 (m, 1H), 3.86 – 3.83 (m, 1H), 3.82 (s, 3H), 3.80 – 3.77 (m, 1H), 3.77 – 3.70 (m, 2H), 3.63 – 3.54 (m, 9H), 3.53 – 3.42 (m, 2H), 3.38 – 3.28 (m, 1H), 3.28 – 3.17 (m, 1H), 2.11 (s, 3H), 2.11 (s, 3H), 2.10 (s, 3H), 2.03 (s, 3H), 2.02 (s, 3H), 1.99 (s, 3H), 1.97 (s, 3H), 1.95 (s, 3H), 1.84 – 1.68 (m, 2H), 1.34 (s, 18H), 1.28 (s, 9H); ^{13}C NMR (100 MHz, acetone-d_6) δ 171.0 (two peaks overlapped, 2C), 170.9, 170.8 (two peaks overlapped, 2C), 170.6, 170.08, 170.06, 170.0, 169.9, 169.3, 157.4, 156.2, 156.0 (two peaks overlapped, 2C), 140.4, 139.5, 139.0, 138.7, 138.5, 129.2 (four peaks overlapped, 8C), 129.1 (2C), 128.9 (2C), 128.7 (three peaks overlapped, 5C), 128.7 (2C), 128.6, 128.54 (2C), 128.52, 128.4, 127.8, 104.2, 101.9, 100.1, 98.4, 98.3, 98.2, 84.53, 84.50, 82.7, 79.7, 79.5, 79.5, 79.3, 77.4, 75.7 (two peaks overlapped, 2C), 75.6, 75.5, 75.4, 75.3, 75.1, 75.0, 74.8, 74.2, 74.0, 72.8, 72.6, 72.0, 71.0, 70.6, 70.3, 70.0, 69.5, 69.0, 67.8, 66.5, 63.0, 62.4 (two peaks overlapped, 2C), 54.9, 54.2, 54.0, 53.0, 52.8, 52.7, 38.4, 30.6, 28.6 (3C), 28.54 (3C), 28.52 (3C), 21.24, 21.17, 21.0, 20.9 (two peaks overlapped, 2C), 20.7 (two peaks overlapped, 2C), 20.6; HRMS: m/z (ESI) calcd for $C_{109}H_{143}N_4O_{47}^+$, [M + H]$^+$, 2259.8917, found 2259.8982. $^1J_{^{13}C1–H1}$ = 175.9 Hz, 175.9 Hz, 169.8 Hz, 174.0 Hz, 162.0 Hz, 161.0 Hz. $^3J_{H1–H2}$ = 8.0 Hz.

The stereochemistry of newly formed anomeric center (C1) of **40** was determined by measuring $^1J_{^{13}C1–H1}$ (161.0 Hz) through un-decoupled HSQC experiments.[11-12] The C2 stereochemistry was determined by measuring $^3J_{H1–H2}$ (8.0 Hz).

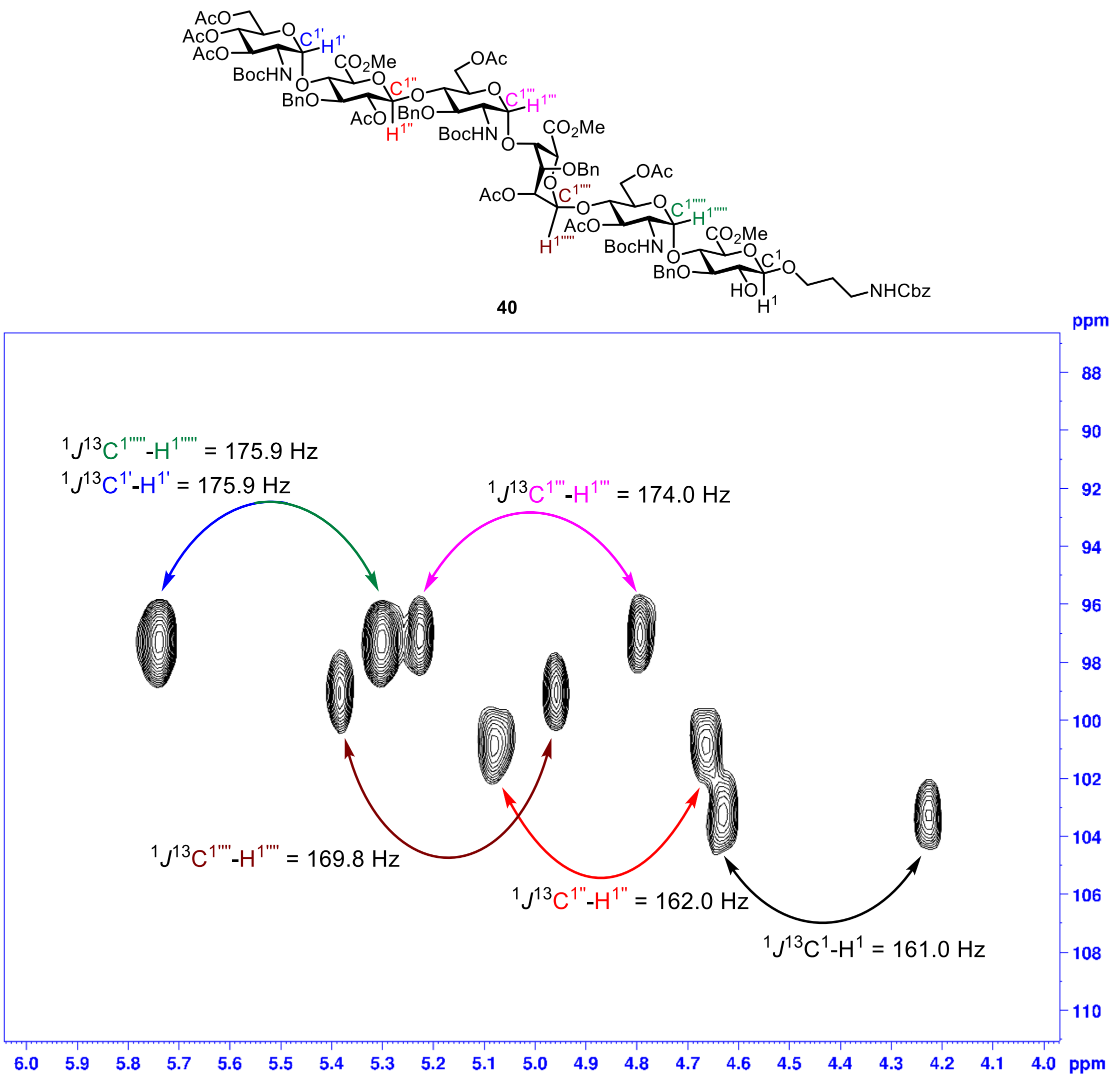

Figure S21. Un-decoupled HSQC Analysis to Determine Stereochemistry of **40**.

A $^1J^{13}_{C1-H1}$ value of 161.0 Hz suggested that the newly formed glycosidic bond is in equatorial position. The $^3J_{H1-H2}$ value of 8.0 Hz suggested that the H2 is in axial position.

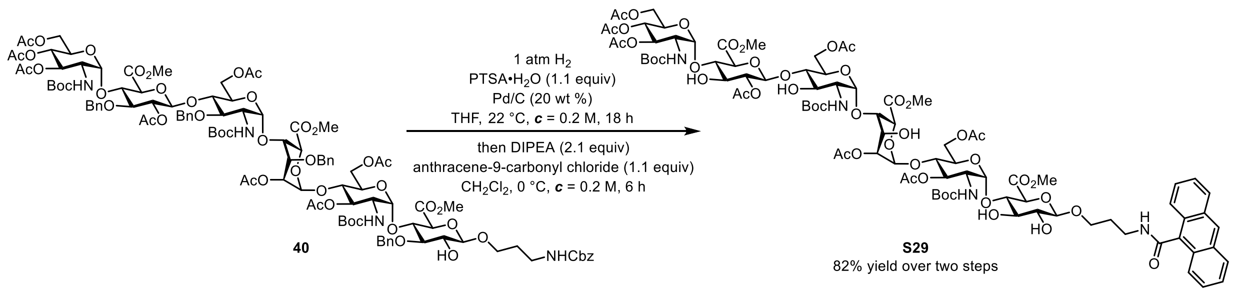

To a sealable 2-dram vial equipped with a stir bar was added compound **40** (226 mg, 0.1 mmol, 1.0 equiv). After the vial was evacuated and backfilled with N_2 three times, PTSA·H_2O (20.9 mg, 0.11 mmol, 1.1 equiv) and Pd/C (45.2 mg, 20 wt %) were added sequentially. The vial was again evacuated and backfilled with N_2 three times, followed by addition of THF (0.5 mL). The vial was then evacuated and backfilled with H_2 three times. The mixture was stirred for 18 h at room temperature, with progress monitored by TLC until completion. The reaction mixture was then filtered through cotton (eluted with MeOH), and the filtrate was concentrated *in vacuo* to afford a white solid, which was used directly in the next step without further purification.

To a flame-dried sealable 2-dram vial equipped with a stir bar was added the crude product from the previous step (0.1 mmol, 1.0 equiv). After the vial was evacuated and backfilled with N_2, CH_2Cl_2 (0.4 mL) was added. The solution was then cooled to 0 °C before DIPEA (37 μL, 0.21 mmol, 2.1 equiv) was added dropwise. The reaction mixture was stirred at 0 °C for 10 min and a solution of freshly prepared anthracene-9-carbonyl chloride (26.5 mg, 0.11 mmol, 1.1 equiv) in anhydrous CH_2Cl_2 (0.1 mL) was added to the reaction mixture dropwise. The reaction mixture was stirred at the same temperature for 6 h until the starting material was fully consumed (monitored by TLC). The reaction mixture was then diluted with CH_2Cl_2 (2 mL) and quenched with MeOH (0.1 mL). The mixture was concentrated *in vacuo*, and the residue was purified through a silica gel flash column (hexanes/acetone: from 100:1 to 2:1) to afford the desired product **S29** (162 mg, 82% yield over two steps) as a white solid.

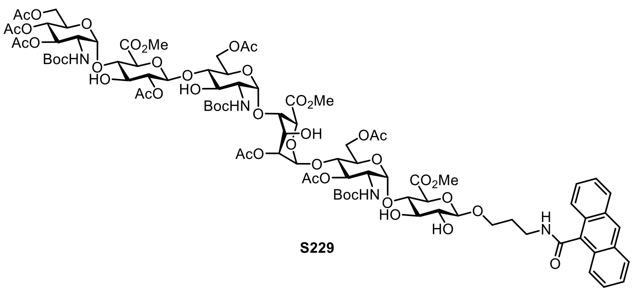

S229

Methyl (3,4,6-tri-*O*-acetyl-2-*tert*-butoxycarbonylamino-2-deoxy-α-D-glucopyranosyl)-(1→4)-(methyl 2-*O*-acetyl-β-D-glucopyranosyluronate)-(1→4)-(6-*O*-acetyl-2-*tert*-butoxycarbonylamino-2-deoxy-α-D-glucopyranosyl)-(1→4)-(methyl 2-*O*-acetyl-α-L-idopyranosyluronate)-(1→4)-(3,6-di-*O*-acetyl-2-*tert*-butoxycarbonylamino-2-deoxy-α-D-glucopyranosyl)-(1→4)-[3-(anthracene-9-carbonylamino)propyl β-D-glucopyranosid]uronate (**S29**): $[\alpha]^{22}_{D}$ +24.0 (acetone, c = 0.3); IR ν_{max} (neat)/cm^{-1}: 3628 (m), 3587 (w), 2922 (w), 2360 (s), 2342 (m), 1746 (s), 1684 (m); ^{1}H NMR (400 MHz, acetone-d_6) δ 8.59 (s, 1H), 8.18 – 7.97 (m, 4H), 7.81 (t, J = 6.0 Hz, 1H), 7.62 – 7.46 (m, 4H), 5.90 (d, J = 9.9 Hz, 1H), 5.83 (d, J = 9.9 Hz, 1H), 5.55 (d, J = 5.5 Hz, 1H), 5.48 (d, J = 3.5 Hz, 1H), 5.31 (d, J = 3.6 Hz, 1H), 5.22 – 5.09 (m, 2H), 5.08 – 4.95 (m, 4H), 4.88 (t, J = 8.5 Hz, 1H), 4.80 (d, J = 8.1 Hz, 1H), 4.77 (d, J = 4.2 Hz, 1H), 4.73 (t, J = 5.1 Hz, 1H), 4.70 – 4.57 (m, 1H), 4.50 (d, J = 7.7 Hz, 1H), 4.47 – 4.40 (m, 1H), 4.37 – 4.29 (m, 2H), 4.21 (dd, J = 12.4, 3.0 Hz, 1H), 4.17 – 4.08 (m, 5H), 4.08 – 4.02 (m, 3H), 4.02 – 3.94 (m, 2H), 3.93 – 3.85 (m, 3H), 3.85 – 3.79 (m, 8H), 3.79 – 3.76 (m, 3H), 3.76 – 3.73 (m, 4H), 3.73 – 3.68 (m, 5H), 3.65 – 3.54 (m, 3H), 3.37 – 3.26 (m, 1H), 2.15 – 2.07 (m, 14H), 2.03 (s, 3H), 1.99 (s, 3H), 1.94 (s, 3H), 1.93 (s, 3H), 1.39 (s, 9H), 1.38 (s, 9H), 1.37 (s, 9H); ^{13}C NMR (100 MHz, acetone-d_6) δ 171.02, 170.96, 170.84, 170.78, 170.7, 170.4, 170.20, 170.16, 169.9, 169.8, 169.6, 169.1, 156.4, 156.18, 156.16, 134.5, 132.2 (2C), 129.3 (2C), 128.8, 128.2 (2C), 127.2 (2C), 126.41 (2C), 126.36 (2C), 104.2, 101.9, 100.3, 99.6, 99.3, 98.5, 81.9, 79.42, 79.37, 79.2, 79.1, 78.0, 77.4, 76.7, 76.6, 75.7, 75.5, 74.9, 74.8, 74.7, 74.3, 72.7, 72.3, 72.1, 71.0, 70.6, 70.3, 69.6, 69.5, 69.2, 68.6, 68.2, 63.2, 62.4, 55.6, 54.6, 54.3, 53.2, 52.9, 52.4, 37.5, 37.3, 28.6 (3C), 28.52 (3C), 28.50 (3C), 21.2 (two peaks overlapped, 2C), 20.9, 20.8, 20.8, 20.7, 20.7, 20.6; HRMS: m/z (ESI) calcd for $C_{88}H_{121}N_4O_{46}^{+}$, $[M + H]^{+}$, 1969.7246, found 1969.7203. $^{1}J_{^{13}C1-H1}$ = 171.9 Hz, 174.1 Hz, 170.6 Hz, 172.8 Hz, 162.0 Hz, 160.7 Hz.

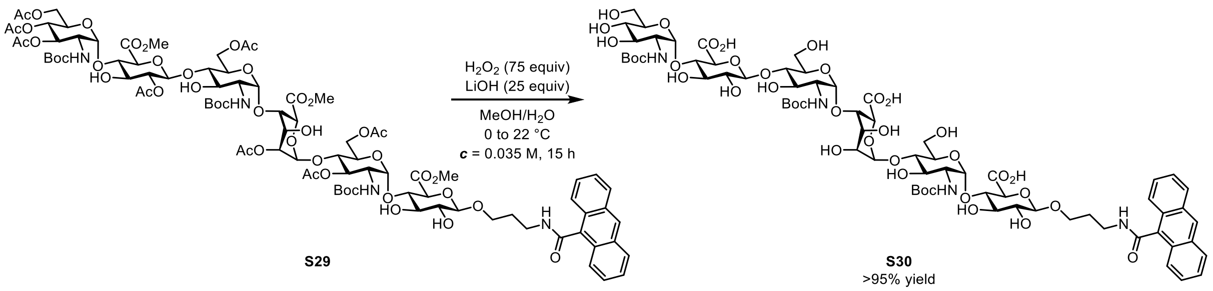

To a sealable 2-dram vial equipped with a stir bar were added compound **S29** (162 mg, 0.082 mmol, 1.0 equiv) and MeOH (1.0 mL). The solution was cooled to 0 °C, and a precooled, premixed solution of H_2O_2 (30 wt % in H_2O, 0.65 mL, 6.17 mmol, 75 equiv) and LiOH (3.0 M in H_2O, 0.69 mL, 2.06 mmol, 25 equiv) was added dropwise at the same temperature. The reaction mixture was subsequently allowed to warm to room temperature and stirred for 15 h, with progress monitored by TLC until completion. The reaction mixture was diluted with MeOH (3 mL), and Amberlite® IRC 120 H resin (1.0 g) was added at 0 °C to neutralize the reaction. The suspension was filtered through a piece of cotton (rinsed with MeOH). $NaHSO_3$ solid was then added portion-wise to the solution to decompose residual H_2O_2, and the progress was monitored using MQuant® Peroxide Test strips. Upon completion, the mixture was filtered through a piece of cotton (rinsed with MeOH), and the filtrate was concentrated *in vacuo* to afford the desired product **S30** (130 mg, quantitative yield) as a white solid.

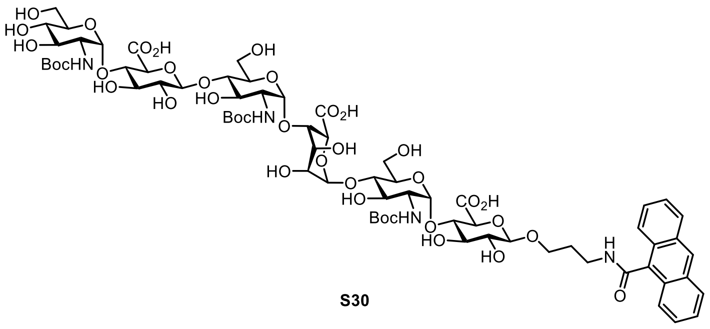

S30

3-(Anthracene-9-carbonylamino)propyl (2-*tert*-butoxycarbonylamino-2-deoxy-α-D-glucopyranosyl)-(1→4)-(β-D-glucopyranosyluronic acid)-(1→4)-(2-*tert*-butoxycarbonylamino-2-deoxy-α-D-glucopyranosyl)-(1→4)-(α-L-idopyranosyluronic acid)-(1→4)-(2-*tert*-butoxycarbonylamino-2-deoxy-α-D-glucopyranosyl)-(1→4)-β-D-

glucopyranosiduronic acid (**S30**): ^{1}H NMR (400 MHz, CD_3OD) δ 8.58 (s, 1H), 8.08 (d, *J* = 8.4 Hz, 2H), 8.02 (d, *J* = 8.6 Hz, 2H), 7.69 – 7.37 (m, 4H), 5.37 (d, *J* = 1.6 Hz, 1H), 5.32 (d, *J* = 3.1 Hz, 1H), 5.08 (d, *J* = 3.4 Hz, 1H), 5.06 – 5.00 (m, 2H), 4.53 (d, *J* = 7.9 Hz, 1H), 4.38 (d, *J* = 7.8 Hz, 1H), 4.13 – 4.04 (m, 1H), 4.04 – 3.96 (m, 2H), 3.96 – 3.86 (m, 3H), 3.84 – 3.70 (m, 12H), 3.71 – 3.61 (m, 7H), 3.60 – 3.49 (m, 6H), 3.47 – 3.37 (m, 1H), 3.30 – 3.22 (m, 1H), 2.14 – 2.03 (m, 2H), 1.45 (s, 9H), 1.45 (s, 18H); ^{13}C NMR (100 MHz, CD_3OD) δ 173.3, 172.4, 172.2, 172.1, 158.5, 158.4 (two peaks overlapped, 2C), 133.2, 132.6 (2C), 129.6 (2C), 129.3, 129.2 (2C), 127.8 (2C), 126.6 (2C), 126.0 (2C), 104.5, 104.1, 103.3, 100.0, 99.8, 97.5, 80.5, 80.42, 80.36, 80.2, 79.22, 79.18, 78.9, 78.3, 77.5, 76.3, 76.0, 74.9, 74.8, 74.2, 73.2, 73.1, 72.6, 71.7 (two peaks overlapped, 2C), 71.60, 71.56, 71.3, 70.0, 69.6, 68.6, 62.1, 61.3, 61.2, 57.0, 56.8, 56.2, 38.2, 30.5, 28.8 (three peaks overlapped, 9 C); HRMS: m/z (ESI) calcd for $C_{69}H_{99}N_4O_{38}^+$, $[M + H]^+$, 1591.5932, found 1591.5953. $^{1}J_{^{13}C1–H1}$ = 171.8 Hz, 172.6 Hz, 170.5 Hz, 170.6 Hz, 163.0 Hz, 159.9 Hz.

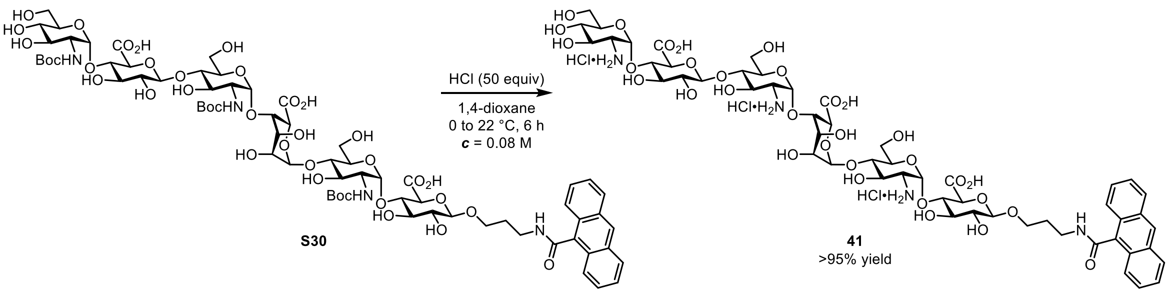

To a sealable 2-dram vial equipped with a stir bar was added compound **S30** (130 mg, 0.082 mmol, 1.0 equiv). After the vial was cooled down to 0 °C, a solution of HCl in 1,4-dioxane (4.0 M, 1.0 mL, 4.08 mmol, 50 equiv) was added dropwise. The mixture was subsequently allowed to warm to room temperature and stirred for 6 h at room temperature, with progress monitored by TLC until completion. The reaction mixture was concentrated *in vacuo* to afford the desired product **41** (114 mg, quantitative yield) as a white solid.

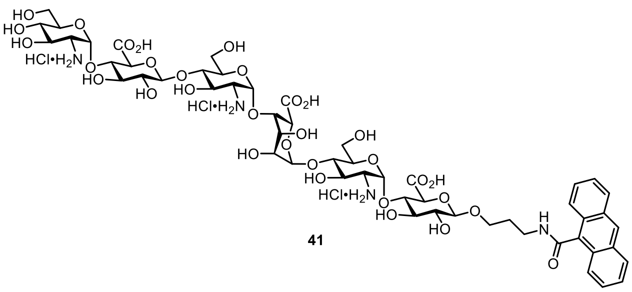

41

3-(Anthracene-9-carbonylamino)propyl (2-amino-2-deoxy-α-D-glucopyranosyl)-(1→4)-(β-D-glucopyranosyluronic acid)-(1→4)-(2-amino-2-deoxy-α-D-glucopyranosyl)-(1→4)-(α-L-idopyranosyluronic acid)-(1→4)-(2-amino-2-deoxy-α-D-glucopyranosyl)-(1→4)-β-D-glucopyranosiduronic acid trihydrochloride (**41**): ^{1}H NMR (400 MHz, CD_3OD) δ 8.58 (s, 1H), 8.08 (d, *J* = 8.4 Hz, 2H), 8.02 (d, *J* = 8.4 Hz, 2H), 7.67 – 7.36 (m, 4H), 5.58 (d, *J* = 3.9 Hz, 1H), 5.53 (d, *J* = 3.9 Hz, 2H), 5.39 (d, *J* = 3.8 Hz, 1H), 5.09 (d, *J* = 2.7 Hz, 1H), 5.07 (d, *J* = 2.6 Hz, 1H), 4.54 (d, *J* = 7.9 Hz, 1H), 4.42 (d, *J* = 7.7 Hz, 1H), 4.18 – 4.11 (m, 1H), 4.10 – 4.02 (m, 3H), 4.01 – 3.92 (m, 2H), 3.90 – 3.83 (m, 3H), 3.82 – 3.78 (m, 4H), 3.77 – 3.72 (m, 8H), 3.72 – 3.67 (m, 3H), 3.67 – 3.63 (m, 2H), 3.62 (t, *J* = 2.9 Hz, 1H), 3.46 (t, *J* = 9.4 Hz, 1H), 3.37 – 3.32 (m, 2H), 3.21 (dq, *J* = 12.0, 3.8 Hz, 2H), 3.14 (dd, *J* = 10.7, 3.8 Hz, 1H), 2.09 (p, *J* = 6.3 Hz, 1H); ^{13}C NMR (100 MHz, CD_3OD) δ 173.0, 172.4, 171.84, 171.81, 133.2 (2C), 132.6 (2C), 129.7 (2C), 129.3, 129.2, 127.8 (2C), 126.6 (2C), 126.0 (2C), 104.6, 104.3, 103.4, 98.2, 98.0, 94.3, 79.7, 79.6, 78.8, 78.0, 77.5, 77.3, 75.5, 75.1, 75.0, 74.8 (two peaks overlapped, 2C), 74.5, 73.4, 73.0, 71.1, 70.9, 70.8, 69.8, 69.4, 68.9, 68.7, 68.5, 61.4, 60.7, 60.6, 56.4, 56.0, 55.8, 38.1, 30.5; HRMS: m/z (ESI) calcd for $C_{54}H_{75}N_4O_{32}^+$, $[M + H]^+$, 1291.4359, found 1291.4346. $^{1}J^{13}{}_{C1–H1}$ = 172.0 Hz, 174.7 Hz, 174.7 Hz, 170.7 Hz, 161.5 Hz, 161.5 Hz.

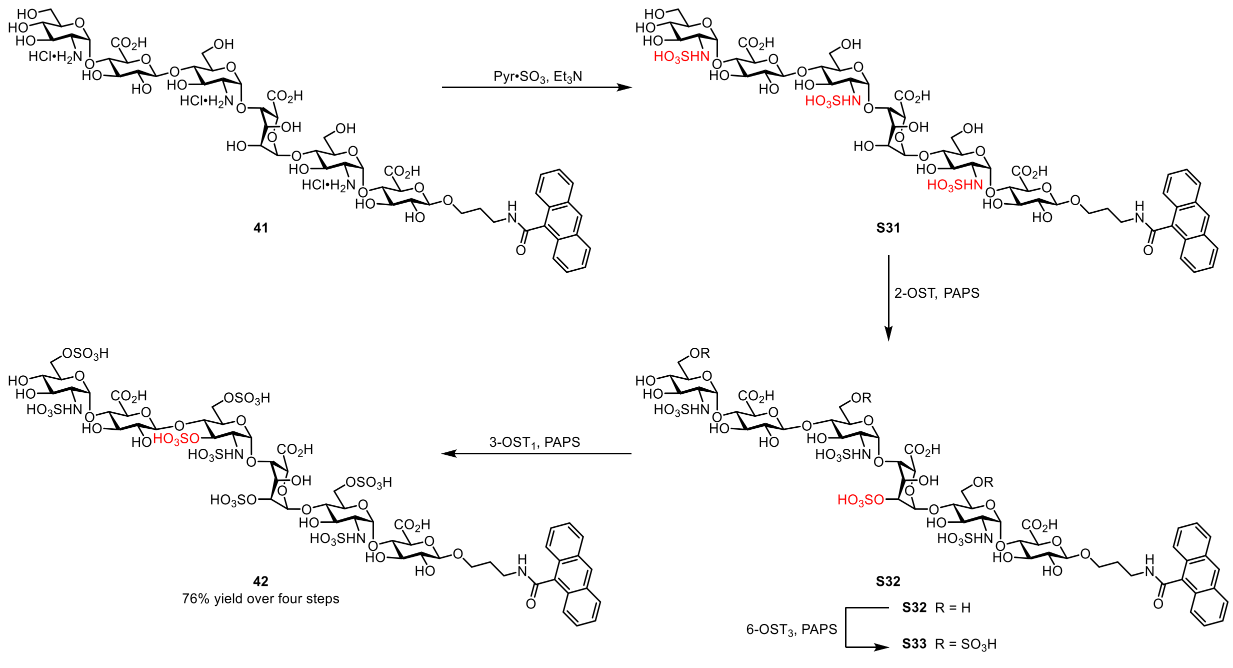

Figure S22. Chemo-Enzymatic Sulfation of **41**.

Expression and Purification of Sulfotransferases

Heparan sulfate (HS) 2-*O*-sulfotrasnferase was expressed in SF9 cells using the baculovirus expression approach and purified from the condition medium using a Heparin Sepharose (GE Health) column.[17] HS 6-*O*-sulfotransferase 3 and 3-*O*-sulfotransferase 1 were expressed in *E. coli* and purified by Ni-Agarose column.[17]

41 (33 mg, 0.0255 mmol) was dissolved in 20mL ddH_2O. A solution of Et_3N/MeOH (v/v: 9:1) was used to adjust the *p*H to 11. Pyr·SO_3 (60 mg, 0.382 mmol, 5 equiv per free NH_2 group) was added in three portions at 30 min interval. The reaction was monitored by HPLC equipped with an ion exchange column (ProPac PA1). The desired product **S31** (94% yield) was purified by Q-Sepharose column (16 x 200 mm) with a gradient elution of 0–30% buffer B (2 M NaCl, 20 mM sodium acetate, *p*H 5.0) from Buffer A (20 mM sodium acetate, *p*H 5.0) in 60 min, at the flow rate of 4 mL/min.

The chemically *N*-sulfated product **S31** was incubated at 37 °C for 12 h with PAPS (0.0414 mmol) and 500 µL 2-OST (8MU/mg) in a 10 mL 50 mM *p*H 7.2 MOPS buffer with 2 mM NaN_3 and 2 mM $MnCl_2$. The reaction was monitored by HPLC equipped with an ion exchange column

(ProPac PA1). The desired product **S32** (95% yield) was purified by Q-Sepharose column (16 x 200 mm) using the aforementioned condition.

The 2-*O*-sulfated product **S32** was incubated at 37 °C for 12 h in a 10 mL 50 mM *p*H 7.2 MOPS buffer with 2 mM NaN_3 and 2 mM $MnCl_2$, using PAPS (0.16 mmol) and 2 mL 6-OST_3 (6.8 mg/mL). The reaction was monitored by HPLC equipped with an ion exchange column (ProPac PA1). The desired product **S33** (88% yield) was purified by Q-Sepharose column (16 x 200 mm) with a gradient elution of 0–70% buffer B (2 M NaCl, 20 mM sodium acetate, *p*H 5.0) from Buffer A (20 mM sodium acetate, *p*H 5.0) in 80 minutes, at the flow rate of 4 mL/min.

The 6-*O*-sulfated product **S33** was incubated at 37 °C for 12 h with PAPS (0.0414 mmol) and 800 μL 3-OST_1 (0.11 mg/mL) in a 10 mL 50mM *p*H 7.2 MOPS buffer with 2 mM NaN_3 and 2 mM $MnCl_2$. The reaction was monitored by HPLC equipped with an ion exchange column (ProPac PA1). The desired product **42** (97% yield) was purified by Q-Sepharose column (16 x 200 mm) using the aforementioned condition.

NMR & HPLC-MS Analysis

All NMR experiments were performed at 303 K on a Bruker Avance III 850 MHz spectrometer with Topspin 2.1 software. The sample (15 mg) was dissolved in 0.5 mL D_2O (99.996%, Sigma, Co.) and lyophilized three times to remove the exchangeable protons. The samples were redissolved in 0.5 mL D_2O with 2 mM EDTA (ethylenediaminetetraacetic-d_{12} acid) and transferred to NMR tubes (OD 5 mm, Norrell). ^1H NMR experiments were performed with 32 scans and an acquisition time of 1.2 seconds and 10 seconds relaxation delay. 2D ^1H–^{13}C experiments were performed with 24 scans and 256 increments, 1.5 sec relaxation delay. ^{13}C NMR experiments were performed with 20k scans and an acquisition time of 0.699 sec.

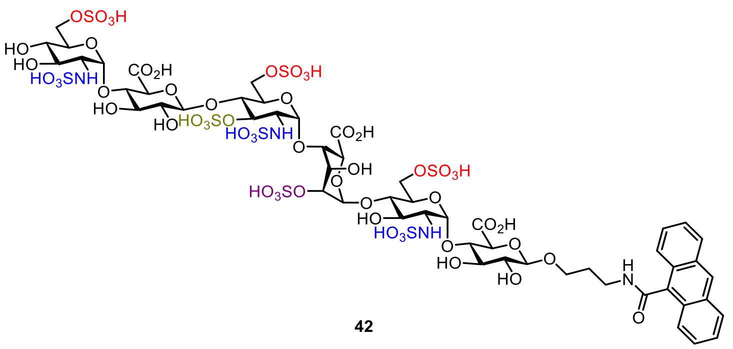

42

3-(Anthracene-9-carbonylamino)propyl (2-deoxy-2-sulfamino-6-*O*-sulfo-α-D-glucopyranosyl)-(1→4)-(β-D-glucopyranosyluronic acid)-(1→4)-(2-deoxy-2-sulfamino-3,6-di-*O*-sulfo-α-D-glucopyranosyl)-(1→4)-(2-*O*-sulfo-α-L-idopyranosyluronic acid)-(1→4)-(2-deoxy-2-sulfamino-6-*O*-sulfo-α-D-glucopyranosyl)-(1→4)-β-D-glucopyranosiduronic acid (**42**): ^{1}H NMR (850 MHz, D_2O) δ 8.67 (s, 1H), 8.14 (d, *J* = 8.4 Hz, 2H), 7.97 (d, *J* = 8.4 Hz, 2H), 7.64 – 7.58 (m, 4H), 5.61 (d, *J* = 3.3 Hz, 1H), 5.52 (d, *J* = 3.3 Hz, 1H), 5.50 (d, *J* = 2.9 Hz, 1H), 5.17 (d, *J* = 3.4 Hz, 1H), 4.77 (d, *J* = 2.8 Hz, 1H), 4.61 (d, *J* = 7.7 Hz, 1H), 4.48 (d, *J* = 7.7 Hz, 1H), 4.47 (d, *J* = 10.8 Hz, 1H), 4.43 (d, *J* = 10.8 Hz, 1H), 4.37 – 4.34 (m, 2H), 4.31 – 4.30 (m, 1H), 4.25 (d, *J* = 10.9 Hz, 1H), 4.19 (d, *J* = 10.9 Hz, 1H), 4.15 – 4.13 (m, 4H), 4.09 – 4.06 (m, 1H), 3.96 (t, *J* = 9.5 Hz, 1H) 3.92 (d, *J* = 10 Hz, 1H), 3.87 (d, *J* = 10 Hz, 1H), 3.84 – 3.80 (m, 3H), 3.78 – 3.71 (m, 7H), 3.64 – 3.60 (m, 2H), 3.56 (t, *J* = 8.2 Hz, 1H), 3.43 (dd, *J* = 10.7, 2.7 Hz, 1H), 3.40 (t, *J* = 8.2 Hz, 1H), 3.31 (t, *J* = 8.2 Hz, 1H), 3.26 (dd, *J* = 10.5, 3.3 Hz, 1H), 3.24 (dd, *J* = 10.2, 3.3 Hz, 1H), 2.09 (dt, *J* = 6.7, 4.4 Hz, 2H); ^{13}C NMR (213.8 MHz, D_2O) δ 177.9, 177.3, 176.8, 174.9, 133.4 (2C), 132.9, 131.4, 131.3 (2C), 130.12 (2C), 130.08 (2C), 128.6 (2C), 126.9 (2C), 104.8, 103.8, 102.1, 100.3, 100.2, 98.8, 80.4, 79.7, 79.52, 79.49, 78.9, 78.8, 78.7, 78.0, 75.5, 75.44, 75.36, 73.8, 73.1, 72.84, 72.83, 72.4, 72.3, 72.2, 71.9, 71.7, 71.2, 70.6, 69.0, 68.9, 68.7, 60.7, 60.6, 59.3, 39.8, 31.1; HRMS: m/z (ESI) calcd for $C_{54}H_{72}N_4O_{56}S_8^{2-}$, $[M - 2H]^{2-}$, 964.0343, found 964.0328.

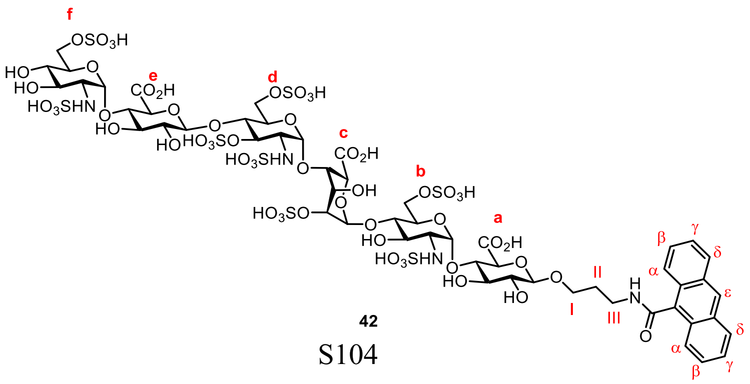

42

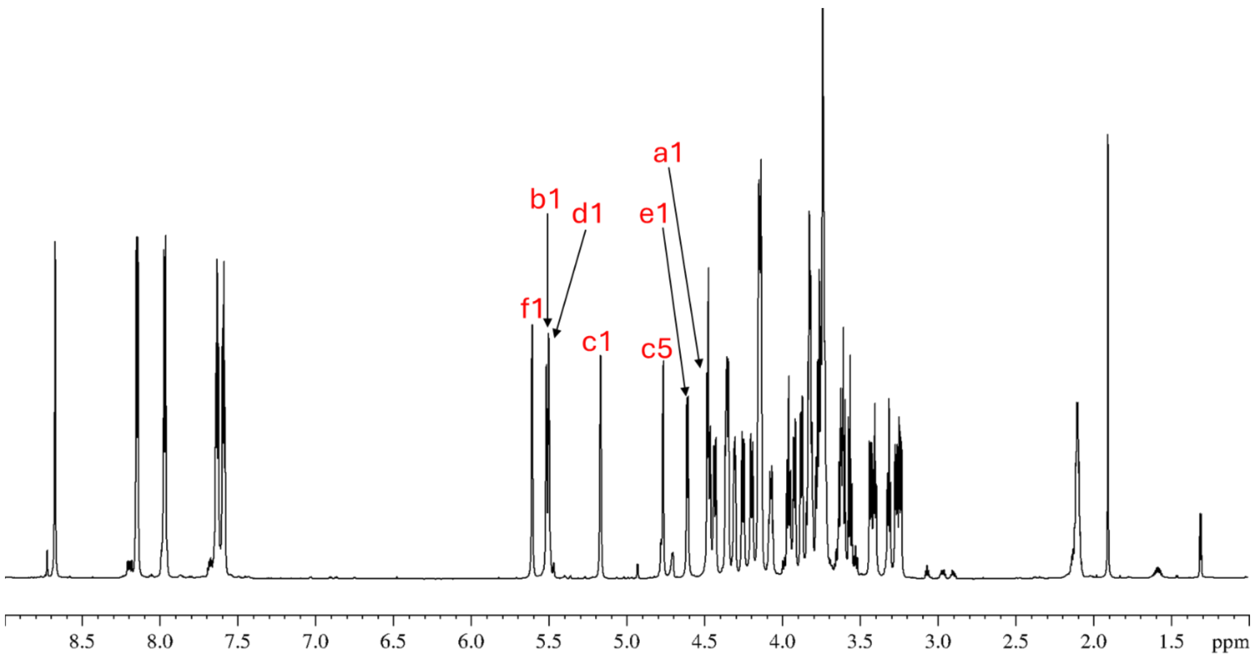

Figure S23a. ^{1}H NMR Chemical Shift Assignment for Anomeric Signals of **42**.

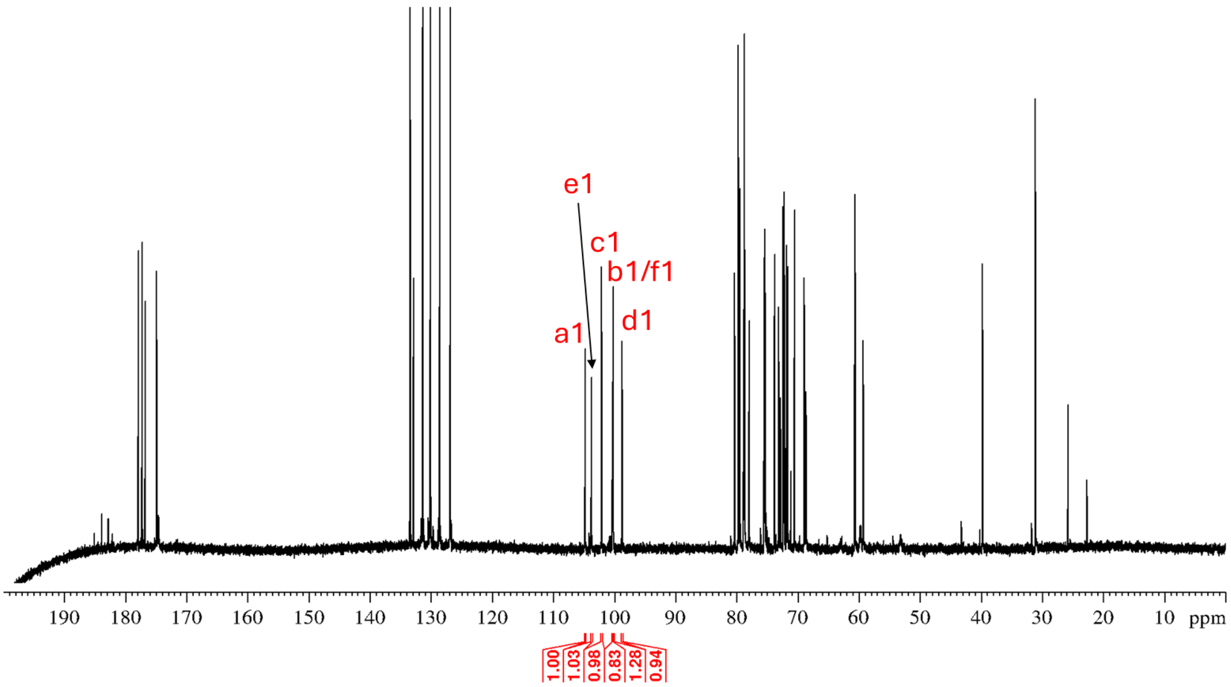

Figure S23b. ^{13}C NMR Chemical Shift Assignment for Anomeric Signals of **42**.

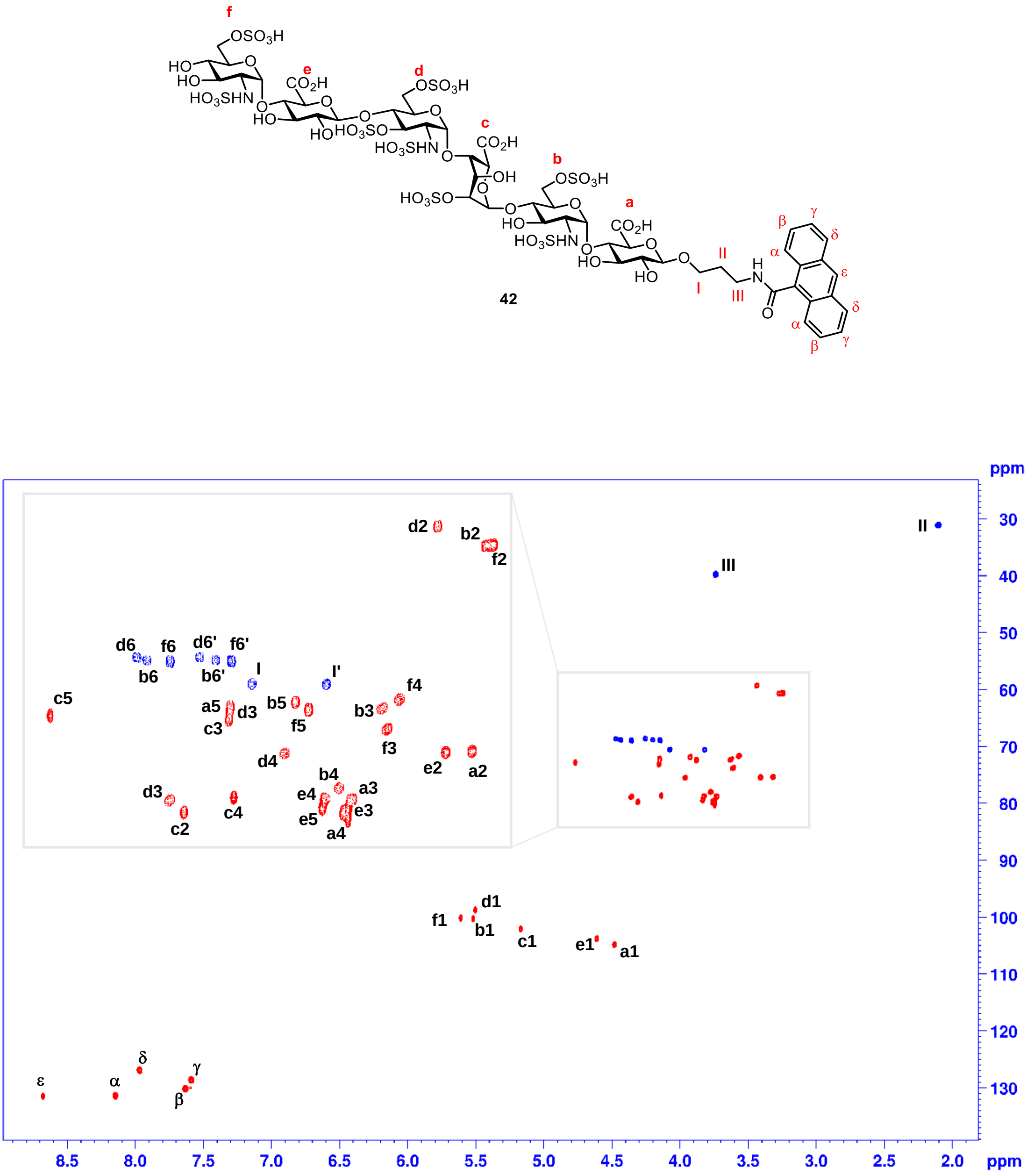

Figure S23c. HSQC Analysis of **42**.

The HSQC spectrum shows that the C3–H correlation in ring **d** appears approximately 0.7 ppm further downfield in the ^{1}H dimension and approximately 6 ppm further downfield in the ^{13}C dimension compared to the corresponding C3–H signals in rings **b** and **f**, suggesting that the C3-OH of GlcN of ring **d** selectively gets sulfated.[18-19] Moreover, the C2–H correlation in ring **c** appears approximately 1.0 ppm further downfield in the ^{1}H dimension and approximately 4 ppm

further downfield in the ^{13}C dimension compared to the corresponding C2–H signals in rings **a** and **e**, suggesting that the C2-OH of IdoA of ring **c** selectively gets sulfated.[18-19] The assignment of ^{1}H NMR chemical shifts of H3 on ring **d** and H2 on ring **c** was based on HSQC–COSY analysis of **42**.

	1	2	3	4	5	6a	6'
a	4.48/104.79	3.31/75.44	3.76/79.75	3.74/80.39	4.14/72.25		
b	5.51/100.36	3.26/60.70	3.62/72.33	3.77/77.99	3.92/71.88	4.19/68.88	4.43/68.88
c	5.16/102.11	**4.30/79.76**	4.16/72.22	4.14/78.68	4.76/72.84		
d	5.50/98.79	3.43/59.31	**4.36/78.87**	3.96/75.54	4.15/73.14	4.25/68.65	4.47/68.65
e	4.61/103.76	3.40/75.43	3.72/78.78	3.83/78.67	3.82/79.49		
f	5.61/100.21	3.24/60.62	3.60/73.83	3.56/71.66	3.88/72.42	4.14/68.97	4.35/68.97
I/I'	4.08/70.76	3.85/70.76					
II	2.11/31.14						
III	3.75/39.91						
a	8.16/131.44						
b	7.59/128.64						
g	7.36/131.17						
d	7.98/126.93						
e	8.70/131.51						

Figure S23d. Full ^{1}H NMR/^{13}C NMR Chemical Shift Assignment for **42**.

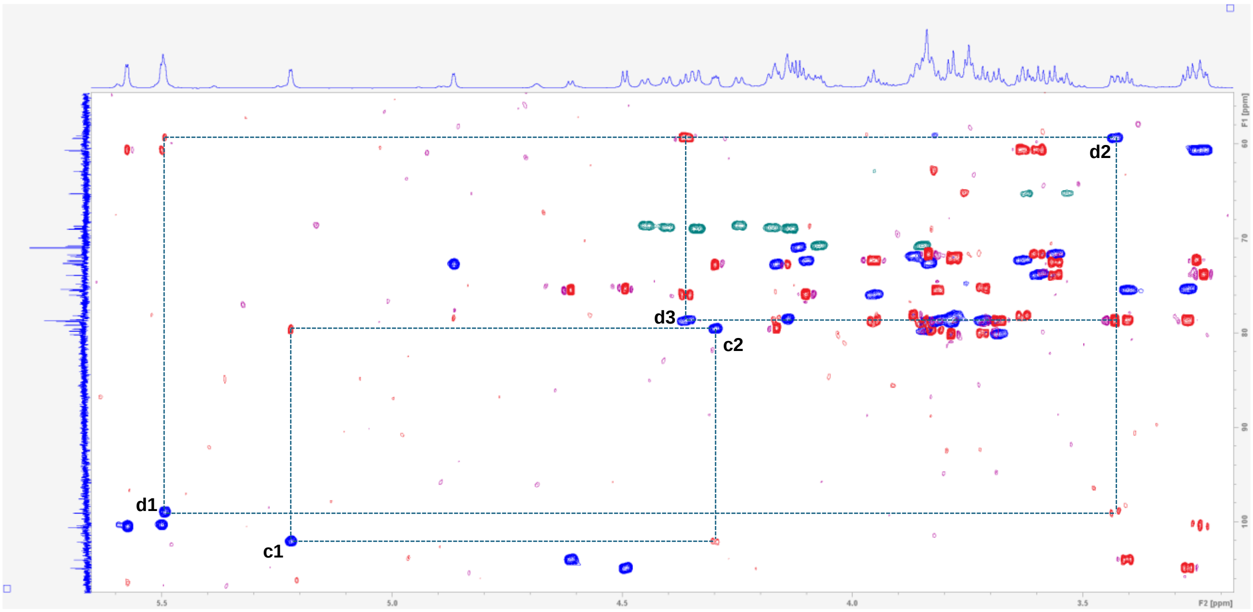

Figure S23e. HSQC–COSY Analysis of **42**.

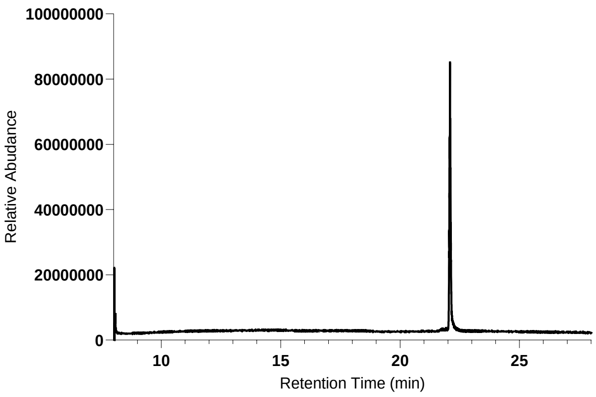

Figure S23f. LC–MS Chromatogram for **42**.

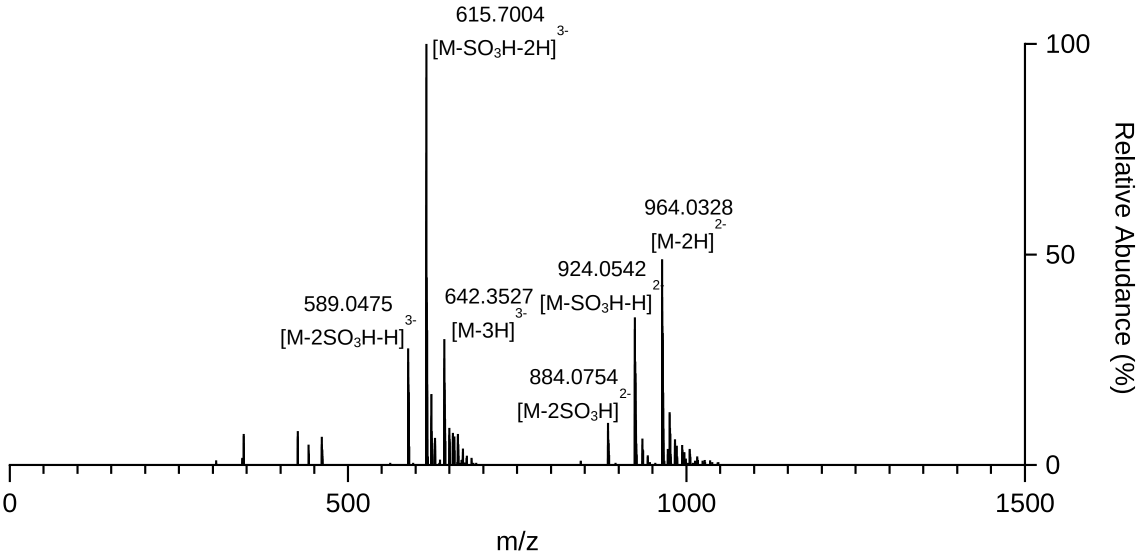

Figure S23g. Mass Spec Analysis of **42**.

I. Assembly of Full-Length Precursors of Heparan Sulfate Octasaccharides

a. Heparan Sulfate GlcN(α1-4)GlcA(β1-4)GlcN(α1-4)IdoA(α1-4)GlcN(α1-4)IdoA(α1-4)GlcN(α1-4)GlcA Module Synthesis

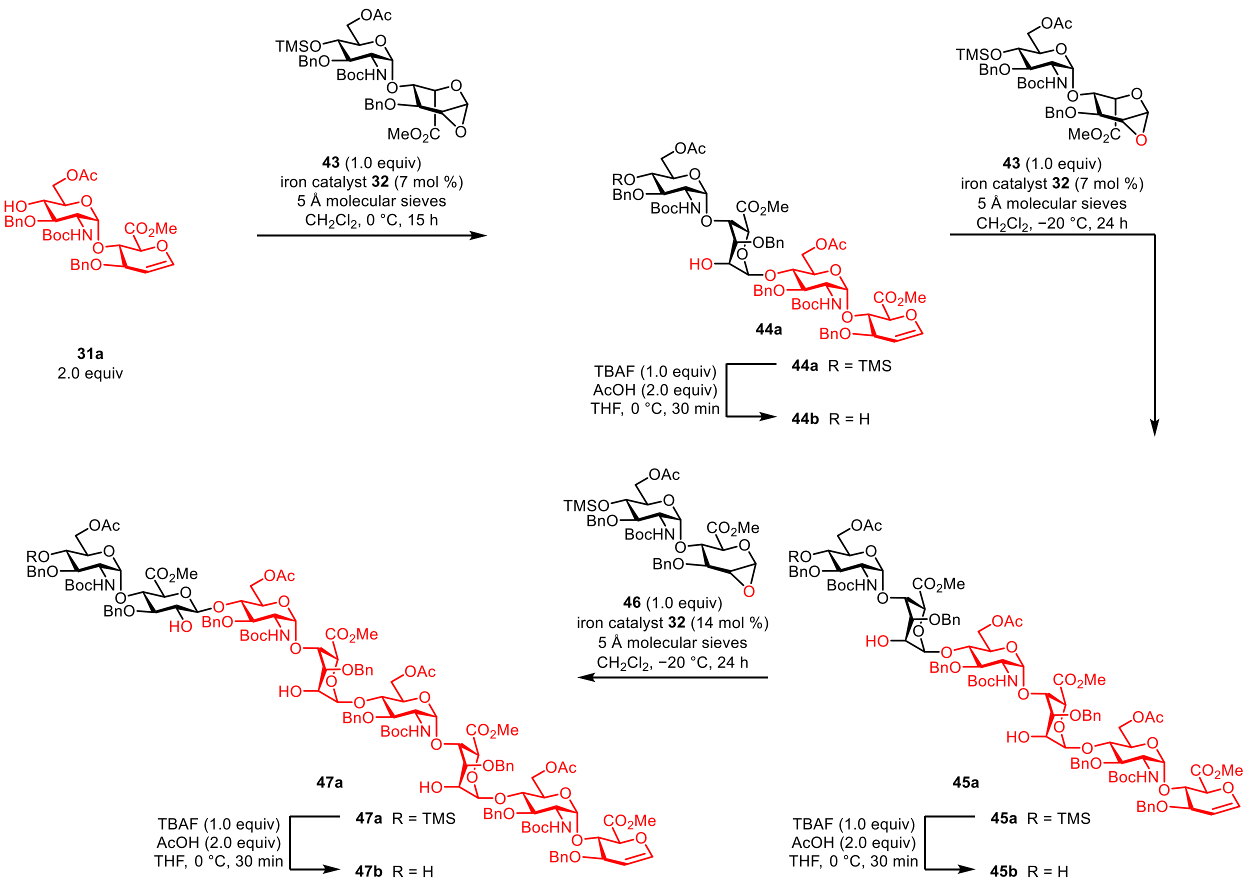

Figure S24. GlcN(α1-4)GlcA(β1-4)GlcN(α1-4)IdoA(α1-4)GlcN(α1-4)IdoA(α1-4)GlcN(α1-4)GlcA Module Synthesis.

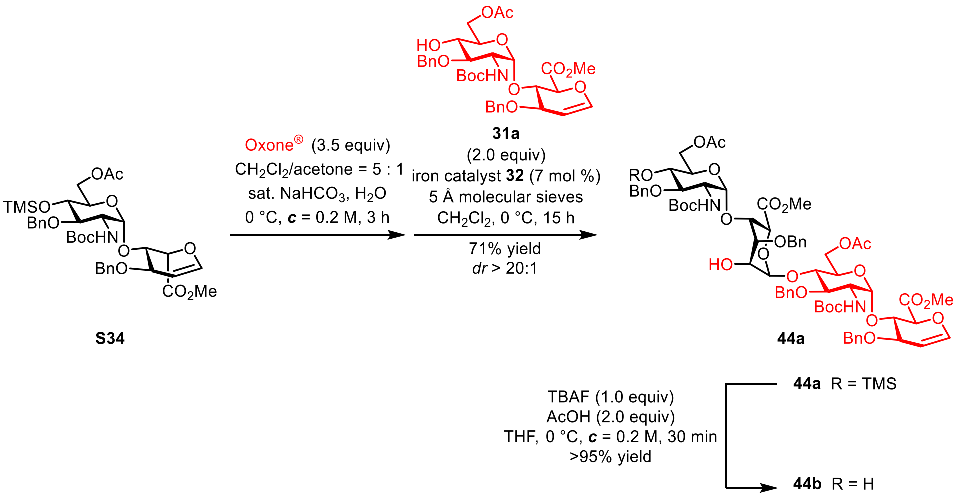

S34 was synthesized according to the following procedure.

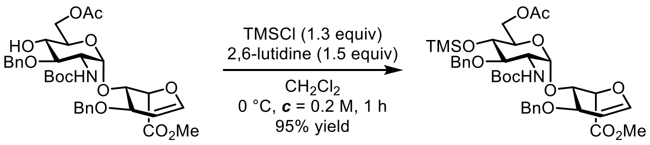

29a **S34**

To a 100 mL flame-dried round-bottom flask equipped with a stir bar, compound **29a** (2.0 g, 3.04 mmol, 1.0 equiv), 2,6-lutidine (0.53 mL, 4.56 mmol, 1.5 equiv), and anhydrous CH_2Cl_2 (15.2 mL) were added. The mixture was cooled to 0 °C before TMSCl (0.50 mL, 3.95 mmol, 1.3 equiv) was added dropwise. The reaction mixture was then stirred at 0 °C for 1 h until the starting material **29a** was fully consumed (monitored by TLC). The reaction mixture was diluted with CH_2Cl_2 (10 mL) before saturated aqueous NH_4Cl solution (15 mL) was added to quench the reaction. The organic phase was separated from the aqueous one, which was further extracted with CH_2Cl_2 (15 mL × 2). The combined organic phase was washed with brine (20 mL) and dried over Na_2SO_4. After concentration *in vacuo*, the residue was purified through a silica gel flash column (hexanes/EtOAc: from 100:1 to 4:1) to afford the desired product **S34** (2.1 g, 95% yield) as white foam.

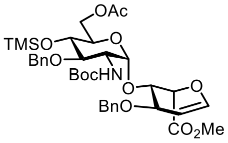

S34

Methyl 3-*O*-benzyl-4-*O*-(6-*O*-acetyl-3-*O*-benzyl-2-*tert*-butoxycarbonylamino-4-*O*-trimethylsilyl-2-deoxy-α-D-glucopyranosyl)-L-guluronal (**S34**): $[\alpha]_D^{23}$ +14.8 (acetone, *c* = 1.0); IR ν_{max} (neat)/cm^{-1}: 2954 (w), 1742 (s), 1717 (s), 1500 (m), 1367 (m), 1250 (s), 1035 (s); ^{1}H NMR (400 MHz, $CDCl_3$) δ 7.61 – 7.04 (m, 10H), 6.66 (d, *J* = 6.2 Hz, 1H), 4.98 (d, *J* = 6.6 Hz, 1H), 4.82 (d, *J* = 3.8 Hz, 1H), 4.70 (d, *J* = 11.2 Hz, 1H), 4.66 – 4.59 (m, 3H), 4.58 (d, *J* = 11.8 Hz, 1H), 4.43 – 4.33 (m, 2H), 4.33 – 4.30 (m, 1H), 4.08 (dd, *J* = 11.8, 3.9 Hz, 1H), 3.84 (s, 3H), 3.84 – 3.76 (m, 1H), 3.70 (dd, *J* = 5.4, 2.2 Hz, 1H), 3.66 (t, *J* = 9.1 Hz, 1H), 3.57 (ddd, *J* = 9.8, 3.7, 2.0 Hz, 1H), 3.35 (dd, *J* = 10.5, 8.5 Hz, 1H), 2.10 (s, 3H), 1.33 (s, 9H), 0.06 (s, 9H); ^{13}C NMR (100 MHz, $CDCl_3$) δ 170.7, 168.7, 155.1, 146.1, 138.2, 137.6, 128.5 (2C), 128.1 (2C), 127.9, 127.7 (2C), 127.6 (2C), 127.4, 98.4, 96.3, 80.1, 79.5, 75.3, 72.4, 71.2, 71.0, 70.9, 70.2, 65.9, 62.6, 53.9, 52.6, 28.2 (3C), 20.8, 0.4 (3C); HRMS: m/z (ESI) calcd for $C_{37}H_{52}NO_{12}Si^+$, [M + H]$^+$, 730.3253, found 730.3259.

To a 250 mL flask equipped with a stir bar at 0 °C, were added glycal **S34** (730 mg, 1.0 mmol, 1.0 equiv) in CH_2Cl_2/acetone mixture (v/v: 5:1, 16.7 mL) and saturated aqueous $NaHCO_3$ solution (28.7 mL), followed by addition of Oxone® ($KHSO_5 \cdot 0.5KHSO_4 \cdot 0.5K_2SO_4$) (2.16 g, 3.5 mmol, 3.5 equiv) in H_2O (18.7 mL) dropwise. After stirring vigorously at 0 °C for 2 h, the reaction mixture was extracted with CH_2Cl_2 (20 mL × 3). The combined organic phase was dried over anhydrous Na_2SO_4 and concentrated *in vacuo*. The residue was further azeotropically dried with anhydrous toluene (10 mL × 3). The obtained glycal epoxide was assayed by ^{1}H NMR to get the diastereomeric ratio (*dr* > 20:1) and directly used in the next step.

To a 25 mL flame-dried round-bottom flask equipped with a stir bar were added glycosyl acceptor **31a** (1.32 g, 2.0 mmol, 2.0 equiv) and freshly activated 5 Å molecular sieves, powder (*ca.* 300 mg). After the flask was evacuated and backfilled with N_2, anhydrous CH_2Cl_2 (1.2 mL) was added. To a flame-dried sealable 2-dram vial equipped with a stir bar were added the iron porphyrin triflate catalyst **32** (0.07 mmol, 7 mol %) and freshly activated 5 Å molecular sieves, powder (*ca.* 100 mg). The vial was evacuated and backfilled with N_2 three times and anhydrous CH_2Cl_2 (1.2 mL) was added. The mixture in the vial was stirred at room temperature for 5 min before it was transferred to the flask. The resulting mixture was stirred at −20 °C for 5 min and a solution of the aforementioned glycal epoxide in anhydrous CH_2Cl_2 (1.6 mL) was then added to the mixture at −20 °C dropwise. The reaction mixture was kept at 0 °C for 15 h and then

quenched with a solution of imidazole (16 mg) in CH_2Cl_2 (0.4 mL) at the same temperature. The mixture was filtered through a pad of Celite® silica gel and eluted with CH_2Cl_2 (20 mL). The organic layer was then concentrated *in vacuo,* and the residue was purified through a silica gel flash column (hexanes/EtOAc: from 100:1 to 3:2) to afford the desired glycosylation product **44a** (997 mg, 71% yield).

To a 25 mL round-bottom flask equipped with a stir bar were added the glycosylation product **44a** obtained from the previous step and THF (4.0 mL). The mixture was cooled to 0 °C and a premixed solution of TBAF (1 M solution in THF, 1.0 mL, 1.0 mmol, 1.0 equiv) and AcOH (114 μL, 2.0 mmol, 2.0 equiv) was then added. The reaction mixture was stirred for 30 min at 0 °C, with progress monitored until completion. The reaction mixture was then diluted with EtOAc (10 mL) before H_2O (5 mL) was added. The organic phase was separated from the aqueous one, which was further extracted with EtOAc (10 mL × 3). The combined organic phase was washed with brine (10 mL) and dried over Na_2SO_4. After concentration *in vacuo*, the residue was purified through a silica gel flash column (hexanes/EtOAc: from 100:1 to 1:1) to afford the desired product **44b** (945 mg, >95% yield).

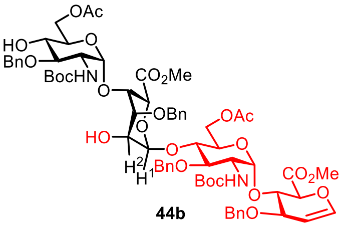

44b

Methyl 3-*O*-benzyl-4-*O*-[(6-*O*-acetyl-3-*O*-benzyl-2-*tert*-butoxycarbonylamino-2-deoxy-α-D-glucopyranosyl)-(1→4)-(methyl 3-*O*-benzyl-α-L-idopyranosyluronate)-(1→4)-(6-*O*-acetyl-3-*O*-benzyl-2-*tert*-butoxycarbonylamino-2-deoxy-α-D-glucopyranosyl)]-D-glucuronal (44b): $[\alpha]_D^{23}$ +35.9 (acetone, c = 1.0); IR ν_{max} (neat)/cm^{-1}: 3432 (w), 2930 (w), 1716 (s), 1717 (s), 1499 (m), 1367 (m), 1246 (s), 1033 (s); ^{1}H NMR (400 MHz, acetone-d_6) δ 7.65 – 7.01 (m, 20H), 6.60 (d, J = 6.4 Hz, 1H), 6.28 (d, J = 9.4 Hz, 1H), 5.87 (d, J = 9.5 Hz, 1H), 5.22 (d, J = 4.0 Hz, 1H), 5.17 (d, J = 3.7 Hz, 1H), 5.08 (d, J = 3.4 Hz, 1H), 5.05 – 4.96 (m, 2H), 4.91 – 4.84 (m, 2H), 4.84 – 4.82 (m, 1H), 4.83 – 4.74 (m, 2H), 4.76 – 4.72 (m, 2H), 4.72 (brs, 1H), 4.61 (d, J = 11.2 Hz, 1H), 4.58 – 4.53 (m, 2H), 4.51 (d, J = 11.6 Hz, 1H), 4.45 – 4.41 (m, 1H), 4.38 – 4.28 (m, 2H),

4.24 (dd, J = 11.9, 4.9 Hz, 1H), 4.12 – 4.02 (m, 2H), 3.96 (t, J = 5.5 Hz, 1H), 3.94 – 3.90 (m, 1H), 3.90 – 3.83 (m, 2H), 3.83 – 3.73 (m, 4H), 3.72 – 3.67 (m, 1H), 3.64 – 3.49 (m, 7H), 2.09 (s, 3H), 1.97 (s, 3H), 1.35 (s, 9H), 1.32 (s, 9H); ^{13}C NMR (100 MHz, acetone-d_6) δ 170.2, 170.1, 169.6, 167.7, 155.6, 155.4, 145.0, 139.5, 139.3, 138.6, 138.5, 128.21 (2C), 128.19 (2C), 128.0 (2C), 127.9 (2C), 127.80 (2C), 127.75 (2C), 127.50 (2C), 127.49, 127.45, 127.4 (2C), 127.0, 126.9, 101.0, 98.9, 98.6, 97.8, 80.4, 78.3, 78.0 (two peaks overlapped, 2C), 76.5, 76.4, 74.2, 73.8, 73.6, 73.4, 72.9, 72.5, 70.9, 70.5, 70.3, 70.1, 69.9, 69.2, 67.8, 62.9, 62.6, 54.5, 54.1, 51.3, 51.3, 27.8 (3C), 27.7 (3C), 20.0, 19.9; HRMS: m/z (ESI) calcd for $C_{68}H_{87}N_2O_{25}^+$, $[M + H]^+$, 1331.5592, found 1331.5558. $^1J_{^{13}C1–H1}$ = 170.2 Hz, 171.5 Hz, 170.6 Hz. $^3J_{H1–H2}$ = 3.4 Hz.

The stereochemistry of newly formed anomeric center (C1) of **44b** was determined by measuring $^1J_{^{13}C1–H1}$ (170.6 Hz) through un-decoupled HSQC experiments.[11-12] The C2 stereochemistry was determined by the stereochemistry of the glycal-α-epoxide **43**.

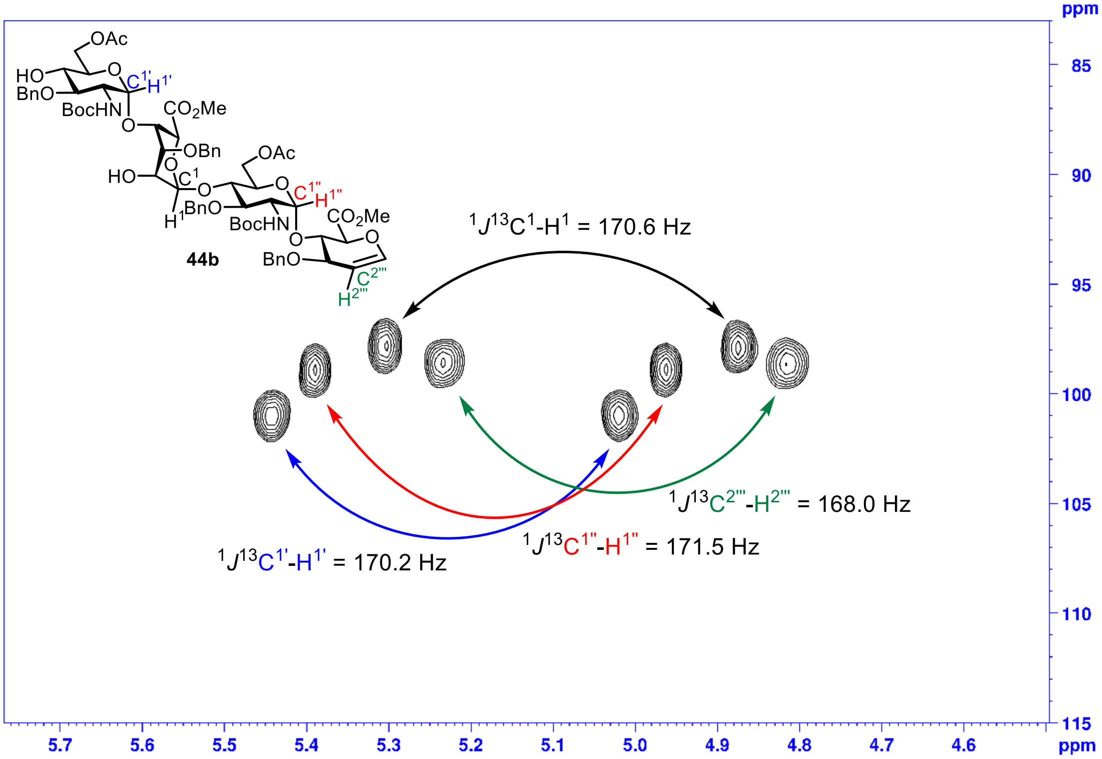

Figure S25. Un-decoupled HSQC Analysis to Determine Stereochemistry of **44b**.

A $^{1}J^{13}_{\mathrm{C1-H1}}$ value of 170.6 Hz suggested that the newly formed glycosidic bond is in axial position, which confirmed the generation of a 1,2-*trans*-α-glycosidic linkage.

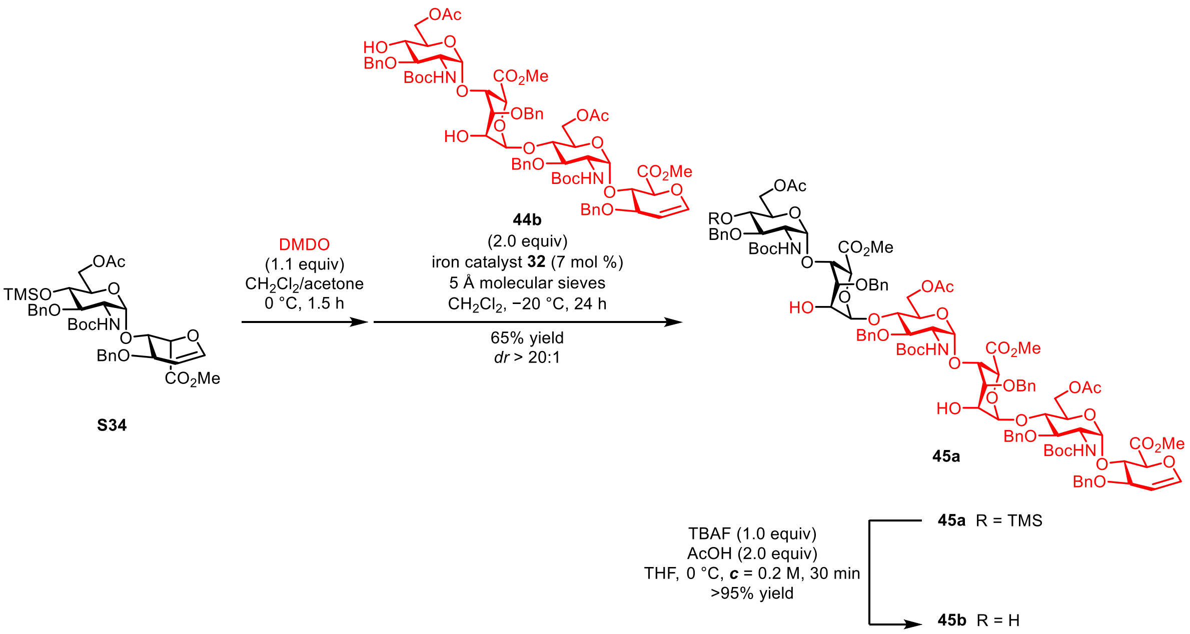

To a flame-dried sealable 3-dram vial (vial **A**) equipped with a stir bar were added glycosyl acceptor **44b** (1.33 g, 1.0 mmol, 2.0 equiv) and freshly activated 5 Å molecular sieves, powder (*ca.* 200 mg). After the vial was evacuated and backfilled with N_2, anhydrous CH_2Cl_2 (0.6 mL) was added. To a second flame-dried sealable 2-dram vial (vial **B**) equipped with a stir bar were added the iron porphyrin triflate catalyst **32** (0.035 mmol, 7 mol %) and freshly activated 5 Å molecular sieves, powder (*ca.* 60 mg). Vial **B** was evacuated and backfilled with N_2 three times and anhydrous CH_2Cl_2 (0.6 mL) was added. The mixture in vial **B** was stirred at room temperature for 5 min before it was transferred to vial **A**. The mixture was stirred at −40 °C for 5 min and a solution of the aforementioned glycal epoxide **43** in anhydrous CH_2Cl_2 (0.8 mL) was then added to the mixture at −40 °C dropwise. The reaction mixture was kept at −20 °C for 24 h and then quenched with a solution of imidazole (8 mg) in CH_2Cl_2 (0.2 mL) at the same temperature. The mixture was filtered through a pad of Celite® silica gel and eluted with CH_2Cl_2 (10 mL). The organic layer was then concentrated *in vacuo*, and the residue was purified

through a silica gel flash column (hexanes/EtOAc: from 100:1 to 1:1) to afford the desired glycosylation product **45a** (675 mg, 65% yield).

To a 25 mL round-bottom flask equipped with a stir bar were added the glycosylation product **45a** obtained from the previous step and THF (2.0 mL). The mixture was cooled to 0 °C and a premixed solution of TBAF (1 M solution in THF, 0.5 mL, 0.5 mmol, 1.0 equiv) and AcOH (57 μL, 1.0 mmol, 2.0 equiv) was then added. The reaction mixture was stirred for 30 min at 0 °C, with progress monitored until completion. The reaction mixture was then diluted with EtOAc (5 mL) before H_2O (2 mL) was added. The organic phase was separated from the aqueous one, which was further extracted with EtOAc (5 mL × 3). The combined organic phase was washed with brine (5 mL) and dried over Na_2SO_4. After concentration *in vacuo*, the residue was purified through a silica gel flash column (hexanes/acetone: from 100:1 to 3:2) to afford the desired product **45b** (652 mg, >95% yield).

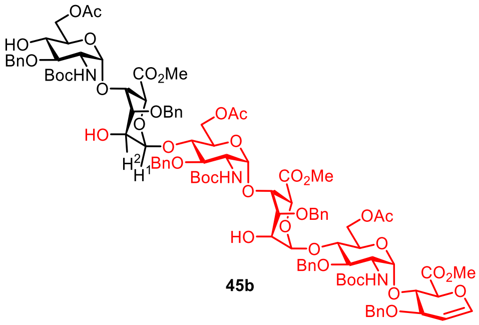

45b

Methyl 3-*O*-benzyl-4-*O*-[(6-*O*-acetyl-3-*O*-benzyl-2-*tert*-butoxycarbonylamino-2-deoxy-α-D-glucopyranosyl)-(1→4)-(methyl 3-*O*-benzyl-α-L-idopyranosyluronate)-(1→4)-(6-*O*-acetyl-3-*O*-benzyl-2-*tert*-butoxycarbonylamino-2-deoxy-α-D-glucopyranosyl)-(1→4)-(methyl 3-*O*-benzyl-α-L-idopyranosyluronate)-(1→4)-(6-*O*-acetyl-3-*O*-benzyl-2-*tert*-butoxycarbonylamino-2-deoxy-α-D-glucopyranosyl)]-D-glucuronal (45b): $[\alpha]_D^{23}$ +35.9 (acetone, *c* = 1.0); IR ν_{max} (neat)/cm^{-1}: 3429 (w), 2933 (w), 1740 (s), 1367 (m), 1499 (m), 1245 (s), 1106 (m), 1031 (s); ^{1}H NMR (400 MHz, acetone-d_6) δ 7.59 – 6.99 (m, 30H), 6.61 (d, *J* = 6.3 Hz, 1H), 6.35 – 6.23 (m, 2H), 5.88 (d, *J* = 9.5 Hz, 1H), 5.21 (d, *J* = 4.0 Hz, 1H), 5.19 (d, *J* = 4.0 Hz, 1H), 5.17 (d, *J* = 3.7 Hz, 1H), 5.07 (d, *J* = 3.6 Hz, 1H), 5.05 (d, *J* = 3.6 Hz, 1H), 5.04 – 4.97 (m, 2H), 4.89 – 4.84 (m, 3H), 4.84 – 4.80 (m, 4H), 4.79 – 4.76 (m, 2H), 4.76 – 4.75 (m, 1H), 4.74 (d, *J* = 7.5 Hz, 1H), 4.71 – 4.67 (m, 1H), 4.61 (d, *J* = 11.3 Hz, 1H), 4.59 – 4.53 (m, 3H),

4.51 (d, J = 11.6 Hz, 1H), 4.45 – 4.39 (m, 2H), 4.37 – 4.31 (m, 2H), 4.32 – 4.28 (m, 1H), 4.24 (dd, J = 11.9, 4.9 Hz, 1H), 4.16 – 4.02 (m, 3H), 3.99 – 3.91 (m, 3H), 3.91 – 3.82 (m, 6H), 3.81 – 3.75 (m, 3H), 3.75 – 3.68 (m, 2H), 3.61 – 3.53 (m, 6H), 3.52 (s, 3H), 3.47 (s, 3H), 2.09 (s, 3H), 2.08 (s, 3H), 1.97 (s, 3H), 1.34 (s, 9H), 1.32 (s, 9H), 1.27 (s, 9H); ^{13}C NMR (100 MHz, acetone-d_6) δ 171.1, 171.0, 170.9, 170.40, 170.35, 168.5, 156.5, 156.4, 156.3, 145.8, 140.4, 140.2, 140.1, 139.5, 139.4, 139.3, 129.14 (2C), 129.10 (2C), 129.06 (2C), 128.9 (two peaks overlapped, 4C), 128.73 (2C), 128.67 (2C), 128.62 (2C), 128.60 (2C), 128.43, 128.38, 128.36 (2C), 128.33, 128.32 (2C), 128.27 (2C), 127.9, 127.8, 127.7, 101.9, 101.7, 99.8, 99.5, 98.6, 98.5, 81.3, 79.6, 79.2, 78.89, 78.86 (two peaks overlapped, 2C), 77.2, 77.09, 77.06, 76.6, 75.1, 75.0, 74.6, 74.4, 74.3, 73.8, 73.6, 73.6, 73.2, 71.8, 71.3, 71.2, 70.9, 70.7, 70.6, 70.5, 70.2, 70.1, 68.7, 63.8, 63.4, 63.1, 55.4, 55.2, 55.0, 52.18, 52.15, 52.1, 28.7 (3C), 28.6 (two peaks overlapped, 6C), 20.92, 20.86, 20.8; HRMS: m/z (ESI) calcd for $C_{102}H_{130}N_3O_{38}^+$, $[M + H]^+$, 2004.8327, found 2004.8381. $^1J_{^{13}C1\text{–}H1}$ = 169.9 Hz, 169.9 Hz, 172.3 Hz, 172.1, 172.1 Hz. $^3J_{H1\text{–}H2}$ = 3.6 Hz.

The stereochemistry of newly formed anomeric center (C1) of **45b** was determined by measuring $^1J_{^{13}C1\text{–}H1}$ (172.1 Hz) through un-decoupled HSQC experiments.[11-12] The C2 stereochemistry was determined by the stereochemistry of glycal-α-epoxide **43**.

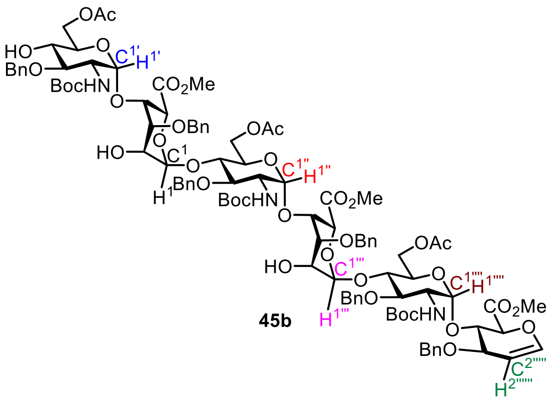

45b

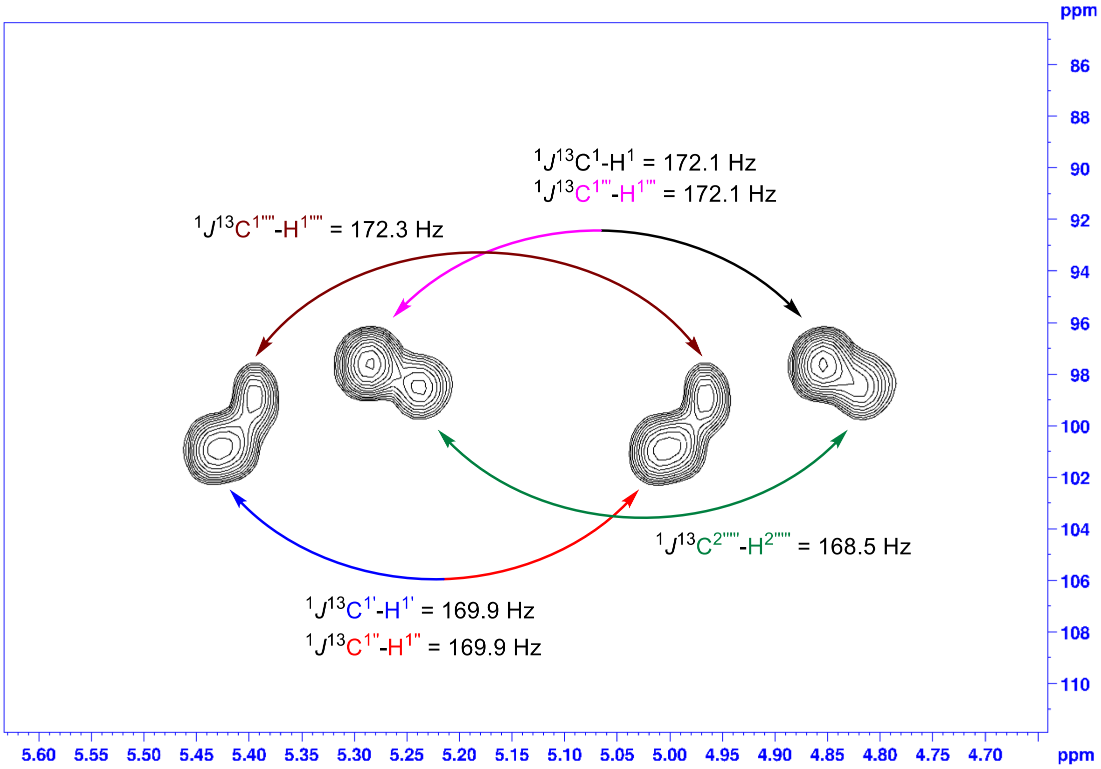

Figure S26. Un-decoupled HSQC Analysis to Determine Stereochemistry of **45b**.

A $^{1}J^{13}{}_{C1–H1}$ value of 172.1 Hz suggested that the newly formed glycosidic bond is in axial position, which confirmed the generation of a 1,2-*trans*-α-glycosidic linkage.

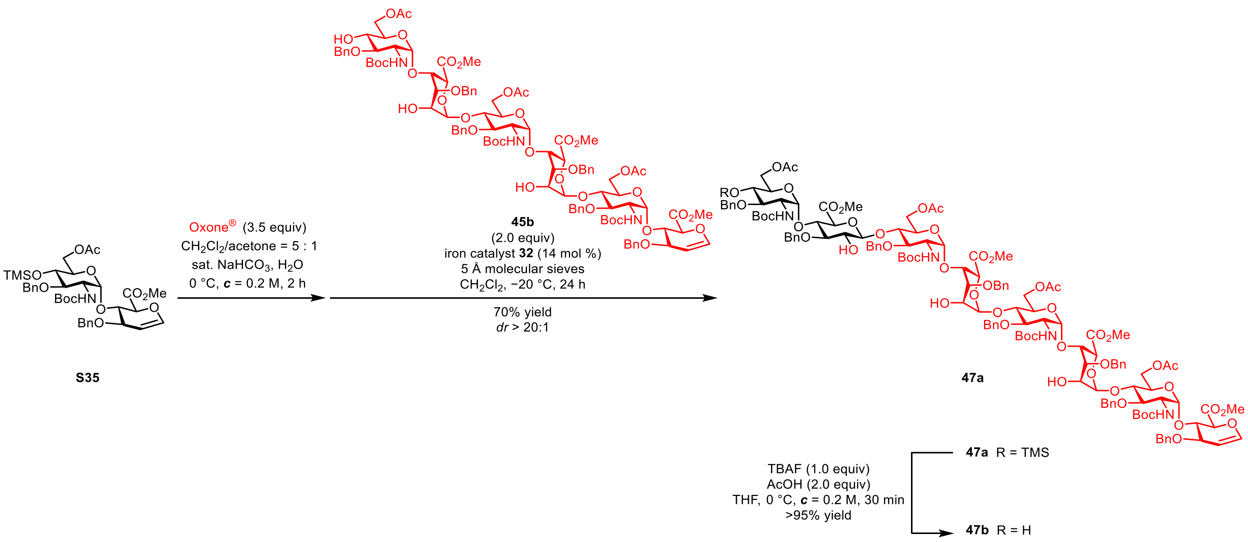

S35 was synthesized according to the following procedure.

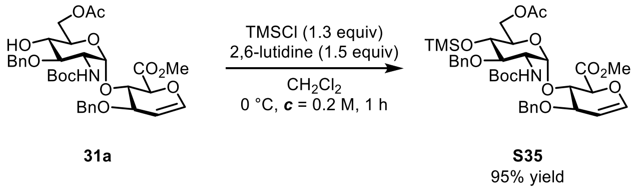

To a 100 mL flame-dried round-bottom flask equipped with a stir bar, compound **31a** (2.0 g, 3.04 mmol, 1.0 equiv), 2,6-lutidine (0.53 mL, 4.56 mmol, 1.5 equiv), and anhydrous CH_2Cl_2 (15.2 mL) were added. The mixture was cooled to 0 °C before TMSCl (0.50 mL, 3.95 mmol, 1.3 equiv) was added dropwise. The reaction mixture was then stirred at 0 °C for 1 h until the starting material **31a** was fully consumed (monitored by TLC). The reaction mixture was diluted with CH_2Cl_2 (10 mL) before saturated aqueous NH_4Cl solution (15 mL) was added to quench the reaction. The organic phase was separated from the aqueous one, which was further extracted with CH_2Cl_2 (15 mL × 2). The combined organic phase was washed with brine (20 mL) and dried over Na_2SO_4. After concentration *in vacuo*, the residue was purified through a silica gel flash column (hexanes/EtOAc: from 100:1 to 4:1) to afford the desired product **S35** (2.1 g, 95% yield) as white foam.

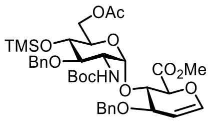

S35

Methyl 3-*O*-benzyl-4-*O*-(6-*O*-acetyl-3-*O*-benzyl-2-*tert*-butoxycarbonylamino-4-*O*-trimethylsilyl-2-deoxy-α-D-glucopyranosyl)-D-glucuronal (**S35**): $[\alpha]_D^{23}$ +67.0 (acetone, *c* = 0.5); IR ν_{max} (neat)/cm^{-1}: 2954 (w), 2359 (w), 1717 (s), 1499 (m), 1499 (m), 1249 (s), 1129 (s), 1035 (s); ^{1}H NMR (400 MHz, $CDCl_3$) δ 7.43 – 7.17 (m, 10H), 6.65 (d, *J* = 6.3 Hz, 1H), 5.04 – 4.95 (m, 2H), 4.95 – 4.87 (m, 1H), 4.70 (ABq, $\Delta\nu_{AB}$ = 34.0 Hz, *J* = 11.2 Hz, 2H), 4.51 (d, *J* = 11.3 Hz, 1H), 4.47 – 4.39 (m, 3H), 4.39 – 4.35 (m, 1H), 4.07 (dd, *J* = 12.0, 5.3 Hz, 1H), 3.96 – 3.82 (m, 2H), 3.80 – 3.69 (m, 1H), 3.66 (t, *J* = 9.2 Hz, 1H), 3.53 (s, 3H), 3.49 – 3.37 (m, 1H), 2.08 (s, 3H), 1.36 (s, 9H), 0.10 (s, 9H); ^{13}C NMR (100 MHz, $CDCl_3$) δ 170.6, 167.9, 155.1, 145.5, 138.1, 137.5, 128.3 (2C), 128.2 (2C), 127.9 (2C), 127.8, 127.6 (2C), 127.4, 98.8, 97.8, 80.4, 79.7, 75.3, 73.5, 72.7, 71.5, 71.2, 69.4, 66.6, 63.1, 54.1, 52.1, 28.3 (3C), 20.7, 0.5 (3C); HRMS: m/z (ESI) calcd for $C_{37}H_{52}NO_{12}Si^+$, $[M + H]^+$, 730.3253, found 730.3272.

To a 50 mL flask equipped with a stir bar at 0 °C, were added glycal **S35** (110 mg, 0.15 mmol, 1.0 equiv) in CH_2Cl_2/acetone mixture (v/v: 5:1, 2.5 mL) and saturated aqueous $NaHCO_3$ solution (4.3 mL), followed by addition of Oxone® ($KHSO_5 \cdot 0.5KHSO_4 \cdot 0.5K_2SO_4$) (324 mg, 0.525 mmol, 3.5 equiv) in H_2O (2.8 mL) dropwise. After stirring vigorously at 0 °C for 2 h, the reaction mixture was extracted with CH_2Cl_2 (3 mL × 3). The combined organic phase was dried over anhydrous Na_2SO_4 and concentrated *in vacuo*. The residue was further azeotropically dried with anhydrous toluene (2 mL × 3). The obtained glycal epoxide was assayed by ^{1}H NMR to get the diastereomeric ratio (*dr* > 20:1) and directly used in the next step.

To a flame-dried sealable 2-dram vial (vial **A**) equipped with a stir bar were added glycosyl acceptor **45b** (602 mg, 0.3 mmol, 2.0 equiv) and freshly activated 5 Å molecular sieves, powder (*ca.* 50 mg). After the vial was evacuated and backfilled with N_2, anhydrous CH_2Cl_2 (0.18 mL) was added. To a second flame-dried sealable 2-dram vial (vial **B**) equipped with a stir bar were added the iron porphyrin triflate catalyst **32** (0.021 mmol, 14 mol %) and freshly activated 5 Å molecular sieves, powder (*ca.* 30 mg). Vial **B** was evacuated and backfilled with N_2 three times

and anhydrous CH_2Cl_2 (0.18 mL) was added. The mixture in vial **B** was stirred at room temperature for 5 min before it was transferred to vial **A**. The mixture was stirred at −40 °C for 5 min and a solution of the aforementioned glycal epoxide in anhydrous CH_2Cl_2 (0.24 mL) was then added to the mixture at −40 °C dropwise. The reaction mixture was kept at −20 °C for 24 h and then quenched with a solution of imidazole (3 mg) in CH_2Cl_2 (1 mL) at the same temperature. The mixture was filtered through a pad of Celite® silica gel and eluted with CH_2Cl_2 (3 mL). The organic layer was then concentrated *in vacuo*, and the residue was purified through a silica gel flash column (hexanes/EtOAc: from 100:1 to 1:1) to afford the desired glycosylation product **47a** (289 mg, 70% yield).

To a 2-dram vial equipped with a stir bar were added the glycosylation product **47a** obtained from the previous step and THF (0.6 mL). The mixture was cooled to 0 °C and a premixed solution of TBAF (1 M solution in THF, 0.15 mL, 0.15 mmol, 1.0 equiv) and AcOH (17 μL, 0.3 mmol, 2.0 equiv) was then added. The reaction mixture was stirred for 30 min at 0 °C, with progress monitored until completion. The reaction mixture was then diluted with EtOAc (2 mL) before H_2O (1 mL) was added. The organic phase was separated from the aqueous one, which was further extracted with EtOAc (2 mL × 3). The combined organic phase was washed with brine (2 mL) and dried over Na_2SO_4. After concentration *in vacuo*, the residue was purified through a silica gel flash column (hexanes/acetone: from 100:1 to 3:2) to afford the desired product **47b** (281 mg, >95% yield).

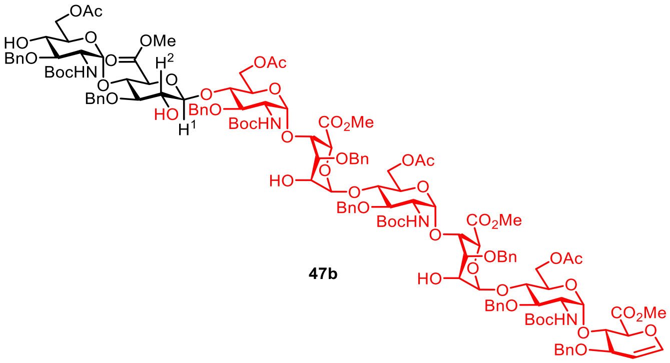

47b

Methyl 3-*O*-benzyl-4-*O*-[(6-*O*-acetyl-3-*O*-benzyl-2-*tert*-butoxycarbonylamino-2-deoxy-α-D-glucopyranosyl)-(1→4)-(methyl 3-*O*-benzyl-β-D-glucopyranosyluronate)-(1→4)-(6-*O*-acetyl-3-*O*-benzyl-2-*tert*-butoxycarbonylamino-2-deoxy-α-D-glucopyranosyl)-(1→4)-(methyl 3-*O*-benzyl-α-L-idopyranosyluronate)-(1→4)-(6-*O*-acetyl-3-*O*-benzyl-2-*tert*-

butoxycarbonylamino-2-deoxy-α-D-glucopyranosyl)-(1→4)-(methyl 3-*O*-benzyl-α-L-idopyranosyluronate)-(1→4)-(6-*O*-acetyl-3-*O*-benzyl-2-*tert*-butoxycarbonylamino-2-deoxy-α-D-glucopyranosyl)]-D-glucuronal (**47b**): $[\alpha]_D^{23}$ +20.6 (acetone, *c* = 0.1); IR ν_{max} (neat)/cm^{-1}: 2919 (w), 2359 (s), 2342 (m), 1739 (m), 1498 (m), 1366 (s), 1232 (m); ^{1}H NMR (800 MHz, acetone-d_6) δ 7.48 – 7.18 (m, 40H), 6.61 (d, *J* = 6.3 Hz, 1H), 6.31 (d, *J* = 9.6 Hz, 1H), 6.28 (d, *J* = 9.5 Hz, 1H), 6.01 (d, *J* = 10.0 Hz, 1H), 5.88 (d, *J* = 9.6 Hz, 1H), 5.49 (d, *J* = 3.8 Hz, 1H), 5.21 (d, *J* = 3.2 Hz, 1H), 5.20 – 5.10 (m, 3H), 5.08 – 5.03 (m, 4H), 5.02 (ddd, *J* = 6.5, 5.0, 1.5 Hz, 1H), 5.00 – 4.98 (m, 1H), 4.87 – 4.84 (m, 2H), 4.84 – 4.78 (m, 4H), 4.77 – 4.74 (m, 3H), 4.73 – 4.69 (m, 2H), 4.62 (d, *J* = 7.8 Hz, 1H), 4.61 (d, *J* = 10.8 Hz, 1H), 4.58 – 4.53 (m, 4H), 4.54 – 4.49 (m, 2H), 4.47 – 4.40 (m, 3H), 4.36 – 4.28 (m, 3H), 4.19 (dd, *J* = 11.9, 4.6 Hz, 1H), 4.09 – 4.04 (m, 3H), 4.03 (d, *J* = 9.5 Hz, 1H), 4.00 (t, *J* = 9.0 Hz, 1H), 3.95 (t, *J* = 5.2 Hz, 1H), 3.94 – 3.91 (m, 2H), 3.91 – 3.84 (m, 7H), 3.84 – 3.81 (m, 4H), 3.81 – 3.78 (m, 2H), 3.77 – 3.68 (m, 2H), 3.63 – 3.58 (m, 3H), 3.58 – 3.55 (m, 5H), 3.55 – 3.53 (m, 4H), 3.53 – 3.50 (m, 2H), 3.49 (s, 3H), 3.46 (s, 3H), 2.09 (s, 3H), 2.08 (s, 3H), 2.05 (s, 3H), 2.02 (s, 3H), 1.35 (s, 9H), 1.34 (s, 9H), 1.30 (s, 9H), 1.26 (s, 9H); ^{13}C NMR (100 MHz, acetone-d_6) δ 171.2, 170.94, 170.91 (two peaks overlapped, 2C), 170.42, 170.37, 169.6, 168.5, 156.5, 156.4, 156.34, 156.30, 145.8, 140.5, 140.4, 140.14, 140.11, 139.5, 139.40, 139.36 (two peaks overlapped, 2C), 129.3 (2C), 129.2 (2C), 129.11 (2C), 129.07 (2C), 129.02 (2C), 128.95 (2C), 128.9 (2C), 128.8 (2C), 128.68 (2C), 128.65 (2C), 128.6 (three peaks overlapped, 6C), 128.5, 128.39, 128.35 (two peaks overlapped, 3C), 128.34, 128.29 (two peaks overlapped, 4C), 127.9, 127.8, 127.7 (two peaks overlapped, 2C), 104.3, 101.9, 101.7, 99.8, 99.5, 98.8, 98.5, 98.4, 85.2, 81.2, 79.6, 79.5, 79.20, 79.16, 79.1, 78.9 (three peaks overlapped, 3C), 77.2, 77.083, 77.077, 76.5, 75.9, 75.8, 75.3, 75.1, 75.01 74.99, 74.9, 74.7, 74.4, 74.3, 73.8, 73.7, 73.58, 73.55, 71.8, 71.2, 70.9, 70.7, 70.6, 70.4, 70.2, 70.14, 70.10 (two peaks overlapped, 2C), 68.7, 63.5 (two peaks overlapped, 2C), 63.1, 63.0, 55.4, 55.3, 55.1, 54.9, 52.7, 52.24, 52.18, 52.16, 28.7 (3C), 28.6 (three peaks overlapped, 9C), 20.94, 20.91, 20.87, 20.8; HRMS: m/z (ESI) calcd for $C_{136}H_{172}N_4O_{51}Na^+$, $[M + Na]^+$, 2700.0881, found 2700.0827. $^{1}J^{13}{}_{C1-H1}$ = 173.9 Hz, 171.5 Hz, 171.5 Hz, 172.2 Hz, 174.7 Hz, 174.7 Hz, 161.7 Hz. $^{3}J_{H1-H2}$ = 7.8 Hz.

The stereochemistry of newly formed anomeric center (C1) of **47b** was determined by measuring ${}^{1}J_{{}^{13}C1\text{–}H1}$ (161.7 Hz) through un-decoupled HSQC experiments.[11-12] The C2 stereochemistry was determined by measuring ${}^{3}J_{H1\text{–}H2}$ (7.8 Hz).

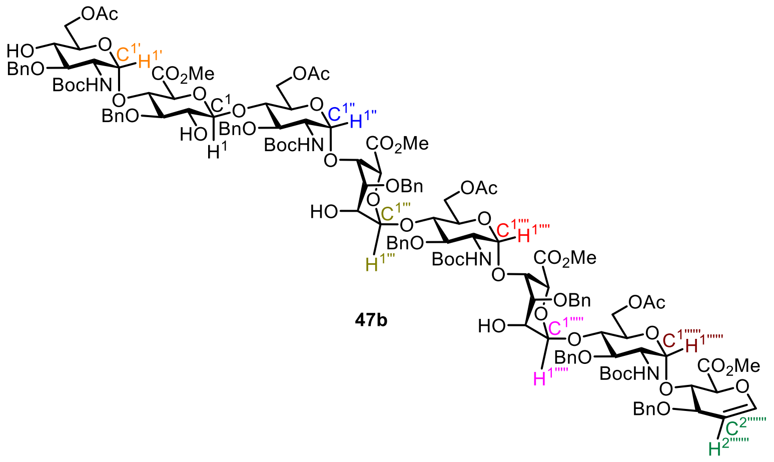

47b

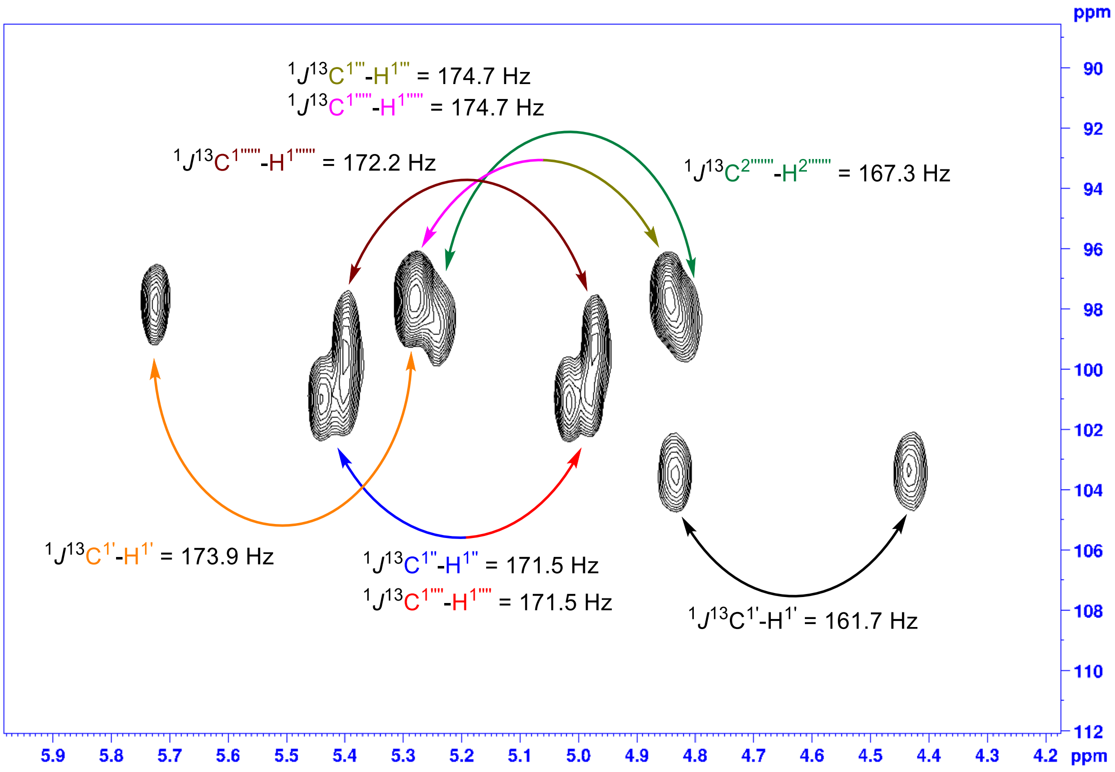

Figure S27. Un-decoupled HSQC Analysis to Determine Stereochemistry of **47b**.

A $^{1}J^{13}_{C1-H1}$ value of 161.7 Hz suggested that the newly formed glycosidic bond is in equatorial position. The $^{3}J_{H1-H2}$ value of 7.8 Hz suggested that H2 is in axial position.

b. Convergent Synthesis of Heparan Sulfate GlcN(α1-4)IdoA(α1-4)GlcN(α1-4)GlcA(β1-4)GlcN(α1-4)GlcA(β1-4)GlcN(α1-4)GlcA Module

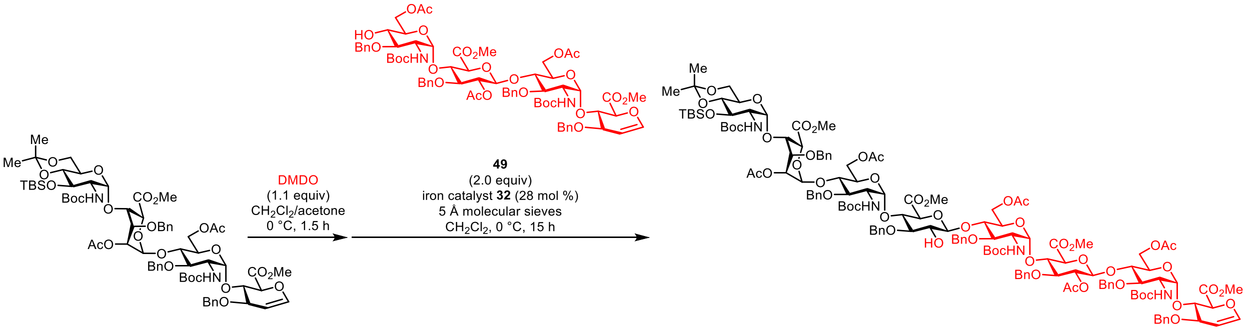

49 was synthesized according to the following procedure.

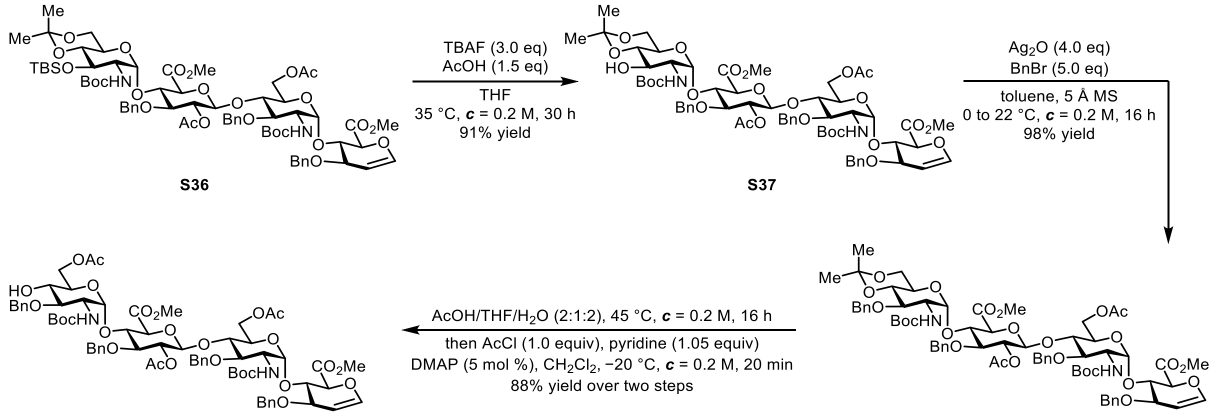

To a flame-dried 50 mL round-bottom flask equipped with a stir bar were added compound **S36** (2.4 g, 1.72 mmol, 1.0 equiv) and THF (3.4 mL). To the mixture was then added a premixed solution of TBAF (1 M solution in THF, 5.16 mL, 5.16 mmol, 3.0 equiv) and AcOH (148 μL, 2.58 mmol, 1.5 equiv). The reaction mixture was stirred for 30 h at 35 °C until the starting material **S36** was fully consumed (monitored by TLC). The reaction was then cooled down to room temperature and diluted with EtOAc (15 mL) before H_2O (10 mL) was added. The organic

phase was separated from the aqueous one, which was further extracted with EtOAc (15 mL × 3). The combined organic phase was washed with brine (10 mL) and dried over Na_2SO_4. After concentration *in vacuo*, the residue was purified through a silica gel flash column (hexanes/EtOAc: from 100:1 to 1:1) to afford the desired product **S37** (2.0 g, 91% yield) as white foam.

To a flame-dried 50 mL round-bottom flask equipped with a stir bar were added the desilylated product **S37** from the previous step (2.0 g, 1.56 mmol, 1.0 equiv), Ag_2O (1.45 g, 6.24 mmol, 4.0 equiv), freshly activated 5 Å molecular sieves (powder, *ca.* 1 g), and anhydrous toluene (7.8 mL). After the vial was evacuated and backfilled with N_2, the mixture was cooled to 0 °C and freshly distilled benzyl bromide (0.93 mL, 7.8 mmol, 5.0 equiv) was added dropwise. The reaction mixture was then stirred at room temperature for 16 h until the starting material **S37** was fully consumed (monitored by TLC). The reaction mixture was then filtered through a short pad of Celite®, further rinsed with acetone (15 mL), and concentrated *in vacuo*. The residue was purified through a silica gel flash column (hexanes/EtOAc: from 100:1 to 3:2) to afford the desired product **S38** (2.1 g, 98% yield) as white foam.

To a flame-dried 50 mL round-bottom flask equipped with a stir bar were added **S38** from the previous step (2.1 g, 1.53 mmol, 1.0 equiv) and THF (1.54 mL). AcOH (3.08 mL) and H_2O (3.08 mL) were then added sequentially to the mixture. The reaction mixture was stirred at 45 °C for 16 h until the starting material **S38** was fully consumed (monitored by TLC). The reaction was then cooled down to room temperature. After concentration *in vacuo*, the residue was azeotropically dried with anhydrous toluene (10 mL × 5) and directly used in the next step without further purification.

To a flame-dried 50 mL round-bottom flask equipped with a stir bar were added crude product from the previous step (1.53 mmol, 1.0 equiv) and DMAP (18.7 mg, 0.153 mmol, 5 mol %). After the vial was evacuated and backfilled with N_2, anhydrous CH_2Cl_2 (6.12 mL) was added. The mixture was cooled to −20 °C before anhydrous pyridine (0.13 mL, 1.61 mmol, 1.05 equiv) and AcCl (1 M in CH_2Cl_2, 1.53 mL, 1.53 mmol, 1.0 equiv) were added dropwise sequentially. The reaction mixture was stirred at −20 °C for 20 min until the starting material was fully

consumed (monitored by TLC). The reaction mixture was then quenched with H_2O (10 mL) and the organic phase was separated from the aqueous one. The aqueous phase was further extracted with CH_2Cl_2 (15 mL × 2). The combined organic phase was washed with brine (15 mL) and dried over Na_2SO_4. After concentration *in vacuo*, the residue was purified through a silica gel flash column (hexanes/EtOAc: from 100:1 to 1:1) to afford the desired product **49** (1.85 g, 88% yield over two steps) as white foam.

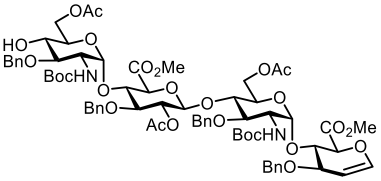

49

Methyl 3-*O*-benzyl-4-*O*-[(6-*O*-acetyl-3-*O*-benzyl-2-*tert*-butoxycarbonylamino-2-deoxy-α-D-glucopyranosyl)-(1→4)-(methyl 2-*O*-acetyl-3-*O*-benzyl-β-D-glucopyranosyluronate)-(1→4)-(6-*O*-acetyl-3-*O*-benzyl-2-*tert*-butoxycarbonylamino-2-deoxy-α-D-glucopyranosyl)]-D-glucuronal (**49**): $[\alpha]_D^{23}$ +47.9 (acetone, *c* = 1.0); IR ν_{max} (neat)/cm^{-1}: 2972 (w), 2361 (m), 1742 (s), 1506 (m), 1367 (s), 1237 (s), 1032 (s); ^{1}H NMR (400 MHz, acetone-d_6) δ 7.40 – 7.35 (m, 4H), 7.34 – 7.31 (m, 9H), 7.30 – 7.27 (m, 5H), 7.26 – 7.21 (m, 2H), 6.58 (d, *J* = 6.3 Hz, 1H), 5.92 (d, *J* = 5.3 Hz, 1H), 5.89 (d, *J* = 5.4 Hz, 1H), 5.42 (d, *J* = 3.7 Hz, 1H), 5.16 (d, *J* = 3.7 Hz, 1H), 5.09 – 5.03 (m, 2H), 5.01 – 4.96 (m, 1H), 4.95 (d, *J* = 3.1 Hz, 1H), 4.88 (d, *J* = 3.6 Hz, 1H), 4.86 (s, 1H), 4.77 (d, *J* = 10.6 Hz, 1H), 4.74 – 4.67 (m, 2H), 4.61 – 4.52 (m, 3H), 4.49 (d, *J* = 11.5 Hz, 1H), 4.42 – 4.37 (m, 1H), 4.32 (d, *J* = 1.9 Hz, 1H), 4.25 – 4.19 (m, 2H), 4.17 (d, *J* = 8.5 Hz, 1H), 4.14 (s, 1H), 4.13 – 4.06 (m, 1H), 3.98 (td, *J* = 7.5, 3.8 Hz, 1H), 3.90 (t, *J* = 3.7 Hz, 1H), 3.87 – 3.75 (m, 3H), 3.71 – 3.61 (m, 2H), 3.59 (s, 3H), 3.57 (s, 1H), 3.55 (s, 3H), 3.54 – 3.48 (m, 2H), 2.07 (s, 3H), 2.05 (s, 3H), 2.02 (s, 3H), 1.363 (s, 9H), 1.355 (s, 9H); ^{13}C NMR (100 MHz, $CDCl_3$) δ 171.7, 170.6, 169.2, 168.0, 167.8, 155.2 (two peaks overlapped, 2C), 145.3, 138.6, 138.3, 137.5, 137.0, 128.5 (2C), 128.3 (two peaks overlapped, 4C), 128.1 (2C), 127.9 (two peaks overlapped, 4C), 127.8 (two peaks overlapped, 2C), 127.72, 127.66, 127.5 (two peaks overlapped, 3C), 127.2, 101.1, 98.6, 98.5, 98.2, 81.8, 79.83, 79.77, 79.5, 78.5, 78.0, 75.0, 74.7, 74.6, 74.5, 74.2, 73.5, 73.2, 73.0, 71.1, 70.0, 69.6, 69.4, 67.3, 62.6, 62.2, 53.8, 53.4, 52.6, 52.1, 28.32 (3C), 28.25 (3C), 20.8 (two peaks overlapped, 2C), 20.6; HRMS: m/z (ESI) calcd for $C_{70}H_{89}N_2O_{26}^+$, $[M + H]^+$, 1373.5698, found 1373.5731.

To a 2-dram vial equipped with a stir bar at 0 °C, were added glycal **48** (140 mg, 0.1 mmol, 1.0 equiv) in CH_2Cl_2 (0.2 mL), followed by the addition of freshly prepared dimethyldioxirane (DMDO in acetone, 0.06 M, 1.83 mL, 0.11 mmol, 1.1 equiv)[16] dropwise. After stirring at 0 °C for 1.5 h, the reaction mixture was concentrated *in vacuo*, then the residue was re-dissolved in anhydrous CH_2Cl_2 (2 mL) and dried over anhydrous Na_2SO_4. The organic phase was concentrated *in vacuo* and the residue was further dried azeotropically with anhydrous toluene (2 mL × 3). The obtained glycal epoxide was assayed by ^{1}H NMR to get the diastereomeric ratio (*dr* > 20:1) and directly used in the next step.

Stereochemistry Determination of Glycal Epoxide S39

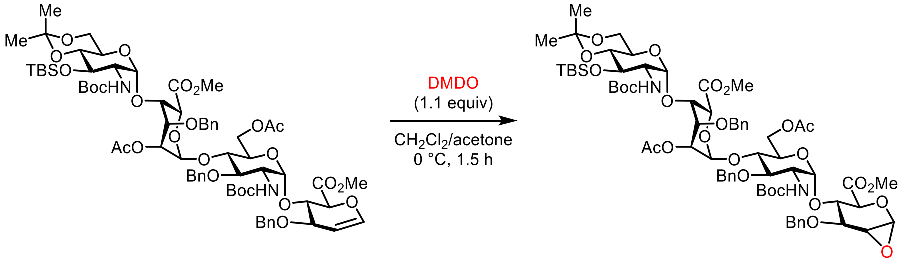

48 **S39**

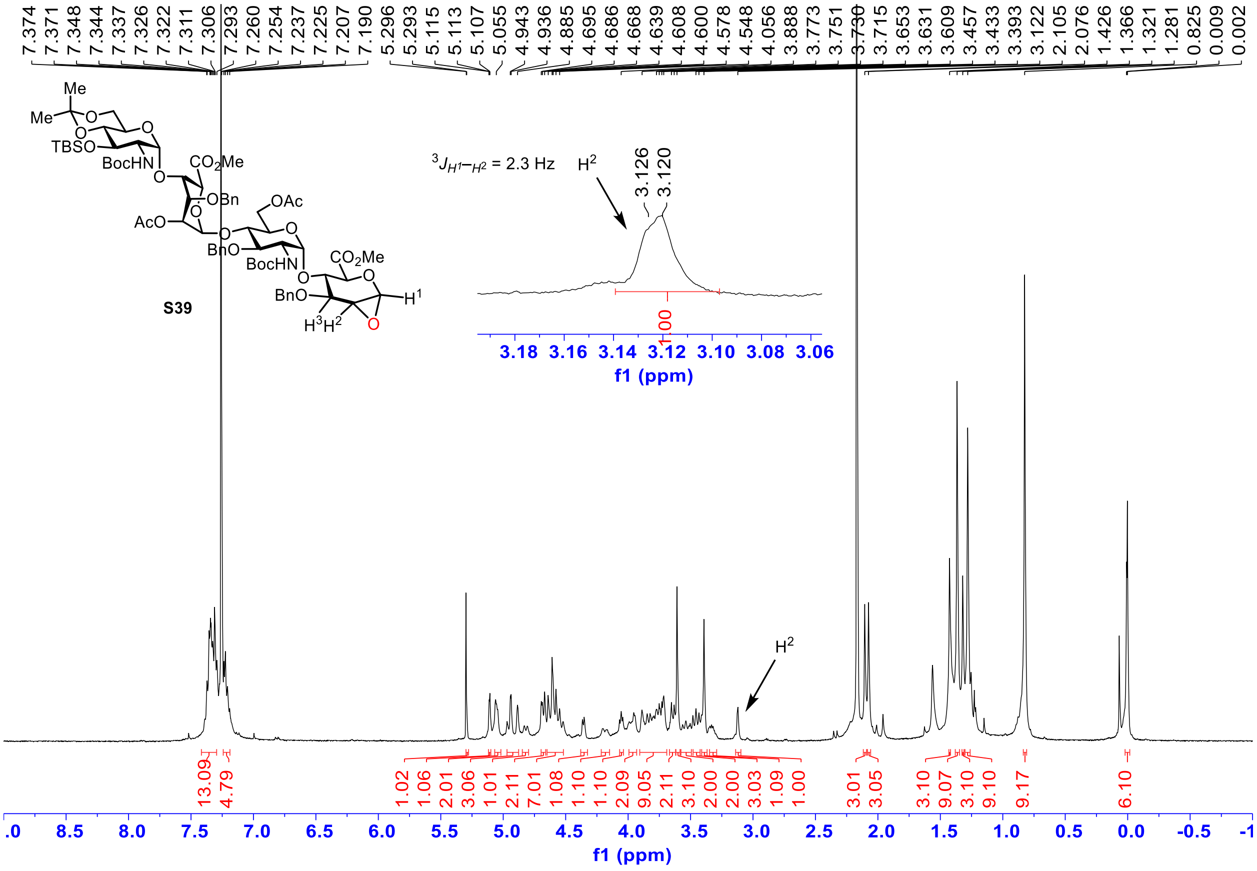

Figure S28. Stereochemistry Determination of Glycal Epoxide **S39**.

Analogous to glucuronate ester α-epoxide **S20**, the C(2)–H of glycal epoxide **S39** appears at 3.12 ppm with ${}^3J_{H1-H2}$ = 2.3 Hz, indicating an α-epoxide (*dr* > 20:1). This assignment was further corroborated by the stereochemistry analysis of the glycosylation product 1,2-*trans*-β-glycoside **50**.

To a flame-dried sealable 2-dram vial (vial **A**) equipped with a stir bar were added glycosyl acceptor **49** (275 mg, 0.2 mmol, 2.0 equiv) and freshly activated 5 Å molecular sieves, powder (*ca.* 50 mg). After the vial was evacuated and backfilled with N_2, anhydrous CH_2Cl_2 (0.2 mL) was added. To a second flame-dried sealable 2-dram vial (vial **B**) equipped with a stir bar were added the iron porphyrin triflate catalyst **32** (0.028 mmol, 28 mol %) and freshly activated 5 Å

molecular sieves, powder (*ca.* 30 mg). Vial **B** was evacuated and backfilled with N_2 three times and anhydrous CH_2Cl_2 (0.27 mL) was added. The mixture in vial **B** was stirred at room temperature for 5 min before it was transferred to vial **A**. The mixture was stirred at −40 °C for 5 min and a solution of the aforementioned glycal epoxide in anhydrous CH_2Cl_2 (0.20 mL) was then added to the mixture at −40 °C dropwise. The reaction mixture was kept at −20 °C for 24 h and then quenched with a solution of imidazole (3 mg) in CH_2Cl_2 (1 mL) at the same temperature. The mixture was filtered through a pad of Celite® silica gel and eluted with CH_2Cl_2 (3 mL). The organic layer was then concentrated *in vacuo*, and the residue was purified through a silica gel flash column (hexanes/ethyl acetate: from 100:1 to 1:1) to afford the desired glycosylation product **50** (195 mg, 70% yield) as white foam.

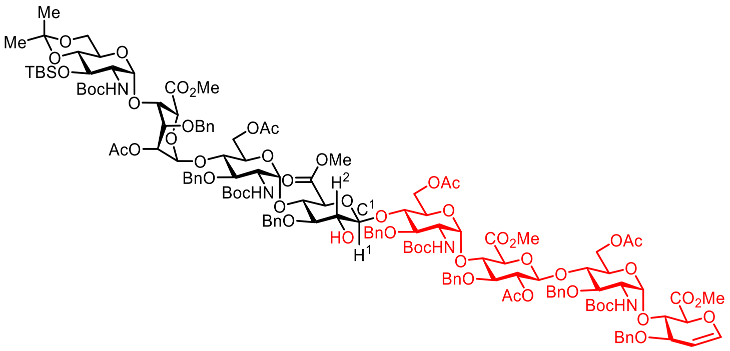

50

Methyl 3-*O*-benzyl-4-*O*-[(2-*tert*-butoxycarbonylamino-3-*O*-*tert*-butyldimethylsilyl-4,6-*O*-isopropylidene-2-deoxy-α-D-glucopyranosyl)-(1→4)-(methyl 2-*O*-acetyl-3-*O*-benzyl-α-L-idopyranosyluronate)-(1→4)-(6-*O*-acetyl-3-*O*-benzyl-2-*tert*-butoxycarbonylamino-2-deoxy-α-D-glucopyranosyl)-(1→4)-(methyl 3-*O*-benzyl-β-D-glucopyranosyluronate)-(1→4)-(6-*O*-acetyl-3-*O*-benzyl-2-*tert*-butoxycarbonylamino-2-deoxy-α-D-glucopyranosyl)-(1→4)-(methyl 2-*O*-acetyl-3-*O*-benzyl-β-D-glucopyranosyluronate)-(1→4)-(6-*O*-acetyl-3-*O*-benzyl-2-*tert*-butoxycarbonylamino-2-deoxy-α-D-glucopyranosyl)]-D-glucuronal (**50**): $[\alpha]_D^{23}$ +36.4 (acetone, *c* = 0.4); IR ν_{max} (neat)/cm^{-1}: 2970 (w), 2360 (s), 1739 (s), 1366 (s), 1229 (s), 1216 (s), 1027 (s); ^{1}H NMR (800 MHz, acetone-d_6) δ 7.43 (d, *J* = 7.6 Hz, 2H), 7.40 – 7.36 (m, 8H), 7.35 – 7.26 (m, 22H), 7.25 – 7.20 (m, 3H), 6.57 (d, *J* = 6.3 Hz, 1H), 6.09 (d, *J* = 9.9 Hz, 1H), 5.95 – 5.84 (m, 2H), 5.58 (d, *J* = 10.1 Hz, 1H), 5.50 (d, *J* = 3.7 Hz, 1H), 5.41 (d, *J* = 3.8 Hz, 1H), 5.16 (d, *J* = 3.7 Hz, 1H), 5.11 (s, 1H), 5.06 (d, *J* = 5.8 Hz, 1H), 5.06 – 5.04 (m, 2H), 5.03 (d, *J* = 2.7 Hz, 1H), 5.00 – 4.97 (m, 1H), 4.96 (t, *J* = 3.4 Hz, 1H), 4.94 (d, *J* = 3.2 Hz, 1H), 4.91 (d, *J* = 3.8 Hz, 1H), 4.86 – 4.83 (m, 2H), 4.79 – 4.76 (m, 2H),

4.76 – 4.74 (m, 2H), 4.73 (d, J = 10.1 Hz, 1H), 4.68 (d, J = 10.6 Hz, 1H), 4.63 – 4.60 (m, 2H), 4.58 (d, J = 11.2 Hz, 1H), 4.56 – 4.50 (m, 4H), 4.49 (d, J = 11.5 Hz, 1H), 4.41 (dd, J = 12.2, 4.1 Hz, 1H), 4.40 – 4.37 (m, 2H), 4.25 (dd, J = 12.4, 3.1 Hz, 1H), 4.22 (dd, J = 11.8, 6.4 Hz, 1H), 4.16 – 4.10 (m, 2H), 4.06 (d, J = 8.7 Hz, 1H), 4.05 – 4.02 (m, 2H), 4.01 – 3.96 (m, 2H), 3.94 (t, J = 4.1 Hz, 1H), 3.93 – 3.89 (m, 2H), 3.89 – 3.84 (m, 3H), 3.83 – 3.80 (m, 2H), 3.80 – 3.74 (m, 3H), 3.71 (td, J = 8.6, 3.8 Hz, 1H), 3.69 – 3.66 (m, 1H), 3.64 – 3.57 (m, 6H), 3.56 (s, 3H), 3.55 (s, 3H), 3.54 – 3.53 (m, 1H), 3.52 – 3.50 (m, 2H), 3.49 (s, 3H), 3.46 (s, 3H), 2.11 (s, 3H), 2.09 (s, 3H), 2.08 (s, 3H), 2.07 (s, 3H), 2.03 (s, 3H), 1.49 (s, 3H), 1.364 (s, 9H), 1.359 (s, 9H), 1.35 (s, 3H), 1.32 (s, 9H), 1.26 (s, 9H), 0.87 (s, 9H), 0.08 (s, 3H), 0.07 (s, 3H); ^{13}C NMR (100 MHz, acetone-d_6) δ 171.0, 170.9, 170.8, 170.2, 169.8 (two peaks overlapped, 2C), 169.7, 169.3, 168.6, 156.31, 156.28, 156.2, 155.9, 145.8, 140.5, 140.4, 139.8, 139.3, 139.2, 138.9, 138.6, 129.2 (two peaks overlapped, 4C), 129.2 (2C), 129.1 (2C), 129.04 (2C), 129.03 (2C), 128.9 (2C), 128.71 (2C), 128.69 (2C), 128.64 (two peaks overlapped, 4C), 128.63 (2C), 128.60 (2C), 128.56 (2C), 128.4, 128.32, 128.30 (two peaks overlapped, 2C), 127.8, 127.7 (two peaks overlapped, 2C), 104.5, 101.8, 99.9, 99.7, 99.5, 99.4, 98.7, 98.4 (two peaks overlapped, 2C), 85.2, 83.2, 79.7, 79.5, 79.32, 79.31, 79.27, 79.2, 79.10, 79.06, 76.1, 75.80, 75.76, 75.7, 75.4, 75.30, 75.28, 75.2, 75.14, 75.08, 75.0, 74.7, 74.6, 74.5, 74.4, 74.1 (two peaks overlapped, 2C), 73.6, 73.3, 72.1, 70.8, 70.6, 70.5, 70.0, 69.6, 69.4, 69.0, 65.2, 63.3, 62.8, 62.7, 62.4, 56.4, 55.3, 55.0, 54.9, 53.1, 52.7, 52.2, 52.1, 29.6 (2C), 28.68 (3C), 28.66 (3C), 28.6 (two peaks overlapped, 6C), 26.3 (3C), 21.4, 20.93, 20.88, 20.8, 19.3, 18.9, -3.7, -4.7; HRMS: m/z (ESI) calcd for $C_{140}H_{186}N_4O_{52}SiNa^+$, $[M + Na]^+$, 2806.1695, found 2806.1622. $^1J_{^{13}C1-H1}$ = 175.2 Hz, 175.7 Hz, 171.3 Hz, 172.3 Hz, 170.4 Hz, 162.6 Hz, 162.2 Hz.

The stereochemistry of newly formed anomeric center (C1) of **50** was determined by measuring $^1J_{^{13}C1-H1}$ (162.2 Hz) through un-decoupled HSQC experiments.[11-12] The C2 stereochemistry was determined by measuring $^3J_{H1-H2}$ (7.8 Hz).

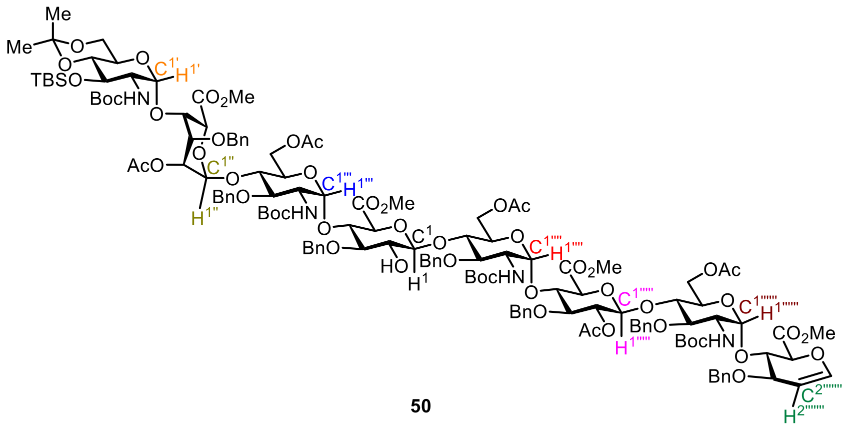

50

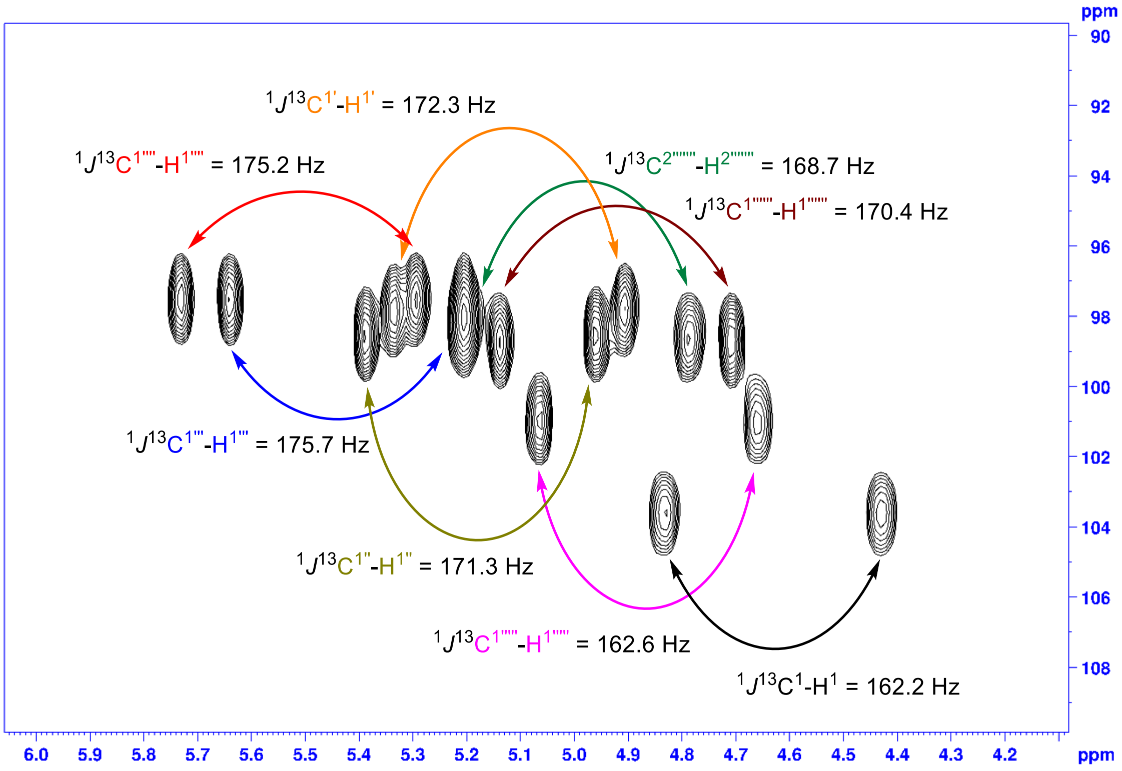

Figure S29. Un-decoupled HSQC Analysis to Determine Stereochemistry of **50**.

A $^{1}J^{13}{}_{\mathrm{C1-H1}}$ value of 162.2 Hz suggested that the newly formed glycosidic bond is in equatorial position, which confirmed the generation of a 1,2-*trans*-β-glycosidic linkage.

c. Convergent Synthesis of Heparan Sulfate GlcN(α1-4)GlcA(β1-4)GlcN(α1-4)IdoA(α1-4)GlcN(α1-4)IdoA(α1-4)GlcN(α1-4)GlcA Module

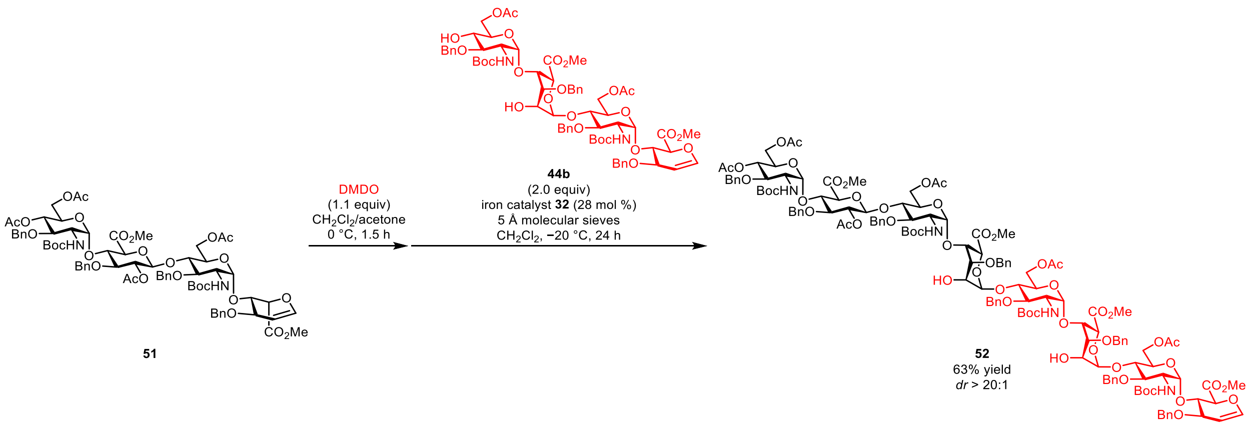

51 was synthesized according to the following procedure.

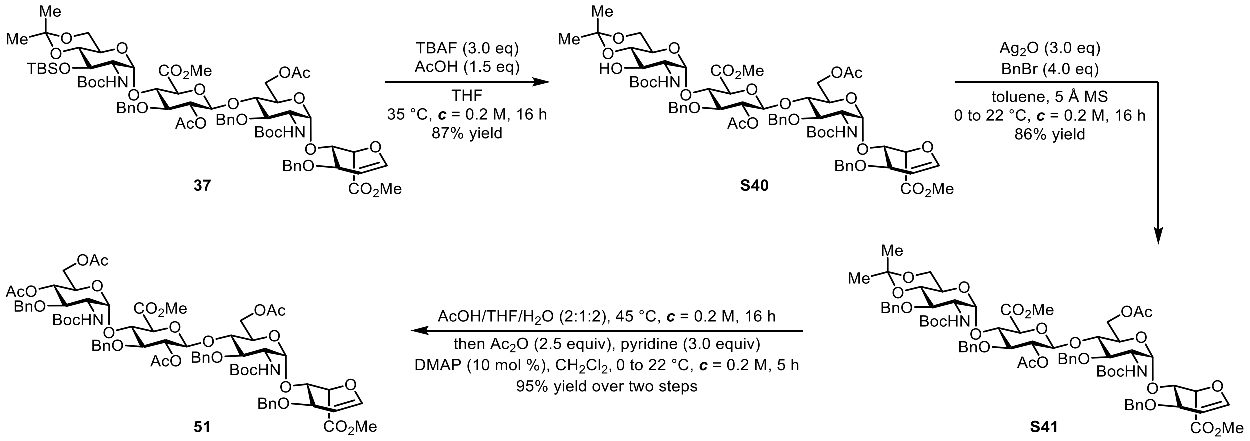

To a sealable 2-dram vial equipped with a stir bar were added compound **37** (300 mg, 0.215 mmol, 1.0 equiv) and THF (0.43 mL). To the mixture was then added a premixed solution of TBAF (1 M solution in THF, 0.645 mL, 0.645 mmol, 3.0 equiv) and AcOH (19 μL, 0.322 mmol, 1.5 equiv). The reaction mixture was stirred for 15 h at 35 °C until the starting material **37** was fully consumed (monitored by TLC). The reaction was then cooled down to room temperature and diluted with EtOAc (3 mL) before H_2O (2 mL) was added. The organic phase was separated from the aqueous one, which was further extracted with EtOAc (3 mL × 3). The combined organic phase was washed with brine (3 mL) and dried over Na_2SO_4. After concentration *in vacuo*, the residue was purified through a silica gel flash column (hexanes/EtOAc: from 100:1 to 2:3) to afford the desired product **S40** (240 mg, 87% yield) as white foam.

To a flame-dried sealable 2-dram vial equipped with a stir bar were added the desilylated product **S40** from the previous step (240 mg, 0.187 mmol, 1.0 equiv), Ag_2O (130 mg, 0.562 mmol, 3.0 equiv), freshly activated 5 Å molecular sieves (powder, *ca*. 100 mg), and anhydrous toluene (0.94 mL). After the vial was evacuated and backfilled with N_2, the mixture was cooled to 0 °C and freshly distilled benzyl bromide (89 μL, 0.748 mmol, 4.0 equiv) was added dropwise. The reaction mixture was then stirred at room temperature for 16 h until the starting material **S40** was fully consumed (monitored by TLC). The reaction mixture was then filtered through a short pad of Celite®, further rinsed with acetone (3 mL), and concentrated *in vacuo*. The residue was purified through a silica gel flash column (hexanes/EtOAc: from 100:1 to 2:1) to afford the desired product **S41** (221 mg, 86% yield) as white foam.

To a sealable 2-dram vial equipped with a stir bar were added **S41** from the previous step (221 mg, 0.161 mmol, 1.0 equiv) and THF (0.16 mL). AcOH (0.32 mL) and H_2O (0.32 mL) were then added sequentially to the mixture. The reaction mixture was stirred at 45 °C for 16 h until the starting material **S41** was fully consumed (monitored by TLC). The reaction was then cooled down to room temperature. After concentration *in vacuo*, the residue was azeotropically dried with anhydrous toluene (3 mL × 5) and directly used in the next step without further purification.

To a flame-dried sealable 2-dram vial equipped with a stir bar were added crude product from the previous step (0.161 mmol, 1.0 equiv) and DMAP (2.0 mg, 0.016 mmol, 10 mol %). After the vial was evacuated and backfilled with N_2, anhydrous CH_2Cl_2 (0.81 mL) was added. The solution was then cooled to 0 °C before pyridine (39 μL, 0.483 mmol, 3.0 equiv) and Ac_2O (38 μL, 0.403 mmol, 2.5 equiv) were added dropwise sequentially. The reaction mixture was gradually warmed to room temperature and stirred for 5 h, with progress monitored by TLC until completion. The reaction mixture was then diluted with CH_2Cl_2 (2 mL) and quenched with saturated aqueous NH_4Cl solution (1.5 mL). The organic phase was separated from the aqueous one, which was further extracted with CH_2Cl_2 (3 mL × 3). The combined organic phase was dried over Na_2SO_4. After concentration *in vacuo*, the residue was purified through a silica gel flash column (hexanes/EtOAc: from 100:1 to 3:2) to afford the desired product **51** (217 mg, 95% yield over two steps) as white foam.

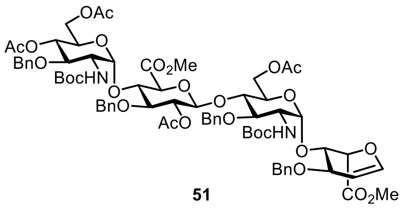

51

Methyl 3-*O*-benzyl-4-*O*-[(4,6-di-*O*-acetyl-3-*O*-benzyl-2-*tert*-butoxycarbonylamino-2-deoxy-α-D-glucopyranosyl)-(1→4)-(methyl 2-*O*-acetyl-3-*O*-benzyl-β-D-glucopyranosyluronate)-(1→4)-(6-*O*-acetyl-3-*O*-benzyl-2-*tert*-butoxycarbonylamino-2-deoxy-α-D-glucopyranosyl)]-L-guluronal (**51**): $[\alpha]_D^{21}$ +12.8 (acetone, *c* = 4.0); IR ν_{max} (neat)/cm^{-1}: 2974 (w), 1742 (s), 1713 (s), 1499 (m), 1366 (m), 1218 (s), 1030 (s); ^{1}H NMR (400 MHz, acetone-d_6) δ 7.47 – 7.16 (m, 20H), 6.61 (d, *J* = 6.1 Hz, 1H), 6.12 (d, *J* = 9.7 Hz, 1H), 5.55 (d, *J* = 9.1 Hz, 1H), 5.50 (d, *J* = 3.7 Hz, 1H), 5.10 – 5.04 (m, 2H), 5.04 – 4.99 (m, 2H), 4.94 (d, *J* = 3.6 Hz, 1H), 4.84 (d, *J* = 7.9 Hz, 1H), 4.78 (d, *J* = 10.6 Hz, 1H), 4.74 – 4.68 (m, 3H), 4.67 – 4.62 (m, 2H), 4.61 – 4.55 (m, 2H), 4.51 (dd, *J* = 12.0, 2.0 Hz, 1H), 4.35 – 4.29 (m, 1H), 4.26 (dd, *J* = 12.1, 4.4 Hz, 1H), 4.20 – 4.14 (m, 2H), 4.13 – 4.10 (m, 1H), 4.09 – 4.02 (m, 2H), 3.98 (td, *J* = 10.2, 3.6 Hz, 1H), 3.91 (dd, *J* = 5.3, 2.3 Hz, 1H), 3.88 – 3.81 (m, 1H), 3.79 (s, 3H), 3.77 – 3.70 (m, 2H), 3.67 (ddd, *J* = 10.5, 4.4, 2.6 Hz, 2H), 3.60 (s, 3H), 3.57 (dd, *J* = 10.4, 1.9 Hz, 1H), 2.12 (s, 3H), 2.10 (s, 3H), 2.01 (s, 3H), 2.01 (s, 3H), 1.36 (s, 9H), 1.35 (s, 9H); ^{13}C NMR (100 MHz, acetone-d_6) δ 171.0, 170.8, 170.0, 169.8, 169.5, 169.3, 156.2, 156.2, 146.4, 140.5, 139.6, 139.5, 138.6, 129.22 (2C), 129.17 (2C), 129.1 (2C), 129.0 (2C), 128.7 (three peaks overlapped, 6C), 128.46 (2C), 128.45, 128.4, 128.2, 127.8, 101.8, 99.8, 98.5, 97.1, 83.1, 79.6, 79.2, 79.1, 78.8, 78.5, 75.3, 75.22, 75.18, 75.0, 74.4, 74.2, 73.3, 72.9, 70.8, 70.6, 70.5, 69.8, 67.4, 62.7 (two peaks overlapped, 2C), 55.0, 54.9, 52.9, 52.7, 28.7 (3C), 28.6 (3C), 21.0, 20.9 (two peak overlapped, 2C), 20.7; HRMS: m/z (ESI) calcd for $C_{72}H_{91}N_2O_{27}^+$, $[M + H]^+$, 1415.5804, found 1415.5784.

To a 2-dram vial equipped with a stir bar at 0 °C, were added glycal **51** (142 mg, 0.1 mmol, 1.0 equiv) in CH_2Cl_2 (0.2 mL), followed by the addition of freshly prepared dimethyldioxirane (DMDO in acetone, 0.06 M, 1.83 mL, 0.11 mmol, 1.1 equiv)[16] dropwise. After stirring at 0 °C for 1.5 h, the reaction mixture was concentrated *in vacuo*, then the residue was re-dissolved in anhydrous CH_2Cl_2 (2 mL) and dried over anhydrous Na_2SO_4. The organic phase was concentrated *in vacuo* and the residue was further dried azeotropically with anhydrous toluene (2

mL × 3). The obtained glycal epoxide was assayed by ^{1}H NMR to get the diastereomeric ratio (*dr* > 20:1) and directly used in the next step.

Stereochemistry Determination of Glycal Epoxide S42

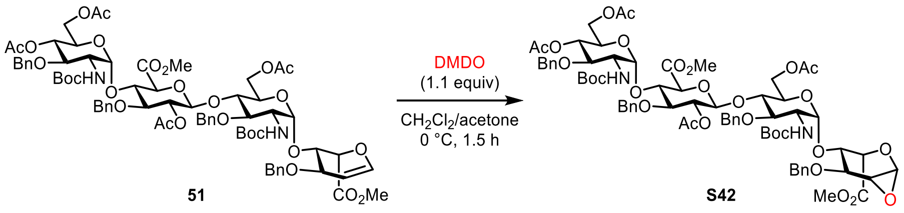

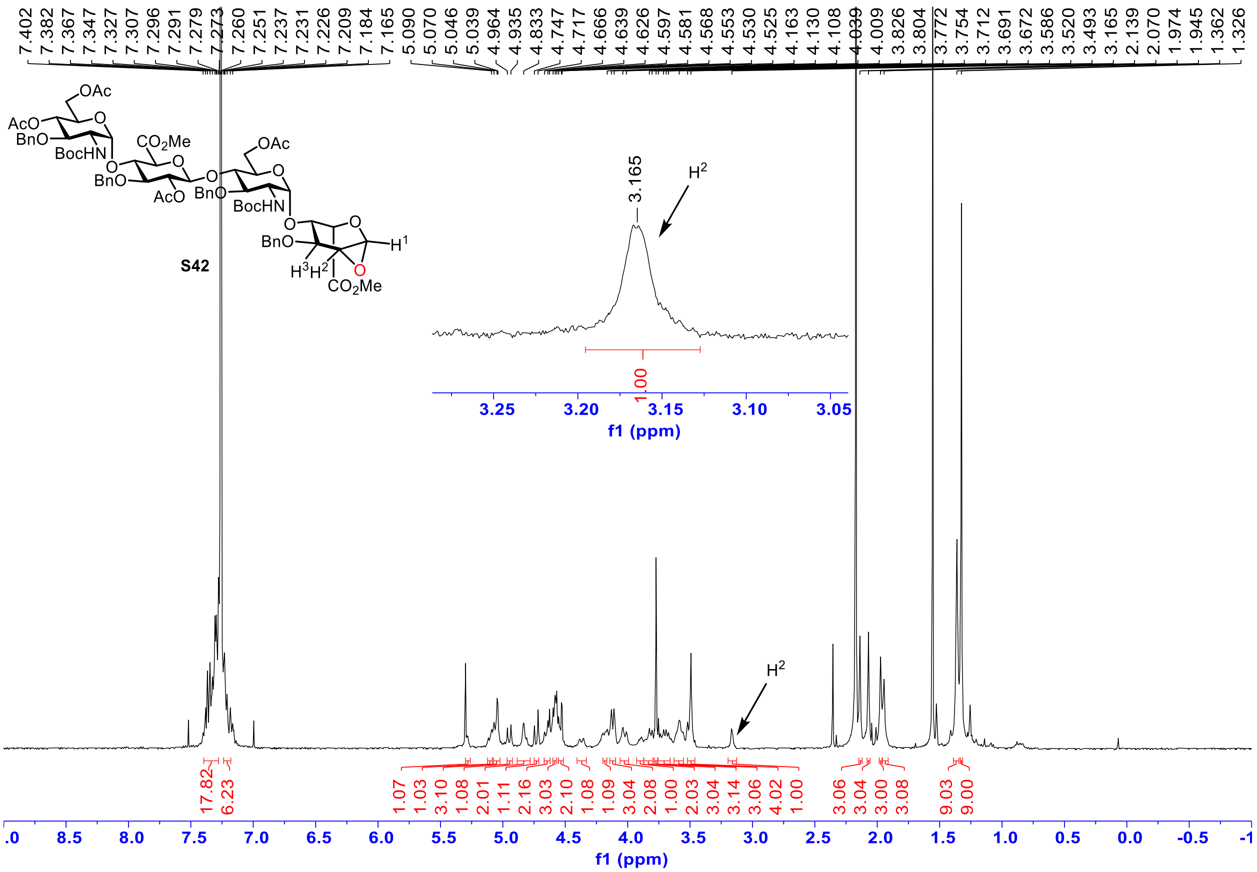

Figure S30. Stereochemistry Determination of Glycal Epoxide **S42**.

Analogous to glucuronate ester α-epoxides, a typical iduronate ester β-epoxide is expected to have a dihedral angle ($\Phi_{1,2}$) of approximately 0° and a dihedral angle ($\Phi_{2,3}$) of about 90°. This conformation results in a small $^3J_{H1\text{–}H2}$ value and a negligible $^3J_{H2\text{–}H3}$ (~0 Hz). Consequently, the

H2 resonance of an iduronate ester α-epoxide appears as a doublet, or as a broad singlet when $^3J_{H1-H2}$ value is too small to be resolved.

For glycal epoxide **S42**, the H2 resonance was observed as a broad singlet in the ^{1}H NMR spectrum, consistent with an α-epoxide assignment (*dr* > 20:1). This assignment was further corroborated by the stereochemistry analysis of the glycosylation product 1,2-*trans*-α-glycoside **52**.

To a flame-dried sealable 2-dram vial (vial **A**) equipped with a stir bar were added glycosyl acceptor **44b** (266 mg, 0.2 mmol, 2.0 equiv) and freshly activated 5 Å molecular sieves, powder (*ca.* 50 mg). After the vial was evacuated and backfilled with N_2, anhydrous CH_2Cl_2 (0.2 mL) was added. To a second flame-dried sealable 2-dram vial (vial **B**) equipped with a stir bar were added the iron porphyrin triflate catalyst **32** (0.028 mmol, 28 mol %) and freshly activated 5 Å molecular sieves, powder (*ca.* 30 mg). Vial **B** was evacuated and backfilled with N_2 three times and anhydrous CH_2Cl_2 (0.27 mL) was added. The mixture in vial **B** was stirred at room temperature for 5 min before it was transferred to vial **A**. The mixture was stirred at −40 °C for 5 min and a solution of the aforementioned glycal epoxide in anhydrous CH_2Cl_2 (0.20 mL) was then added to the mixture at −40 °C dropwise. The reaction mixture was kept at −20 °C for 24 h and then quenched with a solution of imidazole (3 mg) in CH_2Cl_2 (1 mL) at the same temperature. The mixture was filtered through a pad of Celite® silica gel and eluted with CH_2Cl_2 (3 mL). The organic layer was then concentrated *in vacuo*, and the residue was purified through a silica gel flash column (hexanes/acetone: from 100:1 to 1:1) to afford the desired glycosylation product **52** (174 mg, 63% yield) as white foam.

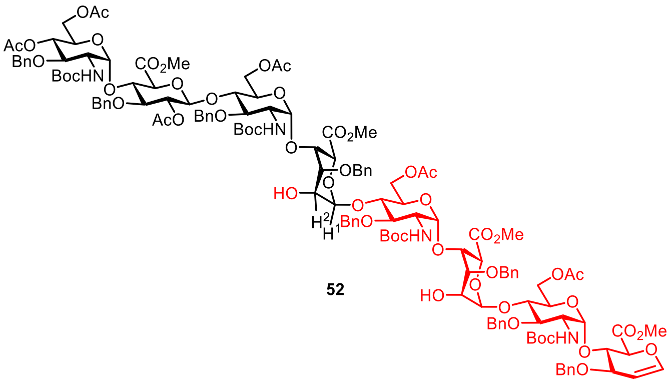

52

Methyl 3-*O*-benzyl-4-*O*-[(4,6-di-*O*-acetyl-3-*O*-benzyl-2-*tert*-butoxycarbonylamino-2-deoxy-α-D-glucopyranosyl)-(1→4)-(methyl 2-*O*-acetyl-3-*O*-benzyl-β-D-glucopyranosyluronate)-(1→4)-(6-*O*-acetyl-3-*O*-benzyl-2-*tert*-butoxycarbonylamino-2-deoxy-α-D-glucopyranosyl)-(1→4)-(methyl 3-*O*-benzyl-α-L-idopyranosyluronate)-(1→4)-(6-*O*-acetyl-3-*O*-benzyl-2-*tert*-butoxycarbonylamino-2-deoxy-α-D-glucopyranosyl)-(1→4)-(methyl 3-*O*-benzyl-α-L-idopyranosyluronate)-(1→4)-(6-*O*-acetyl-3-*O*-benzyl-2-*tert*-butoxycarbonylamino-2-deoxy-α-D-glucopyranosyl)]-D-glucuronal (52): $[\alpha]_D^{22}$ +22.8 (acetone, *c* = 0.2); IR ν_{max} (neat)/cm^{-1}: 2925 (w), 1741 (s), 1712 (s), 1499 (w), 1366 (m), 1219 (s), 1028 (s); ^{1}H NMR (800 MHz, acetone-d_6) δ 7.58 – 7.07 (m, 40H), 6.61 (d, *J* = 6.4 Hz, 1H), 6.31 (d, *J* = 9.6 Hz, 1H), 6.28 (d, *J* = 9.5 Hz, 1H), 6.13 (d, *J* = 9.9 Hz, 1H), 5.88 (d, *J* = 9.6 Hz, 1H), 5.51 (d, *J* = 3.7 Hz, 1H), 5.27 (d, *J* = 5.2 Hz, 1H), 5.19 (d, *J* = 3.9 Hz, 1H), 5.16 (d, *J* = 3.8 Hz, 1H), 5.12 – 5.04 (m, 4H), 5.04 – 5.00 (m, 2H), 5.00 – 4.98 (m, 1H), 4.89 – 4.83 (m, 4H), 4.83 – 4.79 (m, 3H), 4.78 – 4.74 (m, 4H), 4.73 – 4.71 (m, 2H), 4.55 (ABq, $\Delta\nu_{AB}$ = 42.8 Hz, *J* = 11.2 Hz, 2H), 4.62 – 4.58 (m, 2H), 4.58 – 4.53 (m, 3H), 4.51 (d, *J* = 11.5 Hz, 1H), 4.47 – 4.44 (m, 1H), 4.44 – 4.42 (m, 1H), 4.41 – 4.38 (m, 2H), 4.31 (dd, *J* = 12.1, 5.2 Hz, 1H), 4.27 (dd, *J* = 12.0, 5.0 Hz, 1H), 4.20 – 4.13 (m, 2H), 4.13 – 4.08 (m, 2H), 4.07 – 4.01 (m, 3H), 4.01 – 3.96 (m, 1H), 3.95 – 3.92 (m, 3H), 3.91 – 3.87 (m, 3H), 3.86 – 3.82 (m, 4H), 3.81 – 3.74 (m, 3H), 3.71 (t, *J* = 9.8 Hz, 1H), 3.69 – 3.64 (m, 2H), 3.64 – 3.56 (m, 7H), 3.55 (s, 3H), 3.46 (s, 3H), 2.10 (s, 3H), 2.08 (s, 3H), 2.06 (s, 3H), 2.02 (s, 3H), 2.01 (s, 6H), 1.37 (s, 9H), 1.34 (s, 9H), 1.28 (s, 9H), 1.25 (s, 9H); ^{13}C NMR (100 MHz, acetone-d_6) δ 171.0 (two peaks overlapped, 2C), 170.9 (two peaks overlapped, 2C), 170.8, 170.4, 170.0, 169.9, 169.3, 168.6, 156.4 (two peaks overlapped, 2C), 156.31, 156.26, 145.8, 140.5, 140.4, 140.1, 139.7, 139.6, 139.5, 139.4, 138.6, 129.2 (2C), 129.12 (two peaks overlapped, 4C), 129.08 (two peaks overlapped, 4C), 129.0 (2C), 128.92 (2C), 128.86 (2C), 128.8 (2C), 128.71,

128.68 (2C), 128.6 (four peaks overlapped, 6C), 128.5 (2C), 128.4, 128.3 (2C), 128.29 (2C), 128.26 (two peaks overlapped, 3C), 127.8, 127.70, 127.67, 101.9, 101.7, 101.5, 99.8, 99.5, 98.6, 98.5 (two peaks overlapped, 2C), 83.1, 79.5, 79.3, 79.21, 79.16, 78.94 (two peaks overlapped, 2C), 78.90 (two peaks overlapped, 2C), 78.6 (two peaks overlapped, 2C), 77.1, 77.0 (two peaks overlapped, 2C), 75.4, 75.3, 75.2, 75.1, 75.0, 74.7, 74.5, 74.4, 74.2, 73.7, 73.6, 71.9, 71.8, 71.7, 71.2, 70.9, 70.8, 70.6, 70.5, 70.2, 70.1 (three peaks overlapped, 3C), 69.8, 68.7, 63.5, 63.04, 62.96, 62.7, 55.4, 55.2, 55.0, 54.9, 52.9, 52.4, 52.19, 52.17, 28.7 (3C), 28.6 (three peaks overlapped, 9C), 21.00, 20.95, 20.90, 20.87, 20.8, 20.7; HRMS: m/z (ESI) calcd for $C_{140}H_{176}N_4O_{53}Na^+$, $[M + Na]^+$, 2784.1092, found 2784.1055. $^{1}J_{^{13}C1-H1}$ = 175.7 Hz, 168.4 Hz, 170.9 Hz, 168.7 Hz, 171.4 Hz, 171.4 Hz, 160.5 Hz.

The stereochemistry of newly formed anomeric center (C1) of **52** was determined by measuring $^{1}J_{^{13}C1-H1}$ (171.4 Hz) through un-decoupled HSQC experiments.[11-12] The C2 stereochemistry was determined by the stereochemistry of glycal-α-epoxide **S42**.

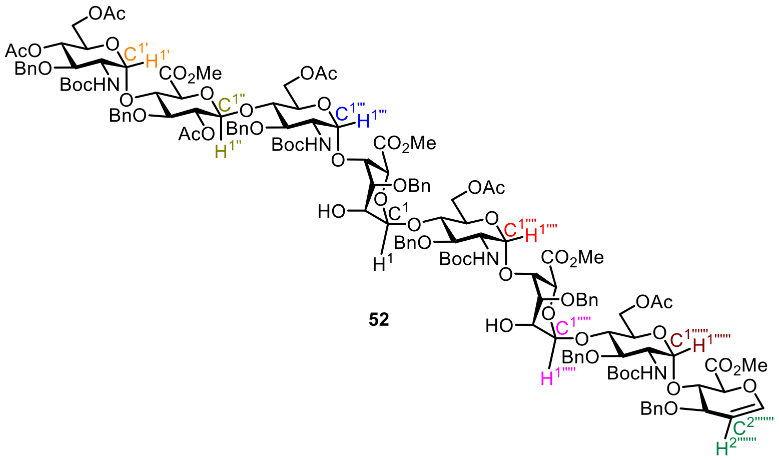

52

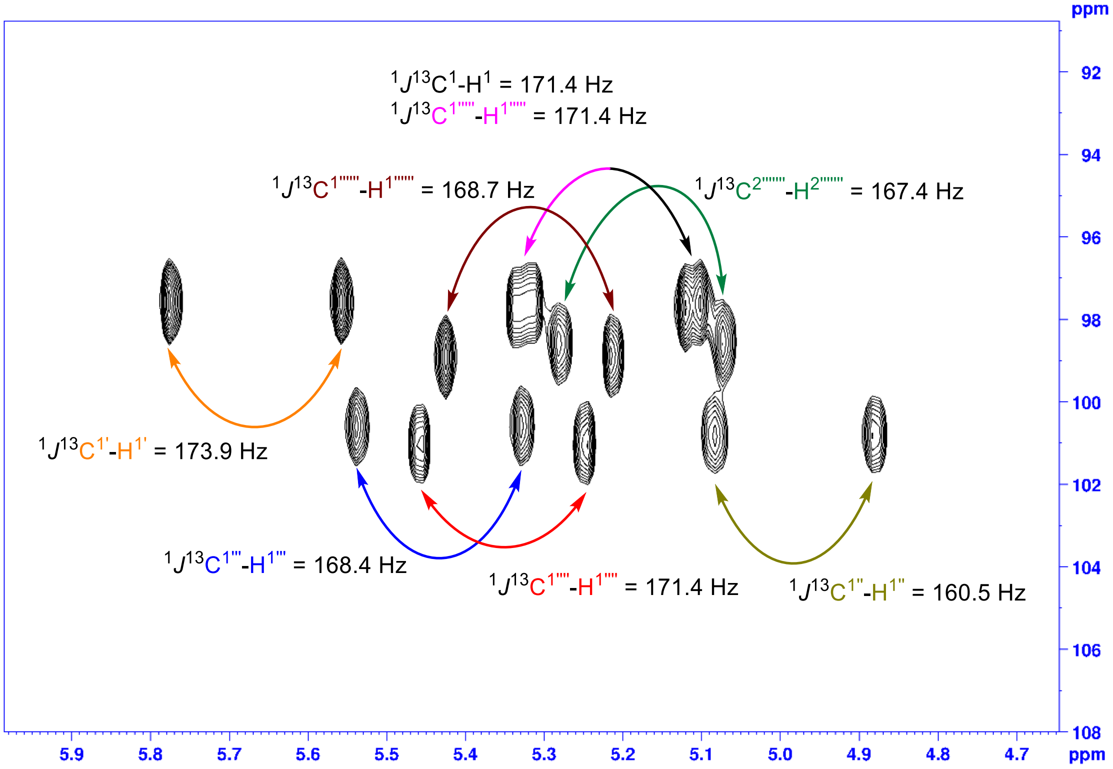

Figure S31. Un-decoupled HSQC Analysis to Determine Stereochemistry of **52**.

A $^{1}J^{13}{}_{C1\text{–}H1}$ value of 171.4 Hz suggested that the newly formed glycosidic bond is in axial position, which confirmed the generation of a 1,2-*trans*-α-glycosidic linkage.

J. Biological Assays of the Heparan Sulfate Hexasaccharide 42

Measuring the anti-Factor Xa Activity of 42

Assays were carried out based on a published method.[20-21] Briefly, human factor Xa (Enzyme Research Laboratories, South Bend, IN) was diluted to 50 U/ml with PBS. Antithrombin (AT from Cutter Biologics) was diluted to 0.03mg/ml with PBS including 1mg/ml BSA). The chromogenic substrate, S-2765 (from Diapharma (Westchester, OH) was made up at 1 mg/ml in water.

Fondaparinux (from Sanofi) and **42** were dissolved in PBS at various concentrations (3 to 600 µg/ml). The reaction mixture, which consisted of 70 µl of antithrombin (Sigma–Aldrich) and 40 µl of the solution containing the saccharides, was incubated at room temperature for 2 min. Factor Xa (100 µl) was then added. After incubating at room temperature for 4 min, 30 µl of S-2765 substrate was added. The absorbance of the reaction mixture was measured at 405 nm continuously for 5 min. The absorbance values were plotted against the reaction time. The initial reaction rates as a function of concentration were used to calculate the IC_{50} values.

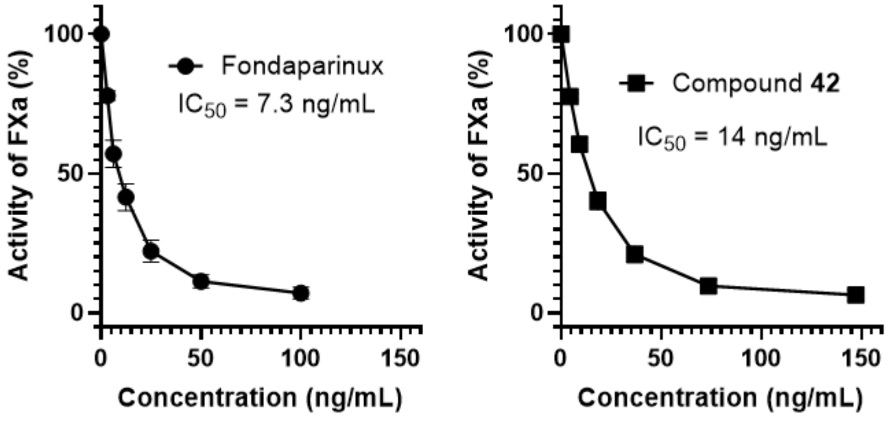

Figure S32. Comparison of the anti-Factor Xa (FXa) Activity between Fondapariux and **42**.

The inhibition of FXa activity was completed in an *in vitro* formation. The data are presented as the format of average ± S.D. (n =3).

The IC_{50} value for **42** was determined to be 14 ng/mL, which is comparable to that of fondaparinux (7.3 ng/mL), suggesting that hexasaccharide **42** displays the anticoagulant activity.

Determination of the Pharmacokinetics of 42 in Mice

Male C57BL/6 mice, 8 weeks of age, were purchased from Jackson Laboratory. Stocks of compound **42** were diluted in normal saline and administered at a dose of 1 mg/kg based on body weight. Following compound administration, anticoagulated blood was collected intravenously via the retro-orbital venous plexus. All animal procedures were approved by the Institutional Animal Care and Use Committee at the University of North Carolina at Chapel Hill. The plasma concentration of compound **42** was determined by a LC–MS/MS method using a ^{13}C-labeled dekaparin as a calibrant. Briefly, 0.4 μg of ^{13}C-dekaparin calibrant was added to the plasma. To the plasma (20 ml), 4 μL of heparin lyase I (0.5 mg/mL), 2.5 μL of heparin lyase II (12.9 mg/mL), and 7.5 μL of enzymatic buffer (100 mM sodium acetate, 2 mM calcium acetate, and 0.1g/L BSA) were added. The mixture was incubated at 37°C overnight to digest compound **42** and the calibrant. Subsequently, the digested products were recovered from the reaction mixture and dried. Then, the dried samples were subjected to AMAC (2-aminoacridone) derivatization by mixing the samples with 5 μL of 0.1M AMAC solution in DMSO and acetic acid (in a 17:3 v/v ratio), and the mixture was kept in the dark at room temperature for 15 min. Next, the samples were incubated with 5 μL of aqueous $NaBH_3CN$ at 45 °C for 2 h. The samples were collected and stored at room temperature for LC–MS/MS analysis. The analysis of AMAC-labeled HS di/trisaccharides was performed on LC–MS/MS, which consists of a Vanquish Flex ultra HPLC and a TSQ Fortis triple-quadrupole MS. The samples were separated on an ACQUITY Glycan BEH Amide column (1.7 μm, 150 mm × 2.1 mm; Waters) at 60 °C. Mobile phase A was 50 mM ammonium formate in water (*p*H 4.4), and mobile phase B was acetonitrile. The elution gradient was: 0–15 min 83–70% B; 15–30 min, 70–50% B; 30.1–35 min, 50% B; 35.1–40 min, 83% B. The flow rate is 0.3 mL/min. The MS system operates in MRM mode. The electrospray ionization was operated in the negative mode using the following parameters: spray voltage at -3.0 kV, sheath gas at 55 Arb, aux gas at 25 Arb, ion transfer tube temperature at 250 °C, and vaporizer temperature at 400 °C. TraceFinder software was applied for data acquisition and analysis.

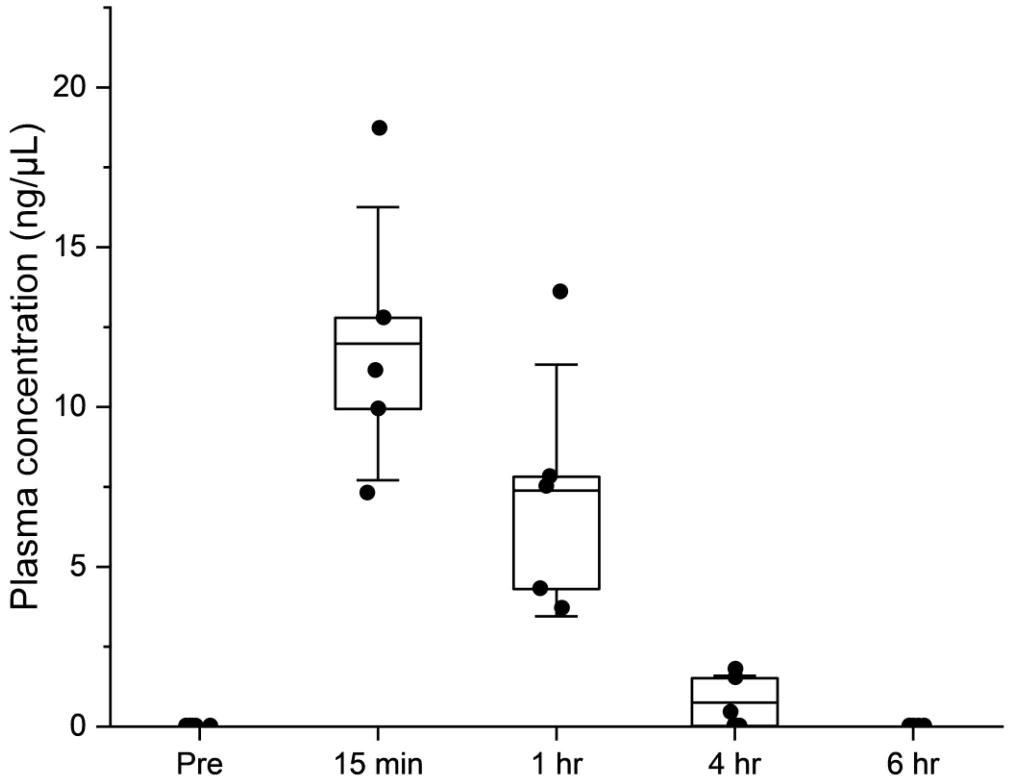

Figure S33. Determination of the Clearance Rate of **42**.

Compound **42** was administered subcutaneously to mice (n=5). The blood samples (20 ml) were collected via the retro-orbital venous plexus at 15-min, 1-hour, 4-hour and 6-hour time points. The plasma concentrations at different time points were determined by the LC–MS/MS method as described above. The plasma concentration of **42** reached the maximum between 15 min and one hour and it was completely cleared after four hours.

K. References

1. Schell, P.; Orgueira, H. A.; Roehrig, S.; Seeberger, P. H., Synthesis and Transformations of D-Glucuronic and L-Iduronic Acid Glycals. *Tetrahedron Lett.* **2001**, *42*, 3811-3814.

2. Li, H.; Zhang, D.; Li, C.; Yin, L.; Jiang, Z.; Luo, Y.; Xu, H., Stereoselective Glycosylation for 1,2-*cis*-Aminoglycoside Assembly by Cooperative Atom Transfer Catalysis. *J. Am. Chem. Soc.* **2024**, *146*, 33316-33323.

3. Ingle, A. B.; Chao, C.-S.; Hung, W.-C.; Mong, K.-K. T., Tuning Reactivity of Glycosyl Imidinium Intermediate for 2-Azido-2-deoxyglycosyl Donors in α-Glycosidic Bond Formation. *Org. Lett.* **2013**, *15*, 5290-5293.

4. House, S. E.; Poon, K. W. C.; Lam, H.; Dudley, G. B., p-Siletanylbenzylidene Acetal: Oxidizable Protecting Group for Diols. *J. Org. Chem.* **2006**, *71*, 420-422.

5. Zhang, X.-W.; Yin, L.; Zhang, D.; Jiang, Z.; Wang, P.; Xu, H., Iron-Catalyzed Highly Stereospecific Glycosylation with Glycal Epoxides. *Angew. Chem. Int. Ed.* **2025**, *64*, e202517634.

6. Nicolaou, K. C.; Mitchell, H. J.; Jain, N. F.; Winssinger, N.; Hughes, R.; Bando, T., Total Synthesis of Vancomycin. *Angew. Chem. Int. Ed.* **1999**, *38*, 240-244.

7. Bernotas, R. C., A Short, Versatile Approach to Polyhydroxylated Pyrrolidines Utilizing a Reductive Elimination-Reductive Amination as a Key Step. *Tetrahedron Lett.* **1990**, *31*, 469-472.

8. Pal, K. B.; Lee, J.; Das, M.; Liu, X.-W., Palladium(II)-Catalyzed Stereoselective Synthesis of *C*-Glycosides from Glycals with Diaryliodonium Salts. *Org. Biomol. Chem.* **2020**, *18*, 2242-2251.

9. Chandrasekhar, S.; Raji Reddy, C.; Jagadeeshwar Rao, R., Facile and Selective Cleavage of Allyl Ethers, Amines and Esters using Polymethylhydrosiloxane–$ZnCl_2$/$Pd(PPh_3)_4$. *Tetrahedron* **2001**, *57*, 3435-3438.

10. Eby, R.; Srivastava, V. K., Conformational Analysis of 1,2-Anhydro-3,4,6-tri-*O*-Benzyl-α-D-Glucopyranose and -β-D-Mannopyranose. *Carbohydr. Res.* **1982**, *102*, 1-9.

11. Bock, K.; Pedersen, C., A Study of ^{13}CH Coupling Constants in Hexopyranoses. *J. Chem. Soc., Perkin Trans. 2* **1974**, 293-297.

12. Bock, K.; Lundt, I.; Pedersen, C., Assignment of Anomeric Structure to Carbohydrates through Geminal ^{13}C-H Coupling Constants. *Tetrahedron Lett.* **1973**, *14*, 1037-1040.

13. Hansen, S. U.; Miller, G. J.; Baráth, M.; Broberg, K. R.; Avizienyte, E.; Helliwell, M.; Raftery, J.; Jayson, G. C.; Gardiner, J. M., Synthesis and Scalable Conversion of l-Iduronamides to Heparin-Related Di- and Tetrasaccharides. *J. Org. Chem.* **2012**, *77*, 7823-7843.

14. Hansen, S. U.; Dalton, C. E.; Baráth, M.; Kwan, G.; Raftery, J.; Jayson, G. C.; Miller, G. J.; Gardiner, J. M., Synthesis of l-Iduronic Acid Derivatives via [3.2.1] and [2.2.2] l-Iduronic Lactones from

Bulk Glucose-Derived Cyanohydrin Hydrolysis: A Reversible Conformationally Switched Superdisarmed/Rearmed Lactone Route to Heparin Disaccharides. *J. Org. Chem.* **2015**, *80*, 3777-3789.

15. Whitfield, D. M.; I., B. G.; Henrianna, P.; Jose, B.; and Sarkar, B., Synthesis of L-Iduronic Acid Derivatives: Crystal Structure of Methyl (Methyl 2,3,4-Tri-O-Acetyl-β-L-Idopyranosid)Uronate. *J. Carbohydr. Chem.* **1991**, *10*, 329-348.

16. Taber, D. F.; DeMatteo, P. W.; Hassan, R. A., Simplified Preparation of Dimethyldioxirane (DMDO). In *Organic Syntheses*, pp 350-357.

17. Xu, Y.; Chandarajoti, K.; Zhang, X.; Pagadala, V.; Dou, W.; Hoppensteadt, D. M.; Sparkenbaugh, E. M.; Cooley, B.; Daily, S.; Key, N. S.; Severynse-Stevens, D.; Fareed, J.; Linhardt, R. J.; Pawlinski, R.; Liu, J., Synthetic Oligosaccharides Can Replace Animal-Sourced Low–Molecular Weight Heparins. *Sci. Transl. Med.* **2017**, *9*, eaan5954.

18. Xu, Y.; Masuko, S.; Takieddin, M.; Xu, H.; Liu, R.; Jing, J.; Mousa, S. A.; Linhardt, R. J.; Liu, J., Chemoenzymatic Synthesis of Homogeneous Ultralow Molecular Weight Heparins. *Science* **2011**, *334*, 498–501.

19. Xu, Y.; Cai, C.; Chandarajoti, K.; Hsieh, P.-H.; Li, L.; Pham, T. Q.; Sparkenbaugh, E. M.; Sheng, J.; Key, N. S.; Pawlinski, R.; Harris, E. N.; Linhardt, R. J.; Liu, J., Homogeneous Low-Molecular-Weight Heparins with Reversible Anticoagulant Activity. *Nat. Chem. Biol.* **2014**, *10*, 248-250.

20. Zhang, L.; Beeler, D. L.; Lawrence, R.; Lech, M.; Liu, J.; Davis, J. C.; Shriver, Z.; Sasisekharan, R.; Rosenberg, R. D., 6-O-Sulfotransferase-1 Represents a Critical Enzyme in the Anticoagulant Heparan Sulfate Biosynthetic Pathway. *J. Biol. Chem.* **2001**, *276*, 42311-42321.

21. Duncan, M. B.; Chen, J.; Krise, J. P.; Liu, J., The Biosynthesis of Anticoagulant Heparan Sulfate by the Heparan Sulfate 3-*O*-Sulfotransferase Isoform 5. *Biochim. Biophys. Acta* **2004**, *1671*, 34-43.

L. NMR Spectra

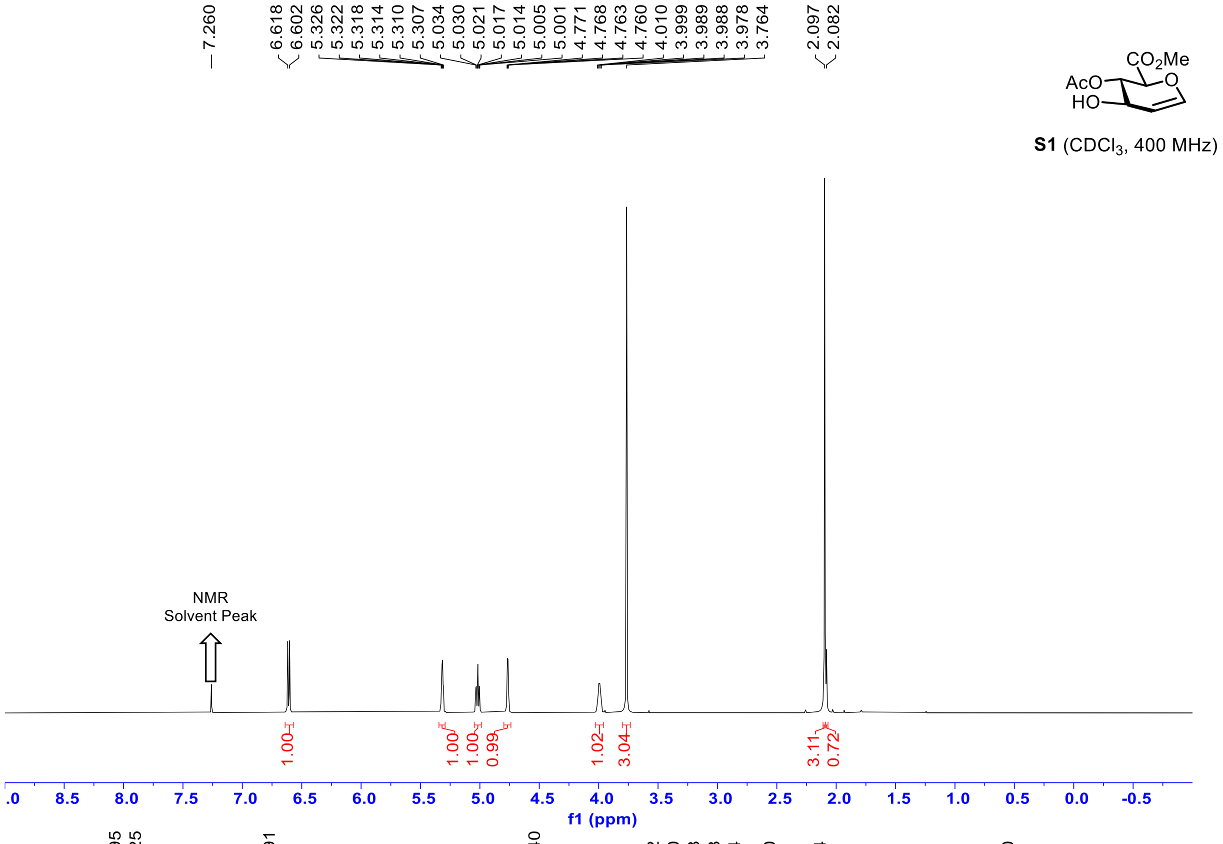

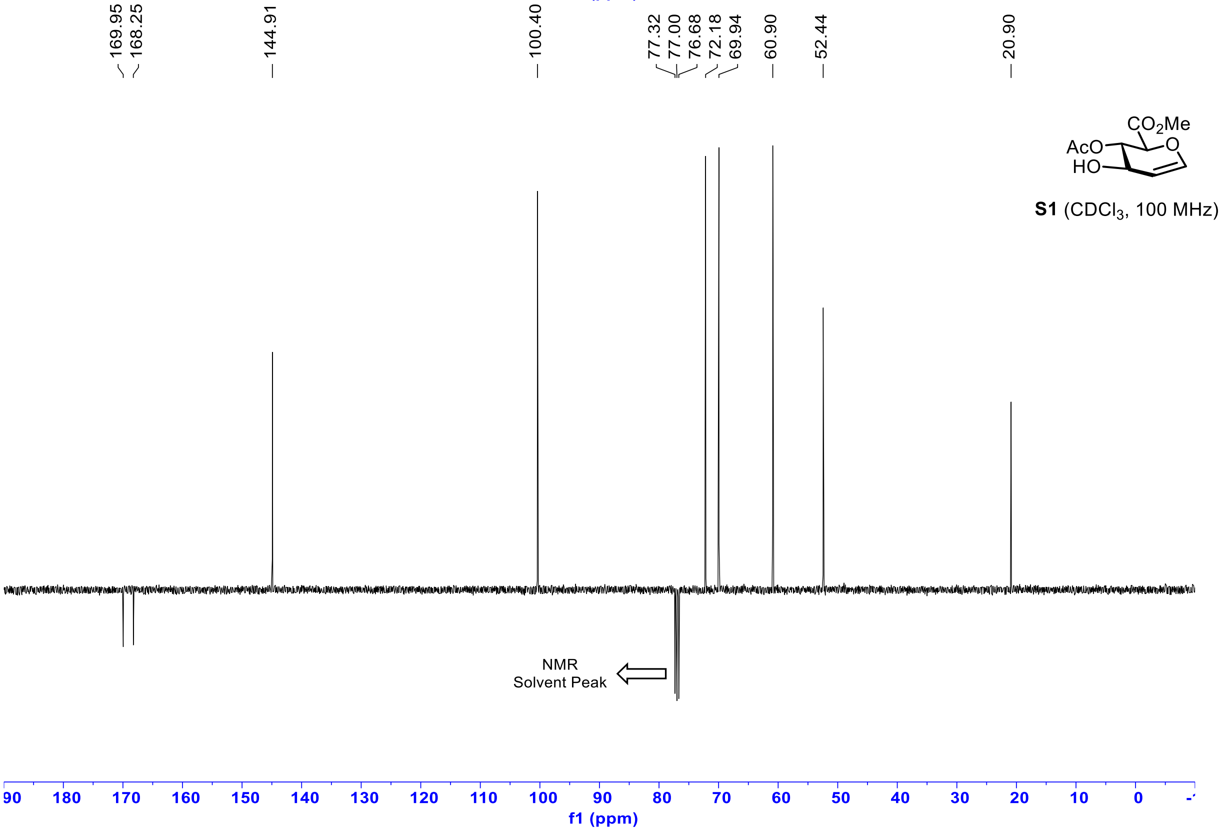

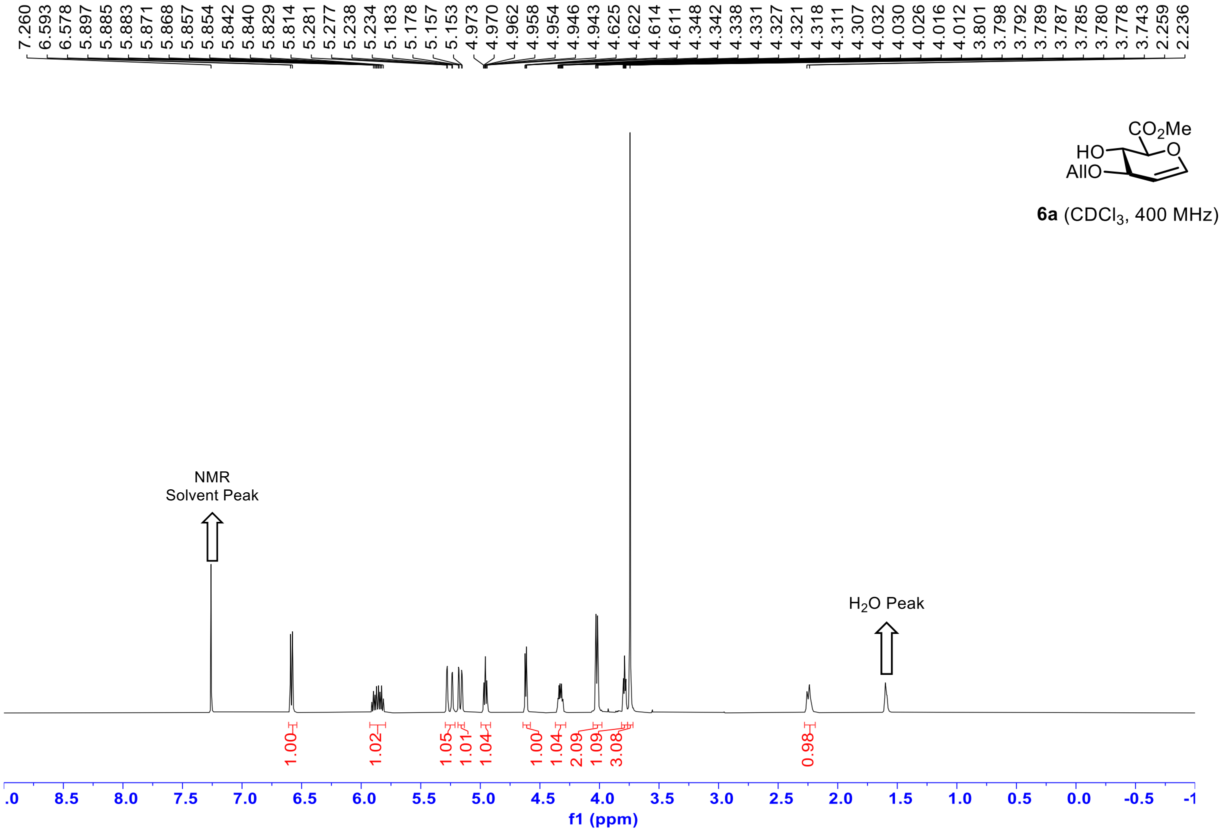
7.260 6.593 6.578 5.897 5.885 5.883 5.871 5.868 5.857 5.854 5.842 5.840 5.829 5.814 5.281 5.277 5.238 5.234 5.183 5.178 5.157 5.153 4.973 4.970 4.962 4.958 4.954 4.946 4.943 4.625 4.622 4.614 4.611 4.348 4.342 4.338 4.331 4.327 4.321 4.318 4.311 4.307 4.032 4.030 4.026 4.016 4.012 3.801 3.798 3.792 3.789 3.787 3.785 3.780 3.778 3.743 2.259 2.236
CO2Me
HO
O
AllO
6a (CDCl3, 400 MHz)
NMR
Solvent Peak
H2O Peak
1.00 1.02 1.05 1.01 1.04 1.00 1.04 2.09 1.09 3.08 0.98
f1 (ppm)

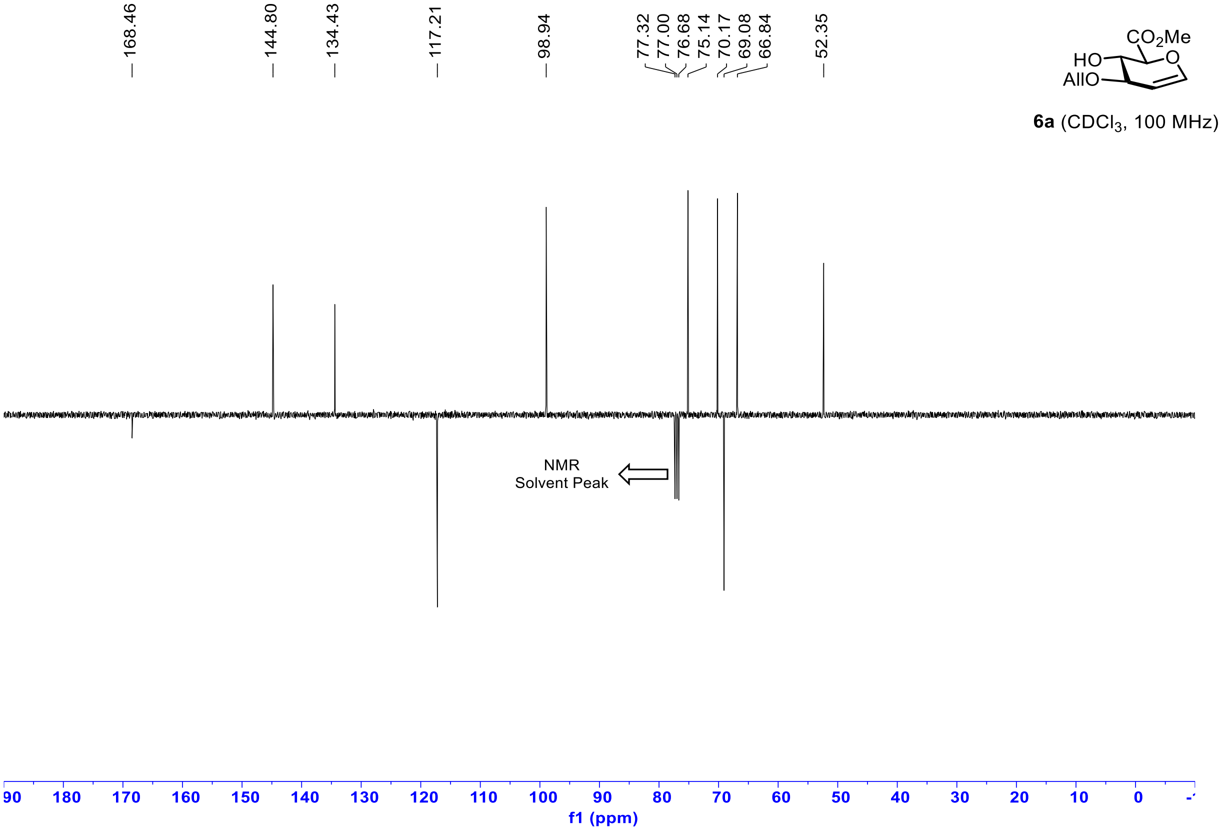
168.46 144.80 134.43 117.21 98.94 77.32 77.00 76.68 75.14 70.17 69.08 66.84 52.35
CO2Me
HO
O
AllO
6a (CDCl3, 100 MHz)
NMR
Solvent Peak
f1 (ppm)

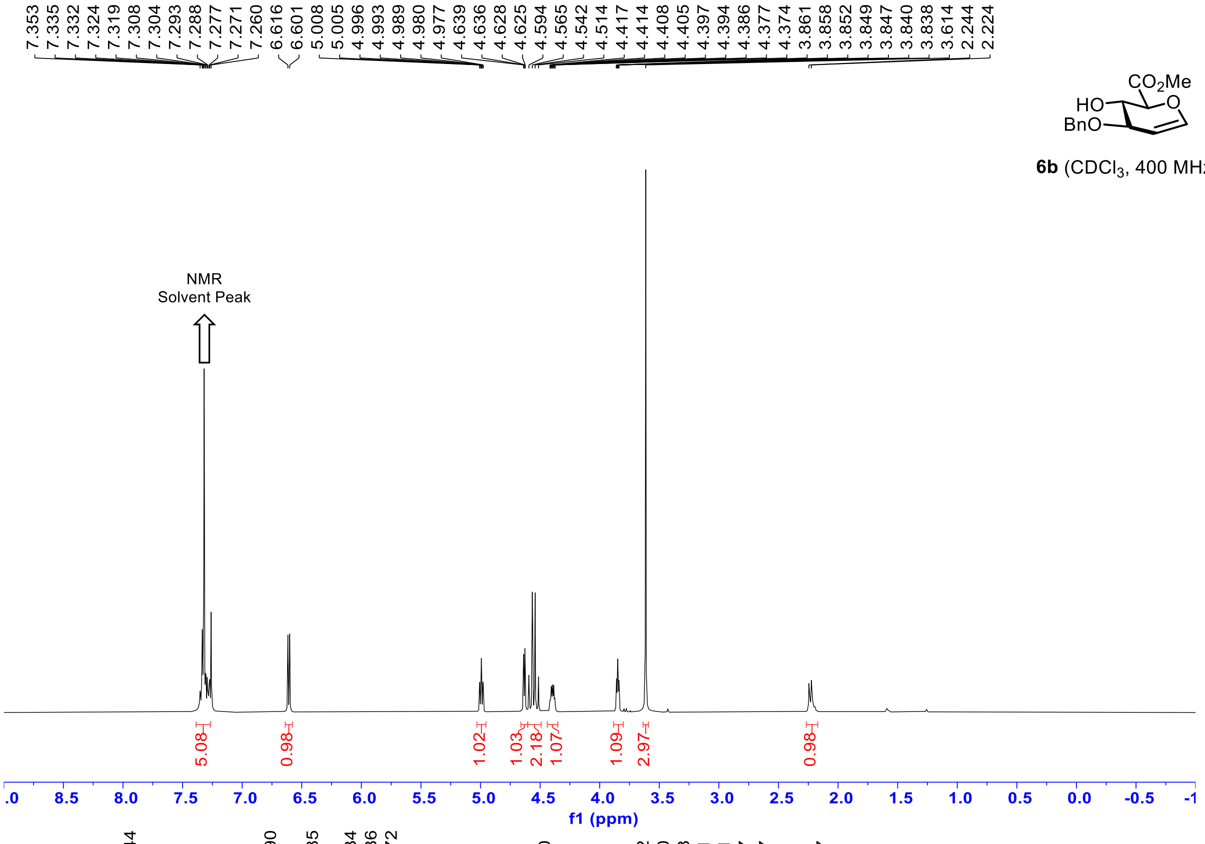

6b ($CDCl_3$, 400 MHz)

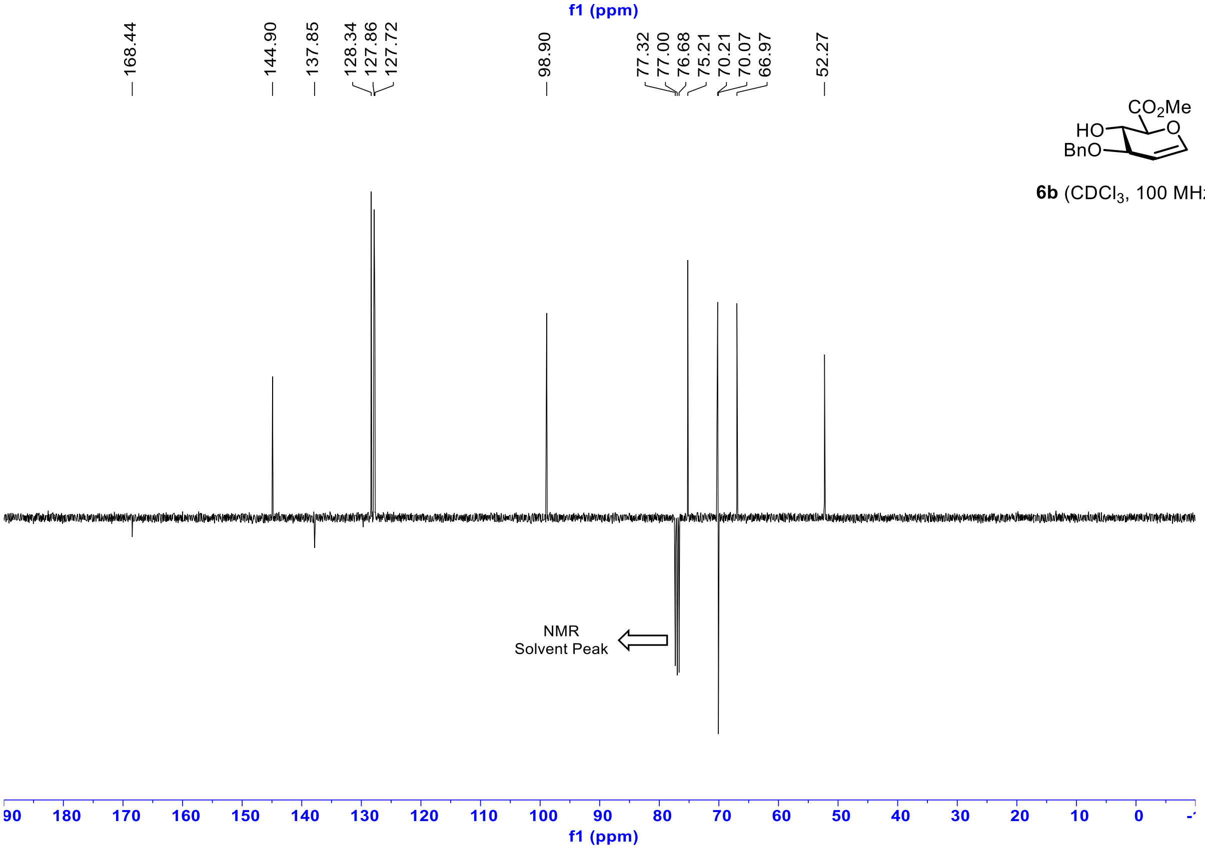

6b ($CDCl_3$, 100 MHz)

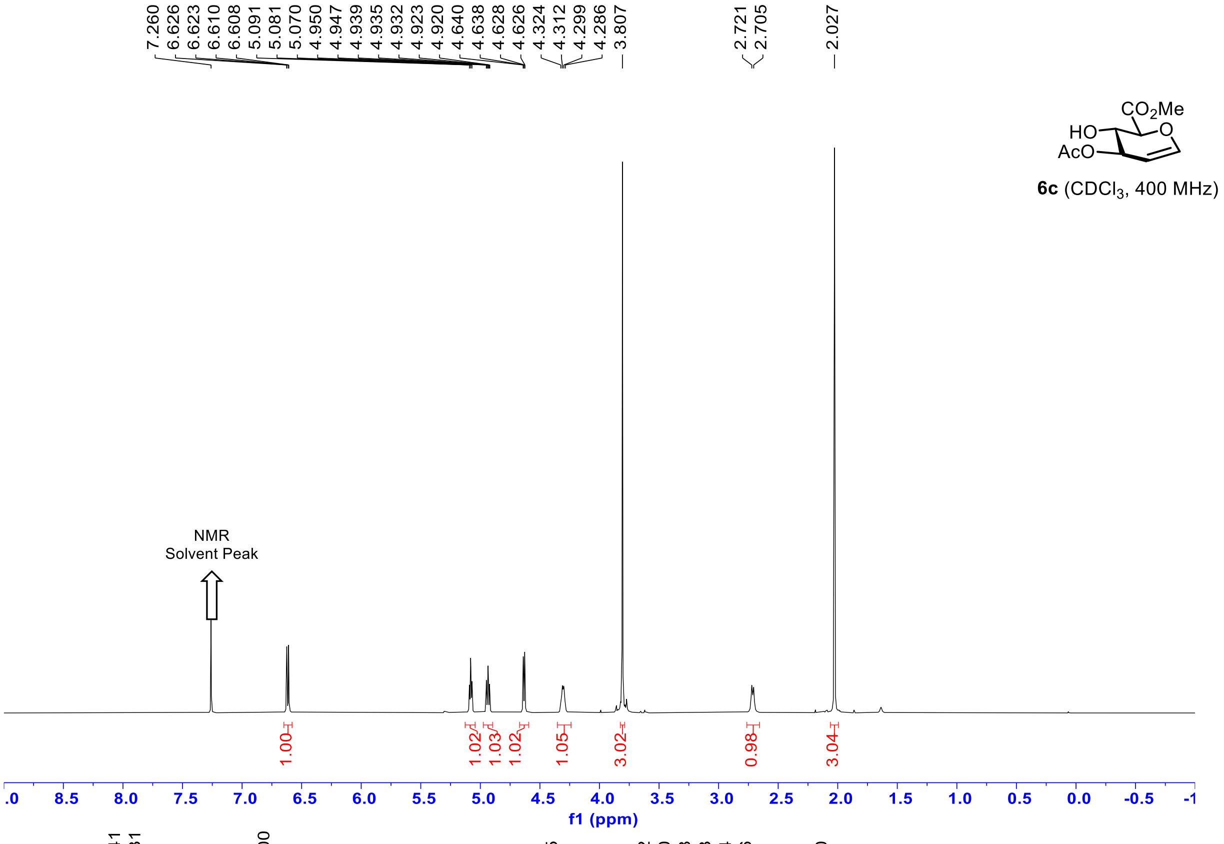
7.260
6.626
6.623
6.610
6.608
5.091
5.081
5.070
4.950
4.947
4.939
4.935
4.932
4.923
4.920
4.640
4.638
4.628
4.626
4.324
4.312
4.299
4.286
3.807
2.721
2.705
2.027
CO2Me
HO
O
AcO
6c (CDCl3, 400 MHz)
NMR
Solvent Peak
1.00
1.02
1.03
1.02
1.05
3.02
0.98
3.04
.0 8.5 8.0 7.5 7.0 6.5 6.0 5.5 5.0 4.5 4.0 3.5 3.0 2.5 2.0 1.5 1.0 0.5 0.0 -0.5 -1
f1 (ppm)

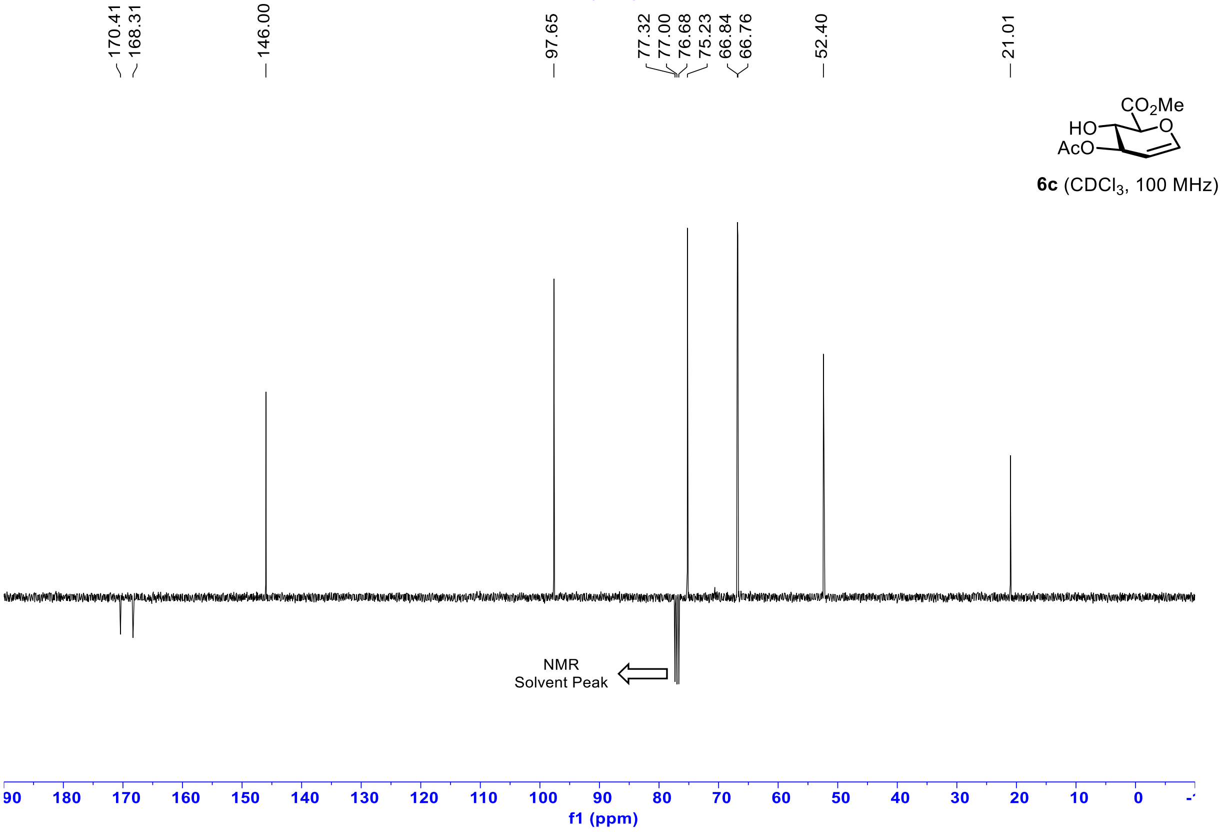
170.41
168.31
146.00
97.65
77.32
77.00
76.68
75.23
66.84
66.76
52.40
21.01
CO2Me
HO
O
AcO
6c (CDCl3, 100 MHz)
NMR
Solvent Peak
90 180 170 160 150 140 130 120 110 100 90 80 70 60 50 40 30 20 10 0
f1 (ppm)

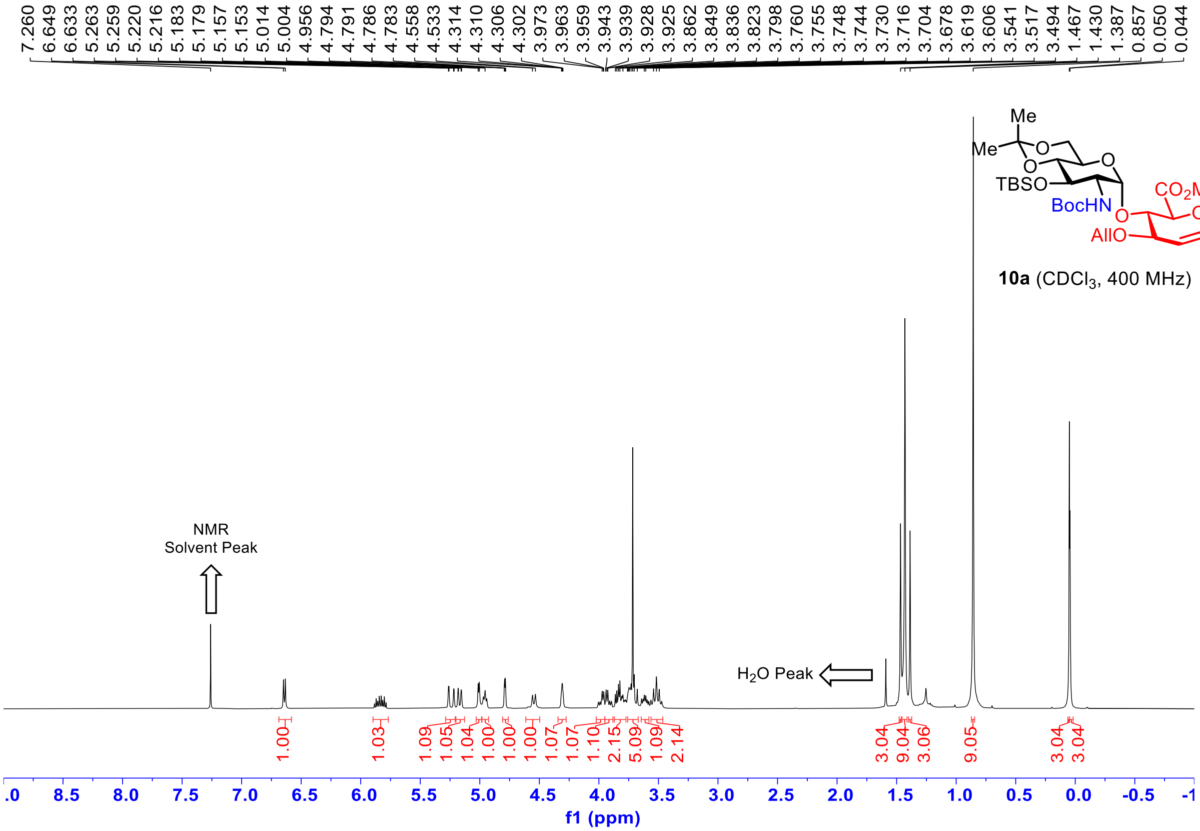
7.260 6.649 6.633 5.263 5.259 5.220 5.216 5.183 5.179 5.157 5.153 5.014 5.004 4.956 4.794 4.791 4.786 4.783 4.558 4.533 4.314 4.310 4.306 4.302 3.973 3.963 3.959 3.943 3.939 3.928 3.925 3.862 3.849 3.836 3.823 3.798 3.760 3.755 3.748 3.744 3.730 3.716 3.704 3.678 3.619 3.606 3.541 3.517 3.494 1.467 1.430 1.387 0.857 0.050 0.044
Me
Me
O
O
O
TBSO
BocHN
CO_2Me
AllO
10a ($CDCl_3$, 400 MHz)
NMR Solvent Peak
H_2O Peak
1.00 1.03 1.09 1.05 1.04 1.00 1.00 1.00 1.07 1.07 1.10 2.15 5.09 1.09 2.14 3.04 9.04 3.06 9.05 3.04 3.04
f1 (ppm)

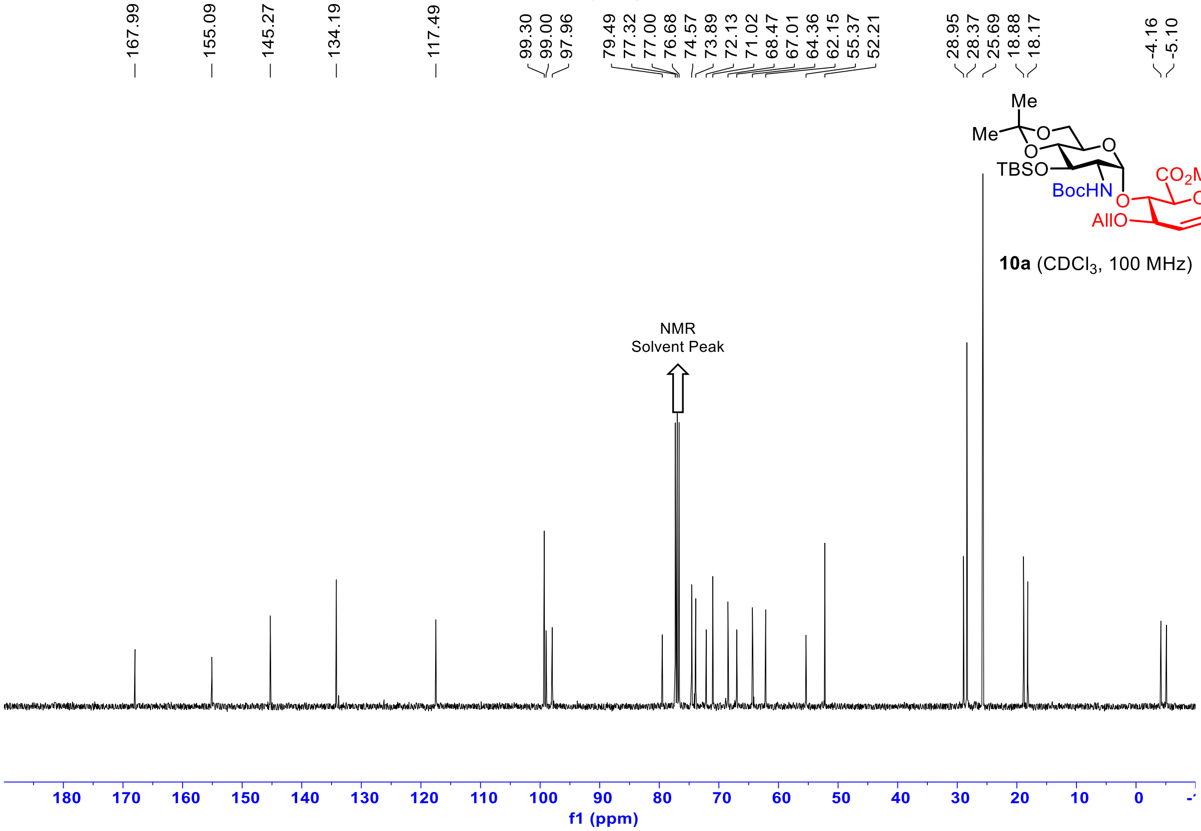
167.99 155.09 145.27 134.19 117.49 99.30 99.00 97.96 79.49 77.32 77.00 76.68 74.57 73.89 72.13 71.02 68.47 67.01 64.36 62.15 55.37 52.21 28.95 28.37 25.69 18.88 18.17 -4.16 -5.10
Me
Me
O
O
O
TBSO
BocHN
CO_2Me
AllO
10a ($CDCl_3$, 100 MHz)
NMR Solvent Peak
f1 (ppm)

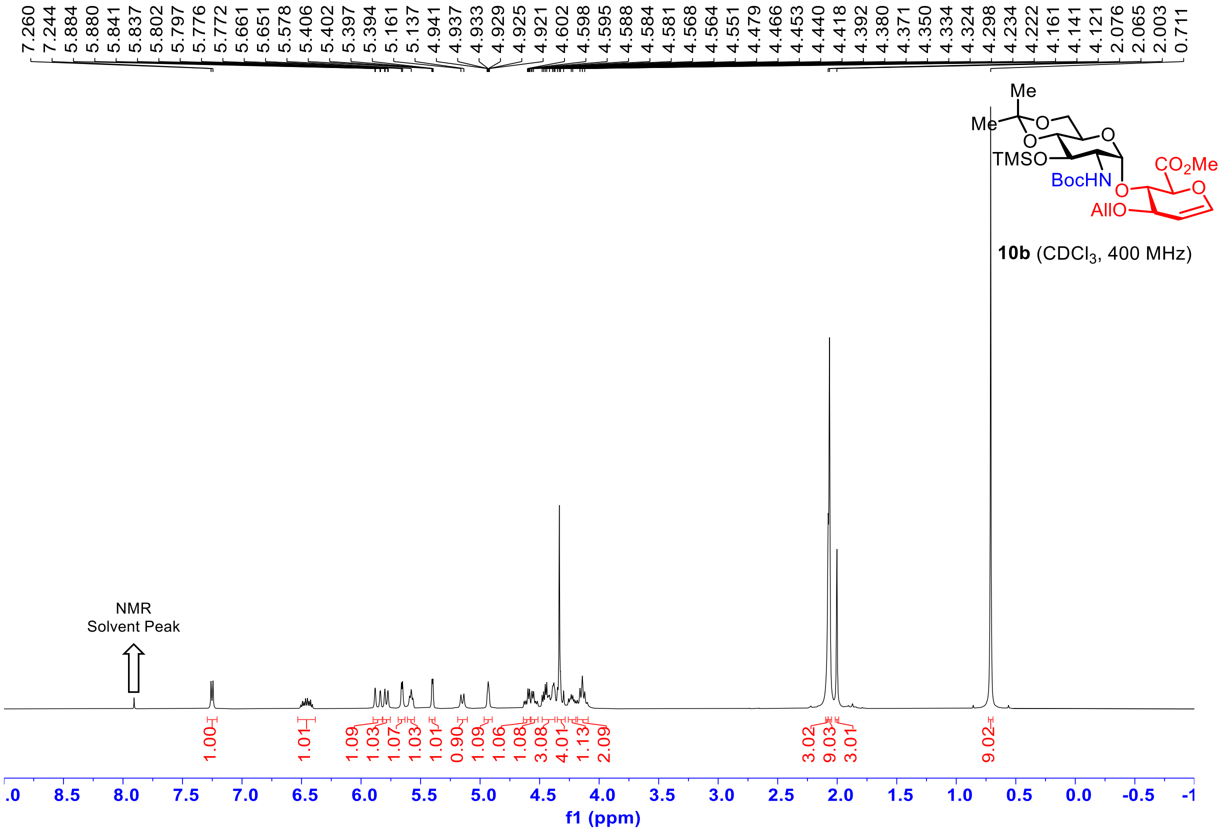
7.260 7.244 5.884 5.880 5.841 5.837 5.802 5.797 5.776 5.772 5.661 5.651 5.578 5.406 5.402 5.397 5.394 5.161 5.137 4.941 4.937 4.933 4.929 4.925 4.921 4.602 4.598 4.595 4.588 4.584 4.581 4.568 4.564 4.551 4.479 4.466 4.453 4.440 4.418 4.392 4.380 4.371 4.350 4.334 4.324 4.298 4.234 4.222 4.161 4.141 4.121 2.076 2.065 2.003 0.711
Me
Me
O
O
O
TMSO
BocHN
O
CO_2Me
O
AllO
10b ($CDCl_3$, 400 MHz)
NMR
Solvent Peak
1.00
1.01
1.09
1.03
1.07
1.03
1.01
0.90
1.09
1.06
1.08
3.08
4.01
1.13
2.09
3.02
9.03
3.01
9.02
f1 (ppm)

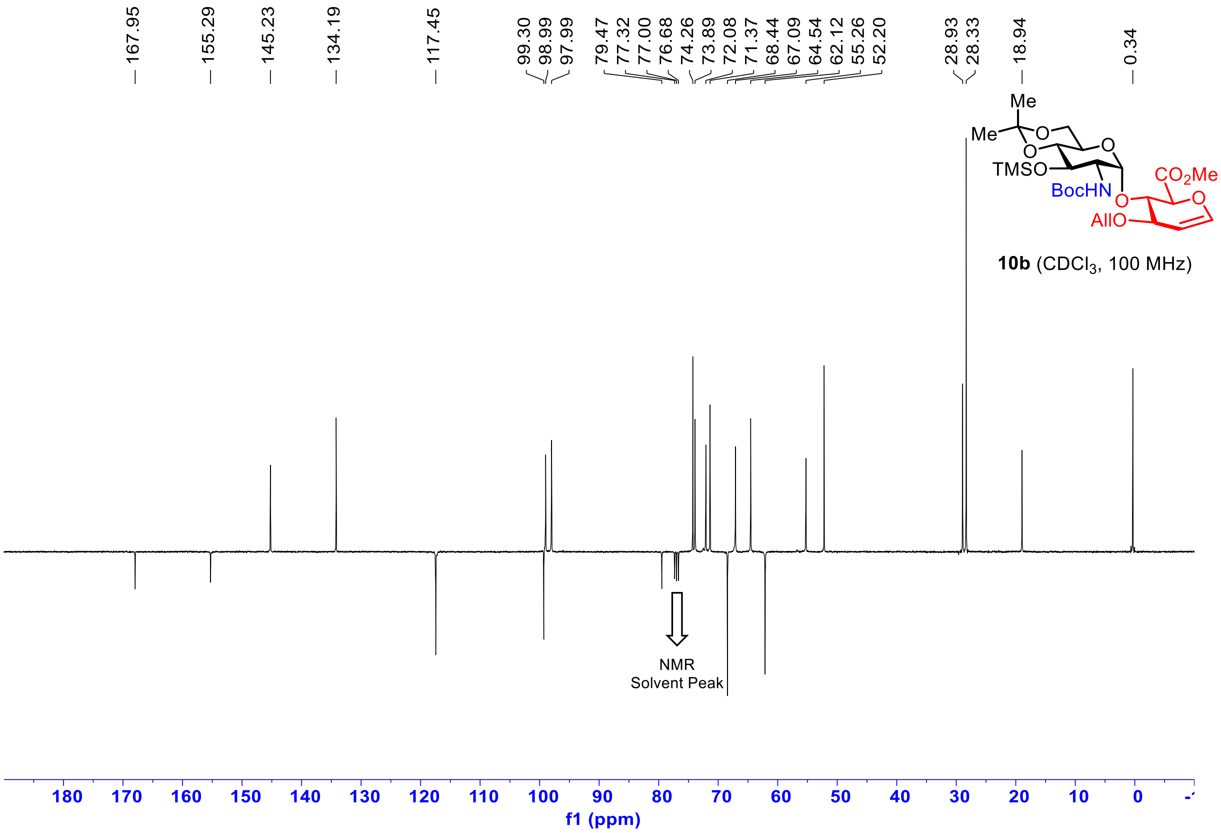
167.95 155.29 145.23 134.19 117.45 99.30 98.99 97.99 79.47 77.32 77.00 76.68 74.26 73.89 72.08 71.37 68.44 67.09 64.54 62.12 55.26 52.20 28.93 28.33 18.94 0.34
Me
Me
O
O
O
TMSO
BocHN
O
CO_2Me
O
AllO
10b ($CDCl_3$, 100 MHz)
NMR
Solvent Peak
180 170 160 150 140 130 120 110 100 90 80 70 60 50 40 30 20 10 0
f1 (ppm)

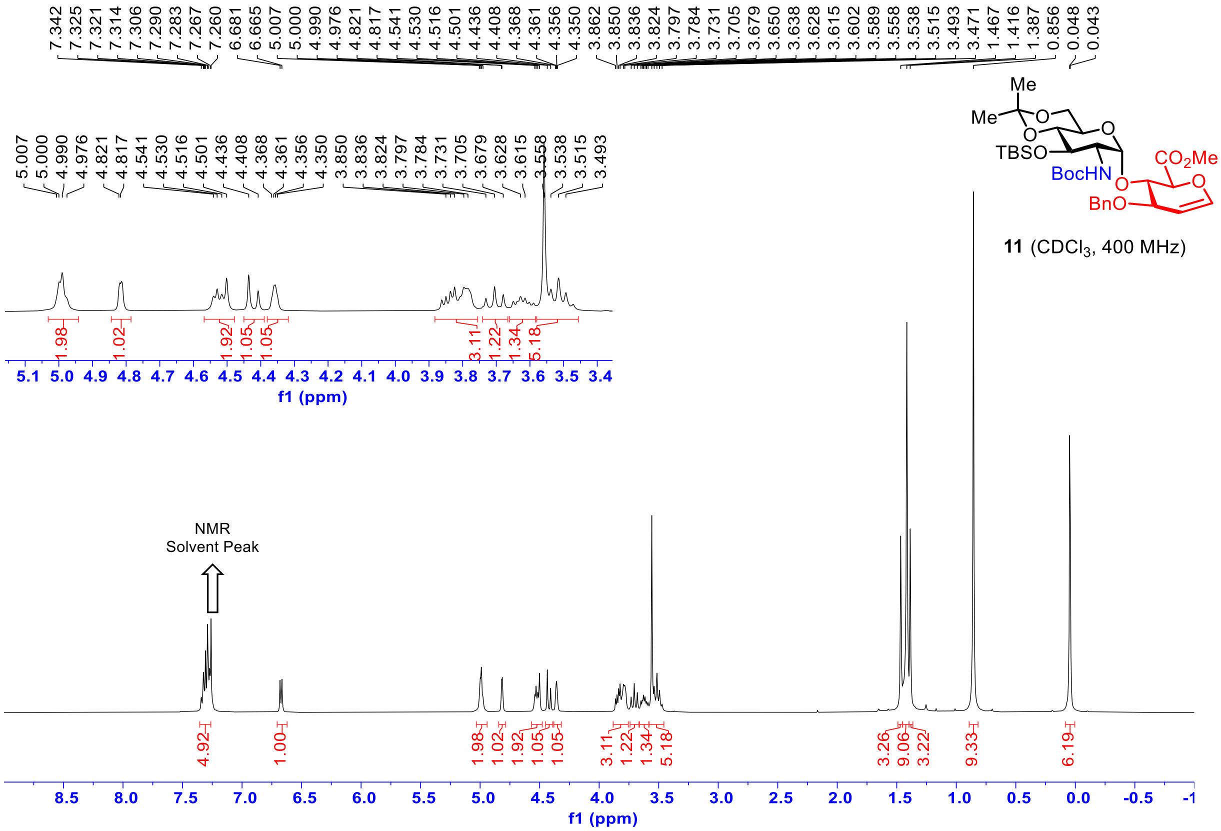
7.342 7.325 7.321 7.314 7.306 7.290 7.283 7.267 7.260 6.681 6.665 5.007 5.000 4.990 4.976 4.821 4.817 4.541 4.530 4.516 4.501 4.436 4.408 4.368 4.361 4.356 4.350 3.862 3.850 3.836 3.824 3.797 3.784 3.731 3.705 3.679 3.650 3.638 3.628 3.615 3.602 3.589 3.558 3.538 3.515 3.493 3.471 1.467 1.416 1.387 0.856 0.048 0.043
Me
Me
TBSO
BocHN
CO2Me
BnO
11 (CDCl3, 400 MHz)
NMR
Solvent Peak
f1 (ppm)

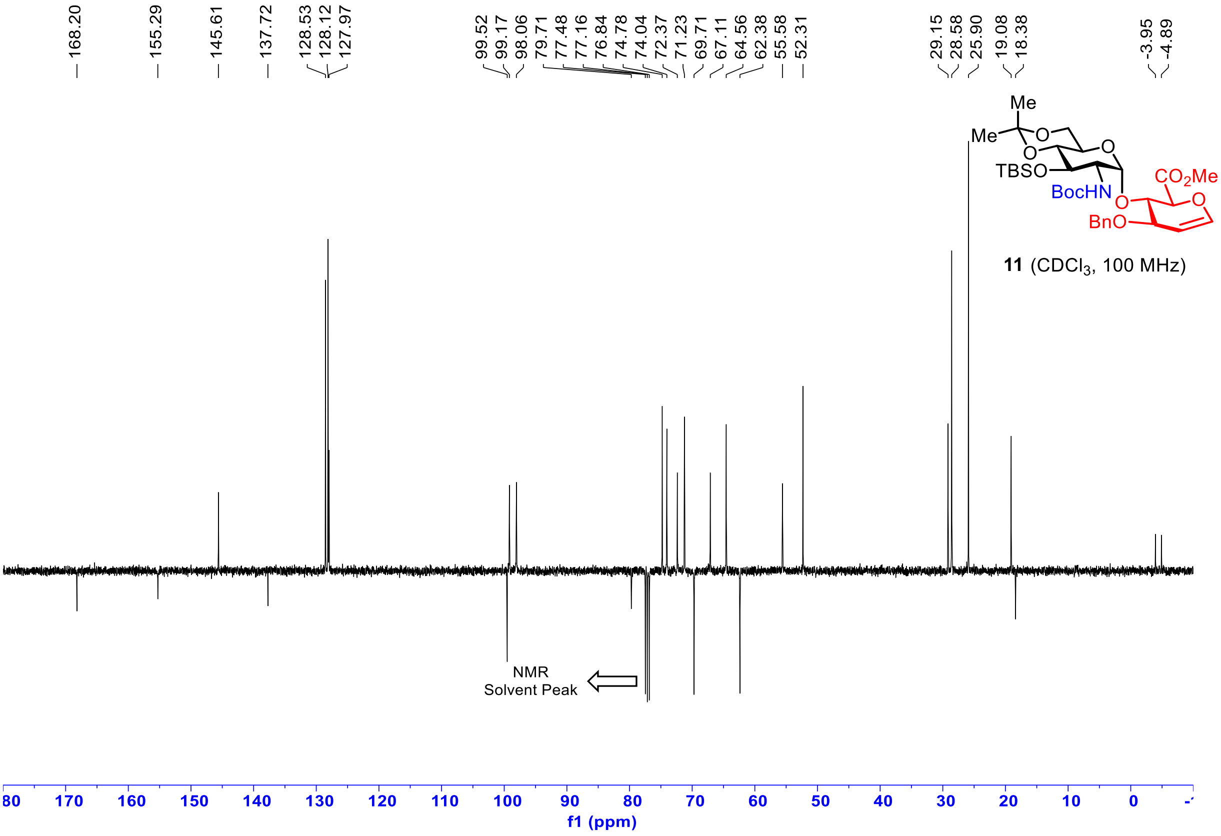
168.20 155.29 145.61 137.72 128.53 128.12 127.97 99.52 99.17 98.06 79.71 77.48 77.16 76.84 74.78 74.04 72.37 71.23 69.71 67.11 64.56 62.38 55.58 52.31 29.15 28.58 25.90 19.08 18.38 -3.95 -4.89
Me
Me
TBSO
BocHN
CO2Me
BnO
11 (CDCl3, 100 MHz)
NMR
Solvent Peak
f1 (ppm)

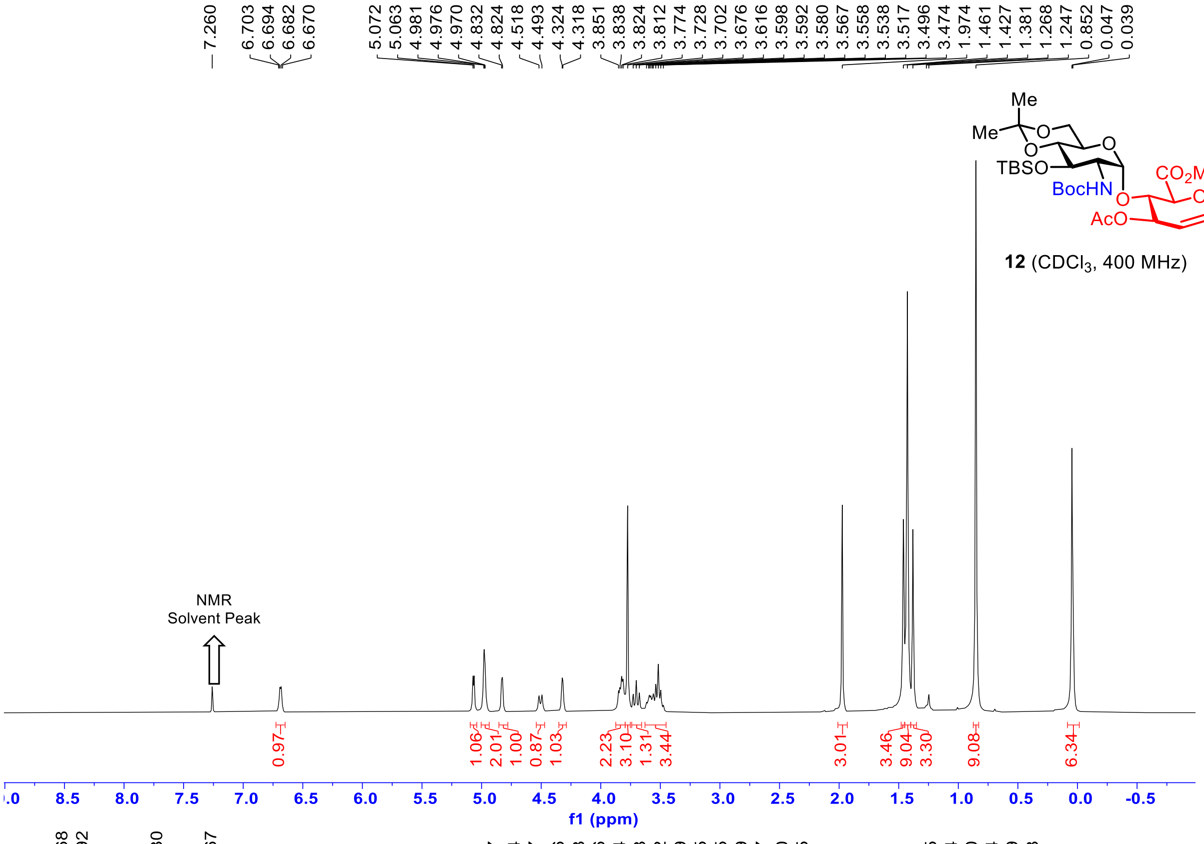
NMR
Solvent Peak
12 (CDCl3, 400 MHz)
f1 (ppm)

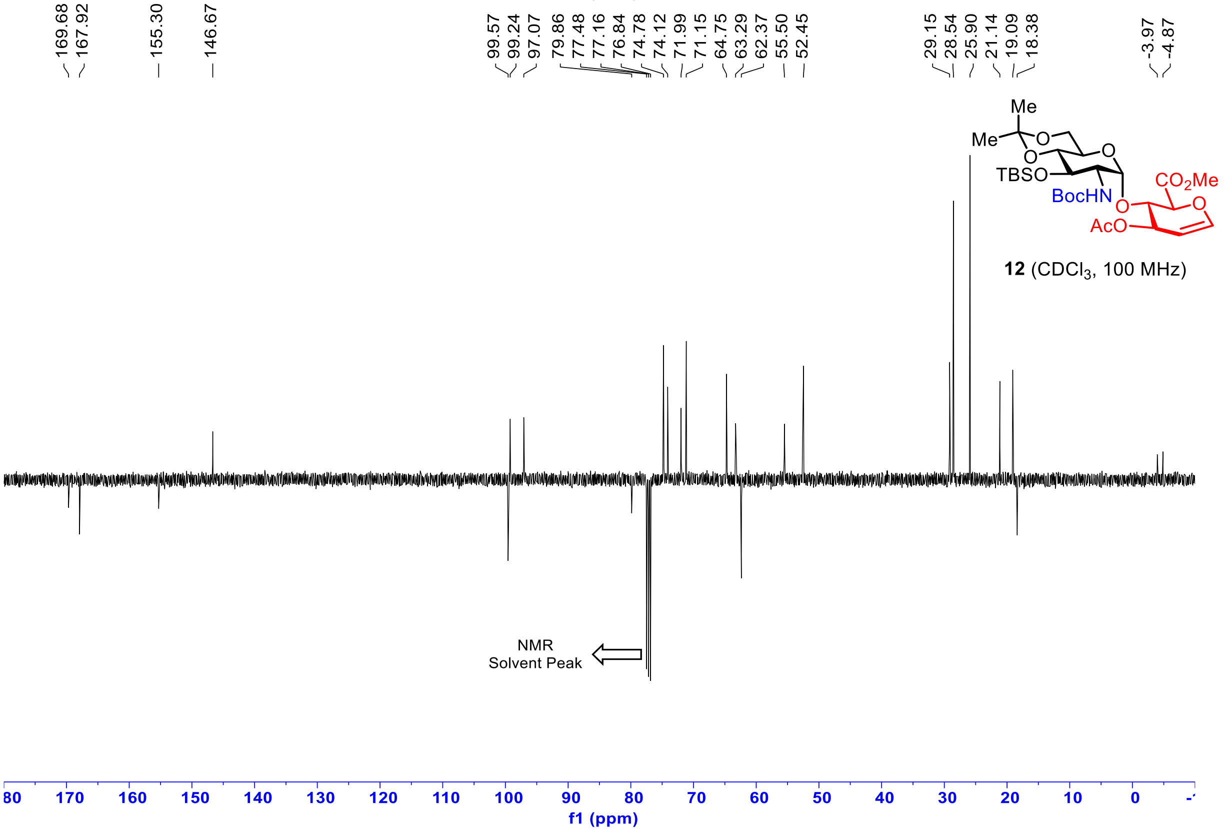
NMR
Solvent Peak
12 (CDCl3, 100 MHz)
f1 (ppm)

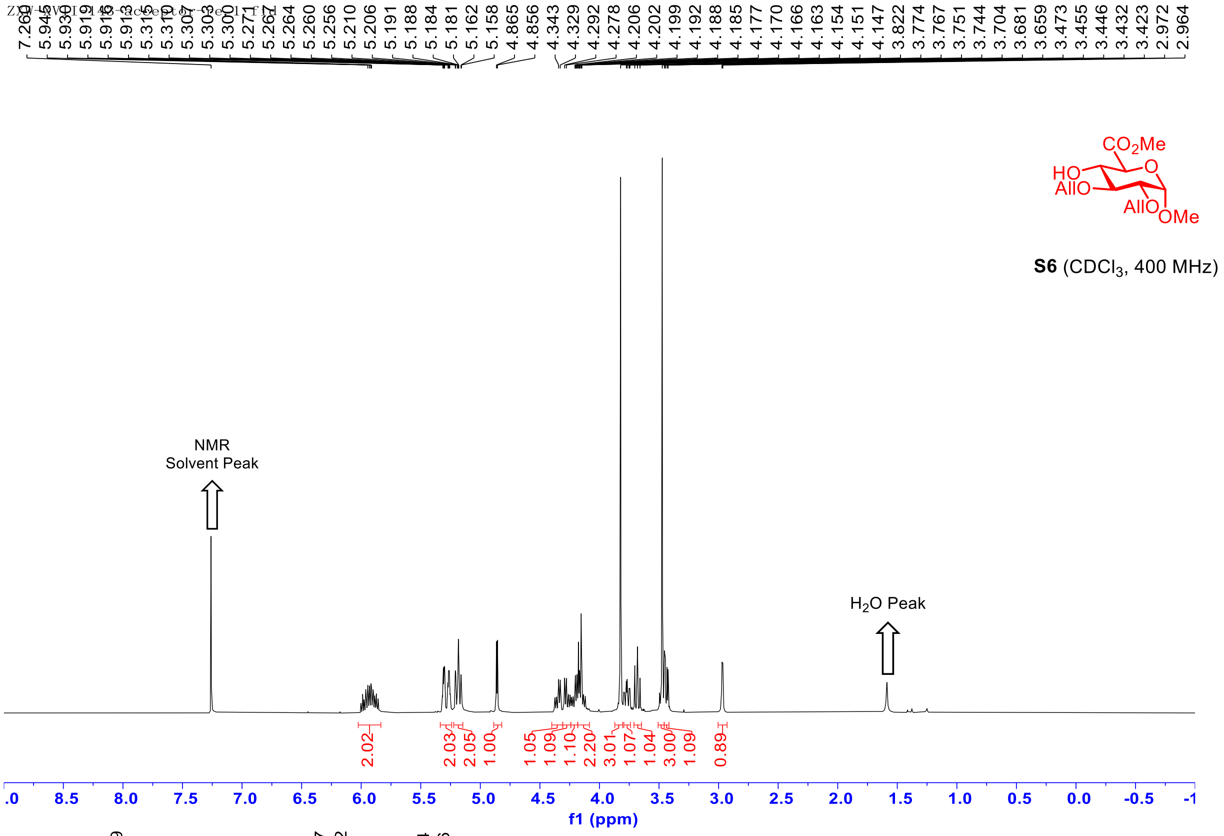
S6 ($CDCl_3$, 400 MHz)
NMR Solvent Peak
H_2O Peak
f1 (ppm)

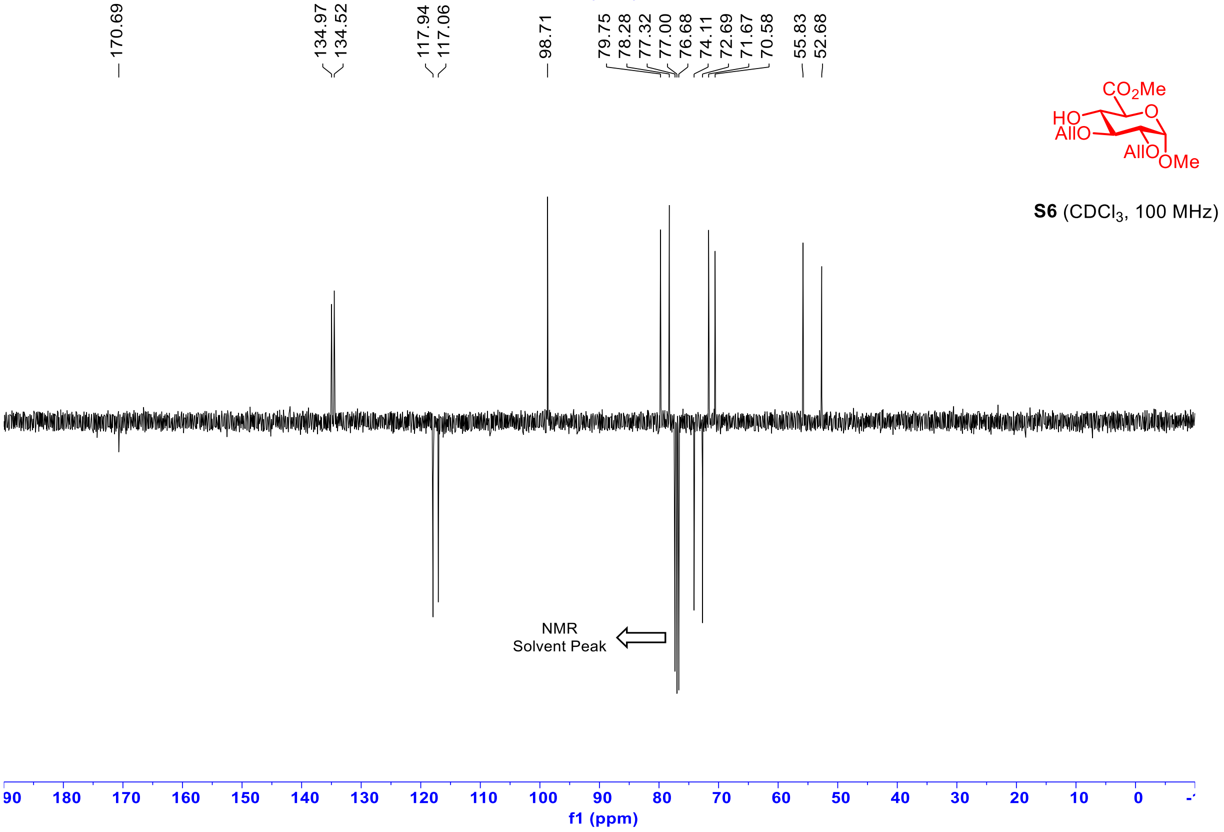
S6 ($CDCl_3$, 100 MHz)
NMR Solvent Peak
f1 (ppm)

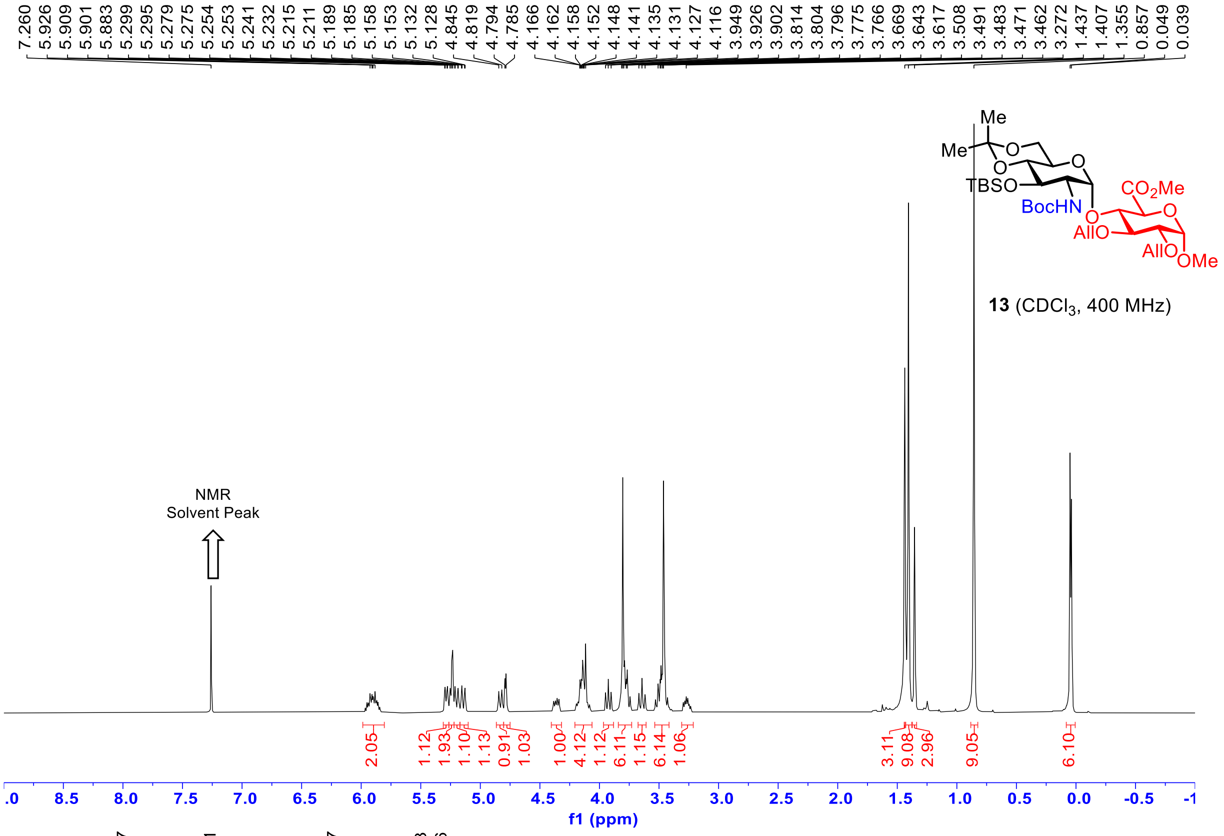
7.260
5.926
5.909
5.901
5.883
5.299
5.295
5.279
5.275
5.254
5.253
5.241
5.232
5.215
5.211
5.189
5.185
5.158
5.153
5.132
5.128
4.845
4.819
4.794
4.785
4.166
4.162
4.158
4.152
4.148
4.141
4.135
4.131
4.127
4.116
3.949
3.926
3.902
3.814
3.804
3.796
3.775
3.766
3.669
3.643
3.617
3.508
3.491
3.483
3.471
3.462
3.272
1.437
1.407
1.355
0.857
0.049
0.039
Me
Me
O
O
O
TBSO
BocHN
O
CO_2Me
O
AllO
AllO
OMe
13 ($CDCl_3$, 400 MHz)
NMR
Solvent Peak
2.05
1.12
1.93
1.10
1.13
0.91
1.03
1.00
4.12
1.12
6.11
1.15
6.14
1.06
3.11
9.08
2.96
9.05
6.10
f1 (ppm)

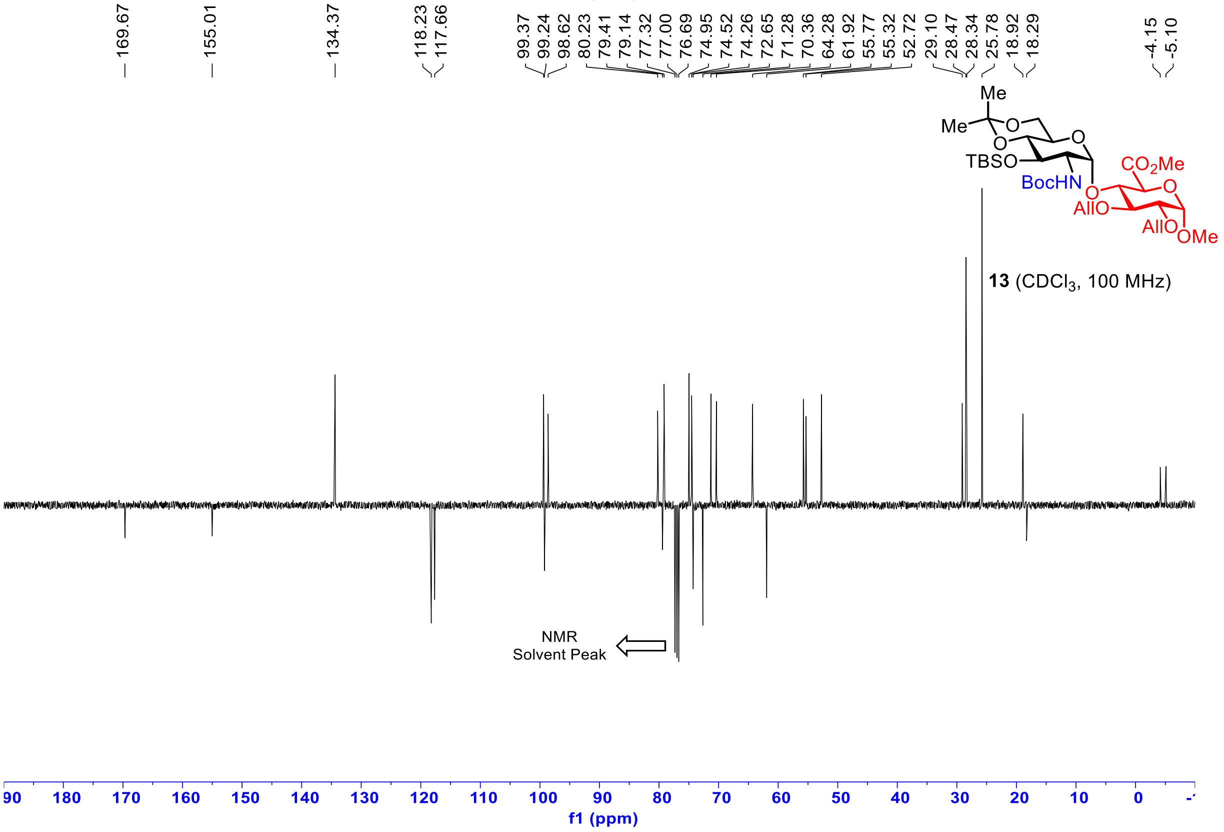
169.67
155.01
134.37
118.23
117.66
99.37
99.24
98.62
80.23
79.41
79.14
77.32
77.00
76.69
74.95
74.52
74.26
72.65
71.28
70.36
64.28
61.92
55.77
55.32
52.72
29.10
28.47
28.34
25.78
18.92
18.29
-4.15
-5.10
Me
Me
O
O
O
TBSO
BocHN
O
CO_2Me
O
AllO
AllO
OMe
13 ($CDCl_3$, 100 MHz)
NMR
Solvent Peak
f1 (ppm)

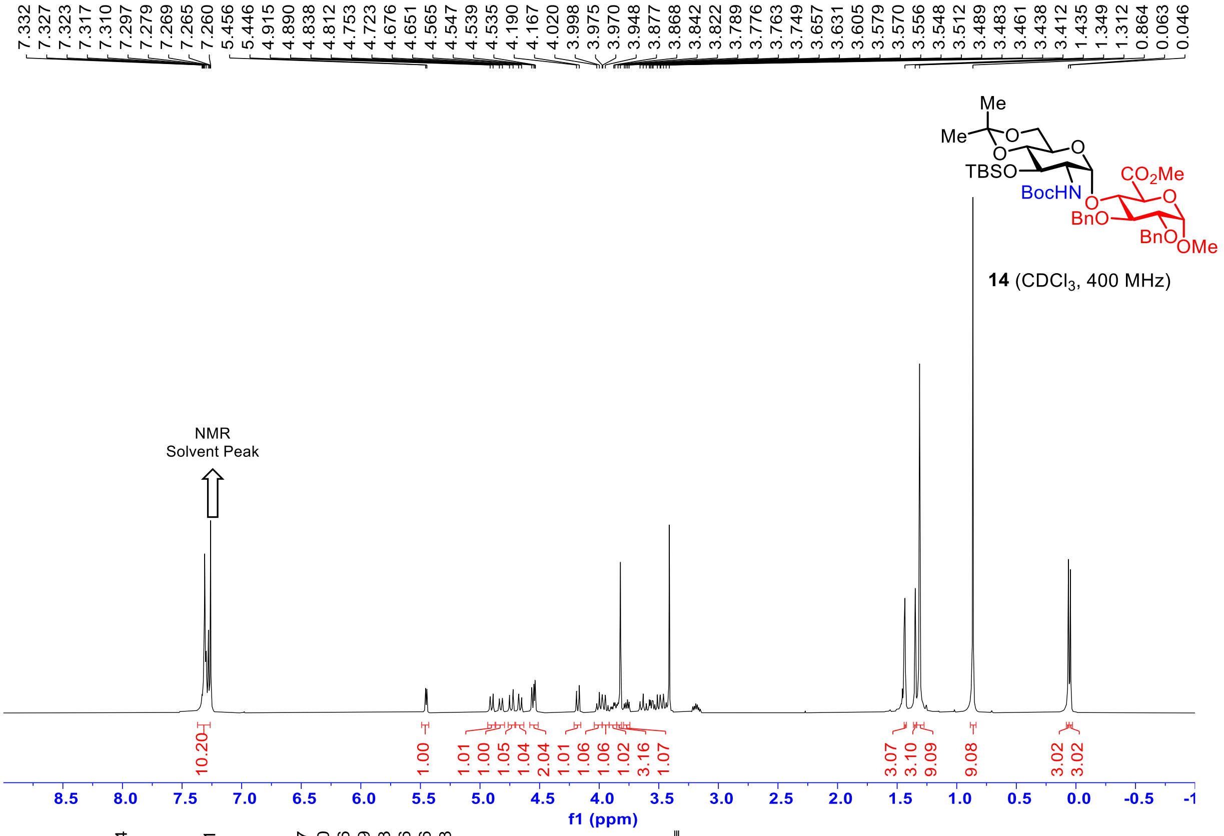
7.332
7.327
7.323
7.317
7.310
7.297
7.279
7.269
7.265
7.260
5.456
5.446
4.915
4.890
4.838
4.812
4.753
4.723
4.676
4.651
4.565
4.547
4.539
4.535
4.190
4.167
4.020
3.998
3.975
3.970
3.948
3.877
3.868
3.842
3.822
3.789
3.776
3.763
3.749
3.657
3.631
3.605
3.579
3.570
3.556
3.548
3.512
3.489
3.483
3.461
3.438
3.412
1.435
1.349
1.312
0.864
0.063
0.046
Me
Me
O
O
O
TBSO
BocHN
O
CO2Me
O
BnO
BnO
OMe
14 (CDCl3, 400 MHz)
NMR
Solvent Peak
10.20
1.00
1.01
1.00
1.05
1.04
2.04
1.01
1.06
1.06
1.02
3.16
1.07
3.07
3.10
9.09
9.08
3.02
3.02
f1 (ppm)

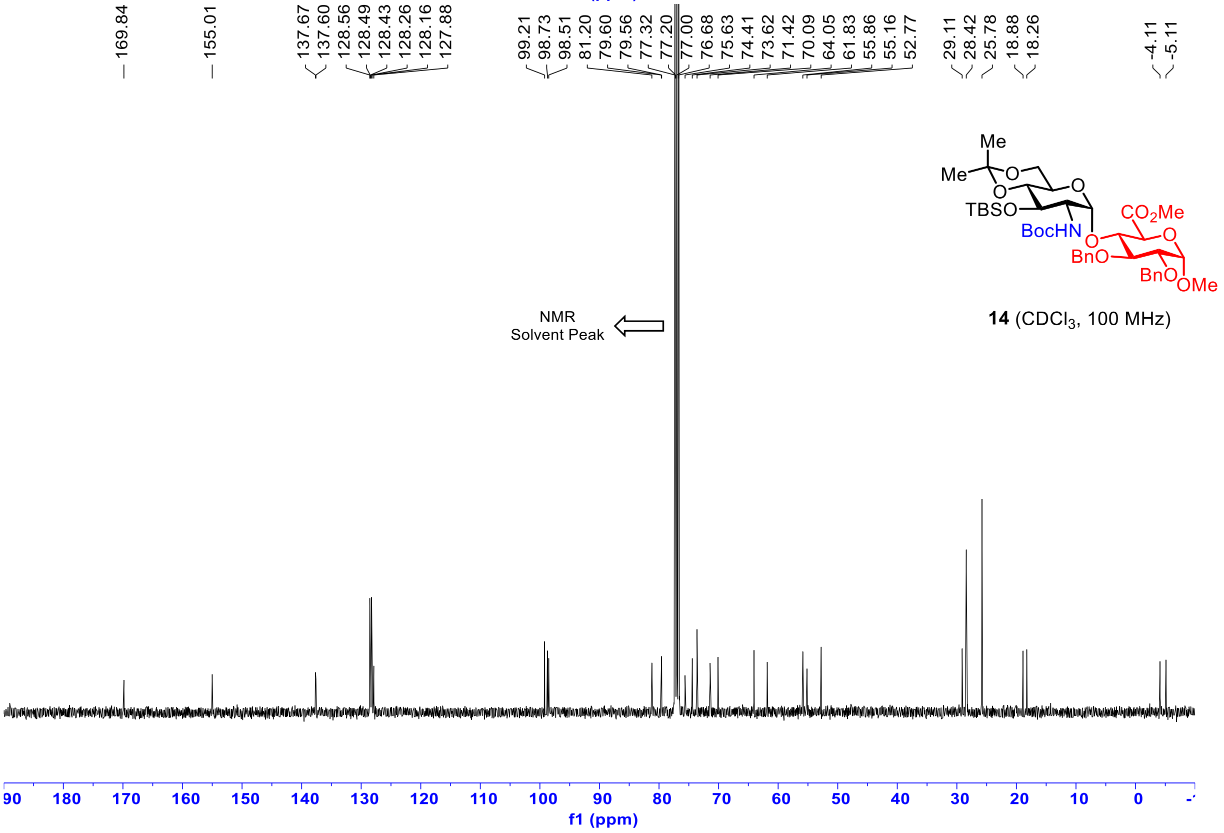
169.84
155.01
137.67
137.60
128.56
128.49
128.43
128.26
128.16
127.88
99.21
98.73
98.51
81.20
79.60
79.56
77.32
77.20
77.00
76.68
75.63
74.41
73.62
71.42
70.09
64.05
61.83
55.86
55.16
52.77
29.11
28.42
25.78
18.88
18.26
-4.11
-5.11
14 (CDCl3, 100 MHz)
NMR
Solvent Peak
f1 (ppm)

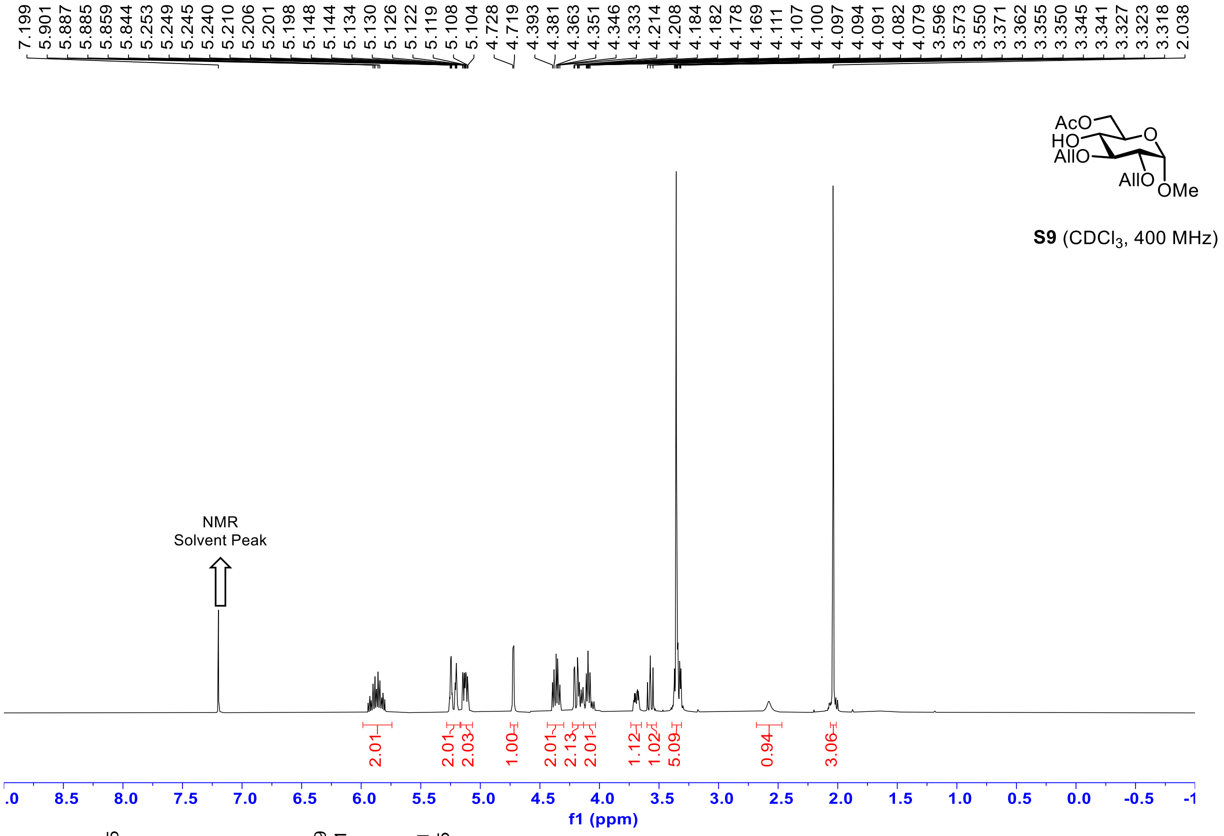

S9 (CDCl3, 400 MHz)
NMR
Solvent Peak
f1 (ppm)

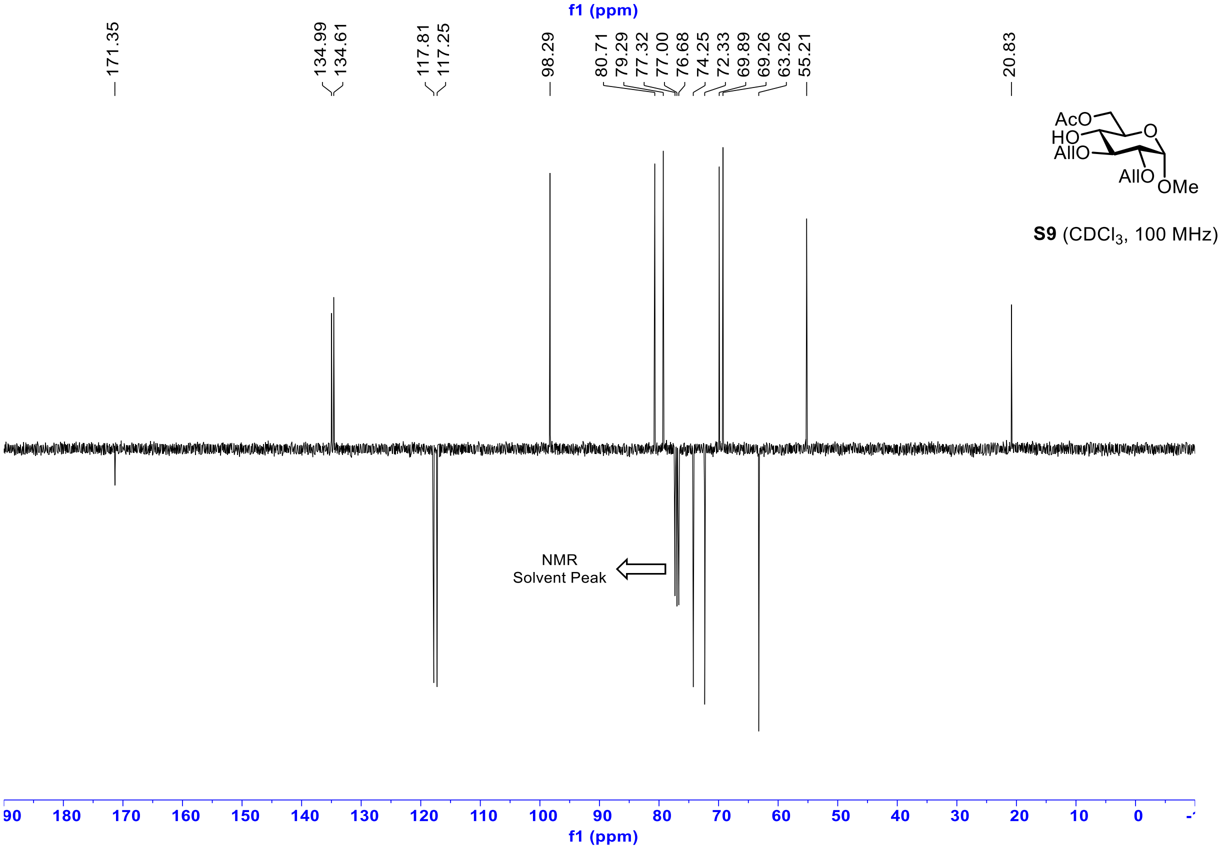

S9 (CDCl3, 100 MHz)
NMR
Solvent Peak
f1 (ppm)

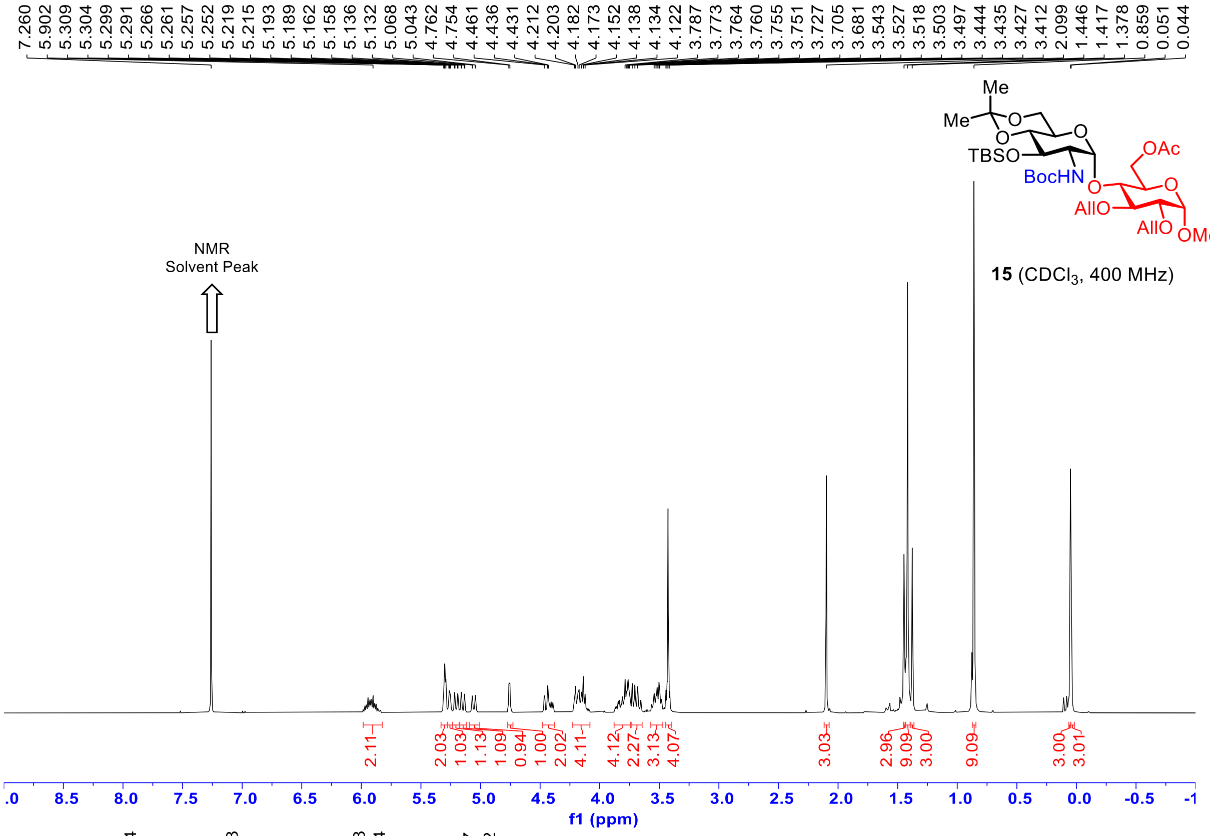
7.260 5.902 5.309 5.304 5.299 5.291 5.266 5.261 5.257 5.252 5.219 5.215 5.193 5.189 5.162 5.158 5.136 5.132 5.068 5.043 4.762 4.754 4.461 4.436 4.431 4.212 4.203 4.182 4.173 4.152 4.138 4.134 4.122 3.787 3.773 3.764 3.760 3.755 3.751 3.727 3.705 3.681 3.543 3.527 3.518 3.503 3.497 3.444 3.435 3.427 3.412 2.099 1.446 1.417 1.378 0.859 0.051 0.044
NMR
Solvent Peak
Me
Me
O
O
O
TBSO
BocHN
O
OAc
O
AllO
AllO
OMe
15 (CDCl3, 400 MHz)
2.11 2.03 1.03 1.13 1.09 0.94 1.00 2.02 4.11 4.12 2.27 3.13 4.07 3.03 2.96 9.09 3.00 9.09 3.00 3.01
f1 (ppm)

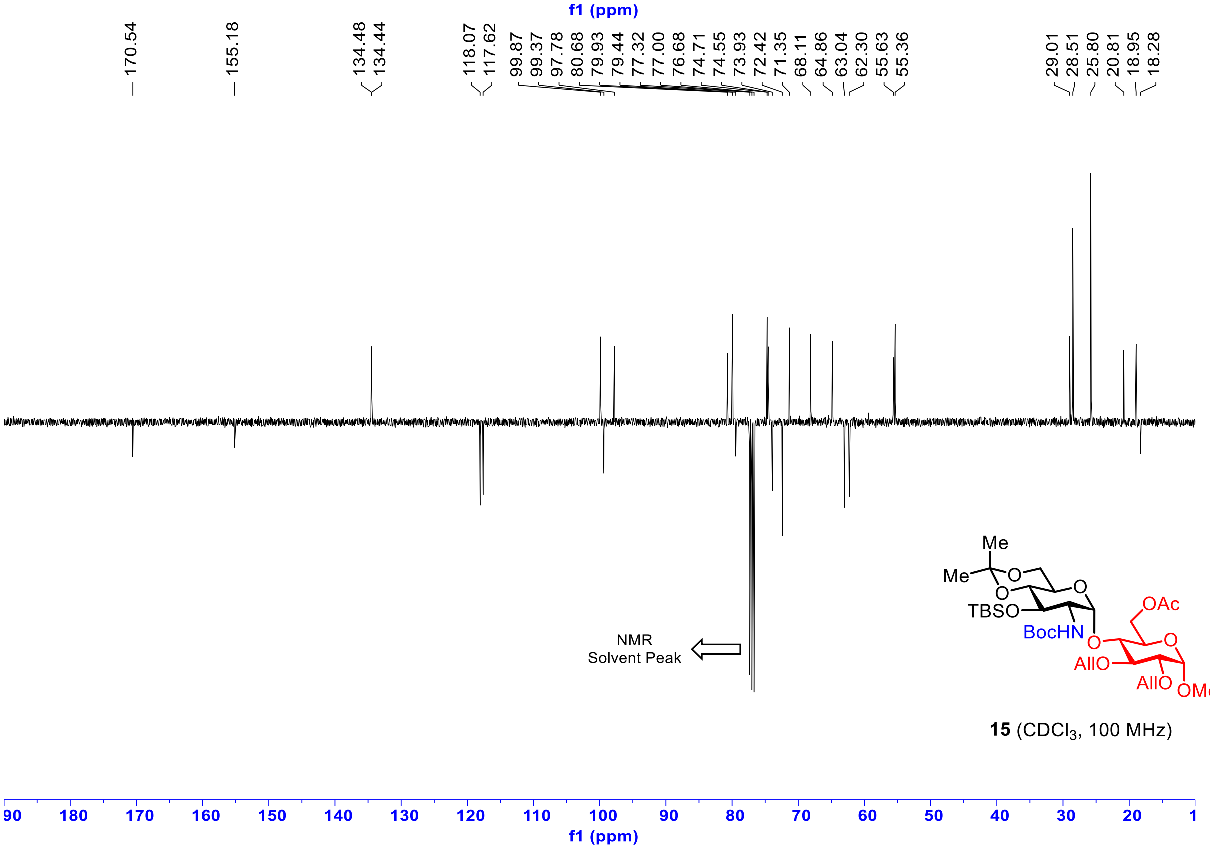
170.54 155.18 134.48 134.44 118.07 117.62 99.87 99.37 97.78 80.68 79.93 79.44 77.32 77.00 76.68 74.71 74.55 73.93 72.42 71.35 68.11 64.86 63.04 62.30 55.63 55.36 29.01 28.51 25.80 20.81 18.95 18.28
Me
Me
O
O
O
TBSO
BocHN
O
OAc
O
AllO
AllO
OMe
NMR
Solvent Peak
15 (CDCl3, 100 MHz)
f1 (ppm)

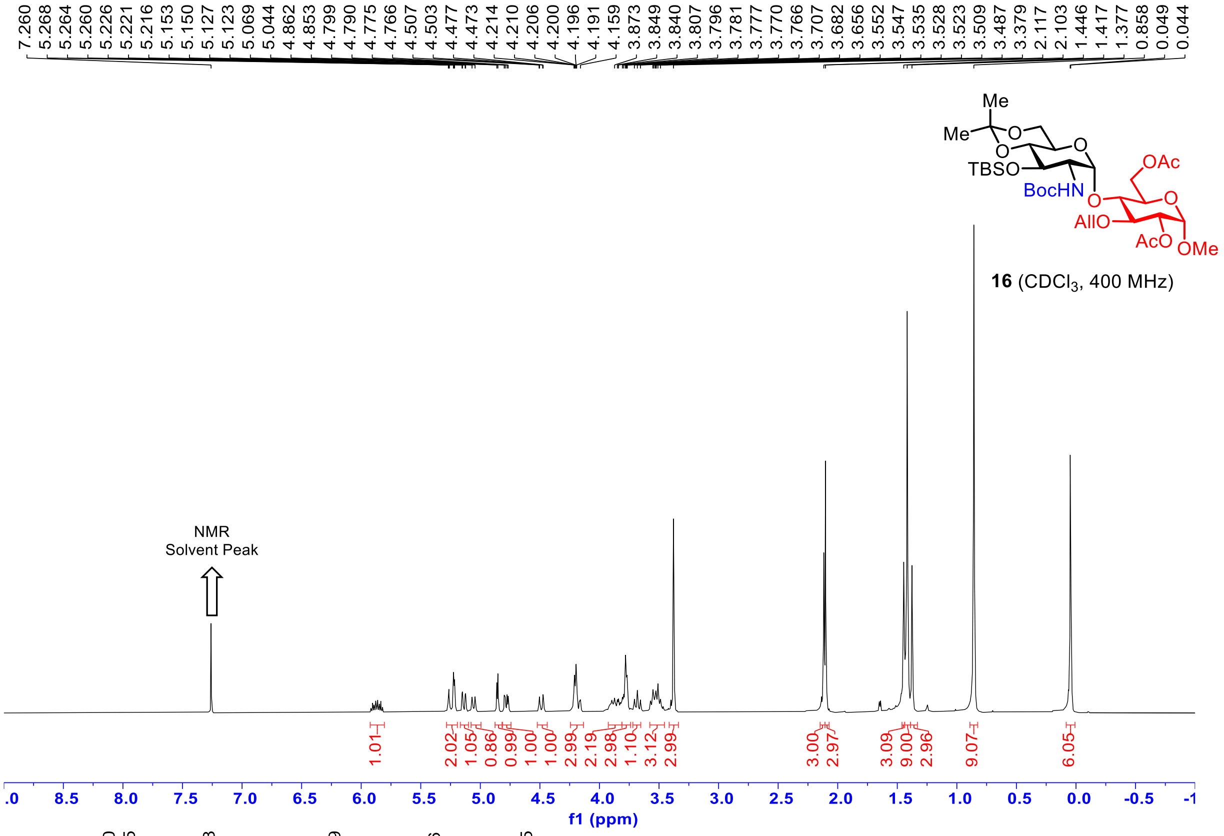
7.260
5.268
5.264
5.260
5.226
5.221
5.216
5.153
5.150
5.127
5.123
5.069
5.044
4.862
4.853
4.799
4.790
4.775
4.766
4.507
4.503
4.477
4.473
4.214
4.210
4.206
4.200
4.196
4.191
4.159
3.873
3.849
3.840
3.807
3.796
3.781
3.777
3.770
3.766
3.707
3.682
3.656
3.552
3.547
3.535
3.528
3.523
3.509
3.487
3.379
2.117
2.103
1.446
1.417
1.377
0.858
0.049
0.044
Me
Me
O
O
O
TBSO
BocHN
O
OAc
O
AllO
AcO
OMe
16 (CDCl$_3$, 400 MHz)
NMR Solvent Peak
1.01
2.02
1.05
0.86
0.99
1.00
1.00
2.99
2.19
2.98
1.10
3.12
2.99
3.00
2.97
3.09
9.00
2.96
9.07
6.05
f1 (ppm)

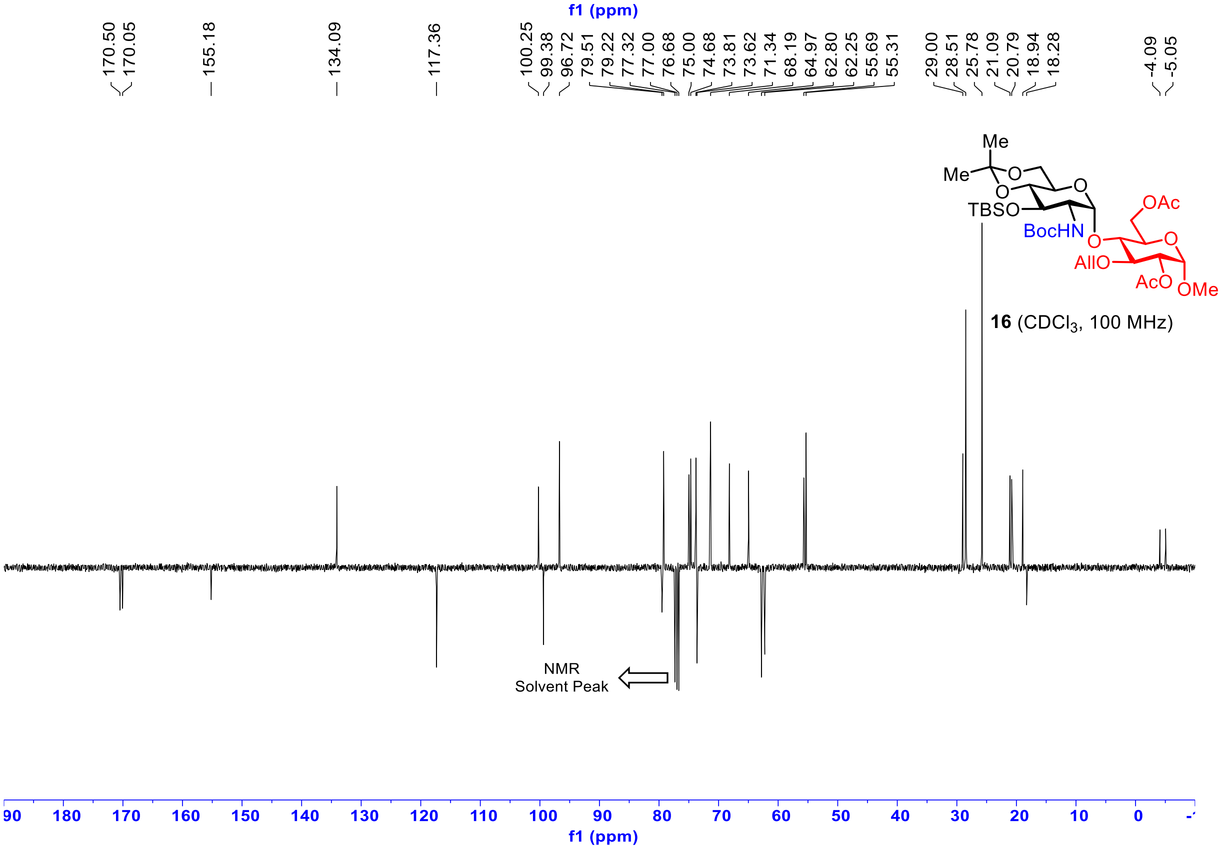
170.50
170.05
155.18
134.09
117.36
100.25
99.38
96.72
79.51
79.22
77.32
77.00
76.68
75.00
74.68
73.81
73.62
71.34
68.19
64.97
62.80
62.25
55.69
55.31
29.00
28.51
25.78
21.09
20.79
18.94
18.28
-4.09
-5.05
Me
Me
O
O
O
TBSO
BocHN
O
OAc
O
AllO
AcO
OMe
16 (CDCl$_3$, 100 MHz)
NMR Solvent Peak
f1 (ppm)

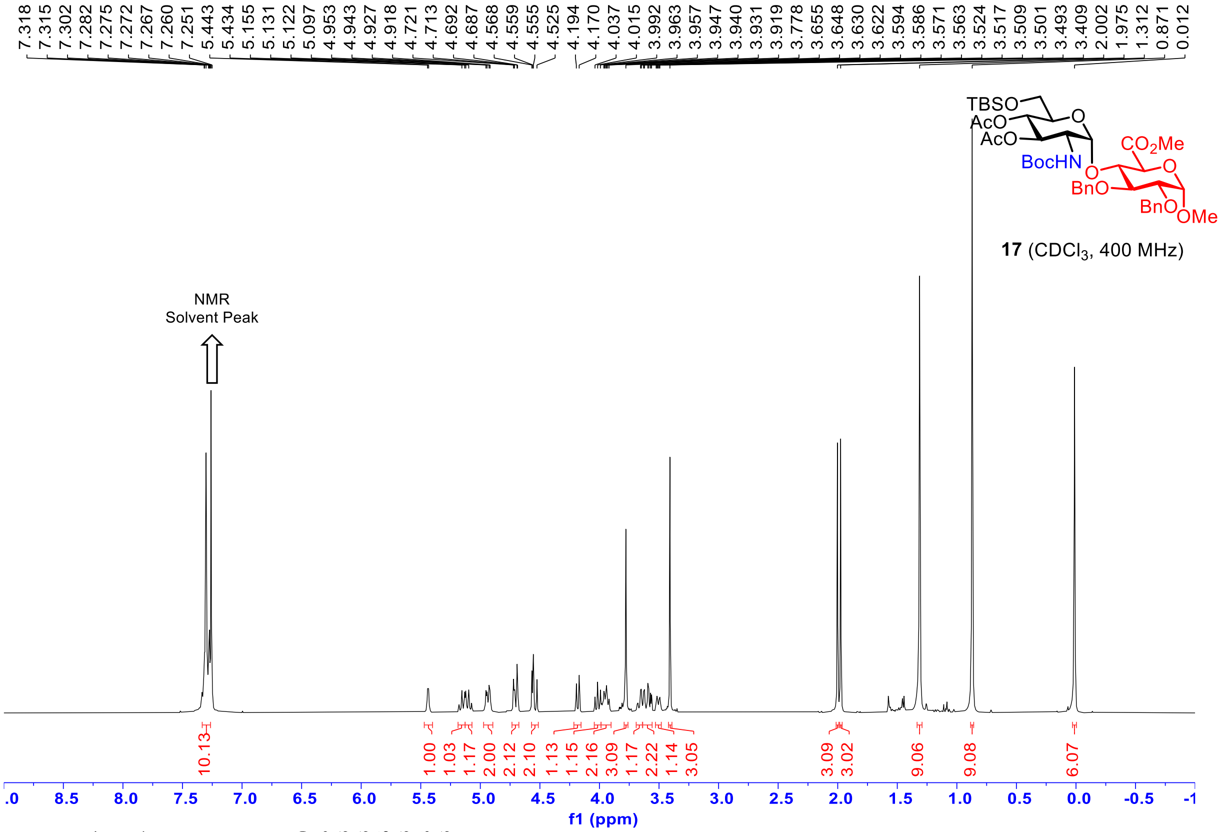
7.318 7.315 7.302 7.282 7.275 7.272 7.267 7.260 7.251 5.443 5.434 5.155 5.131 5.122 5.097 4.953 4.943 4.927 4.918 4.721 4.713 4.692 4.687 4.568 4.559 4.555 4.525 4.194 4.170 4.037 4.015 3.992 3.963 3.957 3.947 3.940 3.931 3.919 3.778 3.655 3.648 3.630 3.622 3.594 3.586 3.571 3.563 3.524 3.517 3.509 3.501 3.493 3.409 2.002 1.975 1.312 0.871 0.012
TBSO
AcO
AcO
BocHN
O
CO2Me
BnO
BnO
OMe
17 (CDCl3, 400 MHz)
NMR
Solvent Peak
10.13
1.00 1.03 1.17 2.00 2.12 2.10 1.13 1.15 2.16 3.09 1.17 2.22 1.14 3.05
3.09 3.02
9.06
9.08
6.07
.0 8.5 8.0 7.5 7.0 6.5 6.0 5.5 5.0 4.5 4.0 3.5 3.0 2.5 2.0 1.5 1.0 0.5 0.0 -0.5 -1
f1 (ppm)

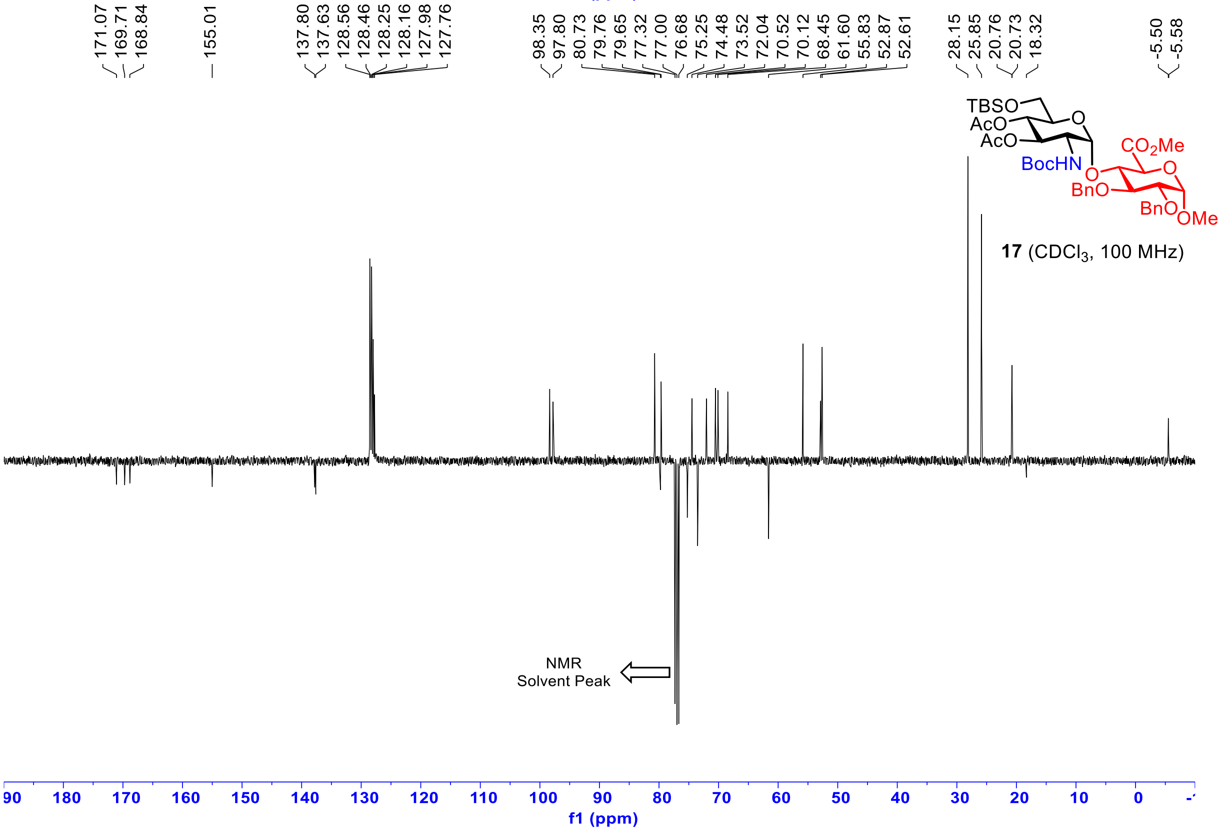
171.07 169.71 168.84 155.01 137.80 137.63 128.56 128.46 128.25 128.16 127.98 127.76 98.35 97.80 80.73 79.76 79.65 77.32 77.00 76.68 75.25 74.48 73.52 72.04 70.52 70.12 68.45 61.60 55.83 52.87 52.61 28.15 25.85 20.76 20.73 18.32 -5.50 -5.58
TBSO
AcO
AcO
BocHN
O
CO2Me
BnO
BnO
OMe
17 (CDCl3, 100 MHz)
NMR
Solvent Peak
90 180 170 160 150 140 130 120 110 100 90 80 70 60 50 40 30 20 10 0
f1 (ppm)

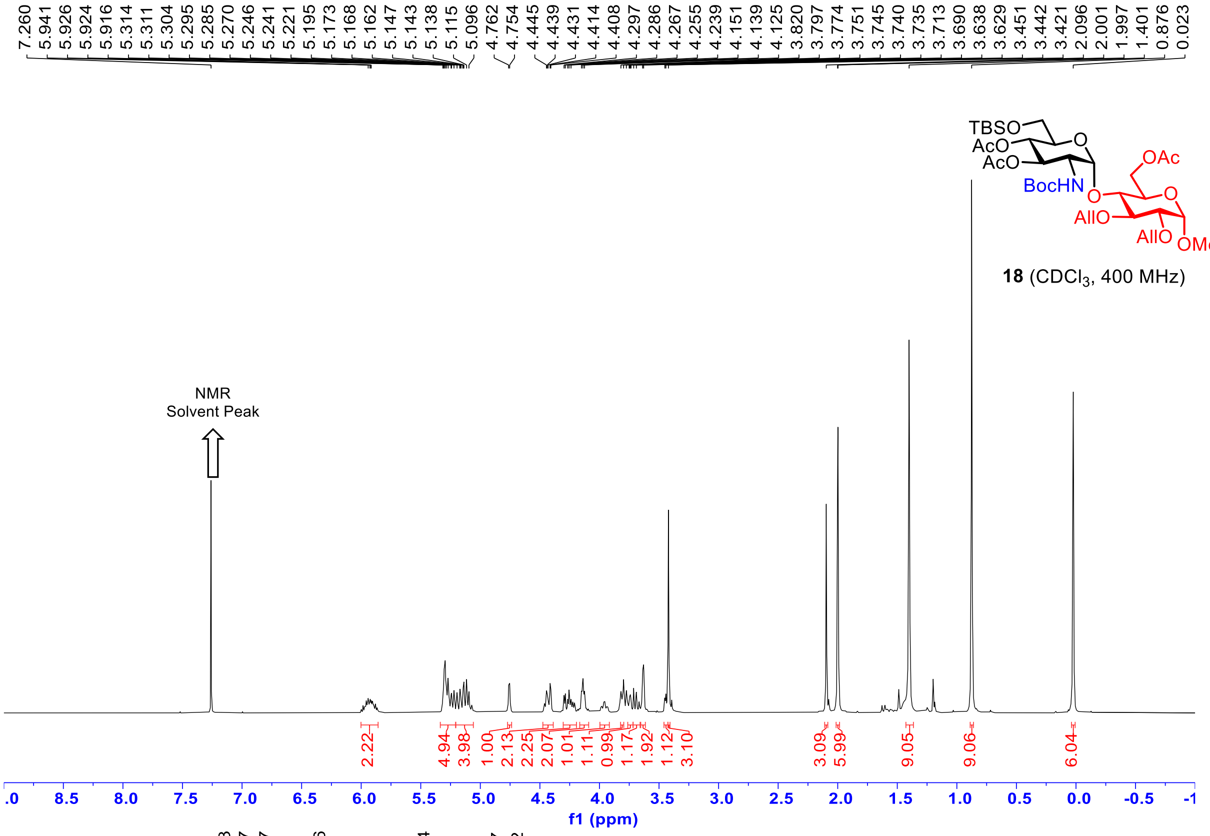
7.260 5.941 5.926 5.924 5.916 5.314 5.311 5.304 5.295 5.285 5.270 5.246 5.241 5.221 5.195 5.173 5.168 5.162 5.147 5.143 5.138 5.115 5.096 4.762 4.754 4.445 4.439 4.431 4.414 4.408 4.297 4.286 4.267 4.255 4.239 4.151 4.139 4.125 3.820 3.797 3.774 3.751 3.745 3.740 3.735 3.713 3.690 3.638 3.629 3.451 3.442 3.421 2.096 2.001 1.997 1.401 0.876 0.023
TBSO
AcO
AcO
BocHN
OAc
AllO
AllO
OMe
18 (CDCl3, 400 MHz)
NMR
Solvent Peak
2.22 4.94 3.98 1.00 2.13 2.25 2.07 1.01 1.11 0.99 1.17 1.92 1.12 3.10 3.09 5.99 9.05 9.06 6.04
f1 (ppm)

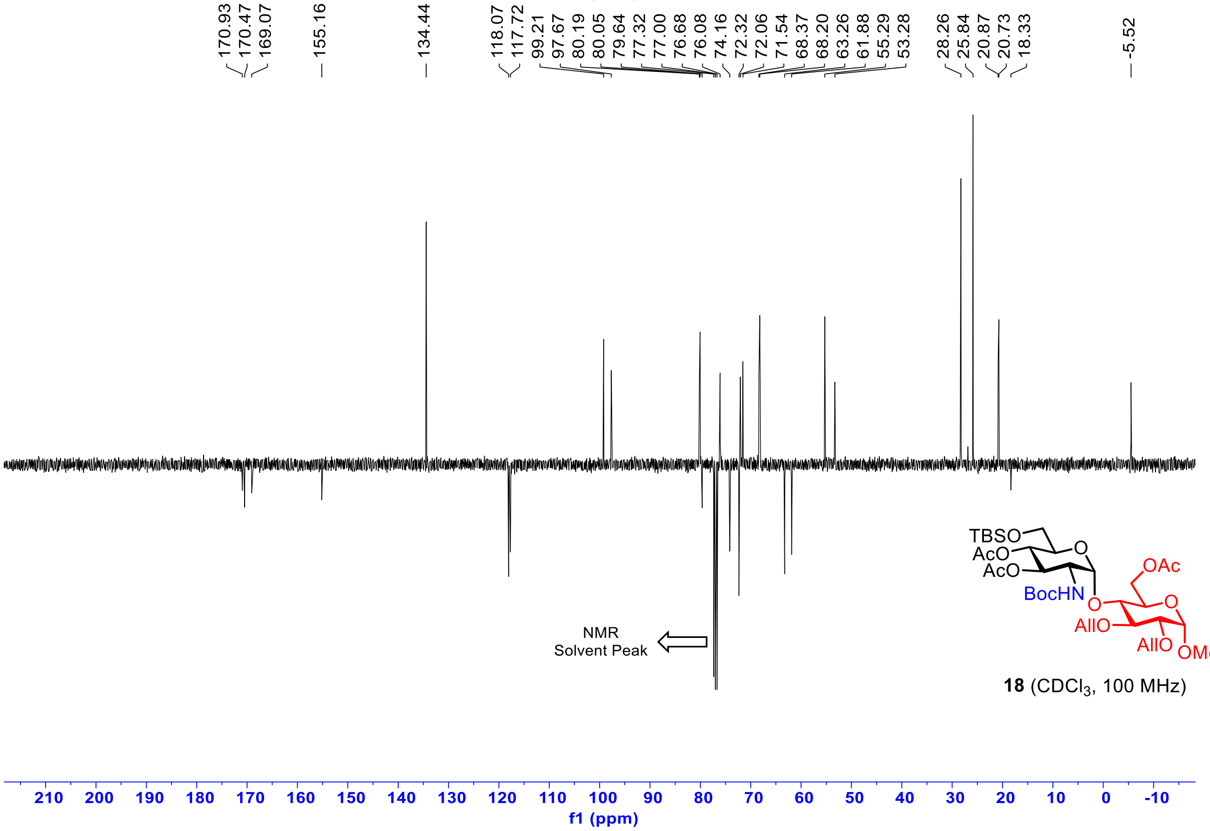
170.93 170.47 169.07 155.16 134.44 118.07 117.72 99.21 97.67 80.19 80.05 79.64 77.32 77.00 76.68 76.08 74.16 72.32 72.06 71.54 68.37 68.20 63.26 61.88 55.29 53.28 28.26 25.84 20.87 20.73 18.33 -5.52
TBSO
AcO
AcO
BocHN
OAc
AllO
AllO
OMe
NMR
Solvent Peak
18 (CDCl3, 100 MHz)
f1 (ppm)

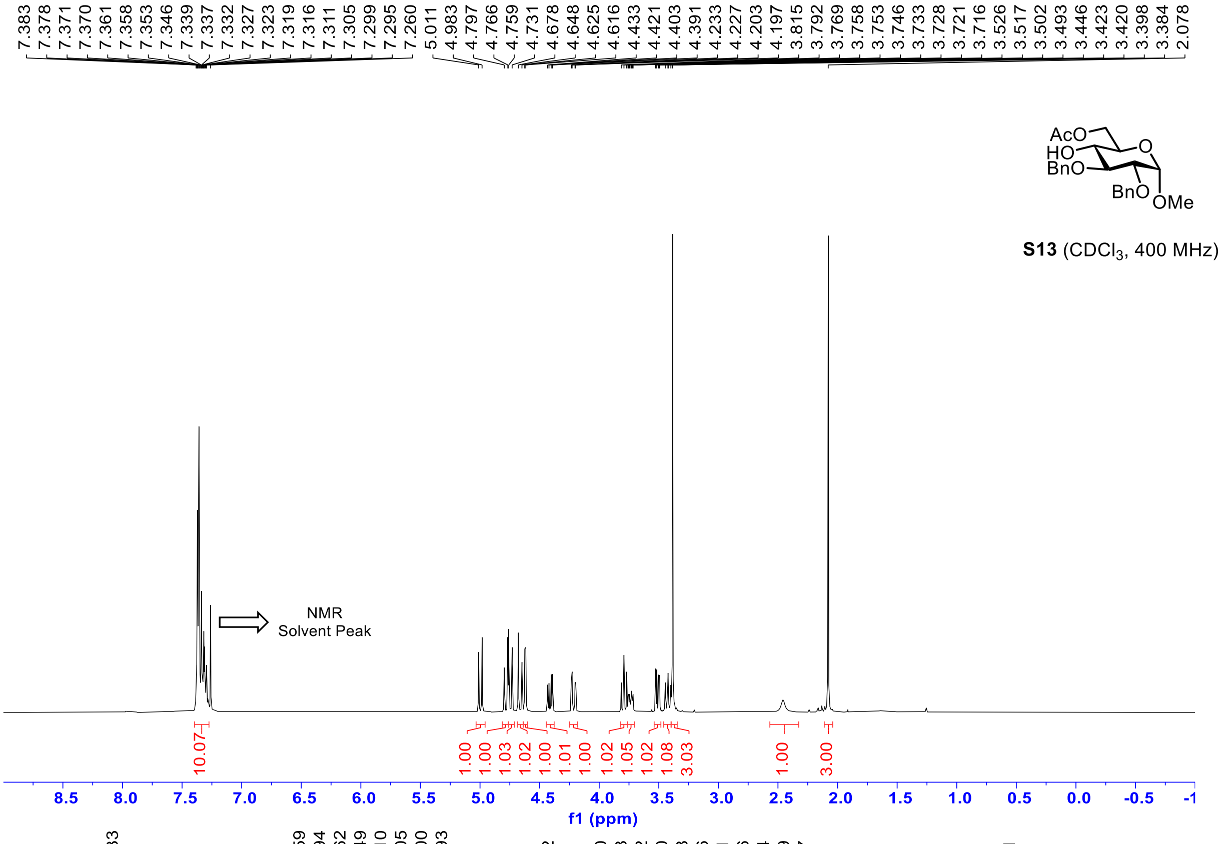
7.383
7.378
7.371
7.370
7.361
7.358
7.353
7.346
7.339
7.337
7.332
7.327
7.323
7.319
7.316
7.311
7.305
7.299
7.295
7.260
5.011
4.983
4.797
4.766
4.759
4.731
4.678
4.648
4.625
4.616
4.433
4.421
4.403
4.391
4.233
4.227
4.203
4.197
3.815
3.792
3.769
3.758
3.753
3.746
3.733
3.728
3.721
3.716
3.526
3.517
3.502
3.493
3.446
3.423
3.420
3.398
3.384
2.078
AcO
HO
BnO
O
BnO
OMe
S13 (CDCl3, 400 MHz)
NMR
Solvent Peak
10.07
1.00
1.00
1.03
1.02
1.00
1.01
1.00
1.02
1.05
1.02
1.08
3.03
1.00
3.00
f1 (ppm)

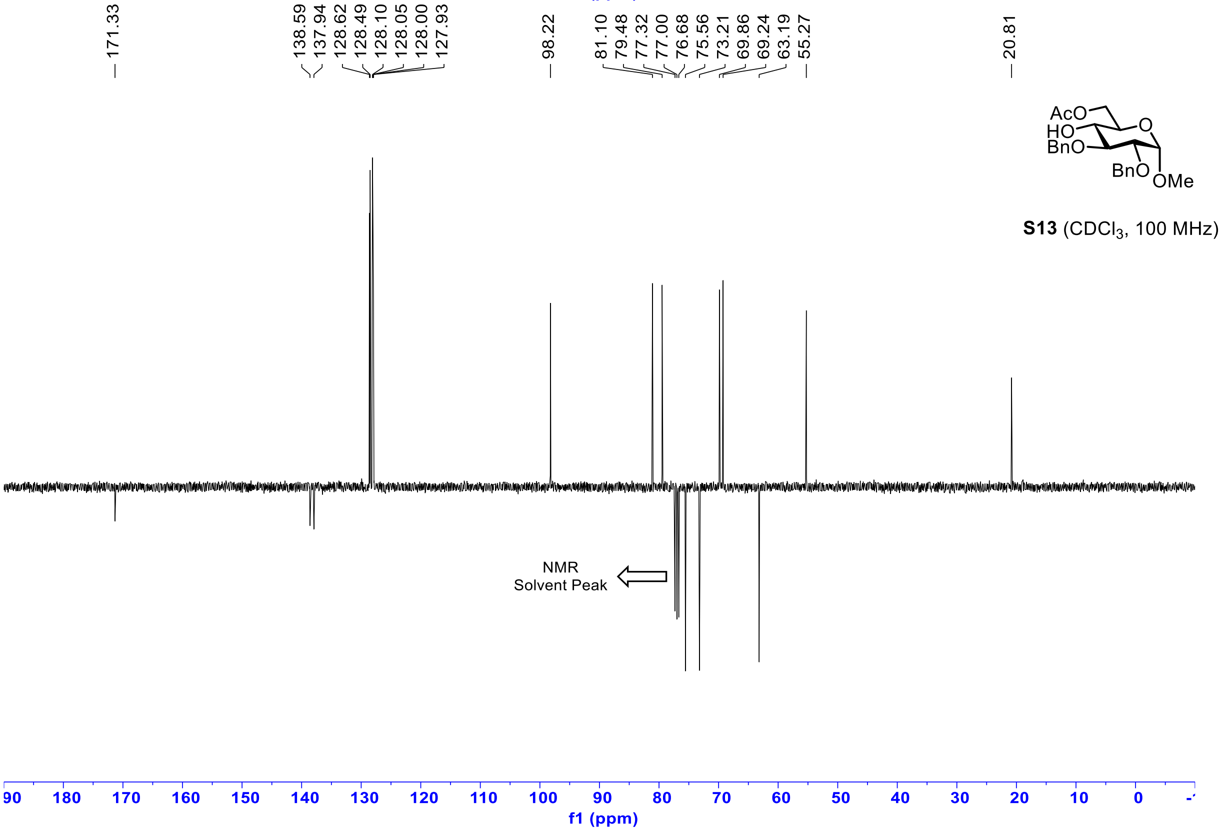
171.33
138.59
137.94
128.62
128.49
128.10
128.05
128.00
127.93
98.22
81.10
79.48
77.32
77.00
76.68
75.56
73.21
69.86
69.24
63.19
55.27
20.81
AcO
HO
BnO
O
BnO
OMe
S13 (CDCl3, 100 MHz)
NMR
Solvent Peak
f1 (ppm)

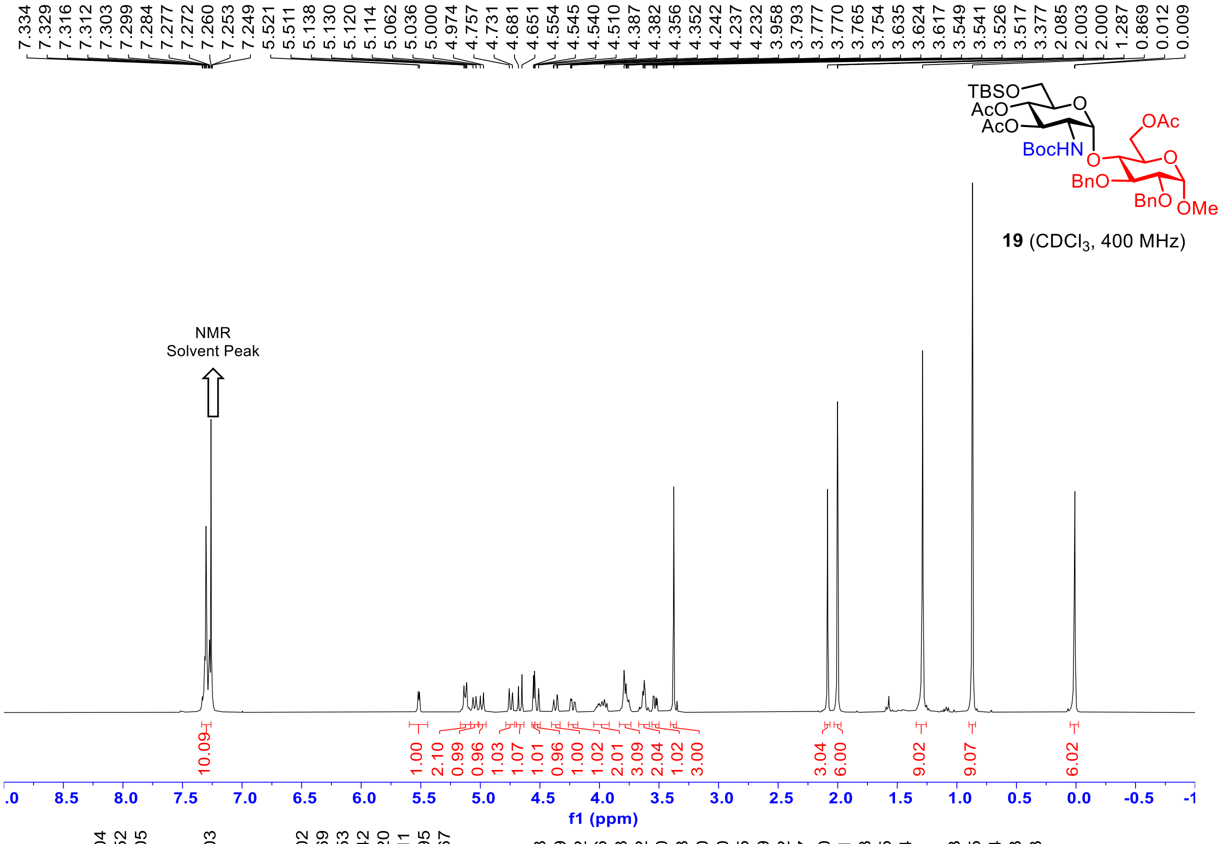
7.334
7.329
7.316
7.312
7.303
7.299
7.284
7.277
7.272
7.260
7.253
7.249
5.521
5.511
5.138
5.130
5.120
5.114
5.062
5.036
5.000
4.974
4.757
4.731
4.681
4.651
4.554
4.545
4.540
4.510
4.387
4.382
4.356
4.352
4.242
4.237
4.232
3.958
3.793
3.777
3.770
3.765
3.754
3.635
3.624
3.617
3.549
3.541
3.526
3.517
3.377
2.085
2.003
2.000
1.287
0.869
0.012
0.009
TBSO
AcO
AcO
O
BocHN
O
OAc
O
BnO
BnO
OMe
19 (CDCl3, 400 MHz)
NMR
Solvent Peak
10.09
1.00
2.10
0.99
0.96
1.03
1.07
1.01
0.96
1.00
1.02
2.01
3.09
2.04
1.02
3.00
3.04
6.00
9.02
9.07
6.02
f1 (ppm)

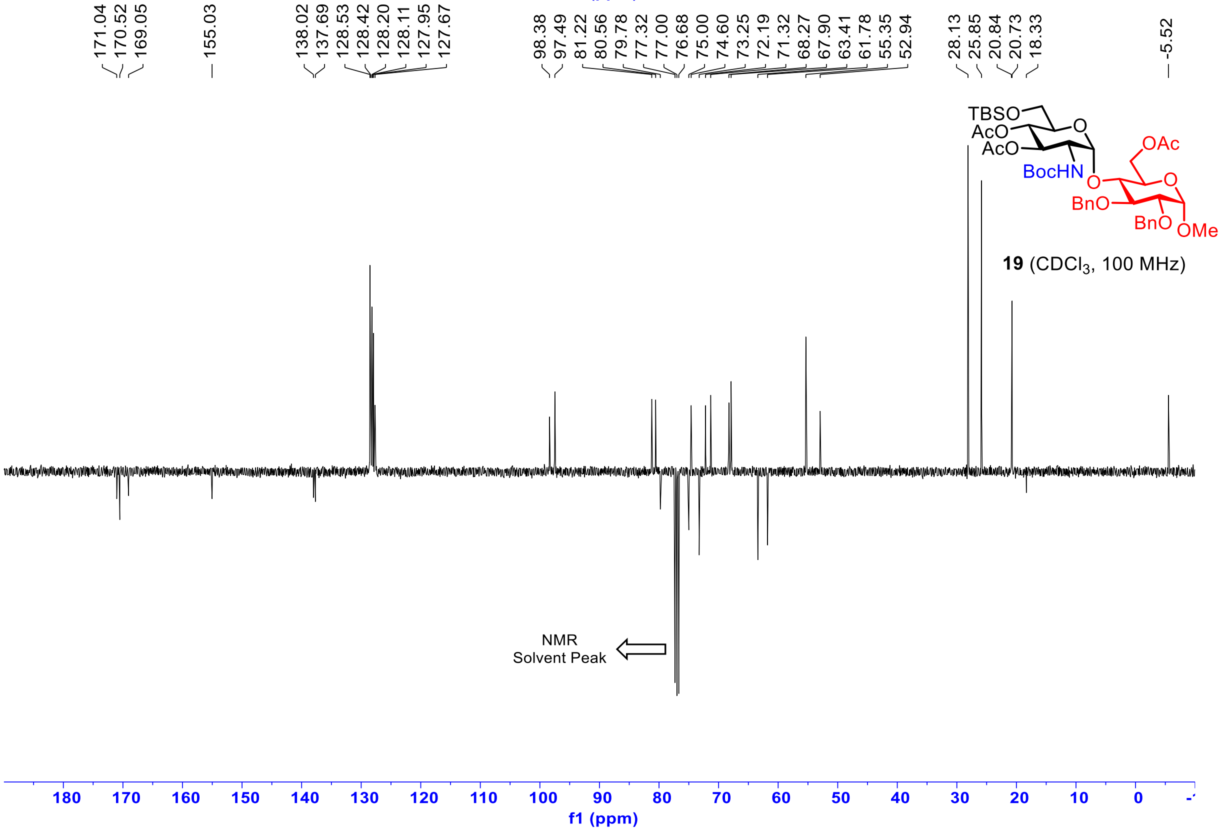
171.04
170.52
169.05
155.03
138.02
137.69
128.53
128.42
128.20
128.11
127.95
127.67
98.38
97.49
81.22
80.56
79.78
77.32
77.00
76.68
75.00
74.60
73.25
72.19
71.32
68.27
67.90
63.41
61.78
55.35
52.94
28.13
25.85
20.84
20.73
18.33
-5.52
TBSO
AcO
AcO
O
BocHN
O
OAc
O
BnO
BnO
OMe
19 (CDCl3, 100 MHz)
NMR
Solvent Peak
f1 (ppm)

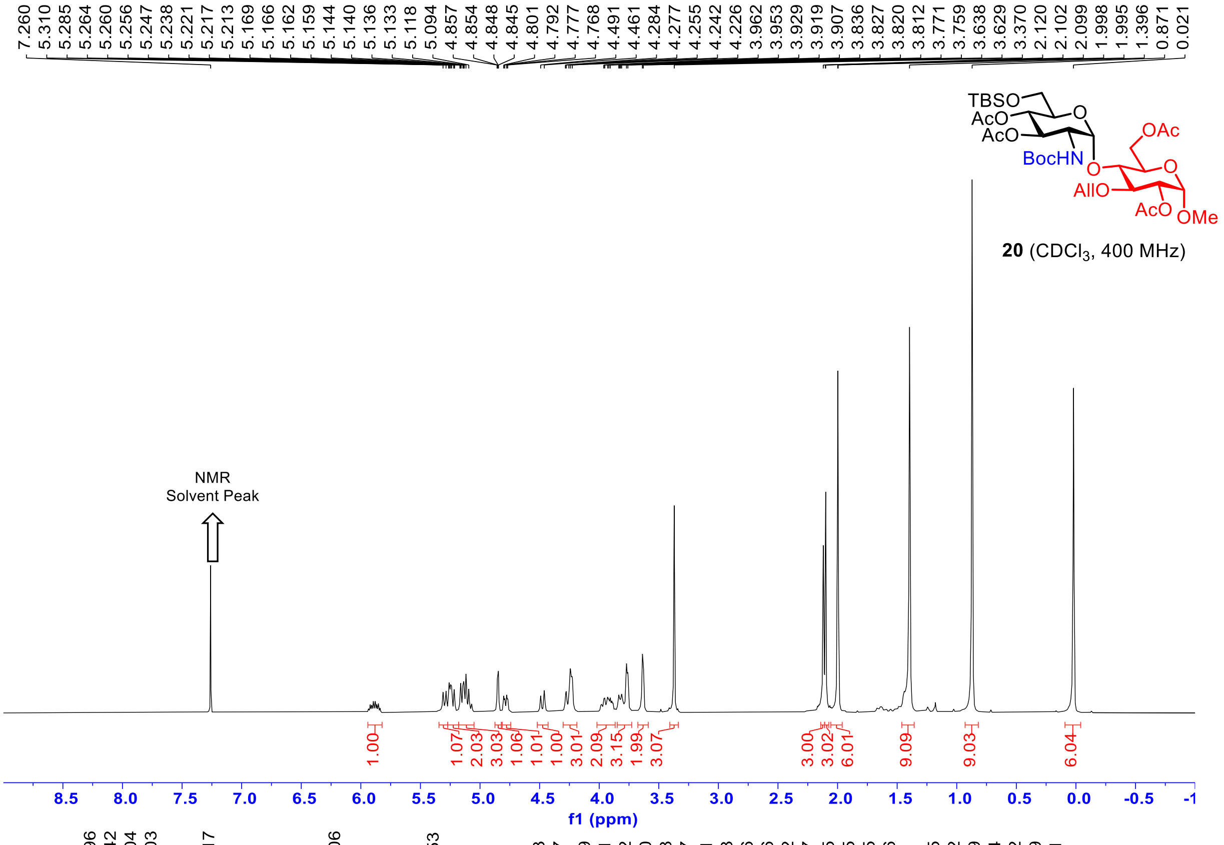
7.260 5.310 5.285 5.264 5.260 5.256 5.247 5.238 5.221 5.217 5.213 5.169 5.166 5.162 5.159 5.144 5.140 5.136 5.133 5.118 5.094 4.857 4.854 4.848 4.845 4.801 4.792 4.777 4.768 4.491 4.461 4.284 4.277 4.255 4.242 4.226 3.962 3.953 3.929 3.919 3.907 3.836 3.827 3.820 3.812 3.771 3.759 3.638 3.629 3.370 2.120 2.102 2.099 1.998 1.995 1.396 0.871 0.021
TBSO
AcO
AcO
BocHN
O
OAc
AllO
AcO
OMe
20 (CDCl3, 400 MHz)
NMR
Solvent Peak
1.00 1.07 2.03 3.03 1.06 1.01 1.00 3.01 2.09 3.15 1.99 3.07 3.00 3.02 6.01 9.09 9.03 6.04
f1 (ppm)

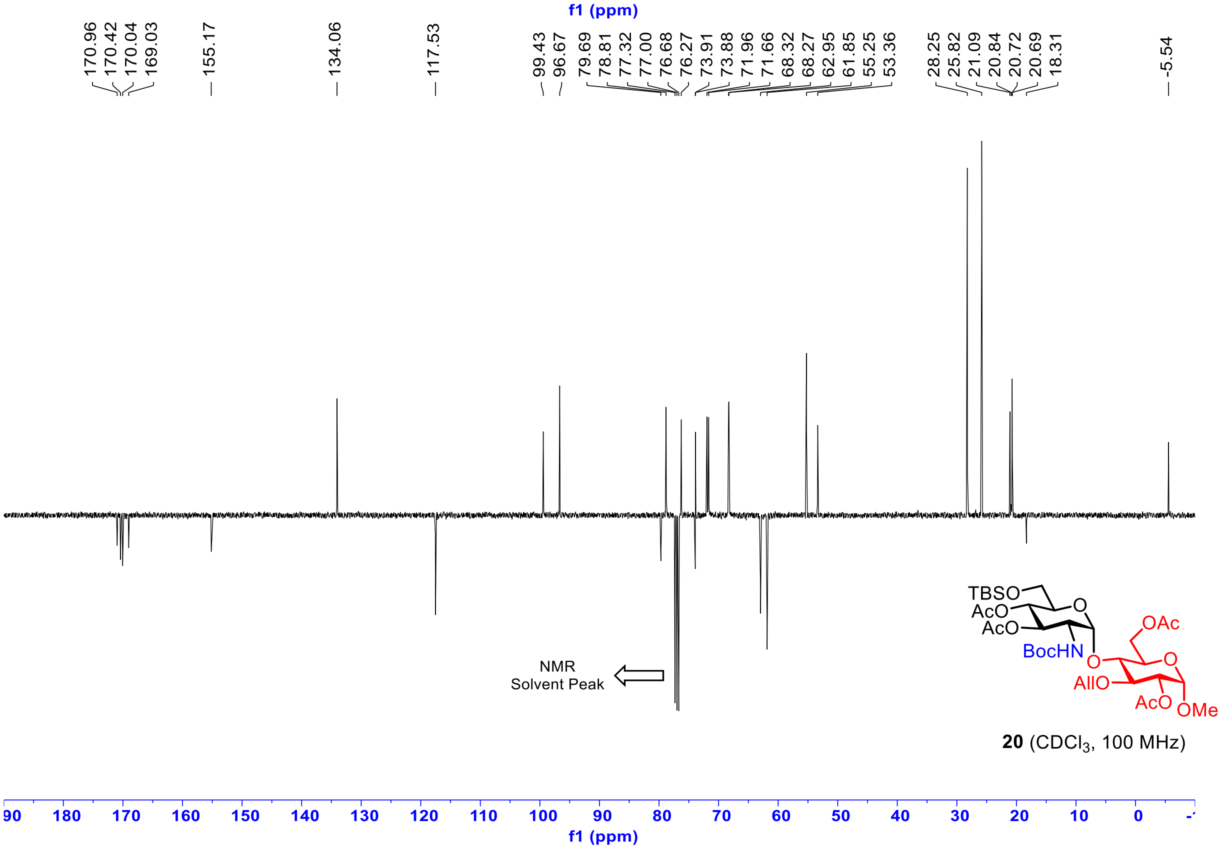
170.96 170.42 170.04 169.03 155.17 134.06 117.53 99.43 96.67 79.69 78.81 77.32 77.00 76.68 76.27 73.91 73.88 71.96 71.66 68.32 68.27 62.95 61.85 55.25 53.36 28.25 25.82 21.09 20.84 20.72 20.69 18.31 -5.54
TBSO
AcO
AcO
BocHN
O
OAc
AllO
AcO
OMe
20 (CDCl3, 100 MHz)
NMR
Solvent Peak
f1 (ppm)

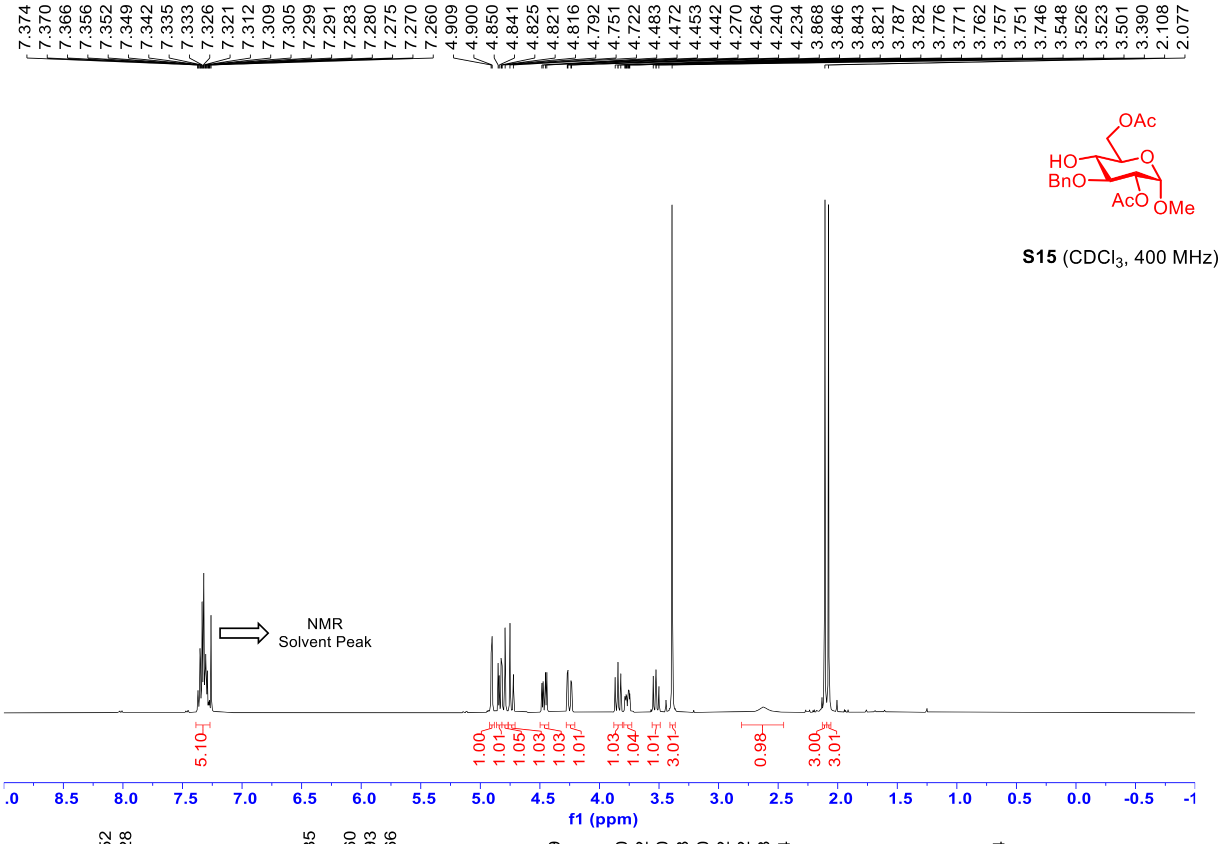

S15 ($CDCl_3$, 400 MHz)

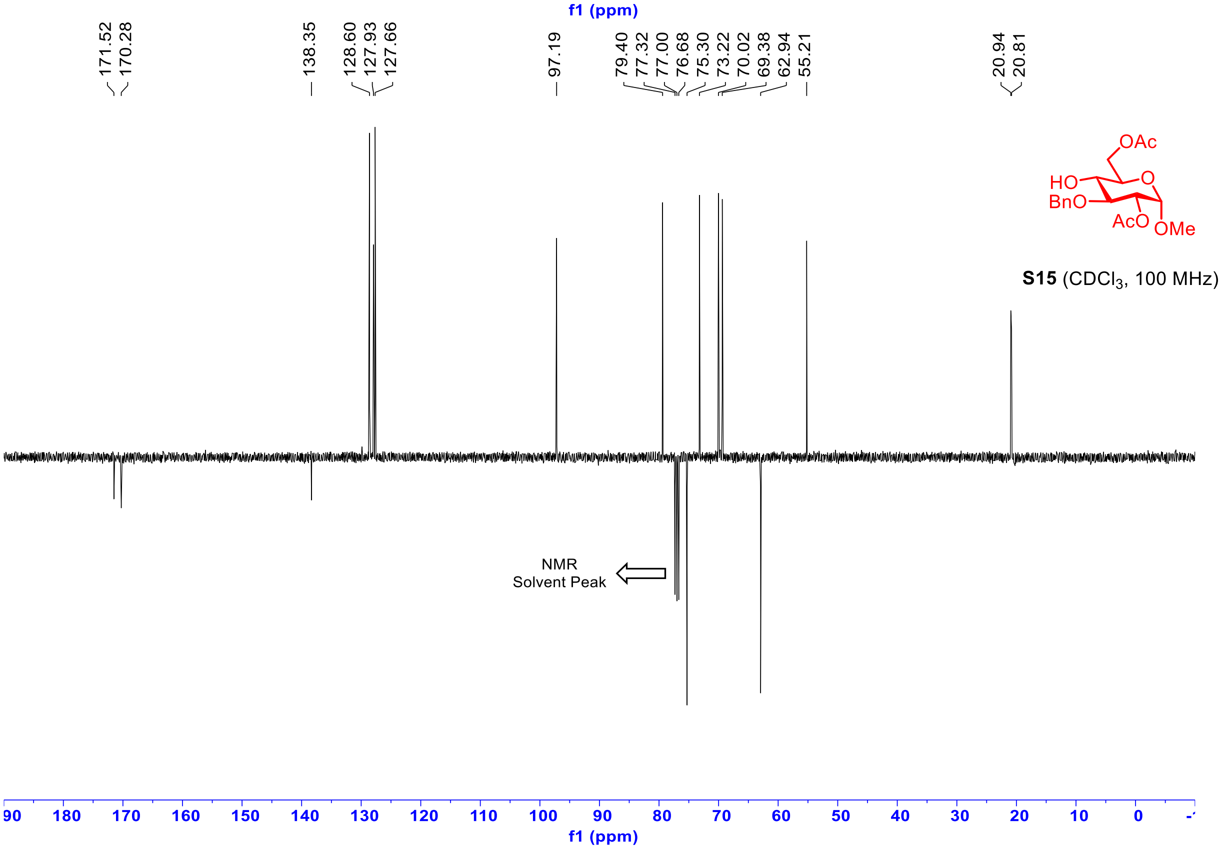

S15 ($CDCl_3$, 100 MHz)

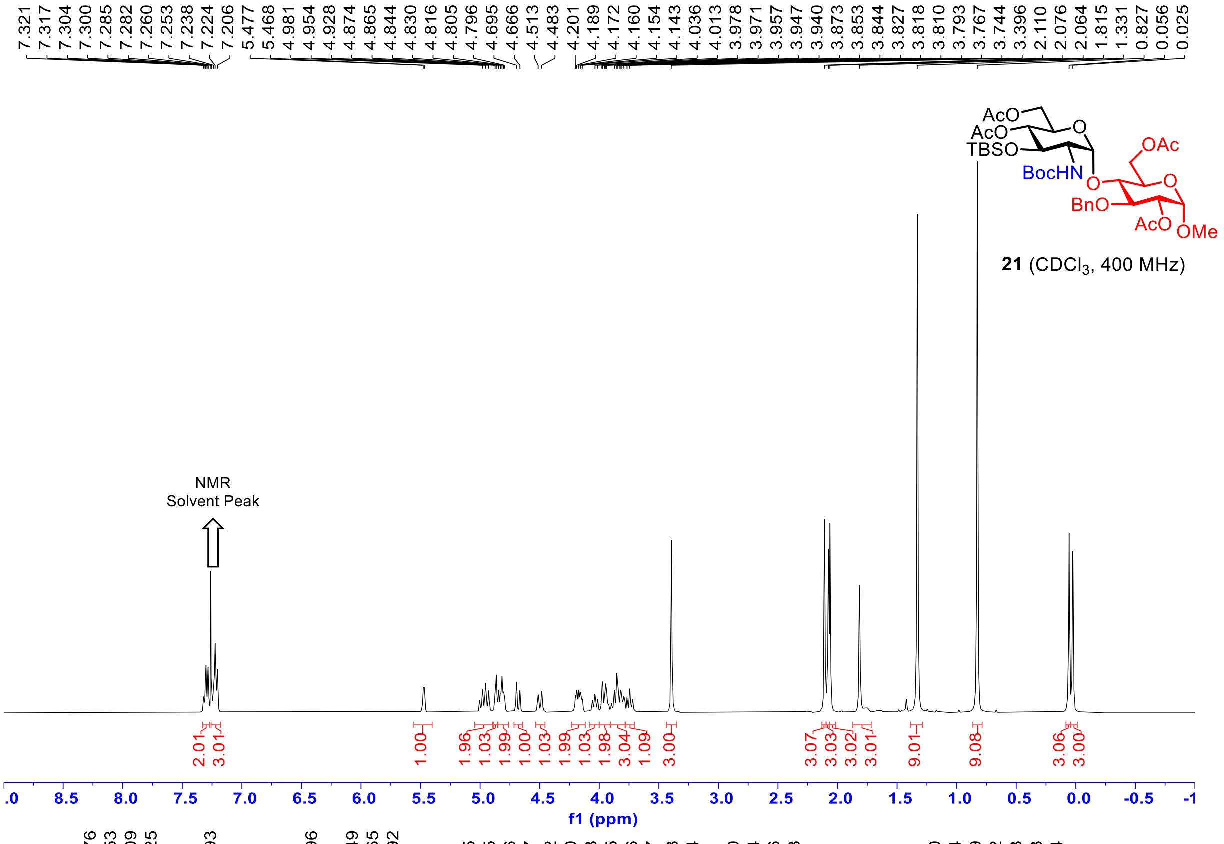
7.321
7.317
7.304
7.300
7.285
7.282
7.260
7.253
7.238
7.224
7.206
5.477
5.468
4.981
4.954
4.928
4.874
4.865
4.844
4.830
4.816
4.805
4.796
4.695
4.666
4.513
4.483
4.201
4.189
4.172
4.160
4.154
4.143
4.036
4.013
3.978
3.971
3.957
3.947
3.940
3.873
3.853
3.844
3.827
3.818
3.810
3.793
3.767
3.744
3.396
2.110
2.076
2.064
1.815
1.331
0.827
0.056
0.025
AcO
AcO
TBSO
O
BocHN
O
OAc
O
BnO
AcO
OMe
21 (CDCl3, 400 MHz)
NMR
Solvent Peak
2.01
3.01
1.00
1.96
1.03
1.99
1.00
1.03
1.99
1.03
1.98
3.04
1.09
3.00
3.07
3.03
3.02
3.01
9.01
9.08
3.06
3.00
f1 (ppm)

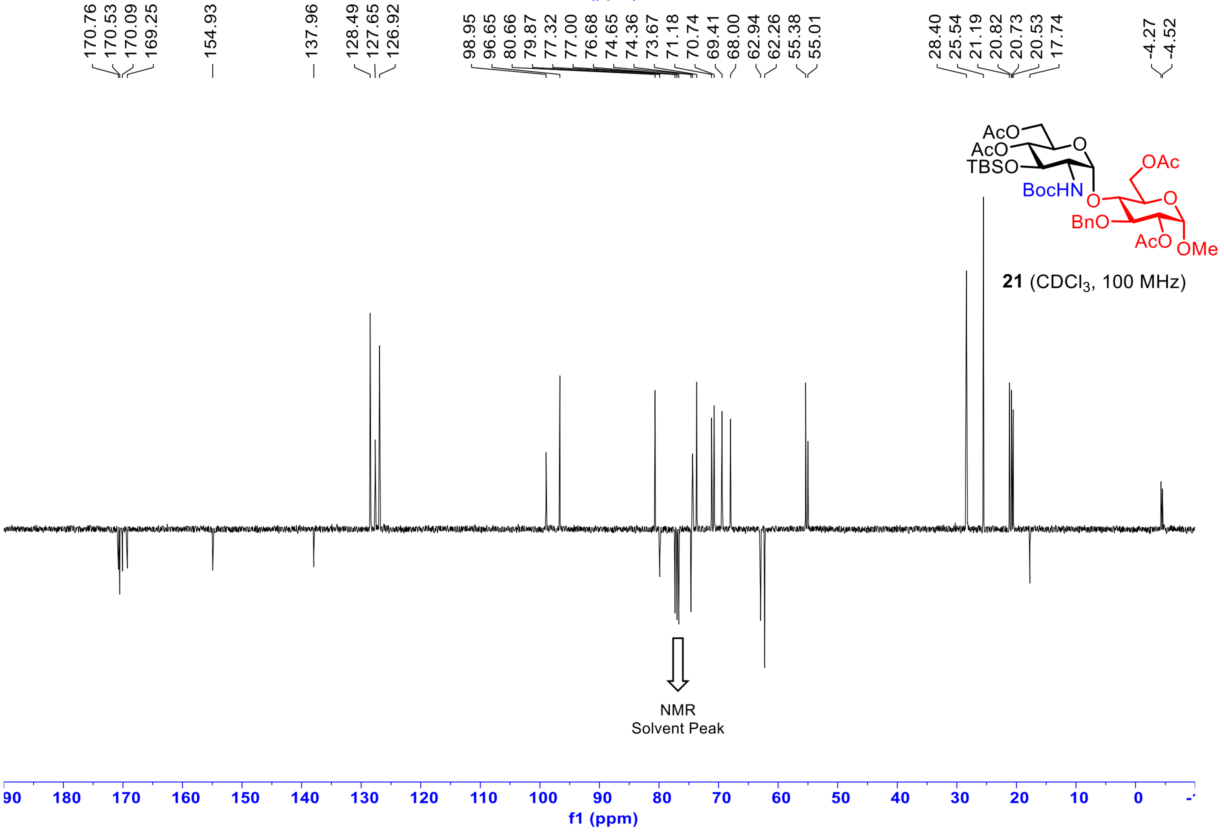
170.76
170.53
170.09
169.25
154.93
137.96
128.49
127.65
126.92
98.95
96.65
80.66
79.87
77.32
77.00
76.68
74.65
74.36
73.67
71.18
70.74
69.41
68.00
62.94
62.26
55.38
55.01
28.40
25.54
21.19
20.82
20.73
20.53
17.74
-4.27
-4.52
AcO
AcO
TBSO
O
BocHN
O
OAc
O
BnO
AcO
OMe
21 (CDCl3, 100 MHz)
NMR
Solvent Peak
f1 (ppm)

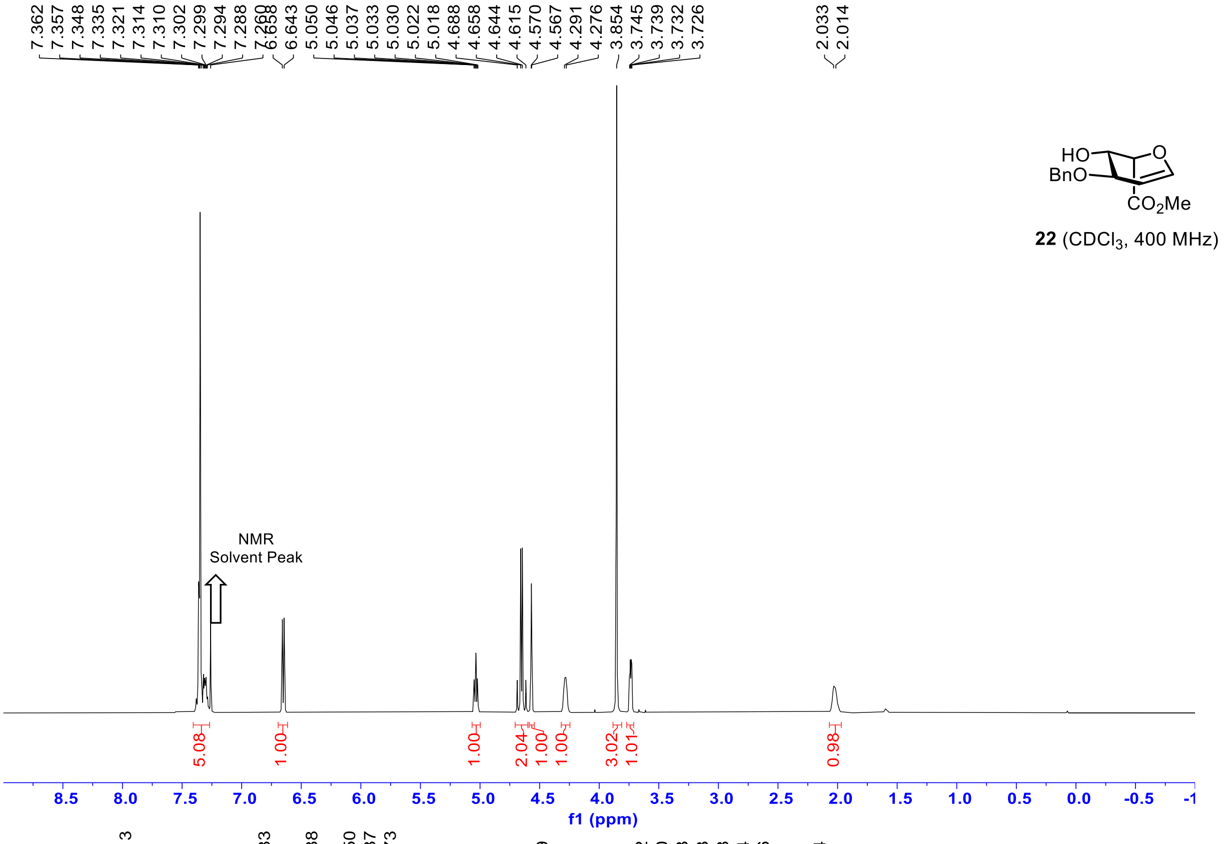
7.362
7.357
7.348
7.335
7.321
7.314
7.310
7.302
7.299
7.294
7.288
7.260
6.658
6.643
5.050
5.046
5.037
5.033
5.030
5.022
5.018
4.688
4.658
4.644
4.615
4.570
4.567
4.291
4.276
3.854
3.745
3.739
3.732
3.726
2.033
2.014
HO
BnO
O
CO2Me
22 (CDCl3, 400 MHz)
NMR
Solvent Peak
5.08
1.00
1.00
2.04
1.00
1.00
3.02
1.01
0.98
f1 (ppm)

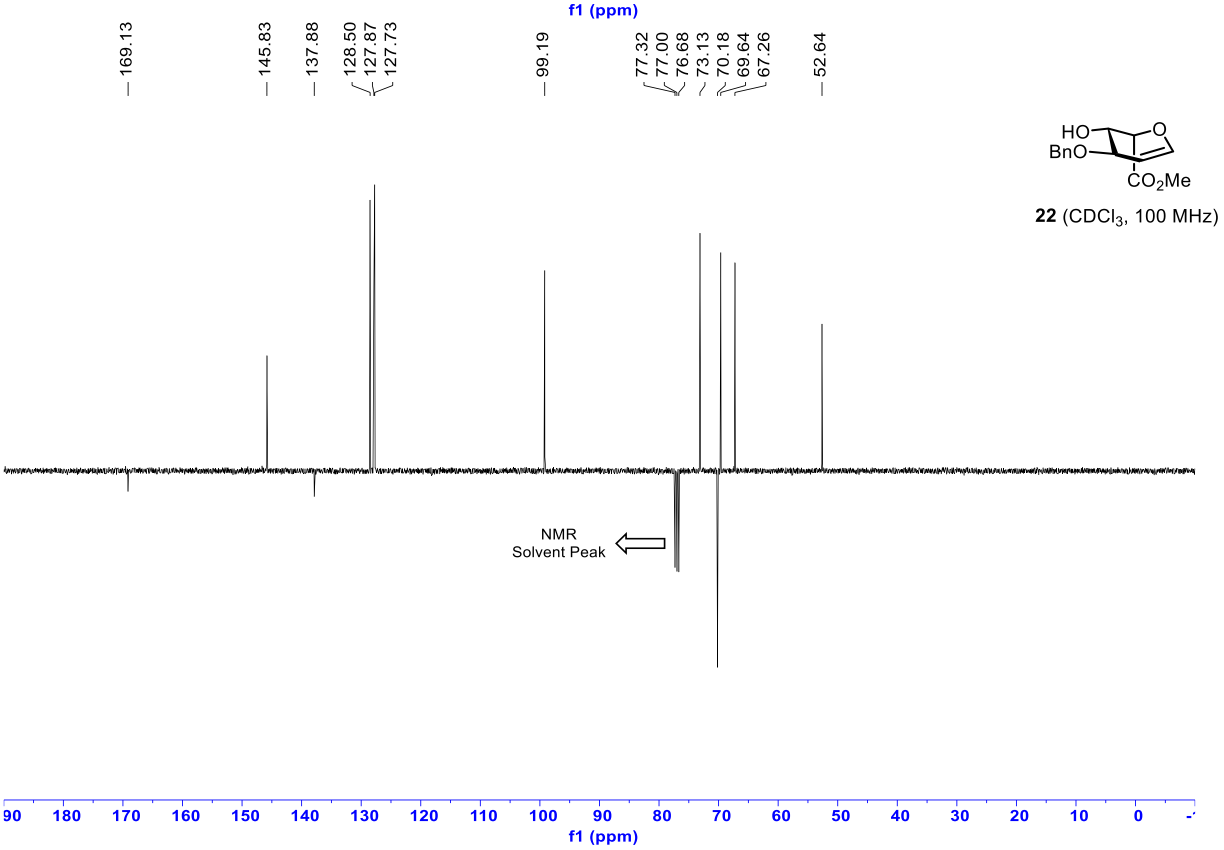
169.13
145.83
137.88
128.50
127.87
127.73
99.19
77.32
77.00
76.68
73.13
70.18
69.64
67.26
52.64
HO
BnO
O
CO2Me
22 (CDCl3, 100 MHz)
NMR
Solvent Peak
f1 (ppm)

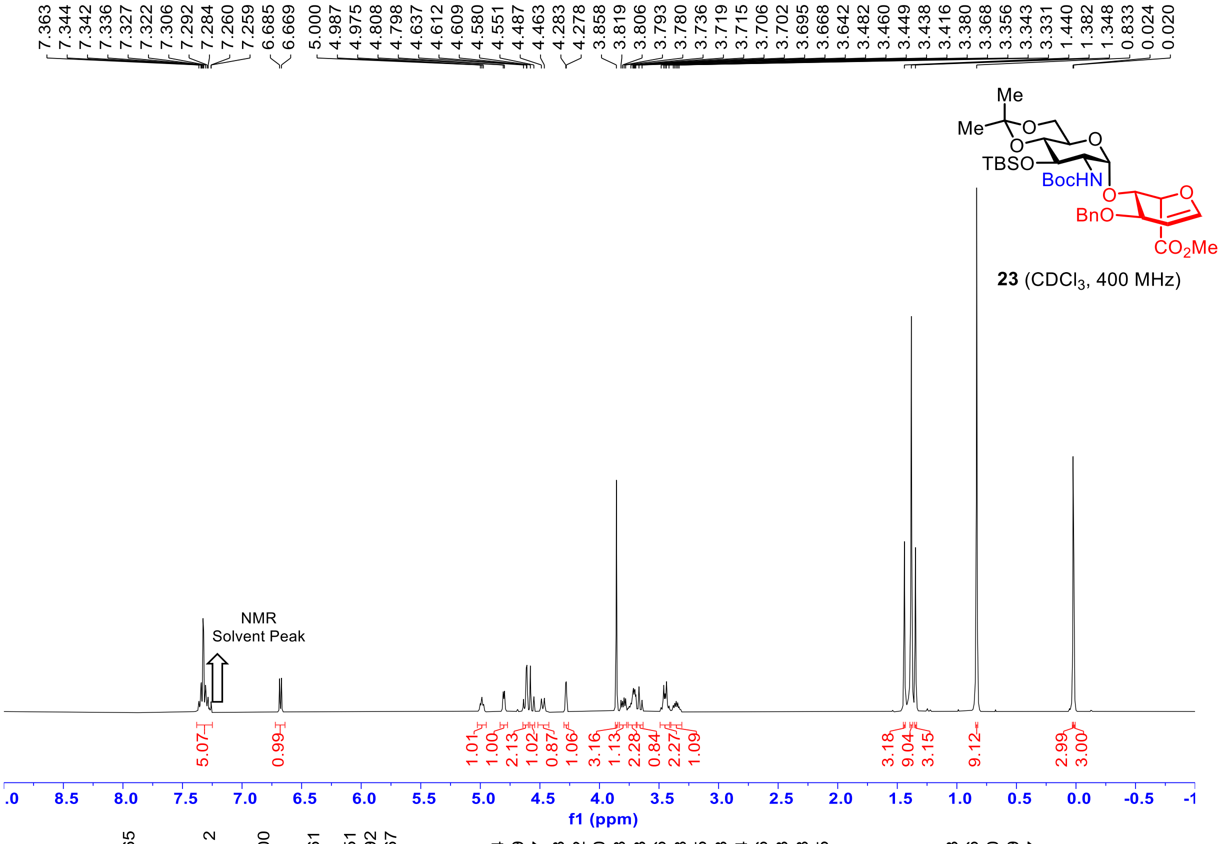
7.363
7.344
7.342
7.336
7.327
7.322
7.306
7.292
7.284
7.260
7.259
6.685
6.669
5.000
4.987
4.975
4.808
4.798
4.637
4.612
4.609
4.580
4.551
4.487
4.463
4.283
4.278
3.858
3.819
3.806
3.793
3.780
3.736
3.719
3.715
3.706
3.702
3.695
3.668
3.642
3.482
3.460
3.449
3.438
3.416
3.380
3.368
3.356
3.343
3.331
1.440
1.382
1.348
0.833
0.024
0.020
Me
Me
O
O
TBSO
O
BocHN
O
BnO
O
CO_2Me
23 ($CDCl_3$, 400 MHz)
NMR Solvent Peak
5.07
0.99
1.01
1.00
2.13
1.02
0.87
1.06
3.16
1.13
2.28
0.84
2.27
1.09
3.18
9.04
3.15
9.12
2.99
3.00
f1 (ppm)

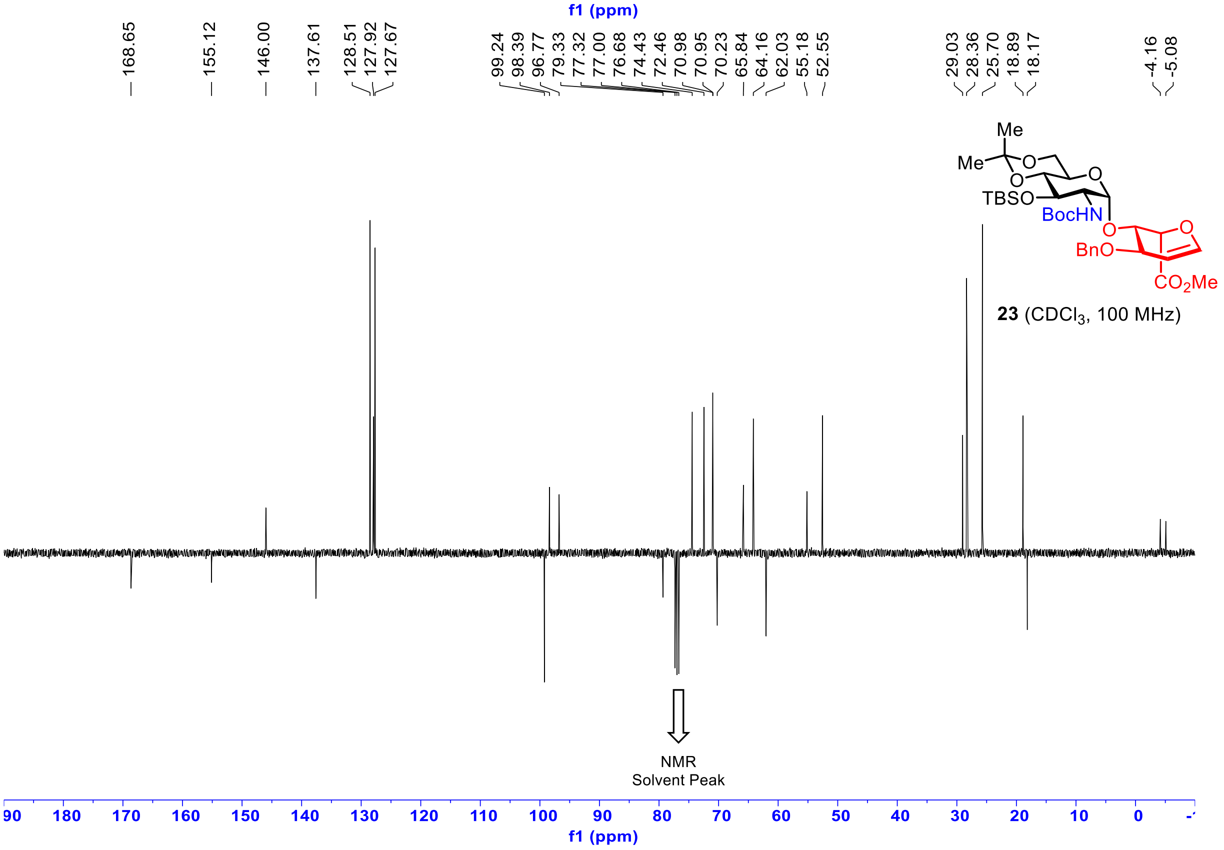
168.65
155.12
146.00
137.61
128.51
127.92
127.67
99.24
98.39
96.77
79.33
77.32
77.00
76.68
74.43
72.46
70.98
70.95
70.23
65.84
64.16
62.03
55.18
52.55
29.03
28.36
25.70
18.89
18.17
-4.16
-5.08
Me
Me
O
O
TBSO
O
BocHN
O
BnO
O
CO_2Me
23 ($CDCl_3$, 100 MHz)
NMR Solvent Peak
f1 (ppm)

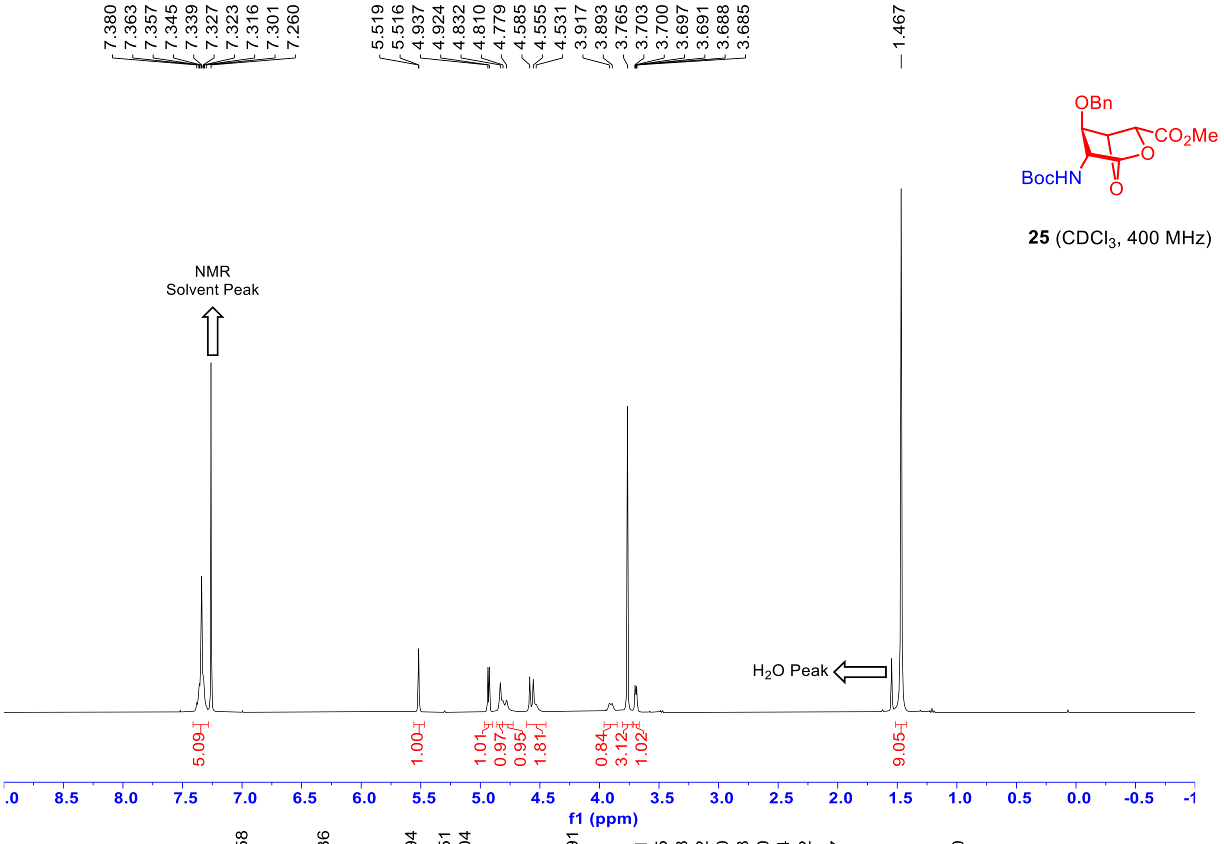

25 ($CDCl_3$, 400 MHz)

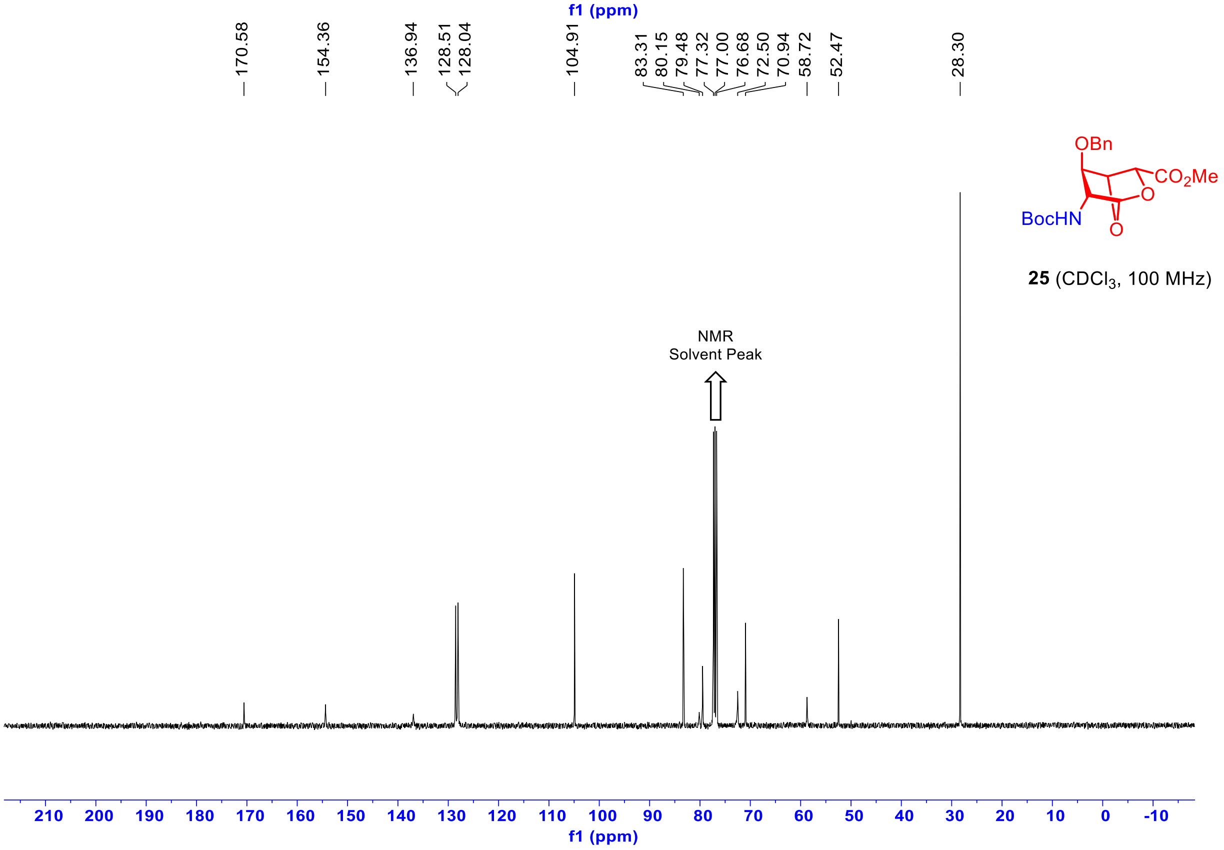

25 ($CDCl_3$, 100 MHz)

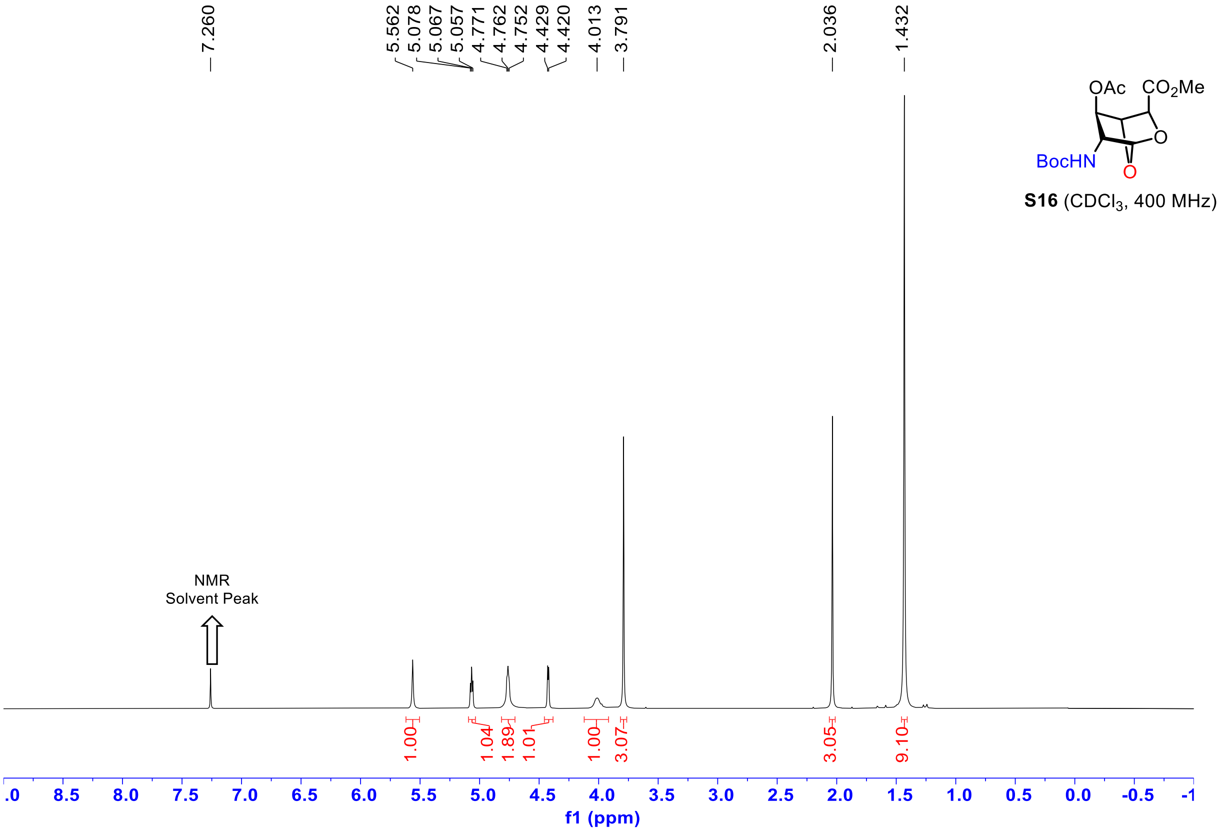

S16 ($CDCl_3$, 400 MHz)

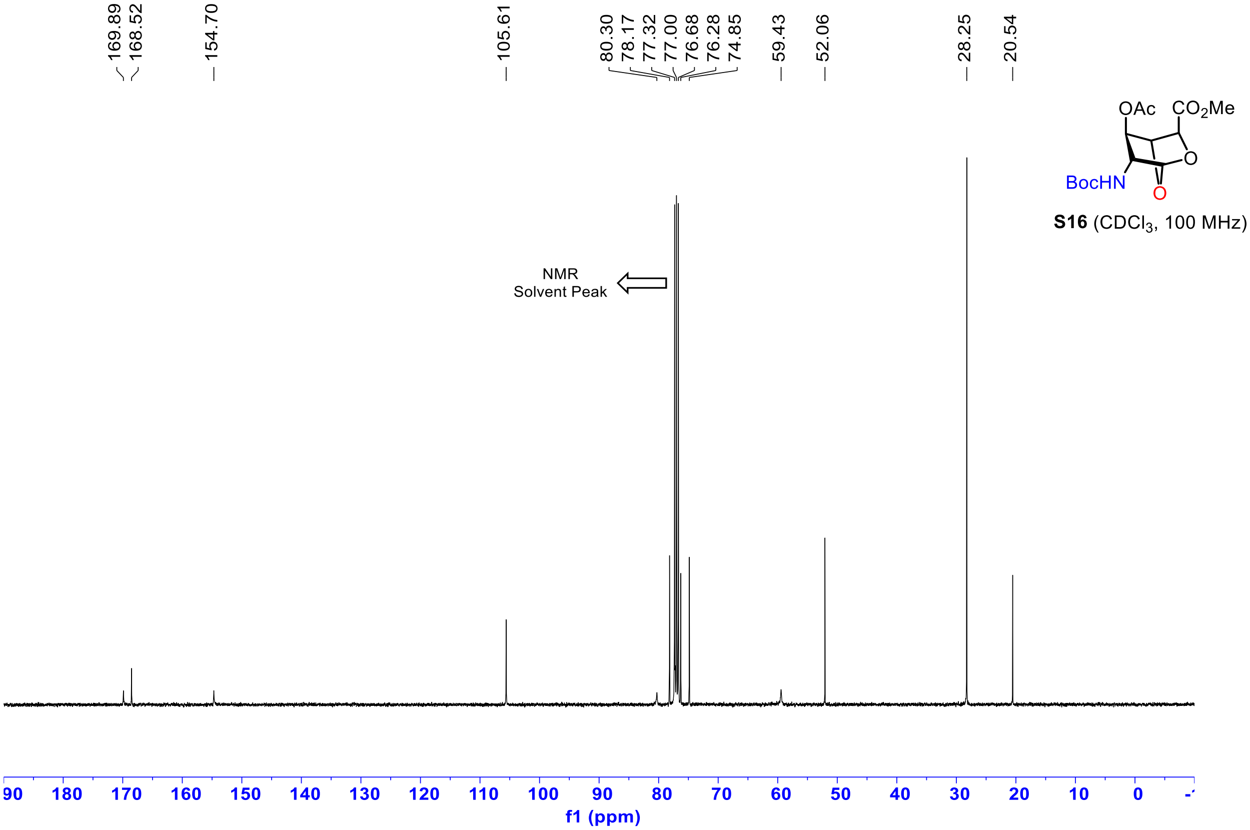

S16 ($CDCl_3$, 100 MHz)

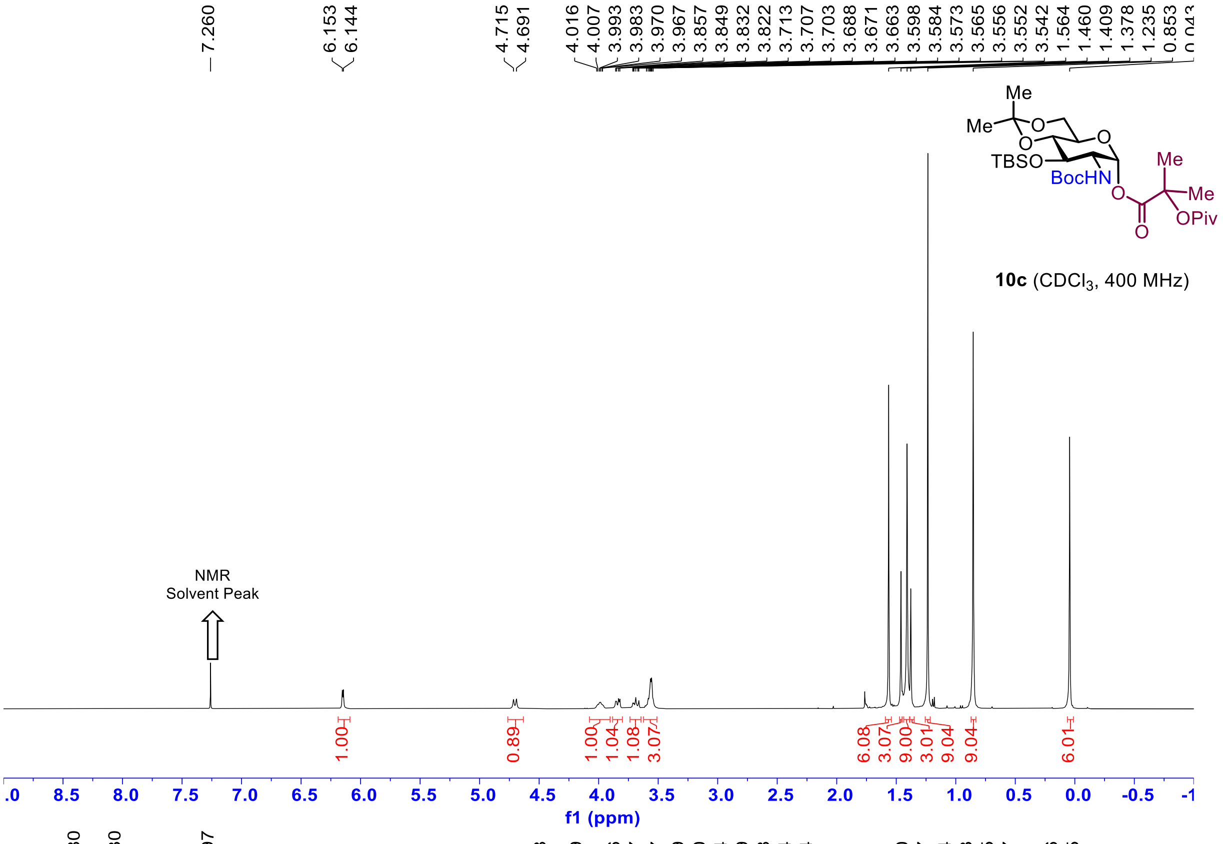

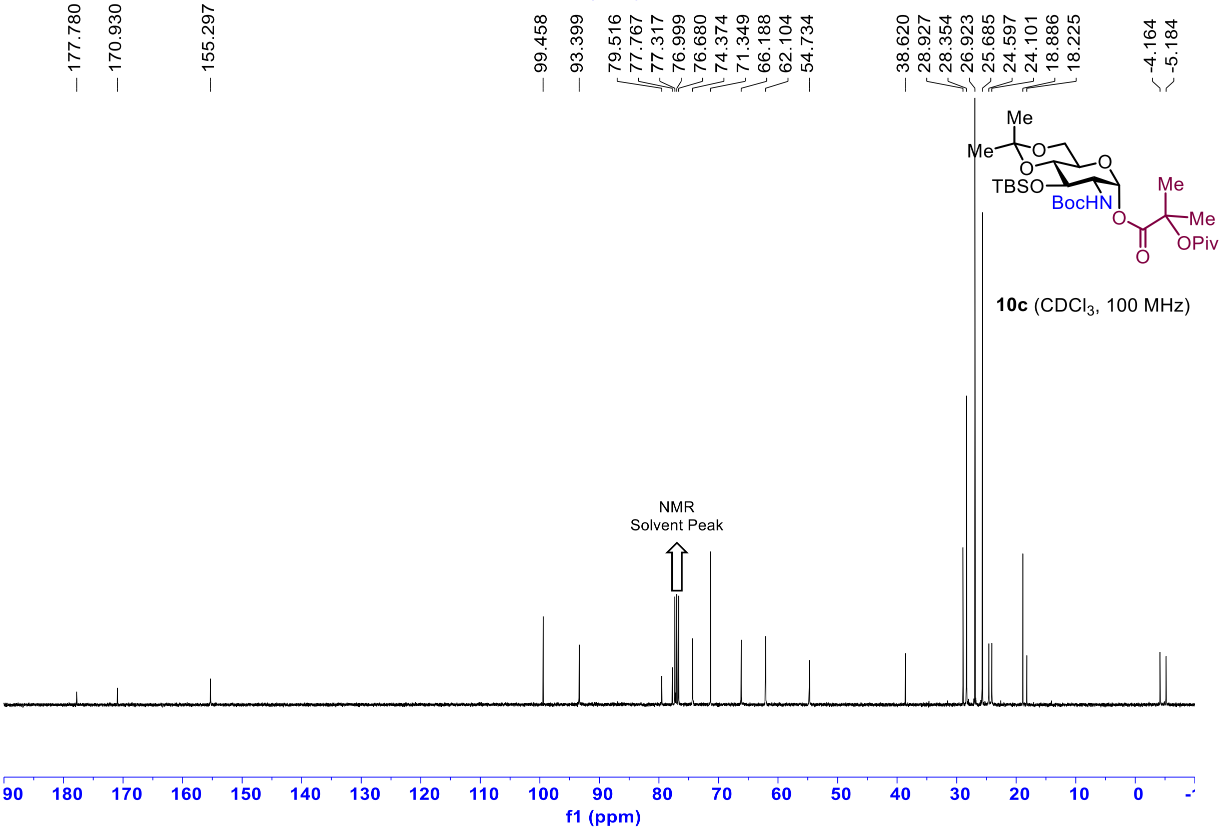

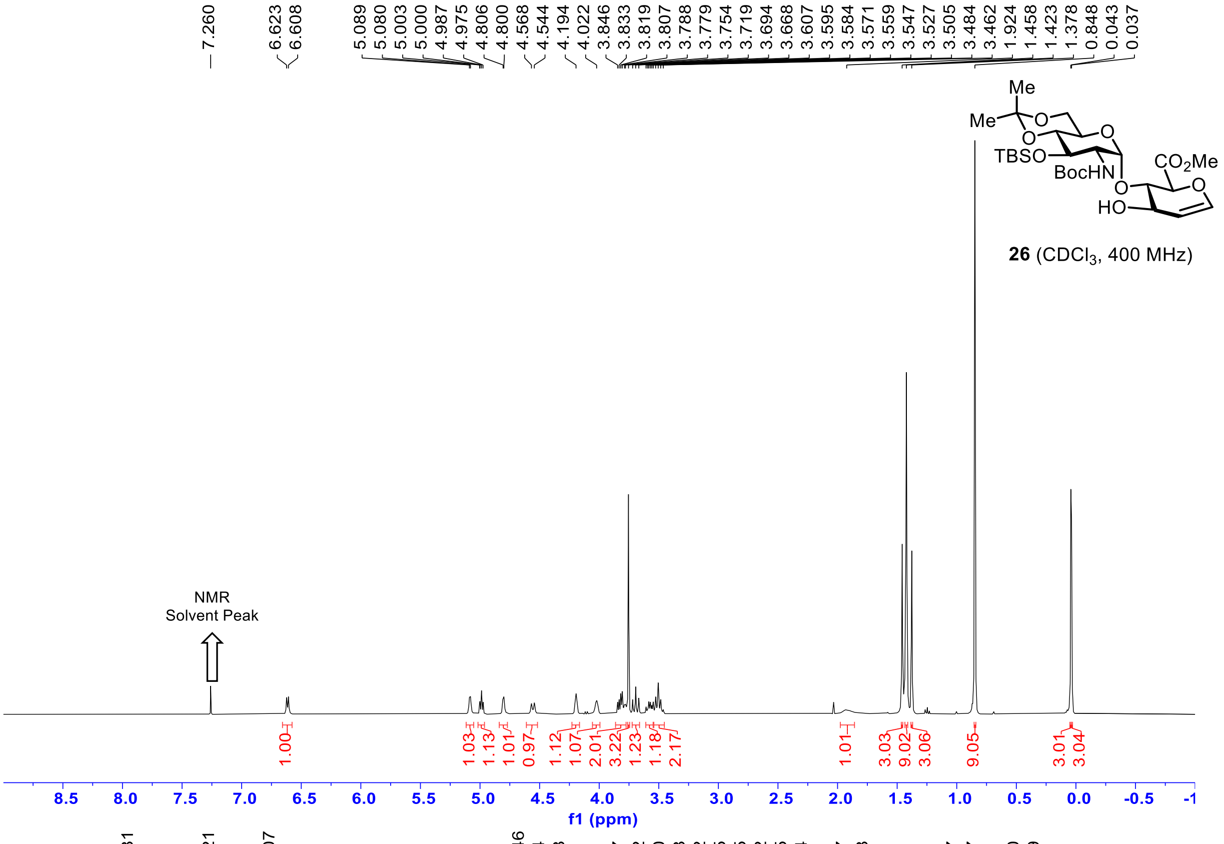
7.260
6.623
6.608
5.089
5.080
5.003
5.000
4.987
4.975
4.806
4.800
4.568
4.544
4.194
4.022
3.846
3.833
3.819
3.807
3.788
3.779
3.754
3.719
3.694
3.668
3.607
3.595
3.584
3.571
3.559
3.547
3.527
3.505
3.484
3.462
1.924
1.458
1.423
1.378
0.848
0.043
0.037
Me
Me
O
O
O
TBSO
BocHN
O
CO2Me
O
HO
26 (CDCl3, 400 MHz)
NMR
Solvent Peak
1.00
1.03
1.13
1.01
0.97
1.12
1.07
2.01
3.22
1.23
1.18
2.17
1.01
3.03
9.02
3.06
9.05
3.01
3.04
8.5
8.0
7.5
7.0
6.5
6.0
5.5
5.0
4.5
4.0
3.5
3.0
2.5
2.0
1.5
1.0
0.5
0.0
-0.5
f1 (ppm)

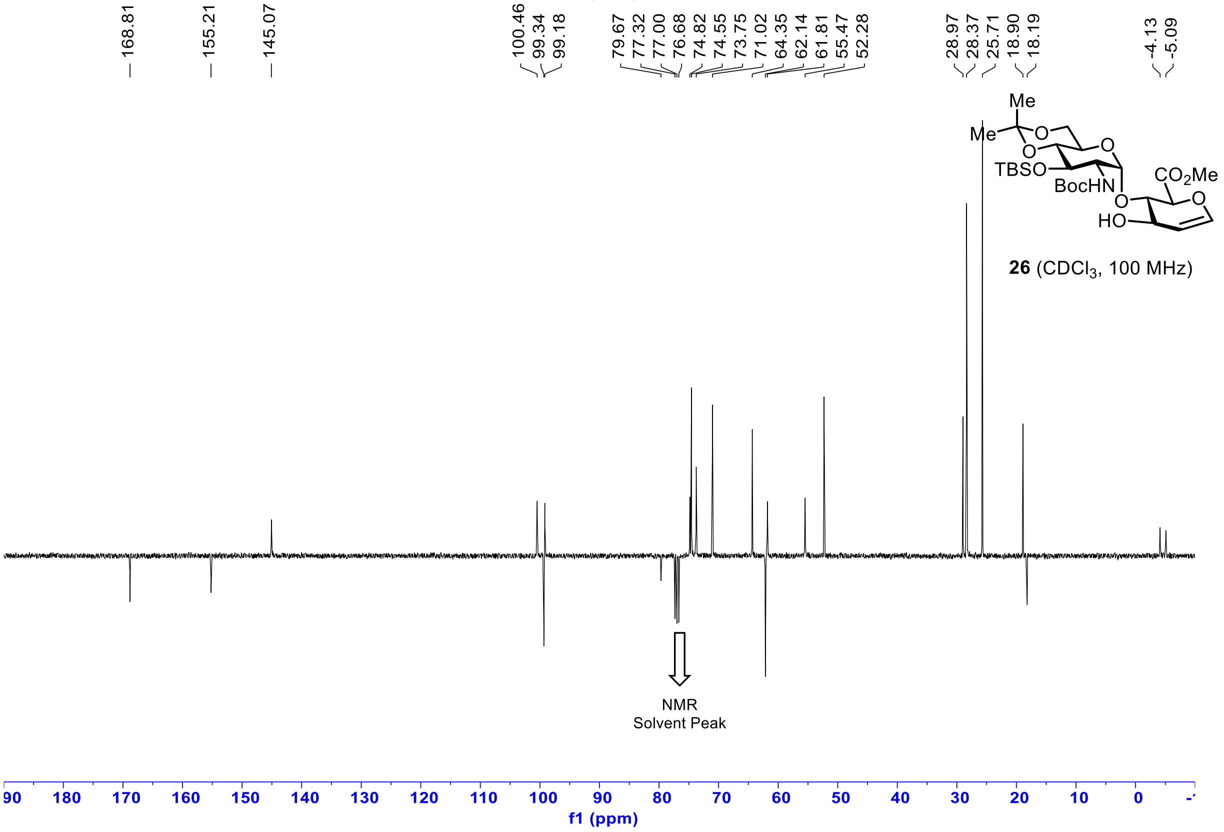
168.81
155.21
145.07
100.46
99.34
99.18
79.67
77.32
77.00
76.68
74.82
74.55
73.75
71.02
64.35
62.14
61.81
55.47
52.28
28.97
28.37
25.71
18.90
18.19
-4.13
-5.09
Me
Me
O
O
O
TBSO
BocHN
O
CO2Me
O
HO
26 (CDCl3, 100 MHz)
NMR
Solvent Peak
180
170
160
150
140
130
120
110
100
90
80
70
60
50
40
30
20
10
0
f1 (ppm)

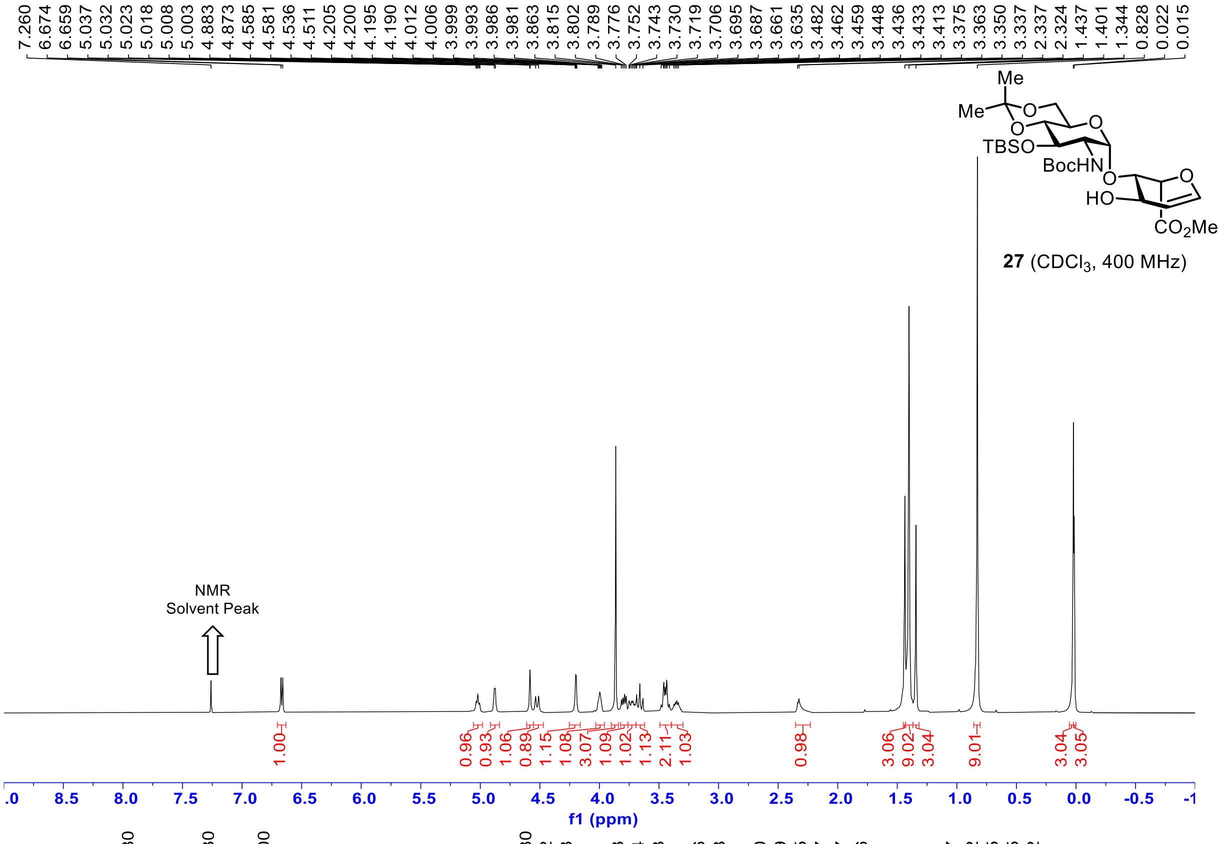
7.260 6.674 6.659 5.037 5.032 5.023 5.018 5.008 5.003 4.883 4.873 4.585 4.581 4.536 4.511 4.205 4.200 4.195 4.190 4.012 4.006 3.999 3.993 3.986 3.981 3.863 3.815 3.802 3.789 3.776 3.752 3.743 3.730 3.719 3.706 3.695 3.687 3.661 3.635 3.482 3.462 3.459 3.448 3.436 3.433 3.413 3.375 3.363 3.350 3.337 2.337 2.324 1.437 1.401 1.344 0.828 0.022 0.015
Me
Me
O
O
O
TBSO
BocHN
O
O
HO
CO_2Me
27 ($CDCl_3$, 400 MHz)
NMR
Solvent Peak
1.00 0.96 0.93 1.06 0.89 1.15 1.08 3.07 1.09 1.02 1.13 2.11 1.03 0.98 3.06 9.02 3.04 9.01 3.04 3.05
f1 (ppm)

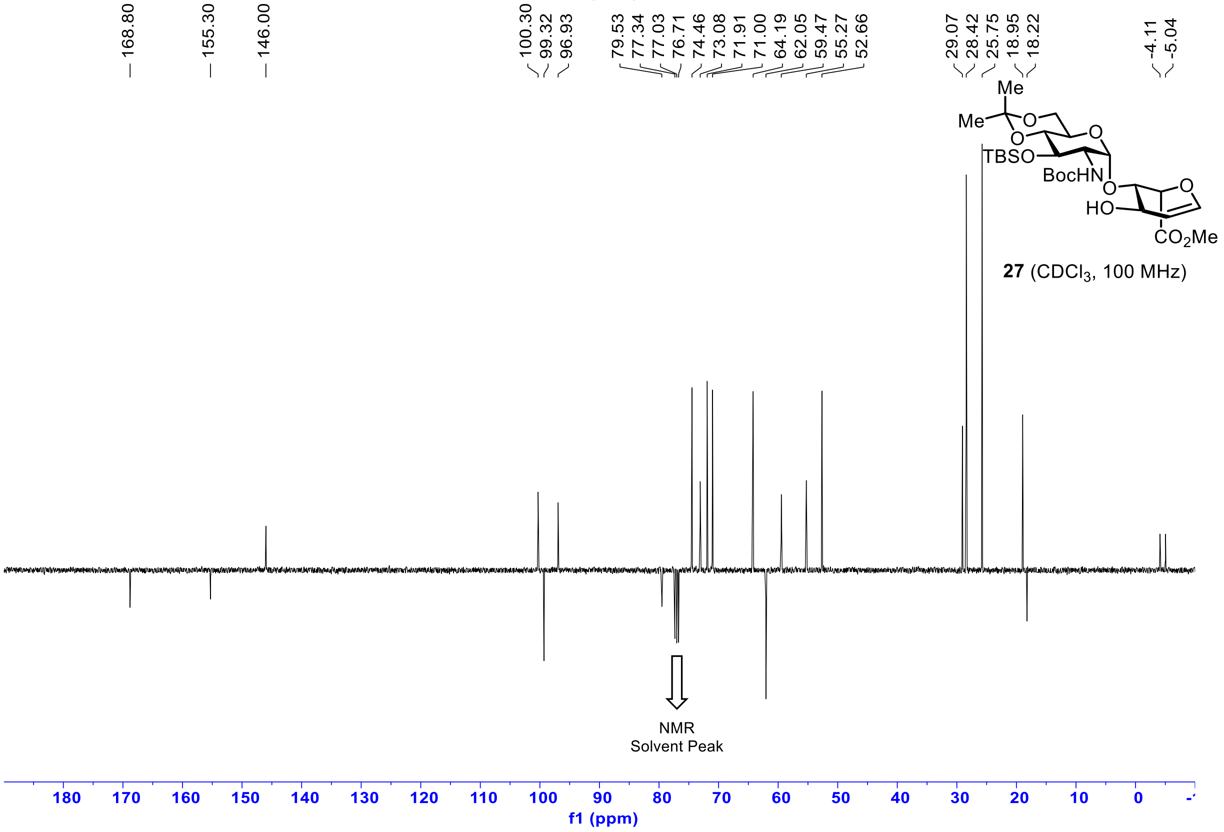
168.80 155.30 146.00 100.30 99.32 96.93 79.53 77.34 77.03 76.71 74.46 73.08 71.91 71.00 64.19 62.05 59.47 55.27 52.66 29.07 28.42 25.75 18.95 18.22 -4.11 -5.04
Me
Me
O
O
O
TBSO
BocHN
O
O
HO
CO_2Me
27 ($CDCl_3$, 100 MHz)
NMR
Solvent Peak
f1 (ppm)

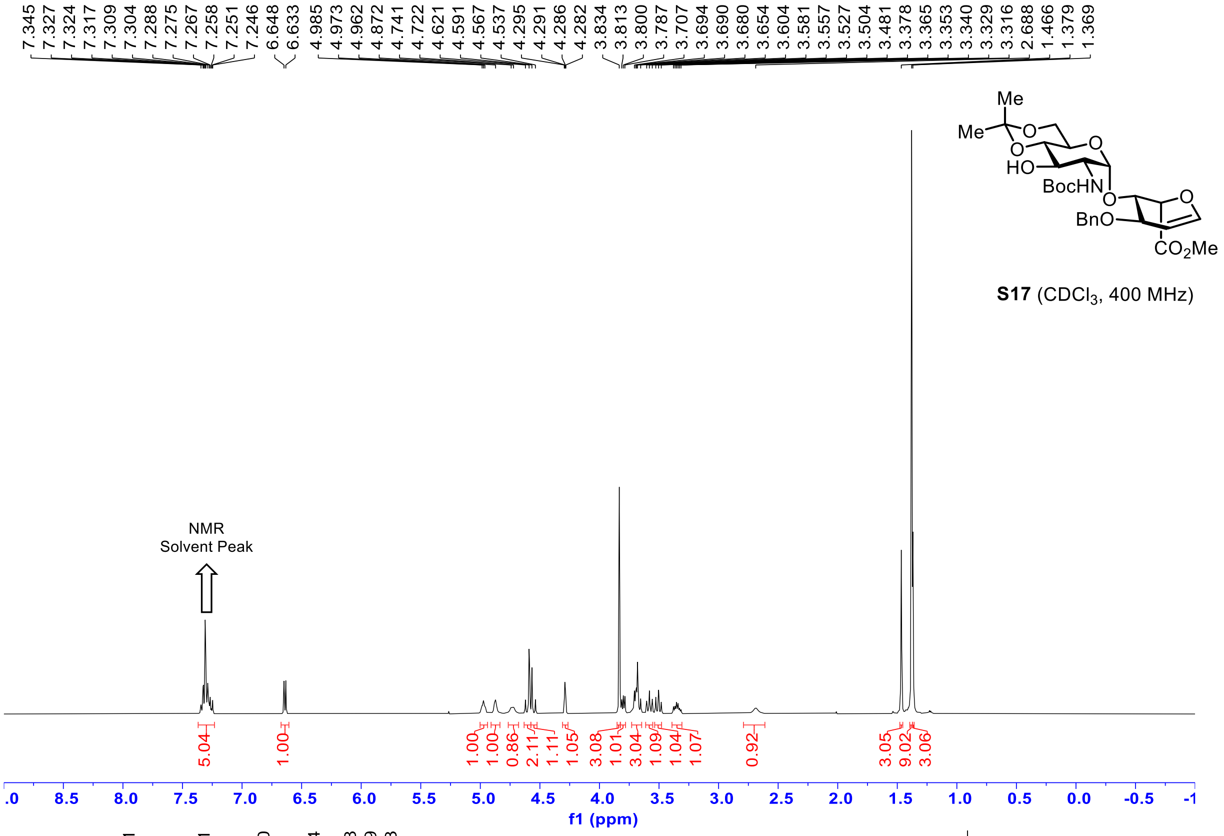
NMR
Solvent Peak
S17 (CDCl3, 400 MHz)
f1 (ppm)

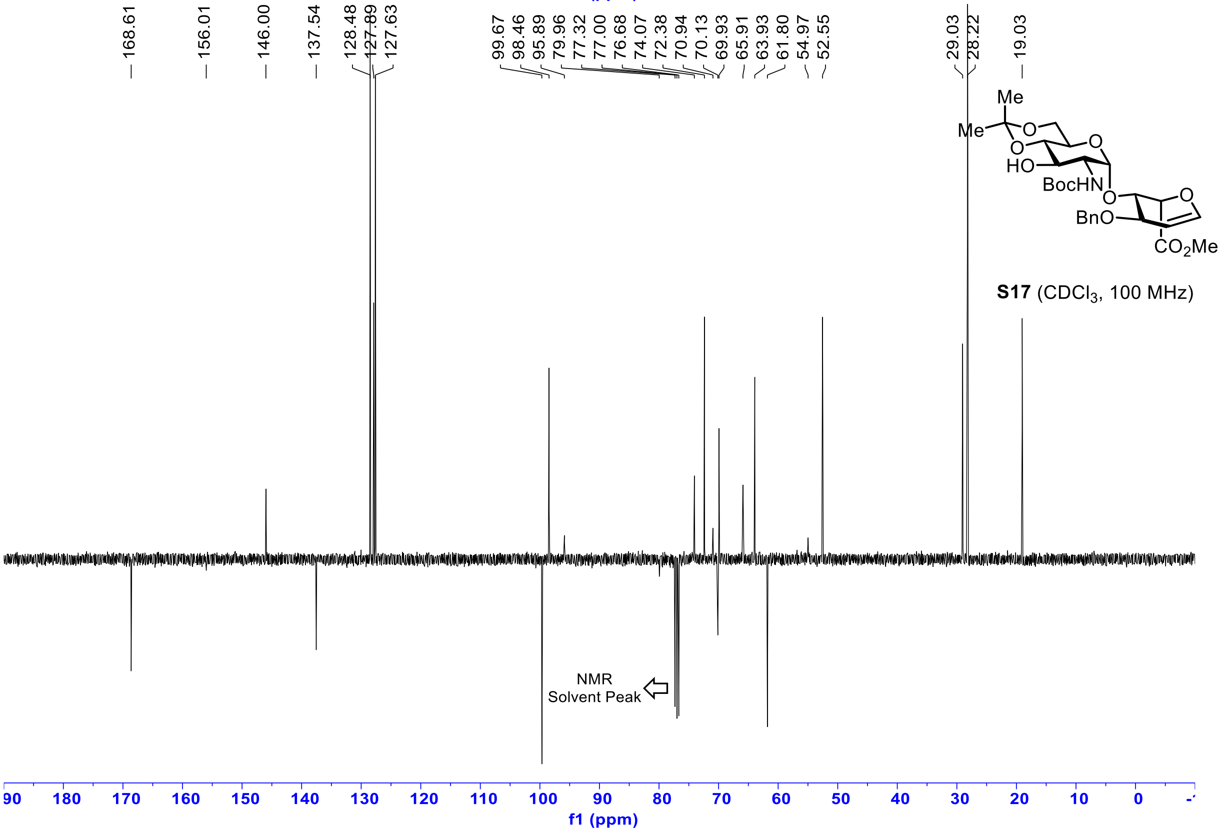
NMR
Solvent Peak
S17 (CDCl3, 100 MHz)
f1 (ppm)

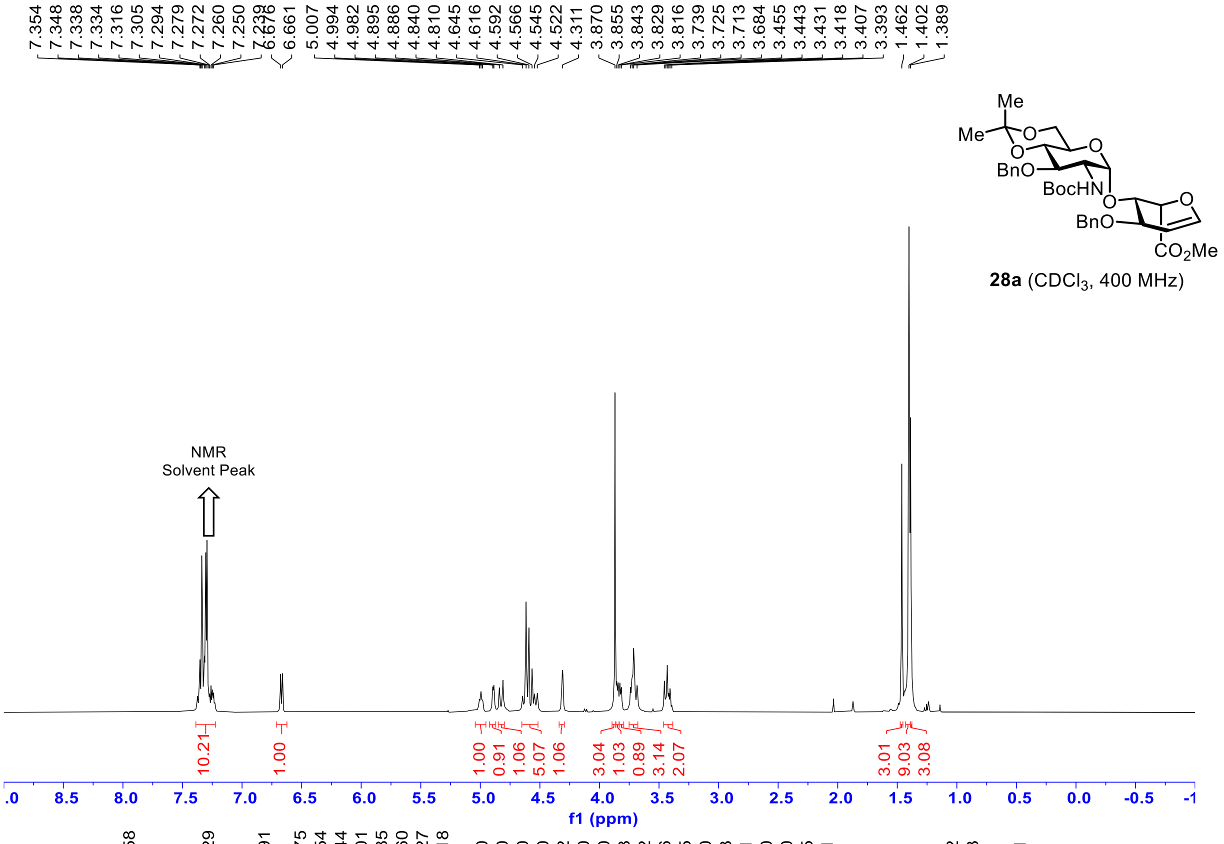

28a ($CDCl_3$, 400 MHz)

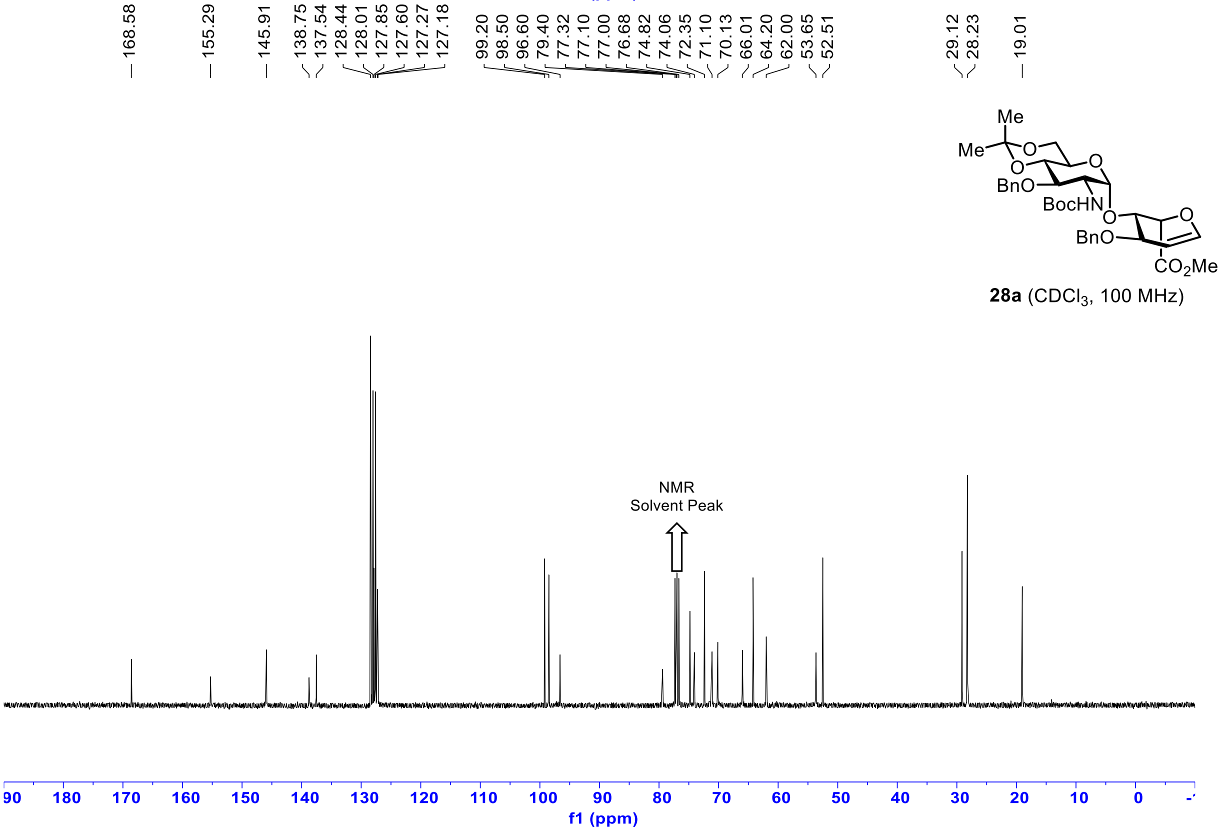

28a ($CDCl_3$, 100 MHz)

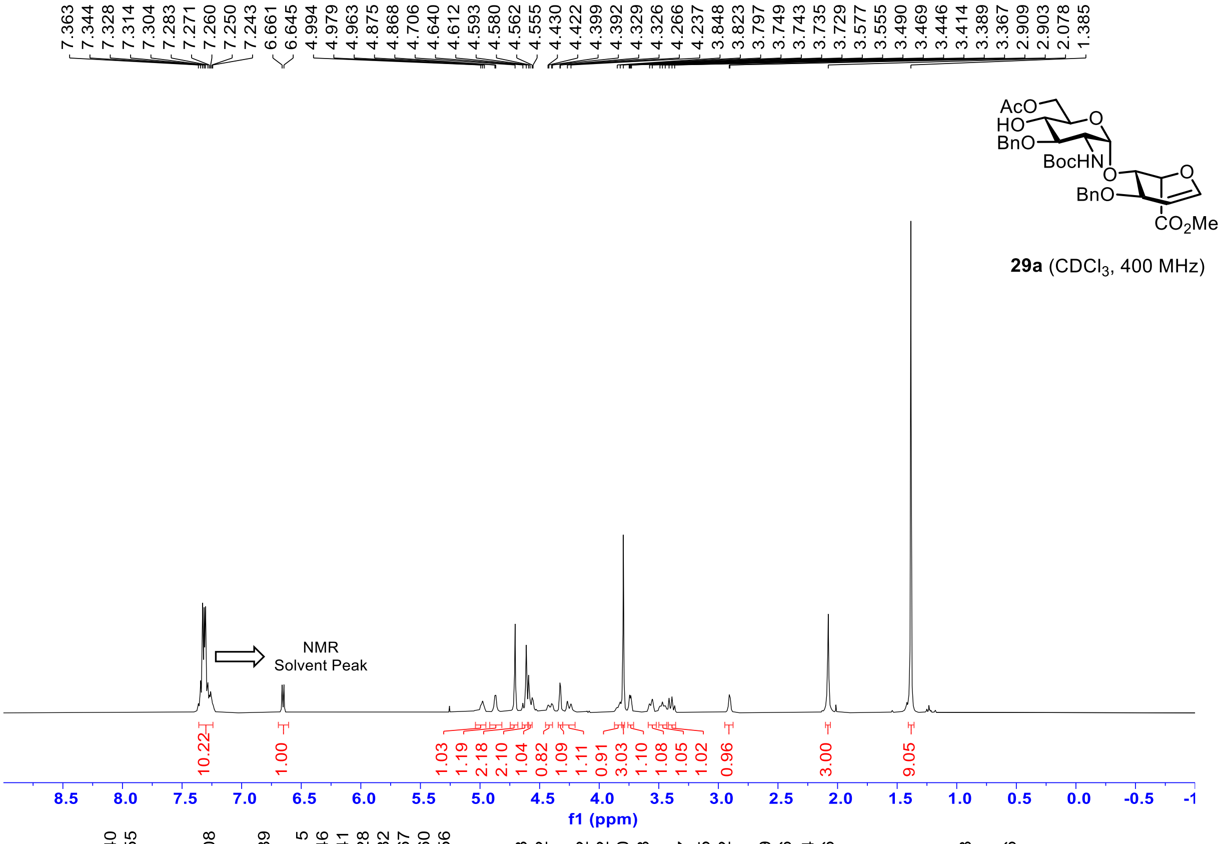
7.363
7.344
7.328
7.314
7.304
7.283
7.271
7.260
7.250
7.243
6.661
6.645
4.994
4.979
4.963
4.875
4.868
4.706
4.640
4.612
4.593
4.580
4.562
4.555
4.430
4.422
4.399
4.392
4.329
4.326
4.266
4.237
3.848
3.823
3.797
3.749
3.743
3.735
3.729
3.577
3.555
3.490
3.469
3.446
3.414
3.389
3.367
2.909
2.903
2.078
1.385
AcO
HO
BnO
O
BocHN
O
O
BnO
CO_2Me
29a ($CDCl_3$, 400 MHz)
NMR
Solvent Peak
10.22
1.00
1.03
1.19
2.18
2.10
1.04
0.82
1.09
1.11
0.91
3.03
1.10
1.08
1.05
1.02
0.96
3.00
9.05
8.5
8.0
7.5
7.0
6.5
6.0
5.5
5.0
4.5
4.0
3.5
3.0
2.5
2.0
1.5
1.0
0.5
0.0
-0.5
f1 (ppm)

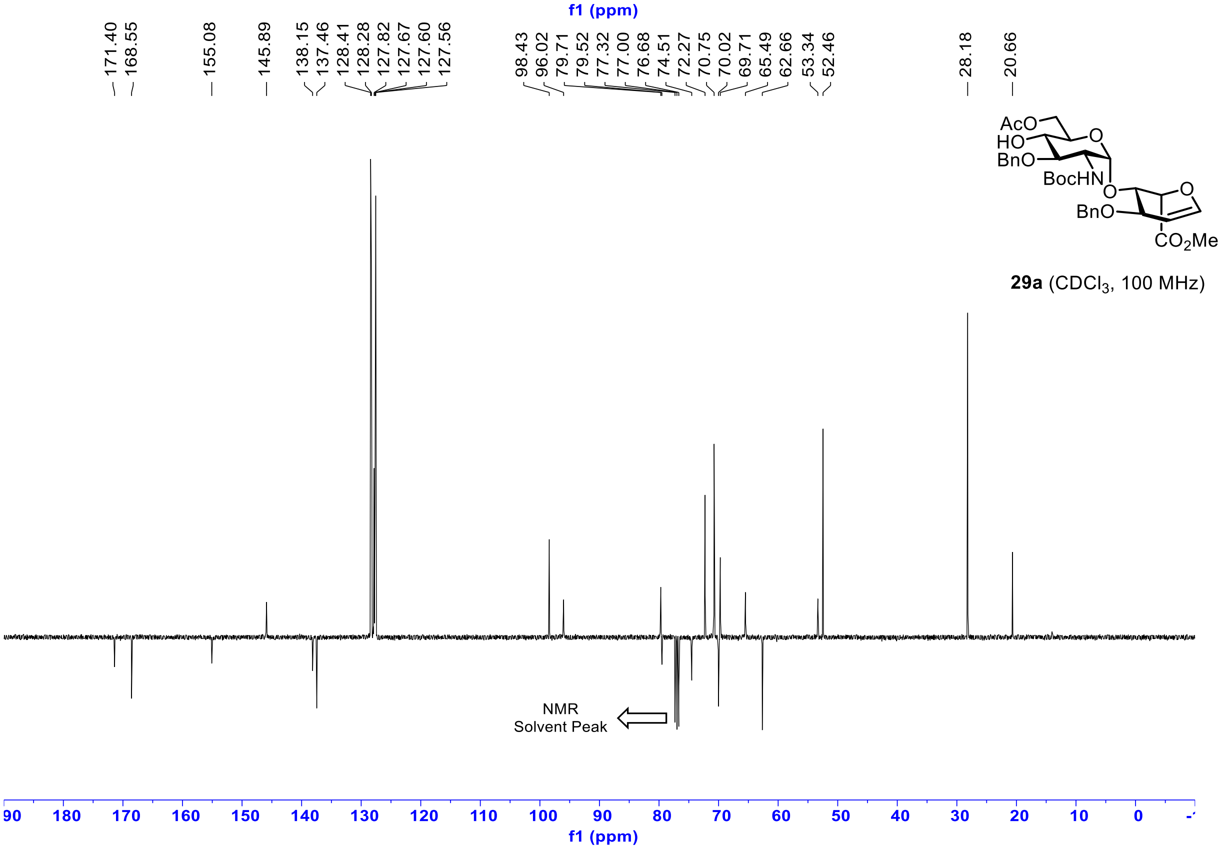
171.40
168.55
155.08
145.89
138.15
137.46
128.41
128.28
127.82
127.67
127.60
127.56
98.43
96.02
79.71
79.52
77.32
77.00
76.68
74.51
72.27
70.75
70.02
69.71
65.49
62.66
53.34
52.46
28.18
20.66
AcO
HO
BnO
O
BocHN
O
O
BnO
CO_2Me
29a ($CDCl_3$, 100 MHz)
NMR
Solvent Peak
180
170
160
150
140
130
120
110
100
90
80
70
60
50
40
30
20
10
0
f1 (ppm)

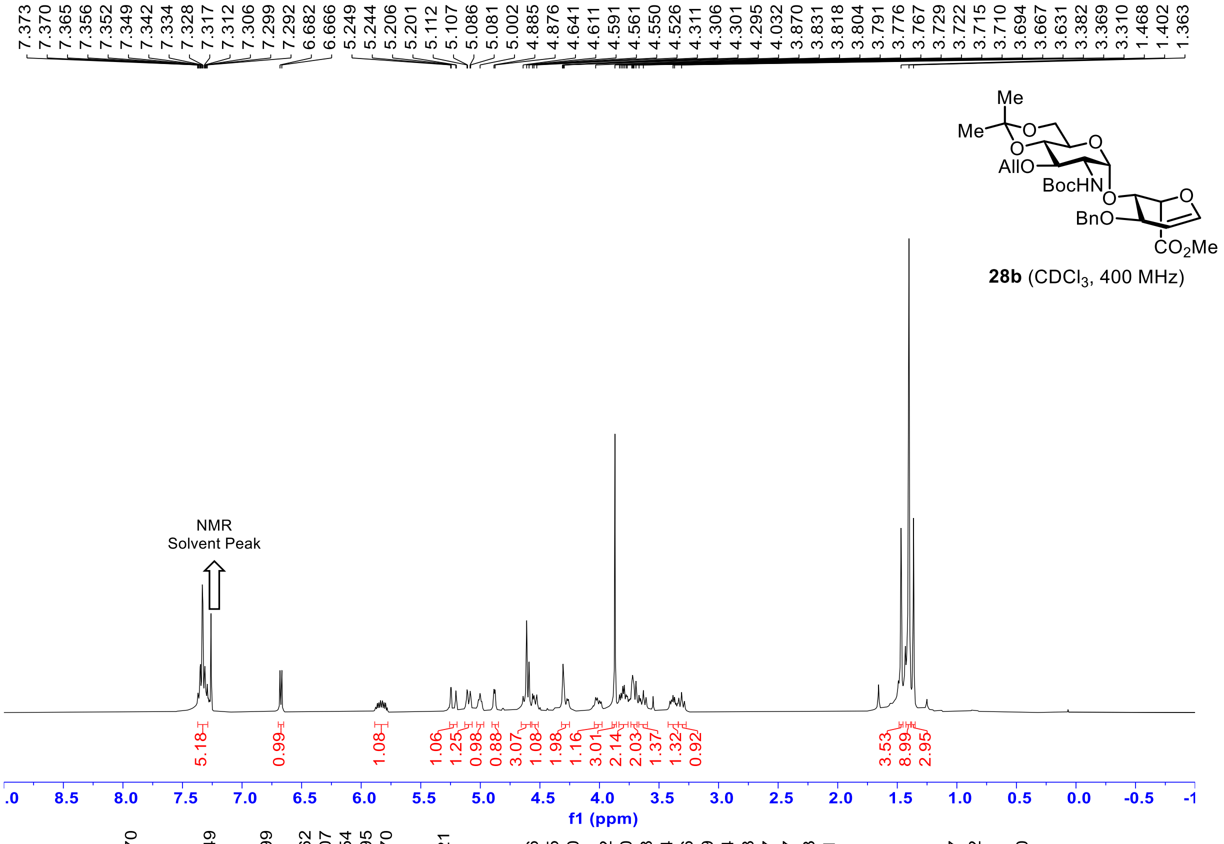
7.373
7.370
7.365
7.356
7.352
7.349
7.342
7.334
7.328
7.317
7.312
7.306
7.299
7.292
6.682
6.666
5.249
5.244
5.206
5.201
5.112
5.107
5.086
5.081
5.002
4.885
4.876
4.641
4.611
4.591
4.561
4.550
4.526
4.311
4.306
4.301
4.295
4.032
3.870
3.831
3.818
3.804
3.791
3.776
3.767
3.729
3.722
3.715
3.710
3.694
3.667
3.631
3.382
3.369
3.310
1.468
1.402
1.363
Me
Me
O
O
O
AllO
BocHN
O
O
BnO
CO2Me
28b (CDCl3, 400 MHz)
NMR
Solvent Peak
5.18
0.99
1.08
1.06
1.25
0.98
0.88
3.07
1.08
1.98
1.16
3.01
2.14
2.03
1.37
1.32
0.92
3.53
8.99
2.95
f1 (ppm)

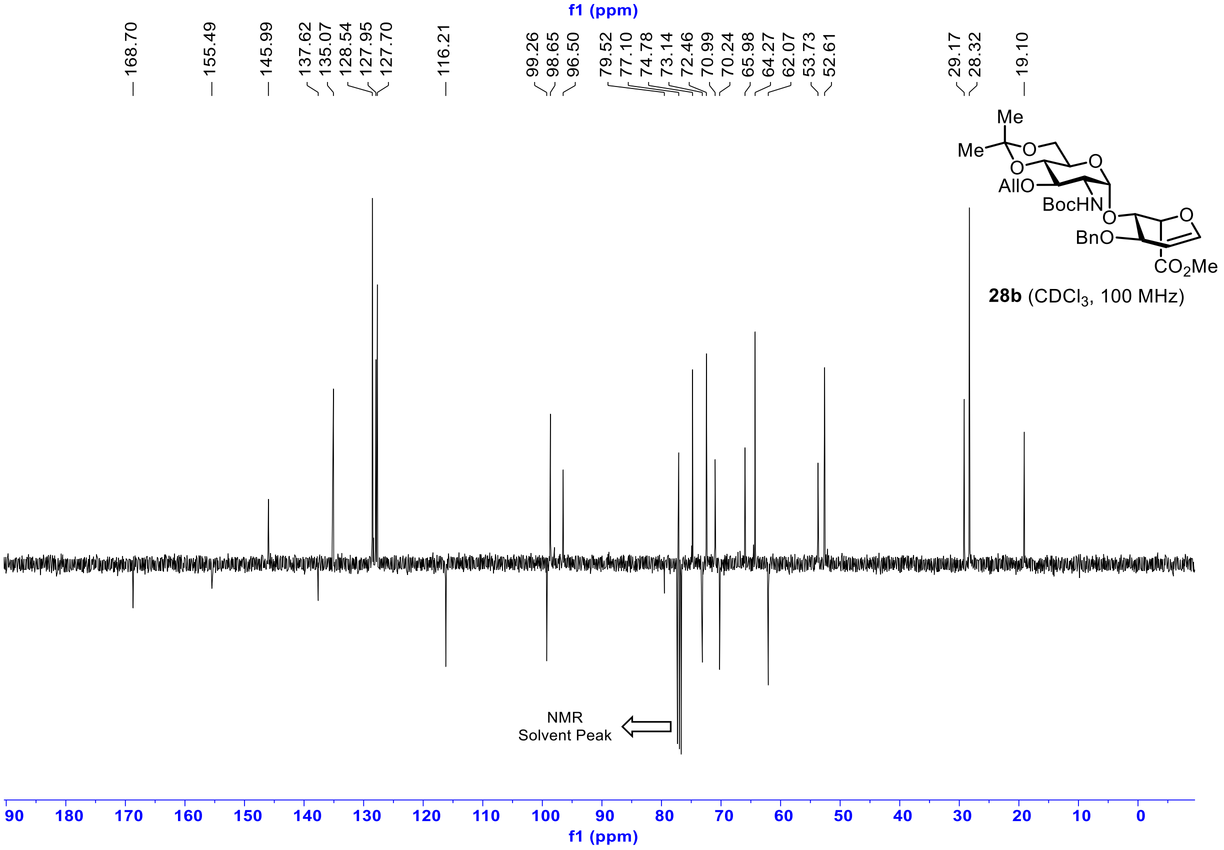
168.70
155.49
145.99
137.62
135.07
128.54
127.95
127.70
116.21
99.26
98.65
96.50
79.52
77.10
74.78
73.14
72.46
70.99
70.24
65.98
64.27
62.07
53.73
52.61
29.17
28.32
19.10
Me
Me
O
O
O
AllO
BocHN
O
O
BnO
CO2Me
28b (CDCl3, 100 MHz)
NMR
Solvent Peak
f1 (ppm)

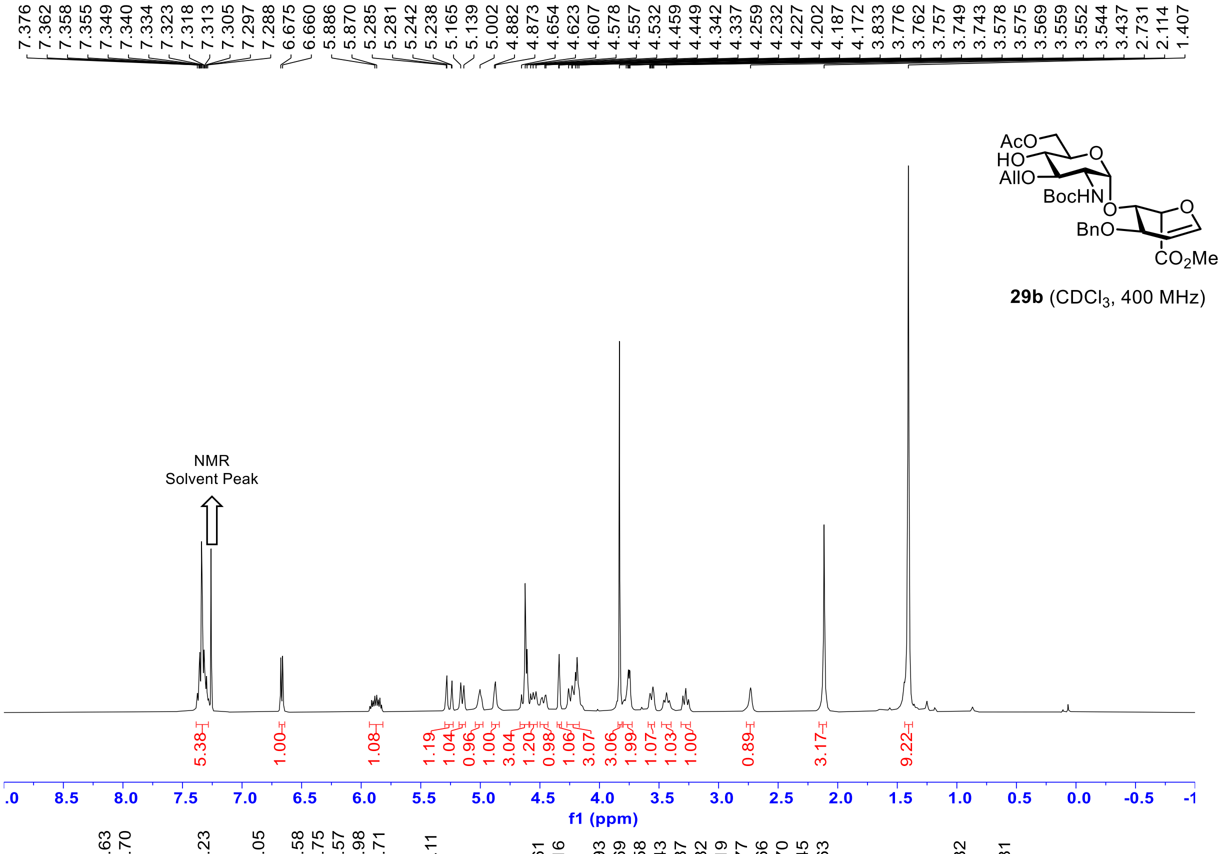

29b ($CDCl_3$, 400 MHz)

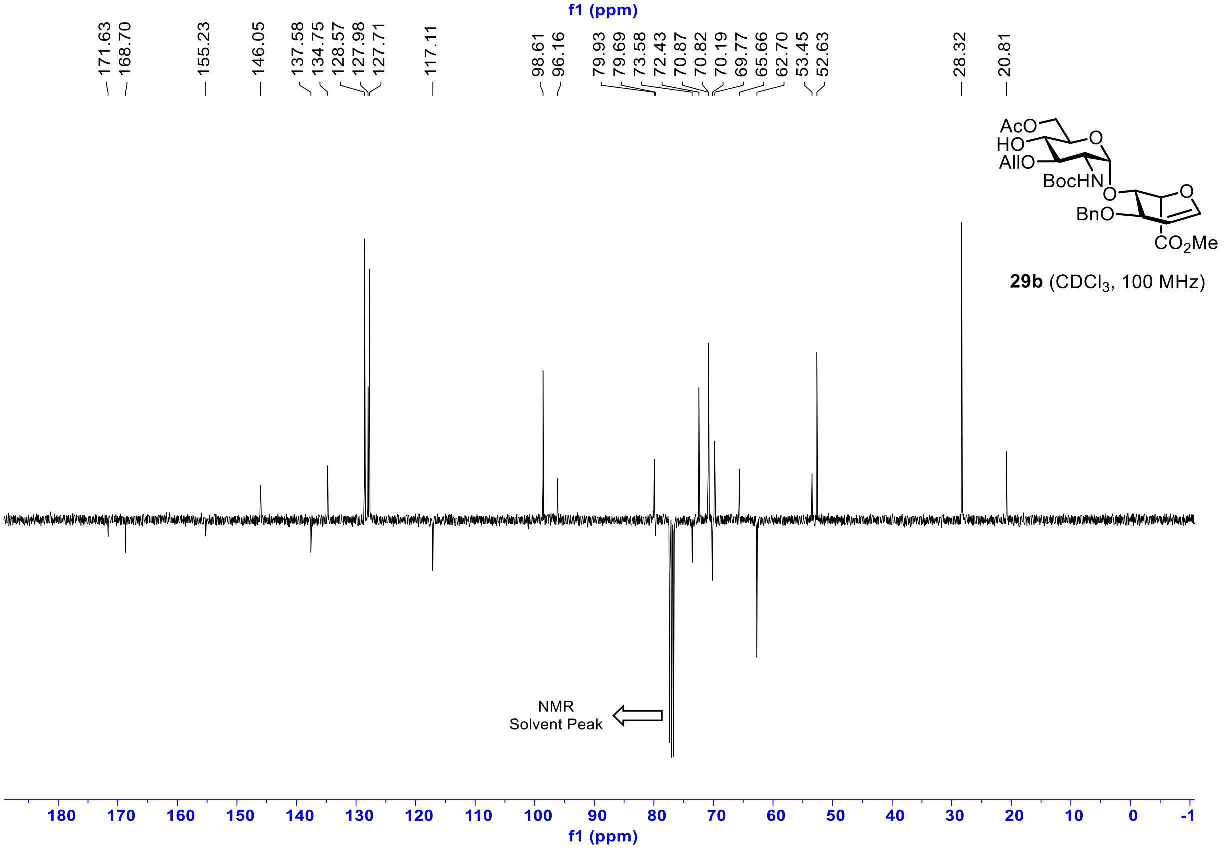

29b ($CDCl_3$, 100 MHz)

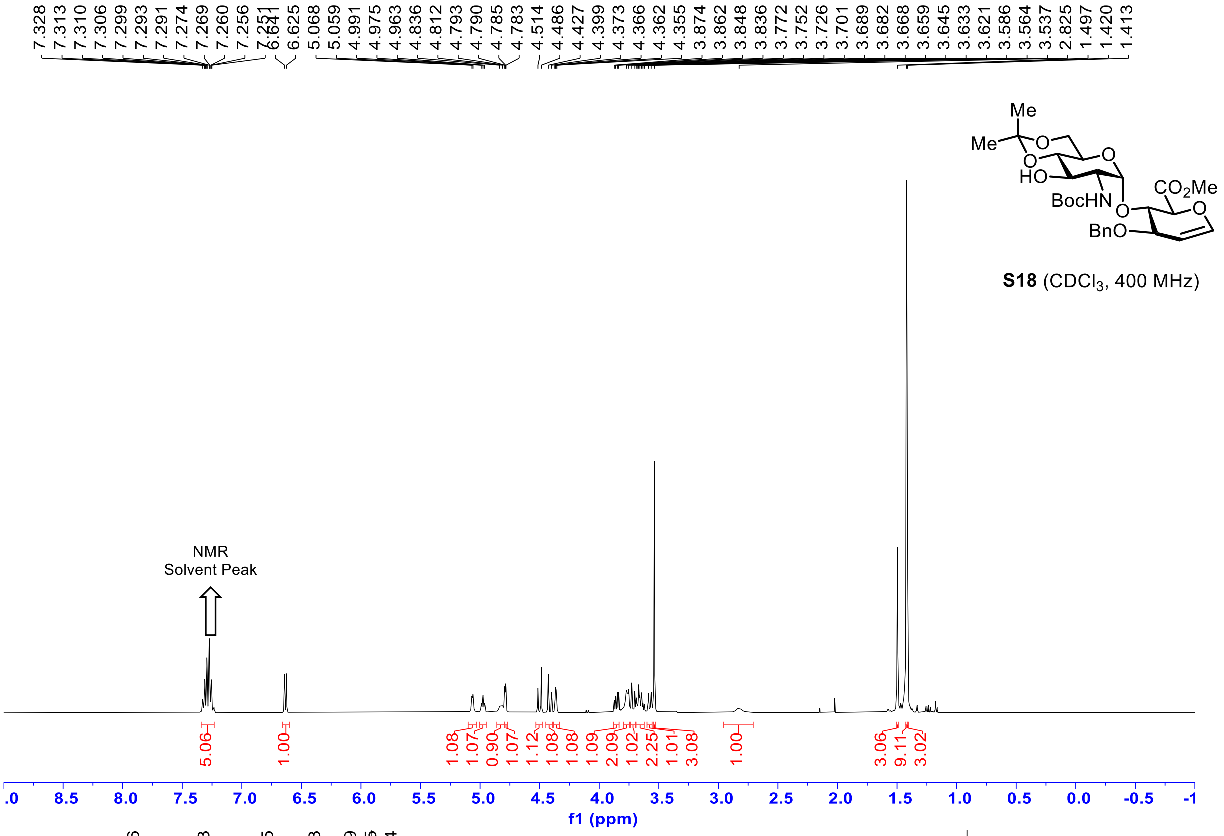
S18 ($CDCl_3$, 400 MHz)
NMR Solvent Peak
f1 (ppm)

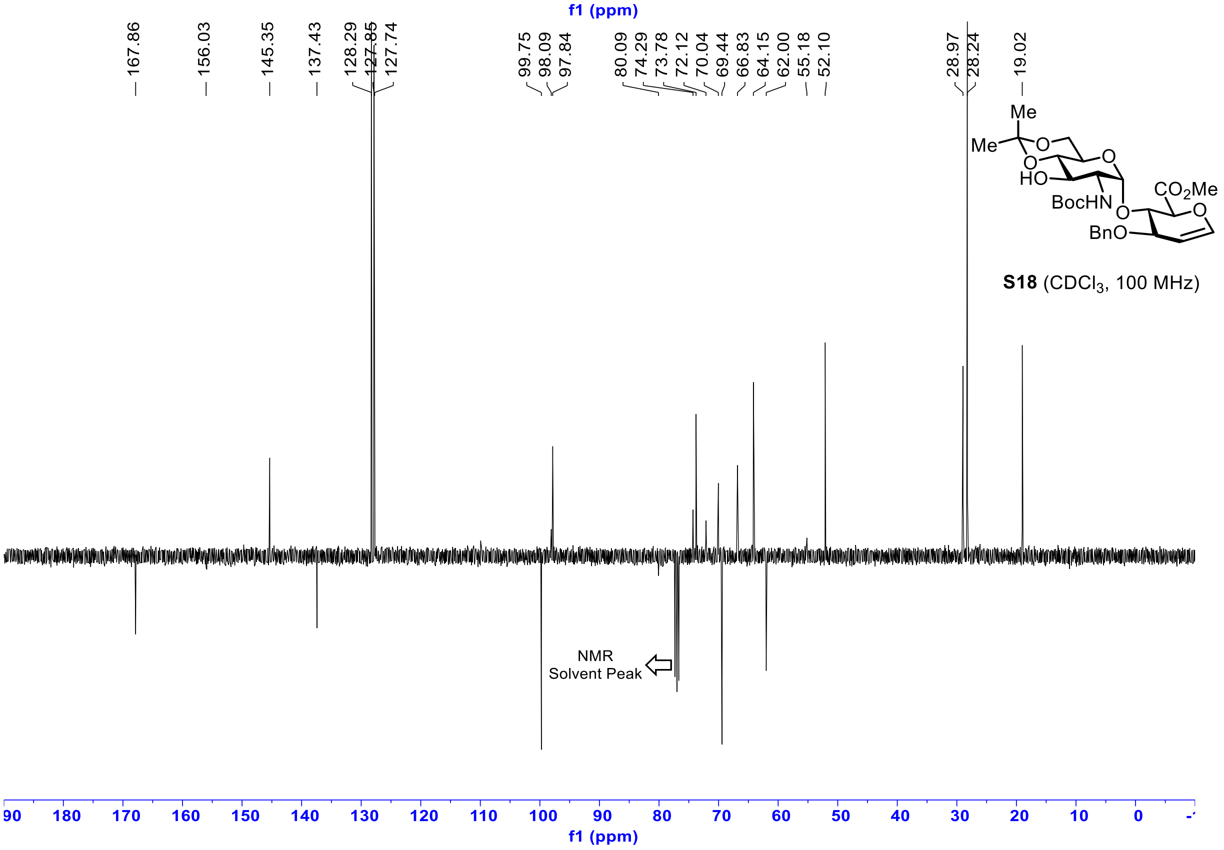
S18 ($CDCl_3$, 100 MHz)
NMR Solvent Peak
f1 (ppm)

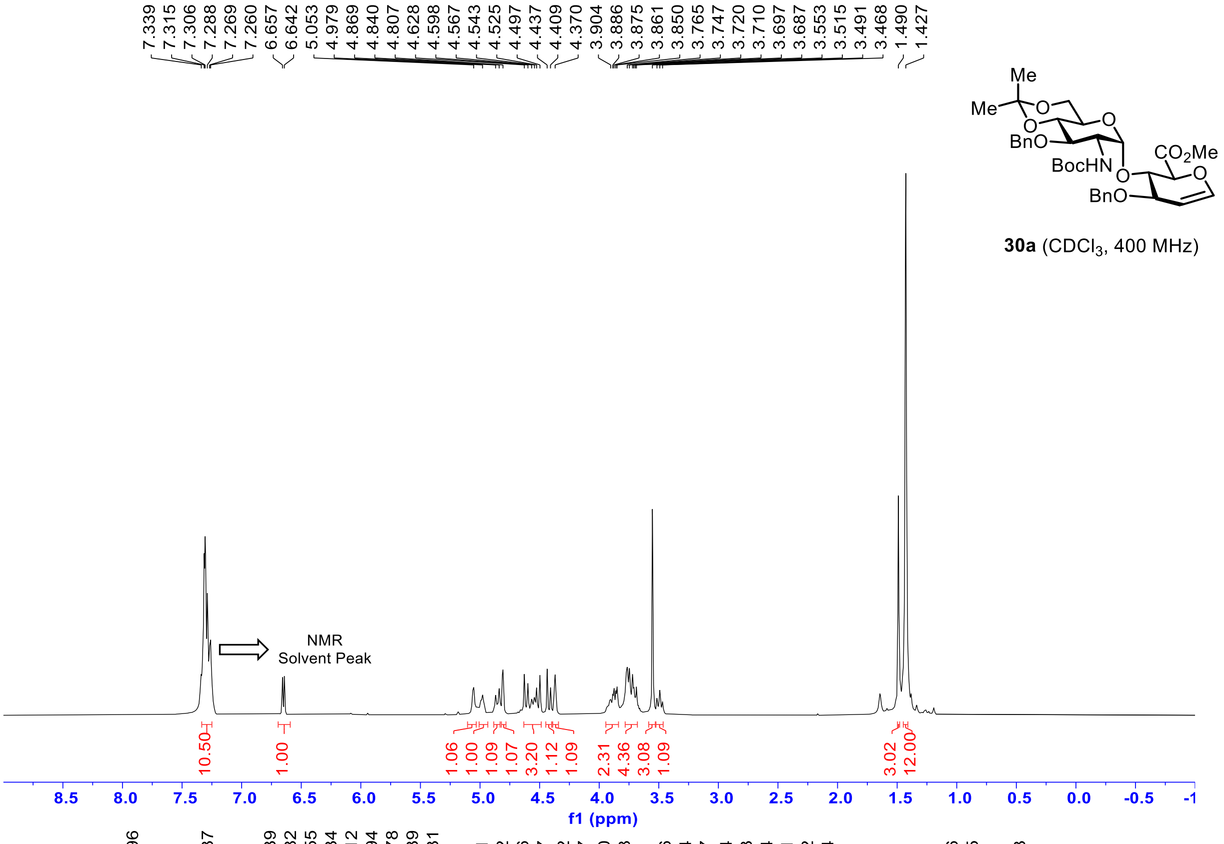

30a ($CDCl_3$, 400 MHz)

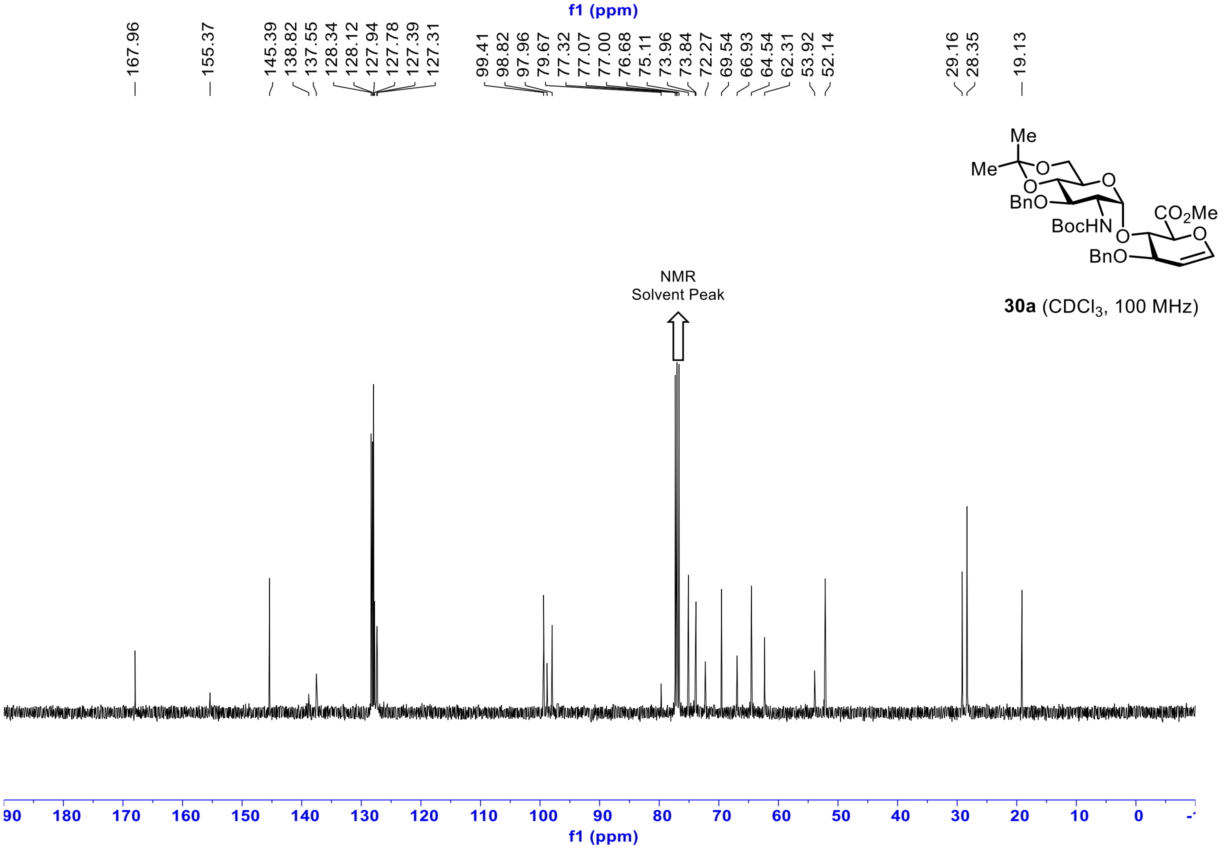

30a ($CDCl_3$, 100 MHz)

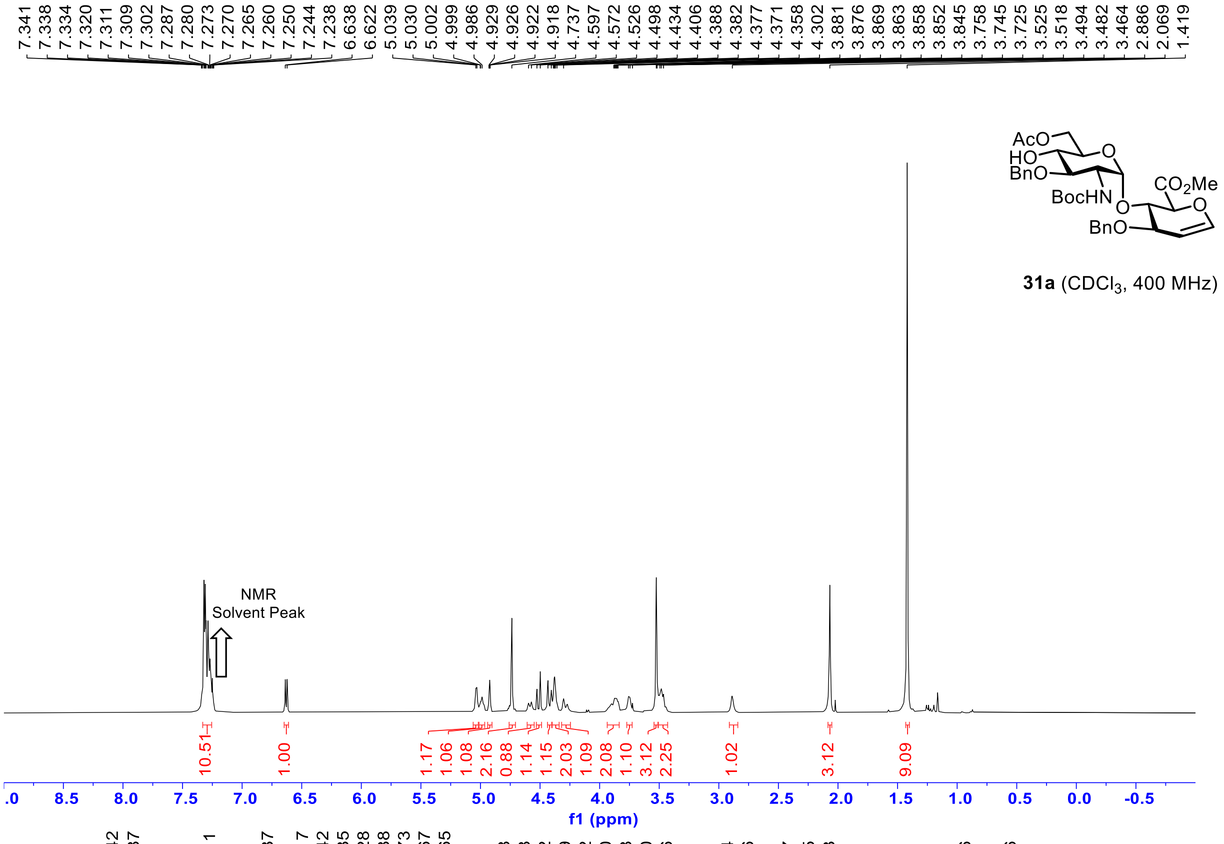

7.341 7.338 7.334 7.320 7.311 7.309 7.302 7.287 7.280 7.273 7.270 7.265 7.260 7.250 7.244 7.238 6.638 6.622 5.039 5.030 5.002 4.999 4.986 4.929 4.926 4.922 4.918 4.737 4.597 4.572 4.526 4.498 4.434 4.406 4.388 4.382 4.377 4.371 4.358 4.302 3.881 3.876 3.869 3.863 3.858 3.852 3.845 3.758 3.745 3.725 3.525 3.518 3.494 3.482 3.464 2.886 2.069 1.419
AcO
HO
BnO
BocHN
O
CO2Me
BnO
31a (CDCl3, 400 MHz)
NMR
Solvent Peak
10.51 1.00 1.17 1.06 1.08 2.16 0.88 1.14 1.15 2.03 1.09 2.08 1.10 3.12 2.25 1.02 3.12 9.09
f1 (ppm)

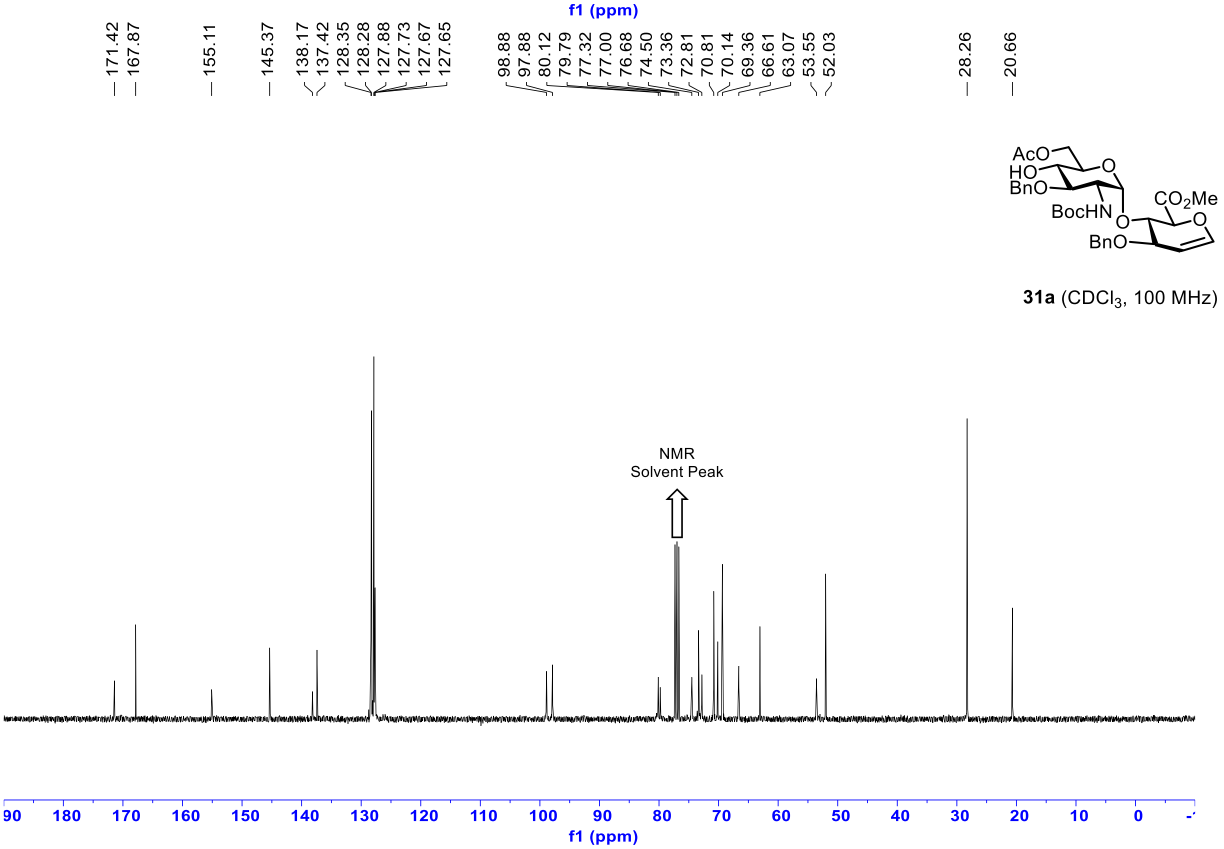

171.42 167.87 155.11 145.37 138.17 137.42 128.35 128.28 127.88 127.73 127.67 127.65 98.88 97.88 80.12 79.79 77.32 77.00 76.68 74.50 73.36 72.81 70.81 70.14 69.36 66.61 63.07 53.55 52.03 28.26 20.66
AcO
HO
BnO
BocHN
O
CO2Me
BnO
31a (CDCl3, 100 MHz)
NMR
Solvent Peak
f1 (ppm)

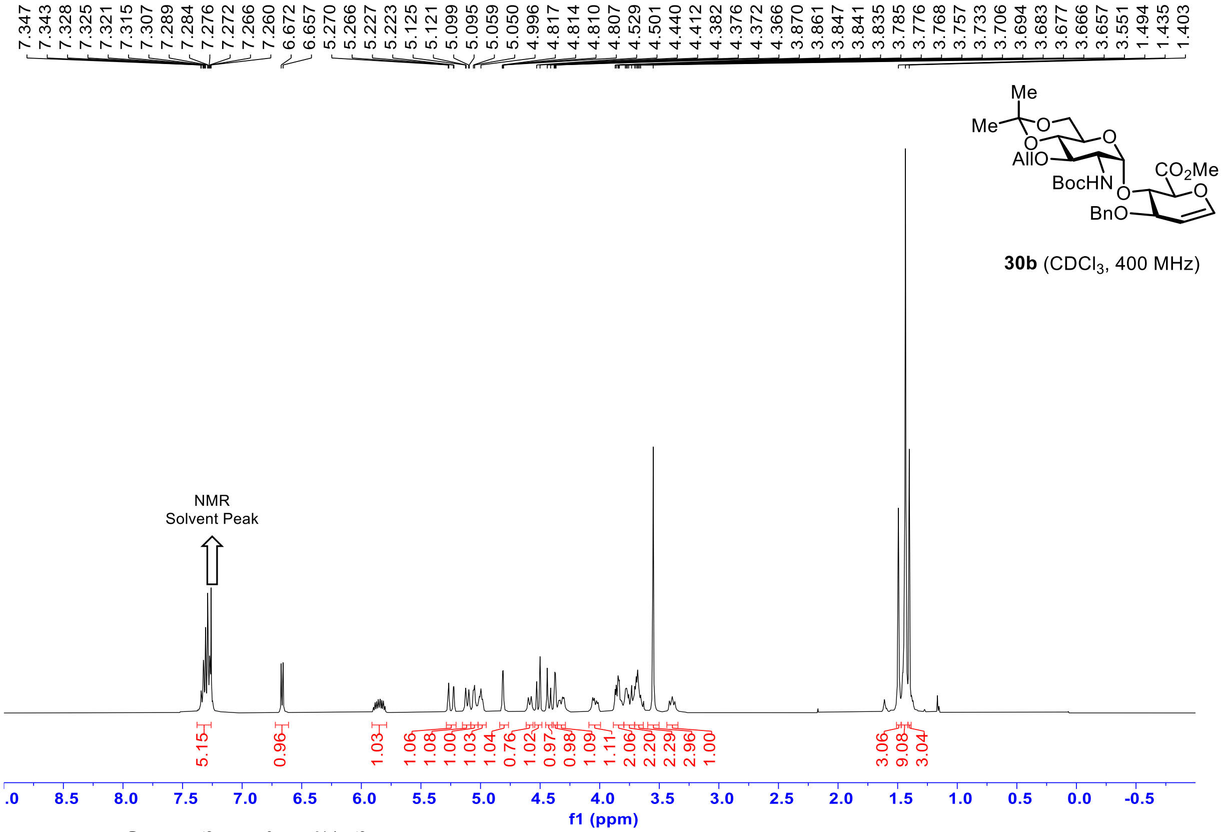
7.347 7.343 7.328 7.325 7.321 7.315 7.307 7.289 7.284 7.276 7.272 7.266 7.260 6.672 6.657 5.270 5.266 5.227 5.223 5.125 5.121 5.099 5.095 5.059 5.050 4.996 4.817 4.814 4.810 4.807 4.529 4.501 4.440 4.412 4.382 4.376 4.372 4.366 3.870 3.861 3.847 3.841 3.835 3.785 3.776 3.768 3.757 3.733 3.706 3.694 3.683 3.677 3.666 3.657 3.551 1.494 1.435 1.403
Me
Me
O
O
O
AllO
BocHN
O
CO_2Me
O
BnO
30b ($CDCl_3$, 400 MHz)
NMR
Solvent Peak
5.15 0.96 1.03 1.06 1.08 1.00 1.03 1.04 0.76 1.02 0.97 0.98 1.09 1.11 2.06 2.20 2.29 2.96 1.00 3.06 9.08 3.04
f1 (ppm)
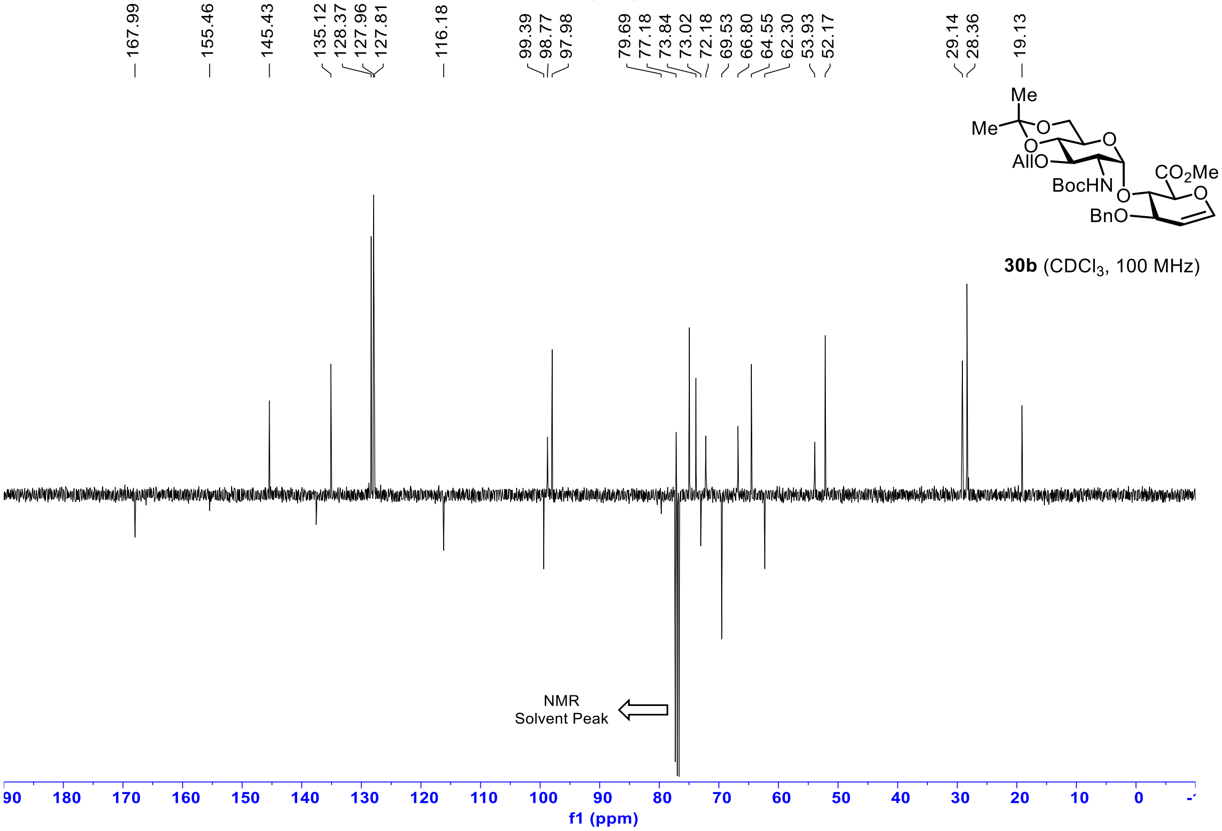
167.99 155.46 145.43 135.12 128.37 127.96 127.81 116.18 99.39 98.77 97.98 79.69 77.18 73.84 73.02 72.18 69.53 66.80 64.55 62.30 53.93 52.17 29.14 28.36 19.13
Me
Me
O
O
O
AllO
BocHN
O
CO_2Me
O
BnO
30b ($CDCl_3$, 100 MHz)
NMR
Solvent Peak
f1 (ppm)

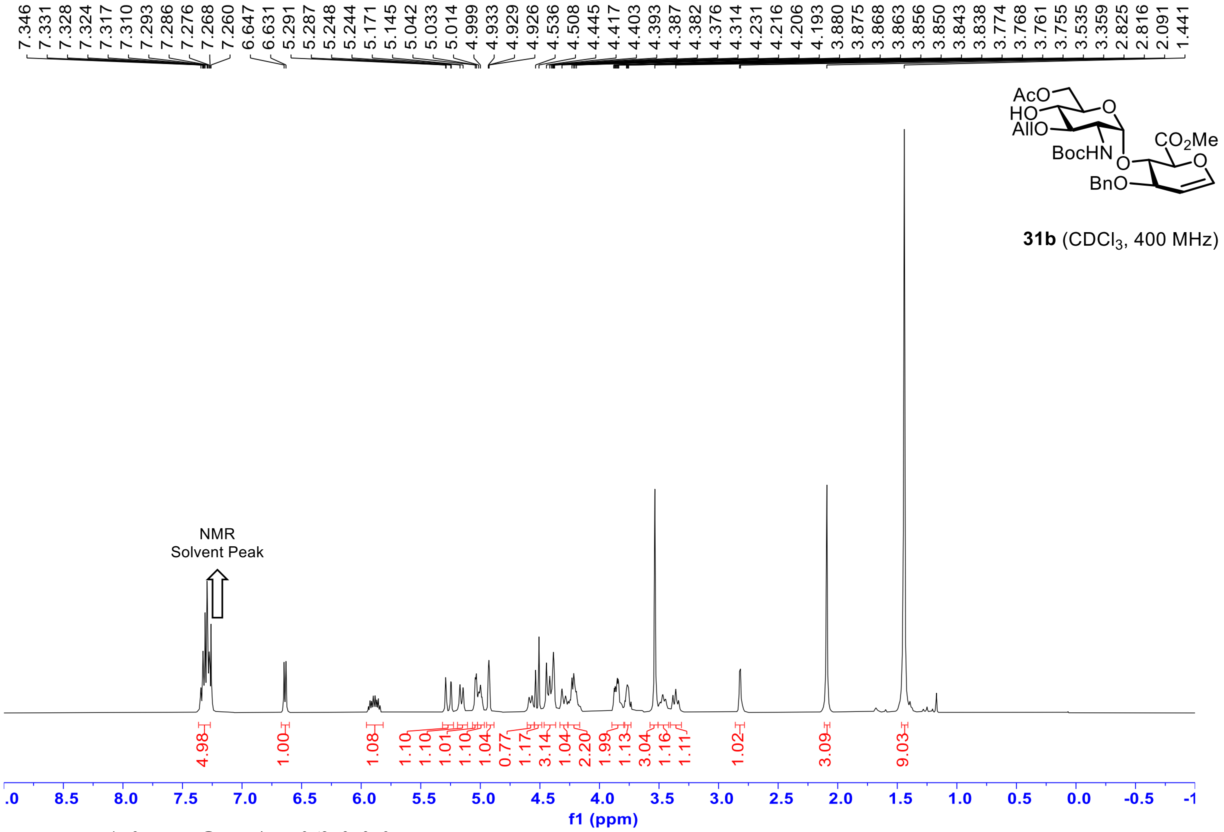
7.346 7.331 7.328 7.324 7.317 7.310 7.293 7.286 7.276 7.268 7.260 6.647 6.631 5.291 5.287 5.248 5.244 5.171 5.145 5.042 5.033 5.014 4.999 4.933 4.929 4.926 4.536 4.508 4.445 4.417 4.403 4.393 4.387 4.382 4.376 4.314 4.231 4.216 4.206 4.193 3.880 3.875 3.868 3.863 3.856 3.850 3.843 3.838 3.774 3.768 3.761 3.755 3.535 3.359 2.825 2.816 2.091 1.441
AcO
HO
AllO
BocHN
O
CO2Me
BnO
31b (CDCl3, 400 MHz)
NMR
Solvent Peak
4.98
1.00
1.08
1.10
1.10
1.01
1.10
1.04
0.77
1.17
3.14
1.04
2.20
1.99
1.13
3.04
1.16
1.11
1.02
3.09
9.03
.0 8.5 8.0 7.5 7.0 6.5 6.0 5.5 5.0 4.5 4.0 3.5 3.0 2.5 2.0 1.5 1.0 0.5 0.0 -0.5 -1
f1 (ppm)

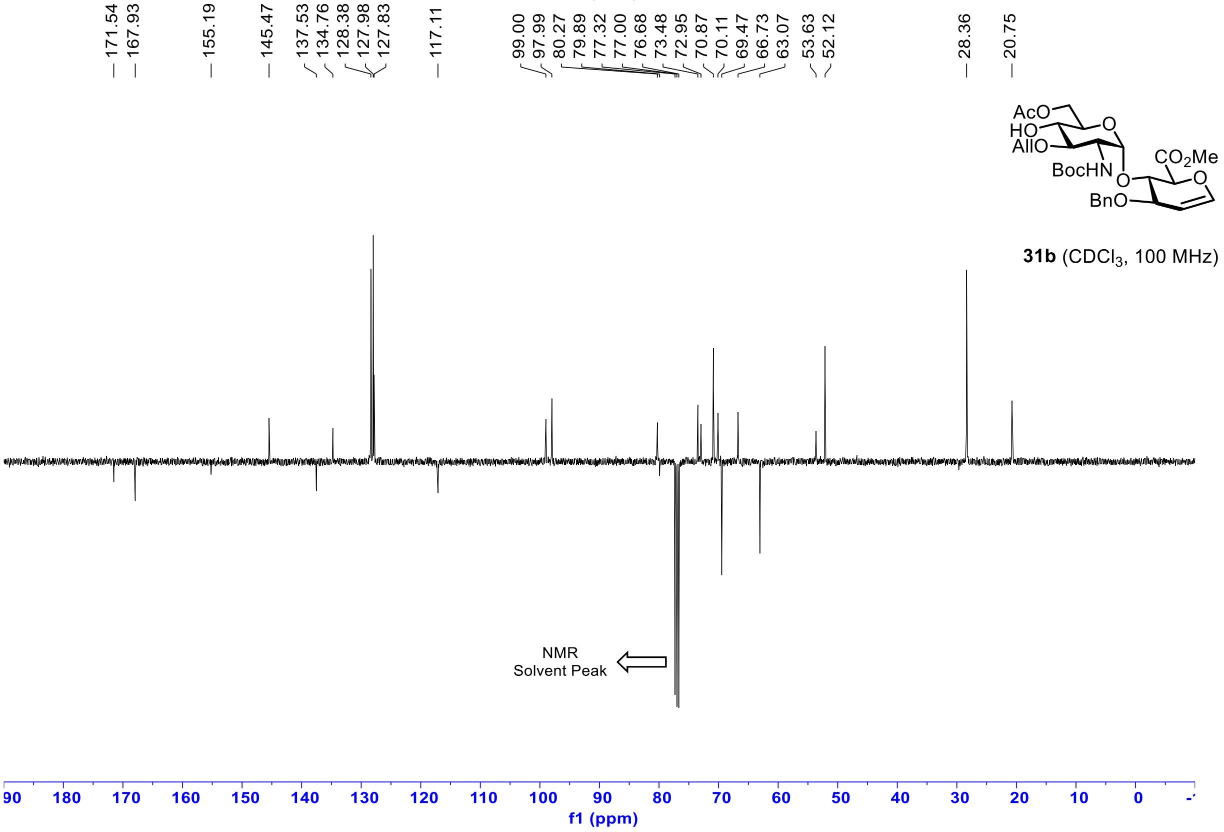
171.54 167.93 155.19 145.47 137.53 134.76 128.38 127.98 127.83 117.11 99.00 97.99 80.27 79.89 77.32 77.00 76.68 73.48 72.95 70.87 70.11 69.47 66.73 63.07 53.63 52.12 28.36 20.75
AcO
HO
AllO
BocHN
O
CO2Me
BnO
31b (CDCl3, 100 MHz)
NMR
Solvent Peak
90 180 170 160 150 140 130 120 110 100 90 80 70 60 50 40 30 20 10 0
f1 (ppm)

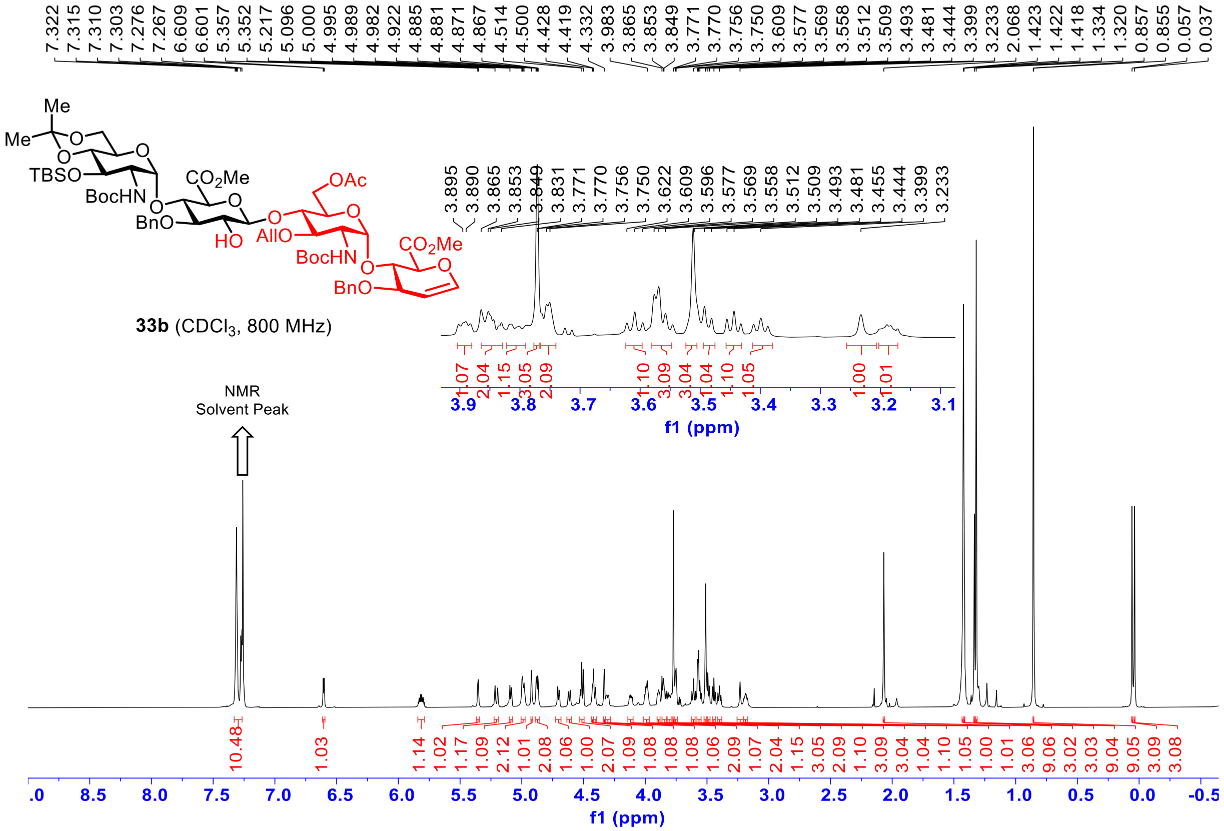
7.322 7.315 7.310 7.303 7.276 7.267 6.609 6.601 5.357 5.352 5.217 5.096 5.000 4.995 4.989 4.982 4.922 4.885 4.881 4.871 4.867 4.514 4.500 4.428 4.419 4.332 3.983 3.865 3.853 3.849 3.771 3.770 3.756 3.750 3.609 3.577 3.569 3.558 3.512 3.509 3.493 3.481 3.444 3.399 3.233 2.068 1.423 1.422 1.418 1.334 1.320 0.857 0.855 0.057 0.037
3.895 3.890 3.865 3.853 3.849 3.831 3.771 3.770 3.756 3.750 3.622 3.609 3.596 3.577 3.569 3.558 3.512 3.509 3.493 3.481 3.455 3.444 3.399 3.233
1.07 2.04 1.15 3.05 2.09 1.10 3.09 3.04 1.04 1.10 1.05 1.00 1.01
f1 (ppm)
33b (CDCl3, 800 MHz)
NMR
Solvent Peak
10.48 1.03 1.14 1.02 1.17 1.09 2.12 1.01 2.08 1.06 1.00 2.07 1.09 1.08 1.08 1.08 1.06 2.09 1.07 2.04 1.15 3.05 2.09 1.10 3.09 3.04 1.04 1.10 1.05 1.00 1.01 3.06 9.06 3.02 3.03 9.04 9.05 3.09 3.08
f1 (ppm)

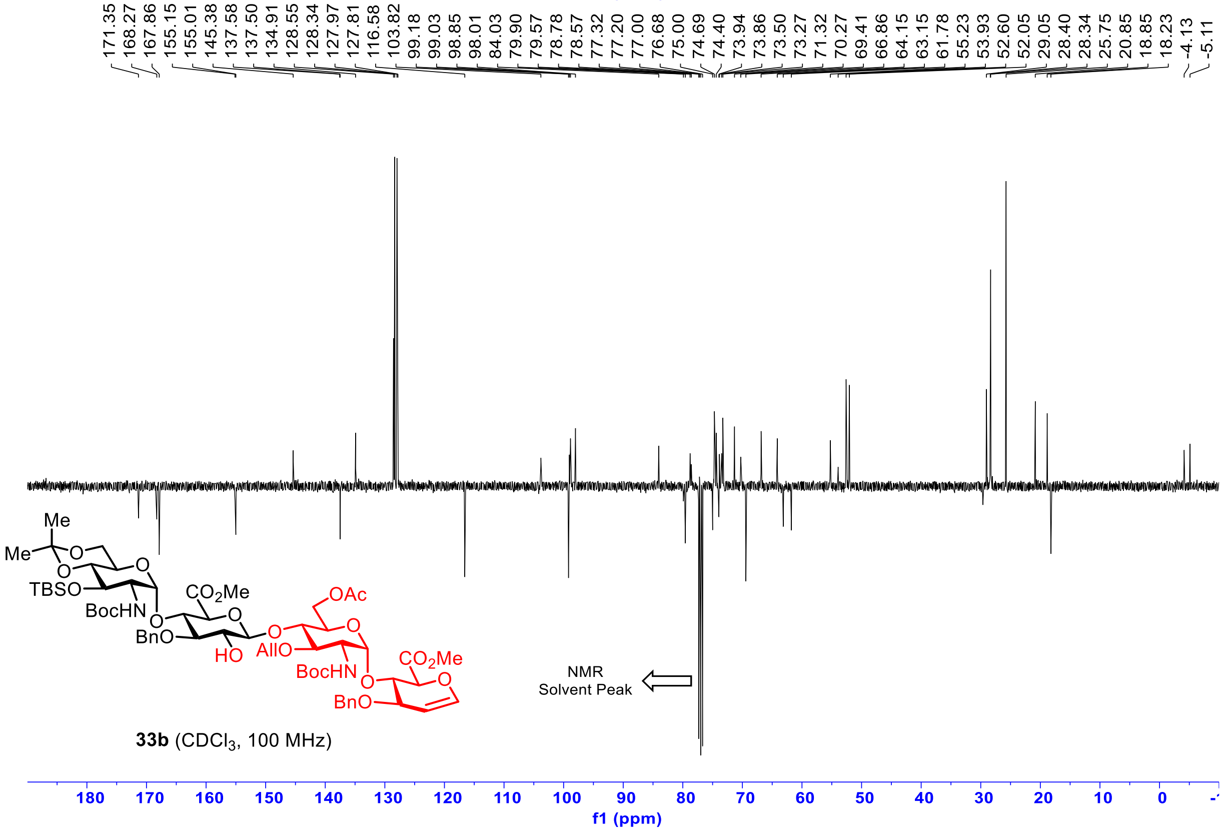
171.35 168.27 167.86 155.15 155.01 145.38 137.58 137.50 134.91 128.55 128.34 127.97 127.81 116.58 103.82 99.18 99.03 98.85 98.01 84.03 79.90 79.57 78.78 78.57 77.32 77.20 77.00 76.68 75.00 74.69 74.40 73.94 73.86 73.50 73.27 71.32 70.27 69.41 66.86 64.15 63.15 61.78 55.23 53.93 52.60 52.05 29.05 28.40 28.34 25.75 20.85 18.85 18.23 -4.13 -5.11
NMR
Solvent Peak
33b (CDCl3, 100 MHz)
f1 (ppm)

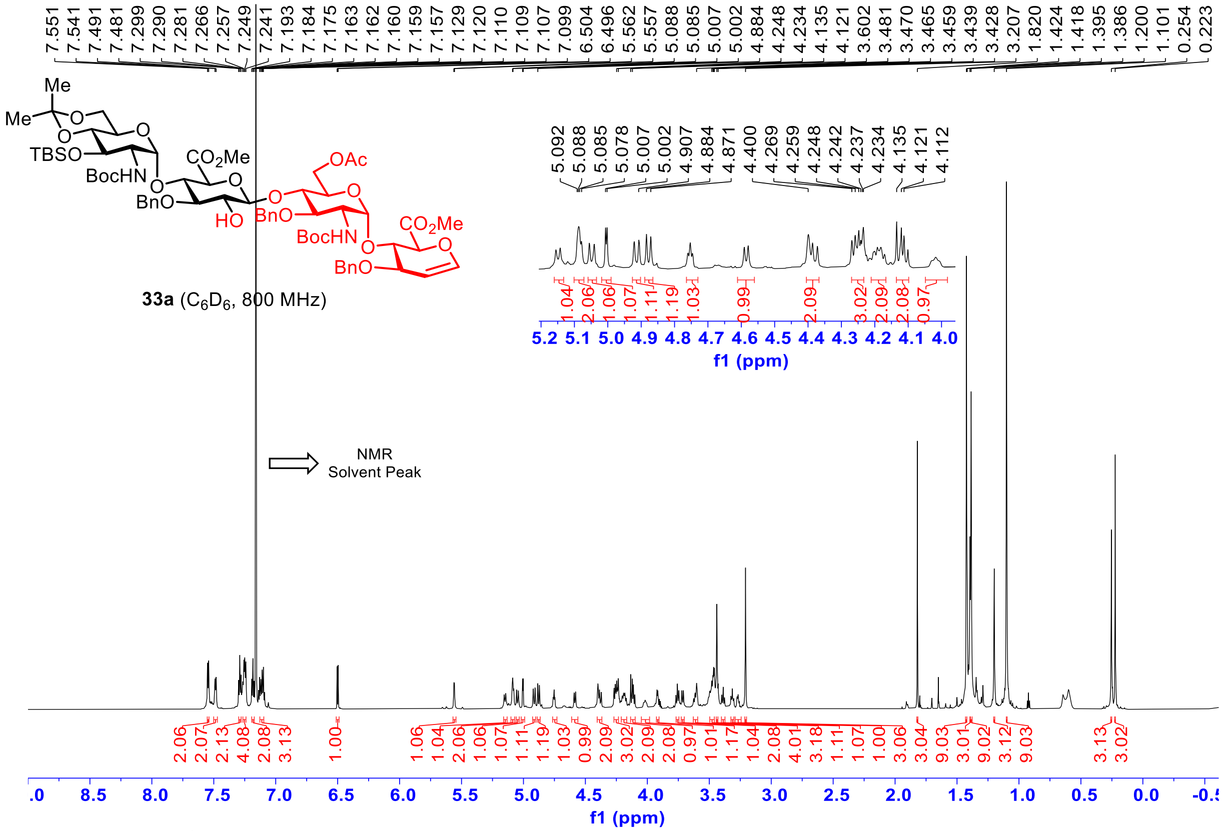
7.551 7.541 7.491 7.481 7.299 7.290 7.281 7.266 7.257 7.249 7.241 7.193 7.184 7.175 7.163 7.162 7.160 7.159 7.157 7.129 7.120 7.110 7.109 7.107 7.099 6.504 6.496 5.562 5.557 5.088 5.085 5.007 5.002 4.884 4.248 4.234 4.135 4.121 3.602 3.481 3.470 3.465 3.459 3.439 3.428 3.207 1.820 1.424 1.418 1.395 1.386 1.200 1.101 0.254 0.223
5.092 5.088 5.085 5.078 5.007 5.002 4.907 4.884 4.871 4.400 4.269 4.259 4.248 4.242 4.237 4.234 4.135 4.121 4.112
1.04 2.06 1.06 1.07 1.11 1.19 1.03 0.99 2.09 3.02 2.09 2.08 0.97
5.2 5.1 5.0 4.9 4.8 4.7 4.6 4.5 4.4 4.3 4.2 4.1 4.0
f1 (ppm)
Me
Me
O
O
O
TBSO
BocHN
O
CO2Me
O
BnO
HO
O
OAc
O
BnO
BocHN
O
CO2Me
O
BnO
33a (C6D6, 800 MHz)
NMR
Solvent Peak
2.06 2.07 2.13 4.08 2.08 3.13 1.00 1.06 1.04 2.06 1.06 1.07 1.11 1.19 1.03 0.99 2.09 3.02 2.09 2.08 0.97 1.01 1.17 1.04 2.08 4.01 3.18 1.11 1.07 1.00 3.06 3.04 9.03 3.01 9.02 3.12 9.03 3.13 3.02
.0 8.5 8.0 7.5 7.0 6.5 6.0 5.5 5.0 4.5 4.0 3.5 3.0 2.5 2.0 1.5 1.0 0.5 0.0 -0.5
f1 (ppm)

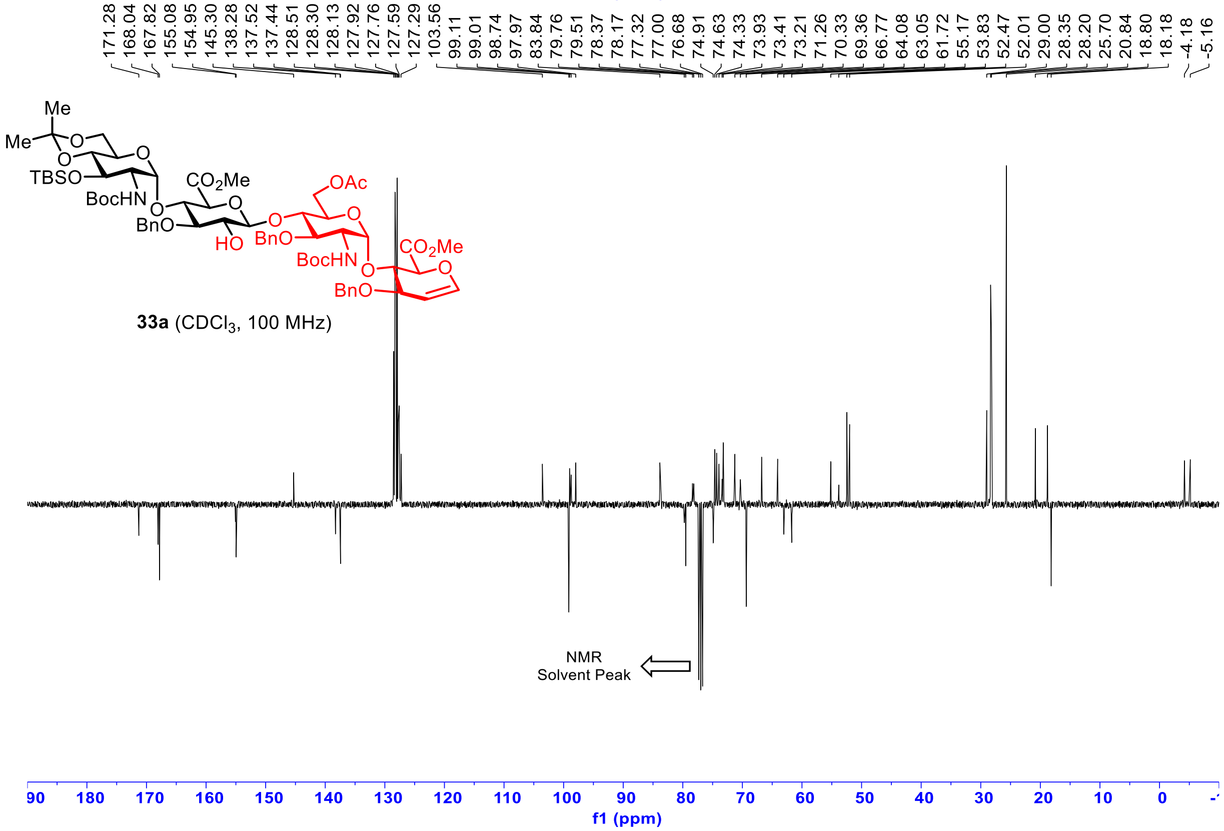
171.28 168.04 167.82 155.08 154.95 145.30 138.28 137.52 137.44 128.51 128.30 128.13 127.92 127.76 127.59 127.29 103.56 99.11 99.01 98.74 97.97 83.84 79.76 79.51 78.37 78.17 77.32 77.00 76.68 74.91 74.63 74.33 73.93 73.41 73.21 71.26 70.33 69.36 66.77 64.08 63.05 61.72 55.17 53.83 52.47 52.01 29.00 28.35 28.20 25.70 20.84 18.80 18.18 -4.18 -5.16
Me
Me
O
O
O
TBSO
BocHN
O
CO2Me
O
BnO
HO
O
OAc
O
BnO
BocHN
O
CO2Me
O
BnO
33a (CDCl3, 100 MHz)
NMR
Solvent Peak
90 180 170 160 150 140 130 120 110 100 90 80 70 60 50 40 30 20 10 0
f1 (ppm)

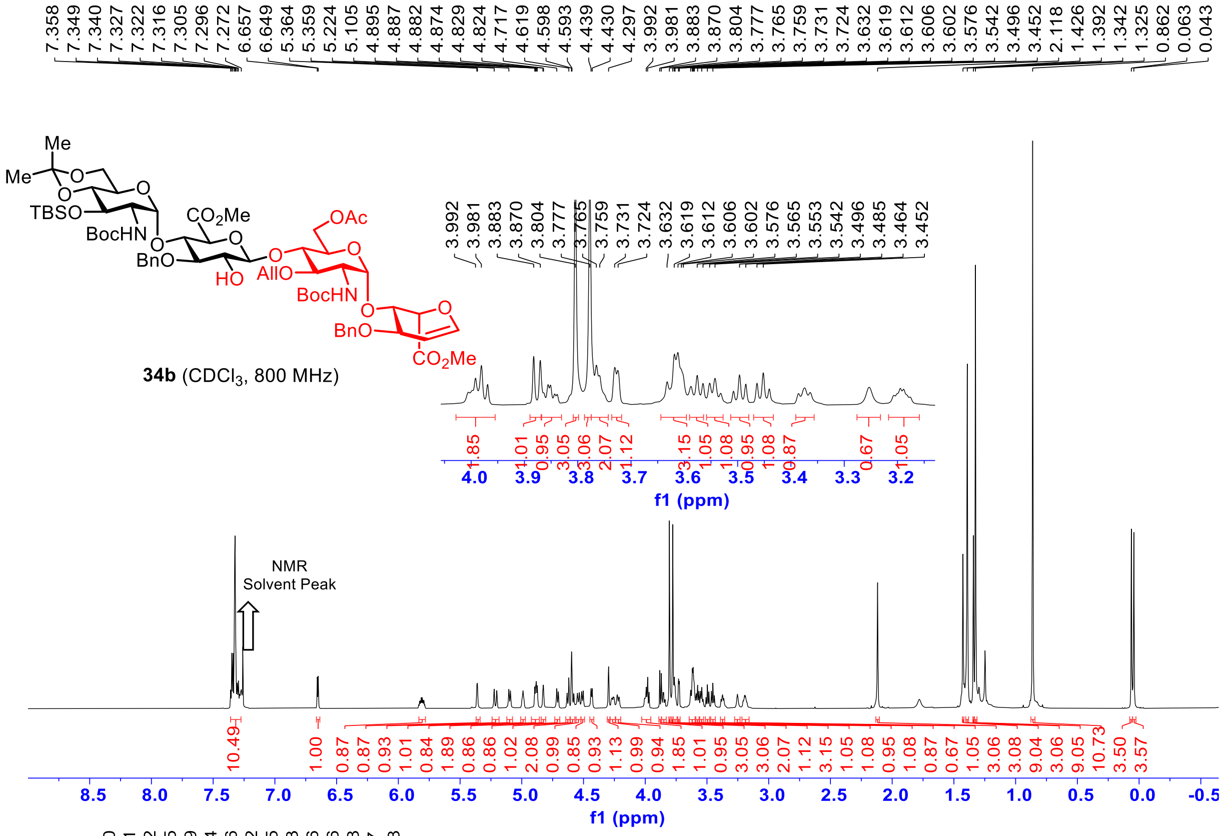
7.358 7.349 7.340 7.327 7.322 7.316 7.305 7.296 7.272 6.657 6.649 5.364 5.359 5.224 5.105 4.895 4.887 4.882 4.874 4.829 4.824 4.717 4.619 4.598 4.593 4.439 4.430 4.297 3.992 3.981 3.883 3.870 3.804 3.777 3.765 3.759 3.731 3.724 3.632 3.619 3.612 3.606 3.602 3.576 3.542 3.496 3.452 2.118 1.426 1.392 1.342 1.325 0.862 0.063 0.043
Me
Me
O
O
O
TBSO
BocHN
CO2Me
O
O
BnO
HO
O
OAc
O
AllO
BocHN
O
O
BnO
CO2Me
34b (CDCl3, 800 MHz)
3.992 3.981 3.883 3.870 3.804 3.777 3.765 3.759 3.731 3.724 3.632 3.619 3.612 3.606 3.602 3.576 3.565 3.553 3.542 3.496 3.485 3.464 3.452
1.85 1.01 0.95 3.05 3.06 2.07 1.12 3.15 1.05 1.08 0.95 1.08 0.87 0.67 1.05
4.0 3.9 3.8 3.7 3.6 3.5 3.4 3.3 3.2
f1 (ppm)
NMR
Solvent Peak
10.49 1.00 0.87 0.87 0.93 1.01 0.84 1.89 0.86 0.86 1.02 2.08 0.99 0.85 0.93 1.13 1.99 0.99 0.94 1.85 1.01 0.95 3.05 3.06 2.07 1.12 3.15 1.05 1.08 0.95 1.08 0.87 0.67 1.05 3.06 3.08 9.04 3.06 9.05 10.73 3.50 3.57
8.5 8.0 7.5 7.0 6.5 6.0 5.5 5.0 4.5 4.0 3.5 3.0 2.5 2.0 1.5 1.0 0.5 0.0 -0.5
f1 (ppm)

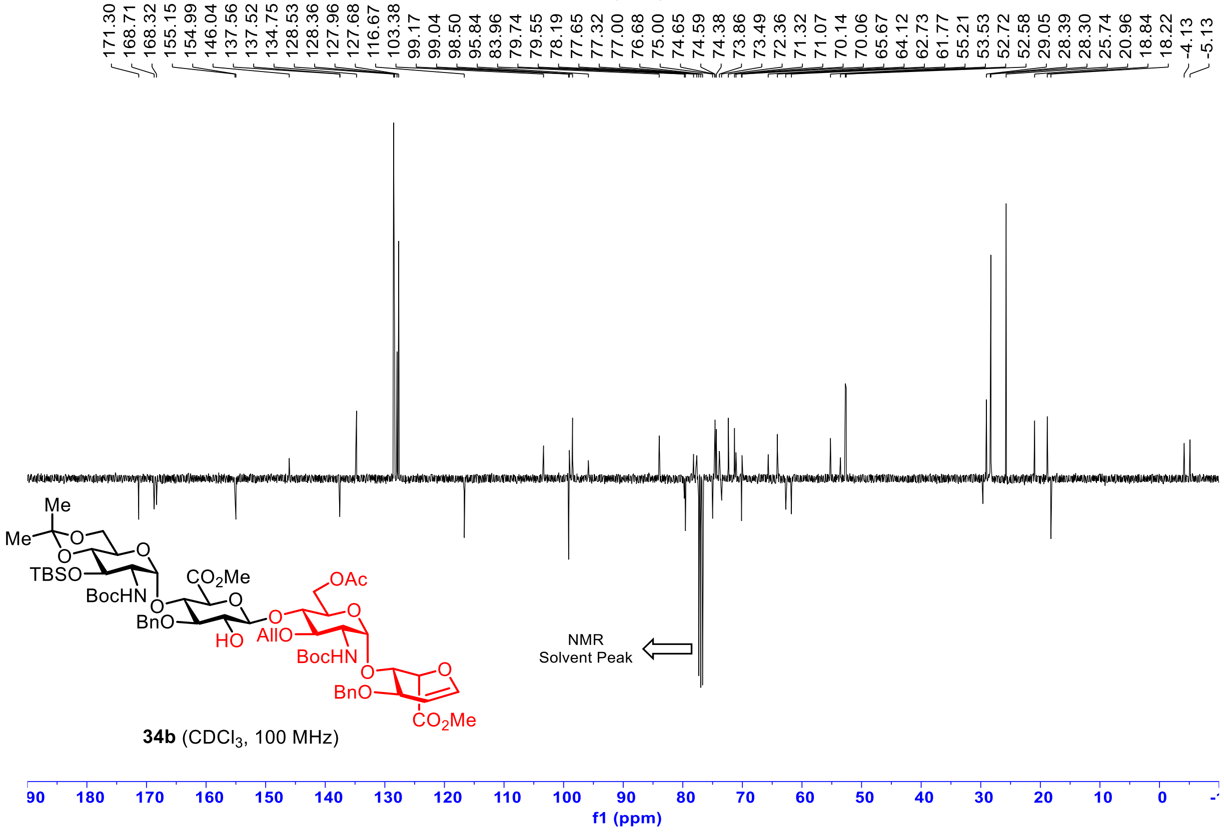
171.30 168.71 168.32 155.15 154.99 146.04 137.56 137.52 134.75 128.53 128.36 127.96 127.68 116.67 103.38 99.17 99.04 98.50 95.84 83.96 79.74 79.55 78.19 77.65 77.32 77.00 76.68 75.00 74.65 74.59 74.38 73.86 73.49 72.36 71.32 71.07 70.14 70.06 65.67 64.12 62.73 61.77 55.21 53.53 52.72 52.58 29.05 28.39 28.30 25.74 20.96 18.84 18.22 -4.13 -5.13
Me
Me
O
O
O
TBSO
BocHN
CO2Me
O
O
BnO
HO
O
OAc
O
AllO
BocHN
O
O
BnO
CO2Me
NMR
Solvent Peak
34b (CDCl3, 100 MHz)
90 180 170 160 150 140 130 120 110 100 90 80 70 60 50 40 30 20 10 0
f1 (ppm)

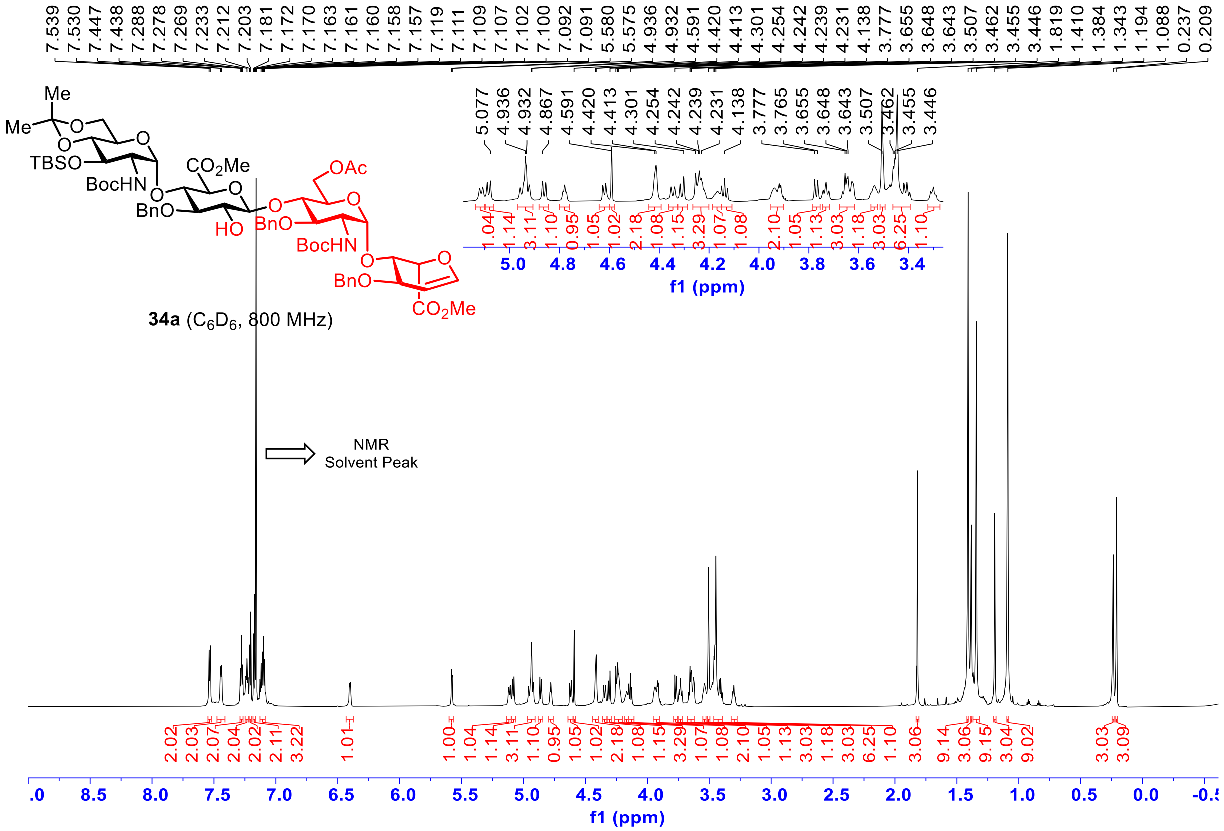

34a (C_6D_6, 800 MHz)

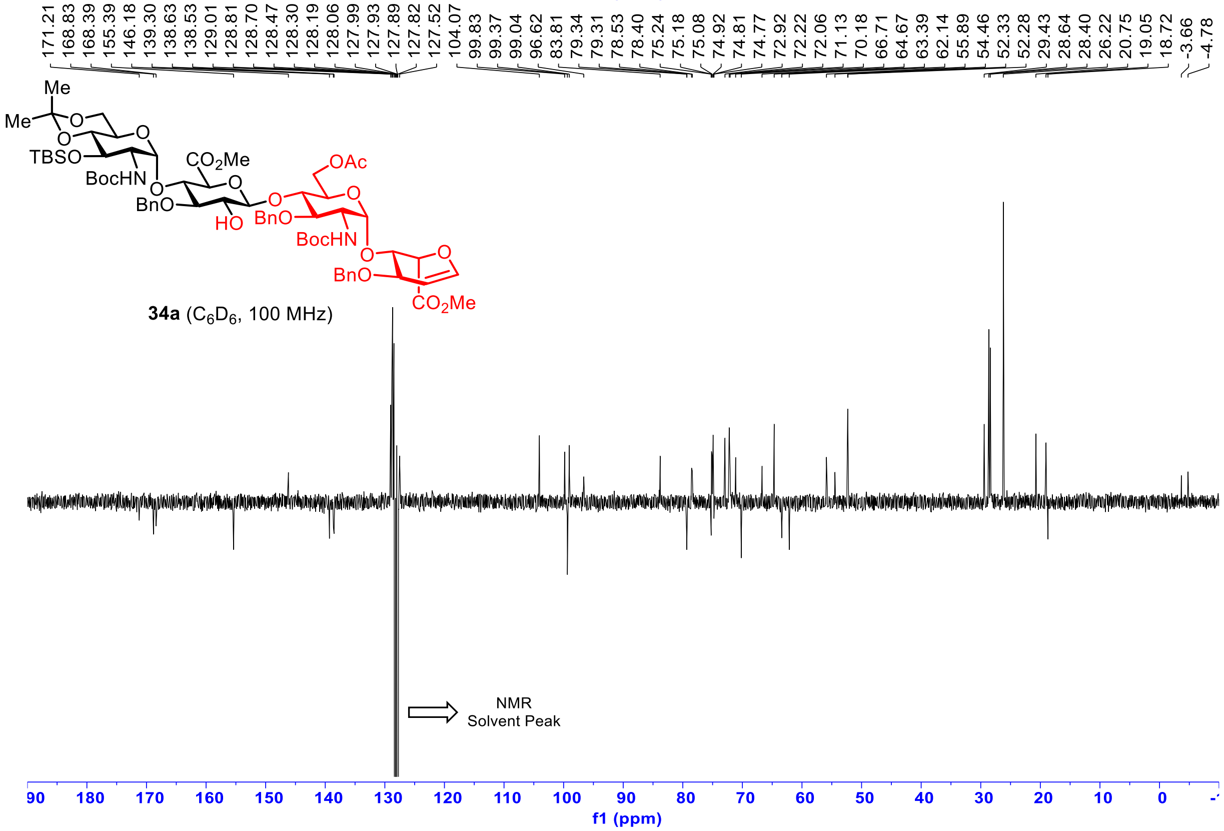

34a (C_6D_6, 100 MHz)

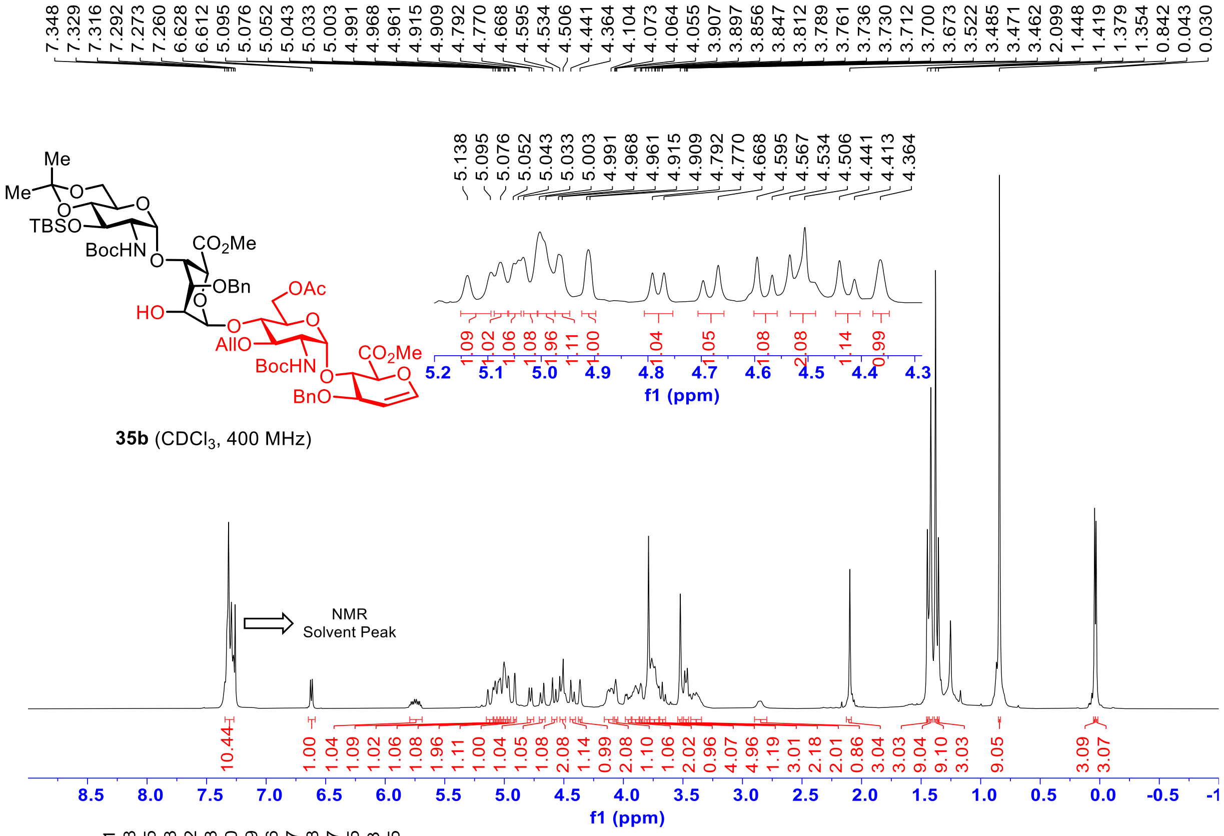

35b (CDCl3, 400 MHz)
NMR Solvent Peak
f1 (ppm)

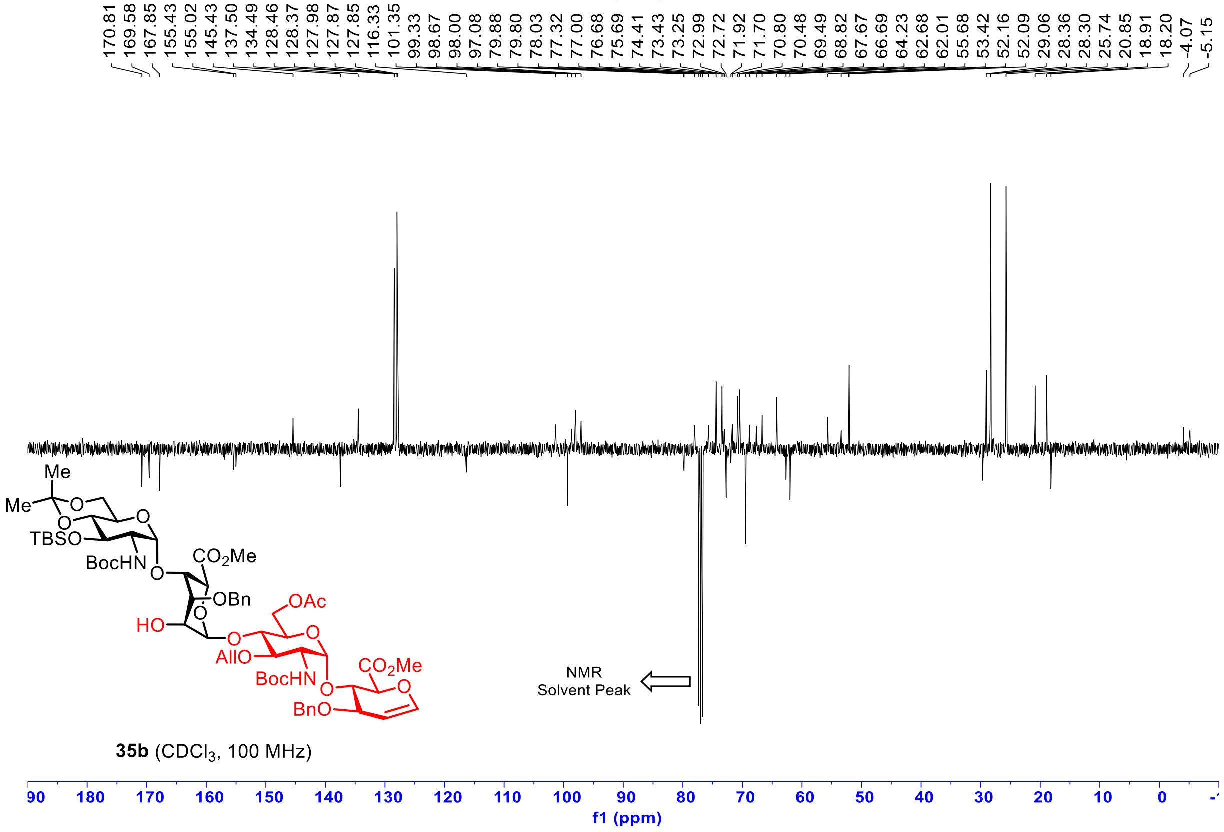

35b (CDCl3, 100 MHz)
NMR Solvent Peak
f1 (ppm)

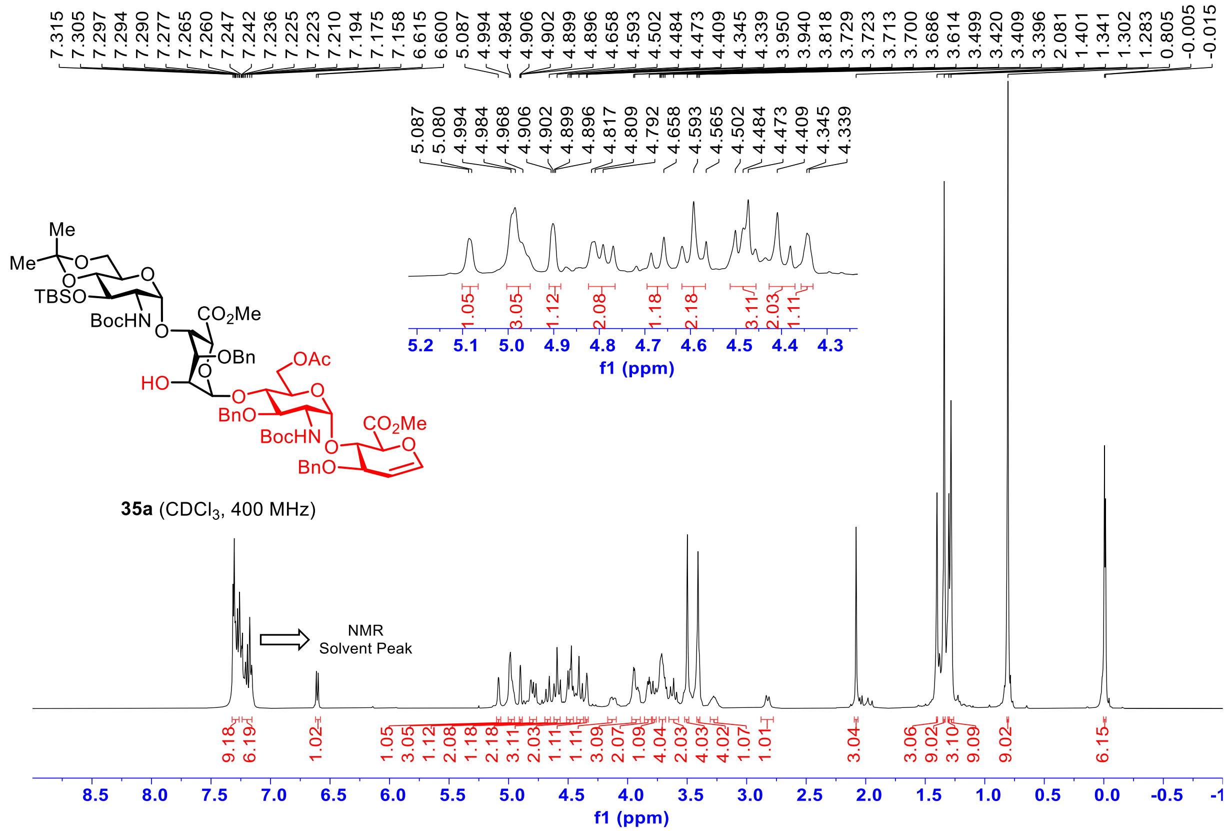

7.315 7.305 7.297 7.294 7.290 7.277 7.265 7.260 7.247 7.242 7.236 7.225 7.223 7.210 7.194 7.175 7.158 6.615 6.600 5.087 4.994 4.984 4.906 4.902 4.899 4.896 4.658 4.593 4.502 4.484 4.473 4.409 4.345 4.339 3.950 3.940 3.818 3.729 3.723 3.713 3.700 3.686 3.614 3.499 3.420 3.409 3.396 2.081 1.401 1.341 1.302 1.283 0.805 -0.005 -0.015
5.087 5.080 4.994 4.984 4.968 4.906 4.902 4.899 4.896 4.817 4.809 4.792 4.658 4.593 4.565 4.502 4.484 4.473 4.409 4.345 4.339
1.05 3.05 1.12 2.08 1.18 2.18 3.11 2.03 1.11
f1 (ppm)
Me
Me
TBSO
BocHN
CO_2Me
OBn
OAc
HO
BnO
BocHN
CO_2Me
BnO
35a ($CDCl_3$, 400 MHz)
NMR
Solvent Peak
9.18 6.19 1.02 1.05 3.05 1.12 2.08 1.18 2.18 3.11 2.03 1.11 1.11 3.09 2.07 1.09 4.04 2.03 4.03 4.02 1.07 1.01 3.04 3.06 9.02 3.10 9.09 9.02 6.15
f1 (ppm)

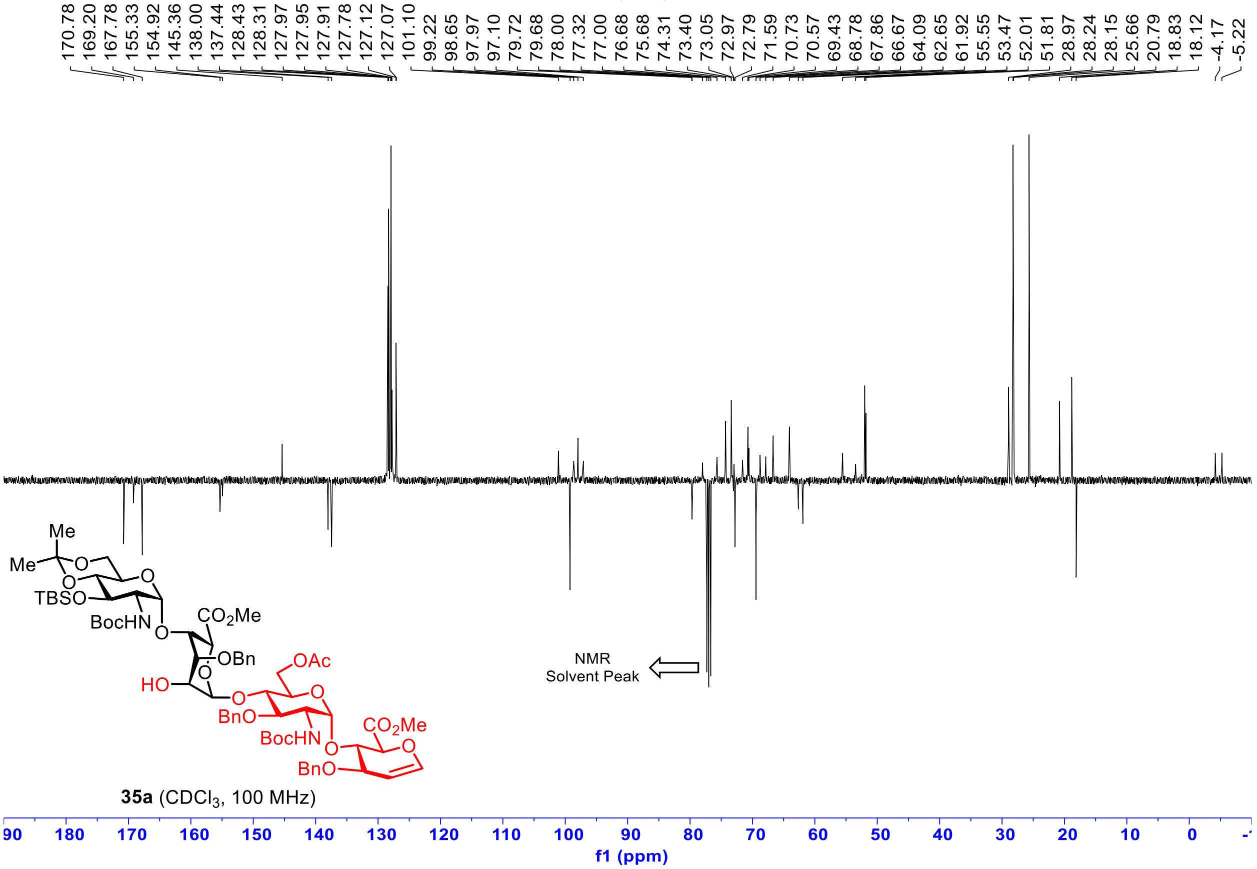

170.78 169.20 167.78 155.33 154.92 145.36 138.00 137.44 128.43 128.31 127.97 127.95 127.91 127.78 127.12 127.07 101.10 99.22 98.65 97.97 97.10 79.72 79.68 78.00 77.32 77.00 76.68 75.68 74.31 73.40 73.05 72.97 72.79 71.59 70.73 70.57 69.43 68.78 67.86 66.67 64.09 62.65 61.92 55.55 53.47 52.01 51.81 28.97 28.24 28.15 25.66 20.79 18.83 18.12 -4.17 -5.22
Me
Me
TBSO
BocHN
CO_2Me
OBn
OAc
HO
BnO
BocHN
CO_2Me
BnO
NMR
Solvent Peak
35a ($CDCl_3$, 100 MHz)
f1 (ppm)

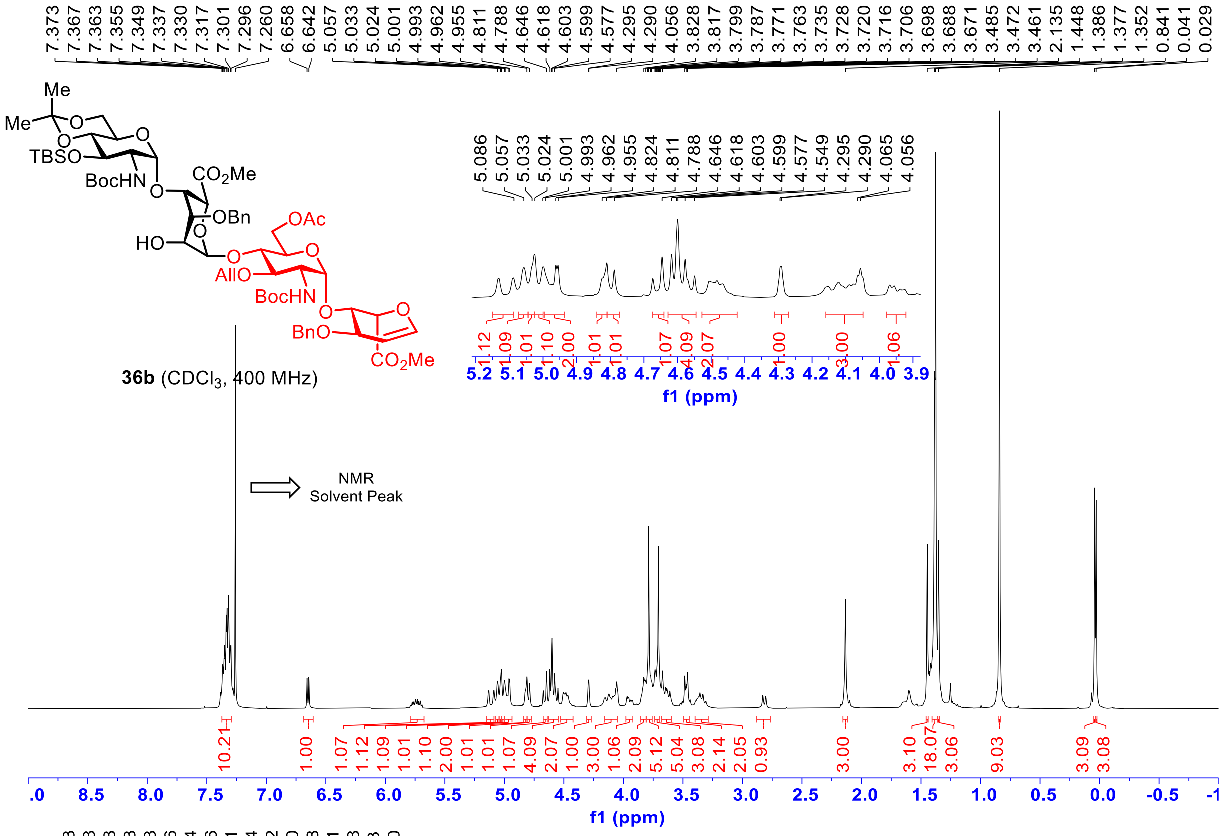
36b (CDCl3, 400 MHz)
NMR Solvent Peak
f1 (ppm)

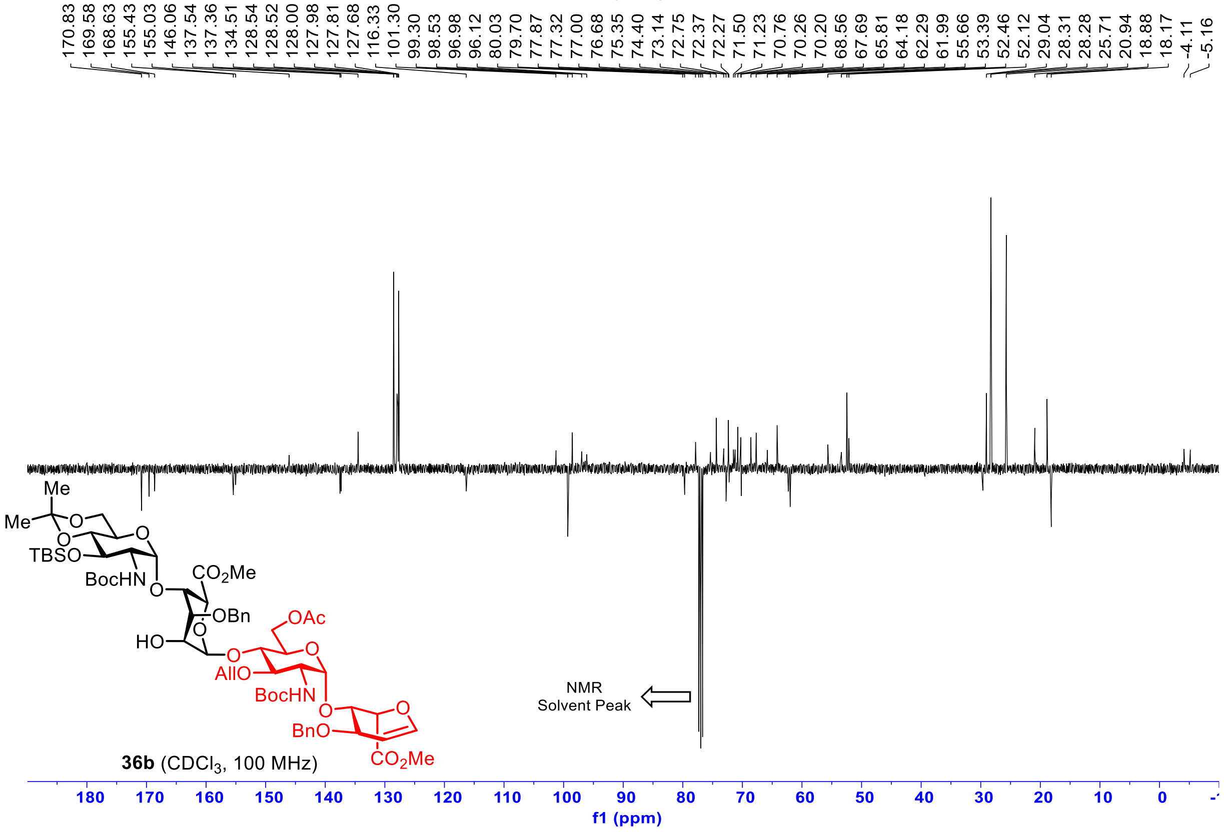
36b (CDCl3, 100 MHz)
NMR Solvent Peak
f1 (ppm)

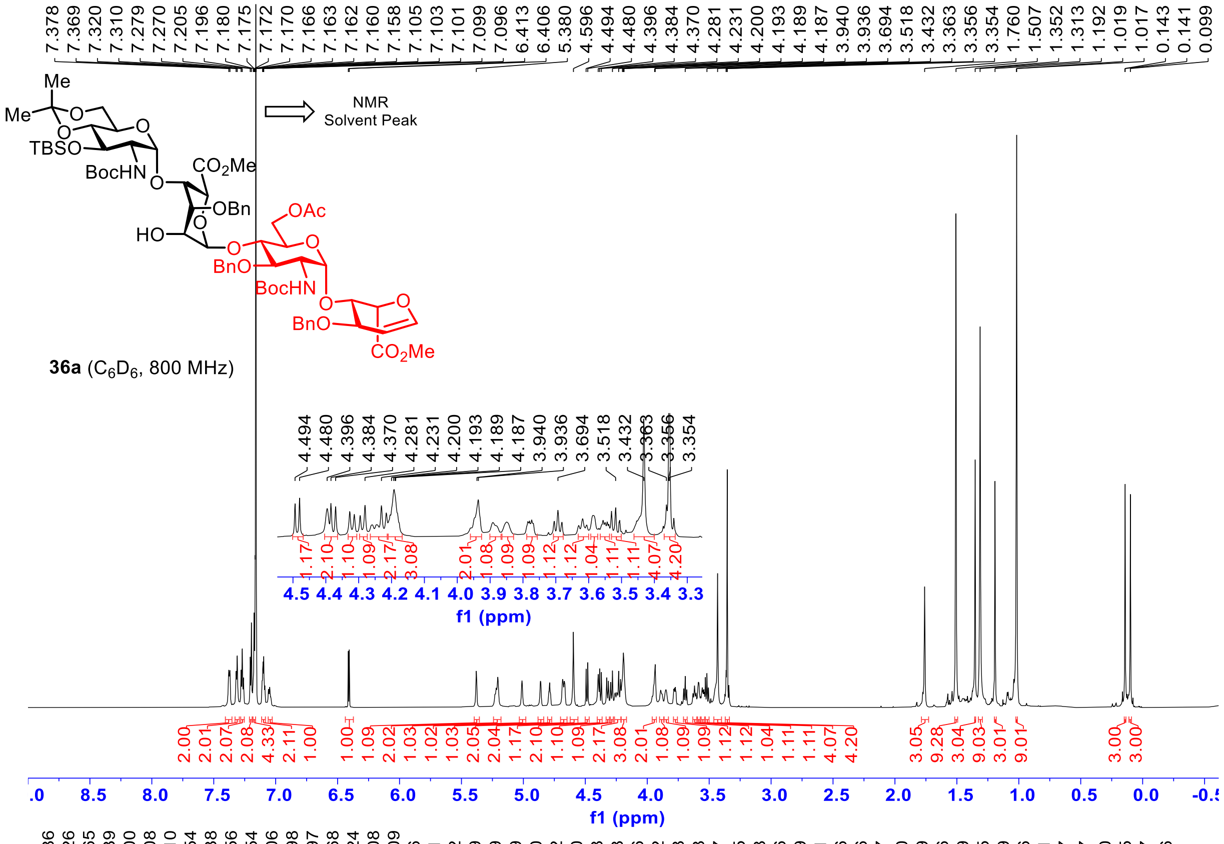
7.378 7.369 7.320 7.310 7.279 7.270 7.205 7.196 7.180 7.175 7.172 7.170 7.166 7.163 7.162 7.160 7.158 7.105 7.103 7.101 7.099 7.096 6.413 6.406 5.380 4.596 4.494 4.480 4.396 4.384 4.370 4.281 4.231 4.200 4.193 4.189 4.187 3.940 3.936 3.694 3.518 3.432 3.363 3.356 3.354 1.760 1.507 1.352 1.313 1.192 1.019 1.017 0.143 0.141 0.099
NMR
Solvent Peak
Me
Me
O
O
O
TBSO
BocHN
O
CO2Me
OBn
OAc
HO
O
O
BnO
BocHN
O
BnO
O
CO2Me
36a (C6D6, 800 MHz)
f1 (ppm)

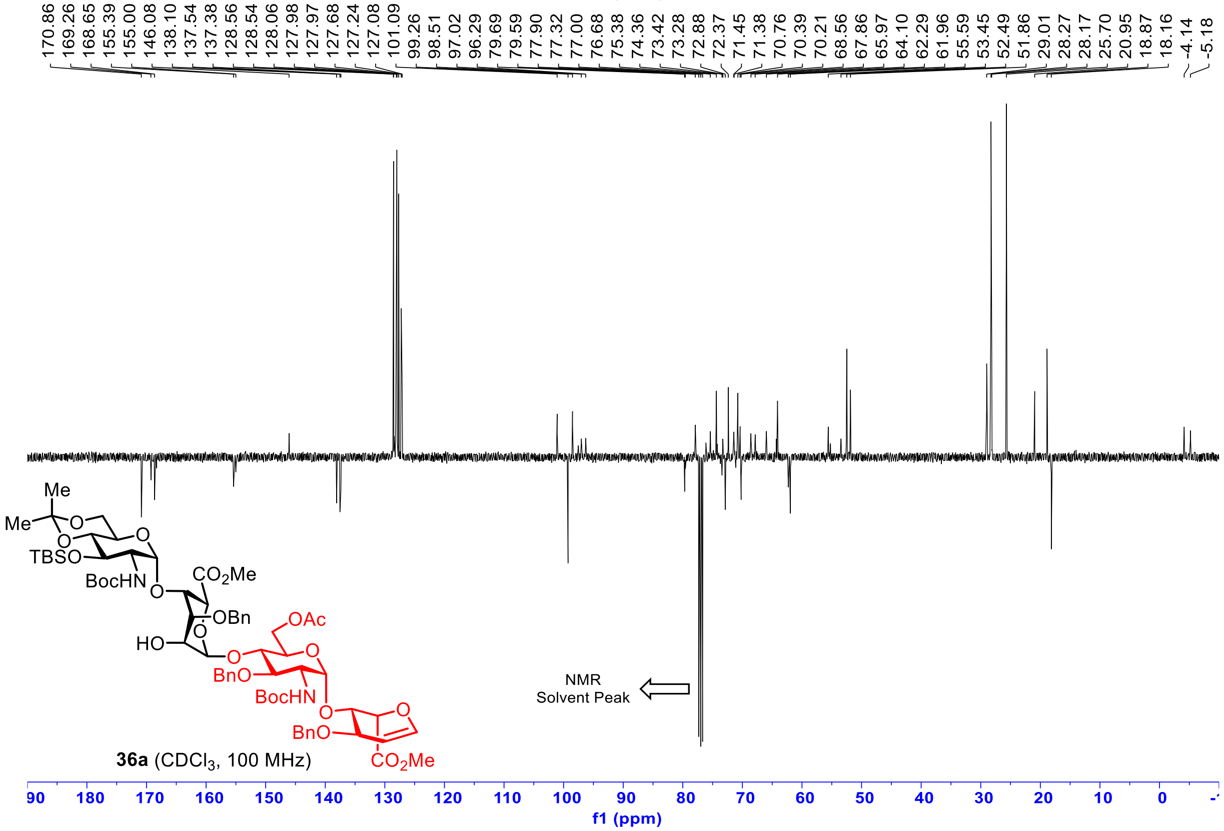
170.86 169.26 168.65 155.39 155.00 146.08 138.10 137.54 137.38 128.56 128.54 128.06 127.98 127.97 127.68 127.24 127.08 101.09 99.26 98.51 97.02 96.29 79.69 79.59 77.90 77.32 77.00 76.68 75.38 74.36 73.42 73.28 72.88 72.37 71.45 71.38 70.76 70.39 70.21 68.56 67.86 65.97 64.10 62.29 61.96 55.59 53.45 52.49 51.86 29.01 28.27 28.17 25.70 20.95 18.87 18.16 -4.14 -5.18
NMR
Solvent Peak
36a (CDCl3, 100 MHz)
f1 (ppm)

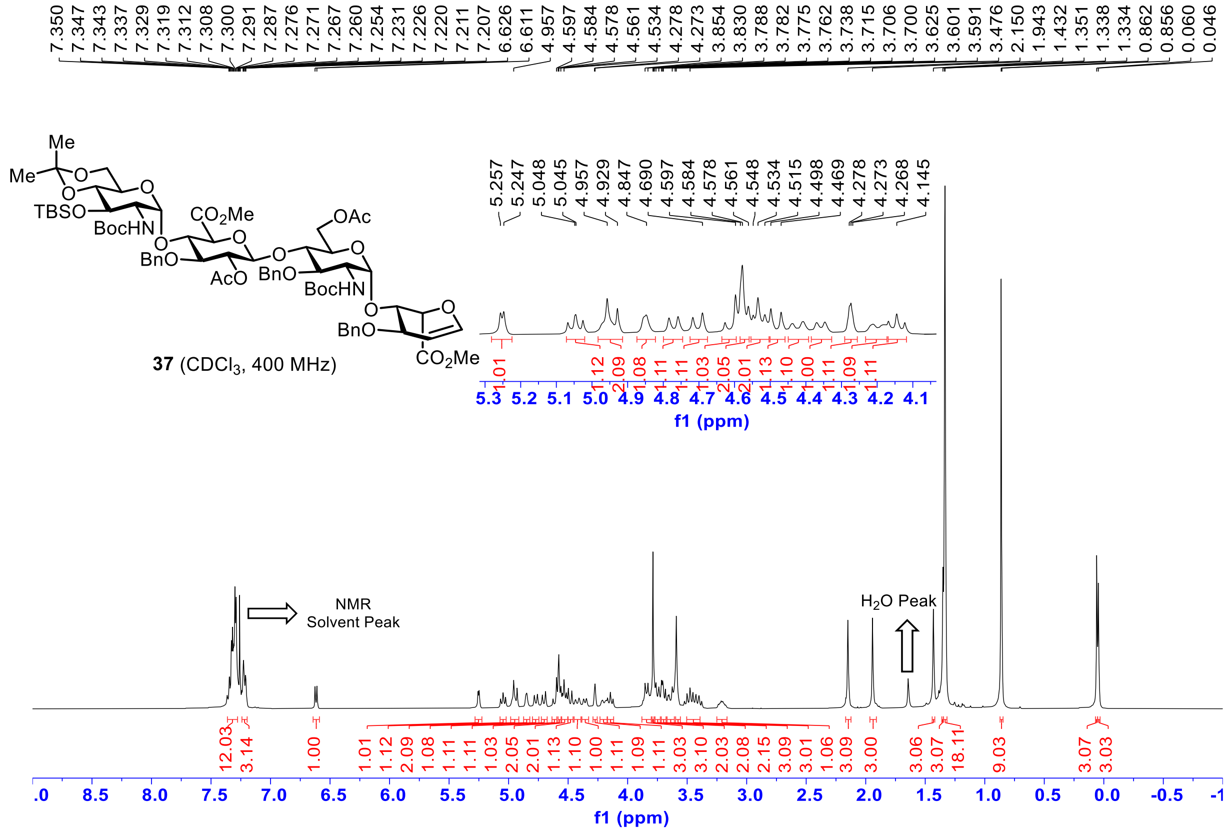
7.350 7.347 7.343 7.337 7.329 7.319 7.312 7.308 7.300 7.291 7.287 7.276 7.271 7.267 7.260 7.254 7.231 7.226 7.220 7.211 7.207 6.626 6.611 4.957 4.597 4.584 4.578 4.561 4.534 4.278 4.273 3.854 3.830 3.788 3.782 3.775 3.762 3.738 3.715 3.706 3.700 3.625 3.601 3.591 3.476 2.150 1.943 1.432 1.351 1.338 1.334 0.862 0.856 0.060 0.046
5.257 5.247 5.045 5.048 4.957 4.929 4.847 4.690 4.597 4.584 4.578 4.561 4.548 4.534 4.515 4.498 4.469 4.278 4.273 4.268 4.145
1.01 1.12 2.09 1.08 1.11 1.11 1.03 2.05 2.01 1.13 1.10 1.00 1.11 1.09 1.11
5.3 5.2 5.1 5.0 4.9 4.8 4.7 4.6 4.5 4.4 4.3 4.2 4.1
f1 (ppm)
Me
Me
O
O
O
TBSO
BocHN
CO2Me
OAc
BnO
AcO
BnO
BocHN
BnO
CO2Me
37 (CDCl3, 400 MHz)
NMR
Solvent Peak
H2O Peak
12.03 3.14 1.00 1.01 1.12 2.09 1.08 1.11 1.11 1.03 2.05 2.01 1.13 1.10 1.00 1.11 1.09 1.11 3.03 3.10 2.03 2.08 2.15 3.09 3.01 1.06 3.09 3.00 3.06 3.07 18.11 9.03 3.07 3.03
.0 8.5 8.0 7.5 7.0 6.5 6.0 5.5 5.0 4.5 4.0 3.5 3.0 2.5 2.0 1.5 1.0 0.5 0.0 -0.5 -1
f1 (ppm)

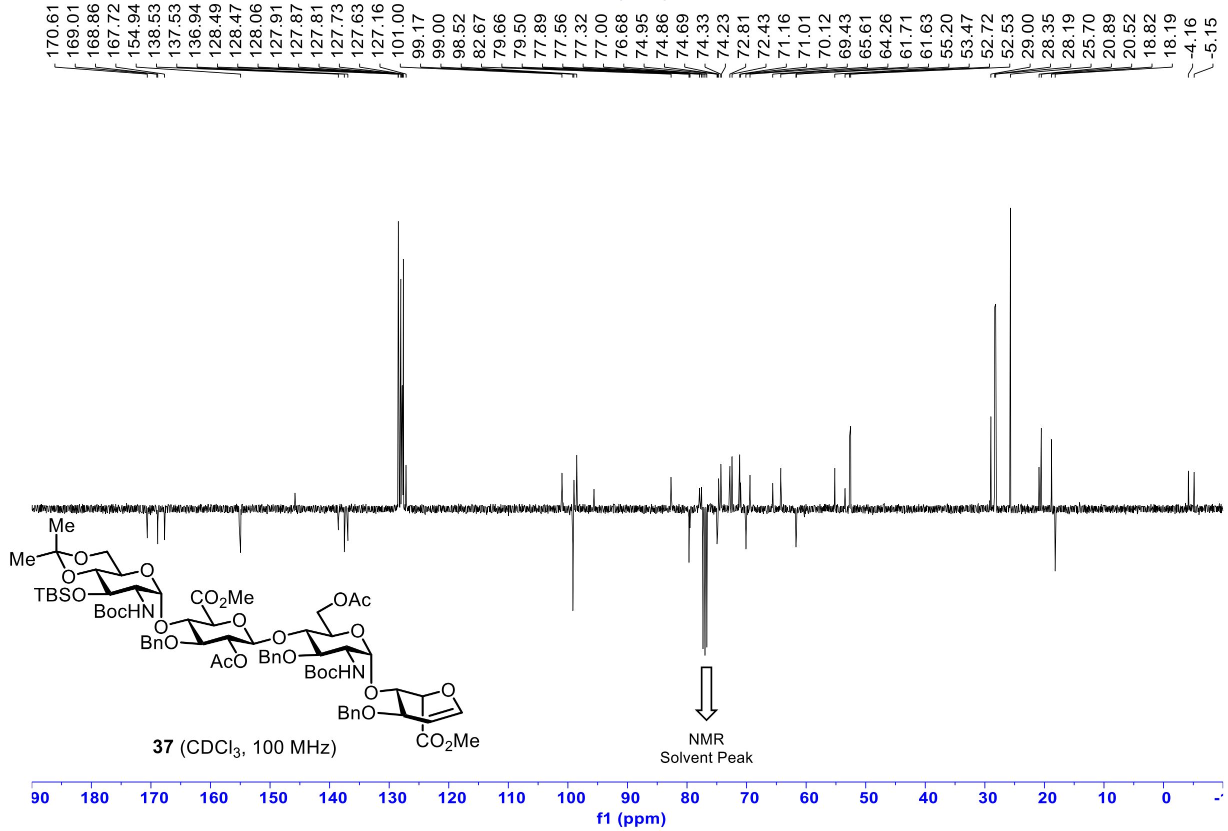
170.61 169.01 168.86 167.72 154.94 138.53 137.53 136.94 128.49 128.47 128.06 127.91 127.87 127.81 127.73 127.63 127.16 101.00 99.17 99.00 98.52 82.67 79.66 79.50 77.89 77.56 77.32 77.00 76.68 74.95 74.86 74.69 74.33 74.23 72.81 72.43 71.16 71.01 70.12 69.43 65.61 64.26 61.71 61.63 55.20 53.47 52.72 52.53 29.00 28.35 28.19 25.70 20.89 20.52 18.82 18.19 -4.16 -5.15
Me
Me
TBSO
BocHN
CO2Me
OAc
BnO
AcO
BnO
BocHN
BnO
CO2Me
37 (CDCl3, 100 MHz)
NMR
Solvent Peak
90 180 170 160 150 140 130 120 110 100 90 80 70 60 50 40 30 20 10 0
f1 (ppm)

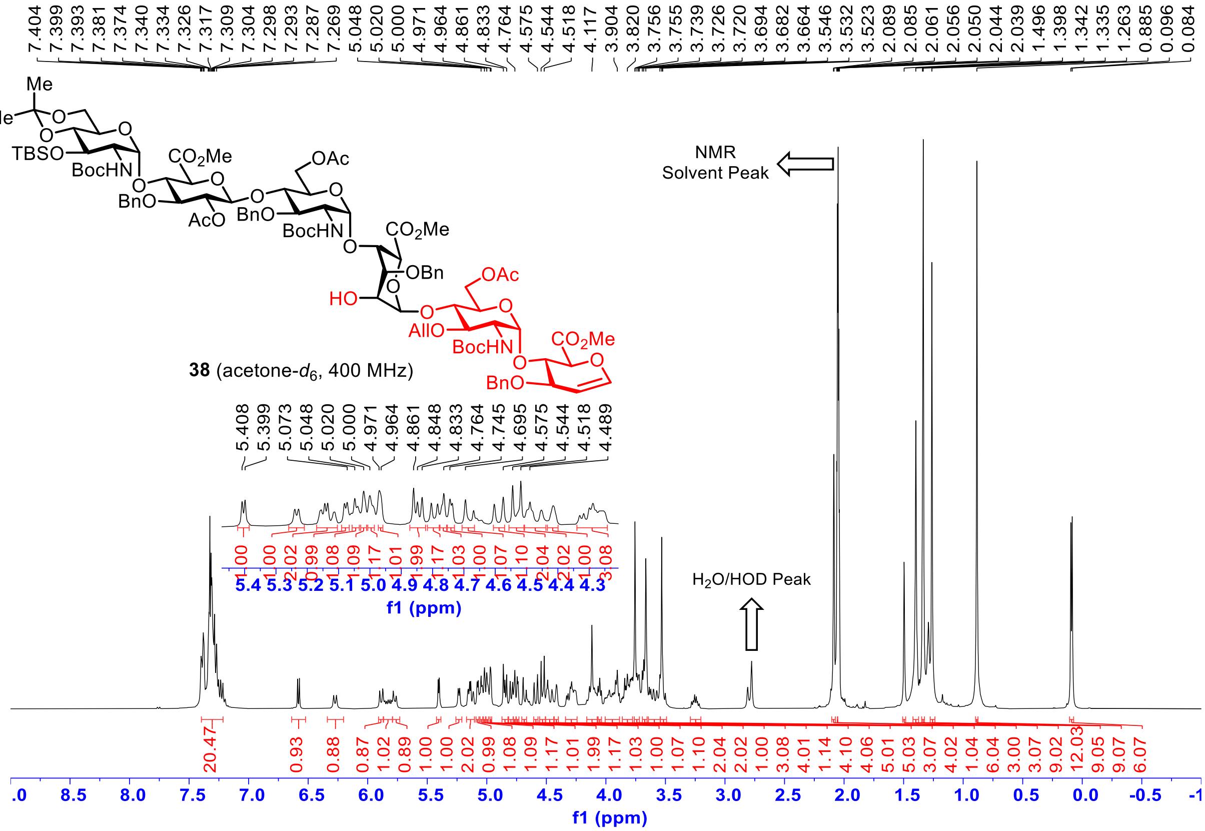

38 (acetone-d_6, 400 MHz)

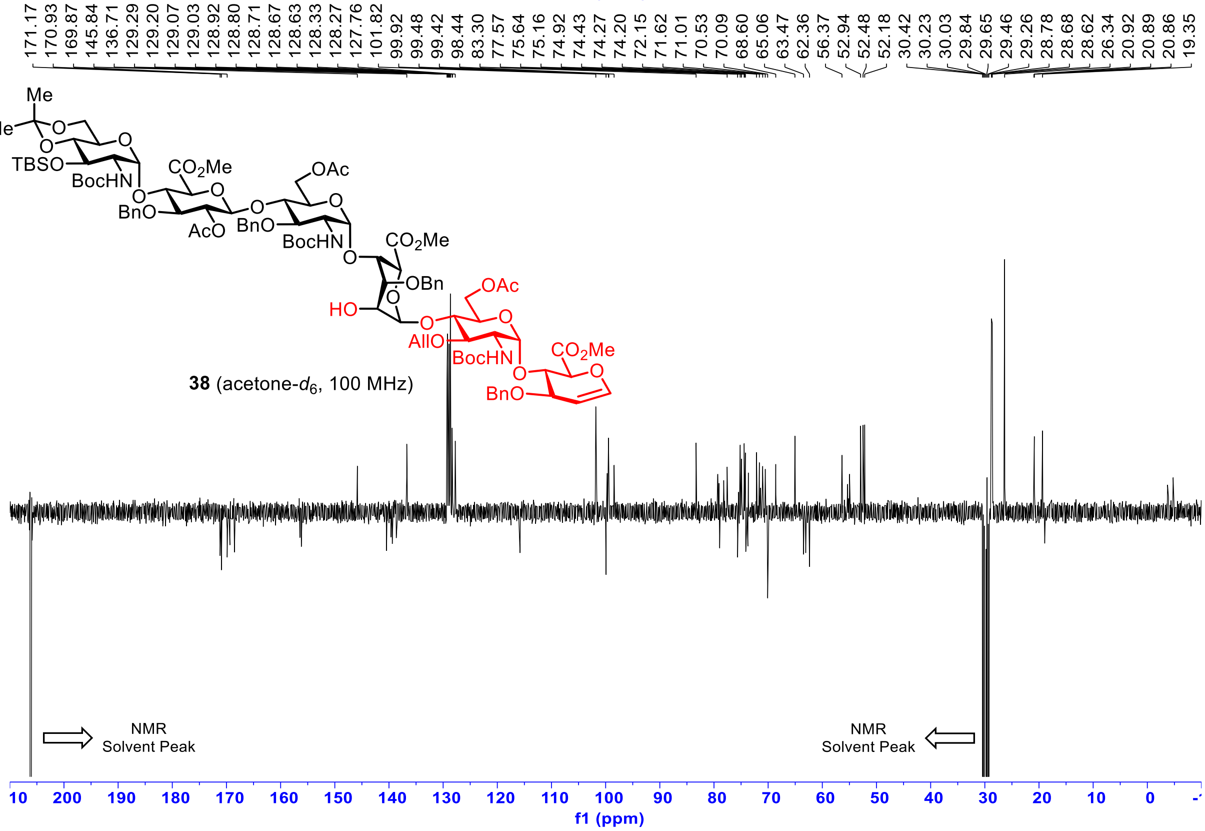

38 (acetone-d_6, 100 MHz)

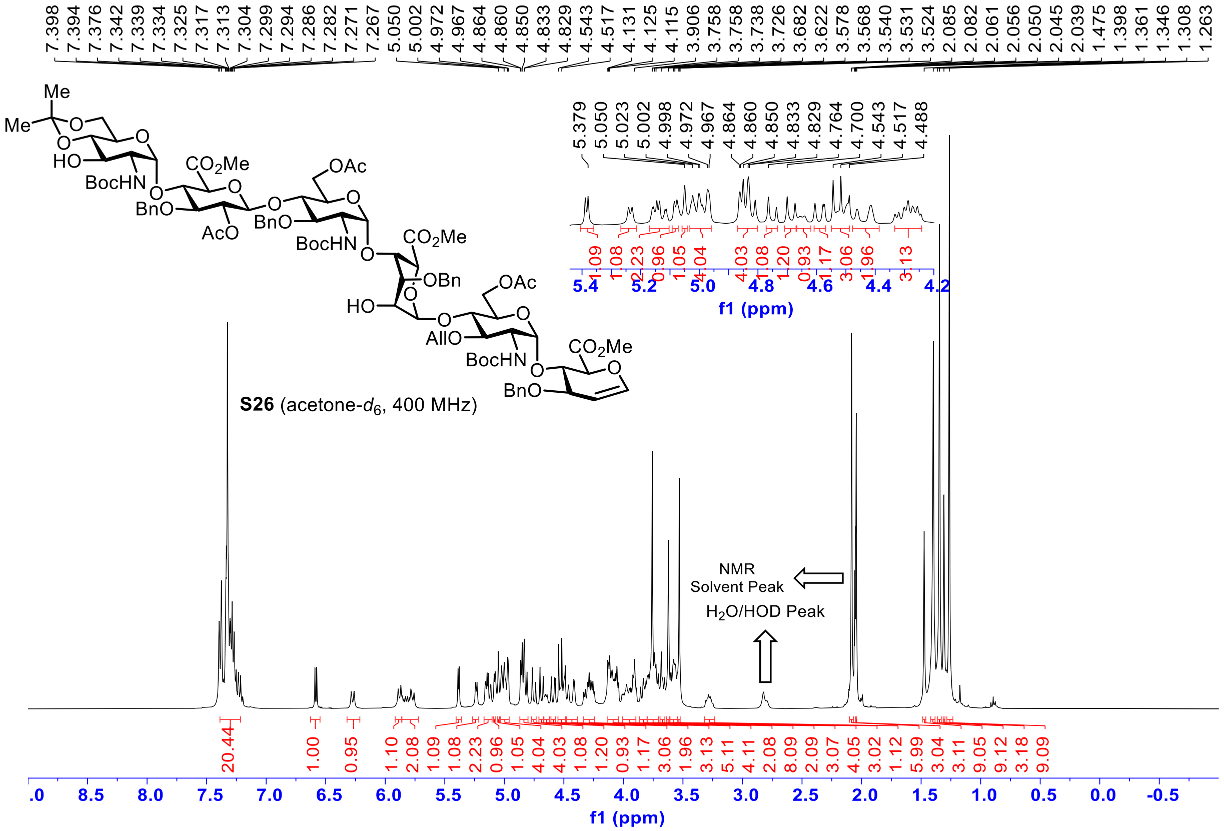
7.398 7.394 7.376 7.342 7.339 7.334 7.325 7.317 7.313 7.304 7.299 7.294 7.286 7.282 7.271 7.267 5.050 5.002 4.972 4.967 4.864 4.860 4.850 4.833 4.829 4.543 4.517 4.131 4.125 4.115 3.906 3.758 3.758 3.738 3.726 3.682 3.622 3.578 3.568 3.540 3.531 3.524 2.085 2.082 2.061 2.056 2.050 2.045 2.039 1.475 1.398 1.361 1.346 1.308 1.263
5.379 5.050 5.023 5.002 4.998 4.972 4.967 4.864 4.860 4.850 4.833 4.829 4.764 4.700 4.543 4.517 4.488
1.09 1.08 2.23 0.96 1.05 4.04 4.03 1.08 1.20 0.93 1.17 3.06 1.96 3.13
5.4 5.2 5.0 4.8 4.6 4.4 4.2
f1 (ppm)
Me
Me
O
O
O
HO
BocHN
O
CO2Me
O
BnO
AcO
O
OAc
O
BnO
BocHN
O
CO2Me
OBn
O
HO
O
OAc
O
AllO
BocHN
O
CO2Me
O
BnO
S26 (acetone-d6, 400 MHz)
NMR
Solvent Peak
H2O/HOD Peak
20.44 1.00 0.95 1.10 2.08 1.09 1.08 2.23 0.96 1.05 4.04 4.03 1.08 1.20 0.93 1.17 3.06 1.96 3.13 5.11 4.11 2.08 8.09 2.09 3.07 4.05 3.02 1.12 5.99 3.04 3.11 9.05 9.12 3.18 9.09
8.5 8.0 7.5 7.0 6.5 6.0 5.5 5.0 4.5 4.0 3.5 3.0 2.5 2.0 1.5 1.0 0.5 0.0 -0.5
f1 (ppm)

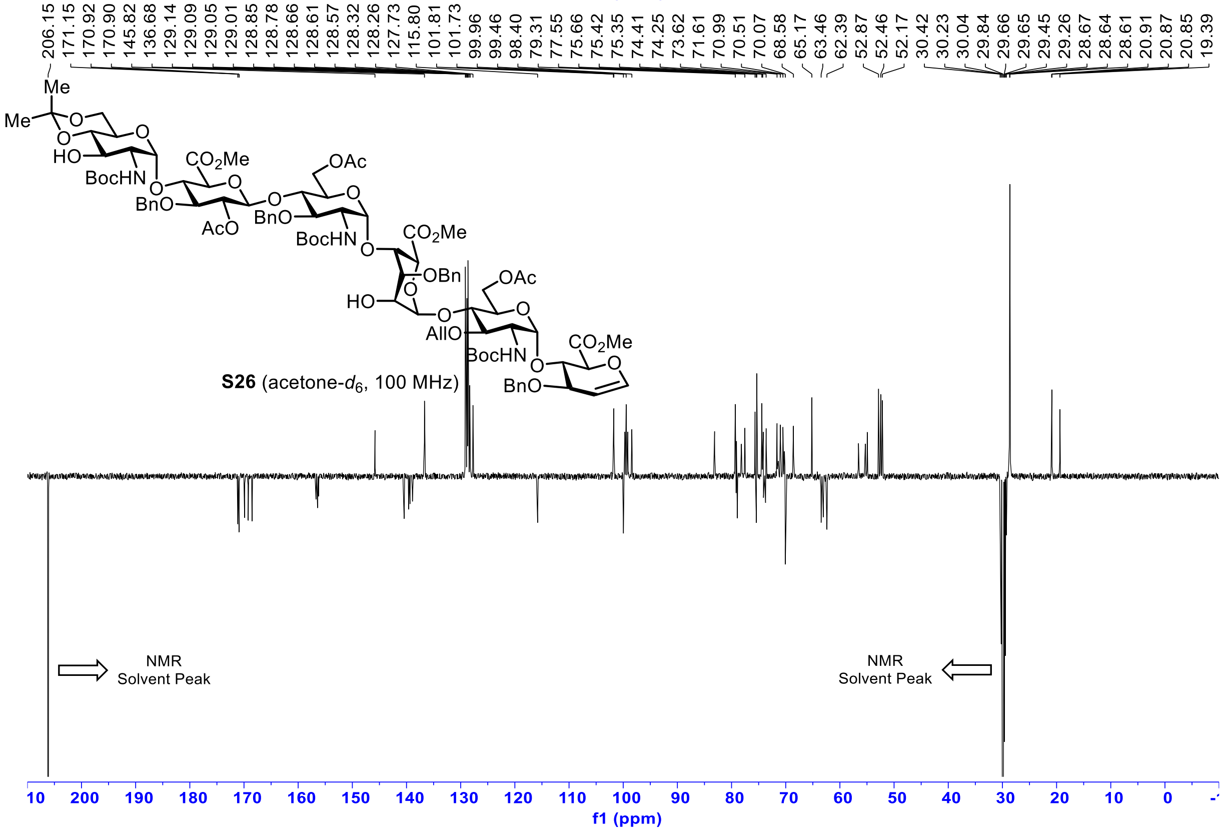
206.15 171.15 170.92 170.90 145.82 136.68 129.14 129.09 129.05 129.01 128.85 128.78 128.66 128.61 128.57 128.32 128.26 127.73 115.80 101.81 101.73 99.96 99.46 98.40 79.31 77.55 75.66 75.42 75.35 74.41 74.25 73.62 71.61 70.99 70.51 70.07 68.58 65.17 63.46 62.39 52.87 52.46 52.17 30.42 30.23 30.04 29.84 29.66 29.65 29.45 29.26 28.67 28.64 28.61 20.91 20.87 20.85 19.39
Me
Me
O
O
O
HO
BocHN
O
CO2Me
O
BnO
AcO
O
OAc
O
BnO
BocHN
O
CO2Me
OBn
O
HO
O
OAc
O
AllO
BocHN
O
CO2Me
O
BnO
S26 (acetone-d6, 100 MHz)
NMR
Solvent Peak
NMR
Solvent Peak
10 200 190 180 170 160 150 140 130 120 110 100 90 80 70 60 50 40 30 20 10 0
f1 (ppm)

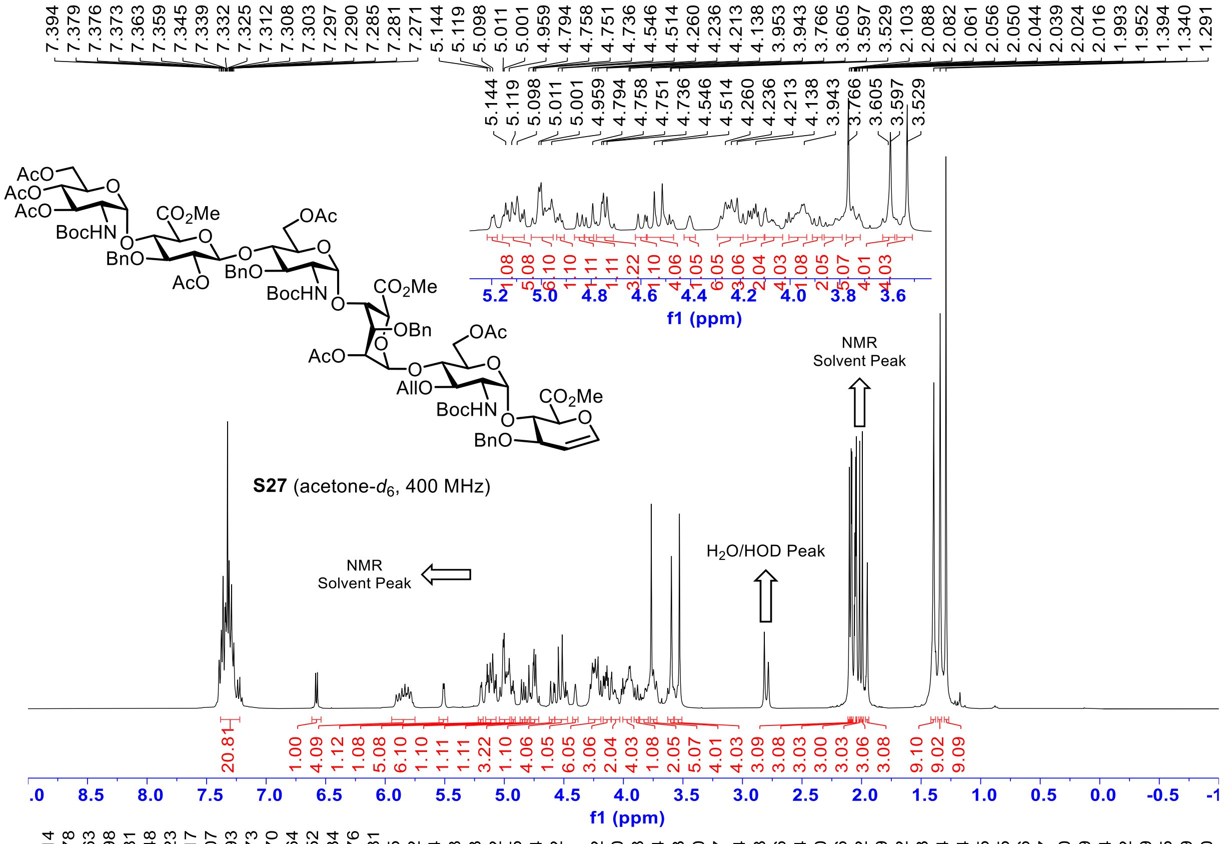
S27 (acetone-d_6, 400 MHz)
NMR Solvent Peak
H2O/HOD Peak
NMR Solvent Peak
f1 (ppm)

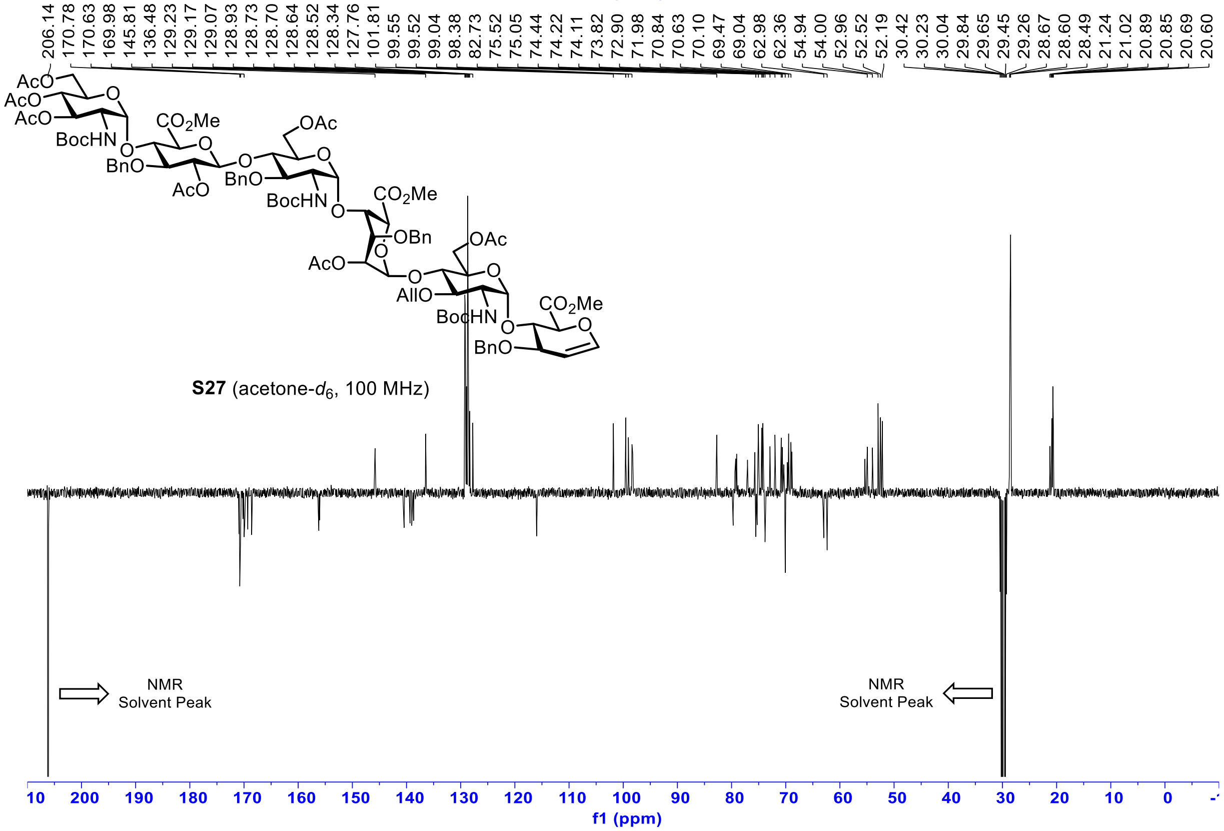
S27 (acetone-d_6, 100 MHz)
NMR Solvent Peak
NMR Solvent Peak
f1 (ppm)

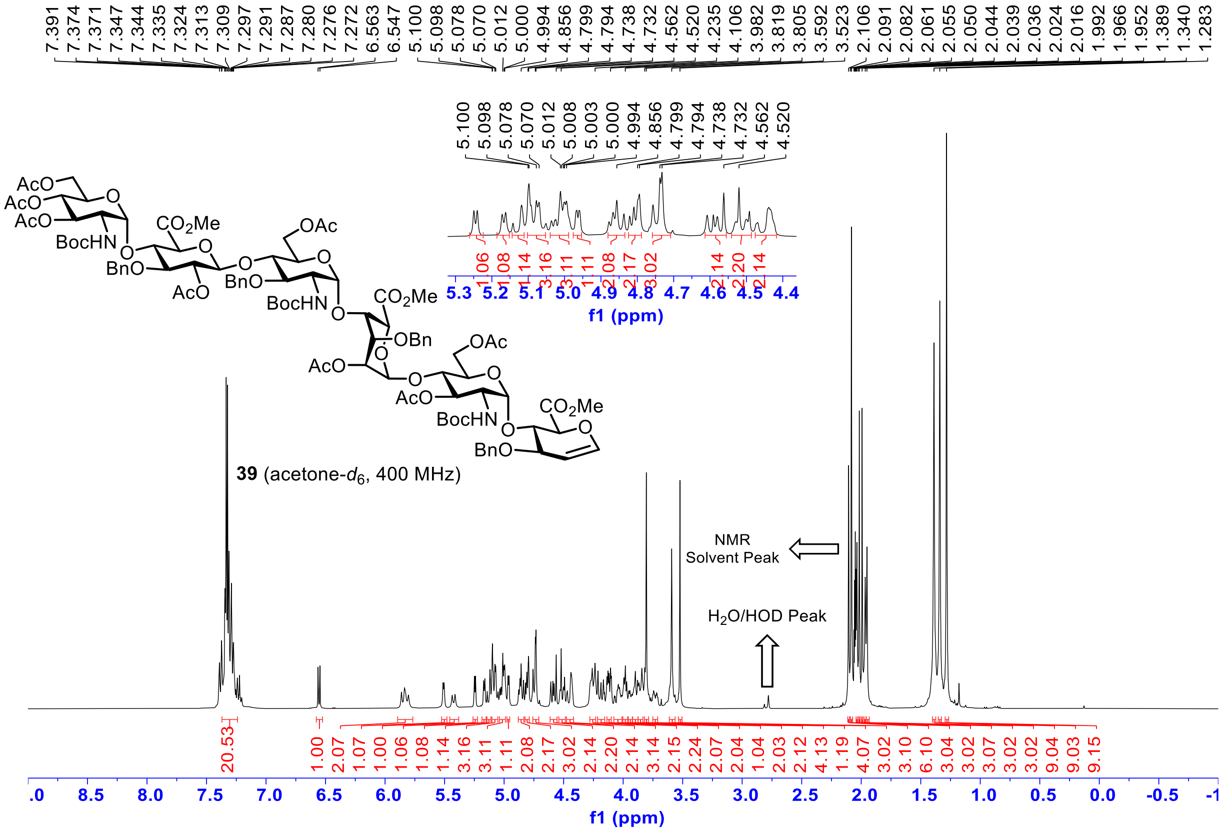

39 (acetone-d_6, 400 MHz)

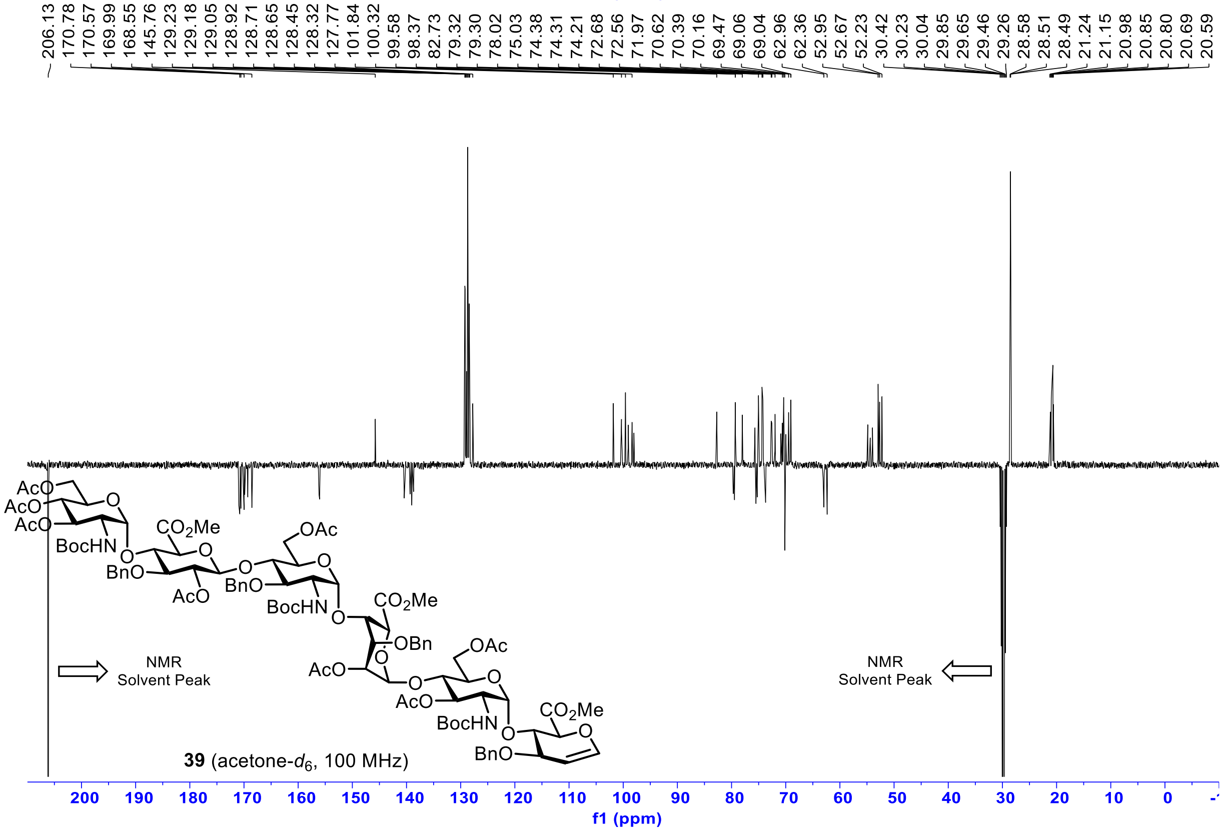

39 (acetone-d_6, 100 MHz)

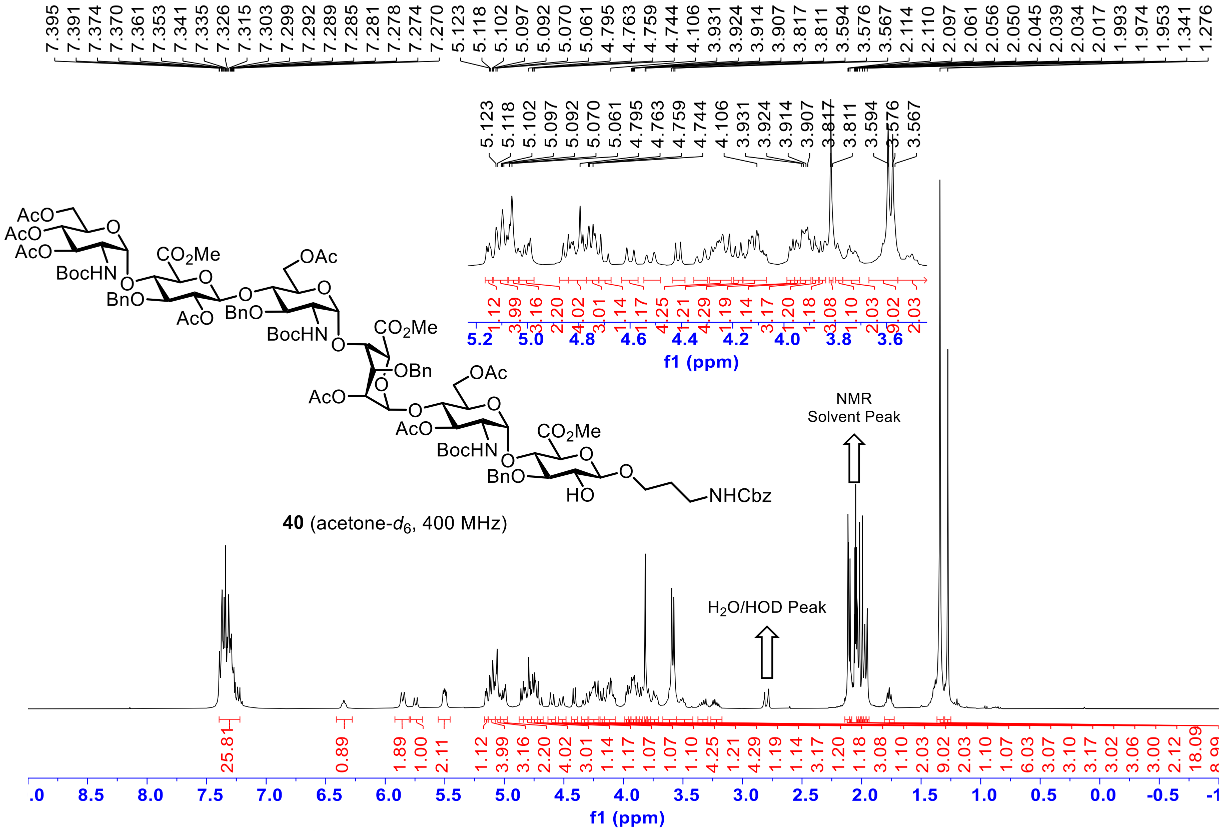
7.395 7.391 7.374 7.370 7.361 7.353 7.341 7.335 7.326 7.315 7.303 7.299 7.292 7.289 7.285 7.281 7.278 7.274 7.270 5.123 5.118 5.102 5.097 5.092 5.070 5.061 4.795 4.763 4.759 4.744 4.106 3.931 3.924 3.914 3.907 3.817 3.811 3.594 3.576 3.567 2.114 2.110 2.097 2.061 2.056 2.050 2.045 2.039 2.034 2.017 1.993 1.974 1.953 1.341 1.276
40 (acetone-d_6, 400 MHz)
NMR
Solvent Peak
H_2O/HOD Peak
f1 (ppm)

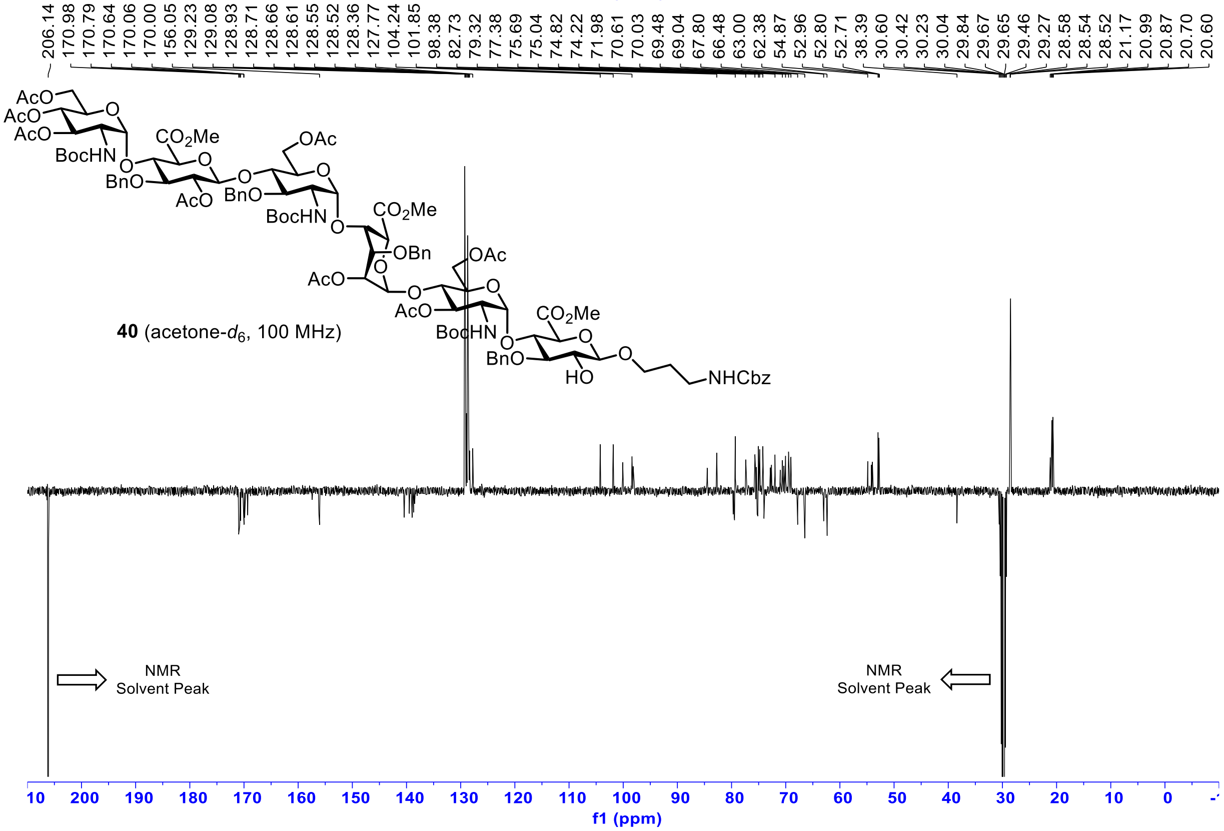
206.14 170.98 170.79 170.64 170.06 170.00 156.05 129.23 129.08 128.93 128.71 128.66 128.61 128.55 128.52 128.36 127.77 104.24 101.85 98.38 82.73 79.32 77.38 75.69 75.04 74.82 74.22 71.98 70.61 70.03 69.48 69.04 67.80 66.48 63.00 62.38 54.87 52.96 52.80 52.71 38.39 30.60 30.42 30.23 30.04 29.84 29.67 29.65 29.46 29.27 28.58 28.54 28.52 21.17 20.99 20.87 20.70 20.60
40 (acetone-d_6, 100 MHz)
NMR
Solvent Peak
f1 (ppm)

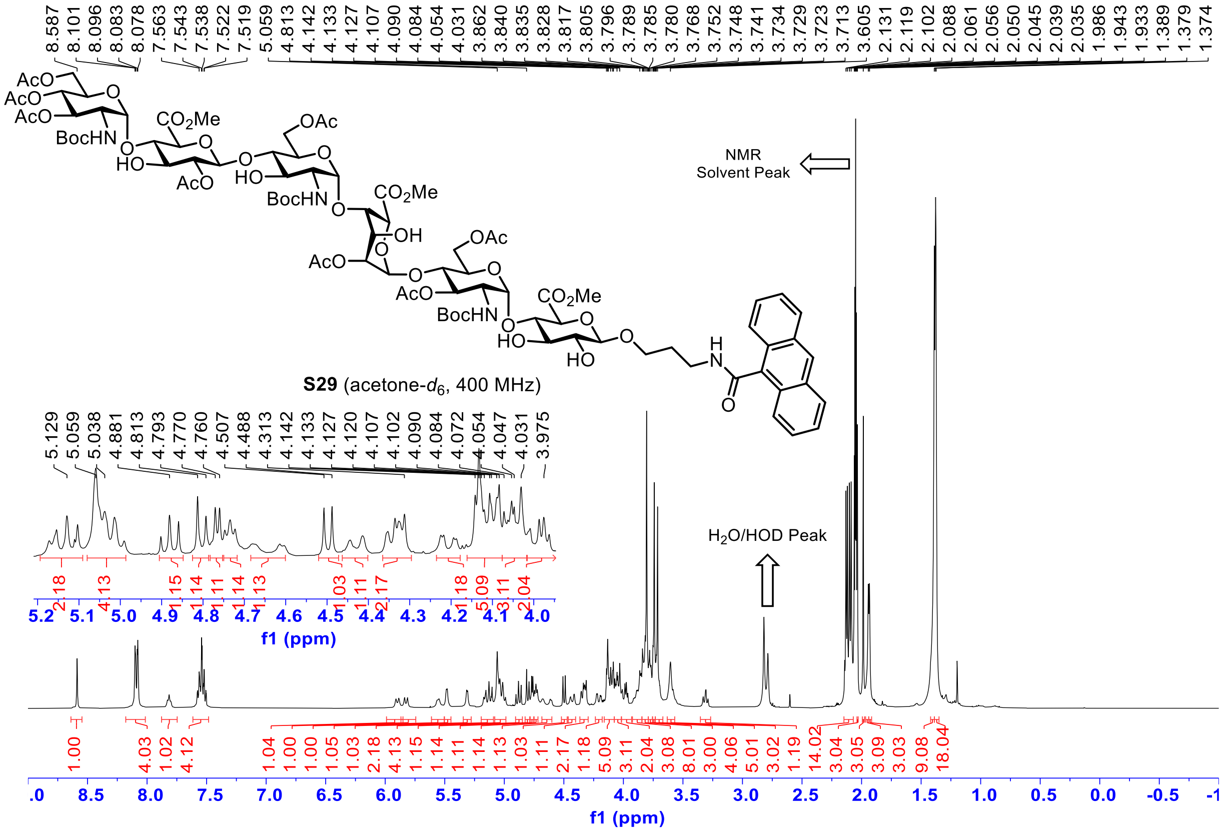
8.587 8.101 8.096 8.083 8.078 7.563 7.543 7.538 7.522 7.519 5.059 4.813 4.142 4.133 4.127 4.107 4.090 4.084 4.054 4.031 3.862 3.840 3.835 3.828 3.817 3.805 3.796 3.789 3.785 3.780 3.768 3.752 3.748 3.741 3.734 3.729 3.723 3.713 3.605 2.131 2.119 2.102 2.088 2.061 2.056 2.050 2.045 2.039 2.035 1.986 1.943 1.933 1.389 1.379 1.374
NMR Solvent Peak
H2O/HOD Peak
S29 (acetone-*d*6, 400 MHz)
f1 (ppm)

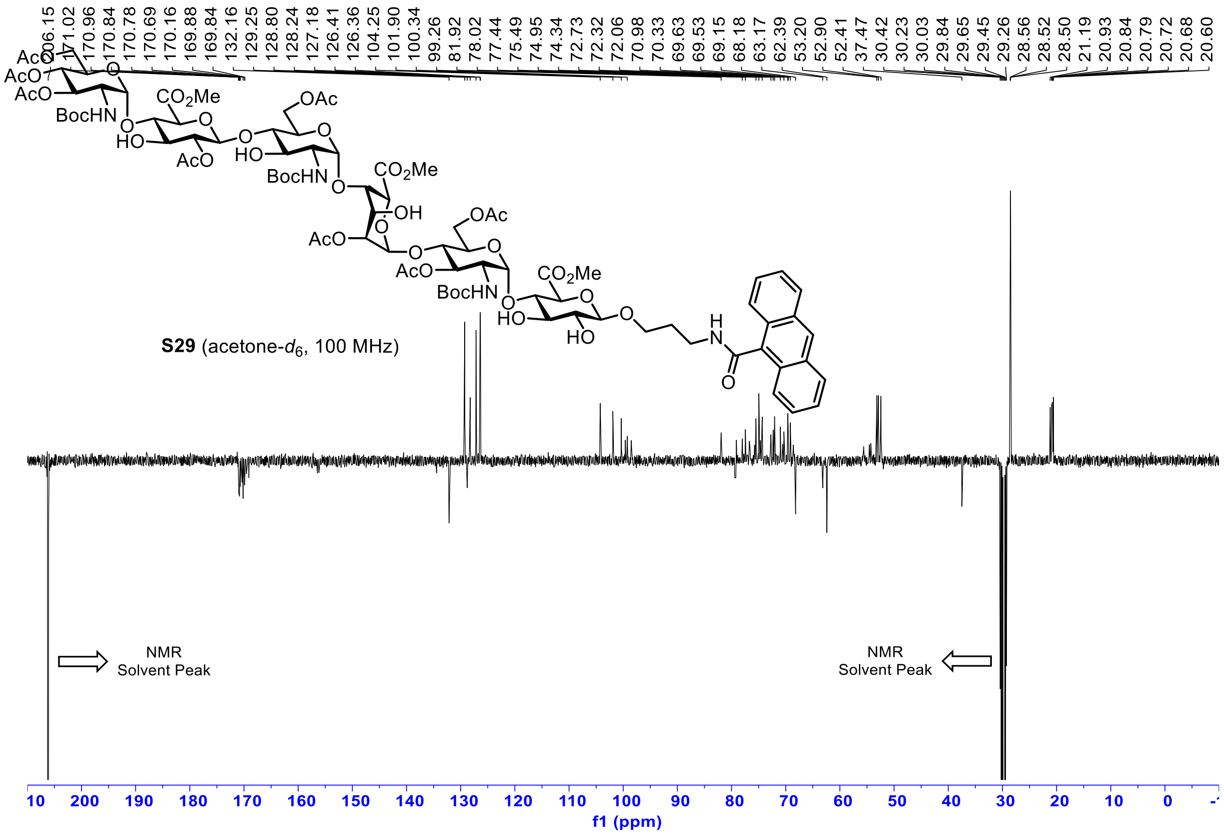
206.15 171.02 170.96 170.84 170.78 170.69 170.16 169.88 169.84 132.16 129.25 128.80 128.24 127.18 126.41 126.36 104.25 101.90 100.34 99.26 81.92 78.02 77.44 75.49 74.95 74.34 72.73 72.32 72.06 70.98 70.33 69.63 69.53 69.15 68.18 63.17 62.39 53.20 52.90 52.41 37.47 30.42 30.23 30.03 29.84 29.65 29.45 29.26 28.56 28.52 28.50 21.19 20.93 20.84 20.79 20.72 20.68 20.60
S29 (acetone-*d*6, 100 MHz)
NMR Solvent Peak
NMR Solvent Peak
f1 (ppm)

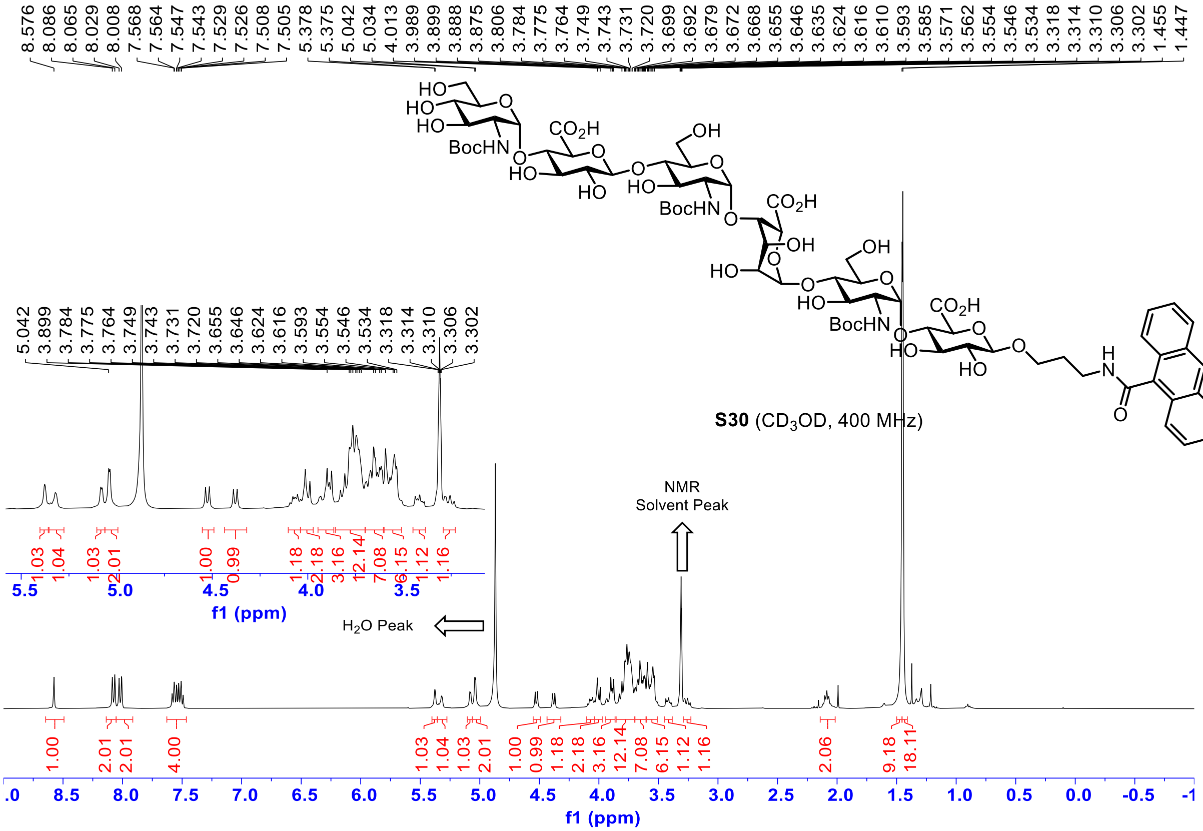
8.576 8.086 8.065 8.029 8.008 7.568 7.564 7.547 7.543 7.529 7.526 7.508 7.505 5.378 5.375 5.042 5.034 4.013 3.989 3.899 3.888 3.875 3.806 3.784 3.775 3.764 3.749 3.743 3.731 3.720 3.699 3.692 3.679 3.672 3.668 3.655 3.646 3.635 3.624 3.616 3.610 3.593 3.585 3.571 3.562 3.554 3.546 3.534 3.318 3.314 3.310 3.306 3.302 1.455 1.447
5.042 3.899 3.784 3.775 3.764 3.749 3.743 3.731 3.720 3.655 3.646 3.624 3.616 3.593 3.554 3.546 3.534 3.318 3.314 3.310 3.306 3.302
S30 (CD_3OD, 400 MHz)
NMR
Solvent Peak
H_2O Peak
1.03 1.04 1.03 2.01 1.00 0.99 1.18 2.18 3.16 12.14 7.08 6.15 1.12 1.16
1.00 2.01 2.01 4.00 1.03 1.04 1.03 2.01 1.00 0.99 1.18 2.18 3.16 12.14 7.08 6.15 1.12 1.16 2.06 9.18 18.11
f1 (ppm)

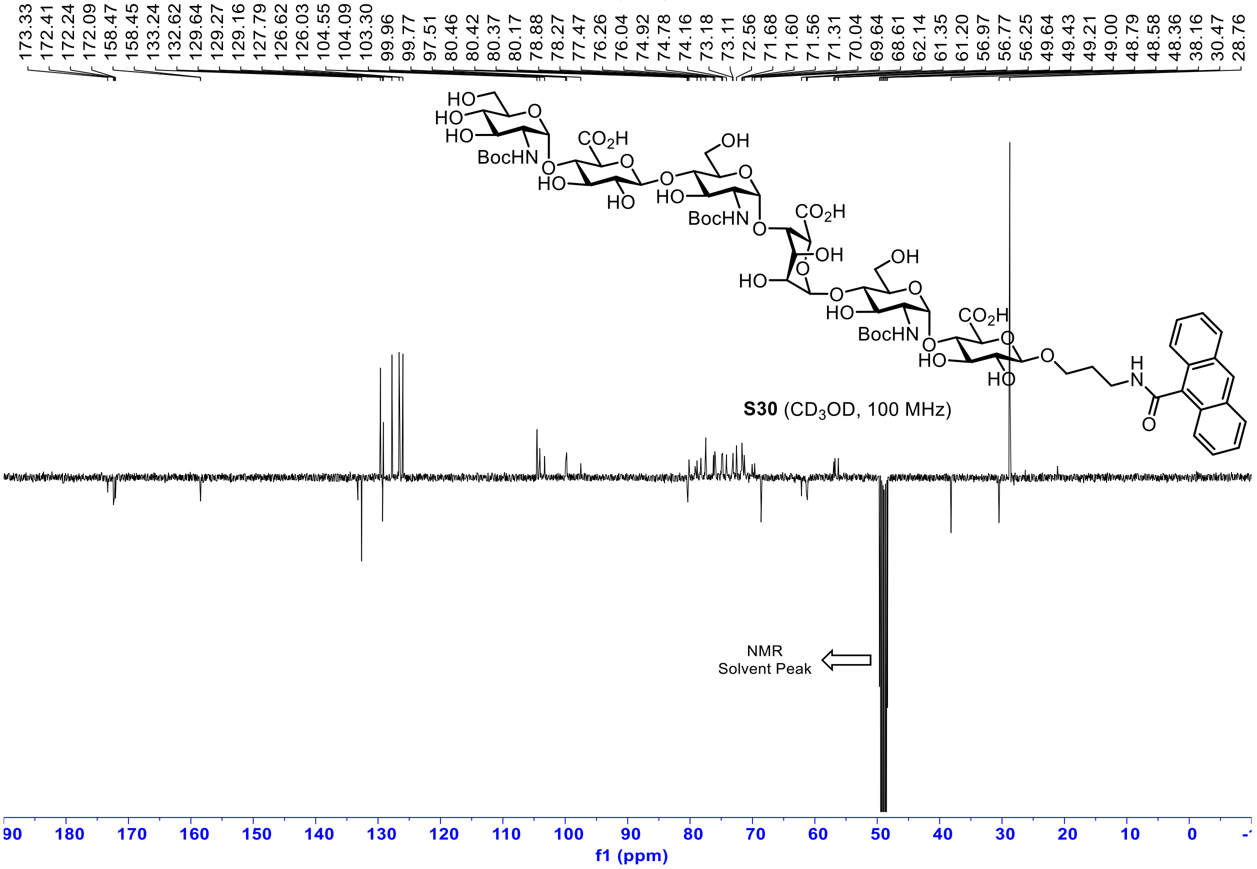
173.33 172.41 172.24 172.09 158.47 158.45 133.24 132.62 129.64 129.27 129.16 127.79 126.62 126.03 104.55 104.09 103.30 99.96 99.77 97.51 80.46 80.42 80.37 80.17 78.88 78.27 77.47 76.26 76.04 74.92 74.78 74.16 73.18 73.11 72.56 71.68 71.60 71.56 71.31 70.04 69.64 68.61 62.14 61.35 61.20 56.97 56.77 56.25 49.64 49.43 49.21 49.00 48.79 48.58 48.36 38.16 30.47 28.76
S30 (CD_3OD, 100 MHz)
NMR
Solvent Peak
f1 (ppm)

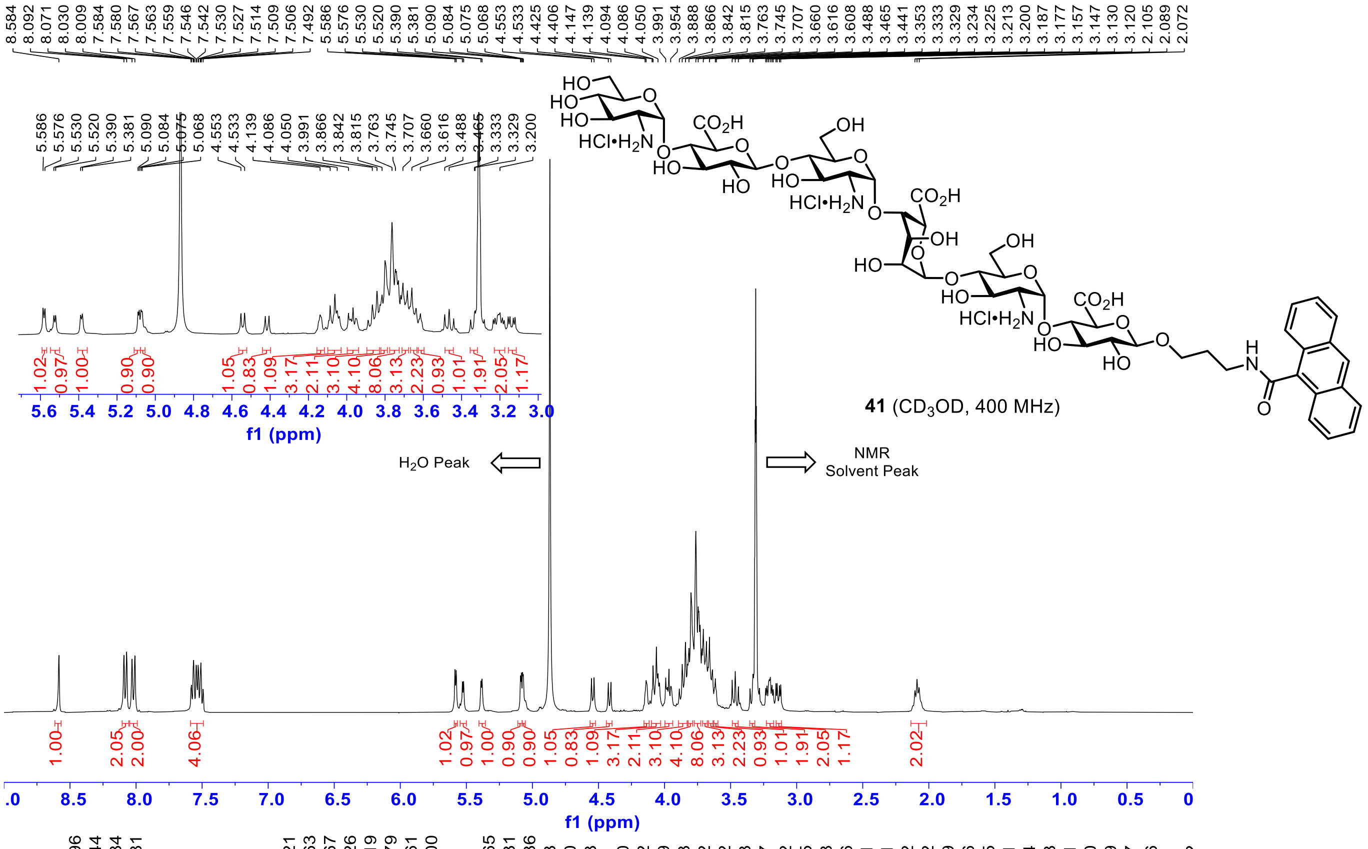
H$_2$O Peak
NMR Solvent Peak
41 (CD$_3$OD, 400 MHz)
f1 (ppm)

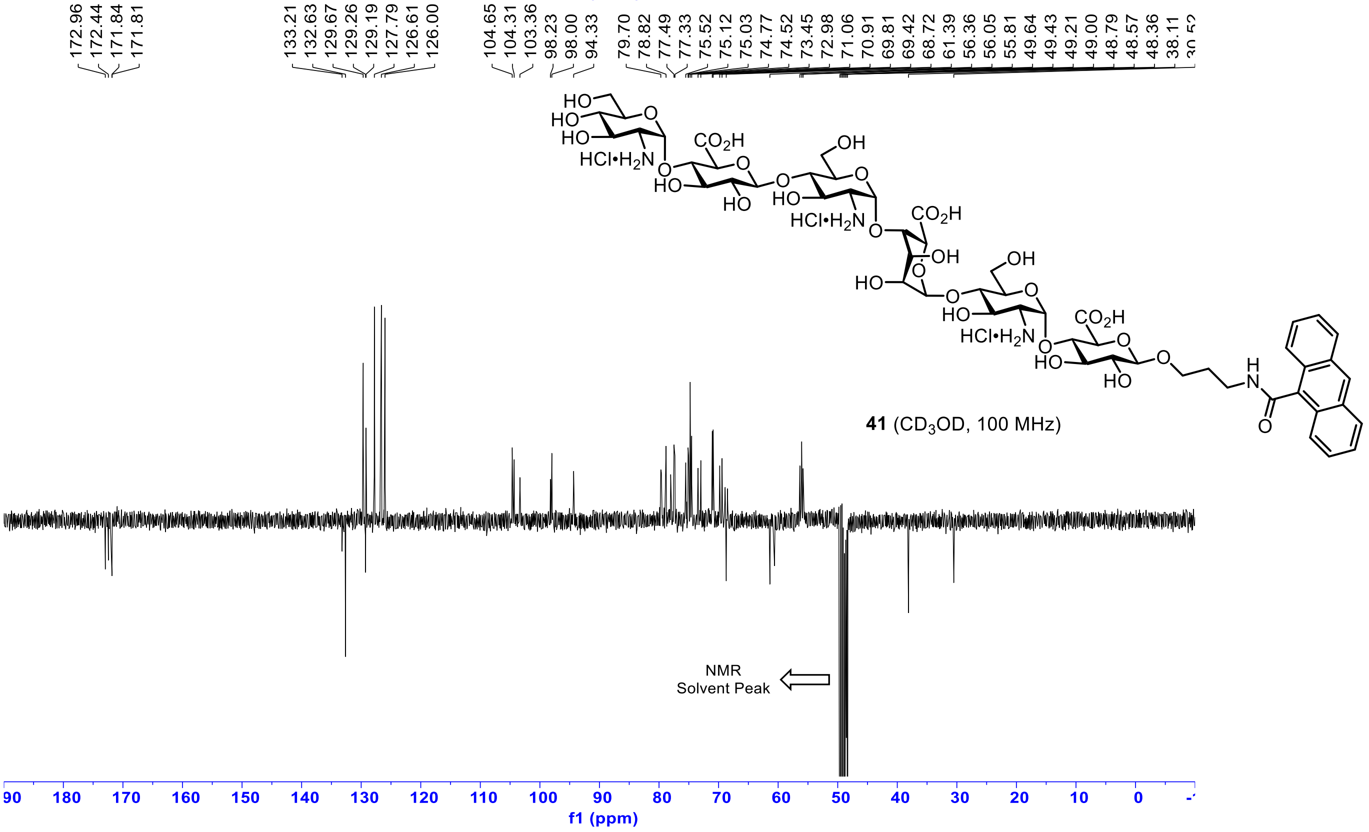
NMR Solvent Peak
41 (CD$_3$OD, 100 MHz)
f1 (ppm)

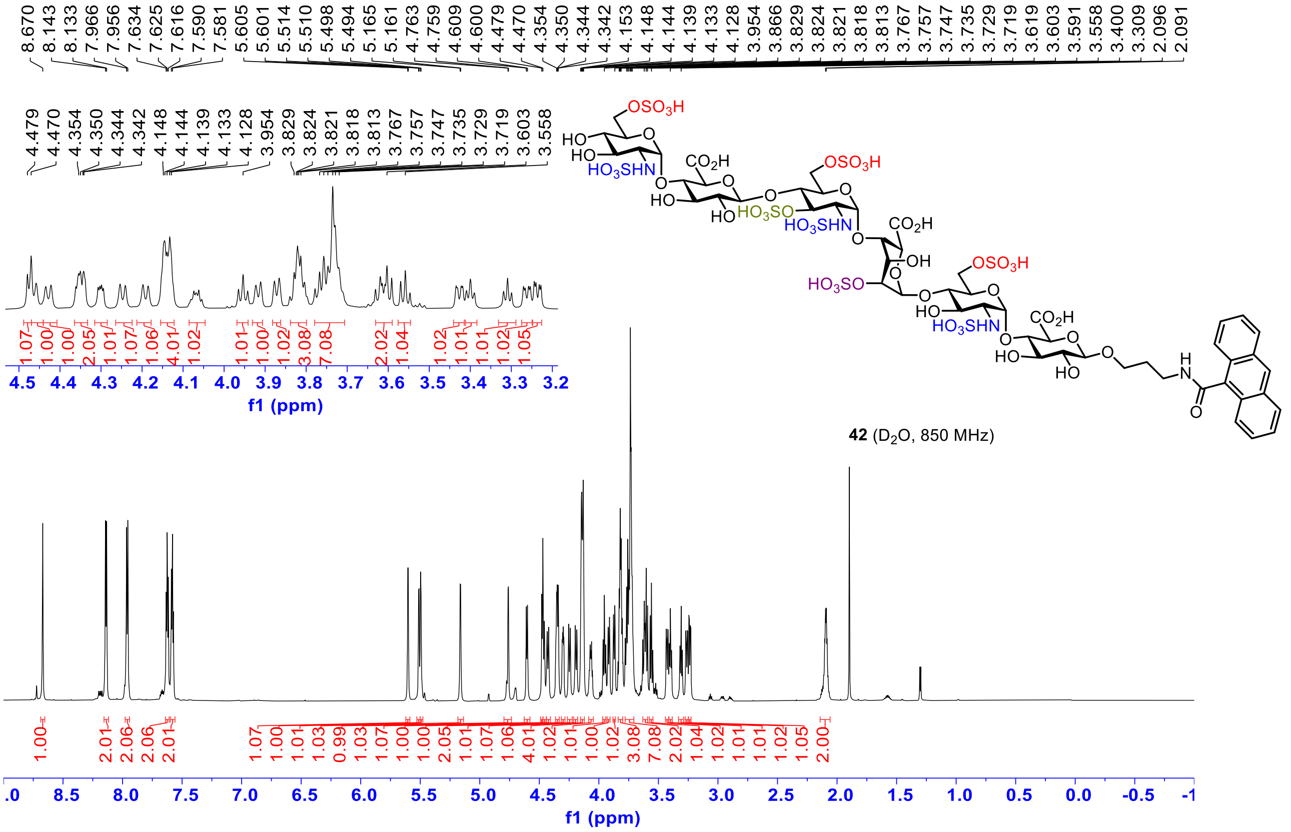

42 (D_2O, 850 MHz)

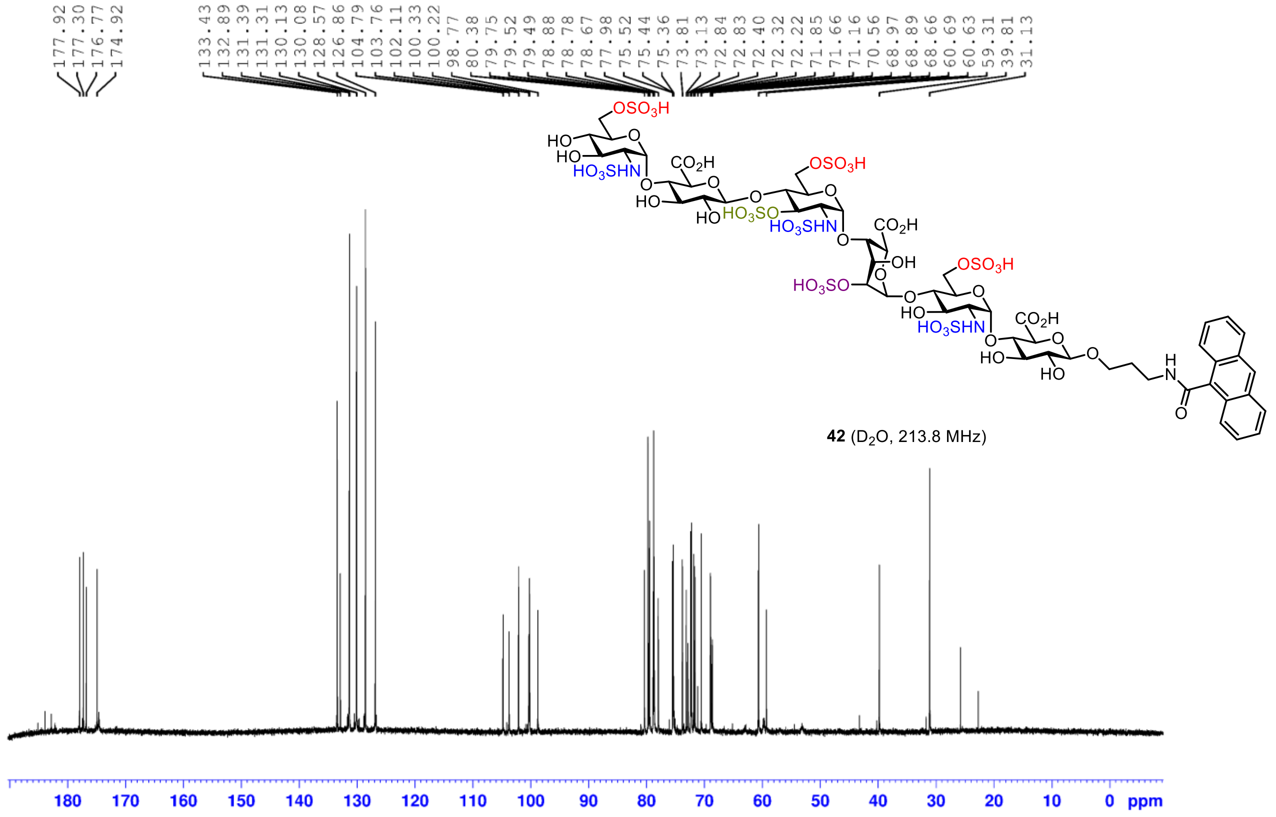

42 (D_2O, 213.8 MHz)

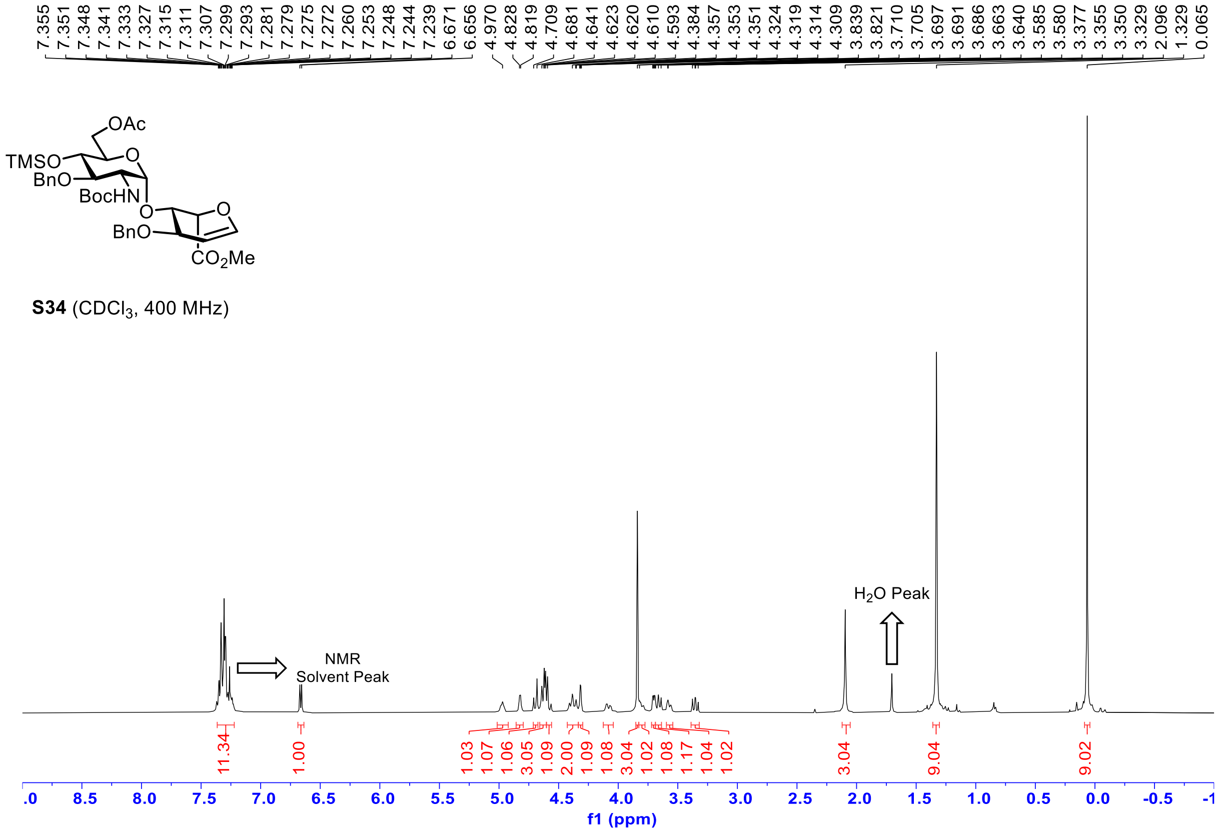
7.355
7.351
7.348
7.341
7.333
7.327
7.315
7.311
7.307
7.299
7.293
7.281
7.279
7.275
7.272
7.260
7.253
7.248
7.244
7.239
6.671
6.656
4.970
4.828
4.819
4.709
4.681
4.641
4.623
4.620
4.610
4.593
4.384
4.357
4.353
4.351
4.324
4.319
4.314
4.309
3.839
3.821
3.710
3.705
3.697
3.691
3.686
3.663
3.640
3.585
3.580
3.377
3.355
3.350
3.329
2.096
1.329
0.065
OAc
TMSO
O
BnO
BocHN
O
O
BnO
CO_2Me
S34 ($CDCl_3$, 400 MHz)
H_2O Peak
NMR
Solvent Peak
11.34
1.00
1.03
1.07
1.06
3.05
1.09
2.00
1.09
1.08
3.04
1.02
1.08
1.17
1.04
1.02
3.04
9.04
9.02
.0
8.5
8.0
7.5
7.0
6.5
6.0
5.5
5.0
4.5
4.0
3.5
3.0
2.5
2.0
1.5
1.0
0.5
0.0
-0.5
-1
f1 (ppm)

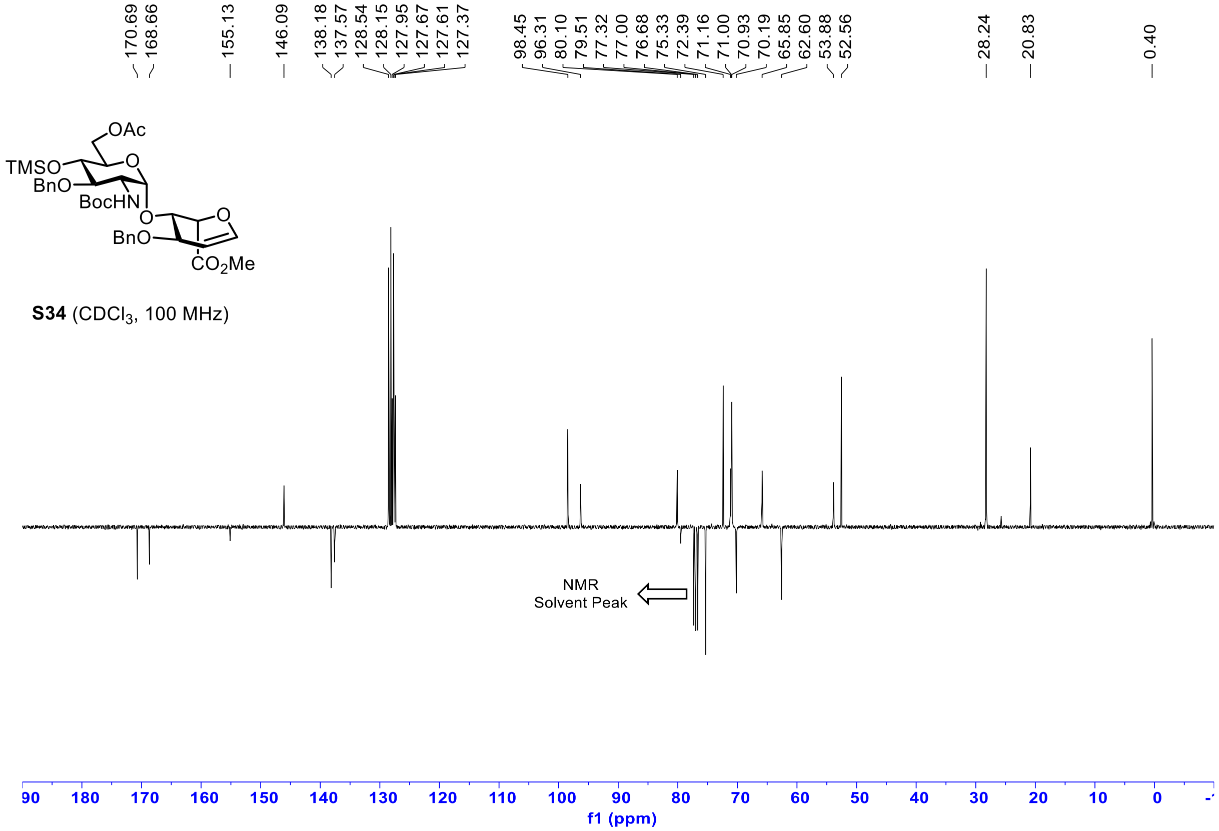
170.69
168.66
155.13
146.09
138.18
137.57
128.54
128.15
127.95
127.67
127.61
127.37
98.45
96.31
80.10
79.51
77.32
77.00
76.68
75.33
72.39
71.16
71.00
70.93
70.19
65.85
62.60
53.88
52.56
28.24
20.83
0.40
OAc
TMSO
O
BnO
BocHN
O
O
BnO
CO_2Me
S34 ($CDCl_3$, 100 MHz)
NMR
Solvent Peak
90
180
170
160
150
140
130
120
110
100
90
80
70
60
50
40
30
20
10
0
f1 (ppm)

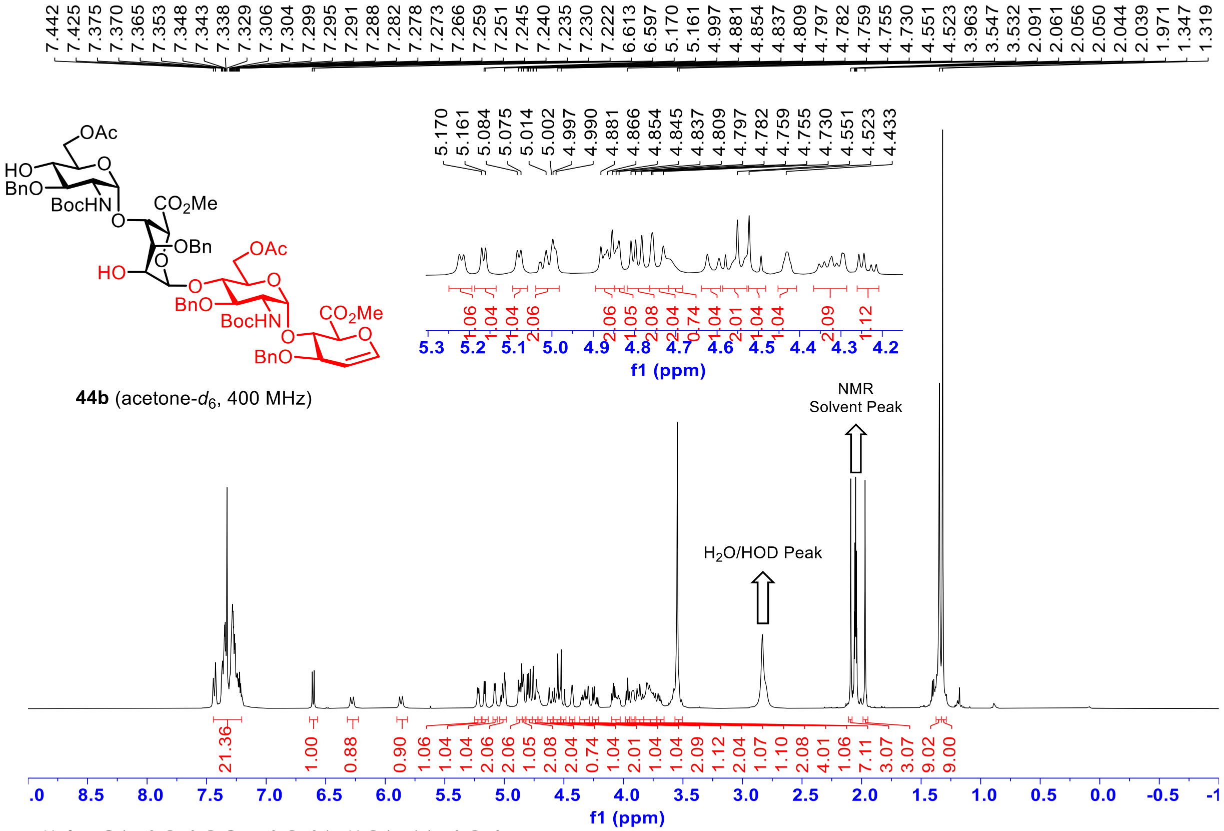
7.442 7.425 7.375 7.370 7.365 7.353 7.348 7.343 7.338 7.329 7.306 7.304 7.299 7.295 7.291 7.288 7.282 7.278 7.273 7.266 7.259 7.251 7.245 7.240 7.235 7.230 7.222 6.613 6.597 5.170 5.161 4.997 4.881 4.854 4.837 4.809 4.797 4.782 4.759 4.755 4.730 4.551 4.523 3.963 3.547 3.532 2.091 2.061 2.056 2.050 2.044 2.039 1.971 1.347 1.319
5.170 5.161 5.084 5.075 5.014 5.002 4.997 4.990 4.881 4.866 4.854 4.845 4.837 4.809 4.797 4.782 4.759 4.755 4.730 4.551 4.523 4.433
44b (acetone-d_6, 400 MHz)
NMR Solvent Peak
H_2O/HOD Peak
f1 (ppm)
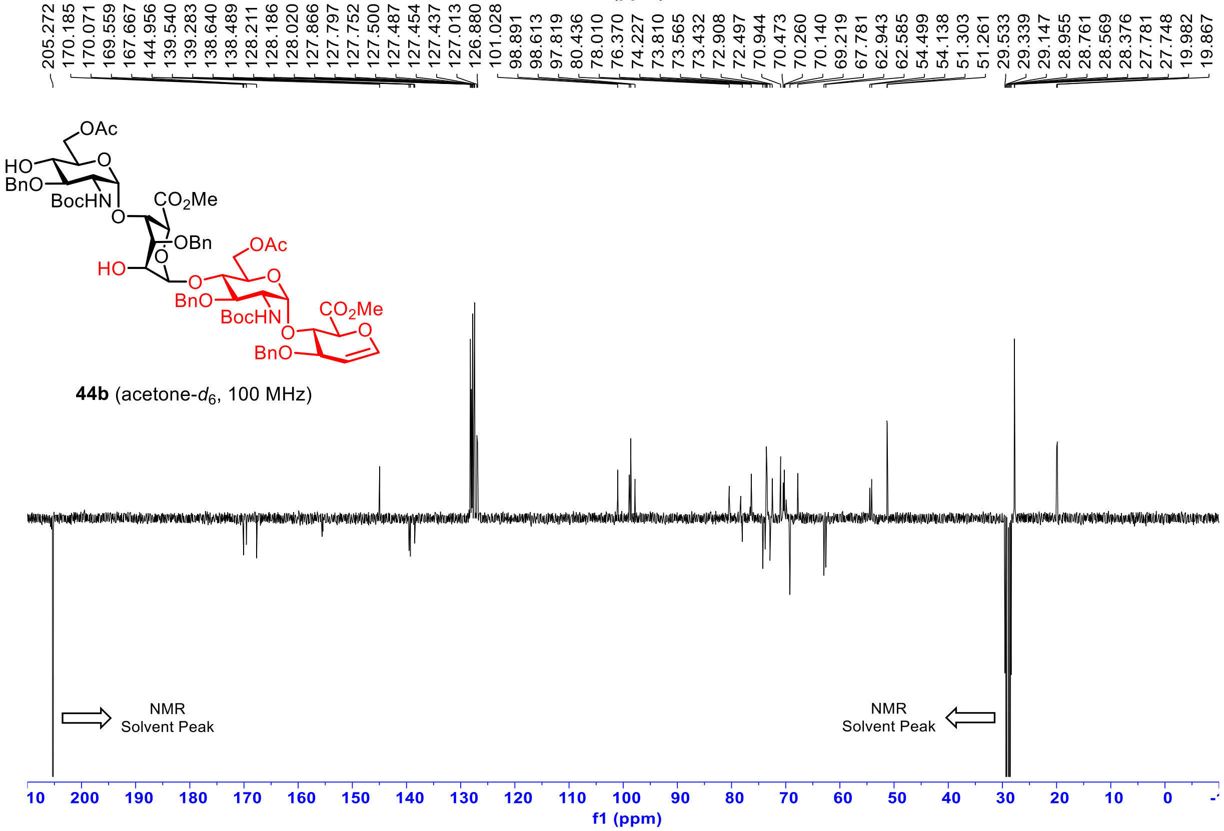
205.272 170.185 170.071 169.559 167.667 144.956 139.540 139.283 138.640 138.489 128.211 128.186 128.020 127.866 127.797 127.752 127.500 127.487 127.454 127.437 127.013 126.880 101.028 98.891 98.613 97.819 80.436 78.010 76.370 74.227 73.810 73.565 73.432 72.908 72.497 70.944 70.473 70.260 70.140 69.219 67.781 62.943 62.585 54.499 54.138 51.303 51.261 29.533 29.339 29.147 28.955 28.761 28.569 28.376 27.781 27.748 19.982 19.867
44b (acetone-d_6, 100 MHz)
NMR Solvent Peak
NMR Solvent Peak
f1 (ppm)

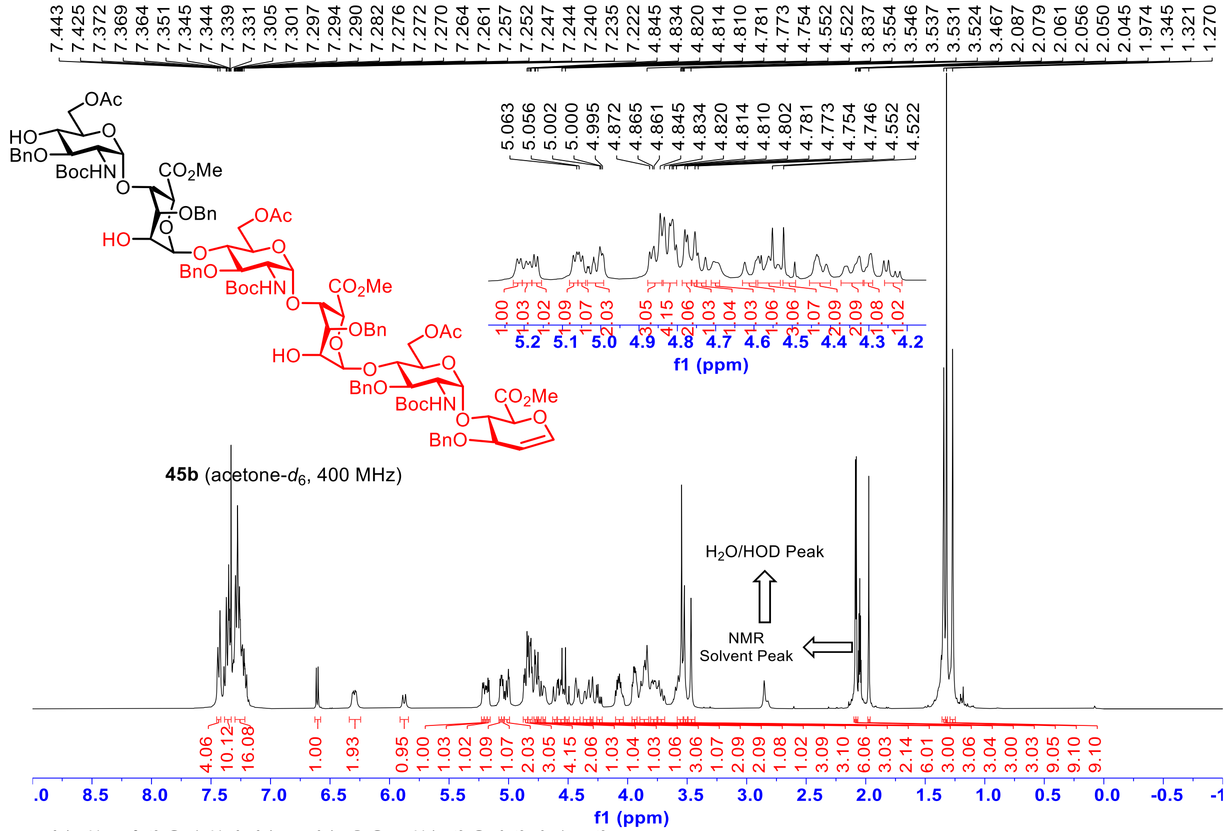
45b (acetone-d_6, 400 MHz)
H2O/HOD Peak
NMR
Solvent Peak
f1 (ppm)

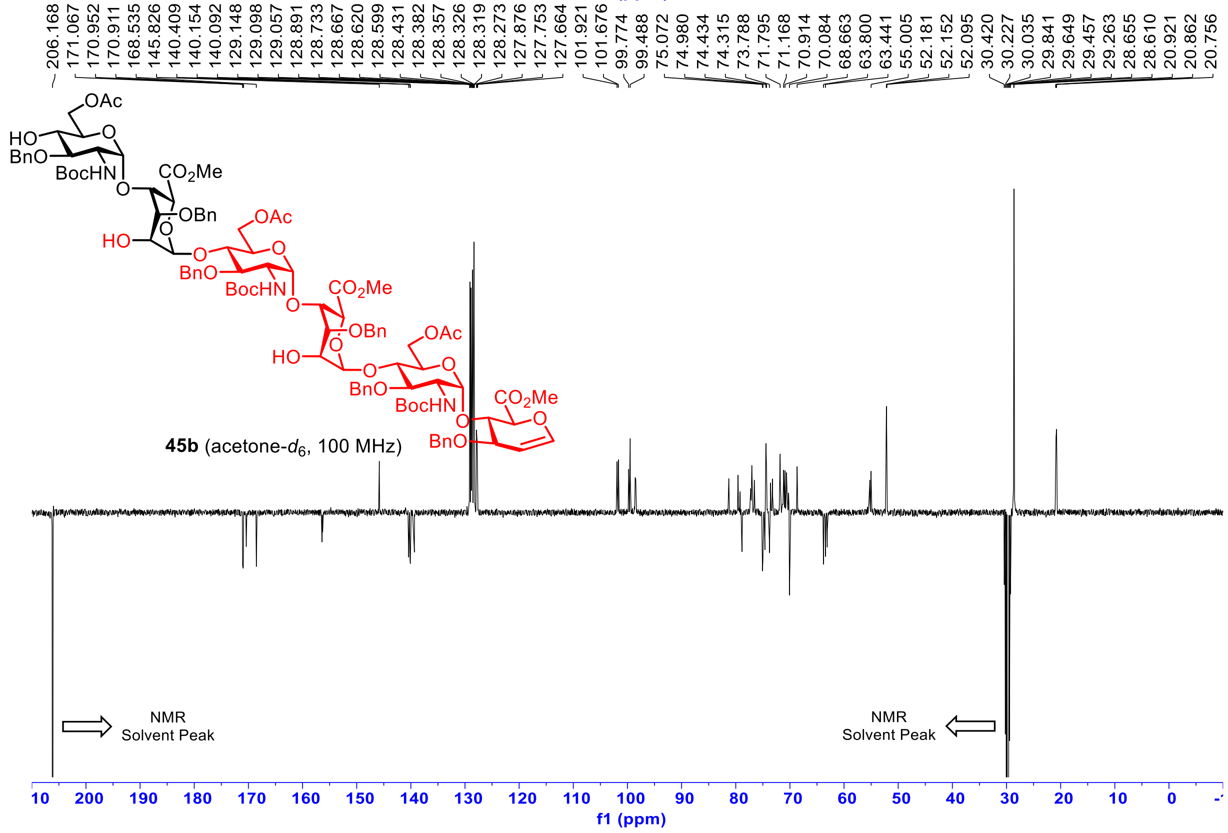
45b (acetone-d_6, 100 MHz)
NMR
Solvent Peak
NMR
Solvent Peak
f1 (ppm)

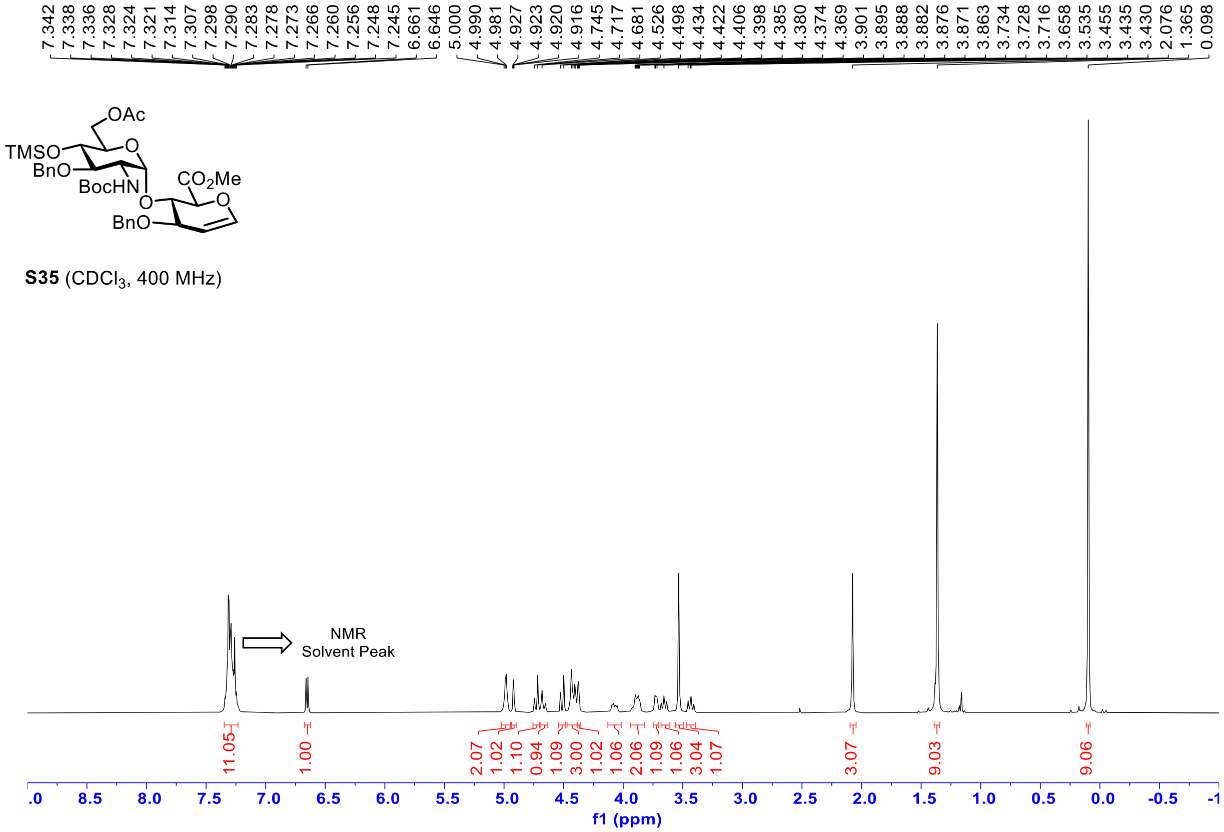
7.342 7.338 7.336 7.328 7.324 7.321 7.314 7.307 7.298 7.290 7.283 7.278 7.273 7.266 7.260 7.256 7.248 7.245 6.661 6.646 5.000 4.990 4.981 4.927 4.923 4.920 4.916 4.745 4.717 4.681 4.526 4.498 4.434 4.422 4.406 4.398 4.385 4.380 4.374 4.369 3.901 3.895 3.888 3.882 3.876 3.871 3.863 3.734 3.728 3.716 3.658 3.535 3.455 3.435 3.430 2.076 1.365 0.098
OAc
TMSO
BnO
BocHN
CO_2Me
O
BnO
S35 ($CDCl_3$, 400 MHz)
NMR
Solvent Peak
11.05
1.00
2.07
1.02
1.10
0.94
1.09
3.00
1.02
1.06
2.06
1.09
1.06
3.04
1.07
3.07
9.03
9.06
f1 (ppm)

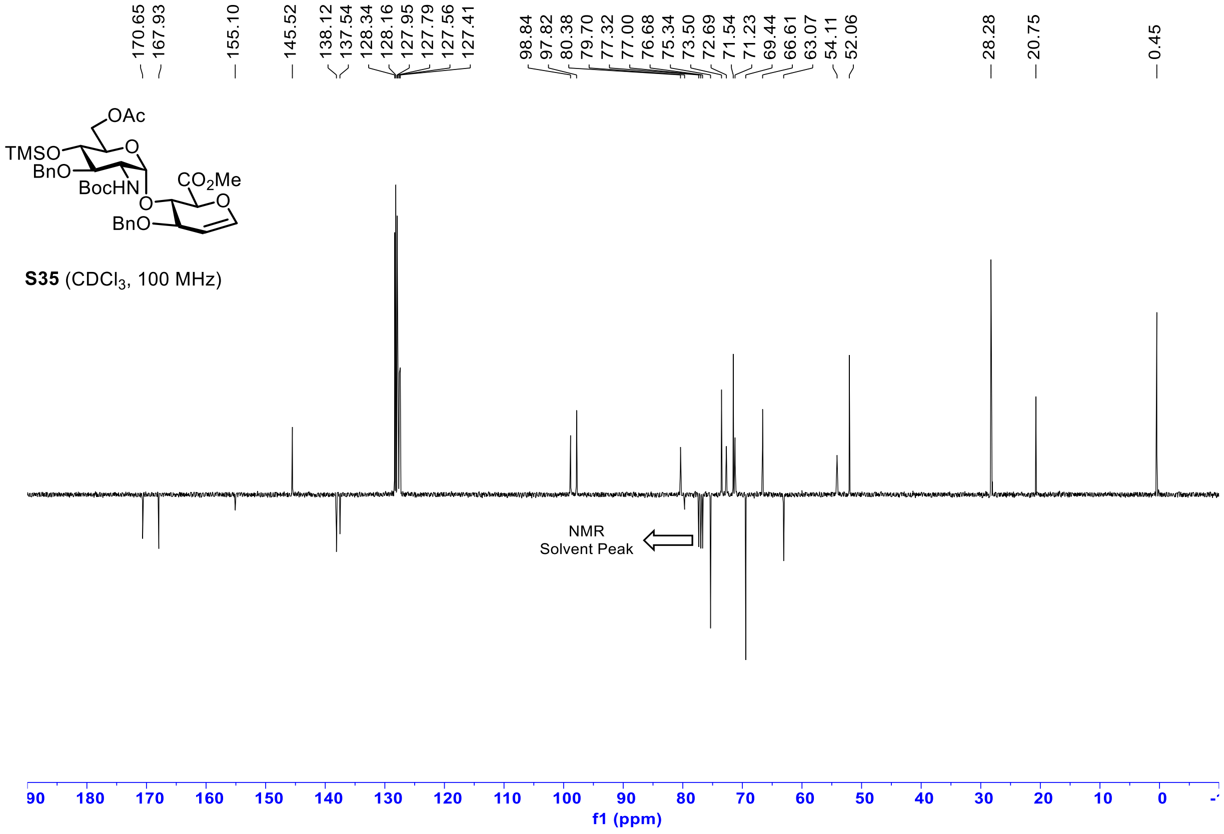
170.65 167.93 155.10 145.52 138.12 137.54 128.34 128.16 127.95 127.79 127.56 127.41 98.84 97.82 80.38 79.70 77.32 77.00 76.68 75.34 73.50 72.69 71.54 71.23 69.44 66.61 63.07 54.11 52.06 28.28 20.75 0.45
OAc
TMSO
BnO
BocHN
CO_2Me
O
BnO
S35 ($CDCl_3$, 100 MHz)
NMR
Solvent Peak
f1 (ppm)

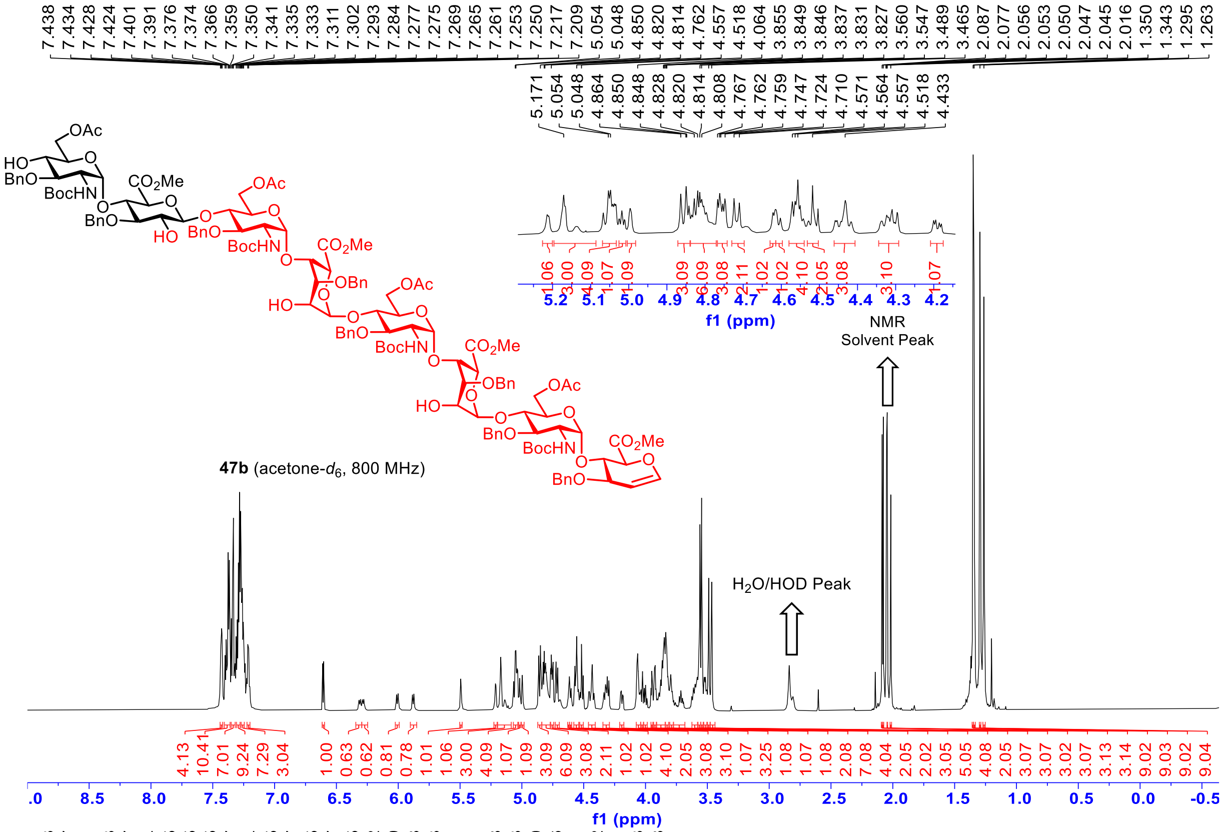
47b (acetone-d_6, 800 MHz)
NMR Solvent Peak
H_2O/HOD Peak
f1 (ppm)

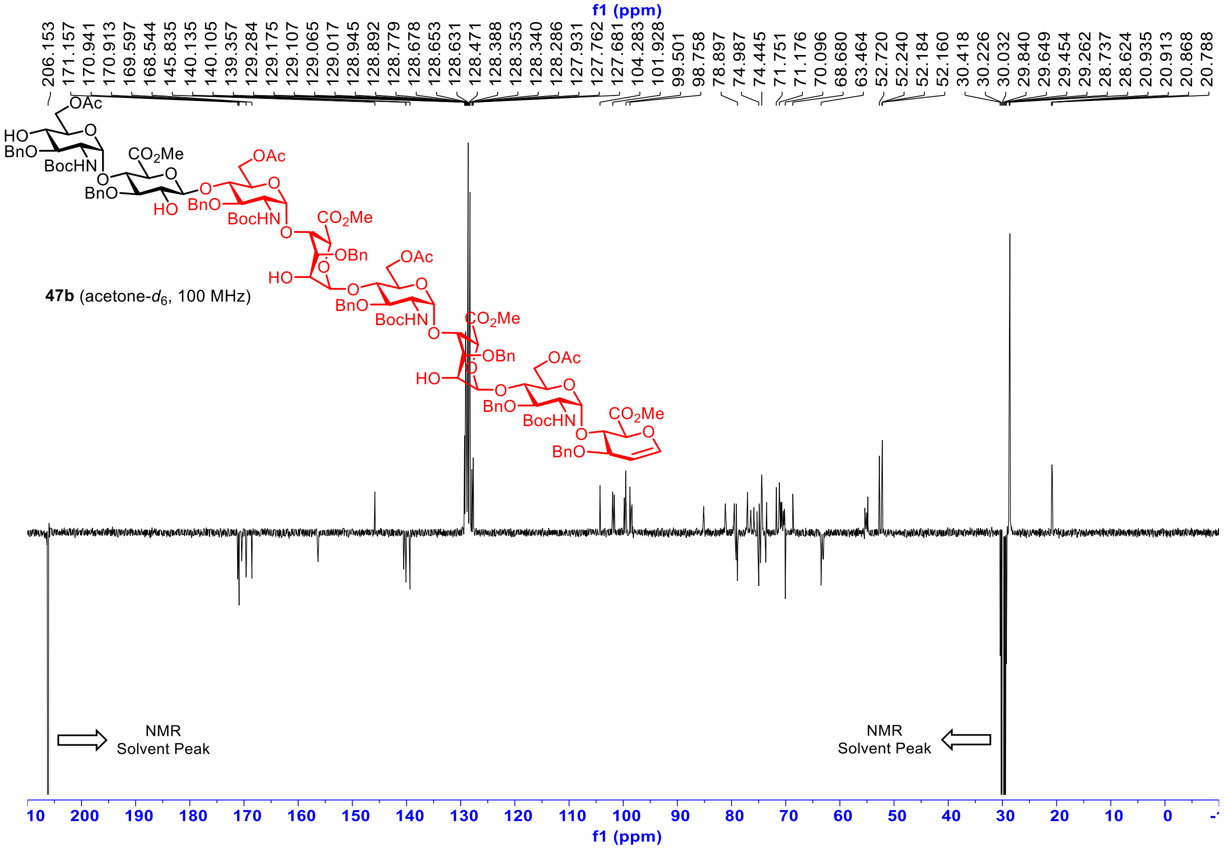
47b (acetone-d_6, 100 MHz)
NMR Solvent Peak
NMR Solvent Peak
f1 (ppm)

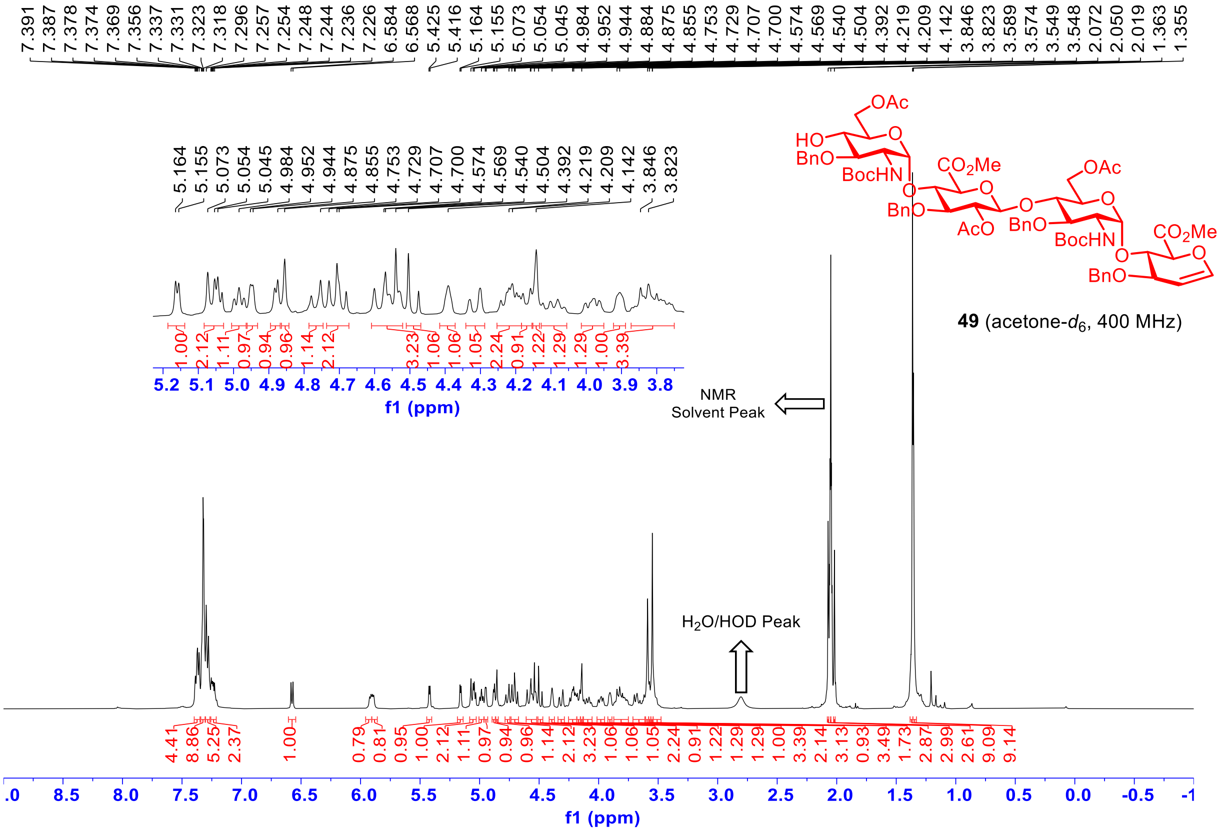
49 (acetone-d_6, 400 MHz)
NMR Solvent Peak
H_2O/HOD Peak
f1 (ppm)

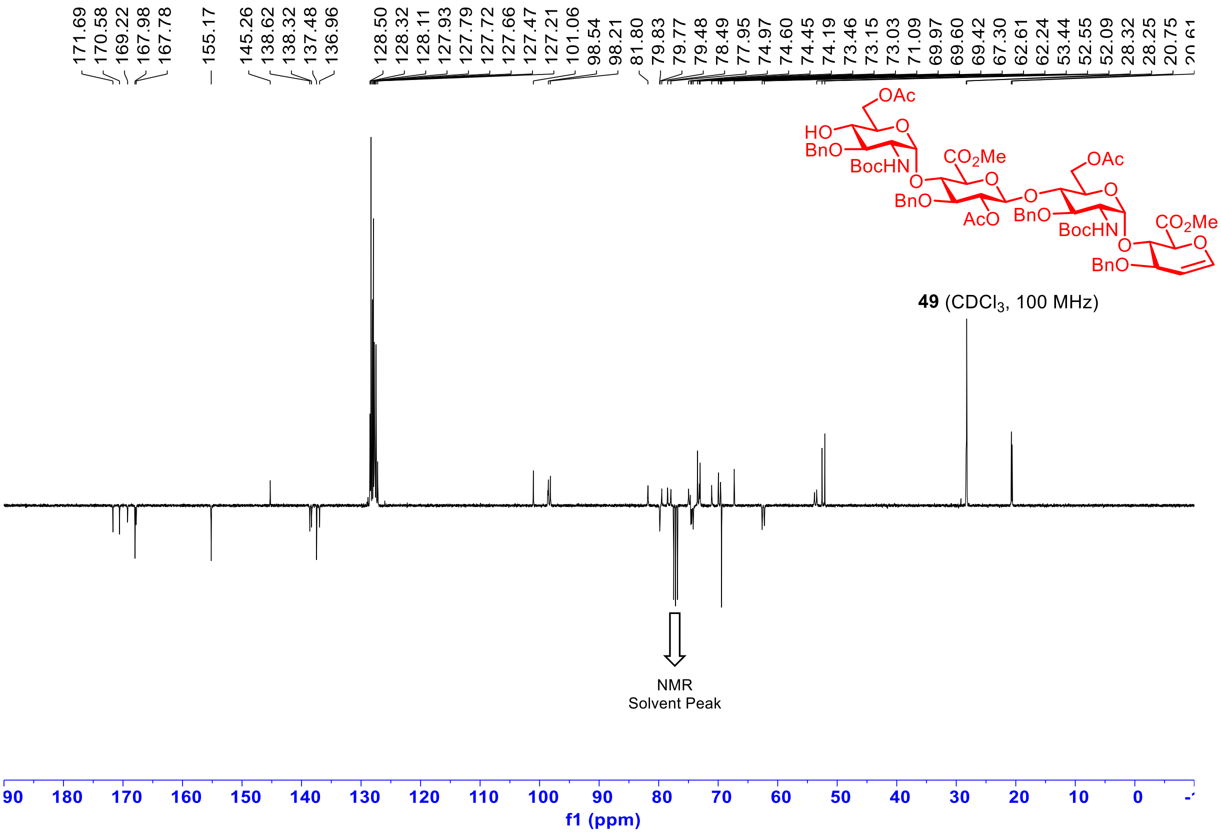
49 ($CDCl_3$, 100 MHz)
NMR Solvent Peak
f1 (ppm)

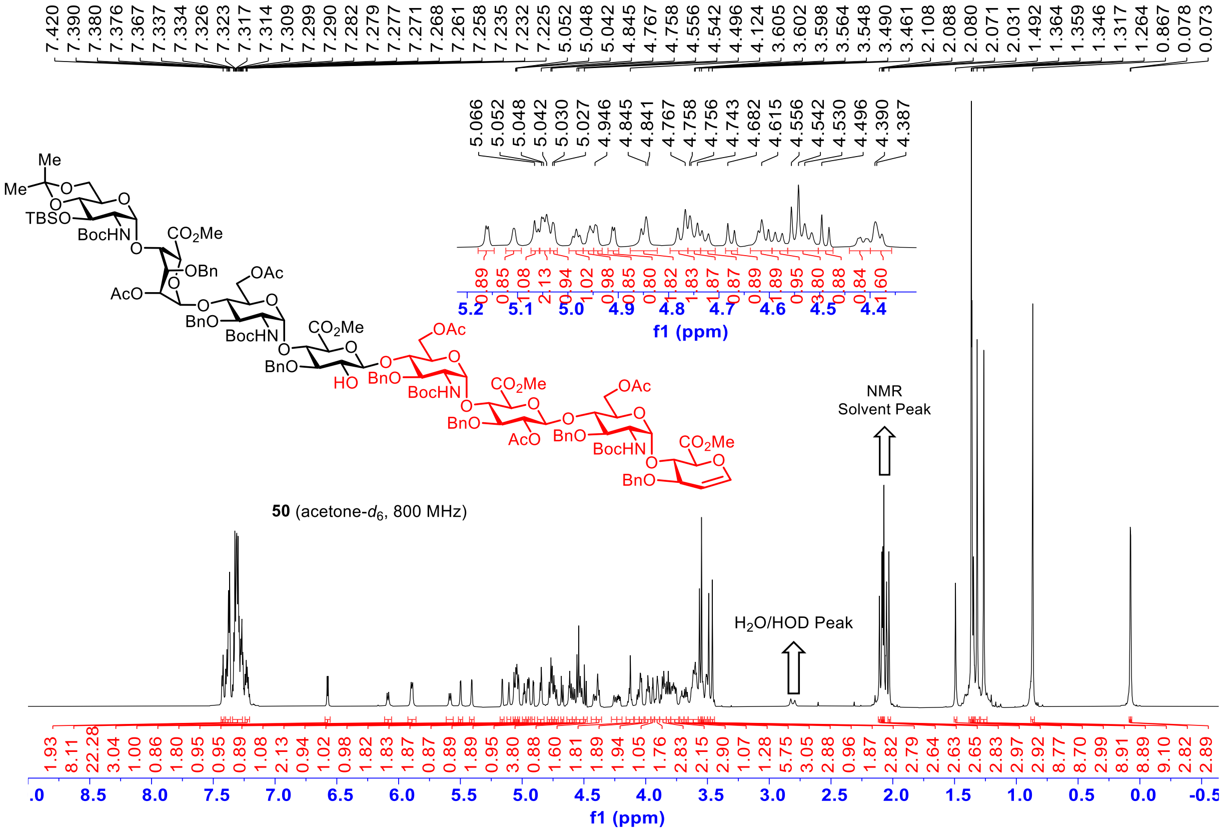
7.420 7.390 7.380 7.376 7.367 7.337 7.334 7.326 7.323 7.317 7.314 7.309 7.299 7.290 7.282 7.279 7.277 7.271 7.268 7.261 7.258 7.235 7.232 7.225 5.052 5.048 5.042 4.845 4.767 4.758 4.556 4.542 4.496 4.124 3.605 3.602 3.598 3.564 3.548 3.490 3.461 2.108 2.088 2.080 2.071 2.031 1.492 1.364 1.359 1.346 1.317 1.264 0.867 0.078 0.073
5.066 5.052 5.048 5.042 5.030 5.027 4.946 4.845 4.841 4.767 4.758 4.756 4.743 4.682 4.615 4.556 4.542 4.530 4.496 4.390 4.387
f1 (ppm)
NMR Solvent Peak
H2O/HOD Peak
50 (acetone-d6, 800 MHz)
1.93 8.11 22.28 3.04 1.00 0.86 1.80 0.95 0.95 0.89 1.08 2.13 0.94 1.02 0.98 1.82 1.83 1.87 0.87 0.89 1.89 0.95 3.80 0.88 1.60 1.81 1.89 1.94 1.05 1.76 2.83 2.15 2.90 1.07 1.28 5.75 3.05 2.88 0.96 1.87 2.82 2.79 2.64 2.63 2.65 2.83 2.97 2.92 8.77 8.70 2.99 8.91 8.89 9.10 2.82 2.89
f1 (ppm)

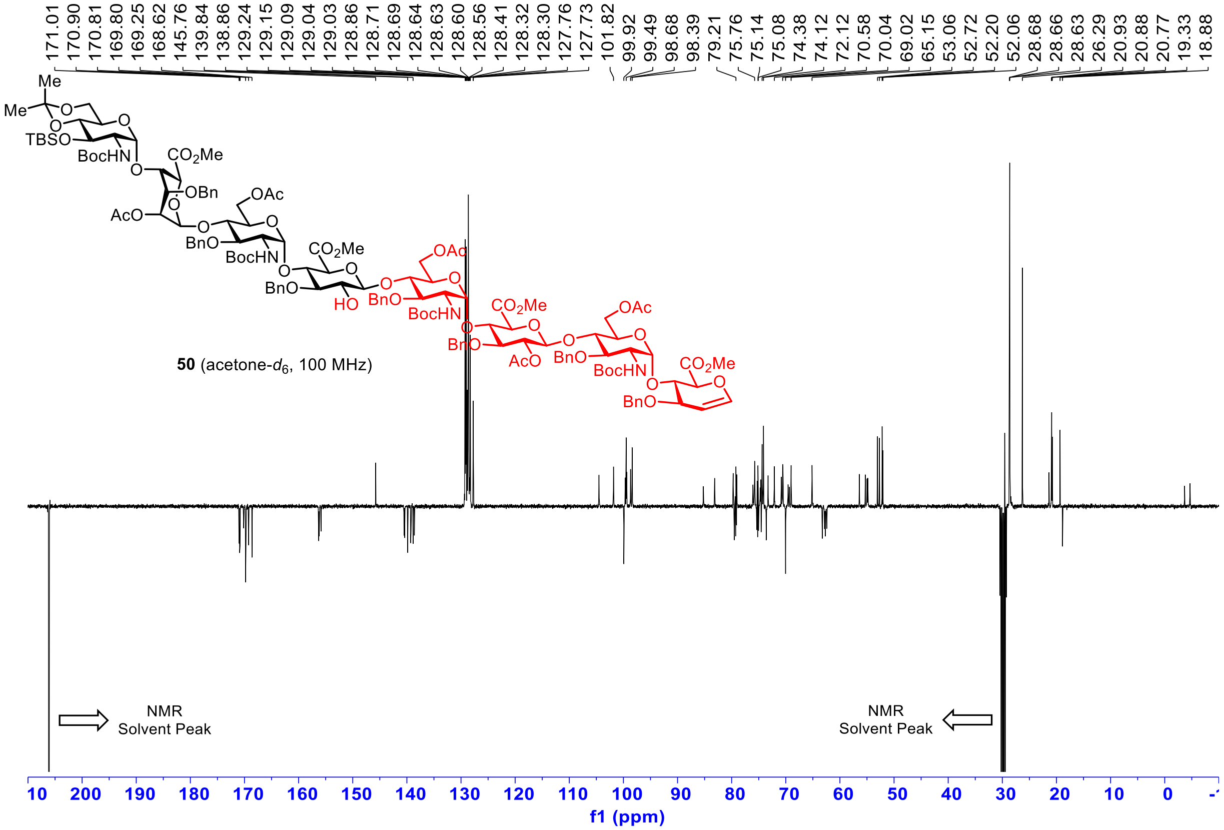
171.01 170.90 170.81 169.80 169.25 168.62 145.76 139.84 138.86 129.24 129.15 129.09 129.04 129.03 128.86 128.71 128.69 128.64 128.63 128.60 128.56 128.41 128.32 128.30 127.76 127.73 101.82 99.92 99.49 98.68 98.39 79.21 75.76 75.14 75.08 74.38 74.12 72.12 70.58 70.04 69.02 65.15 53.06 52.72 52.20 52.06 28.68 28.66 28.63 26.29 20.93 20.88 20.77 19.33 18.88
50 (acetone-d6, 100 MHz)
NMR Solvent Peak
NMR Solvent Peak
f1 (ppm)

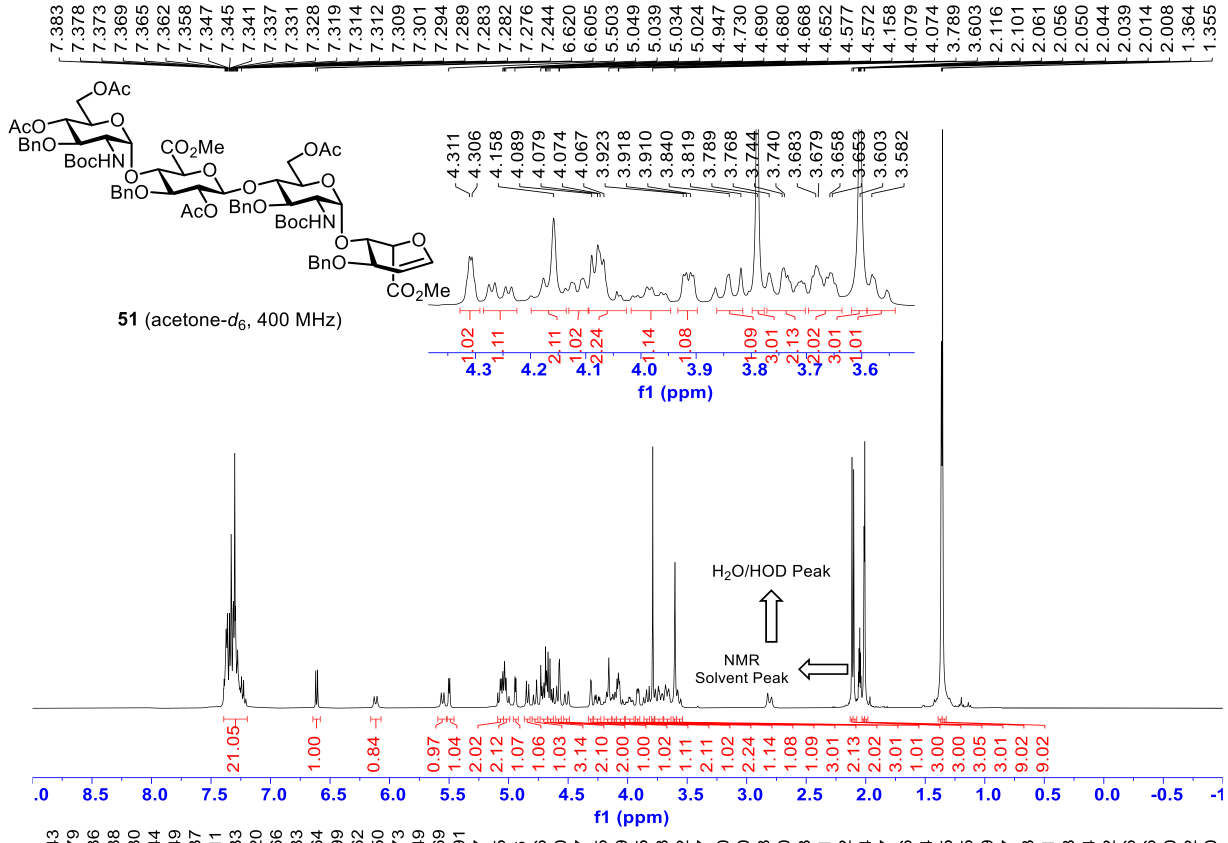
7.383 7.378 7.373 7.369 7.365 7.362 7.358 7.347 7.345 7.341 7.337 7.331 7.328 7.319 7.314 7.312 7.309 7.301 7.294 7.289 7.283 7.282 7.276 7.244 6.620 6.605 5.503 5.049 5.039 5.034 5.024 4.947 4.730 4.690 4.680 4.668 4.652 4.577 4.572 4.158 4.079 4.074 3.789 3.603 2.116 2.101 2.061 2.056 2.050 2.044 2.039 2.014 2.008 1.364 1.355
OAc
AcO
BnO
BocHN
O
CO2Me
BnO
AcO
BnO
BocHN
CO2Me
51 (acetone-d6, 400 MHz)
4.311 4.306 4.158 4.089 4.079 4.074 4.067 3.923 3.918 3.910 3.840 3.819 3.789 3.768 3.744 3.740 3.683 3.679 3.658 3.653 3.603 3.582
1.02 1.11 2.11 1.02 2.24 1.14 1.08 1.09 3.01 2.13 2.02 3.01 1.01
4.3 4.2 4.1 4.0 3.9 3.8 3.7 3.6
f1 (ppm)
H2O/HOD Peak
NMR Solvent Peak
21.05 1.00 0.84 0.97 1.04 2.02 2.12 1.07 1.06 1.03 3.14 2.10 2.00 1.00 1.02 1.11 2.11 1.02 2.24 1.14 1.08 1.09 2.13 2.02 3.01 1.01 3.00 3.00 3.05 3.01 9.02 9.02
.0 8.5 8.0 7.5 7.0 6.5 6.0 5.5 5.0 4.5 4.0 3.5 3.0 2.5 2.0 1.5 1.0 0.5 0.0 -0.5 -1
f1 (ppm)

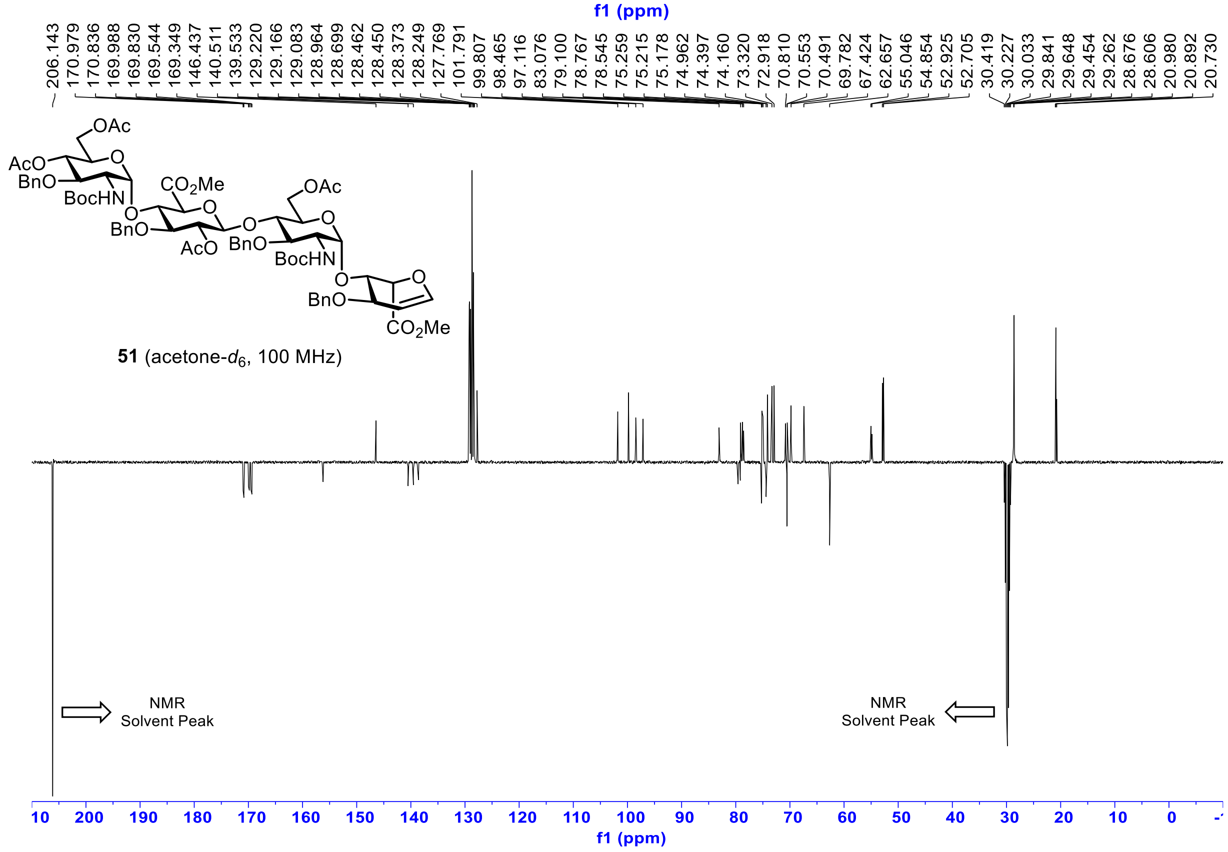
206.143 170.979 170.836 169.988 169.830 169.544 169.349 146.437 140.511 139.533 129.220 129.166 129.083 128.964 128.699 128.462 128.450 128.373 128.249 127.769 101.791 99.807 98.465 97.116 83.076 79.100 78.767 78.545 75.259 75.215 75.178 74.962 74.397 74.160 73.320 72.918 70.810 70.553 70.491 69.782 67.424 62.657 55.046 54.854 52.925 52.705 30.419 30.227 30.033 29.841 29.648 29.454 29.262 28.676 28.606 20.980 20.892 20.730
51 (acetone-d6, 100 MHz)
NMR Solvent Peak
NMR Solvent Peak
10 200 190 180 170 160 150 140 130 120 110 100 90 80 70 60 50 40 30 20 10 0
f1 (ppm)

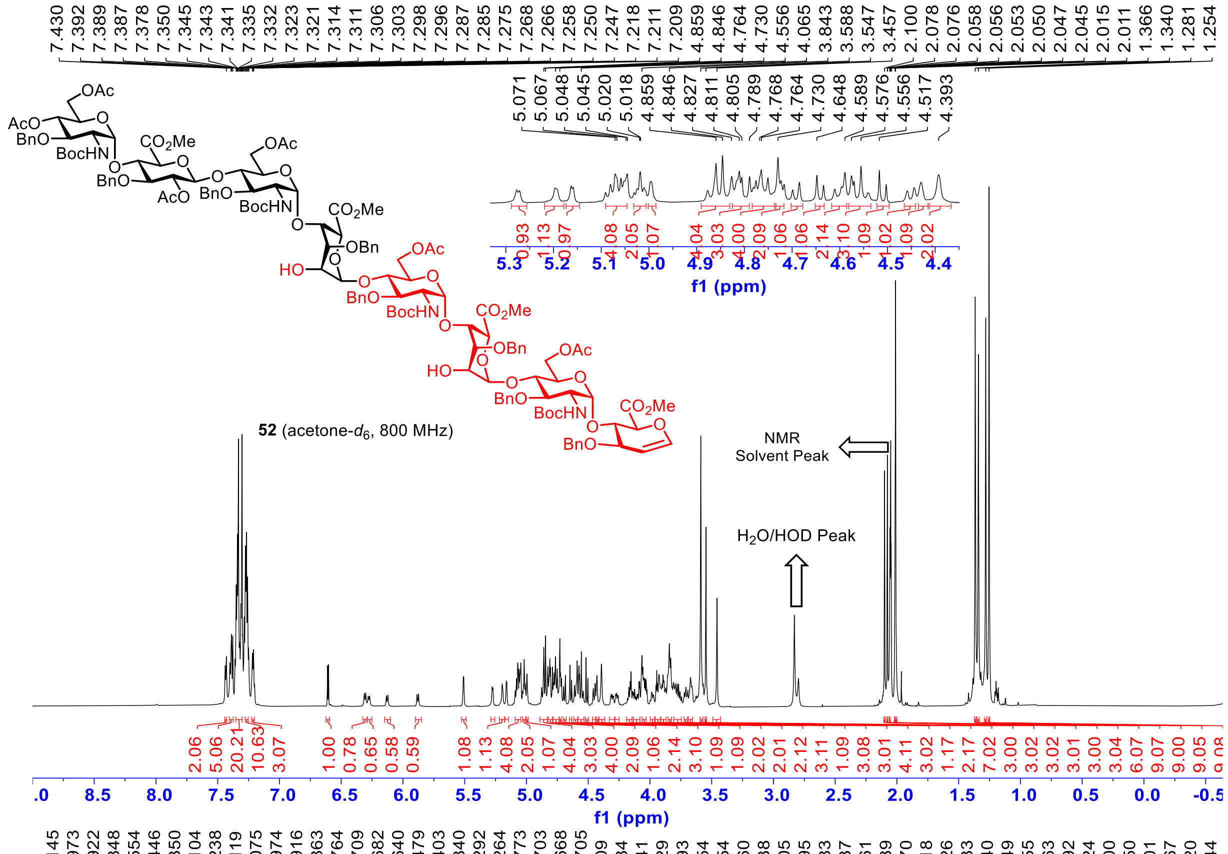
52 (acetone-d_6, 800 MHz)
NMR
Solvent Peak
H_2O/HOD Peak
f1 (ppm)

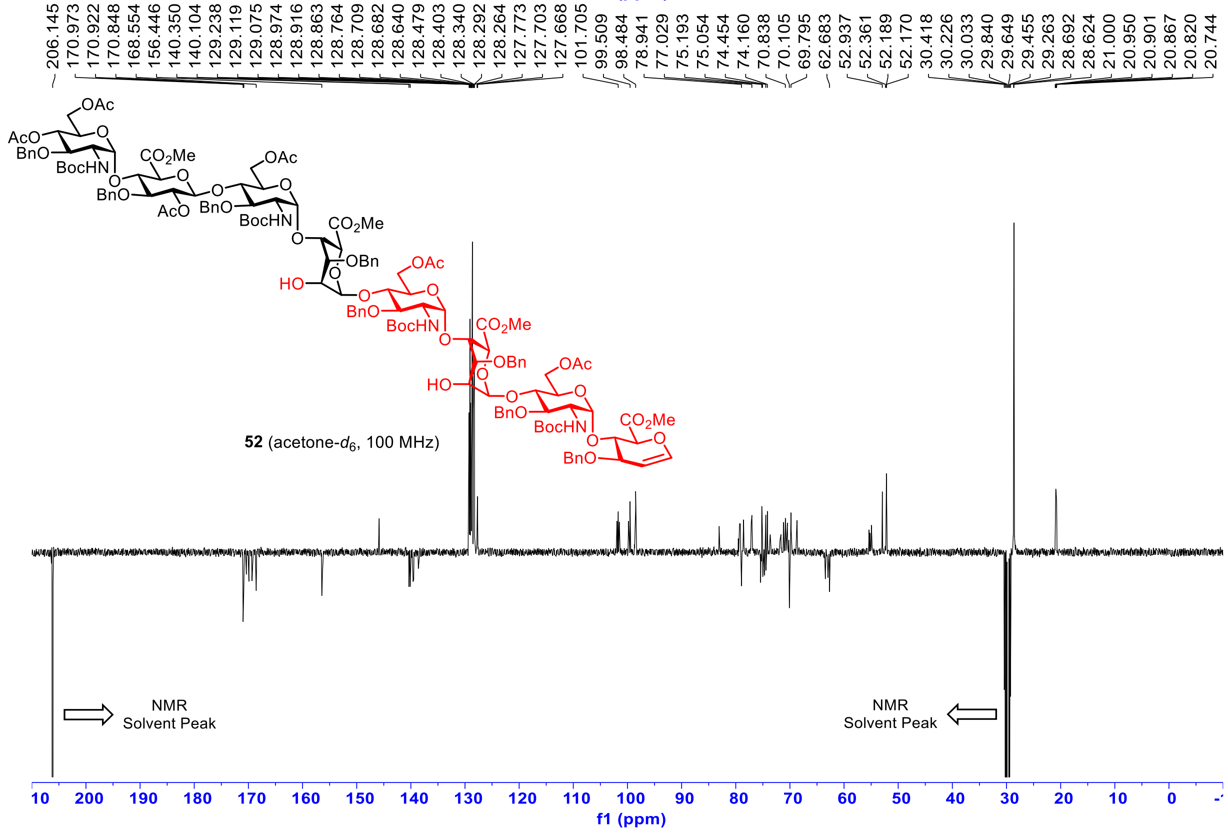
52 (acetone-d_6, 100 MHz)
NMR
Solvent Peak
NMR
Solvent Peak
f1 (ppm)
